# Oxidative Scission of Bicyclo[2.2.2]octenones: Untying the α-Dimethoxycarbonyl

**DOI:** 10.1021/acs.joc.4c02699

**Published:** 2025-01-28

**Authors:** Ting-Zhi Yao, Yi-Cheng Tseng, Jia-Luo Li, Deng-Lian Hou, Gary Jing Chuang

**Affiliations:** Department of Chemistry, Chung Yuan Christian University, Chung-Li 320314, Taiwan

## Abstract

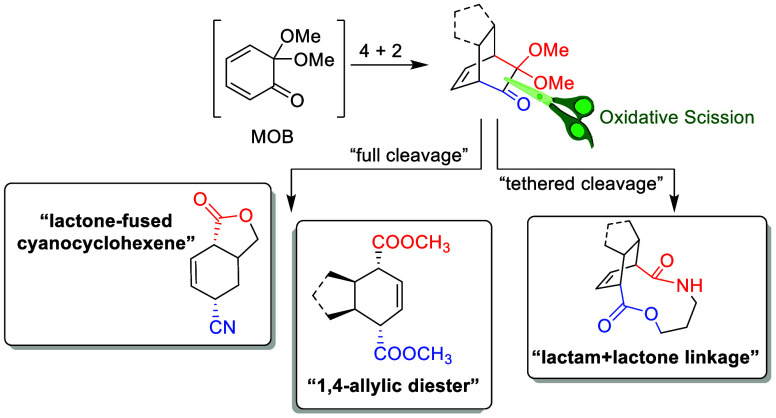

This study explores the selective oxidative scission of bicyclo[2.2.2]octenones derived from masked *o*-benzoquinones (MOBs). By employing the fragmentation of ketoximes and Schmidt-type reactions, we achieved the cleavage of α-dimethoxy carbonyl groups to yield highly functionalized cyclohexene frameworks, showcasing the expanding synthetic utility of bicyclo[2.2.2]octenones in complex organic synthesis. The methodologies developed are anticipated to contribute to advancements in organic and pharmaceutical chemistry.

Organic synthesis involving the rearrangement of one skeletal structure into another has long fascinated synthetic chemists due to the elegance and practicality of the transformation.^1^ By utilizing photoexcitation,^2^ acid–base interaction,^3^ heat,^4^ and more recently photoredox catalysis,^5^ syntheses of many complex structures were achieved through the rearrangement reactions of straightforwardly accessible scaffolds. One example can be found in the synthetic applications of bicyclo[2.2.2]octenones, which could be facilely obtained by the 4 + 2 cycloaddition of masked *o*-benzoquinones (MOBs).^6^ As shown in Scheme 1, the bicyclo[2.2.2]skeleton was transformed into diverse frameworks via side chain-involved sigmatropic rearrangements or photoinduced rearrangement.^7^ The resulting products consist of a variety of structures, such as *cis*-decalin, diquinane, bicyclo[3.2.1]octanone, and fused-5,6,3-tricyclic skeletons. These fruitful examples have proven the utility of densely functionalized bicyclo[2.2.2]octenones and driven us toward further exploration.

**Scheme 1:**
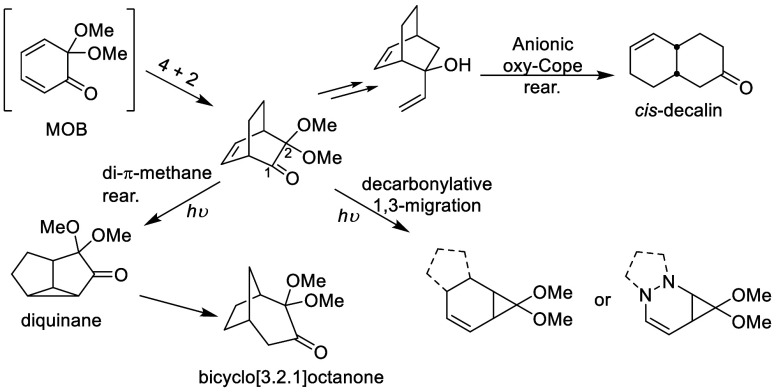
Examples of Rearrangement Products from Bicyclo[2.2.2]octenone

A common feature in the previously mentioned reports about the synthetic applications of bicyclo[2.2.2]octenone is that the α-dimethoxycarbonyl is the key functional group during the rearrangement, especially in the photoinduced processes. However, there have been no reports on the selective oxidative scission at C1–C2 in the presence of olefin in this particular system. It is well recognized that oxidative rearrangement reactions have played a significant role in organic synthesis, combining the transformative power of oxidation with the structural reorganization of rearrangements.^8^ These reactions enable the simultaneous introduction of carbonyl functionalities and the migration of atoms or groups within a molecule, leading to the efficient synthesis of complex, functionally diverse products. Hence, as the extension of our effort in the development of the photoinduced rearrangement of the bicyclo[2.2.2]octenone system and its application in natural product synthesis,^9^ we wish to present our recent progress on the oxidative scission of bicyclo[2.2.2]octenones. By utilizing Beckmann-type fragmentation reactions, hypervalent iodine-mediated oxidation, and the Schmidt reaction, the α-dimethoxy carbonyl functional groups were cleaved to provide different frameworks with a highly functionalized cyclohexene core from the original 4 + 2 adduct of masked *o*-benzoquinone.

As a starting point, we chose the “intramolecular D-A adducts” of MOBs for the synthesis of the corresponding ketoximes,^10^ which later participate as the platform for Beckmann rearrangement/fragmentation.^11^ As shown in Scheme 2, MOBs obtained from the oxidation of 2-methoxyphenols with the presence of alkenol proceeded via an intramolecular D–A reaction, and the resulting cycloaddition adducts **2a** was set to react with hydroxyamine to yield the corresponding α-ketalketoxime of bicyclo[2.2.2]octenones, **3a**. Additionally, when the acidic condition was applied to the ketoxime, we did not observe Beckmann rearrangement, which theoretically cleaves the C_1_–C_2_ bond by inserting a nitrogen to form a lactam ring. Instead, an interesting lactone product with a cyano group was isolated in good yield,^12^ which is likely the result of a Beckmann fragmentation-type reaction. The relative stereochemistry at the fused carbons and the allylic cyano group was further confirmed by X-ray analysis.

**Scheme 2:**
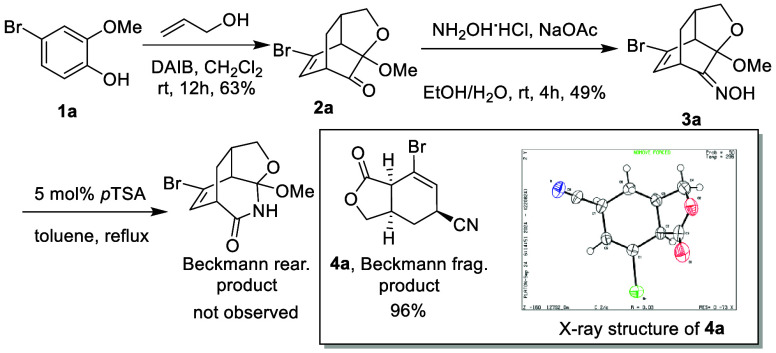
Fragmentation of Ketoxime **3a** to Form Lactone **4a** ORTEP drawing with thermal ellipsoids drawn at 50% probability.

After confirming the structure of the product, we looked into the generality of this oxidative scission. As shown in Table 1, basic model compound **3b** yielded **4b** in 73% yield. Substrates with methyl groups at different positions in the examples of **4d**, **4g**, and **4i** showed reasonable compatibility, giving 43–58% isolated yields. The bromo group was tolerated in the case of the aforementioned **4a**, as well as in the cases of **4c** and **4i**–**k**. TMS and allyl at the original olefin on the ketoximes reacted smoothly to produce **4e** and **4f** in 88% and 84% yields, respectively. As compared to other positions, it should be noted that substituents at the ketal neighboring bridgehead have little effect on the product yield (**4g** and **4h**). Additionally, fused δ-lactone product **4k** was also synthesized from this transformation, although at a lower yield of 33%.

**Table 1:**
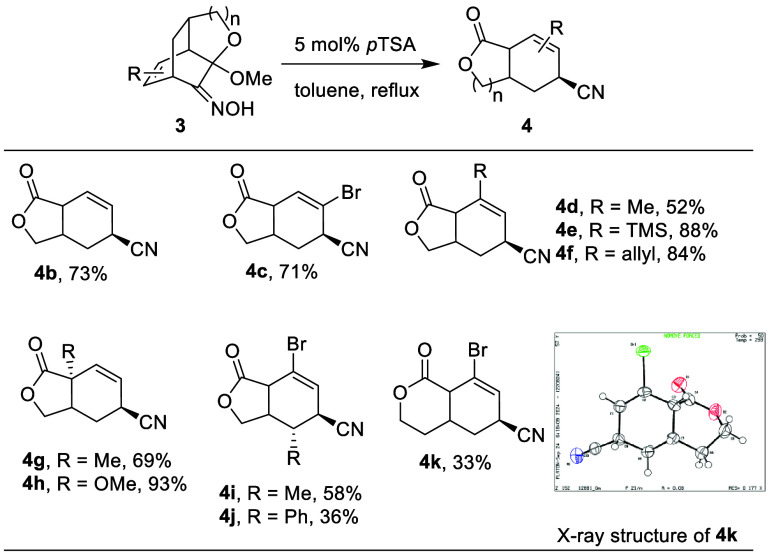
Scope of Ketoxime Fragmentation to Yield Lactone

After this encouraging result, we then explored the reactivity of ketoximes derived from the intermolecular D–A adducts of MOBs. As shown in Scheme 3, oxime formation was carried out under similar conditions. However, exposure of the resulting α-ketalketoxime **6a** with acidic conditions only induced the hydrolysis of the ketal group. Neither the Beckmann rearrangement nor the fragmentation product was observed. Fortunately, utilizing the methodology pioneered by Ciufolini,^13a^ the use of a hypervalent iodine oxidant under acidic conditions promoted the cleavage of oxo-ketoxime **7a** to form a diester with the presence of an olefin, and compound **8a** was isolated in 54% yield.^13^

**Scheme 3:**
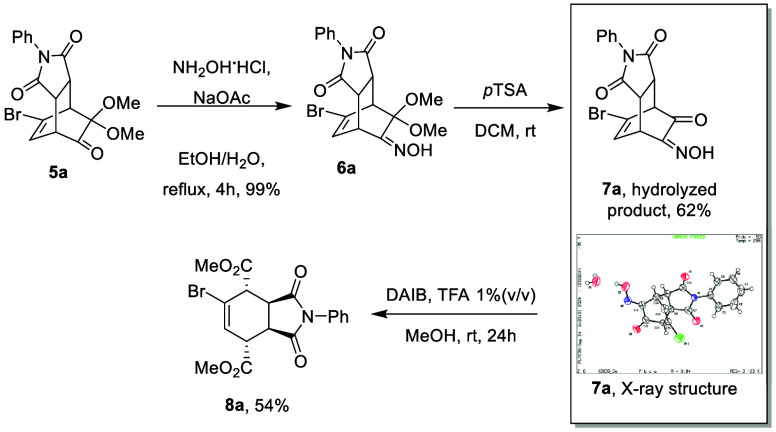
Oxidative Cleavage of α-Oxo-ketoxime **7a** ORTEP drawing with thermal ellipsoids drawn at 50% probability.

Following the finding of the previously mentioned sequential hydrolysis of α-ketalketoxime and oxidative cleavage, we then explored the scope of this method using a variety of ketoximes derived from the D–A adducts of MOBs. As shown in Table 2, **6a**–**j** are examples derived from D–A adducts of *N*-phenylmaleimide and MOBs. The hydrolysis of dimethoxy ketal proceeded with 48–99% yields with the presence of imide and oxime moieties. Ketal protection on the aldehyde (**7d** and **7i**) and ester (**7j**) was also compatible in this acidic condition. As for the target oxidative cleavage, parent molecule **8b** was isolated in 44%yield, and examples with alkyl, allyl, and TMS substitutions at the olefin resulted in products **8c** and **8e**–**g** in 31–62% yields. It is worth noting that because of the symmetrical structure of cleaved product, **7c** and **7g** actually produce the identical products **8c** and **8g**. For the same reason, ketal-substituted **7d** and **7i** gave the same products **8d** and **8i** in 40% and 20% yields, respectively, and bromo compound **8a** (**8h**) was obtained from the reaction of **7h** in 45% yield. We found the electron-withdrawing ester group in the example of **7j** is not compatible with the oxidation and resulted in a messy mixture. Also, substrates with a methyl or methoxy at the bridgehead position seem to have a negative effect on the reaction, as a complex mixture was found in the oxidative cleavage of **7k**, and 21% yield for **8l** was found in the reaction of **7l**. Ketoximes **6m**–**o** with methyl substitutions were found to be compatible in the hydrolysis/oxidative cleavage sequence, giving functionalized *cis*-hydrindane analogues **8m**–**o**. Simpler bicyclo[2.2.2]octene-type structures were also examined, as D–A adducts made from styrene and MOBs were converted to **7p**–**r** in yields of 79–99% in the step of hydrolysis; the subsequent conversion into 3,6-diestercyclohexenes gave **8p**–**r** in moderate yields of 46–62%.

**Table 2:**
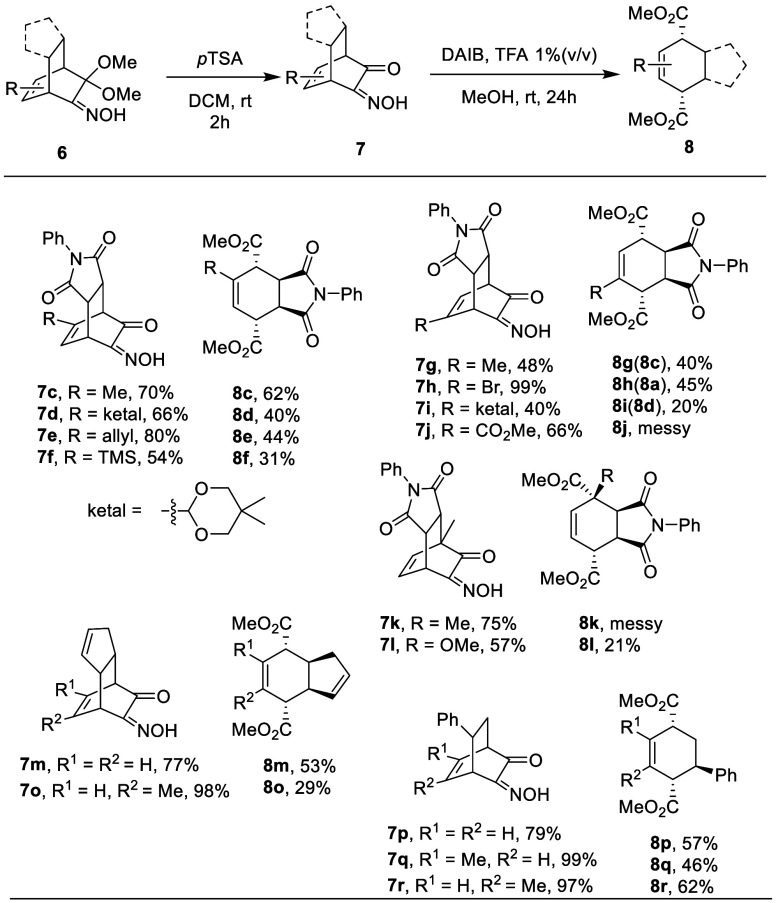
Scope of Hydrolysis/Oxidative Cleavage of Ketoxime **6**

Due to the unique reactivity that we found at the α-dimethoxyketone, we wish to further explore the scope of oxidative cleavage at this particular moiety. Since the original proposed nitrogen insertion by Beckmann rearrangement actually afforded the aforementioned fragmentation, we wish to utilize the Schmidt -type reaction of the target D–A adducts.^14^ Known for its capability of inserting a nitrogen into a carbonyl moiety, this interesting reactivity originating from hydrazoic acid and then alkyl azide was extensively explored by Aubé and others.^15,16^ As shown in Scheme 4, we employed the Schmidt reaction on **5c** with azidoalcohol under acidic conditions.^17^ Intriguingly, instead of obtaining a nitrogen-inserted product on either side of the carbonyl group, two isomers resulting from ring expansion, **9c** and **9c′**, were isolated. The structure of the enlarged ring is particularly interesting due to the construction of lactone-lactam linkage in a single reaction, as it could be described as an oxidative cleavage while tethering the two ends of the carbonyls by ester and amide bonds.

**Scheme 4:**
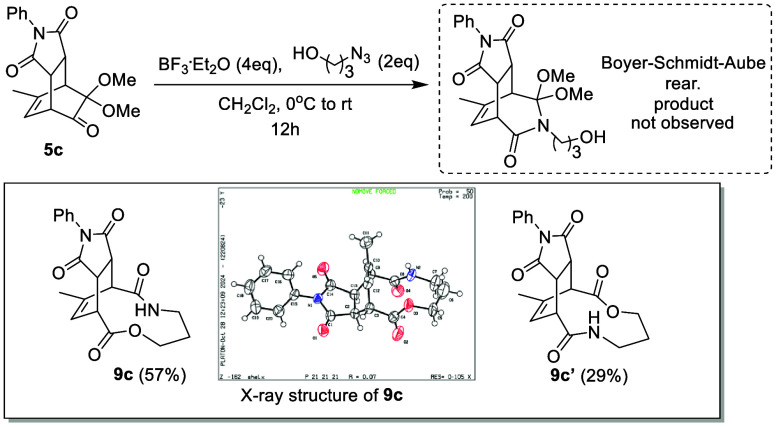
Ring Epansion of **5c** to Form Lactone-Lactam Linked **9c** ORTEP drawing with thermal ellipsoids drawn at 50% probability.

As the structure of the product resulting from treating **5c** with azidopropanol was unfolded, we proceeded to explore the substrate scope of this “tethered oxidative cleavage” of the α-dimethoxyketone on bicyclo[2.2.2]octenones. Shown in Table 3, D–A adduct **5b** resulted in **9b** in 84% yield. When an electron-withdrawing substituent bromo group or methyl ester was presented on the substrate, the yields of the products were found to be lower as in the examples of **9a/9a′** (17%, 1:1), **9j**/**9j′** (33%, 1:3), and **9s/9s′** (32%, 2:1). The ketal on **5d** was found to be incompatible in the unsuccessful construction of **9d** under relatively acidic conditions. The allyl substituent showed reasonable compatibility, as **9e/9e′** was obtained as a 2:1 mixture in 70% yield. **5g** gave an expected mixture of **9g**/**9g′** (identical to **9c′**/**9c**), similar to the reaction of **5c**. Bridgehead substitutions have shown a noticeable effect, where the methyl or methoxy adjacent to the carbonyl (**9t** and **9u**) has a negative impact on the reaction and gives a complex mixture when compared to the same substitution on the opposite bridgehead (**9k** and **9l**). D–A adducts made from cyclopentadiene with MOB are also compatible in this chemoselective tethered oxidative cleavage with the presence of two olefins, yielding **9n** and **9v** in 56% and 64% yields, respectively. Furthermore, bicyclo[2.2.2]octenones made from styrene with MOBs were also examined, as **9p**, **9q**, **9w**, and **9x** were isolated in 50–61% yields. Lastly, we tested the effect of carbon chain length in the azidoalcohol. 2-Azidoethanol was found to be reactive, giving a smaller ring expansion to yield **9y** in 47% yield. However, the reaction using 4-azidobutanol failed to produce **9z**.

**Table 3:**
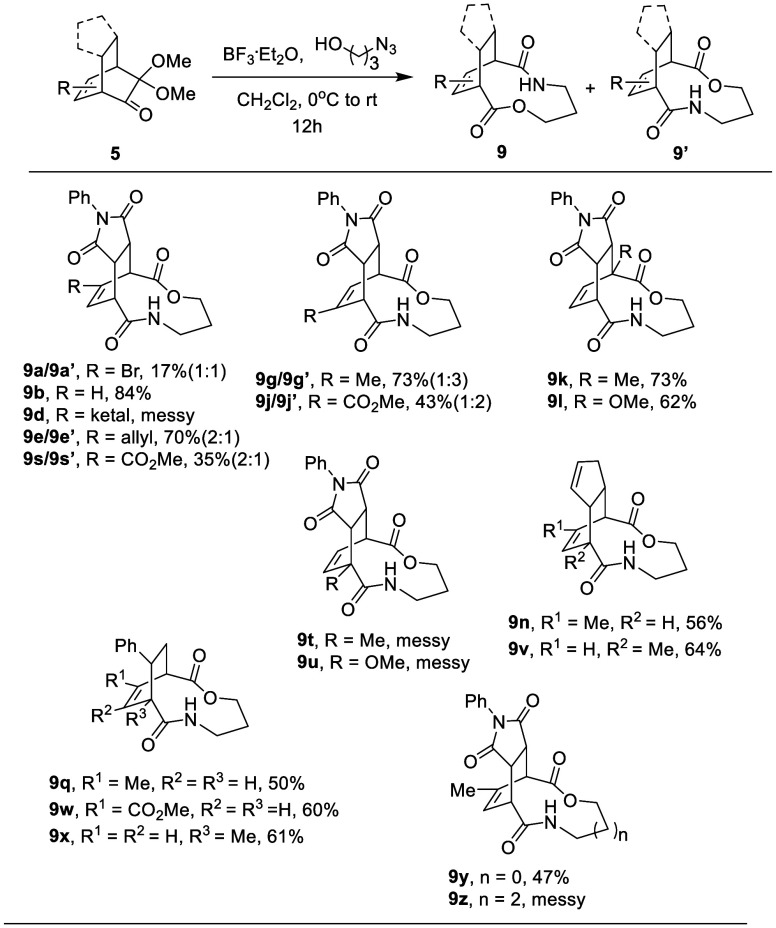
Scope of Tethered Oxidative Cleavage from Insertion of Azidopropanol to **5**

After we established the presented three methods of performing chemoselective oxidative scission on the bicyclo[2.2.2]octenones, the α-dimethoxyketone was clearly important to these unique reactivities, which has driven the target reactions into different outcomes from the original versions with a simpler ketone moiety. In Scheme 5, we disclose the proposed mechanism involved in the presented transformations. In part A of Scheme 5, acidic conditions promoted the leaving of oxime’s hydroxy group on **IA**, and the adjacent cyclic acetal group facilitated the accompanying cleavage of the C–C bond by forming oxonium intermediate **IIA**. Subsequent hydrolysis of **IIIA** gave the formation of the target lactone. As for the α-ketalketoxime in part B, we suspect that without the cyclic structure the acetal was more prone to hydrolyze under acidic conditions. The resulting α-ketoketoxime **7** is more electron-deficient and does not undergo the fragmentation. Following oxidative cleavage to form nitrile oxide/ester and subsequent conversion to ester/ester on both ends, oxidative carbon cleavage was performed one-pot by the hypervalent iodine oxidant. In the case of the tethered oxidative cleavage of **5**, an acid-catalyzed Schmidt reaction proceeded with the azidoalcohol, the hydroxy group of which facilitates the conversion of **IC** to **IIC** through an intermolecular reaction. Subsequently, the 1,2-shift of the acetal’s carbon to the nitrogen with extrusion of N_2_ forms **IIIC**. Instead of continuing on the path of Schmidt by hydrolysis, the acetal provided the elimination of methoxide on intermediate **IVC**, breaking the C–N bond and eventually furnishing the expanded ring with lactone and lactam linkages.

**Scheme 5:**
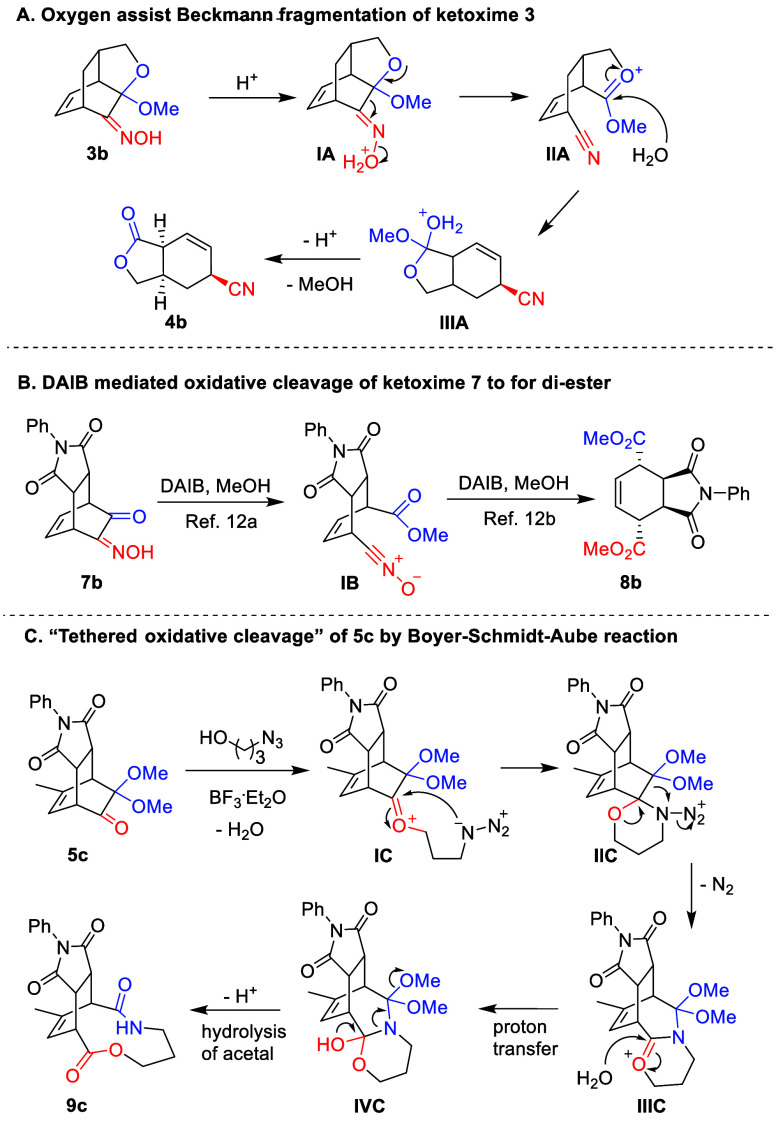
Proposed Reaction Pathway for the Presenting Methods of Oxidative Scission on Bcyclo[2.2.2]octenone Derivatives

In conclusion, we have developed three methods of achieving the oxidative scission of the C–C single bond at the α-dimethoxyketone on bicyclo[2.2.2]octenone skeletons, which could be obtained with structural diversity via the cycloaddition of MOBs. The oxidative cleavages were chemoselective, leaving the olefin intact for further functionalization.Additionally, the cleaved carbonyls were transformed into lactone/nitrile, diesters, and lactam-lactone moieties, providing a variety of functional groups on the cyclohexene products. We anticipate that this methodology would further increase the potential of the bicyclo[2.2.2]skeleton in organic and pharmaceutical synthesis.

## Data Availability

The data underlying this study are available in the published article and its Supporting Information.

## Supporting Information Available

Experimental procedures, NMR spectra, and crystallographic data (PDF)
