# Supplementary material for: Oxidative Scission of Bicyclo[2.2.2]octenones: Untying the α-Dimethoxycarbonyl
Source: J Org Chem. 2025 Jan 28;90(5):2126–31. doi: 10.1021/acs.joc.4c02699 (PMC11812015; doi:10.1021/acs.joc.4c02699)
Supplement: Supplementary file 1 — jo4c02699_si_001.pdf [file jo4c02699_si_001.pdf]

Supporting Information

Oxidative Scission of Bicyclo[2.2.2]octenones : Untying the α-dimethoxycarbonyl

Ting-Zhi Yao, Yi-Cheng Tseng, Jia-Luo Li, Deng-Lian Hou, and Gary Jing Chuang*

Department of Chemistry, Chung Yuan Christian University, Chung-Li, Taiwan 320314

gjchuang@cycu.edu.tw

Table of Contents

Materials and Methods S10

Synthesis of compound 2 .... S10

General procedure for the synthesis of ketoxime 3 using 3a as an example. S10

4-bromo-7a-methoxy-2,3,3a,7a-tetrahydro-3,6-methanobenzofuran-7(*6H*)-one oxime, 3a S11

7a-methoxy-2,3,3a,7a-tetrahydro-3,6-methanobenzofuran-7(*6H*)-one oxime, 3b S11

5-bromo-7a-methoxy-2,3,3a,7a-tetrahydro-3,6-methanobenzofuran-7(*6H*)-one oxime, 3c S12

7a-methoxy-4-methyl-2,3,3a,7a-tetrahydro-3,6-methanobenzofuran-7(*6H*)-one oxime, 3d S12

7a-methoxy-4-(trimethylsilyl)-2,3,3a,7a-tetrahydro-3,6-methanobenzofuran-7(*6H*)-one oxime, 3e S13

4-allyl-7a-methoxy-2,3,3a,7a-tetrahydro-3,6-methanobenzofuran-7(*6H*)-one oxime, 3f ... S14

7a-methoxy-3a-methyl-2,3,3a,7a-tetrahydro-3,6-methanobenzofuran-7(*6H*)-one oxime, 3g S14

3a,7a-dimethoxy-2,3,3a,7a-tetrahydro-3,6-methanobenzofuran-7(6H)-one oxime, 3h S15

4-bromo-7a-methoxy-8-methyl-2,3,3a,7a-tetrahydro-3,6-methanobenzofuran-7(*6H*)-one oxime, 3i S16

4-bromo-7a-methoxy-8-phenyl-2,3,3a,7a-tetrahydro-3,6-methanobenzofuran-7(*6H*)-one oxime, 3j S16

5-bromo-8a-methoxy-3,4,4a,8a-tetrahydro-2H-4,7-methanochromen-8(*7H*)-one oxime, 3k S17

General procedure for the synthesis of lactone 4 using 4a as an example. S18

(3a*R**,5*S**,7a*S**)-7-bromo-1-oxo-1,3,3a,4,5,7a-hexahydroisobenzofuran-5-carbonitrile, 4a S18

(3a*R**,5*S**,7a*S**)-1-oxo-1,3,3a,4,5,7a-hexahydroisobenzofuran-5-carbonitrile, 4b S18

(3a*R**,5*R**,7a*R**)-6-bromo-1-oxo-1,3,3a,4,5,7a-hexahydroisobenzofuran-5-carbonitrile, 4c S19

(3a*R**,5*S**,7a*R**)-7-methyl-1-oxo-1,3,3a,4,5,7a-hexahydroisobenzofuran-5-carbonitrile, 4d S20

(3a*R**,5*S**,7a*S**)-1-oxo-7-(trimethylsilyl)-1,3,3a,4,5,7a-hexahydroisobenzofuran-5-carbonitrile, 4e S20

(3a*R**,5*S**,7a*R**)-7-Allyl-1-oxo-1,3,3a,4,5,7a-hexahydroisobenzofuran-5-carbonitrile, 4f S21

(3a*R**,5*S**,7a*S**)-7a-methyl-1-oxo-1,3,3a,4,5,7a-hexahydroisobenzofuran-5-carbonitrile, S4g S22

(3a*S**,5*S**,7a*R**)-7a-methoxy-1-oxo-1,3,3a,4,5,7a-hexahydroisobenzofuran-5-carbonitrile, 4h S22

(3a*R**,4*S**,5*S**,7a*S**)-7-bromo-4-methyl-1-oxo-1,3,3a,4,5,7a-hexahydroisobenzofuran-5-carbonitrile, 4i S23

(3a*R**,4*S**,5*S**,7a*S**)-7-bromo-1-oxo-4-phenyl-1,3,3a,4,5,7a-hexahydroisobenzofuran-5-carbonitrile, 4j S23

(4a*S**,6*S**,8a*S**)-8-bromo-1-oxo-3,4,4a,5,6,8a-hexahydro-1*H*-isochromene-6-carbonitrile, 4k S24

General procedure for the hydrolysis of 6 using 7a as an example. S25

(3a*S**,4*S**,7*S**,7a*S**,*E*)-6-bromo-9-(hydroxyimino)-2-phenyl-3a,4,7,7a-tetrahydro-1*H*-4,7-ethanoisoindole-1,3,8(2*H*)-trione, 7a S25

(3a*S**,4*R**,7*R**,7a*R**,*E*)-9-(hydroxyimino)-6-methyl-2-phenyl-3a,4,7,7a-tetrahydro-1*H*-4,7-ethanoisoindole-1,3,8(2*H*)-trione, 7c S26

(3a*S**,4*R**,7*R**,7a*R**,*E*)-6-(5,5-Dimethyl-1,3-dioxan-2-yl)-9-(hydroxyimino)-2-phenyl-3a,4,7,7a-tetrahydro-1*H*-4,7-ethanoisoindole- 1,3,8(2*H*)-trione, 7d S26

(3a*S**,4*R**,7*R**,7a*R**,*E*)-6-allyl-9-(hydroxyimino)-2-phenyl-3a,4,7,7a-tetrahydro-1*H*-4,7-ethanoisoindole-1,3,8(2*H*)-trione, 7e S27

(3a*S**,4*S**,7*S**,7a*S**,*E**)-9-(hydroxyimino)-2-phenyl-6-(trimethylsilyl)-3a,4,7,7a-tetrahydro-1*H*-4,7-ethanoisoindole-1,3,8(2*H*)-trione, 7f S28

(3a*S**,4*S**,7*S**,7a*R**,*E*)-9-(hydroxyimino)-5-methyl-2-phenyl-3a,4,7,7a-tetrahydro-1*H*-4,7-ethanoisoindole-1,3,8(2*H*)-trione, 7g S28

(3a*R**,4*R**,7*R**,7a*R**,*E*)-5-bromo-9-(hydroxyimino)-2-phenyl-3a,4,7,7a-tetrahydro-1*H*-4,7-ethanoisoindole-1,3,8(2*H*)-trione, 7h S29

(3a*S**,4*S**,7*S**,7a*R**,*E*)-5-(5,5-dimethyl-1,3-dioxan-2-yl)-9-(hydroxyimino)-2-phenyl-3a,4,7,7a-tetrahydro-1*H*-4,7-ethanoisoindole-1,3,8(2*H*)-trione, 7i S30

Methyl (3a*S**,4*S**,7*S**,7a*R**,*E*)-9-(hydroxyimino)-1,3,8-trioxo-2-phenyl-2,3,3a,4,7,7a-hexahydro-1*H*-4,7-ethanoisoindole-5-carboxylate, 7j S30

(3a*S**,4*R**,7*S**,7a*S**,*E*)-9-(hydroxyimino)-7-methyl-2-phenyl-3a,4,7,7a-tetrahydro-1*H*-4,7-ethanoisoindole-1,3,8(2*H*)-trione, 7k S31

(3a*S**,4*R**,7*R**,7a*S**,*E*)-9-(hydroxyimino)-7-methoxy-2-phenyl-3a,4,7,7a-tetrahydro-1*H*-4,7-ethanoisoindole-1,3,8(2*H*)-trione, 7l S32

(3a*R**,4*R**,7*S**,7a*S**,*E*)-9-(hydroxyimino)-3a,4,7,7a-tetrahydro-1*H*-4,7-ethanoinden-8-one, 7m S32

(3a*R**,4*S**,7*R**,7a*S**,*E*)-9-(hydroxyimino)-5-methyl-3a,4,7,7a-tetrahydro-1H-4,7-ethanoinden-8-one, 7o S33

(1*R**,4*R**,8*R**,*E*)-3-(hydroxyimino)-8-phenylbicyclo[2.2.2]oct-5-en-2-one, 7p S34

(1*R**,4*R**,8*R**,*E*)-3-(hydroxyimino)-6-methyl-8-phenylbicyclo[2.2.2]oct-5-en-2-one, 7q S34

(1R*,4S*,8R*,E)-3-(hydroxyimino)-5-methyl-8-phenylbicyclo[2.2.2]oct-5-en-2-one, 7r. S35

General procedure for the oxidative cleavage of 7 using 8a as an example. S36

dimethyl (3a*S**,4*S**,7*S**,7a*R**)-5-bromo-1,3-dioxo-2-phenyl-2,3,3a,4,7,7a-hexahydro-1*H*-isoindole-4,7-dicarboxylate, 8a(8h) S36

dimethyl (3a*R**,4*R**,7*S**,7a*S**)-1,3-dioxo-2-phenyl-2,3,3a,4,7,7a-hexahydro-1*H*-isoindole-4,7-dicarboxylate, 8c(8g) S37

dimethyl (3a*S**,4*R**,7*R**,7a*R**)-5-(5,5-dimethyl-1,3-dioxan-2-yl)-1,3-dioxo-2-phenyl-2,3,3a,4,7,7a-hexahydro-1*H*-isoindole-4,7-dicarboxylate, 8d(8i) S37

dimethyl (3a*S**,4*R**,7*R**,7a*R**)-5-allyl-1,3-dioxo-2-phenyl-2,3,3a,4,7,7a-hexahydro-1*H*-isoindole-4,7-dicarboxylate, 8e S38

dimethyl (3a*S**,4*S**,7*S**,7a*R**)-1,3-dioxo-2-phenyl-5-(trimethylsilyl)-2,3,3a,4,7,7a-hexahydro-1*H*-isoindole-4,7-dicarboxylate, 8f S39

dimethyl (3a*S**,4*R**,7*R**,7a*R**)-4-methoxy-1,3-dioxo-2-phenyl-2,3,3a,4,7,7a-hexahydro-1*H*-isoindole-4,7-dicarboxylate, 8l S39

dimethyl (3a*R**,4*R**,7*S**,7a*S**)-3a,4,7,7a-tetrahydro-1*H*-indene-4,7-dicarboxylate, 8m.... S40

dimethyl (3a*R**,4*S**,7*R**,7a*S**)-5-methyl-3a,4,7,7a-tetrahydro-1*H*-indene-4,7-dicarboxylate, 8o S41

dimethyl (1*R**,2*R**,5*R**)-1,2,5,6-tetrahydro-[1,1'-biphenyl]-2,5-dicarboxylate, 8p............ S41

dimethyl (1*R**,2*R**,5*R**)-4-methyl-1,2,5,6-tetrahydro-[1,1'-biphenyl]-2,5-dicarboxylate, 8q S42

dimethyl (1*R**,2*S**,5*R**)-3-methyl-1,2,5,6-tetrahydro-[1,1'-biphenyl]-2,5-dicarboxylate, 8r S42

General procedure for the Schmidt reaction for the synthesis of 9, using 9a as an example. S43

9a/9a' S43

(3a*R**,4*R**,12*S**,12a*R**)-2-Phenyl-3a,4,7,8,9,10,12,12a-octahydro-1H-4,12-ethenopyrrolo[3,4-h][1]oxa[5]azacycloundecine-1,3,5,11(2*H*)-tetraone, 9b S44

(3a*R**,4*R**,12*R**,12a*S**)-13-methyl-2-phenyl-3a,4,7,8,9,10,12,12a-octahydro-1H-4,12-ethenopyrrolo[3,4-h][1]oxa[5]azacycloundecine-1,3,5,11(2*H*)-tetraone, 9c(9g'); (3a*S**,4*R**,12*R**,12a*S**)-14-methyl-2-phenyl-3a,4,7,8,9,10,12,12a-octahydro-1H-4,12-ethenopyrrolo[3,4-h][1]oxa[5]azacycloundecine-1,3,5,11(2*H*)-tetraone, 9c'(9g)............... S45

9e/9e' S46

9s/9s' S47

(3a*R**,4*S**,12*S**,12a*R**)-14-methyl-2-phenyl-3a,4,7,8,9,10,12,12a-octahydro-1H-4,12-ethenopyrrolo[3,4-h][1]oxa[5]azacycloundecine-1,3,5,11(2*H*)-tetraone, 9g(9c'),

(3a*S**,4*S**,12*S**,12a*R**)-13-methyl-2-phenyl-3a,4,7,8,9,10,12,12a-octahydro-1H-4,12-ethenopyrrolo[3,4-h][1]oxa[5]azacycloundecine-1,3,5,11(2*H*)-tetraone, 9g'(9c).............. S48

methyl-(3a*R**,4*S**,12*S**,12a*R**)-1,3,5,11-tetraoxo-2-phenyl-2,3,3a,4,5,7,8,9,10,11,12,12a-dodecahydro-1*H*-4,12-ethenopyrrolo[3,4-h][1]oxa[5]azacycloundecine-14-carboxylate, 9j, methyl-(3a*S**,4*S**,12*S**,12a*R**)-1,3,5,11-tetraoxo-2-phenyl-2,3,3a,4,5,7,8,9,10,11,12,12a-dodecahydro-1*H*-4,12-ethenopyrrolo[3,4-h][1]oxa[5]azacycloundecine-13-carboxylate, 9j' S48

(3a*R**,4*R**,12*S**,12a*S**)-12-methyl-2-phenyl-3a,4,7,8,9,10,12,12a-octahydro-1H-4,12-ethenopyrrolo[3,4-h][1]oxa[5]azacycloundecine-1,3,5,11(2*H*)-tetraone, 9k S49

(3a*R**,4*R**,12R*,12a*S**)-12-methoxy-2-phenyl-3a,4,7,8,9,10,12,12a-octahydro-1*H*-4,12-ethenopyrrolo[3,4-h][1]oxa[5]azacycloundecine-1,3,5,11 (2*H*) -tetraone 9l S50

(1*S**,9*R**,9a*S**,12a*R**)-13-methyl-4,5,6,7,9,9a,10,12a-octahydro-2H-1,9-ethenocyclopenta[h][1]oxa[5]azacycloundecine-2,8(1*H*)-dione, 9n S51

(1*R**,9*S**,9a*S**,12a*R**)-1-methyl-4,5,6,7,9,9a,10,12a-octahydro-2H-1,9-ethenocyclopenta[h][1]oxa[5]azacycloundecine-2,8(1*H*)-dione, 9v S51

(1*R**,9*R**,12*R**)-10-methyl-12-phenyl-3-oxa-7-azabicyclo[7.2.2]tridec-10-ene-2,8-dione, 9q S52

methyl (1*R**,9*R**,12*R**)-2,8-dioxo-12-phenyl-3-oxa-7-azabicyclo[7.2.2]tridec-10-ene-10-carboxylate, 9w.. S53

(1*R**,9*R**,12*S**)-1-methyl-12-phenyl-3-oxa-7-azabicyclo[7.2.2]tridec-10-ene-2,8-dione 1-methyl-12-phenyl-3-oxa-7-azabicyclo[7.2.2]tridec-10-ene-2,8-dione, 9x S54

(3aR*,4R*,11R*,11aS*)-12-methyl-2-phenyl-3a,4,8,9,11,11a-hexahydro 4,11-ethenopyrrolo[3,4-g][1,4]oxazecine-1,3,5,10 (2H,7H) -tetraone, 9y S54

^{1}H NMR of 2a.. S56

^{1}H NMR of 2b S57

^{1}H NMR of 2c.. S58

^{1}H NMR of 2d S59

^{1}H NMR of 2e.. S60

^{1}H NMR of 2f S61

^{1}H NMR of 2g S62

^{1}H NMR of 2h S63

^{1}H NMR of 2i S64

^{1}H NMR of 2j S65

^{1}H NMR of 2k S66

^{1}H NMR of 3a.. S67

^{13}C{1H} and DEPT 90, 135 NMR of 3a S68

^{1}H NMR of 3b S69
$^{13}C\{1H\}$ and DEPT 90, 135 NMR of 3b S70
^{1}H NMR of 3c S71
$^{13}C\{1H\}$ and DEPT 90, 135 NMR of 3c S72
^{1}H NMR of 3d S73
$^{13}C\{1H\}$ and DEPT 90, 135 NMR of 3d S74
^{1}H NMR of 3e S75
$^{13}C\{1H\}$ and DEPT 90, 135 NMR of 3e S76
^{1}H NMR of 3f S77
$^{13}C\{1H\}$ and DEPT 90, 135 NMR of 3f S78
^{1}H NMR of 3g S79
$^{13}C\{1H\}$ and DEPT 90, 135 NMR of 3g S80
^{1}H NMR of 3h S81
$^{13}C\{1H\}$ and DEPT 90, 135 NMR of 3h S82
^{1}H NMR of 3i S83
$^{13}C\{1H\}$ and DEPT 90, 135 NMR of 3i S84
^{1}H NMR of 3j S85
$^{13}C\{1H\}$ and DEPT 90, 135 NMR of 3j S86
^{1}H NMR of 3k S87
$^{13}C\{1H\}$ and DEPT 90, 135 NMR of 3k S88
^{1}H NMR of 4a S89
$^{13}C\{1H\}$ and DEPT 90, 135 NMR of 4a S90
^{1}H NMR of 4b S91
$^{13}C\{1H\}$ and DEPT 90, 135 NMR of 4b S92
^{1}H NMR of 4c S93
$^{13}C\{1H\}$ and DEPT 90, 135 NMR of 4c S94
^{1}H NMR of 4d S95
$^{13}C\{1H\}$ and DEPT 90, 135 NMR of 4d S96
^{1}H NMR of 4e S97
$^{13}C\{1H\}$ and DEPT 90, 135 NMR of 4e S98
^{1}H NMR of 4f S99
$^{13}C\{1H\}$ and DEPT 90, 135 NMR of 4f S100
^{1}H NMR of 4g S101
$^{13}C\{1H\}$ and DEPT 90, 135 NMR of 4g S102

^{1}H NMR of 4h S103
^{13}C{1H} and DEPT 90, 135 NMR of 4h S104
^{1}H NMR of 4i S105
^{13}C{1H} and DEPT 90, 135 NMR of 4i S106
^{1}H NMR of 4j S107
^{13}C{1H} and DEPT 90, 135 NMR of 4j S108
^{1}H NMR of 4k S109
^{13}C{1H} and DEPT 90, 135 NMR of 4k S110
^{1}H NMR of 5a S111
^{1}H NMR of 5b S112
^{1}H NMR of 5c S113
^{1}H NMR of 5d S114
^{1}H NMR of 5e S115
^{1}H NMR of 5f S116
^{1}H NMR of 5g S117
^{1}H NMR of 5h S118
^{1}H NMR of 5i S119
^{1}H NMR of 5j S120
^{1}H NMR of 5k S121
^{1}H NMR of 5l S122
^{1}H NMR of 5m S123
^{1}H NMR of 5n S124
^{1}H NMR of 5o S125
^{1}H NMR of 5p S126
^{1}H NMR of 5q S127
^{1}H NMR of 5r S128
^{1}H NMR of 5s S129
^{1}H NMR of 5t S130
^{1}H NMR of 5u S131
^{1}H NMR of 5v S132
^{1}H NMR of 5w S133
^{1}H NMR of 5x S134
^{1}H NMR of 6a S135
^{1}H NMR of 6c S136

^{1}H NMR of 6d S137

^{1}H NMR of 6e S138

^{1}H NMR of 6f S139

^{1}H NMR of 6g S140

^{1}H NMR of 6h S141

^{1}H NMR of 6i S142

^{1}H NMR of 6j S143

^{1}H NMR of 6k S144

^{1}H NMR of 6l S145

^{1}H NMR of 6m S146

^{1}H NMR of 6o S147

^{1}H NMR of 6p S148

^{1}H NMR of 6q S149

^{1}H NMR of 6r S150

^{1}H NMR of 7a S151

^{13}C{1H} and DEPT 90, 135 NMR of 7a S152

^{1}H NMR of 7c S153

^{13}C{1H} and DEPT 90, 135 NMR of 7c S154

^{1}H NMR of 7d S155

^{13}C{1H} and DEPT 90, 135 NMR of 7d S156

^{1}H NMR of 7e S157

^{13}C{1H} and DEPT 90, 135 NMR of 7e S158

^{1}H NMR of 7f S159

^{13}C{1H} and DEPT 90, 135 NMR of 7f S160

^{1}H NMR of 7g S161

^{13}C{1H} and DEPT 90, 135 NMR of 7g S162

^{1}H NMR of 7h S163

^{13}C{1H} and DEPT 90, 135 NMR of 7h S164

^{1}H NMR of 7i S165

^{13}C{1H} and DEPT 90, 135 NMR of 7i S166

^{1}H NMR of 7j S167

^{1}H NMR of 7k S168

^{13}C{1H} NMR of 7k S169

^{1}H NMR of 7l S170

$^{13}C\{1H\}$ and DEPT 90, 135 NMR of 7l S171
^{1}H NMR of 7m S172
$^{13}C\{1H\}$ and DEPT 90, 135 NMR of 7m S173
^{1}H NMR of 7o S174
$^{13}C\{1H\}$ and DEPT 90, 135 NMR of 7o S175
^{1}H NMR of 7p S176
$^{13}C\{1H\}$ and DEPT 90, 135 NMR of 7p S177
^{1}H NMR of 7q S178
$^{13}C\{1H\}$ and DEPT 90, 135 NMR of 7q S179
^{1}H NMR of 7r S180
$^{13}C\{1H\}$ and DEPT 90, 135 NMR of 7r S181
^{1}H NMR of 8a(8h) S182
$^{13}C\{1H\}$ and DEPT 90, 135 NMR of 8a(8h) S183
^{1}H NMR of 8b S184
$^{13}C\{1H\}$ and DEPT 90, 135 NMR of 8b S185
^{1}H NMR of 8c(8g) S186
$^{13}C\{1H\}$ and DEPT 90, 135 NMR of 8c(8g) S187
^{1}H NMR of 8d(8i) S188
$^{13}C\{1H\}$ and DEPT 90, 135 NMR of 8d(8i) S189
^{1}H NMR of 8e S190
$^{13}C\{1H\}$ and DEPT 90, 135 NMR of 8e S191
^{1}H NMR of 8f S192
$^{13}C\{1H\}$ and DEPT 90, 135 NMR of 8f S193
^{1}H NMR of 8l S194
$^{13}C\{1H\}$ and DEPT 90, 135 NMR of 8l S195
^{1}H NMR of 8m S196
$^{13}C\{1H\}$ and DEPT 90, 135 NMR of 8m S197
^{1}H NMR of 8n S198
$^{13}C\{1H\}$ and DEPT 90, 135 NMR of 8n S199
^{1}H NMR of 8o S200
$^{13}C\{1H\}$ and DEPT 90, 135 NMR of 8o S201
^{1}H NMR of 8p S202
$^{13}C\{1H\}$ and DEPT 90, 135 NMR of 8p S203
^{1}H NMR of 8q S204

$^{13}C\{1H\}$ and DEPT 90, 135 NMR of 8q S205
^{1}H NMR of 8r S206
$^{13}C\{1H\}$ and DEPT 90, 135 NMR of 8r S207
^{1}H NMR of 9a S208
$^{13}C\{1H\}$ and DEPT 90, 135 NMR of 9a S209
^{1}H NMR of 9b + 9b’ S210
$^{13}C\{1H\}$ and DEPT 90, 135 NMR of 9b + 9b’ S211
^{1}H NMR of 9c+9c’ S212
$^{13}C\{1H\}$ and DEPT 90, 135 NMR of 9c+9c’ S213
^{1}H NMR of 9e + 9e’ S214
$^{13}C\{1H\}$ and DEPT 90, 135 NMR of 9e + 9e’ S215
^{1}H NMR of 9s + 9s’ S216
$^{13}C\{1H\}$ and DEPT 90, 135 NMR of 9s + 9s’ S217
^{1}H NMR of 9g+9g’ S218
$^{13}C\{1H\}$ and DEPT 90, 135 NMR of 9g+9g’ S219
^{1}H NMR of 9j+9j’ S220
$^{13}C\{1H\}$ and DEPT 90, 135 NMR of 9j+9j’ S221
^{1}H NMR of 9k S222
$^{13}C\{1H\}$ and DEPT 90, 135 NMR of 9k S223
^{1}H NMR of 9l S224
$^{13}C\{1H\}$ and DEPT 90, 135 NMR of 9l S225
^{1}H NMR of 9n S226
$^{13}C\{1H\}$ and DEPT 90, 135 NMR of 9n S227
^{1}H NMR of 9v S228
$^{13}C\{1H\}$ and DEPT 90, 135 NMR of 9v S229
^{1}H NMR of 9q S230
$^{13}C\{1H\}$ and DEPT 90, 135 NMR of 9q S231
^{1}H NMR of 9w S232
$^{13}C\{1H\}$ and DEPT 90, 135 NMR of 9w S233
^{1}H NMR of 9x S234
$^{13}C\{1H\}$ and DEPT 90, 135 NMR of 9x S235
^{1}H NMR of 9y S236
$^{13}C\{1H\}$ and DEPT 90, 135 NMR of 9y S237
ORTEP and x-ray data of 4a S238

ORTEP and x-ray data of 4k (CCDC 2386154) S246
ORTEP and x-ray data of 7a (CCDC 2386155) S254
ORTEP and x-ray data of 9c (CCDC 2393498) S263

Materials and Methods

Reactions were carried out under ambient atmosphere unless otherwise specified. Anhydrous methanol and dichloromethane were dried by distillation from CaH_2. Anhydrous THF, benzene and toluene were dried by distillation from Na/benzophenone. Commercially obtained reagents were used as received unless otherwise specified. Thin layer chromatography (TLC) was performed using Merck TLC Aluminum sheets silica gel 60 F_{254} plates and visualized by fluorescence quenching under UV light and $KMnO_4$ stain. Flash chromatography was performed using silica gel (Chromatorex, MB 70-40/75, 40-75 μm) purchased by Fuji Silysia Chemicals. NMR spectra were recorded on a Bruker AVANCE II operating at 400 MHz for ^1H and 100 MHz for ^{13}C acquisitions, respectively. Chemical shifts are reported in ppm with the solvent resonance as the internal standard. The following solvent chemical shifts were used as reference values (ppm): $CDCl_3$ = 7.26 (^1H), 77.0 (^{13}C). Data is reported as follows: s = singlet, br = broad, d = doublet, t = triplet, q = quartet, m = multiplet; coupling constants in Hz; integration. High-resolution mass spectra were obtained on JMS-700 with time of flight (TOF) analyzer at the Academia Sinica (Taiwan). X-ray crystals data were obtained on Bruker AXS P4 and Bruker D8 Venture. Melting points were determined by using a Büchi melting point B-540.

Synthesis of compound 2

Synthesis of **2** (known compounds) were carried out by using procedure reported in *J. Org. Chem.* 1999, 64, 11, 4111–4118. ^1H NMRs of compound **2** were used for the identification and were found matching with reported data.

General procedure for the synthesis of ketoxime 3 using 3a as an example.

To a solution of **2a** (516 mg, 2.00 mmol, 1 eq) in 17 mL of EtOH/H_2O (1:1) was added hydroxyamine hydrochloride (1.39g, 20 mmol, 10 eq) and sodium acetate (1.64g, 20 mmol, 10 eq). The reaction was then stirred at rt for 4h. 30 mL of saturated $NaHCO_3$ (aq) was added to the reaction mixture and then

extracted with EtOAc. The organic phase was collected and dried with anhydrous $MgSO_4$ and solvent was removed using rotavap. **3a** (270 mg, 49%) was isolated by column chromatography (EA/Hexs = 1:3) as a white solid.

4-bromo-7a-methoxy-2,3,3a,7a-tetrahydro-3,6-methanobenzofuran-7(*6H*)-one oxime, 3a

Spectral data of **3a**: ^{1}H NMR (400 MHz, $CDCl_3$, 24 °C) δ 6.50 (dd, *J* = 3.2 Hz, 1H), 4.26 – 4.23 (m, 1H), 4.11 (q, *J* = 4.0 Hz, 1H), 3.74 (d, *J* = 8.2 Hz, 1H), 3.50 (s, 1H), 3.48 (q, *J* = 2.3 Hz, 1H), 2.64 – 2.60 (m, 1H), 1.86 – 1.79 (m, 1H), 1.72-1.71 (m, 1H). $^{13}C\{^{1}H\}$ NMR (100 MHz, $CDCl_3$) δ 158.1 (C), 133.3 (C), 131.4 (CH), 119.1 (C), 106.0 (C), 72.3(CH_2), 51.5 (CH), 51.4 (CH_3), 35.4 (CH), 32.3 (CH_2), 31.7 (CH). HRMS (ESI-TOF) (m/z): $[M+Na]^+$ calcd for $C_{10}H_{12}BrNO_3Na$ 295.9893, 297.9872; Found, 295.9894, 297.9874. IR (neat, cm^{-1}): 3281, 2935, 1698, 1542, 1369, 1221, 1142, 1055, 1000, 955, 922. mp 165.4-169.4 °C

7a-methoxy-2,3,3a,7a-tetrahydro-3,6-methanobenzofuran-7(*6H*)-one oxime, 3b

2b (199 mg, 1.10 mmol) was reacted according to the general procedure for 4 h. **3b** (190 mg, 88%) was isolated by washing the crude mixture with 50 mL of ether as a white solid. Spectral data of **3b**: ^{1}H NMR (400 MHz, $CDCl_3$, 24 °C) δ 6.38 (t, *J* = 4.0 Hz, 1H), 6.15 (t, *J* = 3.2 Hz, 1H), 4.20 (q, *J* = 2.2 Hz, 1H), 4.10 (dd, *J* = 7.9, 3.9 Hz, 1H), 3.74 (d, *J* = 7.9 Hz, 1H), 3.46 (s, 3H), 3.28 – 3.25 (m, 1H), 2.37 (sext, *J* = 4.0 Hz, 1H), 1.72-1.64 (m, 2H). $^{13}C\{^{1}H\}$ NMR

(100 MHz, CD_3OD) δ 159.1 (C), 132.6 (CH), 128.4 (CH), 72.4 (C), 49.4 (CH_3), 41.4 (CH), 35.2 (CH), 32.0 (CH_2), 29.4 (CH). HRMS (ESI-TOF) (m/z): $[M+Na]^+$ calcd for $C_{10}H_{13}NO_3Na$, 218.0788; Found, 218.0793. IR (neat, cm^{-1}) 2956, 1141, 1053, 959, 947. mp 193.9-196.4 °C

5-bromo-7a-methoxy-2,3,3a,7a-tetrahydro-3,6-methanobenzofuran-7(*6H*)-one oxime, 3c

2c (200 mg, 0.80 mmol) was reacted according to the general procedure for 4 h. **3c** (68 mg, 32%) was isolated by washing the crude mixture with 50 mL of ether as a white solid. Spectral data of **3c**: ^1H NMR (400 MHz, $CDCl_3$, 24 ºC) δ 6.28 (dd, J = 7.2, 2.2 Hz, 1H), 4.37 (td, J = 3.9, 1.9 Hz, 1H), 4.09 (dd, J = 8.1, 4.0 Hz, 1H), 3.73 (d, J = 8.2 Hz,1H), 3.47 (s, 3H), 3.30 (dd, J = 7.1, 4.5 Hz, 1H), 2.47 (td, J = 8.6, 4.3 Hz, 1H), 2.00 – 1.95 (m, 1H), 1.73 (dd, J = 13.7, 3.3 Hz, 1H). $^{13}C\{^1H\}$ NMR (100 MHz, CD_3OD) δ 156.8 (C), 128.0 (CH), 121.6 (C), 105.6 (C), 72.1 (CH_2), 49.6 (CH_3), 44.0 (CH), 39.6 (CH), 35.5 (CH), 32.7 (CH_2). HRMS (ESI-TOF) (m/z): $[M+Na]^+$ calcd for $C_{10}H_{12}BrNO_3Na$, 295.9893, 297.9872; Found, 295.9892, 297.9872. IR (neat, cm^{-1}) 3278, 2936, 1694, 1445, 1223, 1206, 1174, 1012, 986, 895, 735. mp 143.8-145.5 °C

7a-methoxy-4-methyl-2,3,3a,7a-tetrahydro-3,6-methanobenzofuran-7(*6H*)-one oxime, 3d

2d (150 mg, 0.78 mmol) was reacted according to the general procedure for 4

h. **3d** (64 mg, 56%) was isolated by column chromatography (EA/Hexs = 1:1) as a white solid. Spectral data of **3d**: ^{1}H NMR (400 MHz, $CDCl_3$, 24 ºC) δ 5.97 (td, *J* = 6.8, 1.7 Hz, 1H), 4.08 (dd, *J* = 7.7, 3.8 Hz, 2H), 3.74 (d, *J* = 8.0 Hz, 1H), 3.46 (s, 3H), 3.09 (dd, *J* = 4.4, 1.7 Hz, 1H), 2.44-2.36 (m, 1H), 1.87 (d, *J* = 1.6 Hz, 3H), 1.77-1.63 (m, 2H). $^{13}C\{^{1}H\}$ NMR (100 MHz, CD_3OD) δ 157.3 (C), 129.1 (CH), 118.3 (C), 105.4 (C), 71.9 (CH_2), 51.4 (CH), 50.0 (CH), 44.3 (CH), 38.7 (CH), 38.3 (CH), 18.4 (CH_3). HRMS (ESI-TOF) (m/z): $[M+Na]^+$ calcd for $C_{11}H_{15}NO_3Na$, 232.0944; Found, 232.0950. IR (neat, cm^{-1}) 3233, 2955, 1437, 1133, 1093, 1028, 952, 887, 745. mp 201.6-203.8 °C

7a-methoxy-4-(trimethylsilyl)-2,3,3a,7a-tetrahydro-3,6-methanobenzofuran-7(*6H*)-one oxime, 3e

2e (51 mg, 0.20 mmol) was reacted according to the general procedure for 4 h. **3e** (26 mg, 48%) was isolated by column chromatography (EA/Hexs = 1:3) as a white solid. Spectral data of **3e**: ^{1}H NMR (400 MHz, $CDCl_3$, 24 ºC) δ 6.63 (d, *J* = 5.8 Hz, 1H), 4.20 (s, 1H), 4.09 (d, *J* = 5.0 Hz, 1H), 3.74 (d, *J* = 7.2 Hz, 1H), 3.46 (s, 3H), 3.35 (d, *J* = 3.4 Hz, 1H), 2.24 (d, *J* = 2.7 Hz, 1H), 1.7-1.64 (m, 2H), 0.11 (s, 9H).$^{13}C\{^{1}H\}$ NMR (100 MHz, $CDCl_3$) δ 142.8 (C), 140.9 (CH), 72.9 (CH_2), 50.2 (CH_3), 43.3 (CH), 35.3 (CH), 32.1 (CH_2), 30.5 (CH), -2.2 (3 x CH_3). HRMS (ESI-TOF) (m/z): $[M+Na]^+$ calcd for $C_{13}H_{21}NO_3SiNa$, 290.1183; Found, 290.1181. IR (neat, cm^{-1}) 3277, 2953,

1656, 1459, 1246, 1169, 1001, 958, 836. mp 130.2-133.2 °C

4-allyl-7a-methoxy-2,3,3a,7a-tetrahydro-3,6-methanobenzofuran-7(*6H*)-one oxime, 3f

2f (239 mg, 1.10 mmol) was reacted according to the general procedure for 4 h. **3f** (199 mg, 78%) was isolated by column chromatography (EA/Hexs = 1:3) as a white solid. Spectral data of **3f**: ^{1}H NMR (400 MHz, $CDCl_3$, 24 °C) δ 6.02 (dd, *J* = 6.8, 1.3 Hz, 1H), 5.83 – 5.72 (m, 1H), 5.14 – 5.08 (m, 1H), 4.14 – 4.11 (m, 1H), 4.07 (dd, *J* = 7.9, 3.8 Hz, 1H), 3.72 (d, *J* = 7.9 Hz, 1H), 3.44 (s, 3H), 3.12 (dd, *J* = 4.4, 1.6 Hz, 1H), 2.90 (d, *J* = 6.7 Hz, 1H), 2.37 – 2.32 (m, 1H), 1.74 – 1.68 (m, 1H). ^{13}C{^{1}H} NMR (100 MHz, $CDCl_3$) δ 160.5 (C), 139.8 (C), 135.1 (CH), 125.5 (CH), 117.1 (CH_2), 106.1 (C), 72.9 (CH_2), 50.5 (CH_3), 44.8 (CH), 39.6 (CH_2), 35.3 (CH), 32.9 (CH_2), 29.5 (CH). HRMS (ESI-TOF) (m/z): $[M+Na]^+$ calcd for $C_{13}H_{17}NO_3Na$, 258.1101; Found, 258.1101. IR (neat, cm^{-1}) 3261, 2942, 1633, 1165, 1132, 1000, 961, 925. mp 97.8-100.0 °C

7a-methoxy-3a-methyl-2,3,3a,7a-tetrahydro-3,6-methanobenzofuran-7(*6H*)-one oxime, 3g

2g (91 mg, 0.47 mmol) was reacted according to the general procedure for 4 h. **3g** (77 mg, 69%) was isolated by column chromatography (EA/Hexs = 1:5) as a white solid. Spectral data of **3g**: ^{1}H NMR (400 MHz, $CDCl_3$, 24 °C) δ

6.32 (t, J = 7.4 Hz, 1H), 5.91 (d, J = 8.2 Hz, 1H), 4.27 (dd, J = 8.1, 4.1 Hz, 1H), 4.21- 4.16 (m, 1H), 3.64 (d, J = 8.3 Hz, 1H), 3.45 (s, 3H), 2.05 (dd, J = 7.7, 2.2 Hz, 1H), 1.78 (t, J = 11.4 Hz, 1H), 1.67 (dd, J = 11.6, 1.6 Hz, 1H), 1.32 (s, 3H). $^{13}C\{^1H\}$ NMR (100 MHz, $CDCl_3$) δ 158.0 (C), 134.7 (CH), 131.4 (CH), 106.4 (C), 71.5 (CH_2), 52.7 (CH), 41.2 (CH), 34.9 (CH_2), 29.5 (CH), 15.4 (CH). HRMS (ESI-TOF) (m/z): $[M+Na]^+$ calcd for $C_{11}H_{15}NO_3Na$, 232.0944; Found, 232.0938. IR (neat, cm^{-1}) 3295, 2927, 1455, 1143, 1049, 932, 856. mp 167.1-169.1°C

3a,7a-dimethoxy-2,3,3a,7a-tetrahydro-3,6-methanobenzofuran-7(6H)-one oxime, 3h

2h (203 mg, 0.97 mmol) was reacted according to the general procedure for 4 h. **3h** (127 mg, 67%) was isolated by column chromatography (EA/Hexs = 1:5) as a white solid. Spectral data of **3h**: ^1H NMR (400 MHz, $CDCl_3$, 24 ºC) δ 6.36 – 6.27 (m, 1H), 4.31 (dd, J = 7.7, 4.0 Hz, 1H), 4.16 – 4.13 (m, 1H), 3.64 (d, J = 7.7 Hz, 1H), 3.53 (s, 3H), 3.48 (s, 3H), 2.55 (d, J = 1.4 Hz, 1H), 2.54 (d, J = 13.9 Hz, 1H), 2.53 (d, J = 1.2 Hz, 1H). $^{13}C\{^1H\}$ NMR (100 MHz, $CDCl_3$) δ 159.0 (C), 131.1 (CH), 129.3 (CH), 105.8 (C), 86.0 (C), 71.9 (CH_2), 53.9 (CH_3), 52.2 (CH_3), 36.2 (CH), 33.0 (CH_2), 28.7 (CH). HRMS (ESI-TOF) (m/z): $[M+Na]^+$ calcd for $C_{11}H_{15}NO_4Na$, 248.0893. Found, 248.0897. IR (neat, cm^{-1}) 3252, 2942, 1636, 1139, 1049, 948, 869. mp 175.9-179.2 °C

4-bromo-7a-methoxy-8-methyl-2,3,3a,7a-tetrahydro-3,6-methanobenzofuran-7(*6H*)-one oxime, 3i

2i (50 mg, 0.18 mmol) was reacted according to the general procedure for 4 h. **3i** (36 mg, 69%) was isolated by washing the crude mixture with 50 mL of ether as a white solid. Spectral data of **3i**: ^{1}H NMR (400 MHz, $CDCl_3$, 24 °C) δ 6.39 (dd, *J* = 7.2, 2.2 Hz, 1H), 4.11 – 4.06 (m, 1H), 3.75 (d, *J* = 8.1 Hz, 1H), 3.49 (s, 3H), 3.45 (q, *J* = 2.0 Hz, 1H), 2.09 (t, *J* = 3.5 Hz, 1H), 2.00 (d, *J* = 7.3 Hz, 1H), 0.99 (d, *J* = 7.1 Hz, 3H). ^{13}C{^{1}H} NMR (100 MHz, CD_3OD) δ 157.4 (C), 129.1 (CH), 118.3 (C), 105.4 (C), 71.9 (CH_2), 51.3 (CH), 50.0 (CH_3), 44.3 (CH), 38.7 (CH), 38.4 (CH), 18.4 (CH). HRMS (ESI-TOF) (m/z): $[M+Na]^+$ calcd for $C_{11}H_{14}BrNO_3Na$, 310.0049, 312.0029; Found, 310.0058, 312.0037. IR (neat, cm^{-1}) 2955, 2920, 2851, 1762. mp 203.7-206.0 °C

4-bromo-7a-methoxy-8-phenyl-2,3,3a,7a-tetrahydro-3,6-methanobenzofuran-7(*6H*)-one oxime, 3j

2j (27 mg, 0.079 mmol) was reacted according to the general procedure for 4 h. **3j** (26 mg, 93%) was isolated by washing the crude mixture with 50 mL of ether as a white solid. Spectral data of **3j**: ^{1}H NMR (400 MHz, $CDCl_3$, 24 °C) δ 7.34-7.21 (m, 2H), 7.11-7.03 (m, 3H), 6.21 (dd, *J* = 7.1, 2.1 Hz, 1H), 4.37 (dd, *J* = 7.1, 3.0 Hz, 1H), 4.19 (dd, *J* = 8.3, 3.9 Hz, 1H), 3.90 (d, *J* = 8.4 Hz, 1H), 3.65 (dd, *J* = 2.3, 4.4 Hz, 1H), 3.55 (s, 3H), 3.16 (s, 1H), 2.92 (t, *J* = 4.9

Hz, 1H), 1.26 (q, J = 4.8 Hz, 2H). $^{13}C\{^1H\}$ NMR (100 MHz, CD_3OD) δ 156.7 (C), 142.4 (C), 129.0 (CH), 128.0 (CH), 127.9 (CH), 126.4 (CH), 119.5 (C), 105.6 (C), 72.1 (CH_2), 51.8 (CH), 50.1 (CH_3), 48.9 (CH), 44.4 (CH), 39.6 (CH). HRMS (ESI-TOF) (m/z): $[M+Na]^+$ calcd for $C_{16}H_{16}BrNO_3Na$, 372.0206, 374.0185; Found, 372.0197, 374.0177. IR (neat, cm^{-1}) 3269, 2925, 1453, 1140, 1032, 962, 919, 748, 700. mp 215.6-220.3 °C

5-bromo-8a-methoxy-3,4,4a,8a-tetrahydro-2H-4,7-methanochromen-8(*7H*)-one oxime, 3k

2k (193 mg, 0.62 mmol) was reacted according to the general procedure for 4 h. **3k** (130 mg, 73%) was isolated by column chromatography (EA/Hexs = 1:2) as a white solid. Spectral data of **3k**: ^1H NMR (400 MHz, $CDCl_3$, 24 °C) 6.33 (dd, J = 2.1, 7.0 Hz, 1H), 4.30 (td, J = 3.1, 6.2 Hz, 1H), 3.90 (dd, J = 5.9, 12.3 Hz, 1H), 3.79 (ddd, J = 2.6, 12.8, 12.8 Hz, 1H), 3.49 (s, 3H), 2.99 (t, J = 2.8 Hz, 1H), 2.43 (td, J = 3.3, 11.0 Hz, 1H), 1.88 (m, 2H), 1.52 (m, 2H). $^{13}C\{^1H\}$ NMR (100 MHz, $CDCl_3$) δ 155.1 (C), 129.5 (CH), 122.7 (C), 60.2 (CH_2), 52.1 (CH), 49.8 (CH_3), 33.6 (CH), 29.5 (CH_2), 29.0 (CH), 28.7 (CH_2). HRMS (ESI-TOF) (m/z): $[M+Na]^+$ calcd for $C_{11}H_{14}BrNO_3$ 310.0049, 312.0029; Found, 310.0061, 321.0041. IR (neat, cm^{-1}) 3272, 2942, 2877, 1614, 1437, 1143, 960, 841, 750, 648. mp 163.8-165.5 °C.

General procedure for the synthesis of lactone 4 using 4a as an example. To a solution of **3a** (20 mg, 0.07 mmol, 1 eq) in 10 mL of toluene was added *p*-TSA(1mg, 0.0035 mmol, 0.05 eq). The reaction was then stirred at reflux temperature by using an oil bath for 12h. 30 mL of saturated $NaHCO_3$ (aq) was added to the reaction mixture and then extracted with CH_2Cl_2. The organic phase was collected and dried with anhydrous $MgSO_4$ and solvent was removed using rotavap. **4a** (16 mg, 96%) was isolated by column chromatography (EA/Hexs = 1:5) as a white solid.

(3a*R,5*S**,7a*S**)-7-bromo-1-oxo-1,3,3a,4,5,7a-hexahydroisobenzofuran-5-carbonitrile, 4a**

Spectral data of **4a**: ^{1}H NMR (400 MHz, $CDCl_3$, 24 °C) δ 6.30 (s,1H), 4.36 (dd, *J* = 9.6, 5.5 Hz, 1H), 4.14 (d, *J* = 9.6 Hz, 1H), 3.46-3.4 (m, 2H), 2.88-2.79 (m, 1H), 2.35-2.31 (m, 1H), 1.88 (dd, *J* = 13.1, 11.2 Hz, 1H). $^{13}C\{^1H\}$ NMR (100 MHz, $CDCl_3$) δ 171.4 (C), 125.3 (CH), 119.0 (C), 118.3 (C), 69.5 (CH), 46.2 (CH_2), 35.5 (CH), 28.3 (CH), 26.8 (CH_2). HRMS (ESI-TOF) (m/z): $[M+Na]^+$ calcd for $C_9H_8BrNO_2Na$, 263.9631, 265.9610; Found, 263.9632, 265.9611. **IR** (neat, cm^{-1}) 2923, 2853, 1773, 1455, 1375, 1164, 979. mp 142.8-145.6 °C. Crystal for X-ray crystallography was recrystallized from gas-diffusion in ether/ CH_2Cl_2. (CCDC 2386153)

(3a*R,5*S**,7a*S**)-1-oxo-1,3,3a,4,5,7a-hexahydroisobenzofuran-5-carbonitrile, 4b**

3b (54 mg, 0.27 mmol) was reacted according to the general procedure for 12

h. **4b** (33 mg, 73%) was isolated by column chromatography (EA/Hexs = 1:3) as a white solid. Spectral data of **4b**: ^{1}H NMR (400 MHz, $CDCl_3$, 24 ºC) δ δ 6.34 (qd, J = 6.9, 3.2 Hz, 1H), 6.21 (qd, J = 7.9, 2.6Hz, 1H), 4.13 (dd, J = 3.4, 8.0 Hz, 1H), 3.80 (d, J = 8.0 Hz, 1H), 3.51 (s, 3H), 3.33 (qd, J = 2.1, 6.3 Hz, 1H), 3.18 (t, J = 3.4 Hz, 1H), 2.50 (m, 1H), 1.87 (td, J = 2.5, 13.2 Hz, 1H), 1.80 (ddd, J = 2.5, 10.0, 12.9 Hz, 1H). ^{13}C{^{1}H} NMR (100 MHz, $CDCl_3$) δ 174.6 (C), 124.9 (CH), 124.4 (CH), 119.9 (C), 70.6 (CH_2), 39.2 (CH), 32.9 (CH), 28.1 (CH_2), 25.8 (CH). HRMS (ESI-TOF) (m/z): $[M+Na]^+$ calcd for $C_9H_9NO_2Na$, 186.0525; Found, 186.0534. **IR** (neat, cm^{-1}) 2920, 1764, 1375, 1209, 1124, 1051, 979, 731. mp 108.3-110.2 ℃

(3a*R,5*R**,7a*R**)-6-bromo-1-oxo-1,3,3a,4,5,7a-hexahydroisobenzofuran-5-carbonitrile, 4c**

3c (18 mg, 0.07 mmol) was reacted according to the general procedure for 12 h. **4c** (12 mg, 71%) was isolated by column chromatography (EA/Hexs = 1:3) as a white solid. Spectral data of **4c**: ^{1}H NMR (400 MHz, $CDCl_3$, 24 ºC) δ 6.48 (dd, J = 5.0, 2.5 Hz, 1H), 4.43 (dd, J = 9.7, 6.3 Hz, 1H), 4.22 (dd, J = 9.6, 2.7 Hz, 1H), 3.70 – 3.65 (m, 1H), 3.28 – 3.24 (m, 1H), 2.86 – 2.78 (m, 1H), 2.43 (td, J = 13.5, 4.9 Hz, 1H), 2.12 (qd, J = 13.6, 7.1 Hz, 1H). ^{13}C{^{1}H} NMR (100 MHz, $CDCl_3$) δ 173.1 (C), 126.4 (CH), 117.9 (C), 116.6 (C), 70.0 (CH_2), 42.2 (CH), 34.6 (CH), 31.9 (CH), 29.5 (CH_2). HRMS (ESI-TOF) (m/z):

$[M+Na]^+$ calcd for $C_9H_8BrNO_2Na$, 263.9631, 265.9610; Found, 263.9630, 265.9608. IR (neat, cm^{-1}) 2955, 2851, 1766, 1612, 1454, 1378, 1258, 1164, 986. mp 164.7-167.7 °C

(3a*R,5*S**,7a*R**)-7-methyl-1-oxo-1,3,3a,4,5,7a-hexahydroisobenzofuran-5-carbonitrile, 4d**

3d (50 mg, 0.23 mmol) was reacted according to the general procedure for 12 h. **4d** (22 mg, 52%) was isolated by column chromatography (EA/Hexs = 1:3) as a colorless oil. Spectral data of **4d**: ^1H NMR (400 MHz, $CDCl_3$, 24 °C) δ 5.59 (s, 1H), 4.36 (dd, *J* = 9.4, 5.7 Hz, 1H), 4.13 (d, *J* = 9.4 Hz, 1H), 3.35 – 3.31 (m, 1H), 2.98 (d, *J* = 7.2 Hz, 1H), 2.70 – 2.62 (m, 1H), 2.26 – 2.22 (m, 1H), 2.06 (s, 3H), 1.78 (q, *J* = 12.4 Hz, 1H). $^{13}C\{^1H\}$ NMR (100 MHz, $CDCl_3$) δ 174.2 (C), 133.2 (C), 120.3 (C), 118.2 (CH), 70.4 (CH_2), 43.1 (CH), 33.9 (CH), 27.5 (CH_2), 26.4 (CH), 21.8 (CH_3). HRMS (ESI-TOF) (m/z): $[M+Na]^+$ calcd for $C_{10}H_{11}NO_2Na$, 200.0682; Found, 200.0692. IR (neat, cm^{-1}) 2918, 2361, 1766, 1451, 1377, 1229, 1155, 978.

(3a*R,5*S**,7a*S**)-1-oxo-7-(trimethylsilyl)-1,3,3a,4,5,7a-hexahydroisobenzofuran-5-carbonitrile, 4e**

3e (23 mg, 0.10 mmol) was reacted according to the general procedure for 12 h. **4e** (18 mg, 88%) was isolated by column chromatography (EA/Hexs = 1:5) as a white solid. Spectral data of **4e**: ^1H NMR (400 MHz, $CDCl_3$, 24 °C) δ

4.39 (dd, J = 9.4, 5.6 Hz, 1H), 4.10 (d, J = 9.5 Hz, 1H), 3.33 – 3.28 (m, 2H), 2.61 – 2.54 (m, 1H), 2.27 – 2.22 (m, 1H), 1.79 (q, J = 12.8 Hz, 1H), 0.21 (s, 9H). $^{13}C\{^1H\}$ NMR (100 MHz, $CDCl_3$) δ 174.7 (C), 139.2 (C), 131.4 (CH), 120.2 (C), 70.6 (CH_2), 41.8 (CH), 33.6 (CH), 27.5 (CH_2), 27.5 (CH), -1.0 (3 x CH_3). HRMS (ESI-TOF) (m/z): $[M+K]^+$ calcd for $C_{12}H_{17}NO_2SiK$, 274.0660; Found, 274.0651. IR (neat, cm^{-1}) 2920, 1772, 1247, 1167, 981, 841. mp 113.4-115.9 °C

(3a*R,5*S**,7a*R**)-7-Allyl-1-oxo-1,3,3a,4,5,7a-hexahydroisobenzofuran-5-carbonitrile, 4f**

3f (50 mg, 0.21 mmol) was reacted according to the general procedure for 12 h. **4f** (37 mg, 84%) was isolated by column chromatography (EA/Hexs = 1:3) as a white solid. Spectral data of **4f**: ^1H NMR (400 MHz, $CDCl_3$, 24 °C) δ 5.82 – 5.72 (m, 1H), 5.62 (s, 1H), 5.17 (d, J = 5.0 Hz, 1H), 5.14 (s, 1H), 4.36 (dd, J = 9.5, 5.5 Hz, 1H), 4.13 (d, J = 9.5 Hz, 1H), 3.37 – 3.32 (m, 1H), 3.20 (d, J = 6.6 Hz, 2H), 3.10 (d, J = 7.2 Hz, 1H), 2.68 – 2.60 (m, 1H), 2.25 (td, J = 13.1, 4.6 Hz, 1H), 1.80 (q, J = 12.6 Hz, 1H). $^{13}C\{^1H\}$ NMR (100 MHz, $CDCl_3$) δ 174.0 (C), 135.7 (C), 134.2 (CH_2), 120.2 (C), 118.8 (CH), 118.5 (CH_2), 70.3 (CH), 40.9 (CH_2), 38.8 (CH), 33.9 (CH_2), 27.8 (CH), 26.5 (CH_2). HRMS (ESI-TOF) (m/z): $[M+Na]^+$ calcd for $C_{12}H_{13}NO_2Na$, 226.0838; Found, 226.0840. IR (neat, cm^{-1}) 2918, 1766, 1377, 1228, 1150, 1037, 987. mp 92.0-92.4 °C

(3a*R,5*S**,7a*S**)-7a-methyl-1-oxo-1,3,3a,4,5,7a-hexahydroisobenzofuran-5-carbonitrile, 4g**

3g (64 mg, 0.30 mmol) was reacted according to the general procedure for 12 h. **4g** (38 mg, 69%) was isolated by column chromatography (EA/Hexs = 1:5) as a white solid. Spectral data of **4g**: ^{1}H NMR (400 MHz, $CDCl_3$, 24 °C) δ 5.93 (dd, *J* = 2.5, 9.9 Hz, 1H), 5.86 (dd, *J* = 2.6, 9.9 Hz, 1H), 4.46 (dd, *J* = 6.6, 9.6 Hz, 1H), 4.20 (dd, *J* = 4.0, 9.6 Hz, 1H), 3.34 – 3.28 (m, 1H), 2.47 – 2.42 (m, 1H), 2.23 (td, *J* = 5.0, 13.7 Hz, 1H), 1.97 – 1.93 (m, 1H), 1.35 (s, 3H). ^{13}C{^{1}H} NMR (100 MHz, $CDCl_3$) δ 177.7 (C), 131.2 (CH), 122.4 (CH), 120.0 (C), 68.9 (CH_2), 42.8 (C), 39.8 (CH_2), 27.1 (CH), 24.7 (CH), 22.9 (CH_3). HRMS (ESI-TOF) (m/z): $[M+K]^+$ calcd for $C_{10}H_{11}NO_2K$, 216.0421; Found, 216.0431. IR (neat, cm^{-1}) 2925, 1768, 1200, 1088, 985. mp 77.3-78.6 °C

(3a*S,5*S**,7a*R**)-7a-methoxy-1-oxo-1,3,3a,4,5,7a-hexahydroisobenzofuran-5-carbonitrile, 4h**

3h (100 mg, 0.44 mmol) was reacted according to the general procedure for 12 h. **4h** (80 mg, 93%) was isolated by column chromatography (EA/Hexs = 1:3) as a colorless oil. Spectral data of **4h**: ^{1}H NMR (400 MHz, $CDCl_3$, 24 °C) δ 6.17 (s, 2H), 4.56 (dd, *J* = 9.3, 6.5 Hz, 1H), 4.12 (dd, *J* = 9.4, 3.6 Hz, 1H), 3.42 (dd, *J* = 8.9, 5.3 Hz, 1H), 3.39 (s, 3H), 2.76 – 2.70 (m, 1H), 2.31 (td, *J* = 13.9, 5.0 Hz, 1H), 1.99 (qd, *J* = 13.9, 10.8, 8.9 Hz, 1H). ^{13}C{^{1}H} NMR (100 MHz, $CDCl_3$) δ 172.8 (C), 128.7 (CH), 125.6 (CH), 119.5 (C), 75.8 (C), 69.2

(CH_2), 52.4 (CH_3), 38.4 (CH), 29.7 (CH_2), 27.4 (CH_2), 25.2 (CH). HRMS (ESI-TOF) (m/z): $[M+K]^+$ calcd for $C_{10}H_{11}NO_3K$, 232.0371; Found, 232.0376. IR (neat, cm^{-1}) 2922, 1774, 1455, 1197, 1089, 970.

(3a*R,4*S**,5*S**,7a*S**)-7-bromo-4-methyl-1-oxo-1,3,3a,4,5,7a-hexahydroisobenzofuran-5-carbonitrile, 4i**

3i (22 mg, 0.12 mmol) was reacted according to the general procedure for 12 h. **4i** (12 mg, 58%) was isolated by column chromatography (EA/Hexs = 1:3) as a white solid. Spectral data of **4i**: ^{1}H NMR (400 MHz, $CDCl_3$, 24 °C) δ 6.26 (s, 1H), 4.34 – 4.27 (m, 1H), 3.48 (d, *J* = 7.1 Hz, 1H), 3.08 (td, *J* = 10.6, 2.3 Hz, 1H), 2.49 – 2.43 (m, 1H), 1.97 – 1.86 (m, 1H), 1.32 (d, *J* = 6.5 Hz, 2H). $^{13}C\{^1H\}$ NMR (100 MHz, $CDCl_3$) δ 171.5 (C), 125.2 (CH), 118.5 (C), 118.2 (C), 67.4 (CH_2), 46.8 (CH), 42.3 (CH), 36.7 (CH), 30.9 (CH), 17.3 (CH_3). HRMS (ESI-TOF) (m/z): $[M+Na]^+$ calcd for $C_{10}H_{10}BrNO_2Na$, 277.9787, 279.9767; Found, 277.9794, 279.9774. **IR** (neat, cm^{-1}) 2920, 1776, 1165, 1144. mp 160.4-163.7 °C.

(3a*R,4*S**,5*S**,7a*S**)-7-bromo-1-oxo-4-phenyl-1,3,3a,4,5,7a-hexahydroisobenzofuran-5-carbonitrile, 4j**

3j (26 mg, 0.074 mmol) was reacted according to the general procedure for 12 h. **4j** (9 mg, 36%) was isolated by column chromatography (EA/Hexs = 1:5) as a white solid. Spectral data of **4j**: ^{1}H NMR (400 MHz, $CDCl_3$, 24 °C) δ 7.46 – 7.39 (m, 3H), 7.25 (d, *J* = 6.8 Hz, 1H), 6.38 (t, *J* = 1.7 Hz, 2H), 4.11

(dd, J = 9.9, 5.3 Hz, 1H), 3.93 (d, J = 9.9 Hz, 1H), 3.63 (td, J = 6.9, 1.8 Hz, 1H), 3.55 (td, J = 2.2, 11.0 Hz, 1H), 3.04 (qd, J = 12.4, 4.1 Hz, 1H), 2.90 (dd, J = 11.8, 11.8 Hz, 1H). $^{13}C\{^1H\}$ NMR (100 MHz, $CDCl_3$) δ 166.1 (C), 136.3 (C), 129.7 (2 x CH), 129.1 (CH),127.1 (2 x CH), 126.1 (C), 126.0 (C), 118.1 (C), 69.1 (CH_2), 46.4(CH), 45.5 (CH), 40.7 (CH_2), 34.1 (CH). HRMS (ESI-TOF) (m/z): $[M+Na]^+$ calcd for $C_{15}H_{12}BrNO_2Na$, 339.9944, 341.9923. Found, 339.9941, 341.9922. IR (neat, cm^{-1}) 2960,1452, 1236, 1236, 1211, 1143, 1060, 1003, 945, 921, 853.

mp 209.7-211.1 °C

(4a*S,6*S**,8a*S**)-8-bromo-1-oxo-3,4,4a,5,6,8a-hexahydro-1*H*-isochromene-6-carbonitrile, 4k**

3k (50 mg, 0.17 mmol) was reacted according to the general procedure for 12 h. **4k** (15 mg, 33%) was isolated by column chromatography (EA/Hexs = 1:3) as a white solid. Spectral data of **4k**: ^1H NMR (400 MHz, $CDCl_3$, 24 °C) δ 6.29 (t, J = 1.1 Hz, 1H), 4.38 (ddd, J = 11.8, 5.5, 2.4 Hz, 1H), 4.30 (ddd, J = 12.0, 12.0, 2.8 Hz, 1H), 3.50 (d, J = 7.5 Hz, 1H), 3.41 (tdd, J = 11.1, 5.3, 2.7 Hz, 1H), 2.56-2.28 (m, 1H), 2.33 (tdd, J = 14.7, 9.6, 2.6 Hz, 1H), 2.26-2.19 (m, 1H), 1.72-1.59 (m, 2H), 1.25 (s, 1H). $^{13}C\{^1H\}$ NMR (100 MHz, $CDCl_3$) δ 169.0 (C), 125.8 (CH), 120.8 (C), 118.2 (C), 65.7 (CH), 47.5 (CH_2), 31.2 (CH_2), 30.6 (CH), 29.7 (CH), 29.2 (CH_2), 29.1 (CH). HRMS (ESI-TOF) (m/z): $[M+Na]^+$ calcd for $C_{10}H_{10}BrNO_2Na$ $[M+Na]^+$, 277.9787, 279.9767.

Found, 277.9782, 279.9762. IR (neat, cm^{-1}) 2925, 1741, 1163, 1083, 856. mp 132.7-133.9 °C. Crystal for X-ray crystallography was recrystallized from gas-diffusion in ether/ CH_2Cl_2. (CCDC 2386154)

General procedure for the hydrolysis of 6 using 7a as an example.
To a solution of **6a** (89 mg, 0.21 mmol, 1 eq) in 1 mL of DCM was added *p*-TSA(36mg, 0.21 mmol, 1 eq). The reaction was then stirred at rt temperature for 2h. 30 mL of saturated $NaHCO_3$ (aq) was added to the reaction mixture and then extracted with CH_2Cl_2. The organic phase was collected and dried with anhydrous $MgSO_4$ and solvent was removed using rotavap. The crude mixture was washed with 30 mL of ether to give the product **7a** (49 mg, 62 % yield) as a white solid.

(3a*S,4*S**,7*S**,7a*S**,*E*)-6-bromo-9-(hydroxyimino)-2-phenyl-3a,4,7,7a-tetrahydro-1*H*-4,7-ethanoisoindole-1,3,8(2*H*)-trione, 7a**

Spectral data of **7a**: **^{1}H NMR** (400 MHz, $CDCl_3$, 24 °C, δ) 8.80 (br, 1H), 7.55 – 7.44 (m, 3H), 7.28 – 7.23 (m, 2H), 6.68 (dd, *J* = 6.9, 2.2 Hz, 1H), 5.10 (dd, *J* = 6.8, 3.5 Hz, 1H), 4.24 (dd, *J* = 3.1, 2.2 Hz, 1H), 3.60 (dd, *J* = 8.4, 3.2 Hz, 1H), 3.45 (dd, *J* = 8.4, 3.4 Hz, 1H). $^{13}C\{^1H\}$ NMR (100 MHz, $CDCl_3$) 188.0 (C), 173.9 (C), 172.8 (C), 131.2 (C), 130.8 (CH), 129.4 (CH), 129.3 (CH), 126.3 (CH), 119.1 (C), 58.7 (CH), 42.2 (CH), 41.3 (CH), 35.9 (CH). HRMS (ESI-TOF) (m/z): $[M+Na]^+$ calcd for $C_{16}H_{11}BrN_2O_4Na$, 396.9794, 398.9774 Found. 396.9804, 398.9784. IR (neat, cm^{-1}) : 3346, 2922, 2851, 1721, 1630, 1461, 1370, 1275, 1091, 749. mp 242.6-242.9 °C. Crystal for X-ray crystallography was recrystallized from gas-diffusion in ether/ CH_2Cl_2. (CCDC

2386155)

(3a*S,4*R**,7*R**,7a*R**,*E*)-9-(hydroxyimino)-6-methyl-2-phenyl-3a,4,7,7a-tetrahydro-1*H*-4,7-ethanoisoindole-1,3,8(2*H*)-trione, 7c**

6c (356 mg, 1 mmol) was reacted according to the general procedure for 2 h. **7c** (220 mg, 70%) was isolated by washing the crude mixture with 50 mL of ether as a white solid. Spectral data of **7c**: ^{1}H NMR (400 MHz, $CDCl_3$, 24 °C, δ) 9.07 (br, 1H), 7.51 - 7.39 (m, 3H), 7.19 - 7.16 (m, 2H), 6.09 (dt, *J* = 6.5, 1.6 Hz, 1H), 4.93 (dd, *J* = 6.3, 3.3 Hz, 1H), 3.85 (dd, *J* = 3.0, 1.8 Hz, 1H), 3.50 (dd, *J* = 8.4, 3.2 Hz, 1H), 3.40 (dd, *J* = 8.5, 3.3 Hz, 1H), 1.94 (d, *J* = 1.4 Hz, 3H). ^{13}C{^{1}H} NMR (100 MHz, $CDCl_3$) 190.9 (C), 174.8 (C), 174.4 (C), 149.1 (C), 140.5 (C), 131.4 (C), 129.4 (CH), 129.1 (CH), 126.3 (CH), 123.8 (CH), 54.6 (CH), 42.5 (CH), 40.8 (CH), 33.9 (CH), 21.1 (CH_3). HRMS (ESI-TOF) (m/z): $[M+H]^+$ calcd for $C_{17}H_{15}N_2O_4$, 311.1032; Found, 311.1015. IR (neat, cm^{-1}): 3345, 2955, 2850, 1721, 1631, 1598, 1468, 1326, 1191, 1124, 1017.

mp 229.5-231.7 °C

(3a*S,4*R**,7*R**,7a*R**,*E*)-6-(5,5-Dimethyl-1,3-dioxan-2-yl)-9-(hydroxyimino)-2-phenyl-3a,4,7,7a-tetrahydro-1*H*-4,7-ethanoisoindole-1,3,8(2*H*)-trione, 7d**

6d (97 mg, 0.21 mmol) was reacted according to the general procedure for 2 h. **7d** (56 mg, 66%) was isolated by washing the crude mixture with 50 mL of ether as a white solid. Spectral data of **7d**: ^{1}H NMR (400 MHz, $CDCl_3$, 24 °C,

δ) 10.02 (s, 1H), 7.47 – 7.35 (m, 3H), 7.24 – 7.18 (m, 2H), 6.50 (d, *J* = 6.4 Hz, 1H), 5.03 (dd, *J* = 6.5, 3.2 Hz, 1H), 4.89 (s, 1H), 4.17 (t, *J* = 2.1 Hz, 1H), 3.68 – 3.60 (m, 2H), 3.50 – 3.43 (m, 4H), 1.17 (s, 3H), 0.73 (s, 3H). $^{13}C\{^{1}H\}$ NMR (100 MHz, $CDCl_3$) 190.7 (C), 174.9 (C), 173.9 (C), 147.9 (C), 139.7 (C), 131.6 (C), 129.2 (CH), 129.0 (CH), 127.8 (CH), 126.6 (CH), 98.4 (CH), 77.2 (CH_2), 77.1 (CH_2), 49.6 (CH), 42.6 (CH), 40.8 (CH), 33.3 (CH), 30.2 (C), 22.9 (CH_3), 21.8 (CH_3). HRMS (ESI-TOF) (m/z): $[M+H]^+$ calcd for $C_{22}H_{23}N_2O_6$, 411.1556; Found, 411.1543. IR (neat, cm^{-1}) : 3345, 2955, 2851, 1720, 1711, 1467, 1383, 1017, 749. mp 232.8-235.4 °C

(3a*S,4*R**,7*R**,7a*R**,*E*)-6-allyl-9-(hydroxyimino)-2-phenyl-3a,4,7,7a-tetrahydro-1*H*-4,7-ethanoisoindole-1,3,8(2*H*)-trione, 7e**

6e (100 mg, 0.26 mmol) was reacted according to the general procedure for 2 h. **7e** (69 mg, 80%) was isolated by washing the crude mixture with 50 mL of ether as a white solid. Spectral data of **7e**: ^{1}H NMR (400 MHz, $CDCl_3$, 24 °C, δ) 9.24 (br, 1H), 7.52 – 7.40 (m, 3H), 7.20 – 7.14 (m, 2H), 6.11 (dd, *J* = 6.2, 1.5 Hz, 1H), 5.74 – 5.61 (m, 1H), 5.18 – 5.06 (m, 2H), 4.98 (dd, *J* = 6.4, 3.3 Hz, 1H), 3.90 (dd, *J* = 3.1, 1.8 Hz, 1H), 3.50 (dd, *J* = 8.4, 3.1 Hz, 1H), 3.43 (dd, *J* = 8.6, 3.3 Hz, 1H), 2.96 (d, *J* = 6.6 Hz, 2H). $^{13}C\{^{1}H\}$ NMR (100 MHz, $CDCl_3$) 190.8 (C),174.6 (C), 174.3 (C), 149.1 (C), 142.7 (C), 132.0 (CH) 131.4 (C), 129.4 (CH), 129.2 (CH), 126.3 (CH), 123.8 (CH), 119.1 (CH_2),

53.5 (CH), 42.5 (CH), 40.9 (CH), 39.1 (CH_2), 33.9 (CH), 1.0 (C). HRMS (ESI-TOF) (m/z): $[M+H]^+$ calcd for $C_{19}H_{17}N_2O_4$, 337.1183; Found. 337.1186 IR (neat, cm^{-1}) : 3316, 2923, 2851, 1736, 1461, 1379, 1261, 1091, 749. mp 146.6-147.8 °C

(3a*S,4*S**,7*S**,7a*S**,*E**)-9-(hydroxyimino)-2-phenyl-6-(trimethylsilyl)-3a,4,7,7a-tetrahydro-1*H*-4,7-ethanoisoindole-1,3,8(2*H*)-trione, 7f**

6f (100 mg, 0.24 mmol) was reacted according to the general procedure for 2 h. **7f** (51 mg, 54%) was isolated by washing the crude mixture with 50 mL of ether as a white solid. Spectral data of **7f**: ^{1}H NMR (400 MHz, $CDCl_3$, 24 °C, δ) 8.85 (s, 1H), 7.58 – 7.39 (m, 1H), 7.20 (d, *J* = 7.8 Hz, 1H), 6.74 (d, *J* = 5.8 Hz, 1H), 5.06 (dd, *J* = 5.9, 3.1 Hz, 1H), 4.11 (s, 1H), 3.51 (dd, *J* = 8.9, 2.8 Hz, 1H), 3.45 (dd, *J* = 8.5, 3.5 Hz, 1H), 0.11 (s, 9H). $^{13}C\{^1H\}$ NMR (100 MHz, $CDCl_3$) 191.2 (C), 174.8 (C), 174.3 (C), 148.5 (C), 146.8 (C), 139.2 (CH), 129.3 (CH), 129.1 (CH), 126.2 (CH), 52.1 (CH), 42.0 (CH), 40.4 (CH), 34.6 (CH), -2.5 (3 x CH_3). HRMS (ESI-TOF) (m/z): $[M+H]^+$ calcd for $C_{19}H_{21}N_2O_4Si$ $[M + H]^+$, 369.1265; Found, 369.1271. IR (neat, cm^{-1}) : 3345, 2951, 2851, 1721, 1598, 1483, 1426, 1382, 1275, 1017, 764. mp 236.2-238.1 °C

(3a*S,4*S**,7*S**,7a*R**,*E*)-9-(hydroxyimino)-5-methyl-2-phenyl-3a,4,7,7a-**

tetrahydro-1*H*-4,7-ethanoisoindole-1,3,8(2*H*)-trione, 7g

6g (100 mg, 0.28 mmol) was reacted according to the general procedure for 2 h. **7g** (42 mg, 48%) was isolated by washing the crude mixture with 50 mL of ether as a white solid. Spectral data of **7g**: ^{1}H NMR (400 MHz, $CDCl_3$, 24 °C, δ) 10.51 (br, 1H), 7.52 - 7.38 (m, 1H), 7.16 (d, J = 7.5 Hz, 1H), 6.00 (d, J = 6.1 Hz, 1H), 4.83 (s, 1H), 3.86 (dd, J = 6.7, 1.7 Hz, 1H), 3.47 (s, 2H), 1.95 (s, 3H). ^{13}C{^{1}H} NMR (100 MHz, $CDCl_3$) 191.8 (C), 175.0 (C), 174.9 (C), 148.2 (C), 143.2 (C), 131.4 (C), 129.3 (CH), 129.1 (CH), 126.3 (CH), 121.3 (CH), 49.6 (CH), 41.8 (CH), 41.4 (CH), 38.7 (CH), 21.1 (C). HRMS (ESI-TOF) (m/z): $[M+Na]^+$ calcd for $C_{17}H_{14}N_2NaO_4$, 333.0846; Found, 333.0854. IR (neat, cm^{-1}) : 3346, 2956, 2851, 1736, 1692, 1493, 1382, 1275, 1188, 1001, 764. mp 267.2-268.9 °C

(3a*R,4*R**,7*R**,7a*R**,*E*)-5-bromo-9-(hydroxyimino)-2-phenyl-3a,4,7,7a-tetrahydro-1*H*-4,7-ethanoisoindole-1,3,8(2*H*)-trione, 7h**

6h (99 mg, 0.24 mmol) was reacted according to the general procedure for 2 h. **7h** (91 mg, 99%) was isolated by washing the crude mixture with 50 mL of ether as a white solid. Spectral data of **7h**: ^{1}H NMR (400 MHz, $CDCl_3$, 24 °C, δ) 9.75 (s, 1H), 7.57 – 7.36 (m, 3H), 7.30 – 7.18 (m, 2H), 6.61 (dd, J = 6.9, 2.4 Hz, 1H), 5.18 (s, 1H), 4.04 (d, J = 6.7 Hz, 1H), 3.51 (s, 2H). ^{13}C{^{1}H} NMR (100 MHz, $CDCl_3$) 189.1 (C), 173.9 (C), 173.1 (C), 146.6 (C), 131.2 (C), 129.5 (CH), 129.3 (CH), 128.9 (CH), 126.4 (CH), 121.5 (C), 51.8 (CH), 43.7

(CH), 42.5 (CH), 41.2 (CH). HRMS (ESI-TOF) (m/z): $[M+Na]^+$ calcd for $C_{16}H_{11}BrN_2NaO_4$, 396.9794, 398.9774; Found, 396.9804, 398.9784. IR (neat, cm^{-1}): 3346, 2955, 2850, 1721, 1589, 1467, 1382, 1124, 1091. mp 244.9 °C (decomposed)

(3a*S,4*S**,7*S**,7a*R**,*E*)-5-(5,5-dimethyl-1,3-dioxan-2-yl)-9-(hydroxyimino)-2-phenyl-3a,4,7,7a-tetrahydro-1*H*-4,7-ethanoisoindole-1,3,8(2*H*)-trione, 7i**

6i (100 mg, 0.21 mmol) was reacted according to the general procedure for 2 h. **7i** (34 mg, 40%) was isolated by washing the crude mixture with 50 mL of ether as a white solid. Spectral data of **7i**: ^{1}H NMR (400 MHz, $CDCl_3$, 24 °C, δ) 8.82 (s, 1H), 7.50 – 7.36 (m, 3H), 7.25 – 7.20 (m, 2H), 6.48 (d, J = 6.5 Hz, 1H), 5.20 (dd, J = 3.1, 2.1 Hz, 1H), 4.91 (d, J = 0.8 Hz, 1H), 4.06 (dd, J = 6.5, 3.3 Hz, 1H), 3.70 – 3.61 (m, 2H), 3.53 – 3.45 (m, 3H), 3.38 (dd, J = 8.5, 3.2 Hz, 1H), 1.19 (s, 3H), 0.74 (s, 3H). $^{13}C\{^1H\}$ NMR (100 MHz, $CDCl_3$) 190.9 (C), 174.3 (C), 173.8 (C), 148.5 (C), 131.6 (C), 129.2 (CH), 129.0 (CH), 126.6 (CH), 125.5 (CH), 98.3 (CH), 77.3 (CH_2), 77.1 (CH_2), 49.2 (CH), 41.9 (CH), 41.6 (CH), 34.3 (CH), 30.2 (C), 22.9 (CH_3), 21.8 (CH_3). HRMS (ESI-TOF) (m/z): $[M+H]^+$ calcd for $C_{22}H_{23}N_2O_6$, 411.1551; Found, 411.1555. IR (neat, cm^{-1}) : 3392, 2923, 1712, 1379, 1018, 749. mp 167.3 °C (decompose)

Methyl (3a*S,4*S**,7*S**,7a*R**,*E*)-9-(hydroxyimino)-1,3,8-trioxo-2-phenyl-**

2,3,3a,4,7,7a-hexahydro-1*H*-4,7-ethanoisoindole-5-carboxylate, 7j

6j (50 mg, 0.13 mmol) was reacted according to the general procedure for 2 h. **7j** (27 mg, 66%) was isolated by washing the crude mixture with 50 mL of ether as a white solid. Spectral data of **7j**: ^{1}H NMR (400 MHz, $CDCl_3$, 24 °C, δ) 8.72 (s, 1H), 7.48 – 7.39 (m, 3H), 7.35 (dd, J = 6.7, 2.1 Hz, 1H), 7.14 – 7.09 (m, 2H), 5.63 (dd, J = 3.4, 2.2 Hz, 1H), 4.23 (dd, J = 6.6, 3.0 Hz, 1H), 3.83 (s, 3H), 3.57 (dd, J = 8.6, 2.7 Hz, 1H), 3.50 (dd, J = 8.5, 3.4 Hz, 1H). HRMS (ESI-TOF) (m/z): $[M+H]^+$ calcd for $C_{18}H_{15}N_2O_6$ $[M + H]^+$, 355.0925; Found, 355.0932. IR (neat, cm^{-1}) : 3346, 2920, 2840, 1728, 1387, 1182, 1088, 939, 888, 775. mp 234.8 °C (decompose)

(3a*S,4*R**,7*S**,7a*S**,*E*)-9-(hydroxyimino)-7-methyl-2-phenyl-3a,4,7,7a-tetrahydro-1*H*-4,7-ethanoisoindole-1,3,8(2*H*)-trione, 7k**

6k (200 mg, 0.56 mmol) was reacted according to the general procedure for 2 h. **7k** (131 mg, 75%) was isolated by washing the crude mixture with 50 mL of ether as a white solid. Spectral data of **7k**: ^{1}H NMR (400 MHz, $CDCl_3$, 24 °C, δ) 7.51 – 7.39 (m, 3H), 7.23 – 7.18 (m, 2H), 6.48 (dd, J =7.9, 6.4 Hz, 1H), 6.15 (dd, J = 8.1, 1.0 Hz, 1H), 5.03 (ddd, J = 6.3, 3.3, 1.7 Hz, 1H), 3.44 (dd, J = 7.4, 3.3 Hz, 1H), 3.08 (d, J = 8.6 Hz, 1H), 1.72 (s, 3H). $^{13}C\{^1H\}$ NMR (100 MHz, $CDCl_3$) 148.0, 139.3, 135.8, 131.2, 129.3, 129.3, 129.1, 129.0, 128.9, 126.4, 60.4, 47.4, 44.7, 43.5, 43.2, 33.3, 21.1, 16.9, 14.8, 14.2. HRMS (ESI-TOF) (m/z): $[M+Na]^+$ calcd for $C_{17}H_{14}N_2NaO_4$, 333.0846; Found,

333.0853. IR (neat, cm^{-1}): 3412, 2924, 1710, 1614, 1588, 1487, 1275, 1091, 762. mp 225.3 °C (decompose)

(3a*S,4*R**,7*R**,7a*S**,*E*)-9-(hydroxyimino)-7-methoxy-2-phenyl-3a,4,7,7a-tetrahydro-1*H*-4,7-ethanoisoindole-1,3,8(2*H*)-trione, 7l**

6l (94 mg, 0.25 mmol) was reacted according to the general procedure for 2 h. **7l** (48 mg, 57%) was isolated by washing the crude mixture with 50 mL of ether as a white solid. Spectral data of **7l**: ^{1}H NMR (400 MHz, $CDCl_3$, 24 °C, δ) 8.50 (br, 1H), 7.53 - 7.40 (m, 3H), 7.27 - 7.22 (m, 2H), 6.46 - 6.43 (m, 2H), 5.03 (dt, *J* = 4.8, 3.4 Hz, 1H), 3.85 (s, 3H), 3.69 (d, *J* = 8.7 Hz, 1H), 3.52 - 3.47 (m, 2H). $^{13}C\{^1H\}$ NMR (100 MHz, $CDCl_3$) 173.9 (C), 171.7 (C), 133.3 (C), 131.3 (C), 129.8 (CH), 129.3 (CH), 129.1 (CH), 126.3 (CH), 85.3 (C), 54.3 (CH_3), 42.7 (CH), 41.0 (CH), 32.9 (CH), 21.1 (C), 14.2 (C). HRMS (ESI-TOF) (m/z): $[M+H]^+$ calcd for $C_{17}H_{15}N_2O_5$, 327.0976; Found, 327.0978. IR (neat, cm^{-1}) : 3328, 2953, 2850, 1737, 1383, 1262, 1088, 939, 867, 753, 624.

mp 225.3-227.2 °C

(3a*R,4*R**,7*S**,7a*S**,*E*)-9-(hydroxyimino)-3a,4,7,7a-tetrahydro-1*H*-4,7-ethanoinden-8-one, 7m**

6m (302 mg, 1.3 mmol) was reacted according to the general procedure for 2 h. **7m** (186 mg, 77%) was isolated by column chromatography (EA/Hexs = 1:3) as a colorless oil. Spectral data of **7m**: ^{1}H NMR (400 MHz, $CDCl_3$, 24 °C, δ) 8.71 (s, 1H), 6.29 - 6.22 (m, 2H), 5.71 (qd, *J* = 5.7, 2.2 Hz, 1H), 5.53

(qd, J = 6.0, 2.0 Hz, 1H), 4.41 - 4.37 (m, 1H), 3.46 - 3.41 (m, 1H), 3.27 - 3.21 (m, 1H), 2.90 - 2.82 (m, 1H), 2.63 (qdd, J = 17.3, 10.1, 2.1 Hz, 1H), 2.10 - 2.01 (m, 1H). $^{13}C\{^1H\}$ NMR (100 MHz, $CDCl_3$) 195.3 (C), 133.8 (CH), 133.0 (CH), 130.2 (CH), 128.3 (CH), 53.9 (CH), 49.9 (CH), 38.7 (CH_2), 36.5 (CH), 36.2 (CH). HRMS (ESI-TOF) (m/z): $[M+Na]^+$ calcd for $C_{11}H_{11}NNaO_2$, 212.0682; Found, 212.0679. IR (neat, cm^{-1}) : 3352, 2954, 2849, 1736, 1692, 1413, 1382, 1186, 1092, 891, 714.

(3a*R,4*S**,7*R**,7a*S**,*E*)-9-(hydroxyimino)-5-methyl-3a,4,7,7a-tetrahydro-1H-4,7-ethanoinden-8-one, 7o**

6o (301 mg, 1.2 mmol) was reacted according to the general procedure for 2 h. **7o** (239 mg, 98%) was isolated by column chromatography (EA/Hexs = 1:3) as a colorless oil. Spectral data of **7o**: ^1H NMR (400 MHz, $CDCl_3$, 24 °C, δ) 10.08 (s, 1H), 5.79 (d, J = 6.3 Hz, 1H), 5.71 - 5.67 (m, 1H), 5.50 – 5.46 (m, 1H), 4.18 (t, J = 2.5 Hz, 1H), 3.29 – 3.20 (m, 1H), 2.83 – 2.75 (m, 1H), 2.59 (qdd, J = 17.2, 10.0, 2.1 Hz, 1H), 2.12 – 2.03 (m, 1H), 1.79 (d, J = 1.6 Hz, 3H). $^{13}C\{^1H\}$ NMR (100 MHz, $CDCl_3$) 196.0 (C), 151.7 (C), 142.9 (C), 133.6 (CH), 129.6 (CH), 119.9 (CH), 53.3 (CH), 49.7 (CH), 41.6 (CH), 38.6 (CH), 38.6 (CH_2), 36.1 (CH), 21.8 (CH_3). HRMS (ESI-TOF) (m/z): $[M+Na]^+$ calcd for $C_{12}H_{13}NNaO_2$, 226.0839; Found, 226.0834. IR (neat, cm^{-1}) : 3269, 3047, 2955, 2850, 1726, 1641, 1442, 1413, 1264, 1090, 1051, 934, 894, 786, 646.

(1*R,4*R**,8*R**,*E*)-3-(hydroxyimino)-8-phenylbicyclo[2.2.2]oct-5-en-2-one, 7p**

6o (636 mg, 2.3 mmol) was reacted according to the general procedure for 2 h. **7o** (501 mg, 79%) was isolated by column chromatography (EA/Hexs = 1:5) as a colorless oil. Spectral data of **7o**: ^{1}H NMR (400 MHz, $CDCl_3$, 24 °C, δ) 7.37 - 7.20 (m, 5H), 6.58 (dt, *J* = 7.3, 1.6 Hz, 1H), 6.34 (t, *J* = 7.1 Hz, 1H), 4.49 (td, *J* = 1.9, 6.3 Hz, 1H), 3.55 - 3.49 (m, 1H), 3.43 - 3.35 (m, 1H), 2.55 (ddd, *J* = 13.5, 10.4, 2.8 Hz, 1H), 1.98 (ddd, *J* = 13.8, 5.9, 3.0 Hz, 1H). ^{13}C{^{1}H} NMR (100 MHz, $CDCl_3$) 195.4 (C), 151.5 (C), 143.9 (C), 132.0 (CH), 131.6 (CH), 128.5 (CH), 127.9 (CH), 126.9 (CH), 49.1 (CH), 40.6 (CH), 39.2 (CH), 31.6 (CH_2). HRMS (ESI-TOF) (m/z): $[M+H]^+$ calcd for $C_{14}H_{14}NO_2$, 228.1019; Found, 228.1019. IR (neat, cm^{-1}): 3210, 2954, 2850, 1736, 1493, 1461, 1325, 1178, 1007, 863, 699.

(1*R,4*R**,8*R**,*E*)-3-(hydroxyimino)-6-methyl-8-phenylbicyclo[2.2.2]oct-5-en-2-one, 7q**

6q (541 mg, 1.89 mmol) was reacted according to the general procedure for 2 h. **7q** (450 mg, 99%) was isolated by as a colorless oil without further purification. Spectral data of **7q**: ^{1}H NMR (400 MHz, $CDCl_3$, 24 °C, δ) 9.53 (br, 1H), 7.35 - 7.16 (m, 1H), 5.96 (d, *J* = 6.2 Hz, 1H), 4.40 (dd, *J* = 6.3, 2.0 Hz, 1H), 3.42 - 3.35 (t, *J* = 7.3 Hz, 1H), 3.34 (d, *J* = 1.7 Hz, 1H), 2.54

(ddd, J = 13.4, 10.4, 2.7 Hz, 1H), 2.02 (d, J = 1.4 Hz, 3H), 1.95 (ddd, J = 13.8, 5.9, 2.9 Hz, 1H). $^{13}C\{^1H\}$ NMR (100 MHz, $CDCl_3$) 195.5 (C), 152.2 (C), 144.2 (C), 141.4 (C), 128.5 (CH), 127.8 (CH), 126.8 (CH), 124.0 (CH), 54.5 (C), 41.2 (C), 38.9 (C), 31.4 (CH_2), 20.4 (CH_3). HRMS (ESI-TOF) (m/z): $[M+Na]^+$ calcd for $C_{15}H_{15}NNaO_2$, 264.0995; Found, 264.0997. IR (neat, cm^{-1}) : 3279, 2955, 2850, 1736, 1620, 1598, 1493, 1006, 894, 699.

(1R*,4S*,8R*,E)-3-(hydroxyimino)-5-methyl-8-phenylbicyclo[2.2.2]oct-5-en-2-one, 7r

6r (301 mg, 1.05 mmol) was reacted according to the general procedure for 2 h. **7r** (221 mg, 87%) was isolated by column chromatography (EtOAc: hexanes = 1: 3) as a colorless oil. Spectral data of **7r**: ^1H NMR (400 MHz, $CDCl_3$, 24 °C, δ) 9.53 (br, 1H), 7.31 - 7.21 (m, 1H), 6.17 (td, J = 6.5, 1.6 Hz, 1H), 4.20 (t, J = 2.2 Hz, 1H), 3.46 - 3.36 (m, 2H), 2.46 (ddd, J = 13.8, 10.1, 2.3 Hz, 1H), 2.04 (ddd, J = 14.1, 5.2, 3.1 Hz, 1H), 1.64 (d, J = 1.6 Hz, 3H). $^{13}C\{^1H\}$ NMR (100 MHz, $CDCl_3$) 195.8 (C), 151.4 (C), 143.6 (C), 141.8 (C), 128.5 (CH), 127.7 (CH), 126.9 (CH), 123.9 (CH), 48.4 (CH), 45.3 (CH), 40.6 (CH), 30.8 (CH_2), 22.1 (CH_3). HRMS (ESI-TOF) (m/z): $[M+H]^+$ calcd for $C_{15}H_{16}NO_2$, 242.1176; Found, 242.1171. IR (neat, cm^{-1}) : 3347, 2956, 2850, 1725, 1461, 1451, 1261,1091, 750.

General procedure for the oxidative cleavage of 7 using 8a as an example. To a solution of **7a** (0.100 g, 0.26 mmol, 1 eq) in 26 mL of MeOH was added TFA(0.26mL) and DAIB (0.258g, 0.78mmol, 3 eq). The reaction was then stirred at rt temperature for 24h. 30 mL of saturated $NaHCO_3$ (aq) was added to the reaction mixture and then extracted with CH_2Cl_2. The organic phase was collected and dried with anhydrous $MgSO_4$ and solvent was removed using rotavap. **8a** (59 mg, 54 % yield) was isolated by column chromatography (EtOAc: hexanes = 1: 1) as a white solid.

dimethyl (3a*S,4*S**,7*S**,7a*R**)-5-bromo-1,3-dioxo-2-phenyl-2,3,3a,4,7,7a-hexahydro-1*H*-isoindole-4,7-dicarboxylate, 8a(8h)**

Spectral data of **8a**: ^{1}H NMR (400 MHz, $CDCl_3$, 24 °C, δ) 7.54 - 7.37 (m, 3H), 7.30 - 7.23 (m, 2H), 6.48 (d, *J* = 8.0 Hz, 1H), 4.15 (s, 1H), 4.07 (d, *J* = 9.0 Hz, 1H), 3.94 (d, *J* = 9.5 Hz, 1H), 3.91 (d, *J* = 8.1 Hz, 1H), 3.78 (s, 3H), 3.74 (s, 3H). ^{13}C{^{1}H} NMR (100 MHz, $CDCl_3$) 176.9 (C), 176.3 (C), 170.0 (C), 169.4 (C), 131.5 (C), 129.3 (CH), 129.0 (CH), 127.5 (CH), 126.3 (CH), 119.7 (C), 53.3 (CH_3), 53.1 (CH_3), 49.8 (CH), 43.3 (CH), 43.0 (CH), 39.6 (CH). HRMS (ESI-TOF) (m/z): $[M+H]^+$ calcd for $C_{18}H_{17}BrNO_6$, 422.0234, 424.0213; Found. 422.0238, 424.0217. IR (neat, cm^{-1}) : 2955, 1741, 1493,

1275, 1199, 749. mp 116.2-118.3 °C.

dimethyl (3a*R,4*R**,7*S**,7a*S**)-1,3-dioxo-2-phenyl-2,3,3a,4,7,7a-hexahydro-1*H*-isoindole-4,7-dicarboxylate, 8c(8g)**

7c (100 mg, 0.32 mmol) was reacted according to the general procedure for 2 h. **8c** (71 mg, 62%) was isolated by column chromatography (EA/Hexs = 1:3) as a colorless oil. Spectral data of **8c(8g)**: ^{1}H NMR (400 MHz, $CDCl_3$, 24 °C, δ) 7.49 - 7.36 (m, 3H), 7.25 - 7.21 (m, 1H), 5.81 (dd, *J* = 7.7, 1.5 Hz, 1H), 3.97 (dd, *J* = 3.4, 1.0 Hz, 2H), 3.79 (d, *J* = 7.7 Hz, 1H), 3.71 (s, 3H), 3.70 (s, 4H), 1.94 (d, *J* = 0.8 Hz, 3H). $^{13}C\{^1H\}$ NMR (100 MHz, $CDCl_3$) 177.9 (C), 177.6 (C), 171.5 (C), 171.0 (C), 136.2 (C), 131.7 (C), 129.2 (CH), 128.8 (CH), 126.3 (CH), 120.1 (CH), 52.7 (CH_3), 45.2 (CH), 41.7 (CH), 41.4 (CH), 40.3 (CH), 24.4 (CH_3). HRMS (ESI-TOF) (m/z): $[M+Na]^+$ calcd for $C_{19}H_{19}NNaO_6$, 380.1104; Found. 380.1110 IR (neat, cm^{-1}) : 2921, 1737, 1598, 1450, 1370, 1261, 1124, 1091, 749. mp 121.7 °C (decompose)

dimethyl (3a*S,4*R**,7*R**,7a*R**)-5-(5,5-dimethyl-1,3-dioxan-2-yl)-1,3-dioxo-2-phenyl-2,3,3a,4,7,7a-hexahydro-1*H*-isoindole-4,7-dicarboxylate, 8d(8i)**

7d (100 mg, 0.24 mmol) was reacted according to the general procedure for 2 h. **8d** (47 mg, 40%) was isolated by column chromatography (EA/Hexs = 1:1) as a white solid. Spectral data of **8d(8i)**: **^{1}**H NMR (400 MHz, CDCl3, 24 °C,

δ) 7.52 - 7.37 (m, 1H), 7.26 - 7.22 (m, 1H), 6.37 (dd, *J* = 8.2, 0.8 Hz, 1H), 4.96 (d, *J* = 0.9 Hz, 1H), 4.13 (d, *J* = 1.0 Hz, 1H), 4.05 - 3.95 (m, H), 3.73 (s, 3H), 3.74 (s, 3H), 3.66 (ddd, *J* = 11.2, 5.3, 2.6 Hz, 2H), 3.50 (t, *J* = 11.5 Hz, 2H), 1.17 (s, 3H), 0.75 (s, 3H). $^{13}C\{^1H\}$ NMR (100 MHz, $CDCl_3$) 177.5 (C), 173.6 (C), 172.6 (C), 172.0 (C), 132.1 (CH), 131.8 (CH), 129.0 (CH), 128.5 (CH), 128.0 (CH), 126.4 (CH), 93.6 (C), 53.5 (CH_3), 53.1 (CH_3), 53.0 (CH_3), 48.8 (CH), 39.9 (CH), 38.6 (CH). HRMS (ESI-TOF) (m/z): $[M+Na]^+$ calcd for $C_{24}H_{27}NNaO_8$, 480.1629; Found, 480.1642. IR (neat, cm^{-1}) : 2955, 2850, 1725, 1442, 1306, 1092, 595. mp 155.3-160.4 °C

dimethyl (3a*S,4*R**,7*R**,7a*R**)-5-allyl-1,3-dioxo-2-phenyl-2,3,3a,4,7,7a-hexahydro-1*H*-isoindole-4,7-dicarboxylate, 8e**

7e (107 mg, 0.32 mmol) was reacted according to the general procedure for 2 h. **8e** (54 mg, 44%) was isolated by column chromatography (EA/Hexs = 1:1) as a colorless oil. Spectral data of **8e**: ^1H NMR (400 MHz, $CDCl_3$, 24 °C, δ)7.48 - 7.35 (m, 3H), 7.22 - 7.18 (m, 1H), 5.85 (dt, *J* = 7.7, 2.2 Hz, 1H), 5.65 (dddd, *J* = 16.5, 10.5, 7.9, 6.1 Hz, 1H), 5.12 (s, 1H), 5.10 - 5.06 (m, 1H), 3.98 (ddd, *J* = 17.8, 9.3, 1.1 Hz, 2H), 3.86 (d, *J* = 7.8 Hz, 1H), 3.75 (d, *J* = 0.8 Hz, 1H), 3.70 (s, 3H), 3.69 (s, 3H), 3.02 (ddd, *J* = 15.3, 6.1, 1.2 Hz, 1H), 2.93 (dd, *J* = 15.2, 7.9 Hz, 1H). $^{13}C\{^1H\}$ NMR (100 MHz, $CDCl_3$) 177.7 (C), 177.2 (C), 171.3 (C), 171.0 (C), 138.4 (C), 133.6 (CH), 131.7 (C), 129.2 (CH), 128.8 (CH), 126.2 (CH), 121.0 (CH), 118.3 (CH_2), 52.6 (CH_3), 52.6 (CH_3),43.3 (CH), 41.7 (CH), 41.5 (CH_2), 41.3 (CH), 40.6 (CH). HRMS (ESI-TOF) (m/z):

[M+Na]$^+$ calcd for $C_{21}H_{21}NNaO_6$, 406.1261; Found, 406.1263. IR (neat, cm^{-1}) : 2955, 1725, 1 1462, 1378, 1275, 1096, 764, 749.

dimethyl (3a*S,4*S**,7*S**,7a*R**)-1,3-dioxo-2-phenyl-5-(trimethylsilyl)-2,3,3a,4,7,7a-hexahydro-1*H*-isoindole-4,7-dicarboxylate, 8f**

7f (102 mg, 0.28 mmol) was reacted according to the general procedure for 2 h. **8f** (36 mg, 31%) was isolated by column chromatography (EA/Hexs = 1:4) as a white solid. Spectral data of **8f**: ^{1}H NMR (400 MHz, $CDCl_3$, 24 °C, δ) 7.45 - 7.35 (m, 3H), 7.21 - 7.16 (m, 2H), 6.41 (d, *J* = 7.7 Hz, 1H), 4.05 - 3.94 (m, 3H), 3.89 (dd, *J* = 7.8, 0.9 Hz, 1H), 3.73 (s, 3H), 3.69 (s, 3H), 0.13 (s, 9H). $^{13}C\{^1H\}$ NMR (100 MHz, $CDCl_3$) 178.0 (C), 177.4 (C), 171.6 (C), 171.0 (C), 142.0 (C), 134.5 (CH), 131.7 (C), 129.2 (CH), 128.8 (CH), 126.2 (CH), 52.7 (CH_3), 52.5 (CH_3), 42.1 (CH), 41.7 (CH), 41.5 (CH), 40.4 (CH), -2.2 (CH_3). HRMS (ESI-TOF) (m/z): [M+Na]$^+$ calcd for $C_{21}H_{25}NNaO_6Si$, 438.1343; Found, 438.1338. IR (neat, cm^{-1}) : 3180, 2916, 2848, 1721, 1383, 1084, 578.

mp 145.1-147.4 °C

dimethyl (3a*S,4*R**,7*R**,7a*R**)-4-methoxy-1,3-dioxo-2-phenyl-2,3,3a,4,7,7a-hexahydro-1*H*-isoindole-4,7-dicarboxylate, 8l**

7l(96 mg, 0.30 mmol) was reacted according to the general procedure for 2 h. **8l** (23 mg, 21%) was isolated by column chromatography (EA/Hexs = 1:3) as a white solid. Spectral data of **8l**: ^{1}H NMR (400 MHz, CDCl3, 24 °C, δ) 7.49 - 7.36 (m, 3H), 7.33 - 7.29 (m, 2H), 6.41 (dd, *J* = 9.6, 2.5 Hz, 1H), 6.13

(dd, J = 9.6, 3.6 Hz, 1H), 3.91 (dd, J = 10.4, 6.2 Hz, 1H), 3.91 (s, 3H), 3.89 (s, 3H), 3.67 (ddd, J = 6.5, 3.7, 2.7 Hz, 1H), 3.44 (d, J = 9.8 Hz, 1H), 3.29 (s, 3H). $^{13}C\{^1H\}$ NMR (100 MHz, $CDCl_3$) 177.5 (C), 173.6 (C), 172.6 (C), 172.0 (C), 132.1 (CH), 131.8 (CH), 129.0 (CH), 128.5 (CH), 128.0 (CH), 126.4 (CH), 93.6 (C), 53.5 (CH_3), 53.1 (CH_3), 53.0 (CH_3), 48.8 (CH), 39.9 (CH), 38.6 (CH). HRMS (ESI-TOF) (m/z): $[M+Na]^+$ calcd for $C_{19}H_{19}NNaO_7$, 396.1054 Found; 396.1058. IR (neat, cm^{-1}) :2955, 2850, 1737, 1467, 1380, 1344, 1292, 1090. mp 165.4-169.8 °C

dimethyl (3a*R,4*R**,7*S**,7a*S**)-3a,4,7,7a-tetrahydro-1*H*-indene-4,7-dicarboxylate, 8m**

7m(104 mg, 0.55 mmol) was reacted according to the general procedure for 2 h. **8m** (67 mg, 53%) was isolated by column chromatography (EA/Hexs = 1:5) as a colorless oil. Spectral data of **8m**: ^1H NMR (400 MHz, $CDCl_3$, 24 °C, δ) 6.03 (tdd, J = 20.5, 9.7, 2.6 Hz, 2H), 5.78 - 5.74 (m, 2H), 3.75 (s, 3H), 3.73 (s, 3H), 3.10 - 3.02(m, 1H), 2.82 (qdd, J = 18.5, 7.9, 2.6 Hz, 2H), 2.75 - 2.58 (m, 2H), 2.25 - 2.16 (m, 2H). $^{13}C\{^1H\}$ NMR (100 MHz, $CDCl_3$) 174.6 (C), 174.4 (C), 133.4 (CH), 130.5 (CH), 127.8 (CH), 127.7 (CH), 52.1 (CH), 45.7 (CH), 45.3 (CH), 44.7 (CH), 38.4 (CH_2), 38.2 (CH). HRMS (ESI-TOF) (m/z): $[M+Na]^+$ calcd for $C_{13}H_{16}NaO_4$, 259.0941; Found, 259.0938. IR (neat, cm^{-1}) : 2952, 1727, 1441, 1390, 1167, 716.

dimethyl (3a*R,4*S**,7*R**,7a*S**)-5-methyl-3a,4,7,7a-tetrahydro-1*H*-indene-4,7-dicarboxylate, 8o**

7o(200 mg, 0.98 mmol) was reacted according to the general procedure for 2 h. **8o** (77 mg, 29%) was isolated by column chromatography (EA/Hexs = 1:10) as a colorless oil. Spectral data of **8o**: ^{1}H NMR (400 MHz, $CDCl_3$, 24 °C, δ) 5.75 - 5.69 (m, 2H), 5.60 (dq, *J* = 6.0, 2.0 Hz, 1H), 3.72 (s, 3H), 3.71 (s, 3H), 3.24 - 3.18 (m, 1H), 2.91 - 2.76 (m, 3H), 2.60 (qdd, *J* = 16.2, 7.5, 2.3 Hz, 1H), 2.22 - 2.15 (m, 1H), 1.77 (t, *J* = 2.2 Hz, 3H). $^{13}C\{^{1}H\}$ NMR (100 MHz, $CDCl_3$) 174.7 (C), 174.0 (C), 134.2 (C), 133.5 (CH), 130.7 (CH), 122.5 (CH), 52.0 (CH_3), 51.9 (CH_3), 49.0 (CH), 46.7 (CH), 45.8 (CH), 39.3 (CH_2), 37.1 (CH), 21.7 (CH_3). HRMS (ESI-TOF) (m/z): $[M+Na]^+$ calcd for $C_{14}H_{18}NaO_4$, 273.1097; Found, 273.1095. IR (neat, cm^{-1}) : 2955, 2850, 1725, 1494, 1462, 1378, 1275, 1096, 764.

dimethyl (1*R,2*R**,5*R**)-1,2,5,6-tetrahydro-[1,1'-biphenyl]-2,5-dicarboxylate, 8p**

7p(100 mg, 0.44 mmol) was reacted according to the general procedure for 2 h. **8p** (69 mg, 57%) was isolated by column chromatography (EA/Hexs = 1:10) as a colorless oil. Spectral data of **8p**: ^{1}H NMR (400 MHz, $CDCl_3$, 24 °C, δ) 7.32 - 7.18 (m, 5H), 6.08 - 5.92 (m, 2H), 3.72 (s, 3H), 3.59 (s, 3H), 3.44 - 3.33 (m, 1H), 3.10 (s, 1H), 2.29 (dt, *J* = 13.6, 3.6 Hz, 1H), 1.96 (ddd, *J* =

13.9, 9.4, 5.5 Hz, 1H). $^{13}C\{^1H\}$ NMR (100 MHz, $CDCl_3$) 173.4 (C), 173.3 (C), 143.6 (C), 128.5 (CH), 127.2 (CH), 126.9 (CH), 126.7 (CH), 126.6 (CH), 52.1 (CH_3), 52.0 (CH_3), 47.8 (CH), 39.7 (CH), 39.0 (CH), 30.7 (CH_2). HRMS (ESI-TOF) (m/z): $[M+Na]^+$ calcd for $C_{16}H_{18}NaO_4$, 297.1097; Found, 297.1090. IR (neat, cm^{-1}) : 2954, 2849, 1737, 1658, 1580, 1493, 1385, 1344, 1198, 761.

dimethyl (1*R,2*R**,5*R**)-4-methyl-1,2,5,6-tetrahydro-[1,1'-biphenyl]-2,5-dicarboxylate, 8q**

7q(95 mg, 0.39 mmol) was reacted according to the general procedure for 2 h. **8q** (53 mg, 46%) was isolated by column chromatography (EA/Hexs = 1:3) as a colorless oil. Spectral data of **8q**: ^1H NMR (400 MHz, $CDCl_3$, 24 °C, δ) 7.31 - 7.17 (m, 5H), 5.68 - 5.64 (m, 1H), 3.74 (s, 3H), 3.57 (s, 3H), 3.44 - 3.30 (m, 2H), 3.05 - 3.00 (t, J = 2.8 Hz, 1H), 2.21 (td, J = 13.5, 2.9 Hz, 1H), 1.99 (ddd, J = 13.5, 11.9, 6.0 Hz, 1H), 1.84 (s, 3H). $^{13}C\{^1H\}$ NMR (100 MHz, $CDCl_3$) 173.7 (C), 173.5 (C), 143.7 (C), 133.1 (C), 128.5 (CH), 127.3 (CH), 126.6 (CH), 122.6 (CH), 52.0 (CH_3), 51.9 (CH_3), 49.3 (CH), 45.0 (CH), 38.3 (CH), 32.7 (CH_2), 22.9 (CH). HRMS (ESI-TOF) (m/z): $[M+H]^+$ calcd for $C_{17}H_{21}O_4$, 289.1434; Found, 289.1437. IR (neat, cm-^{1}) : 2952, 1737, 1441, 1357, 1167, 771.

dimethyl (1*R,2*S**,5*R**)-3-methyl-1,2,5,6-tetrahydro-[1,1'-biphenyl]-2,5-**

dicarboxylate, 8r

7r(102 mg, 0.42 mmol) was reacted according to the general procedure for 2 h. **8r** (75 mg, 62%) was isolated by column chromatography (EA/Hexs = 1:5) as a colorless oil. Spectral data of **8r**: ^{1}H NMR (400 MHz, $CDCl_3$, 24 °C, δ) 7.32 - 7.15 (m, 5H), 5.79 (s, 1H), 3.71 (s, 3H), 3.57 (s, 3H), 3.40 - 3.32 (m, 1H), 3.22 (d, *J* = 8.0 Hz, 1H), 3.07 (s, 1H), 2.30 (td, *J* = 13.5, 3.5 Hz, 1H), 1.96 - 1.86 (m, 1H), 1.78 (s, 3H). $^{13}C\{^1H\}$ NMR (100 MHz, $CDCl_3$) 173.7 (C), 173.6 (C), 143.2 (C), 133.0 (C), 128.5 (CH), 127.0 (CH), 126.7 (CH), 121.8 (CH), 52.4 (CH), 51.9 (CH_3), 51.7 (CH_3), 40.6 (CH), 39.9 (CH), 30.0 (CH_2), 21.9 (CH_3). HRMS (ESI-TOF) (m/z): $[M+Na]^+$ calcd for $C_{17}H_{20}NaO_4$, 311.1254; Found, 311.1245. IR (neat, cm^{-1}) : 2952, 1740, 1448, 1375, 1120.

General procedure for the Schmidt reaction for the synthesis of 9, using 9a as an example.

To a solution of **5a** (50 mg, 0.12 mmol, 1 eq) in 0.73 mL of anhydrous DCM was added 3-azidopropan-1-ol(23 µL, 0.24 mmol, 2 eq). The reaction was then cooled to 0°C and $BF_3{\cdot}Et_2O$(61.0 µL, 0.494 mmol, 4.2 eq) was added dropwise. The reaction was then stirred at 0°C for 30min and then rt temperature for 12h. 10 mL of saturated $NaHCO_3$ (aq) was added to the reaction mixture and then extracted with CH_2Cl_2. The organic phase was collected and dried with anhydrous $MgSO_4$ and solvent was removed using rotavap. **9a/9a'** (9 mg, 17%, 1:1) was obtained as inseparable mixture by column chromatography (EA/Hexs = 1:1.5) as a white solid.

9a/9a'

Spectral data of **9a/9a' mixture**: ^{1}H NMR (400 MHz, $CDCl_3$, 24 °C, δ) : 7.36-

7.52 (m, 3H), 7.22-7.30 (m, 2H), 6.59 (d, J = 8.0 Hz, 1H), 5.67 (dd, J = 8.2, 4.0 Hz 1H), 4.77 (dddd, J = 11.5, 10.5, 9.7, 2.0 Hz, 1H), 4.31 (ddd, J = 44.0, 9.1, 1.4. Hz, 1H), 3.97-4.23 (m, 4H), 3.95 (ddd, J = 4.1, 4.0, 1.2 Hz, 1H), 3.84 (dd, J = 8.2, 0.9 Hz, 1H), 3.00-3.14 (m, 1H), 1.94-2.10 (m, 1H), 1.77-1.93 (m, 1H). $^{13}C\{^1H\}$ NMR (100 MHz, $CDCl_3$): 176.7 (C), 176.2 (C), 168.8 (C), 168.1 (C), 131.6 (1C), 129.9 (CH), 129.2 (CH), 129.0 (CH), 127.9 (CH), 126.3 (CH), 122.8 (1C), 120.3 (C), 66.9 (CH), 66.7 (CH), 52.6 (C), 51.8 (CH), 45.3 (CH), 44.5 (CH), 44.1 (C), 43.3 (C), 41.8 (CH), 41.3 (C), 39.7 (CH_2). HRMS (ESI-TOF) (m/z): $[M+Na]^+$ calcd for $C_{19}H_{17}N_2O_5NaBr$, 455.0213, 457.0193; Found**,** 455.0212, 457.0192. **IR** (neat, cm^{-1}) : 3418, 2954, 1713, 1667, 1524, 1457, 1388, 1267, 1191, 849, 734, 697. mp 267.3-274.6 °C.

(3a*R,4*R**,12*S**,12a*R**)-2-Phenyl-3a,4,7,8,9,10,12,12a-octahydro-1H-4,12-ethenopyrrolo[3,4-h][1]oxa[5]azacycloundecine-1,3,5,11(2*H*)-tetraone, 9b**

5b(66.4 mg, 0.200 mmol) was reacted according to the general procedure. **9b** (60 mg, 84%) was isolated by column chromatography (EA/Hexs = 3:1) as a white solid. Spectral data of **9b**: ^{1}H NMR (400 MHz, $CDCl_3$, 24 °C, δ) : 7.36-7.50 (m, 3H), 7.24 (t, J = 4.4 Hz, 2H), 6.40 (dd, J = 7.9, 7.7 Hz, 1H), 6.31 (dd, J = 7.5, 7.4 Hz, 1H), 5.67 (d, J = 8.3 Hz, 1H), 4.75 (ddd, J = 10.1, 6.0, 1.8 Hz,1H), 4.27 (dd, J = 8.1, 1.1 Hz, 1H), 4.17 (ddd, J = 10.2, 7.2, 2.6 Hz, 1H), 4.01 (dd, J = 9.2, 1.1 Hz, 1H), 3.90-4.00 (m, 2H), 3.79 (d, J = 7.4 Hz, 1H),

3.03 (dt, J = 8.6, 1.0 Hz, 1H), 1.96-2.08 (m, 1H), 1.73-1.87 (m, 1H). $^{13}C\{^1H\}$ NMR (100 MHz, $CDCl_3$) : 177.8 (C), 177.3 (C), 170.0 (C), 169.9 (C), 131.7 (C), 131.1 (CH), 129.2 (CH), 128.8 (CH), 128.3 (CH), 126.3 (CH), 66.1 (CH_2), 42.8 (C), 42.5 (C), 42.1 (C), 41.9 (C), 39.7 (CH_2), 29.7 (C), 26.1 (CH_2), 15.3 (C). HRMS (ESI-TOF) (m/z): $[M+Na]^+$ calcd for $C_{19}H_{18}N_2O_5Na$, 377.1108 Found**,** 377.1104. IR (neat, cm^{-1}) : 3416, 3053, 2948, 1780, 1665, 1524, 1188, 1040, 995, 767. mp 236.4 °C (decomposed)

(3a*R,4*R**,12*R**,12a*S**)-13-methyl-2-phenyl-3a,4,7,8,9,10,12,12a-octahydro-1H-4,12-ethenopyrrolo[3,4-h][1]oxa[5]azacycloundecine-1,3,5,11(2*H*)-tetraone, 9c(9g'); (3a*S**,4*R**,12*R**,12a*S**)-14-methyl-2-phenyl-3a,4,7,8,9,10,12,12a-octahydro-1H-4,12-ethenopyrrolo[3,4-h][1]oxa[5]azacycloundecine-1,3,5,11(2*H*)-tetraone, 9c'(9g)**

5c(68.3 mg, 0.200 mmol) was reacted according to the general procedure. **9c/9c'** (64 mg, 86%, 2:1) was obtained by column chromatography (EA/Hexs = 1:2) as an inseparable mixture. Crystal for X-ray crystallography was recrystallized from gas-diffusion in ether/ CH_2Cl_2. (CCDC 2393498)

larger scale: **5c**(681.6 mg,1.99 mmol) was reacted according to the general procedure. **9c/9c'** (337mg, 46%, 2:1) was obtained by column chromatography (EA/Hexs = 1:2) as an inseparable mixture.

Spectral data of **9c/9c'**: ^1H NMR (400 MHz, $CDCl_3$, 24 °C, δ) : 7.35-7.51 (m, 5H), 7.23 (q, J = 2.8 Hz, 3H), 6.06 (d, J = 8.0 Hz, 0.5H), 5.93 (d, J = 7.6 Hz, 1H), 5.69 (q, J = 8.8 Hz, 1.5H), 4.77-4.86 (m, 1H), 4.70-4.77 (m, 0.5H), 4.22

(ddd, *J* = 8.8, 5.1, 1.0 Hz, 2H), 4.09-4.17 (m, 1H), 4.00 (dd, *J* = 9.1, 1.3 Hz, 1H), 3.86-3.99 (m, 2.5H), 3.78 (s, 1H), 3.70 (d, *J* = 7.4 Hz, 1H), 3.62 (s, 0.5H), 3.02 (d, *J* = 10.6 Hz, 1.5H), 2.03 (d, *J* = 1.4 Hz, 4H), 1.98 (d, *J* = 1.4 Hz, 2H), 1.70-1.85 (m, 2H), 1.63 (s, 3.5H). $^{13}C\{^1H\}$ NMR (100 MHz, $CDCl_3$) : 178.0 (C), 177.8 (C), 177.5 (C), 177.4 (C), 170.5 (C), 170.4 (C), 169.8 (C), 169.6 (C), 140.8 (C), 137.7 (C), 131.8 (C), 131.7 (C), 129.1 (CH), 128.8 (CH), 128.8 (2×CH), 126.2 (2×CH), 123.4 (CH), 120.7 (CH), 77.2 (C), 66.0 (CH_2), 65.9 (CH_2), 48.0 (CH), 46.8 (CH), 43.5 (CH), 43.1 (CH), 42.6 (CH), 42.4 (CH), 42.0 (CH), 41.4 (CH), 39.8 (CH_2), 39.7 (CH_2), 29.7 (C), 26.1 (CH_2), 25.7 (CH), 24.7 (CH), 24.4 (CH). HRMS (ESI-TOF) (m/z): $[M+Na]^+$ calcd for $C_{20}H_{20}N_2O_5Na$, 391.1264; Found**,** 391.1257. IR (neat, cm^{-1}) : 3474, 2942, 1955, 1778, 1597, 1387, 1191, 916, 789, 735. mp 172.3-190.5 °C

9e/9e'

5e(74 mg, 0.20 mmol) was reacted according to the general procedure. **9e/9e'** (55 mg, 70%, 2:1) was isolated by column chromatography (EA/Hexs = 1:1) as an inseparable mixture. Spectral data of **9e/9e'**: ^{1}H NMR (400 MHz, $CDCl_3$, 24 °C, δ) : 7.34-7.49 (m, 3H), 7.14-7.23 (m, 2H), 6.01 (dd, *J* = 49.2, 7.9 Hz, 1H), 5.59-5.74 (m, 2H), 5.10-5.21 (m, 2H), 4.70-4.81 (m, 1H), 4.22 (ddd, *J* = 9.1, 3.3, 1.3 Hz, 1H), 4.09-4.19 (m, 1H), 4.01 (dd, *J* = 9.1, 1.2 Hz, 1H), 3.85-3.99 (m, 1H), 3.80 (d, *J* = 0.8 Hz, 1H), 3.70 (t, *J* = 21.1 Hz, 1H), 3.07 (ddd, *J*

= 15.4, 6.6, 1.1 Hz, 3H), 1.93-2.08 (m, 1H), 1.67-1.87 (m, 1H). $^{13}C\{^1H\}$ NMR (100 MHz, $CDCl_3$) : 178.0 (C), 177.1 (C), 170.4 (C), 169.8 (C), 143.0 (CH), 133.0 (CH_2), 129.2 (CH_2), 128.8 (CH_2), 126.2 (CH_2), 124.0 (CH_2), 121.3 (CH_2), 119.0 (CH), 66.1 (CH), 45.1 (CH_2), 43.4 (CH_2), 42.5 (CH_2), 42.2 (CH_2), 42.0 (CH_2), 42.0 (CH), 39.6 (CH), 26.2 (CH). HRMS (ESI-TOF) (m/z): $[M+Na]^+$ calcd for $C_{22}H_{22}N_2O_5Na$, 417.1421; Found, 417.1412. IR (neat, cm^{-1}): 3390, 2924, 2853, 1711, 1665, 1498, 1265, 1193, 1039, 738, 696. mp 196.2-208.6 °C

9s/9s'

5s(74 mg, 0.20 mmol) was reacted according to the general procedure. **9s/9s'** (55 mg, 35%, 2:1) was isolated by column chromatography (EA/Hexs = 1:1) as an inseparable mixture. Spectral data of **9s/9s'**: ^1H NMR (400 MHz, $CDCl_3$, 24 °C, δ) : 7.41-7.48 (m, 3H), 7.34-7.41 (m, 2H), 7.19-7.24 (m, 3H), 7.16 (d, *J* = 7.8 Hz, 1H), 5.98 (t, *J* = 4.4 Hz, 1H), 5.37 (d, *J* = 8.2 Hz, 0.5H), 4.76-4.84 (m, 0.5H), 4.61-4.70 (m, 1H), 4.58 (d, *J* = 1.0 Hz, 0.5H), 4.34 (s, 2H), 4.32 (t, *J* = 1.8 Hz, 1H), 4.15 (q, *J* = 3.5 Hz, 0.5H), 3.95-4.08 (m, 6H), 3.86 (s, 3H), 3.83 (s, 1.5H), 2.92-3.06 (m, 1.5H), 1.98 (d, *J* = 15.6 Hz, 0.5H), 1.82 (q, *J* = 4.0 Hz, 2H), 1.69-1.78 (m, 0.5H). $^{13}C\{^1H\}$ NMR (100 MHz, $CDCl_3$): 177.1 (2×C), 176.9 (C), 176.5 (C), 169.7 (C), 169.2 (C), 168.5 (C), 168.2 (C), 166.9 (C), 164.5 (C), 137.6 (CH), 136.3 (CH), 133.9 (C), 132.3 (C), 131.5 (2×C),

129.1 (2×CH), 128.8 (2×CH), 126.2 (CH), 126.2 (CH), 77.2 (CH_2), 67.1 (CH_2), 66.5 (CH_2), 53.0 (CH), 52.8 (CH), 43.6 (CH), 42.9 (CH), 42.8 (CH), 42.7 (CH), 42.3 (CH), 41.7 (CH), 41.7 (CH), 41.4 (CH), 39.7 (CH), 39.1 (CH), 28.1 (CH_2), 26.4 (CH_2). HRMS (ESI-TOF) (m/z): $[M+Na]^+$ calcd for $C_{21}H_{20}N_2O_7Na$, 435.1163 Found**,** 435.1153. IR (neat, cm^{-1}): 3398, 2954, 2850, 1715, 1497, 1385, 1190, 720, 695. mp 265.7-269.2 ℃

(3a*R,4*S**,12*S**,12a*R**)-14-methyl-2-phenyl-3a,4,7,8,9,10,12,12a-octahydro-1H-4,12-ethenopyrrolo[3,4-h][1]oxa[5]azacycloundecine-1,3,5,11(2*H*)-tetraone, 9g(9c'), (3a*S**,4*S**,12*S**,12a*R**)-13-methyl-2-phenyl-3a,4,7,8,9,10,12,12a-octahydro-1H-4,12-ethenopyrrolo[3,4-h][1]oxa[5]azacycloundecine-1,3,5,11(2*H*)-tetraone, 9g'(9c)**

5g(48 mg, 0.14 mmol) was reacted according to the general procedure. **9g/9g'** (38 mg, 73%, 1:3) was isolated by column chromatography (EA/Hexs = 2:1) as an inseparable mixture.

methyl-(3a*R,4*S**,12*S**,12a*R**)-1,3,5,11-tetraoxo-2-phenyl-2,3,3a,4,5,7,8,9,10,11,12,12a-dodecahydro-1*H*-4,12-ethenopyrrolo[3,4-h][1]oxa[5]azacycloundecine-14-carboxylate, 9j, methyl-(3a*S**,4*S**,12*S**,12a*R**)-1,3,5,11-tetraoxo-2-phenyl-2,3,3a,4,5,7,8,9,10,11,12,12a-dodecahydro-1*H*-4,12-ethenopyrrolo[3,4-h][1]oxa[5]azacycloundecine-13-carboxylate, 9j'**

5j(78 mg, 0.20 mmol) was reacted according to the general procedure. **9j/9j'** (37 mg, 43%, 1:2) was isolated by column chromatography (EA/Hexs = 1:1) as an inseparable mixture. Spectral data of **9j/9j'**: ^1H NMR (400 MHz, D_6-

DMSO, 24 °C, δ) : 7.55 (t, *J* = 4.0 Hz, 0.3H), 7.47 (q, *J* = 6.3 Hz, 3H), 7.41 (t, *J* = 7.3 Hz, 1.5H), 7.26 (d, *J* = 8.1 Hz, 1H), 7.12-7.18 (m,2.5H), 7.09 (d, *J* = 6.6 Hz, 1H), 4.36-4.39 (m, 1H), 4.22 (dd, *J* = 9.0, 1.2 Hz, 1H), 4.11 (d, *J* = 10.1 Hz, 0.6H), 4.03 (t, *J* = 4.5 Hz, 3H), 3.80-3.95 (m, 2H), 3.75 (d, *J* = 5.6 Hz, 4H), 3.68 (q, *J* = 10.4 Hz, 1H), 2.86 (d, *J* = 7.9 Hz, 1H), 1.61-1.90 (m, 2.5H). $^{13}C\{^1H\}$ NMR (100 MHz, D_6-DMSO): 177.9 (C), 177.8 (C), 177.4 (C), 177.3 (C), 169.4 (C), 169.0 (C), 169.0 (C), 168.2 (C), 164.9 (C), 164.9 (C), 139.0 (CH), 138.5 (CH), 132.0 (C), 131.3 (C), 129.8 (CH), 129.0 (CH), 128.6 (CH), 126.7 (CH), 126.6 (CH), 66.9 (CH_2), 52.4 (CH), 52.4 (CH), 43.6 (CH), 42.7 (CH), 42.5 (CH), 42.4 (CH), 41.6 (CH), 41.1 (CH), 40.9 (CH), 39.0 (CH_2), 38.6 (C), 25.4 (CH_2). HRMS (ESI-TOF) (m/z): $[M+Na]^+$ calcd for $C_{21}H_{20}N_2O_7Na$, 435.1163 Found**,** 435.1156. **IR** (neat, cm^{-1}) : 3396, 2955, 2850, 1715, 1667, 1535, 1497, 1253, 1192, 1082, 757, 696. mp 265.7-269.2 ℃

(3a*R,4*R**,12*S**,12a*S**)-12-methyl-2-phenyl-3a,4,7,8,9,10,12,12a-octahydro-1H-4,12-ethenopyrrolo[3,4-h][1]oxa[5]azacycloundecine-1,3,5,11(2*H*)-tetraone, 9k**

5k(67 mg, 0.20 mmol) was reacted according to the general procedure. **9k** (53 mg, 73%) was isolated by column chromatography (EA/Hexs = 1:1) as a white solid. Spectral data of **9k**: ^1H NMR (400 MHz, $CDCl_3$, 24 °C, δ) : 7.48 (t, *J* = 7.5 Hz, 2H), 7.41 (t, *J* = 7.3 Hz, 1H), 7.17 (d, *J* = 7.5 Hz, 2H), 7.11 (d, *J* = 7.1 Hz, 1H), 6.25 (t, *J* = 8.6 Hz, 1H), 5.97 (d, *J* = 9.4 Hz, 1H), 4.52 (t, *J* = 11.4

Hz, 1H), 4.07 (d, *J* = 8.6 Hz, 1H), 3.92 (q, *J* = 3.9 Hz, 1H), 3.78 (d, *J* = 8.7 Hz, 1H), 3.60-3.75 (m, 1H), 3.53 (d, *J* = 7.4 Hz, 1H), 2.86 (d, *J* = 13.6 Hz, 1H), 1.65-1.87 (m, 2H), 1.50 (s, 3H). $^{13}C\{^1H\}$ NMR (100 MHz, D_6-DMSO): 178.4 (C), 175.7 (C), 173.5 (C), 169.5 (C), 135.0 (CH), 132.3 (C), 128.9 (CH), 128.5 (CH), 127.4 (CH), 127.0 (CH), 66.3 (CH_2), 46.3 (CH), 44.7 (C), 42.9 (CH), 42.1 (CH), 38.8 (CH_2), 26.2 (CH_2), 24.2 (CH_3). HRMS (ESI-TOF) (m/z): $[M+Na]^+$ calcd for $C_{20}H_{20}N_2O_5Na$, 391.1264 Found**,** 391.1256. IR (neat, cm^{-1}) : 3446, 2917, 2850, 1772, 1652, 1541, 1385, 1083, 669. mp 276.0-281.0 °C

(3a*R,4*R**,12R*,12a*S**)-12-methoxy-2-phenyl-3a,4,7,8,9,10,12,12a-octahydro-1*H*-4,12-ethenopyrrolo[3,4-h][1]oxa[5]azacycloundecine-1,3,5,11 (2*H*) -tetraone 9l**

5l(73 mg, 0.20 mmol) was reacted according to the general procedure. **9l** (48 mg, 62%) was isolated by column chromatography (EA/Hexs = 2:1) as a white solid. Spectral data of **9l**: ^1H NMR (400 MHz, $CDCl_3$, 24 °C, δ) : 7.34-7.48 (m, 3H), 7.23 (t, *J* = 4.3 Hz, 2H), 6.14 (d, *J* = 6.2 Hz, 2H), 5.70 (d, *J* = 7.7 Hz, 1H), 4.71 (ddd, *J* = 11.3, 6.9, 1.3 Hz, 1H), 4.53 (d, *J* = 8.8 Hz, 1H), 4.00-4.11 (m, 1H), 3.91-4.00 (m, 3H), 3.48 (s, 3H), 3.05 (dd, *J* = 12.2, 3.0 Hz, 1H), 1.76-1.96 (m, 2H). $^{13}C\{^1H\}$ NMR (100 MHz, $CDCl_3$) : 177.1 (C), 173.8 (C), 169.7 (C), 169.5 (C), 133.5 (CH), 131.7 (C), 129.1 (CH), 128.7 (CH), 127.9 (CH), 126.5 (CH), 80.9 (C), 77.2 (C), 66.5 (CH_2), 53.4 (CH), 43.3 (CH), 42.3 (CH),

41.9 (CH), 39.1 (CH_2), 28.4 (CH_2), 15.3 (C). HRMS (ESI-TOF) (m/z): $[M+Na]^+$ calcd for $C_{20}H_{20}N_2O_6Na$, 407.1214 Found**,** 407.1207. IR (neat, cm^{-1}) : 3380, 2934, 1667, 1497, 1384, 1269, 1133, 823, 699. mp 216.7-217.6 °C

(1*S,9*R**,9a*S**,12a*R**)-13-methyl-4,5,6,7,9,9a,10,12a-octahydro-2H-1,9-ethenocyclopenta[h][1]oxa[5]azacycloundecine-2,8(1*H*)-dione, 9n**

5n(55 mg, 0.24 mmol) was reacted according to the general procedure. **9n** (36 mg, 56%) was isolated by column chromatography (EA/Hexs = 1:1) as a light yellow oil. Spectral data of **9n**: ^1H NMR (400 MHz, $CDCl_3$, 24 °C, δ) : 5.80 (d, *J* = 7.0 Hz, 1H), 5.68 (dd, *J* = 5.3, 2.5 Hz, 1H), 5.42 (dd, *J* = 5.4, 2.4 Hz, 1H), 3.84 (t, *J* = 6.2 Hz, 2H), 3.61 (d, *J* = 7.6 Hz, 1H), 3.54 (s, 1H), 3.43 (d, *J* = 5.8 Hz, 3H), 3.10 (dddd, *J* = 10.2, 9.7, 4.8, 2.0 Hz, 1H), 2.73 (dd, *J* = 17.3, 10.0 Hz, 2H), 1.86-1.98 (m, 4H), 1.65-1.76 (m, 3H). $^{13}C\{^1H\}$ NMR (100 MHz, $CDCl_3$): 174.5 (C), 173.9 (C), 137.5 (C), 133.1 (CH_2), 131.3 (CH_2), 121.2 (CH_2), 58.9 (CH_3), 57.8 (CH_2), 50.6 (CH_2), 48.5 (CH_2), 39.9 (CH), 37.7 (CH_2), 37.6 (CH), 30.7 (CH), 23.5 (CH_2). HRMS (ESI-TOF) (m/z): $[M+Na]^+$ calcd for $C_{15}H_{19}NO_3Na$, 284.1257 Found**,** 284.1250. IR (neat, cm^{-1}) : 3469, 2955, 2853, 1709, 1660, 1444, 1333, 1275, 1174, 699.

(1*R,9*S**,9a*S**,12a*R**)-1-methyl-4,5,6,7,9,9a,10,12a-octahydro-2H-1,9-ethenocyclopenta[h][1]oxa[5]azacycloundecine-2,8(1*H*)-dione, 9v**

5v(49 mg, 0.21 mmol) was reacted according to the general procedure. **9v** (28

mg, 64%) was isolated by column chromatography (EA/Hexs = 1:2) as a light-yellow oil. Spectral data of **9v**: ^{1}H NMR (400 MHz, $CDCl_3$, 24 °C, δ) : 6.19 (t, *J* = 8.0 Hz, 1H), 5.84 (d, *J* = 8.6 Hz, 1H), 5.75 (ddd, *J* = 6.0, 4.2, 2.0 Hz, 1H), 5.57 (ddd, *J* = 5.9, 4.2, 1.9 Hz, 1H), 3.89 (t, *J* = 6.2 Hz, 2H), 3.72 (d, *J* = 7.4 Hz, 1H), 3.42 (t, *J* = 5.7 Hz, 2H), 3.09-3.25 (m, 2H), 2.73 (dddd, *J* = 17.3, 9.7, 4.2, 2.2 Hz, 1H), 2.05 (md, *J* = 17.3, 2.2Hz, 1H), 1.66-1.76 (m, 2H), 1.45 (s, 3H). ^{13}C{^{1}H} NMR (100 MHz, $CDCl_3$): 175.7 (C), 174.4 (C), 136.6 (CH), 133.8 (CH), 129.5 (CH), 127.0 (CH), 58.9 (CH_2), 54.3 (CH), 51.6 (CH), 40.6 (CH_2), 38.5 (CH), 38.3 (CH_2), 30.7 (CH_2), 22.4 (CH_3). HRMS (ESI-TOF) (m/z): $[M+Na]^+$ calcd for $C_{15}H_{19}NO_3Na$, 284.1257 Found**,** 284.1250. IR (neat, cm^{-1}) : 3414, 2924, 2853, 1705, 1660, 1442, 1260, 1181, 1096, 747.

(1*R,9*R**,12*R**)-10-methyl-12-phenyl-3-oxa-7-azabicyclo[7.2.2]tridec-10-ene-2,8-dione, 9q**

5q(44.1 mg, 0.162 mmol) was reacted according to the general procedure. **9q** (25 mg, 50%) was isolated by column chromatography (EA/Hexs = 1:2) as a light-yellow oil. Spectral data of **9q**: ^{1}H NMR (400 MHz, CDCl3, 24 °C, δ) : 7.21-7.35 (m, 3H), 7.17 (t, J = 4.2 Hz, 2H), 6.57 (t, J = 8.0 Hz, 1H), 6.38 (t, J = 8.1 Hz, 1H), 3.85-3.97 (m, 3H), 3.83 (d, J = 7.4 Hz, 1H), 3.60 (dd, J = 10.1, 6.9 Hz, 1H), 3.49 (t, J = 5.6 Hz, 2H), 2.73 (ddd, J = 14.1, 10.1, 2.2 Hz, 1H), 1.99 (qd, J = 14.1, 3.9 Hz, 1H), 1.72-1.83 (m, 2H). ^{13}C{^{1}H} NMR (100 MHz,

$CDCl_3$): 174.2 (2×C), 144.6 (C), 141.4 (C), 128.8 (CH), 128.7 (CH), 127.3 (CH), 127.3 (CH), 127.0 (CH), 127.0 (CH), 124.1 (CH), 120.5 (CH), 59.0 (CH_2), 54.9 (CH), 53.3 (CH), 52.7 (CH), 40.9 (CH), 37.9 (CH_2), 37.8 (CH_2), 32.9 (CH_2), 30.7 (CH_2), 21.6 (CH), 21.4 (CH). HRMS (ESI-TOF) (m/z): $[M+Na]^+$ calcd for $C_{18}H_{21}NO_3Na$, 322.1414 Found**,** 322.1406. IR (neat, cm^{-1}) : 3455, 2923, 2853, 1709, 1660, 1443, 1333, 1063, 761.

methyl (1*R,9*R**,12*R**)-2,8-dioxo-12-phenyl-3-oxa-7-azabicyclo[7.2.2]tridec-10-ene-10-carboxylate, 9w**

5w(65 mg, 0.21 mmol) was reacted according to the general procedure. **9w** (43 mg, 60%) was isolated by column chromatography (EA/Hexs = 1:1) as a light-yellow oil. Spectral data of **9w**: ^1H NMR (400 MHz, $CDCl_3$, 24 °C, δ) : 7.25-7.33 (m, 3H), 7.14-7.23 (m, 3H), 5.80 (d, *J* = 7.7 Hz, 1H), 4.45-4.53 (m, 1H), 4.17 (ddd, *J* = 10.6, 6.9, 3.2 Hz, 1H), 3.98-4.10 (m, 1H), 3.88 (s, 3H), 3.74-3.81 (m, 1H), 3.73 (dd, *J* = 3.8, 3.4 Hz, 1H), 3.57 (dd, *J* = 3.3, 7.0 Hz, 1H), 2.90-2.98 (m, 1H), 2.85 (ddd, *J* = 13.3, 7.6, 2.7 Hz, 1H), 1.87-1.98 (m, 1H), 1.69-1.79 (m, 1H), 1.60-1.70 (m, 1H). $^{13}C\{^1H\}$ NMR (100 MHz, $CDCl_3$): 171.9 (C), 170.8 (C), 167.1 (C), 146.3 (C), 139.4 (CH), 135.1 (C), 128.8 (CH), 127.2 (CH), 126.7 (CH), 66.6 (CH_2), 52.6 (CH_3), 49.7 (CH), 41.3 (CH), 41.0 (CH), 39.1 (CH_2), 34.7 (CH_2), 27.6 (CH_2). HRMS (ESI-TOF) (m/z): $[M+Na]^+$ calcd for $C_{19}H_{21}NO_5Na$, 366.1312 Found**,** 366.1302. IR (neat, cm^{-1}) : 3409,

2925, 2853, 1725, 1663, 1529, 1440, 1377, 1210, 1068, 764, 702.

(1*R,9*R**,12*S**)-1-methyl-12-phenyl-3-oxa-7-azabicyclo[7.2.2]tridec-10-ene-2,8-dione 1-methyl-12-phenyl-3-oxa-7-azabicyclo[7.2.2]tridec-10-ene-2,8-dione, 9x**

5x(55 mg, 0.20 mmol) was reacted according to the general procedure. **9x** (37 mg, 61%) was isolated by column chromatography (EA/Hexs = 1:1) as a light-yellow oil. Spectral data of **9x**: ^{1}H NMR (400 MHz, $CDCl_3$, 24 °C, δ) of : 7.21-7.32 (m, 3H), 7.13 (t, *J* = 4.0 Hz, 2H), 6.56 (t, *J* = 8.1 Hz, 1H), 5.95 (d, *J* = 8.5 Hz, 1H), 3.96 (t, *J* = 6.2 Hz, 2H), 3.87 (qd, *J* = 6.2, 2.1 Hz, 1H), 3.49 (t, *J* = 5.6 Hz, 2H), 3.25 (dd, *J* = 10.1, 6.6 Hz, 1H), 2.78 (ddd, *J* = 14.4, 10.2, 1.8 Hz, 2H), 2.13 (ddd, *J* = 14.4, 6.5, 5.3 Hz, 1H), 1.73-1.82 (m, 2H), 1.02 (s, 1H). $^{13}C\{^{1}H\}$ NMR (100 MHz, $CDCl_3$): 175.8 (C), 174.9 (C), 143.0 (C), 135.4 (CH), 130.3 (CH), 128.4 (CH), 127.1 (CH), 59.1 (CH_2), 51.3 (C), 46.4 (CH), 46.3 (CH), 38.5 (CH_2), 34.8 (CH_2), 30.7 (CH_2), 23.4 (CH). HRMS (ESI-TOF) (m/z): $[M+Na]^+$ calcd for $C_{18}H_{21}NO_3Na$, 322.1414 Found**,** 322.1406. IR (neat, cm^{-1}): 3377, 2920, 2851, 1724, 1381, 1114, 749.

(3aR*,4R*,11R*,11aS*)-12-methyl-2-phenyl-3a,4,8,9,11,11a-hexahydro 4,11-ethenopyrrolo[3,4-g][1,4]oxazecine-1,3,5,10 (2H,7H) -tetraone, 9y

5y(71 mg, 0.21 mmol) was reacted according to the general procedure. **9y** (34 mg, 47%) was isolated by column chromatography (EA/Hexs = 1:3) as a white

solid. Spectral data of **9y**: ^{1}H NMR (400 MHz, $CDCl_3$, 24 °C, δ) : 7.37-7.51 (m, 3H), 7.15-7.20 (m, 2H), 6.00 (td, *J* = 7.5, 1.7 Hz, 1H), 4.28 (dd, *J* = 7.4, 2.0 Hz, 1H), 4.21 (t, *J* = 1.9 Hz, 1H), 4.01 (t, *J* = 5.0 Hz, 2H), 3.70-3.77 (m, 3H), 3.67 (dd, *J* = 8.8, 1.9 Hz, 1H), 1.94 (d, *J* = 1.7 Hz, 3H). $^{13}C\{^{1}H\}$ NMR (100 MHz, $CDCl_3$): 174.5 (C), 174.3 (C), 171.4 (C), 170.6 (C), 139.4 (C), 131.3 (CH), 129.3 (CH), 129.2 (CH), 126.1 (CH), 120.7 (CH), 60.9 (CH_2), 52.8 (CH), 47.6 (CH), 43.7 (CH_2), 42.1 (CH), 42.0 (CH), 22.2 (CH_3). HRMS (ESI-TOF) (m/z): $[M+Na]^+$ calcd for $C_{19}H_{18}N_2O_5Na$, 377.1108 Found**,** 377.1105. IR (neat, cm-^{1}) : 3489, 2957, 1715, 1666, 1435, 1382, 1150, 1047, 808, 692. mp 179.9-180.8 °C

^{1}H NMR of 2a

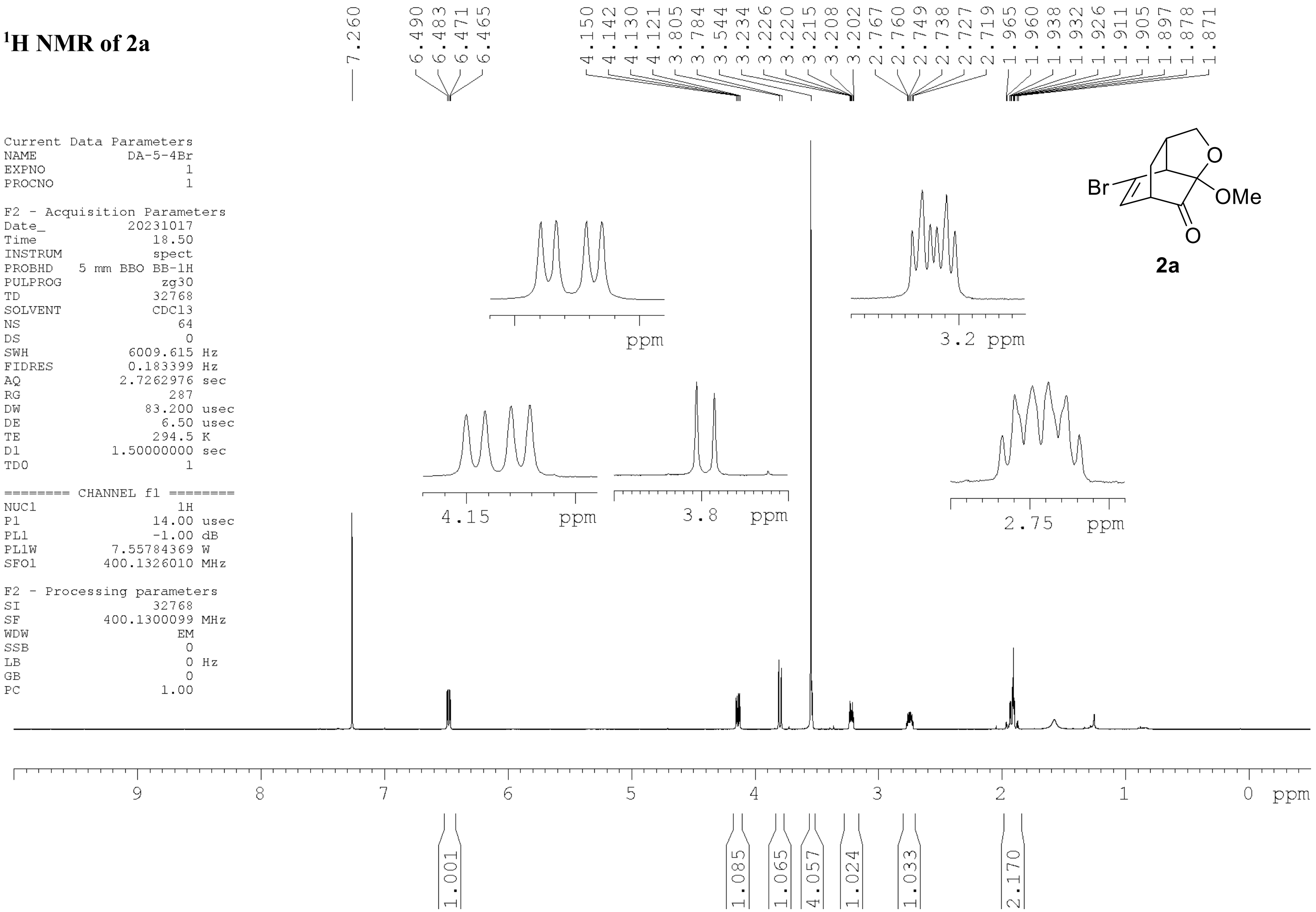

2a

^{1}H NMR of 2b

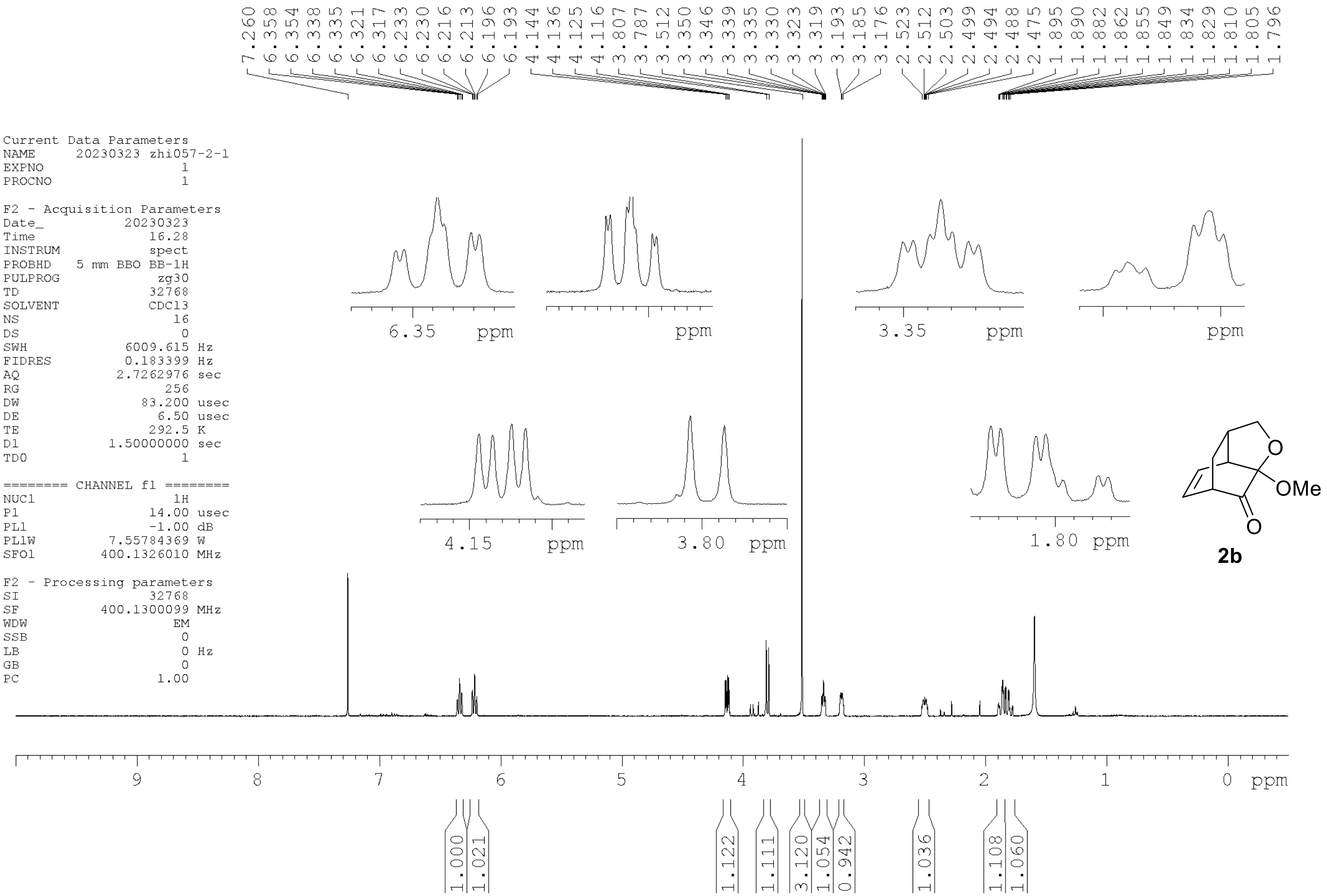

7.260
6.358
6.354
6.338
6.335
6.321
6.317
6.233
6.230
6.216
6.213
6.196
6.193
4.144
4.136
4.125
4.116
3.807
3.787
3.512
3.350
3.346
3.339
3.335
3.330
3.323
3.319
3.193
3.185
3.176
2.523
2.512
2.503
2.499
2.494
2.488
2.475
1.895
1.890
1.882
1.862
1.855
1.849
1.834
1.829
1.810
1.805
1.796
Current Data Parameters
NAME 20230323 zhi057-2-1
EXPNO 1
PROCNO 1
F2 - Acquisition Parameters
Date_ 20230323
Time 16.28
INSTRUM spect
PROBHD 5 mm BBO BB-1H
PULPROG zg30
TD 32768
SOLVENT CDCl3
NS 16
DS 0
SWH 6009.615 Hz
FIDRES 0.183399 Hz
AQ 2.7262976 sec
RG 256
DW 83.200 usec
DE 6.50 usec
TE 292.5 K
D1 1.50000000 sec
TD0 1
======== CHANNEL f1 ========
NUC1 1H
P1 14.00 usec
PL1 -1.00 dB
PL1W 7.55784369 W
SFO1 400.1326010 MHz
F2 - Processing parameters
SI 32768
SF 400.1300099 MHz
WDW EM
SSB 0
LB 0 Hz
GB 0
PC 1.00
6.35 ppm
ppm
3.35 ppm
ppm
4.15 ppm
3.80 ppm
1.80 ppm
O
OMe
O
2b
9
8
7
6
5
4
3
2
1
0 ppm
1.000
1.021
1.122
1.111
3.120
1.054
0.942
1.036
1.108
1.060

^{1}H NMR of 2c

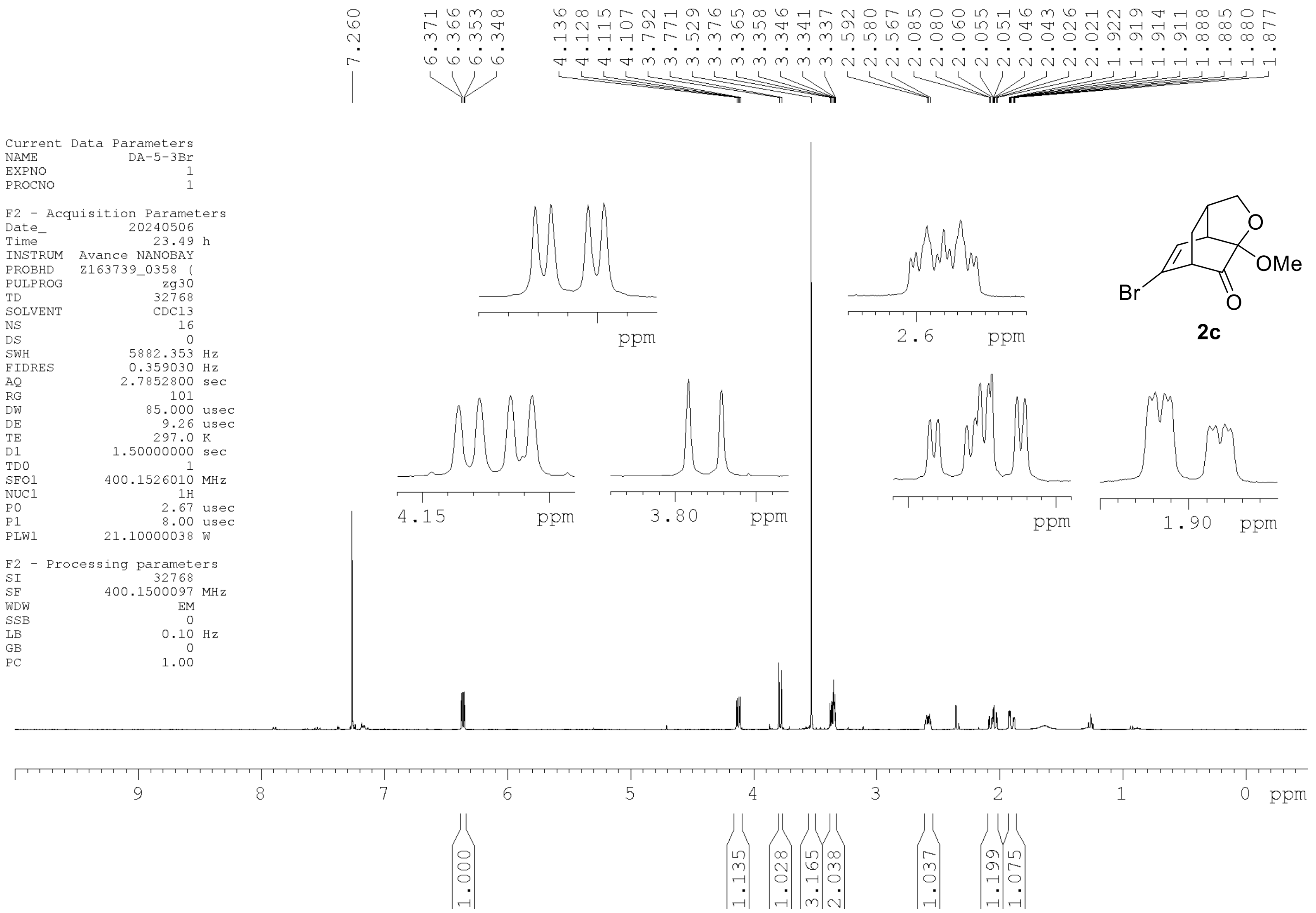
Current Data Parameters
NAME DA-5-3Br
EXPNO 1
PROCNO 1
F2 - Acquisition Parameters
Date_ 20240506
Time 23.49 h
INSTRUM Avance NANOBAY
PROBHD Z163739_0358 (
PULPROG zg30
TD 32768
SOLVENT CDCl3
NS 16
DS 0
SWH 5882.353 Hz
FIDRES 0.359030 Hz
AQ 2.7852800 sec
RG 101
DW 85.000 usec
DE 9.26 usec
TE 297.0 K
D1 1.50000000 sec
TD0 1
SFO1 400.1526010 MHz
NUC1 1H
P0 2.67 usec
P1 8.00 usec
PLW1 21.10000038 W
F2 - Processing parameters
SI 32768
SF 400.1500097 MHz
WDW EM
SSB 0
LB 0.10 Hz
GB 0
PC 1.00
7.260
6.371 6.366 6.353 6.348
4.136 4.128 4.115 4.107 3.792 3.771 3.529 3.376 3.365 3.358 3.346 3.341 3.337 2.592 2.580 2.567 2.085 2.080 2.060 2.055 2.051 2.046 2.043 2.026 2.021 1.922 1.919 1.914 1.911 1.888 1.885 1.880 1.877
OMe
Br
O
2c
1.000 1.135 1.028 3.165 2.038 1.037 1.199 1.075
ppm

^{1}H NMR of 2d

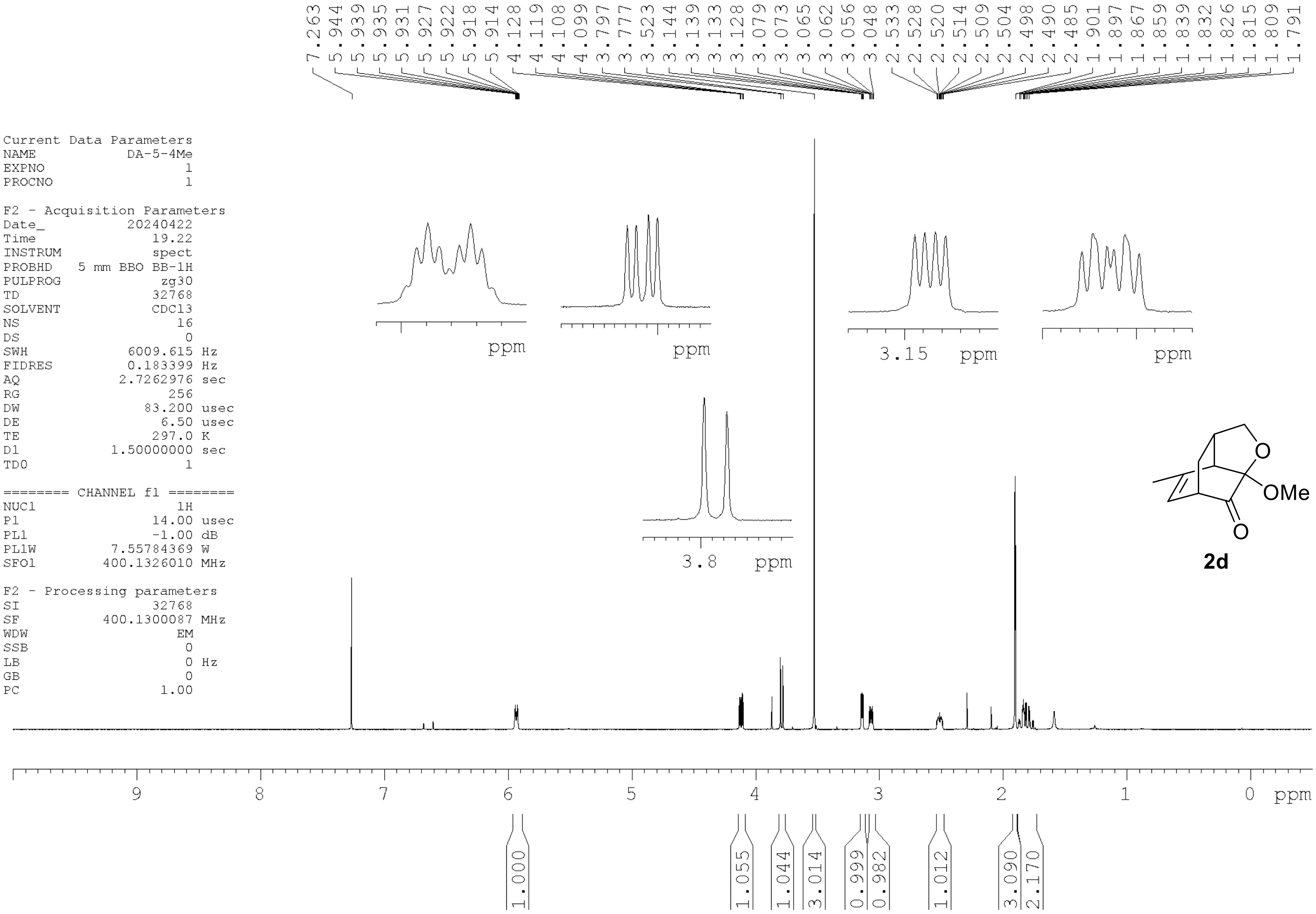

2d

^{1}H NMR of 2e

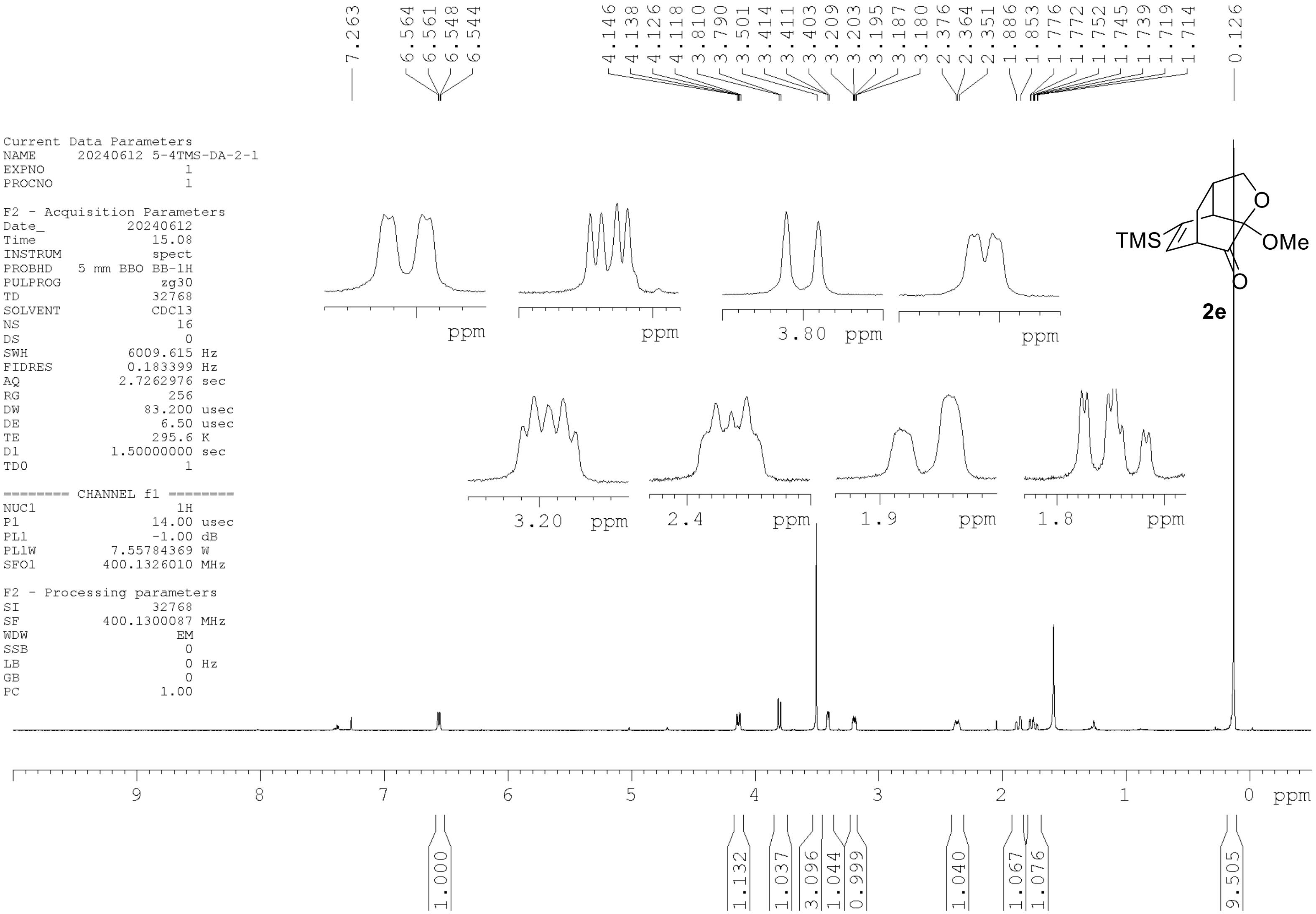


```
Current Data Parameters
NAME      20240612 5-4TMS-DA-2-1
EXPNO                  1
PROCNO                 1

F2 - Acquisition Parameters
Date_            20240612
Time                15.08
INSTRUM             spect
PROBHD   5 mm BBO BB-1H
PULPROG              zg30
TD                  32768
SOLVENT             CDCl3
NS                     16
DS                      0
SWH              6009.615 Hz
FIDRES           0.183399 Hz
AQ              2.7262976 sec
RG                    256
DW                 83.200 usec
DE                   6.50 usec
TE                  295.6 K
D1             1.50000000 sec
TD0                     1

======== CHANNEL f1 ========
NUC1                   1H
P1                  14.00 usec
PL1                 -1.00 dB
PL1W           7.55784369 W
SFO1         400.1326010 MHz

F2 - Processing parameters
SI                  32768
SF            400.1300087 MHz
WDW                    EM
SSB                     0
LB                      0 Hz
GB                      0
PC                   1.00
```

^{1}H NMR of 2f

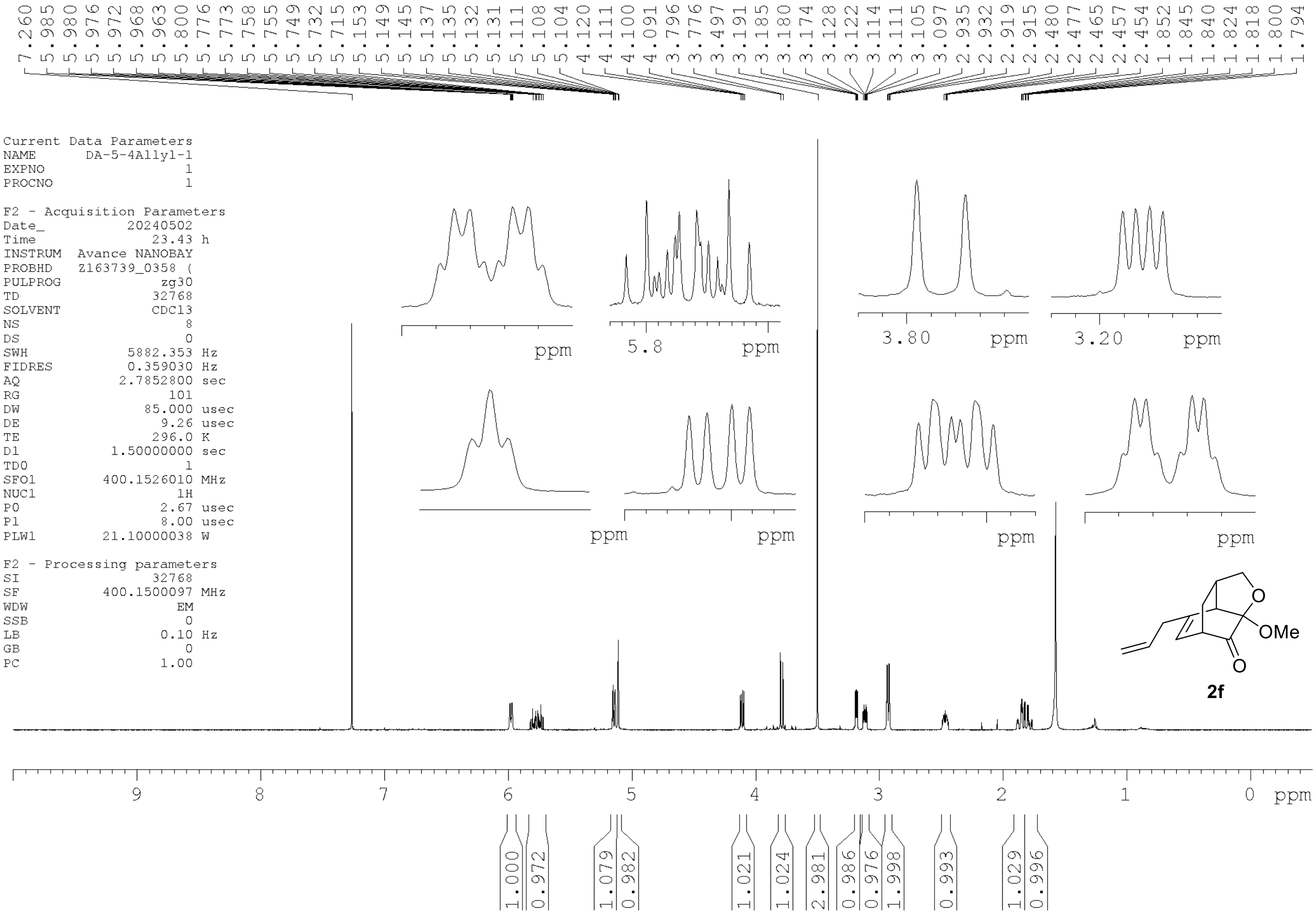
Current Data Parameters
NAME DA-5-4Allyl-1
EXPNO 1
PROCNO 1
F2 - Acquisition Parameters
Date_ 20240502
Time 23.43 h
INSTRUM Avance NANOBAY
PROBHD Z163739_0358 (
PULPROG zg30
TD 32768
SOLVENT CDCl3
NS 8
DS 0
SWH 5882.353 Hz
FIDRES 0.359030 Hz
AQ 2.7852800 sec
RG 101
DW 85.000 usec
DE 9.26 usec
TE 296.0 K
D1 1.50000000 sec
TD0 1
SFO1 400.1526010 MHz
NUC1 1H
P0 2.67 usec
P1 8.00 usec
PLW1 21.10000038 W
F2 - Processing parameters
SI 32768
SF 400.1500097 MHz
WDW EM
SSB 0
LB 0.10 Hz
GB 0
PC 1.00
7.260 5.985 5.980 5.976 5.972 5.968 5.963 5.800 5.776 5.773 5.758 5.755 5.749 5.732 5.715 5.153 5.149 5.145 5.137 5.135 5.132 5.131 5.111 5.108 5.104 4.120 4.111 4.100 4.091 3.796 3.776 3.497 3.191 3.185 3.180 3.174 3.128 3.122 3.114 3.111 3.105 3.097 2.935 2.932 2.919 2.915 2.480 2.477 2.465 2.457 2.454 1.852 1.845 1.840 1.824 1.818 1.800 1.794
1.000 0.972 1.079 0.982 1.021 1.024 2.981 0.986 0.976 1.998 0.993 1.029 0.996
OMe
O
O
2f
ppm

^{1}H NMR of 2g

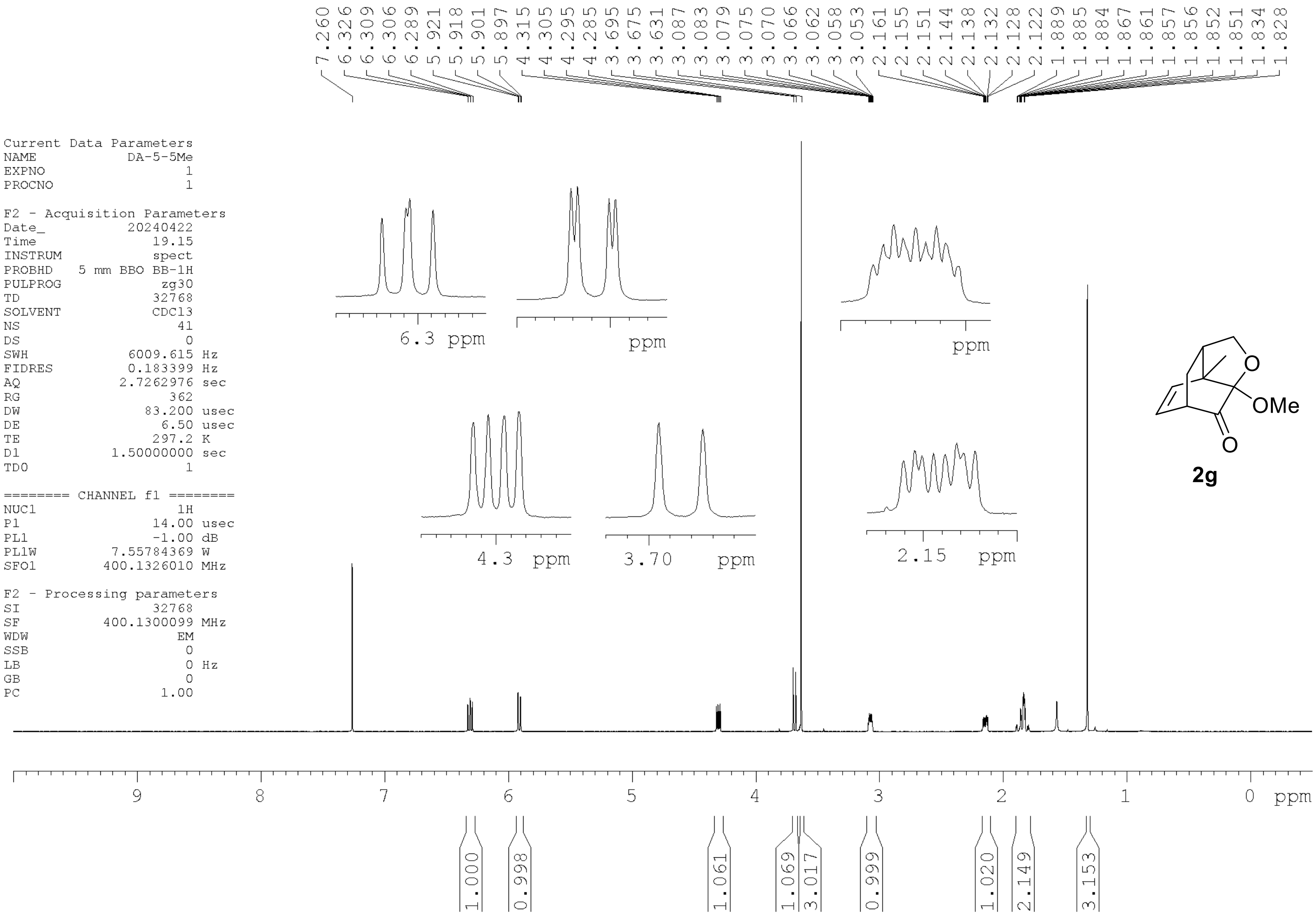

2g

^{1}H NMR of 2h

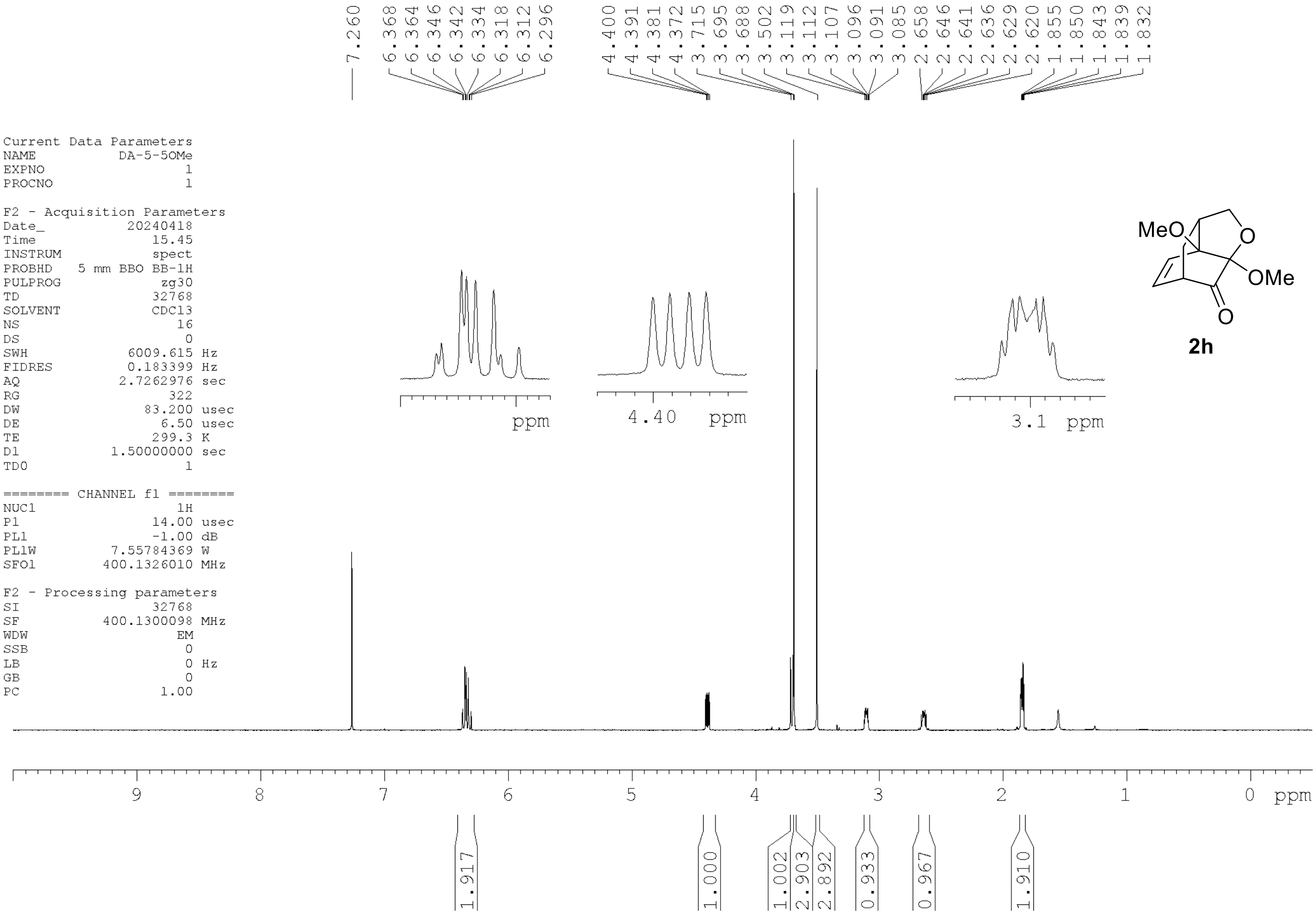


```
Current Data Parameters
NAME          DA-5-5OMe
EXPNO                 1
PROCNO                1

F2 - Acquisition Parameters
Date_          20240418
Time              15.45
INSTRUM           spect
PROBHD   5 mm BBO BB-1H
PULPROG            zg30
TD                32768
SOLVENT           CDCl3
NS                   16
DS                    0
SWH            6009.615 Hz
FIDRES         0.183399 Hz
AQ            2.7262976 sec
RG                  322
DW               83.200 usec
DE                 6.50 usec
TE                299.3 K
D1           1.50000000 sec
TD0                   1

======== CHANNEL f1 ========
NUC1                 1H
P1                14.00 usec
PL1               -1.00 dB
PL1W         7.55784369 W
SFO1        400.1326010 MHz

F2 - Processing parameters
SI                32768
SF          400.1300098 MHz
WDW                  EM
SSB                   0
LB                    0 Hz
GB                    0
PC                 1.00
```

^{1}H NMR of 2i

```
Current Data Parameters
NAME          DA-Me-4Br
EXPNO                 1
PROCNO                1

F2 - Acquisition Parameters
Date_          20240108
Time              19.15
INSTRUM           spect
PROBHD   5 mm BBO BB-1H
PULPROG            zg30
TD                32768
SOLVENT           CDCl3
NS                   16
DS                    0
SWH            6009.615 Hz
FIDRES         0.183399 Hz
AQ            2.7262976 sec
RG                  256
DW               83.200 usec
DE                 6.50 usec
TE                293.8 K
D1           1.50000000 sec
TD0                   1

======== CHANNEL f1 ========
NUC1                 1H
P1                14.00 usec
PL1               -1.00 dB
PL1W         7.55784369 W
SFO1        400.1326010 MHz

F2 - Processing parameters
SI                32768
SF          400.1300101 MHz
WDW                  EM
SSB                   0
LB                    0 Hz
GB                    0
PC                 1.00
```

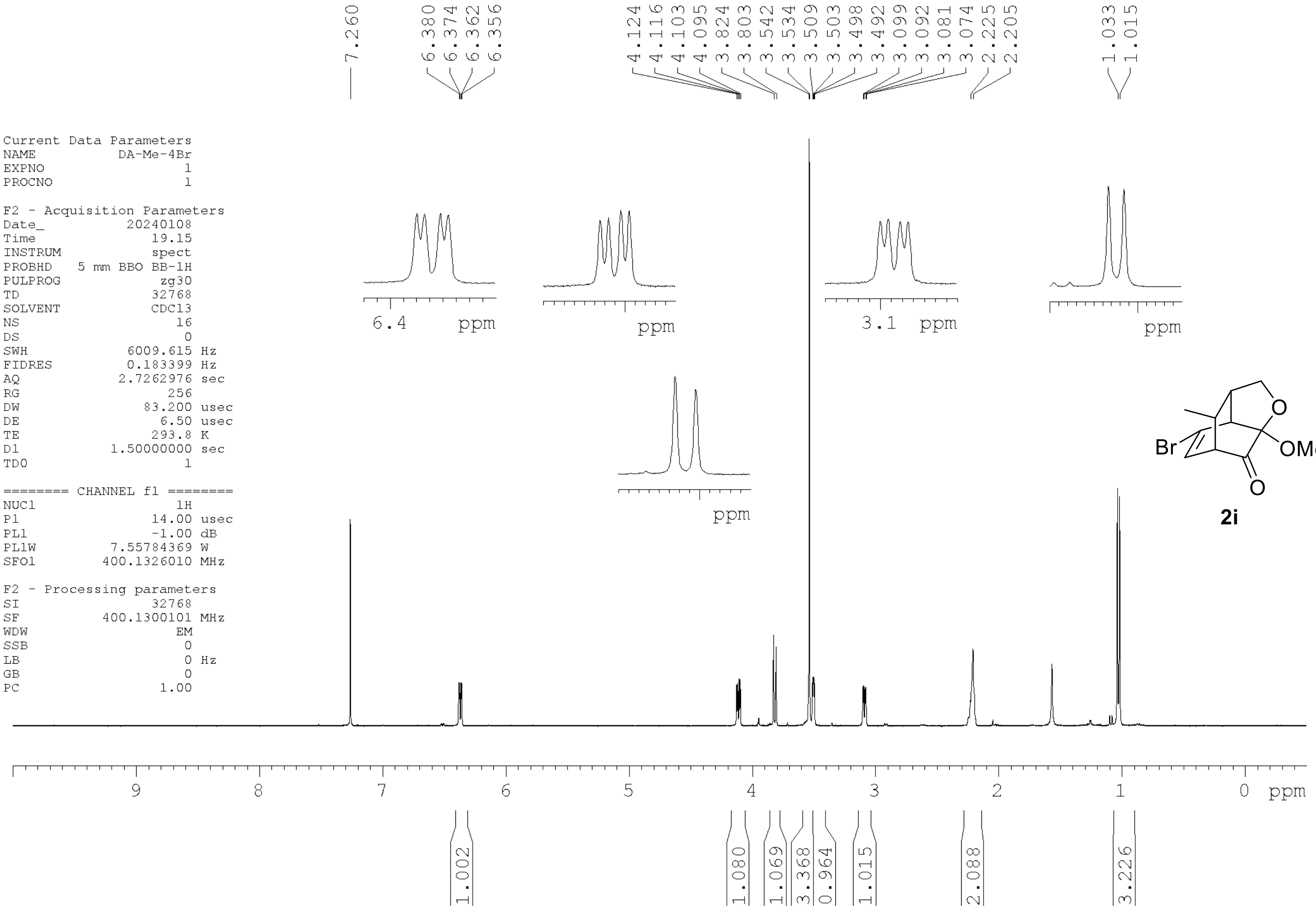

2i

^{1}H NMR of 2j

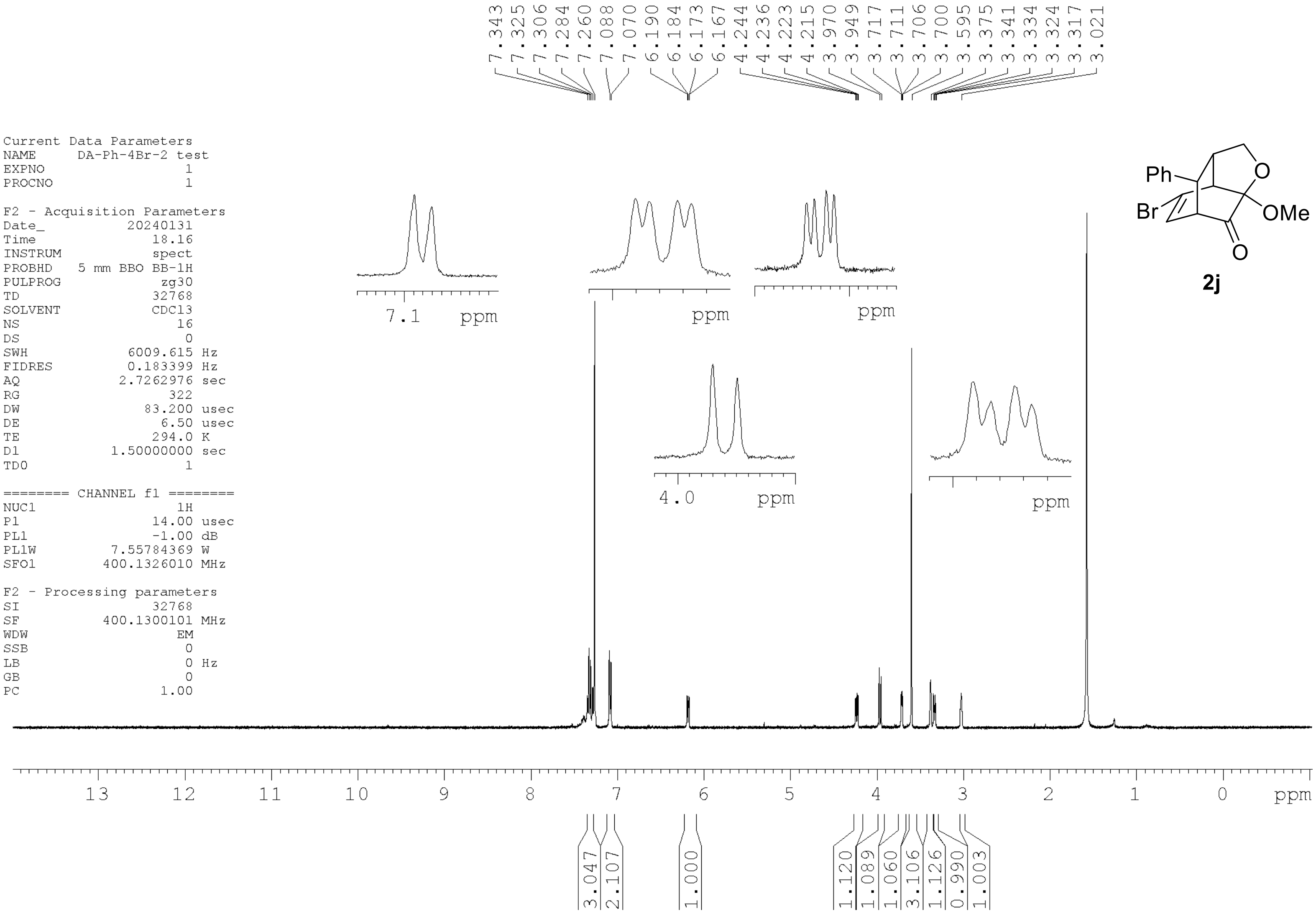

2j

^{1}H NMR of 2k

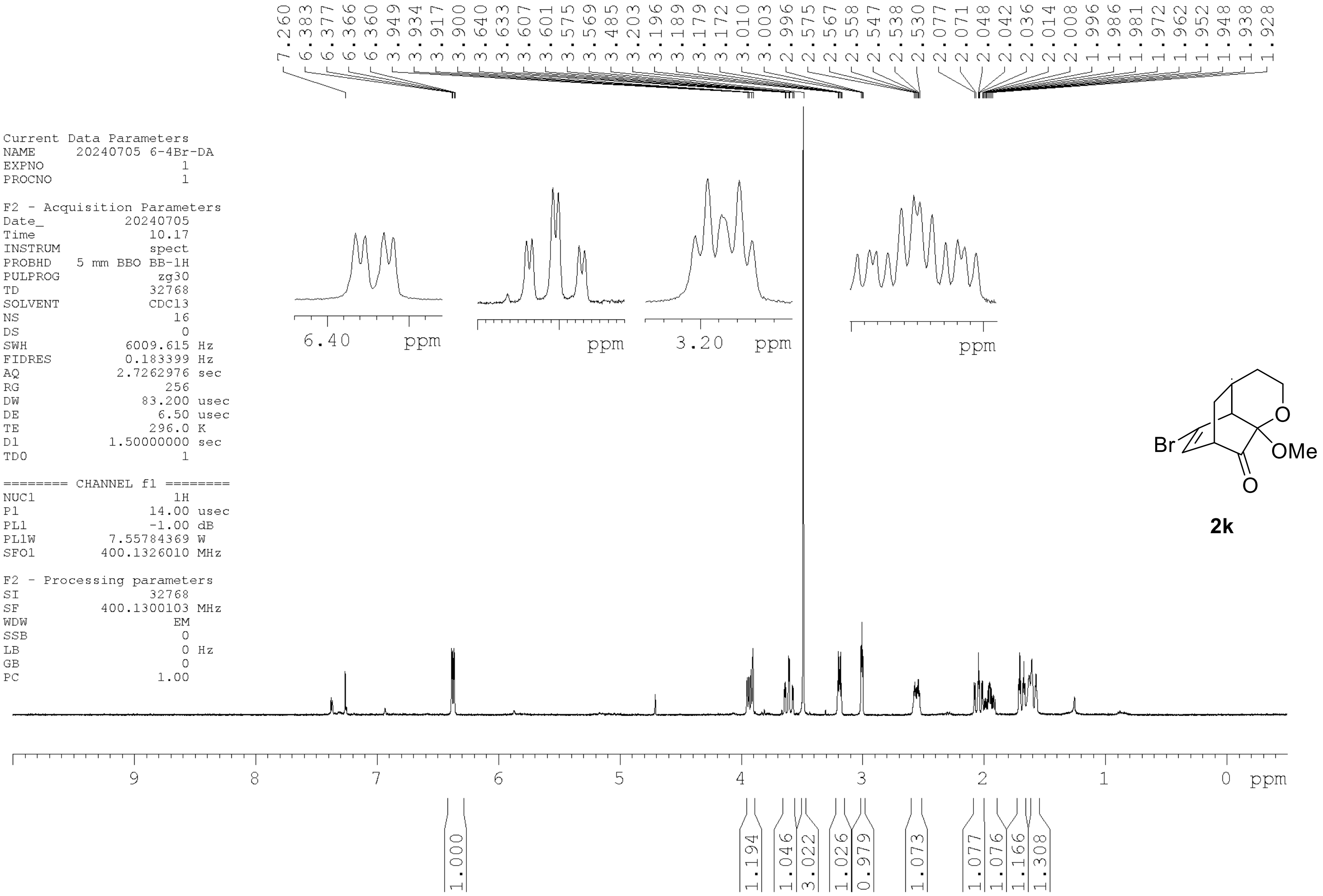

2k

^{1}H NMR of 3a

Current Data Parameters
NAME Oxime-5-4Br-1
EXPNO 1
PROCNO 1

F2 - Acquisition Parameters
Date_ 20230619
Time 16.49
INSTRUM spect
PROBHD 5 mm BBO BB-1H
PULPROG zg30
TD 32768
SOLVENT CDCl3
NS 16
DS 0
SWH 6009.615 Hz
FIDRES 0.183399 Hz
AQ 2.7262976 sec
RG 287
DW 83.200 usec
DE 6.50 usec
TE 296.8 K
D1 1.50000000 sec
TD0 1

======== CHANNEL f1 ========
NUC1 1H
P1 14.00 usec
PL1 -1.00 dB
PL1W 7.55784369 W
SFO1 400.1326010 MHz

F2 - Processing parameters
SI 32768
SF 400.1300099 MHz
WDW EM
SSB 0
LB 0 Hz
GB 0
PC 1.00

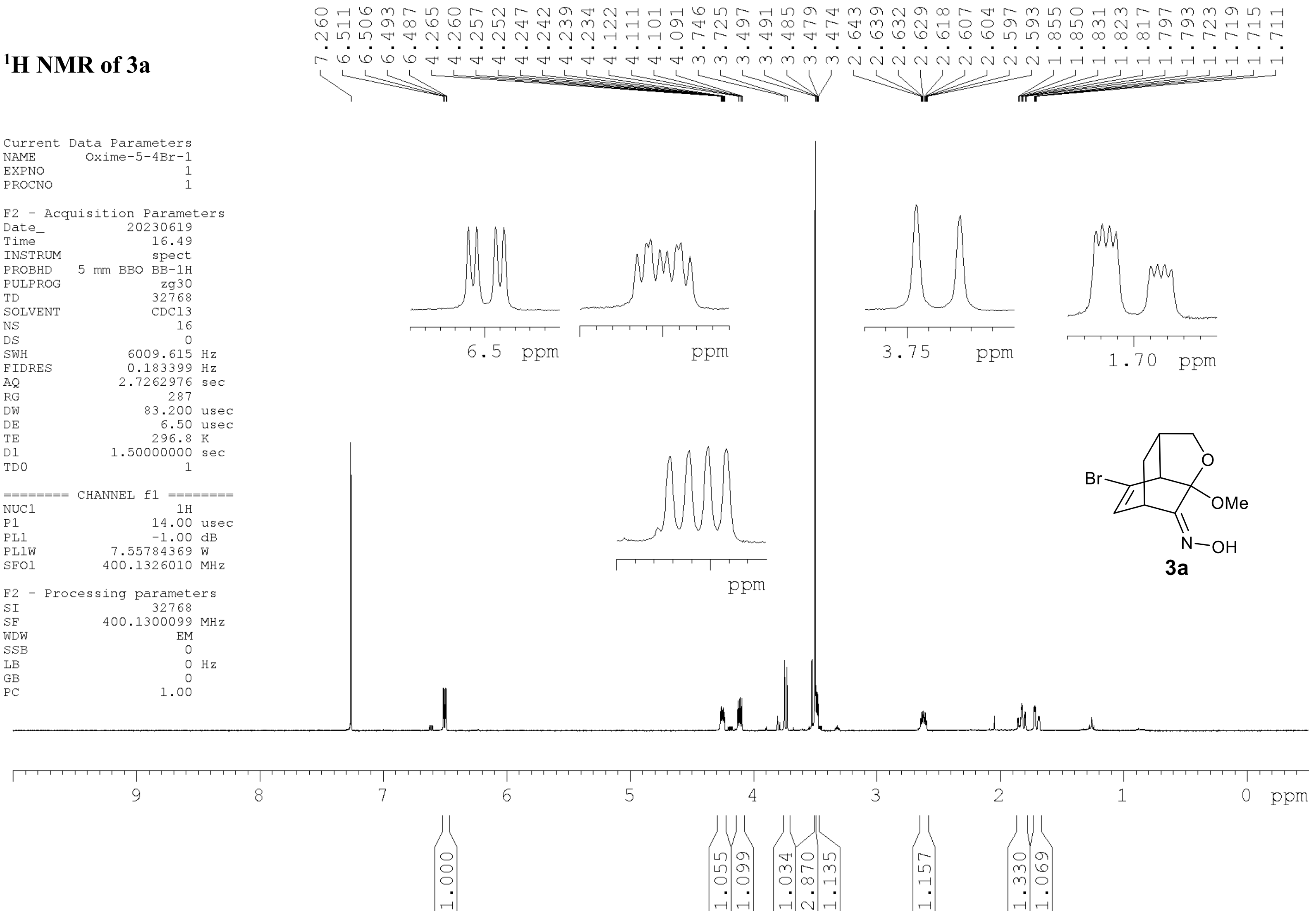

3a

^{13}C{1H} and DEPT 90, 135 NMR of 3a

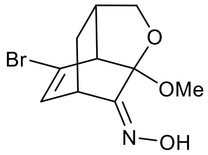

3a

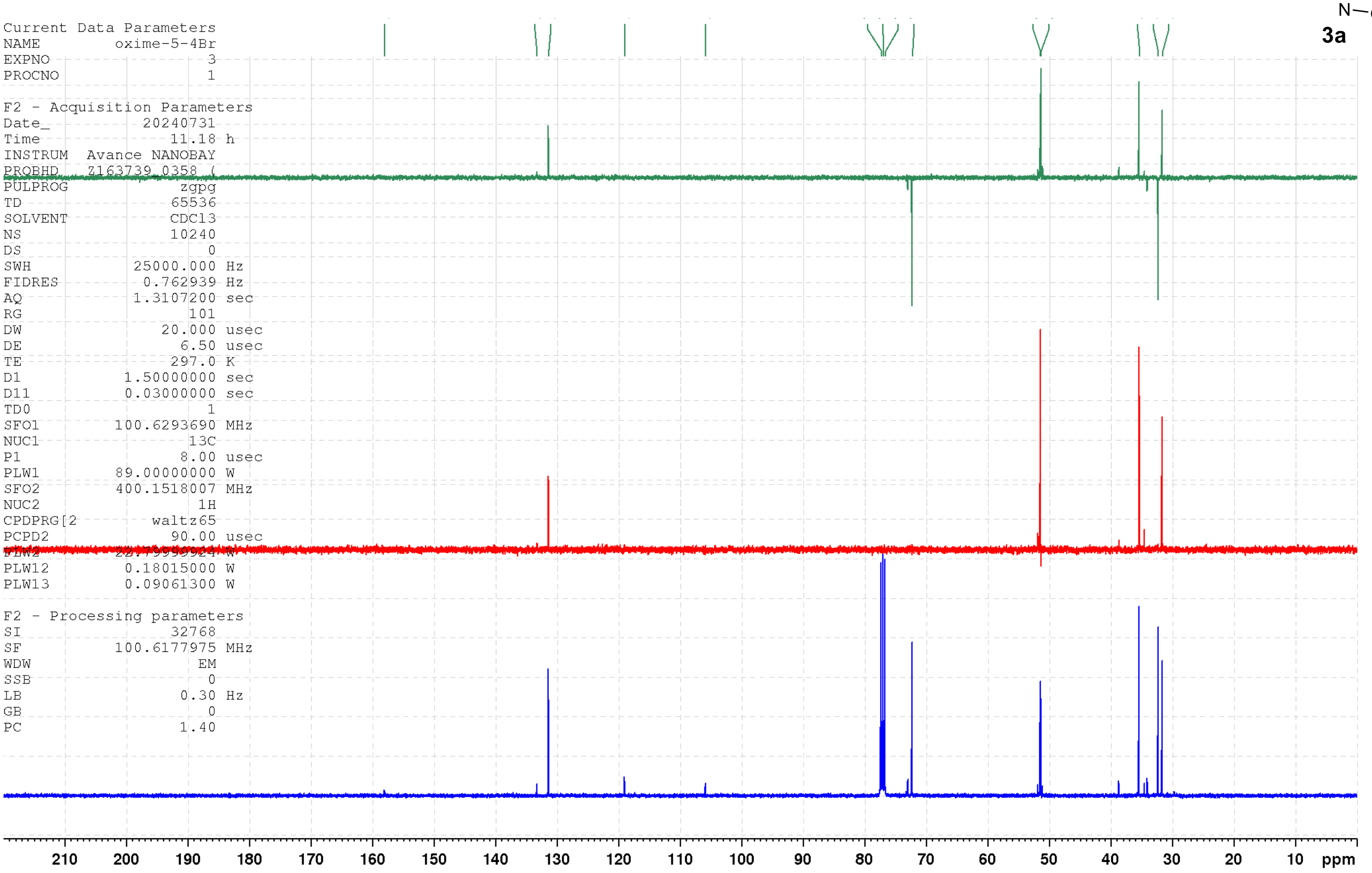

^{1}H NMR of 3b

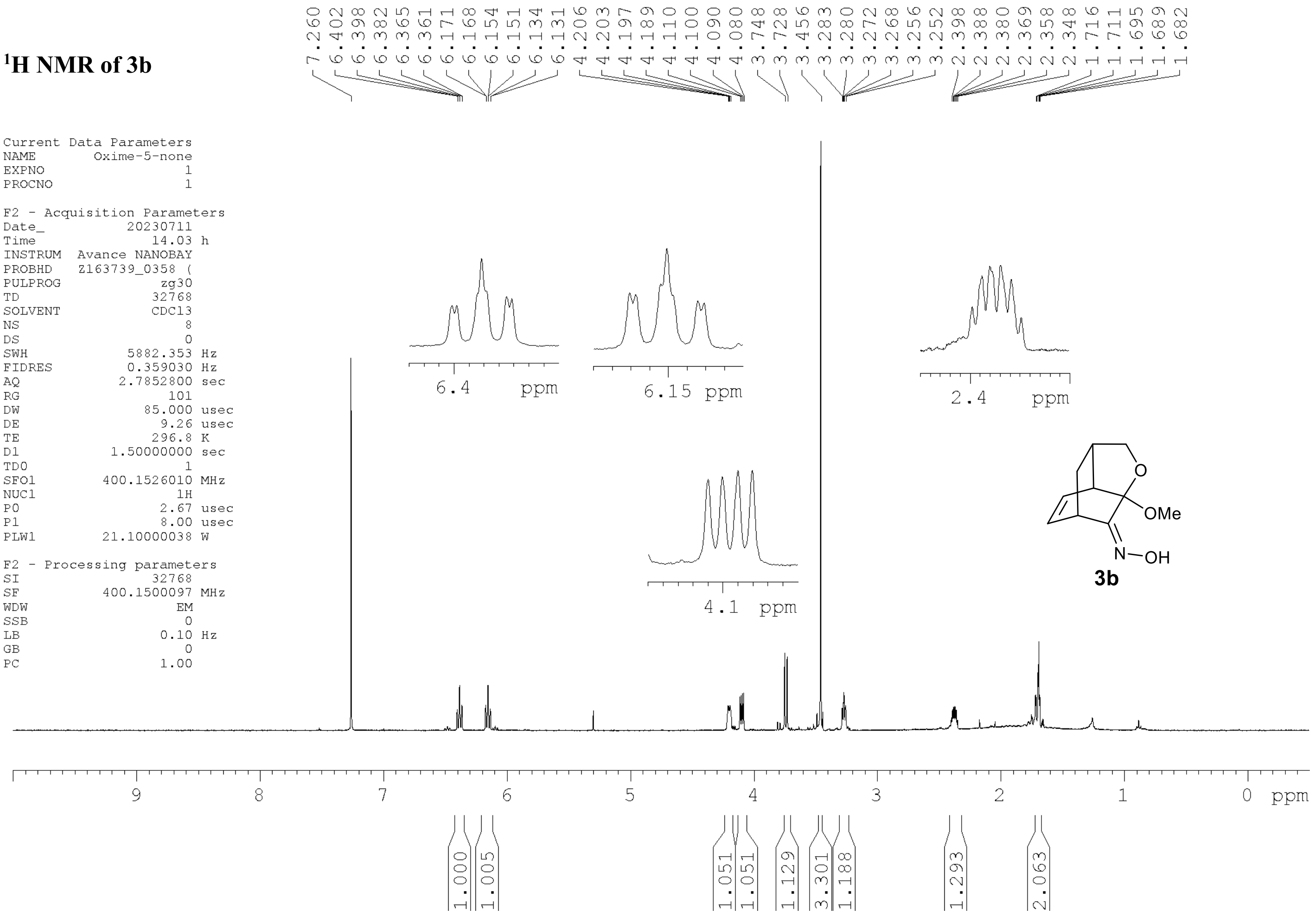

7.260
6.402
6.398
6.382
6.365
6.361
6.171
6.168
6.154
6.151
6.134
6.131
4.206
4.203
4.197
4.189
4.110
4.100
4.090
4.080
3.748
3.728
3.456
3.283
3.280
3.272
3.268
3.256
3.252
2.398
2.388
2.380
2.369
2.358
2.348
1.716
1.711
1.695
1.689
1.682
Current Data Parameters
NAME Oxime-5-none
EXPNO 1
PROCNO 1
F2 - Acquisition Parameters
Date_ 20230711
Time 14.03 h
INSTRUM Avance NANOBAY
PROBHD Z163739_0358 (
PULPROG zg30
TD 32768
SOLVENT CDCl3
NS 8
DS 0
SWH 5882.353 Hz
FIDRES 0.359030 Hz
AQ 2.7852800 sec
RG 101
DW 85.000 usec
DE 9.26 usec
TE 296.8 K
D1 1.50000000 sec
TD0 1
SFO1 400.1526010 MHz
NUC1 1H
P0 2.67 usec
P1 8.00 usec
PLW1 21.10000038 W
F2 - Processing parameters
SI 32768
SF 400.1500097 MHz
WDW EM
SSB 0
LB 0.10 Hz
GB 0
PC 1.00
6.4 ppm
6.15 ppm
2.4 ppm
4.1 ppm
O
OMe
N–OH
3b
9
8
7
6
5
4
3
2
1
0 ppm
1.000
1.005
1.051
1.051
1.129
3.301
1.188
1.293
2.063

^{13}C{1H} and DEPT 90, 135 NMR of 3b

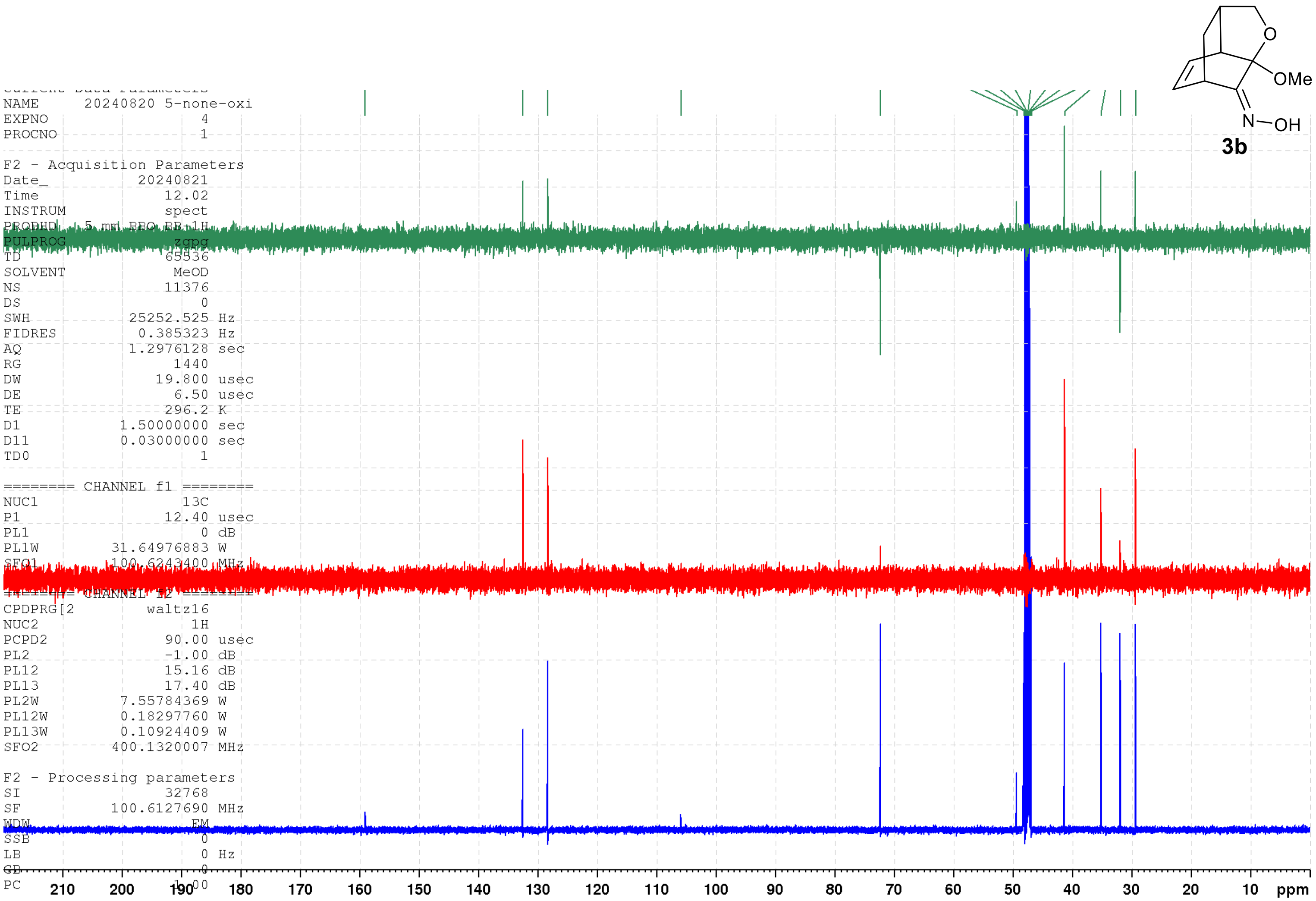

^{1}H NMR of 3c

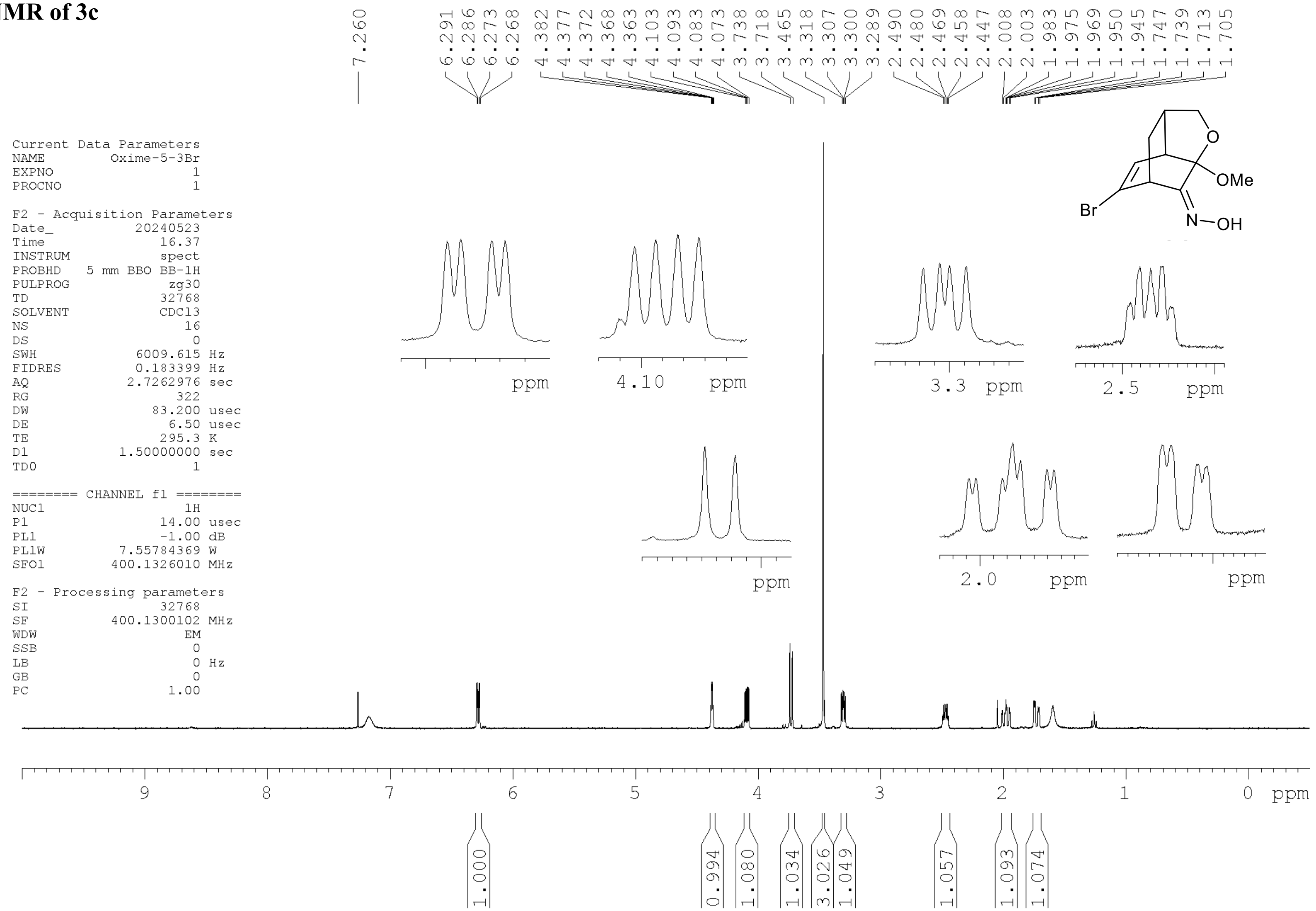
Current Data Parameters
NAME Oxime-5-3Br
EXPNO 1
PROCNO 1
F2 - Acquisition Parameters
Date_ 20240523
Time 16.37
INSTRUM spect
PROBHD 5 mm BBO BB-1H
PULPROG zg30
TD 32768
SOLVENT CDCl3
NS 16
DS 0
SWH 6009.615 Hz
FIDRES 0.183399 Hz
AQ 2.7262976 sec
RG 322
DW 83.200 usec
DE 6.50 usec
TE 295.3 K
D1 1.50000000 sec
TD0 1
======== CHANNEL f1 ========
NUC1 1H
P1 14.00 usec
PL1 -1.00 dB
PL1W 7.55784369 W
SFO1 400.1326010 MHz
F2 - Processing parameters
SI 32768
SF 400.1300102 MHz
WDW EM
SSB 0
LB 0 Hz
GB 0
PC 1.00
7.260
6.291
6.286
6.273
6.268
4.382
4.377
4.372
4.368
4.363
4.103
4.093
4.083
4.073
3.738
3.718
3.465
3.318
3.307
3.300
3.289
2.490
2.480
2.469
2.458
2.447
2.008
2.003
1.983
1.975
1.969
1.950
1.945
1.747
1.739
1.713
1.705
OMe
Br
N—OH
O
1.000
0.994
1.080
1.034
3.026
1.049
1.057
1.093
1.074
ppm

^{13}C{1H} and DEPT 90, 135 NMR of 3c

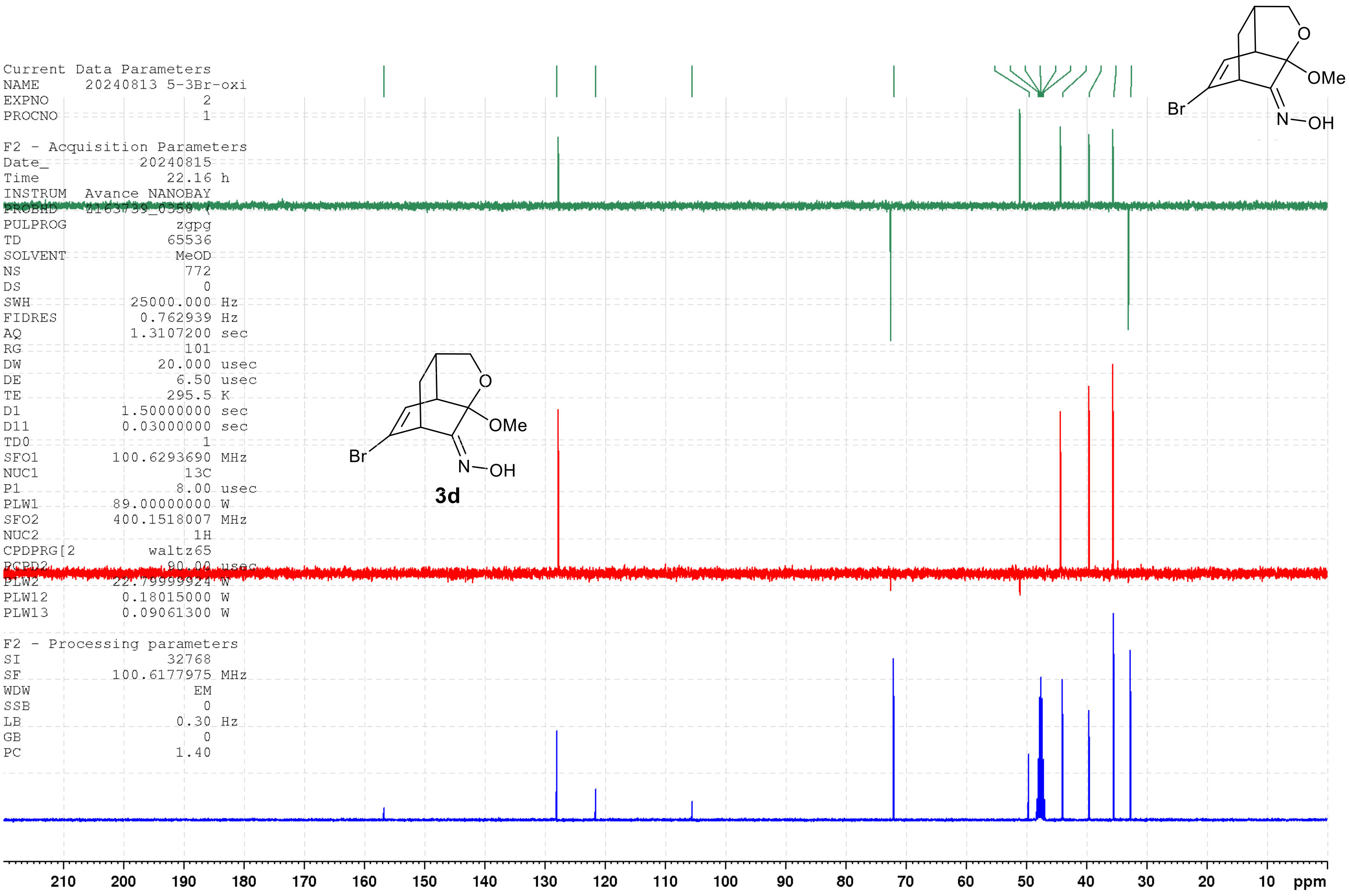

^{1}H NMR of 3d

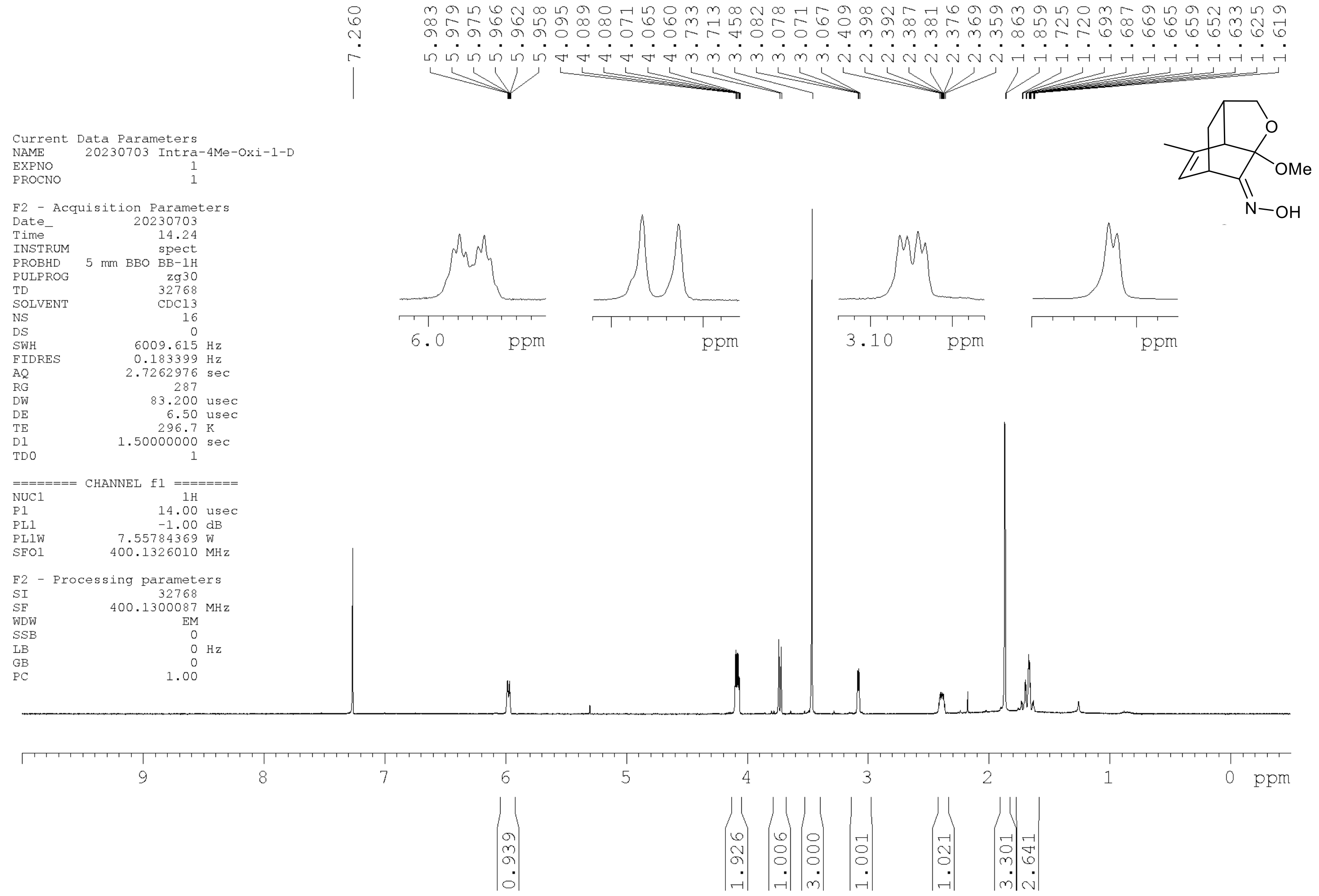

7.260
5.983
5.979
5.975
5.966
5.962
5.958
4.095
4.089
4.080
4.071
4.065
4.060
3.733
3.713
3.458
3.082
3.078
3.071
3.067
2.409
2.398
2.392
2.387
2.381
2.376
2.369
2.359
1.863
1.859
1.725
1.720
1.693
1.687
1.669
1.665
1.659
1.652
1.633
1.625
1.619
OMe
O
N
OH
Current Data Parameters
NAME 20230703 Intra-4Me-Oxi-1-D
EXPNO 1
PROCNO 1
F2 - Acquisition Parameters
Date_ 20230703
Time 14.24
INSTRUM spect
PROBHD 5 mm BBO BB-1H
PULPROG zg30
TD 32768
SOLVENT CDCl3
NS 16
DS 0
SWH 6009.615 Hz
FIDRES 0.183399 Hz
AQ 2.7262976 sec
RG 287
DW 83.200 usec
DE 6.50 usec
TE 296.7 K
D1 1.50000000 sec
TD0 1
======== CHANNEL f1 ========
NUC1 1H
P1 14.00 usec
PL1 -1.00 dB
PL1W 7.55784369 W
SFO1 400.1326010 MHz
F2 - Processing parameters
SI 32768
SF 400.1300087 MHz
WDW EM
SSB 0
LB 0 Hz
GB 0
PC 1.00
6.0
ppm
ppm
3.10
ppm
ppm
9
8
7
6
5
4
3
2
1
0
ppm
0.939
1.926
1.006
3.000
1.001
1.021
3.301
2.641

^{13}C\{1H\} and DEPT 90, 135 NMR of 3d

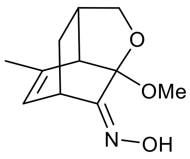

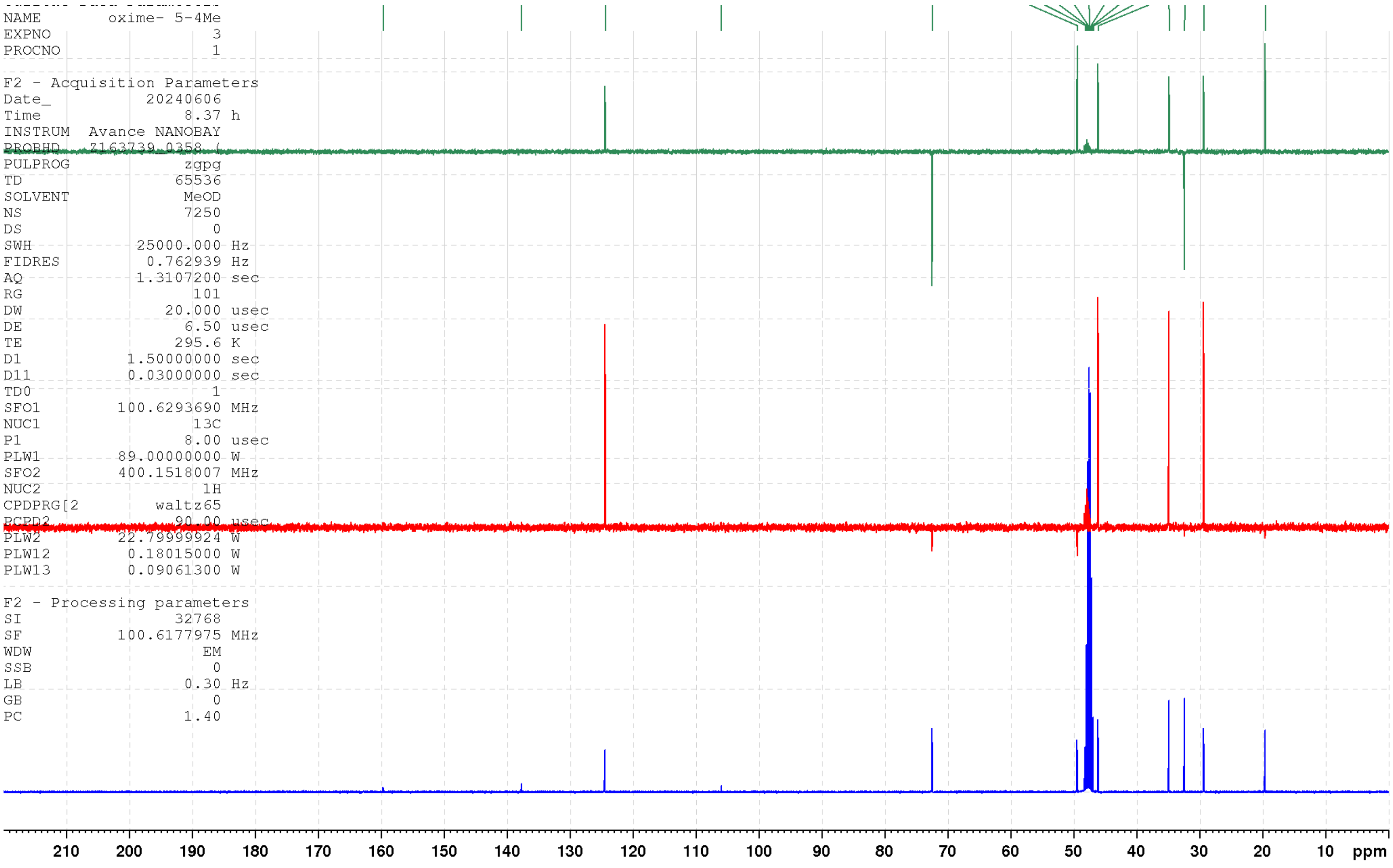

^{1}H NMR of 3e

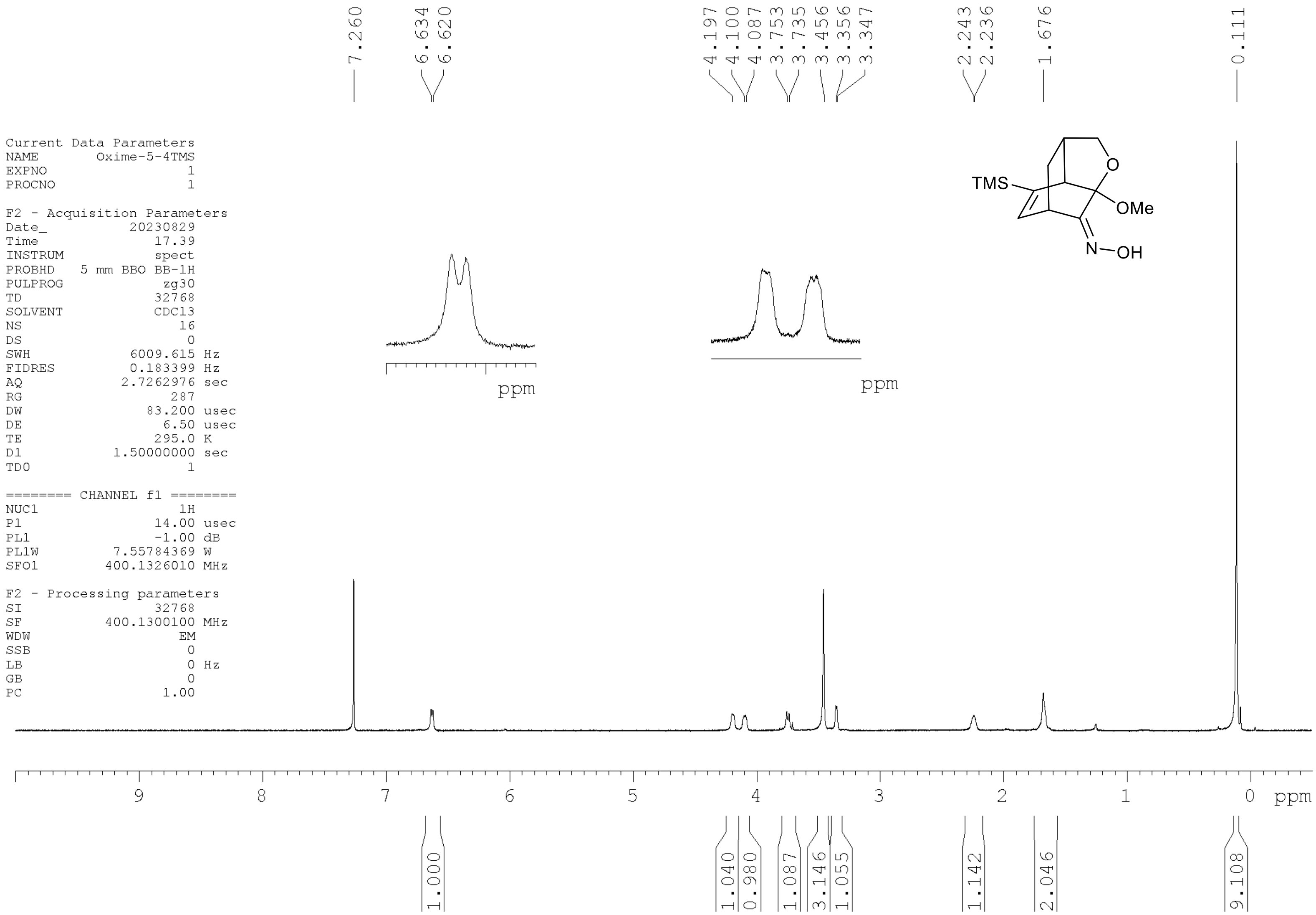

7.260
6.634
6.620
4.197
4.100
4.087
3.753
3.735
3.456
3.356
3.347
2.243
2.236
1.676
0.111
Current Data Parameters
NAME Oxime-5-4TMS
EXPNO 1
PROCNO 1
F2 - Acquisition Parameters
Date_ 20230829
Time 17.39
INSTRUM spect
PROBHD 5 mm BBO BB-1H
PULPROG zg30
TD 32768
SOLVENT CDCl3
NS 16
DS 0
SWH 6009.615 Hz
FIDRES 0.183399 Hz
AQ 2.7262976 sec
RG 287
DW 83.200 usec
DE 6.50 usec
TE 295.0 K
D1 1.50000000 sec
TD0 1
======== CHANNEL f1 ========
NUC1 1H
P1 14.00 usec
PL1 -1.00 dB
PL1W 7.55784369 W
SFO1 400.1326010 MHz
F2 - Processing parameters
SI 32768
SF 400.1300100 MHz
WDW EM
SSB 0
LB 0 Hz
GB 0
PC 1.00
TMS
O
OMe
N
OH
ppm
ppm
9
8
7
6
5
4
3
2
1
0
ppm
1.000
1.040
0.980
1.087
3.146
1.055
1.142
2.046
9.108

^{13}C{1H} and DEPT 90, 135 NMR of 3e

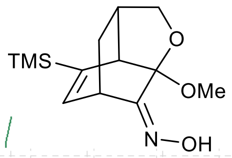

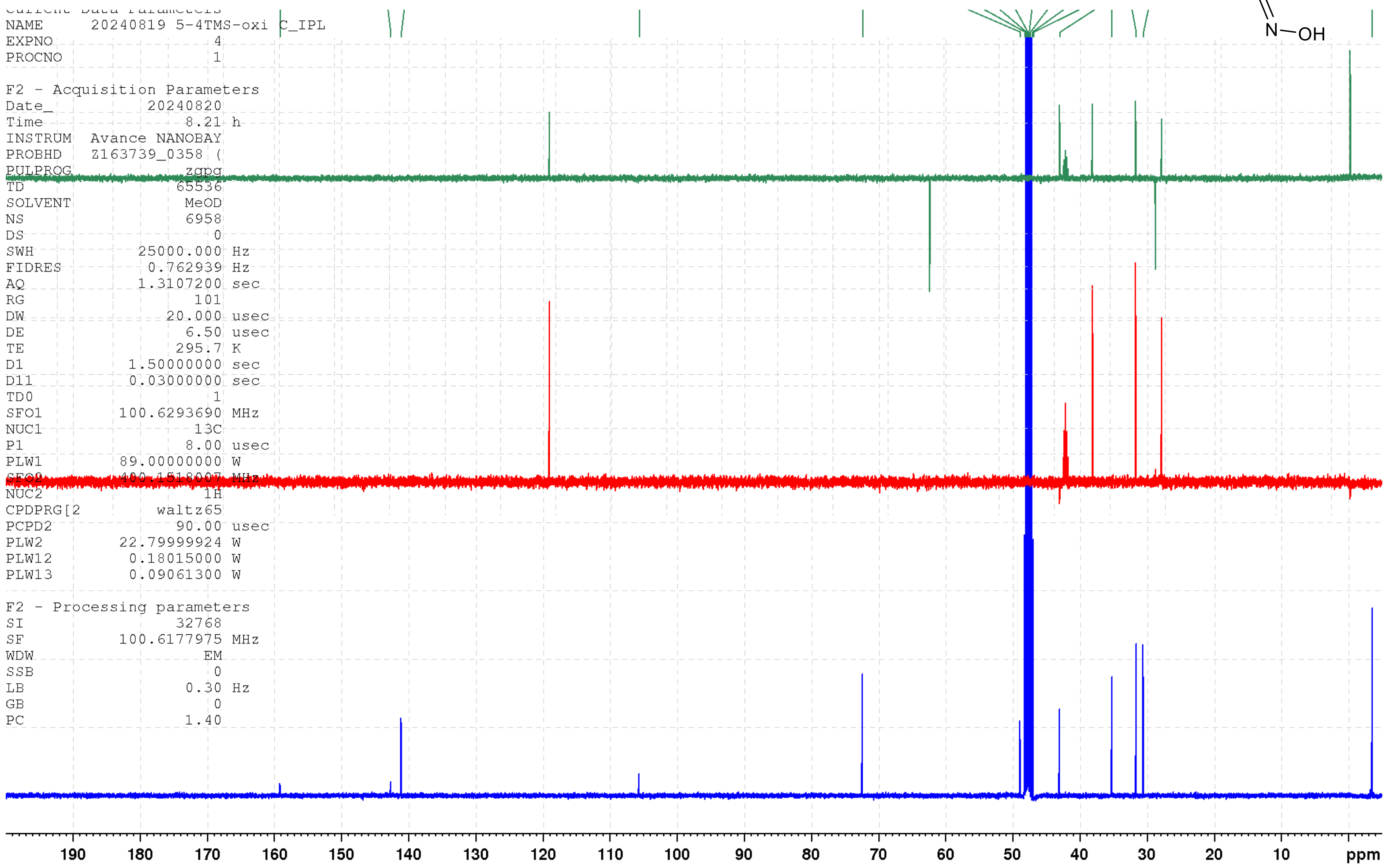

^{1}H NMR of 3f

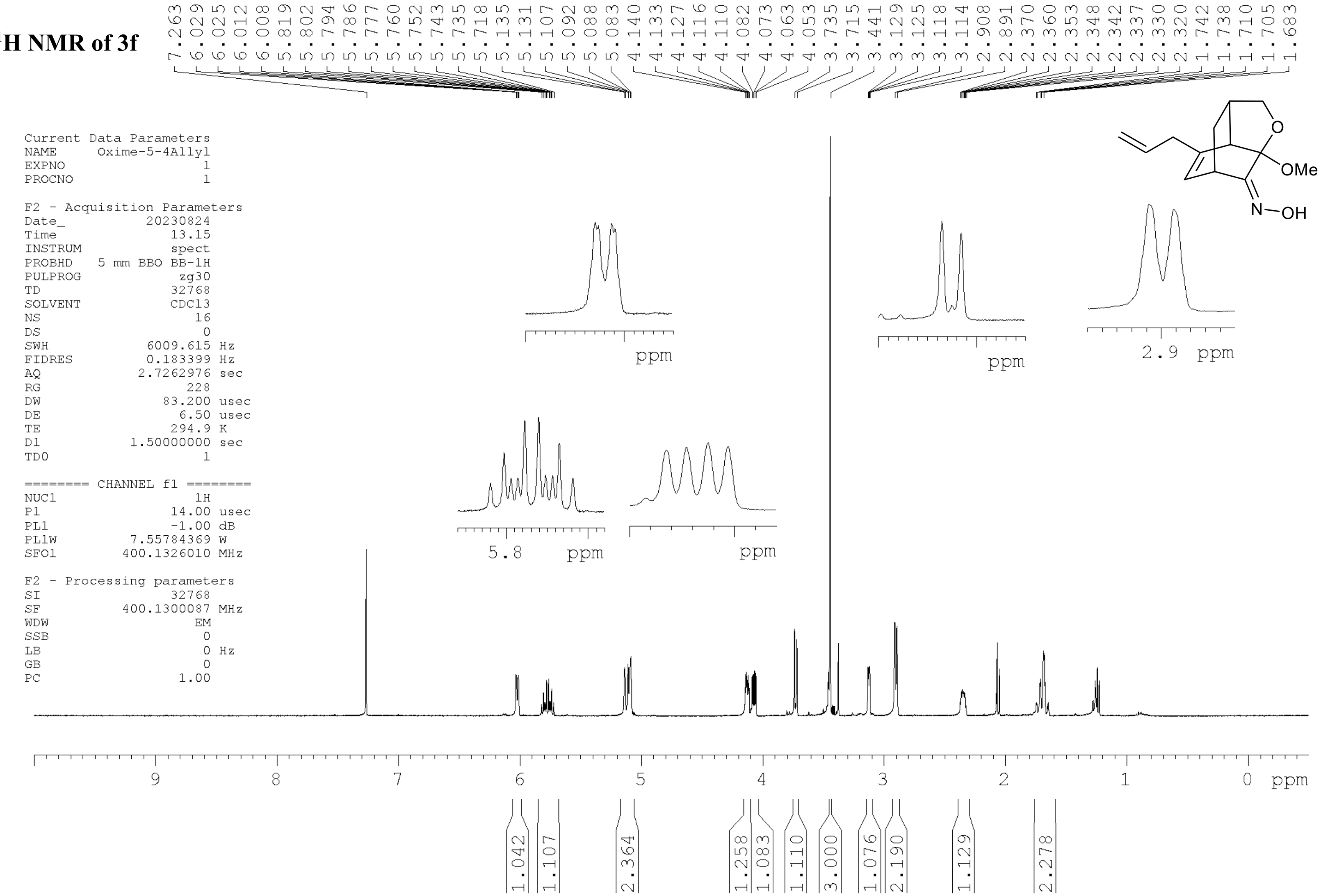
7.263 6.029 6.025 6.012 6.008 5.819 5.802 5.794 5.786 5.777 5.760 5.752 5.743 5.735 5.718 5.135 5.131 5.107 5.092 5.088 5.083 4.140 4.133 4.127 4.116 4.110 4.082 4.073 4.063 4.053 3.735 3.715 3.441 3.129 3.125 3.118 3.114 2.908 2.891 2.370 2.360 2.353 2.348 2.342 2.337 2.330 2.320 1.742 1.738 1.710 1.705 1.683
Current Data Parameters
NAME Oxime-5-4Allyl
EXPNO 1
PROCNO 1
F2 - Acquisition Parameters
Date_ 20230824
Time 13.15
INSTRUM spect
PROBHD 5 mm BBO BB-1H
PULPROG zg30
TD 32768
SOLVENT CDCl3
NS 16
DS 0
SWH 6009.615 Hz
FIDRES 0.183399 Hz
AQ 2.7262976 sec
RG 228
DW 83.200 usec
DE 6.50 usec
TE 294.9 K
D1 1.50000000 sec
TD0 1
======== CHANNEL f1 ========
NUC1 1H
P1 14.00 usec
PL1 -1.00 dB
PL1W 7.55784369 W
SFO1 400.1326010 MHz
F2 - Processing parameters
SI 32768
SF 400.1300087 MHz
WDW EM
SSB 0
LB 0 Hz
GB 0
PC 1.00
O
OMe
N
OH
ppm
ppm
2.9 ppm
5.8 ppm
ppm
9 8 7 6 5 4 3 2 1 0 ppm
1.042 1.107 2.364 1.258 1.083 1.110 3.000 1.076 2.190 1.129 2.278

^{13}C{1H} and DEPT 90, 135 NMR of 3f

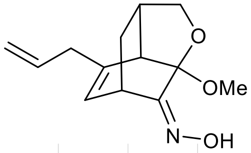

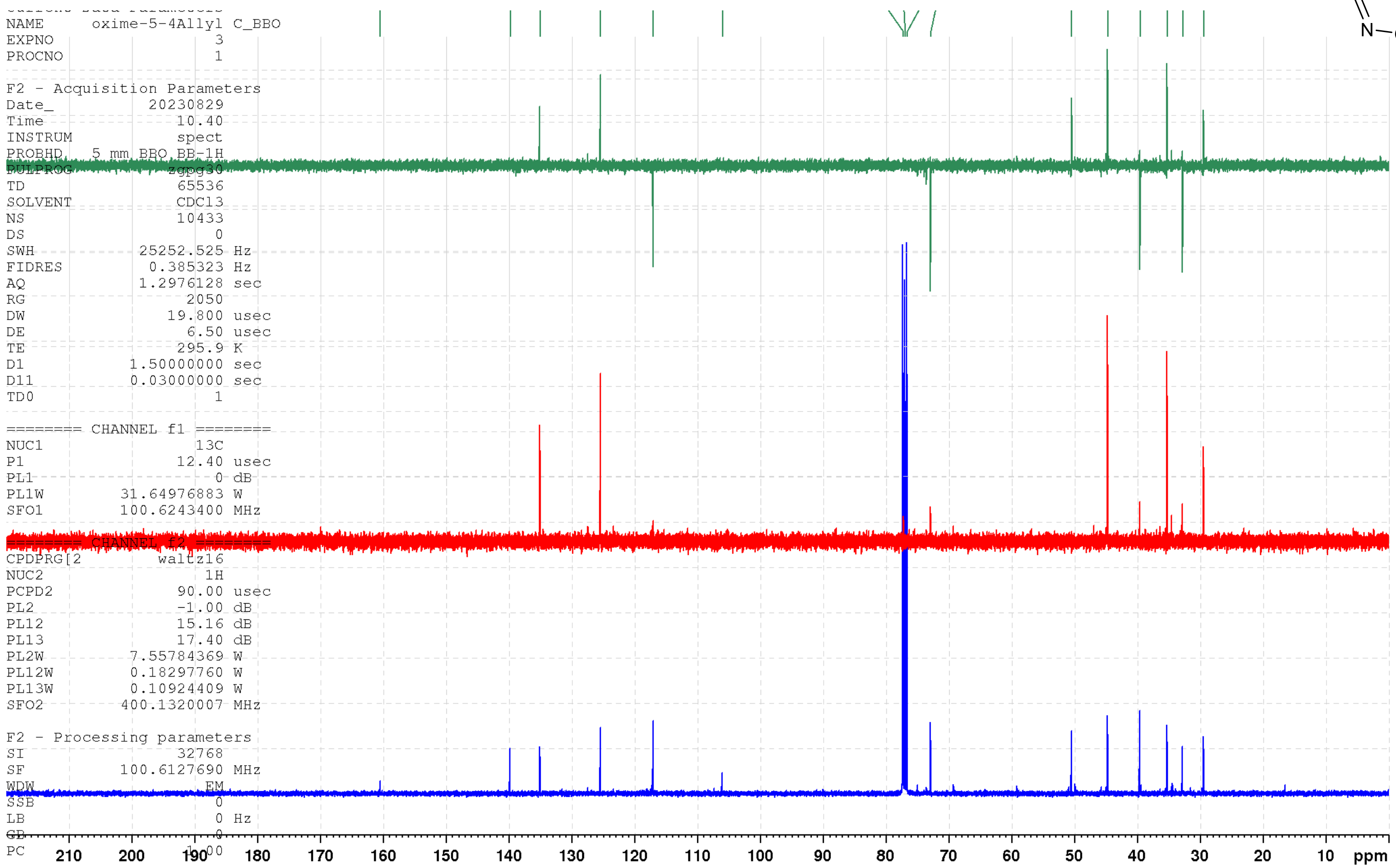

^{1}H NMR of 3g

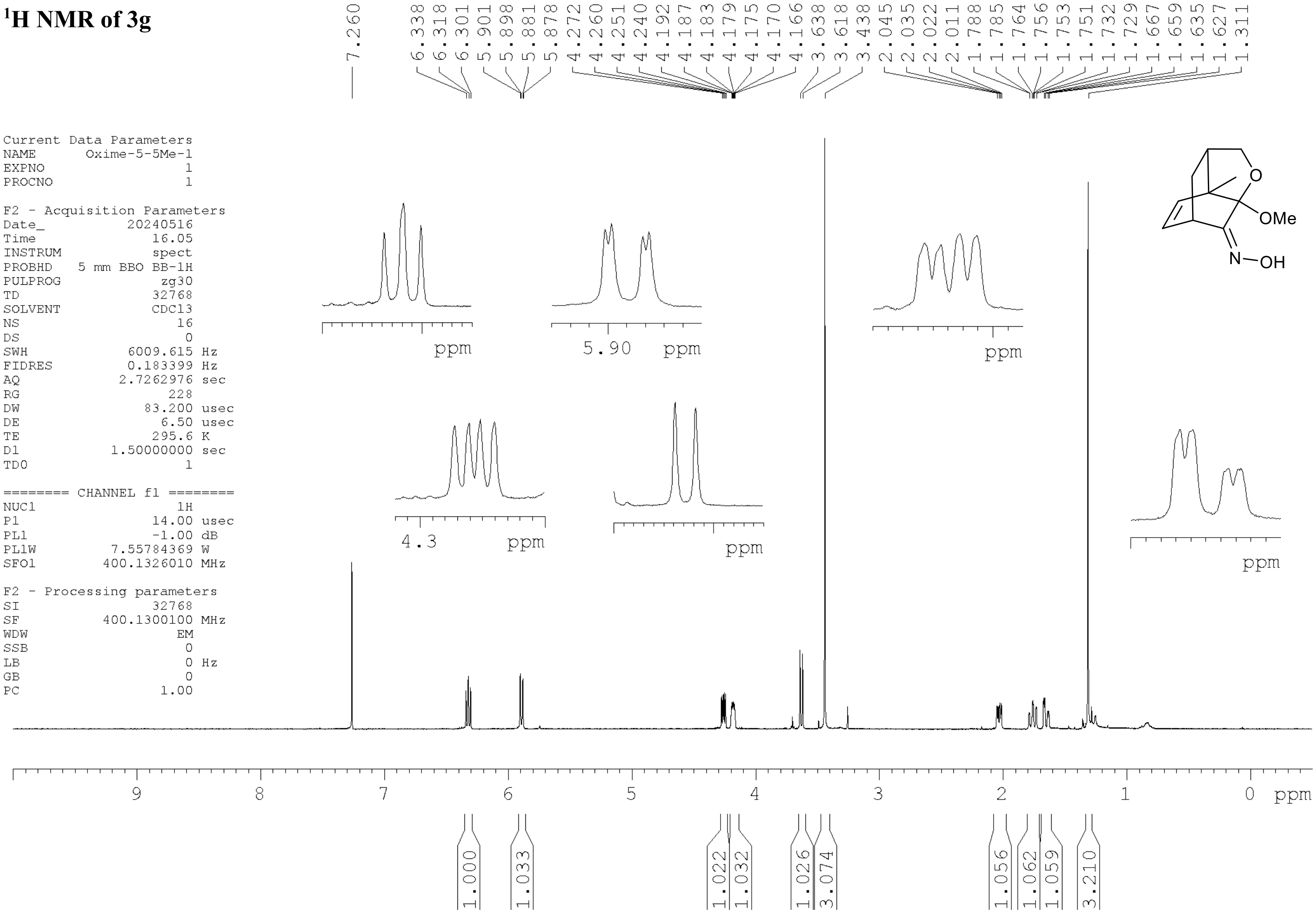
Current Data Parameters
NAME Oxime-5-5Me-1
EXPNO 1
PROCNO 1
F2 - Acquisition Parameters
Date_ 20240516
Time 16.05
INSTRUM spect
PROBHD 5 mm BBO BB-1H
PULPROG zg30
TD 32768
SOLVENT CDCl3
NS 16
DS 0
SWH 6009.615 Hz
FIDRES 0.183399 Hz
AQ 2.7262976 sec
RG 228
DW 83.200 usec
DE 6.50 usec
TE 295.6 K
D1 1.50000000 sec
TD0 1
======== CHANNEL f1 ========
NUC1 1H
P1 14.00 usec
PL1 -1.00 dB
PL1W 7.55784369 W
SFO1 400.1326010 MHz
F2 - Processing parameters
SI 32768
SF 400.1300100 MHz
WDW EM
SSB 0
LB 0 Hz
GB 0
PC 1.00
7.260
6.338 6.318 6.301 5.901 5.898 5.881 5.878 4.272 4.260 4.251 4.240 4.192 4.187 4.183 4.179 4.175 4.170 4.166 3.638 3.618 3.438 2.045 2.035 2.022 2.011 1.788 1.785 1.764 1.756 1.753 1.751 1.732 1.729 1.667 1.659 1.635 1.627 1.311
OMe
O
N−OH
5.90 ppm
4.3 ppm
ppm
1.000 1.033 1.022 1.032 1.026 3.074 1.056 1.062 1.059 3.210

^{13}C{1H} and DEPT 90, 135 NMR of 3g

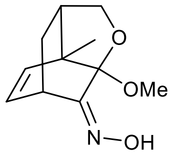

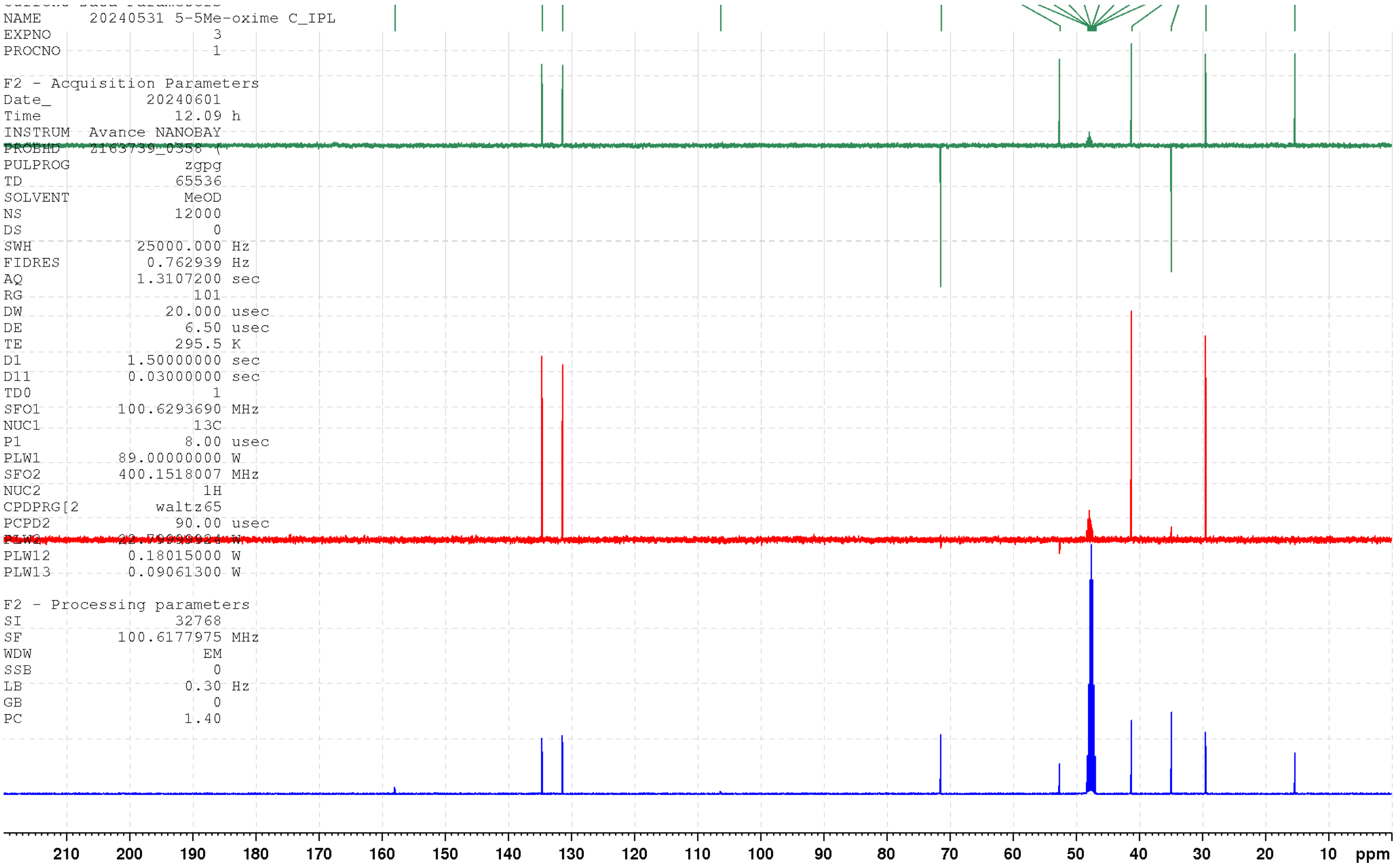

^{1}H NMR of 3h

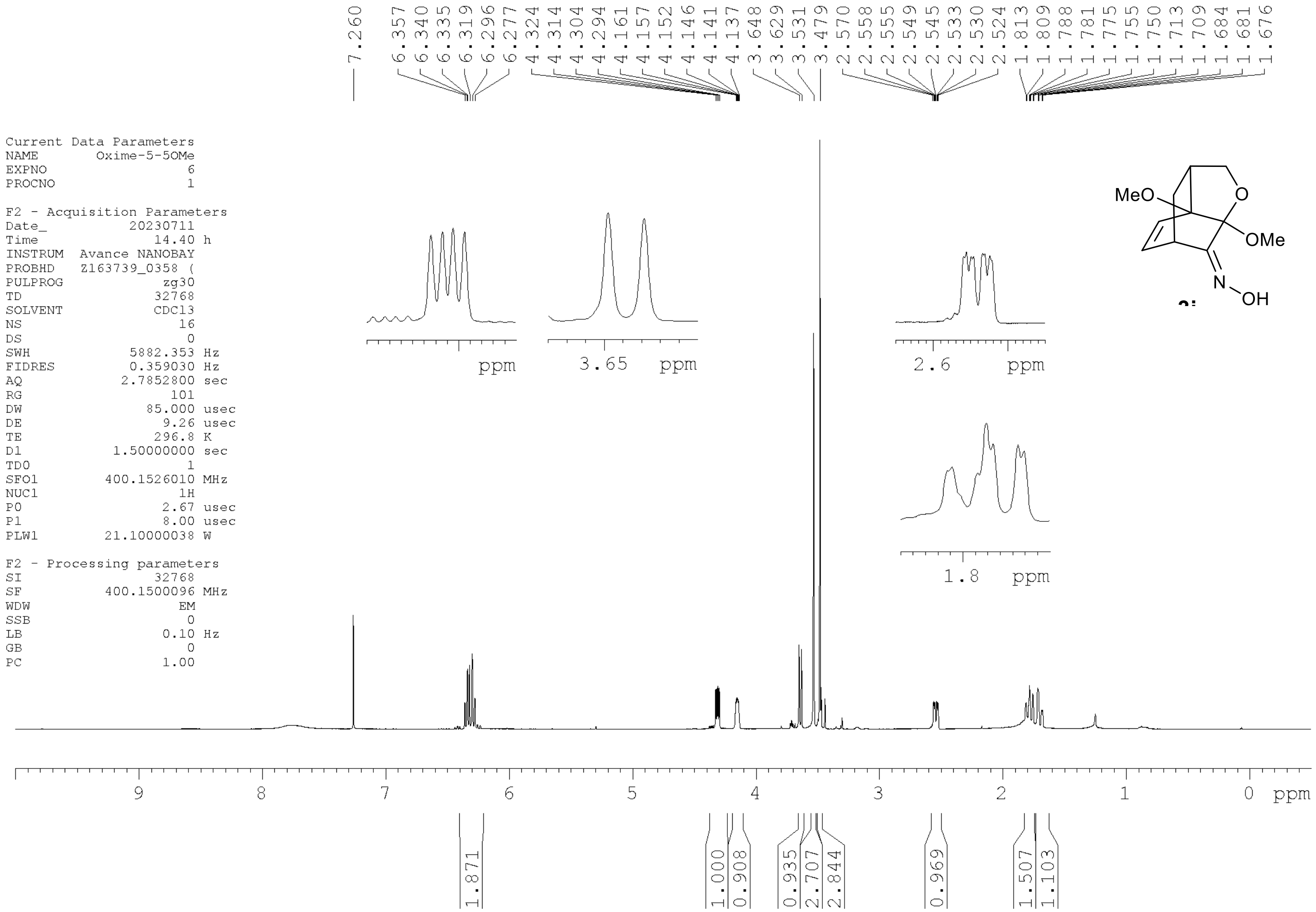
7.260
6.357 6.340 6.335 6.319 6.296 6.277
4.324 4.314 4.304 4.294 4.161 4.157 4.152 4.146 4.141 4.137
3.648 3.629 3.531 3.479
2.570 2.558 2.555 2.549 2.545 2.533 2.530 2.524
1.813 1.809 1.788 1.781 1.775 1.755 1.750 1.713 1.709 1.684 1.681 1.676
Current Data Parameters
NAME Oxime-5-5OMe
EXPNO 6
PROCNO 1
F2 - Acquisition Parameters
Date_ 20230711
Time 14.40 h
INSTRUM Avance NANOBAY
PROBHD Z163739_0358 (
PULPROG zg30
TD 32768
SOLVENT CDCl3
NS 16
DS 0
SWH 5882.353 Hz
FIDRES 0.359030 Hz
AQ 2.7852800 sec
RG 101
DW 85.000 usec
DE 9.26 usec
TE 296.8 K
D1 1.50000000 sec
TD0 1
SFO1 400.1526010 MHz
NUC1 1H
P0 2.67 usec
P1 8.00 usec
PLW1 21.10000038 W
F2 - Processing parameters
SI 32768
SF 400.1500096 MHz
WDW EM
SSB 0
LB 0.10 Hz
GB 0
PC 1.00
MeO
O
OMe
N
OH
ppm
3.65 ppm
2.6 ppm
1.8 ppm
9 8 7 6 5 4 3 2 1 0 ppm
1.871
1.000 0.908
0.935 2.707 2.844
0.969
1.507 1.103

^{13}C{1H} and DEPT 90, 135 NMR of 3h

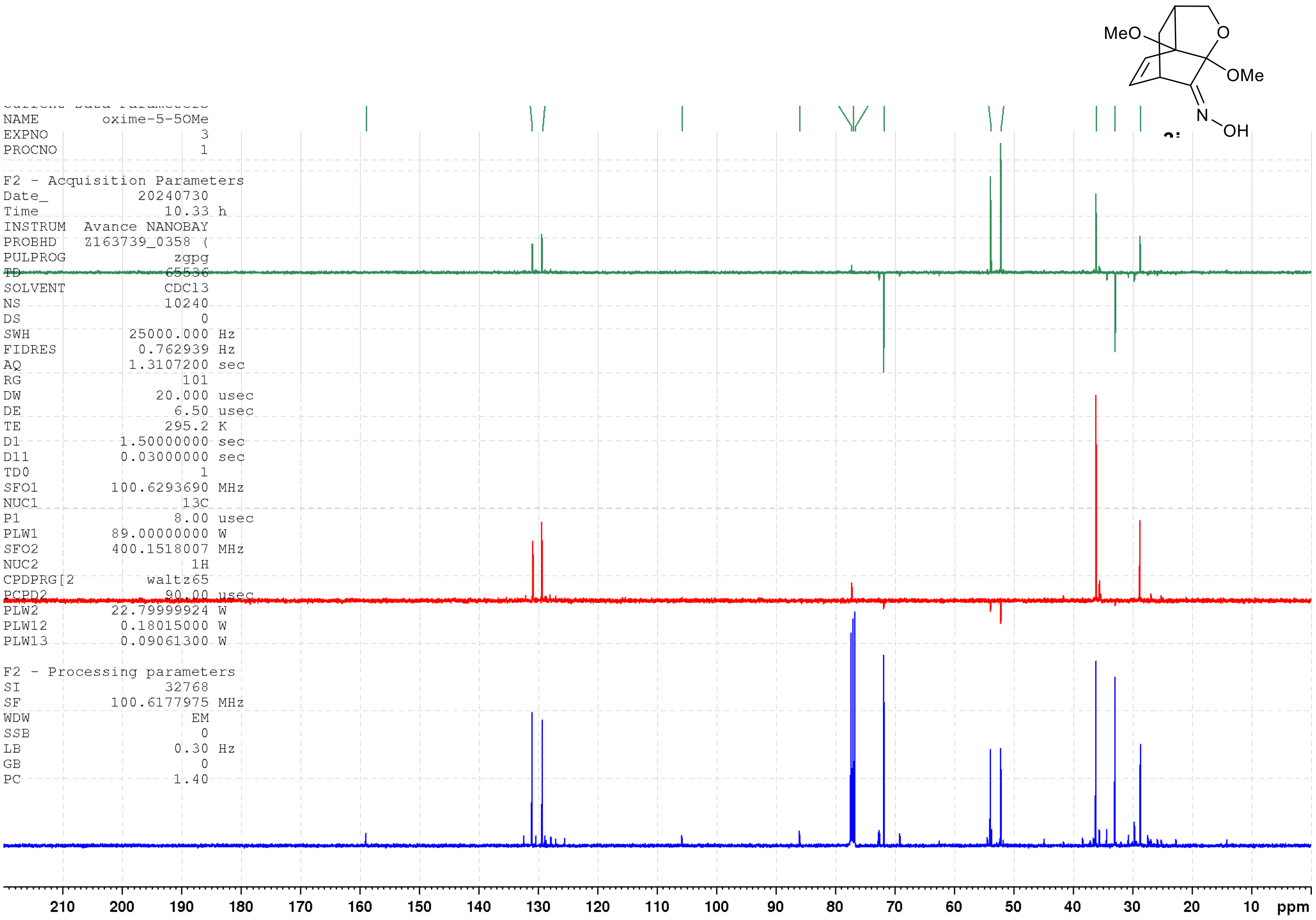

^{1}H NMR of 3i

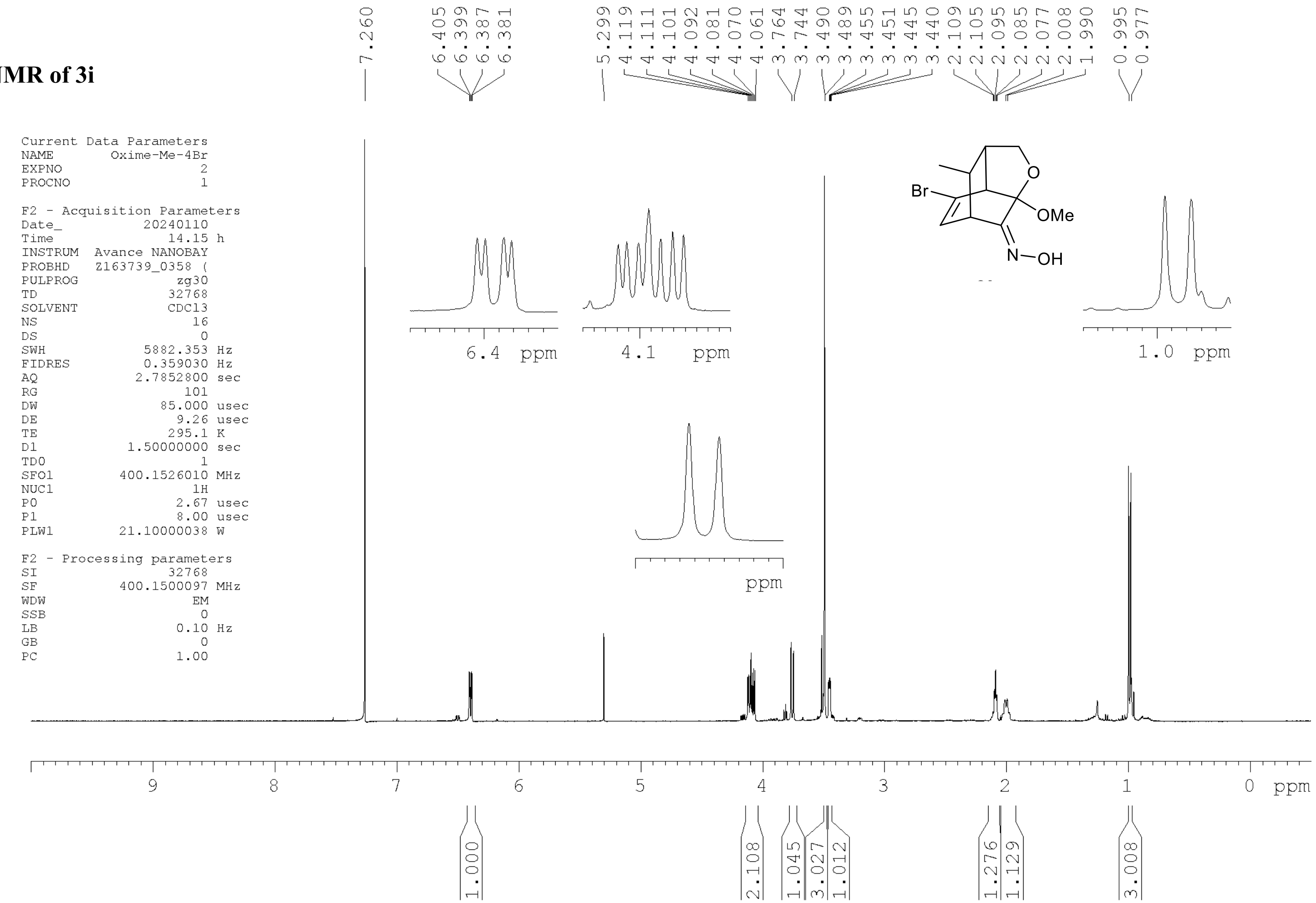
Current Data Parameters
NAME Oxime-Me-4Br
EXPNO 2
PROCNO 1
F2 - Acquisition Parameters
Date_ 20240110
Time 14.15 h
INSTRUM Avance NANOBAY
PROBHD Z163739_0358 (
PULPROG zg30
TD 32768
SOLVENT CDCl3
NS 16
DS 0
SWH 5882.353 Hz
FIDRES 0.359030 Hz
AQ 2.7852800 sec
RG 101
DW 85.000 usec
DE 9.26 usec
TE 295.1 K
D1 1.50000000 sec
TD0 1
SFO1 400.1526010 MHz
NUC1 1H
P0 2.67 usec
P1 8.00 usec
PLW1 21.10000038 W
F2 - Processing parameters
SI 32768
SF 400.1500097 MHz
WDW EM
SSB 0
LB 0.10 Hz
GB 0
PC 1.00
Br
O
OMe
N
OH
6.4 ppm
4.1 ppm
1.0 ppm
ppm

^{13}C{1H} and DEPT 90, 135 NMR of 3i

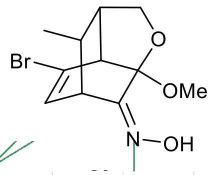

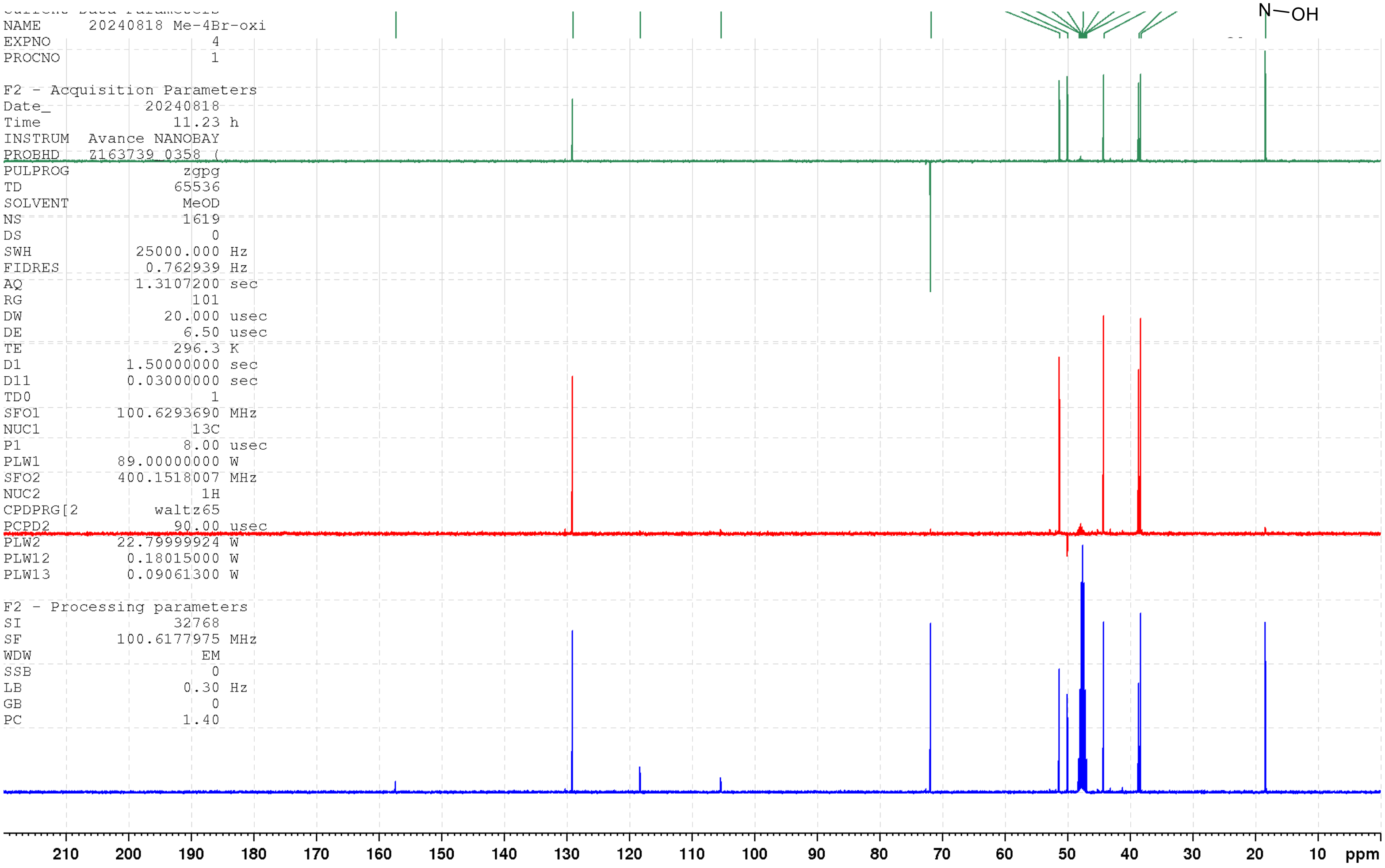

^{1}H NMR of 3j

```
Current Data Parameters
NAME        Oxime-Ph-4Br
EXPNO                  1
PROCNO                 1

F2 - Acquisition Parameters
Date_           20240523
Time               16.45
INSTRUM            spect
PROBHD   5 mm BBO BB-1H
PULPROG             zg30
TD                 32768
SOLVENT            CDCl3
NS                    16
DS                     0
SWH             6009.615 Hz
FIDRES          0.183399 Hz
AQ             2.7262976 sec
RG                   362
DW                83.200 usec
DE                  6.50 usec
TE                 295.2 K
D1            1.50000000 sec
TD0                    1

======== CHANNEL f1 ========
NUC1                  1H
P1                 14.00 usec
PL1                -1.00 dB
PL1W          7.55784369 W
SFO1         400.1326010 MHz

F2 - Processing parameters
SI                 32768
SF           400.1300100 MHz
WDW                   EM
SSB                    0
LB                     0 Hz
GB                     0
PC                  1.00
```

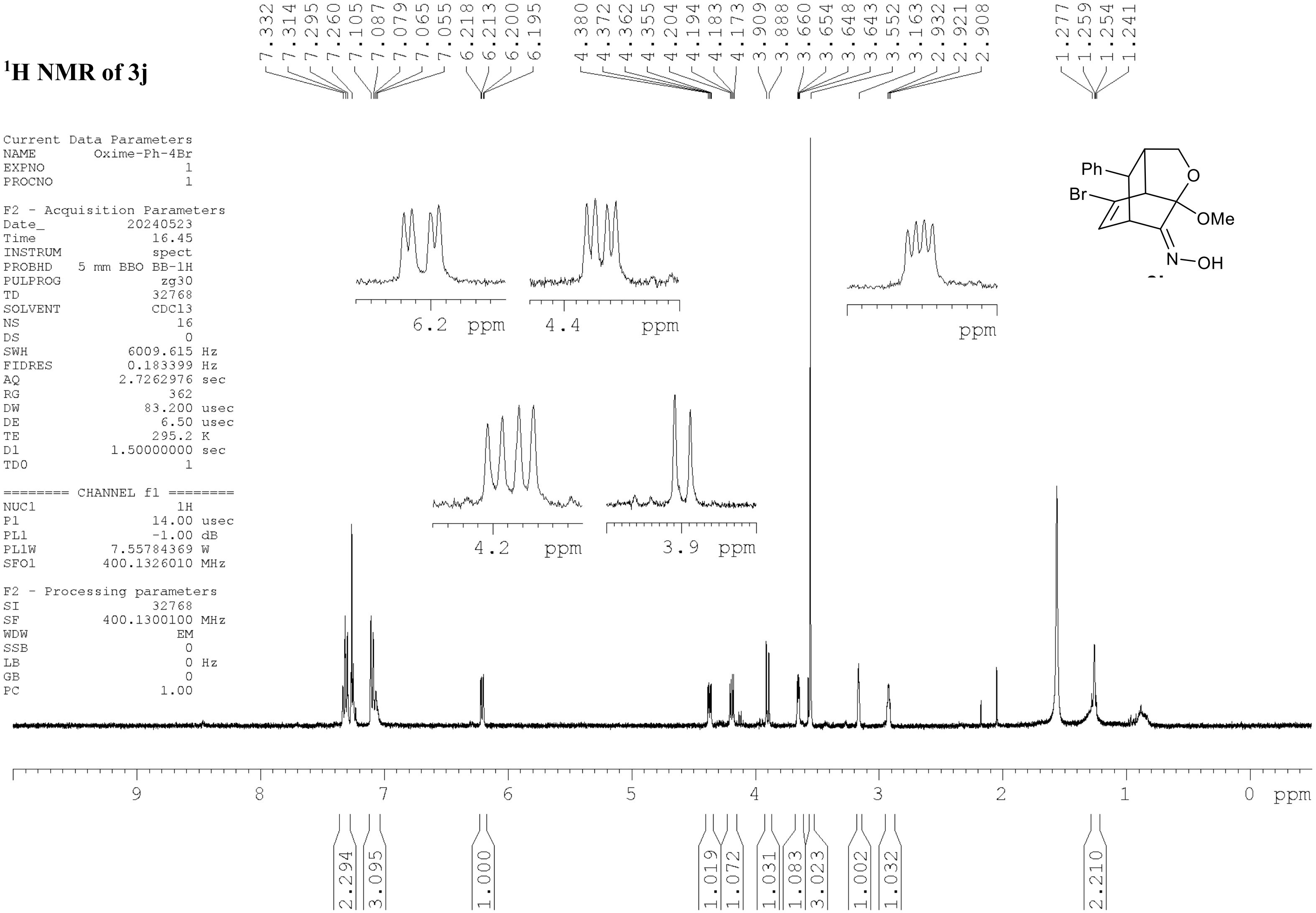

^{13}C{1H} and DEPT 90, 135 NMR of 3j

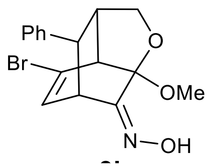

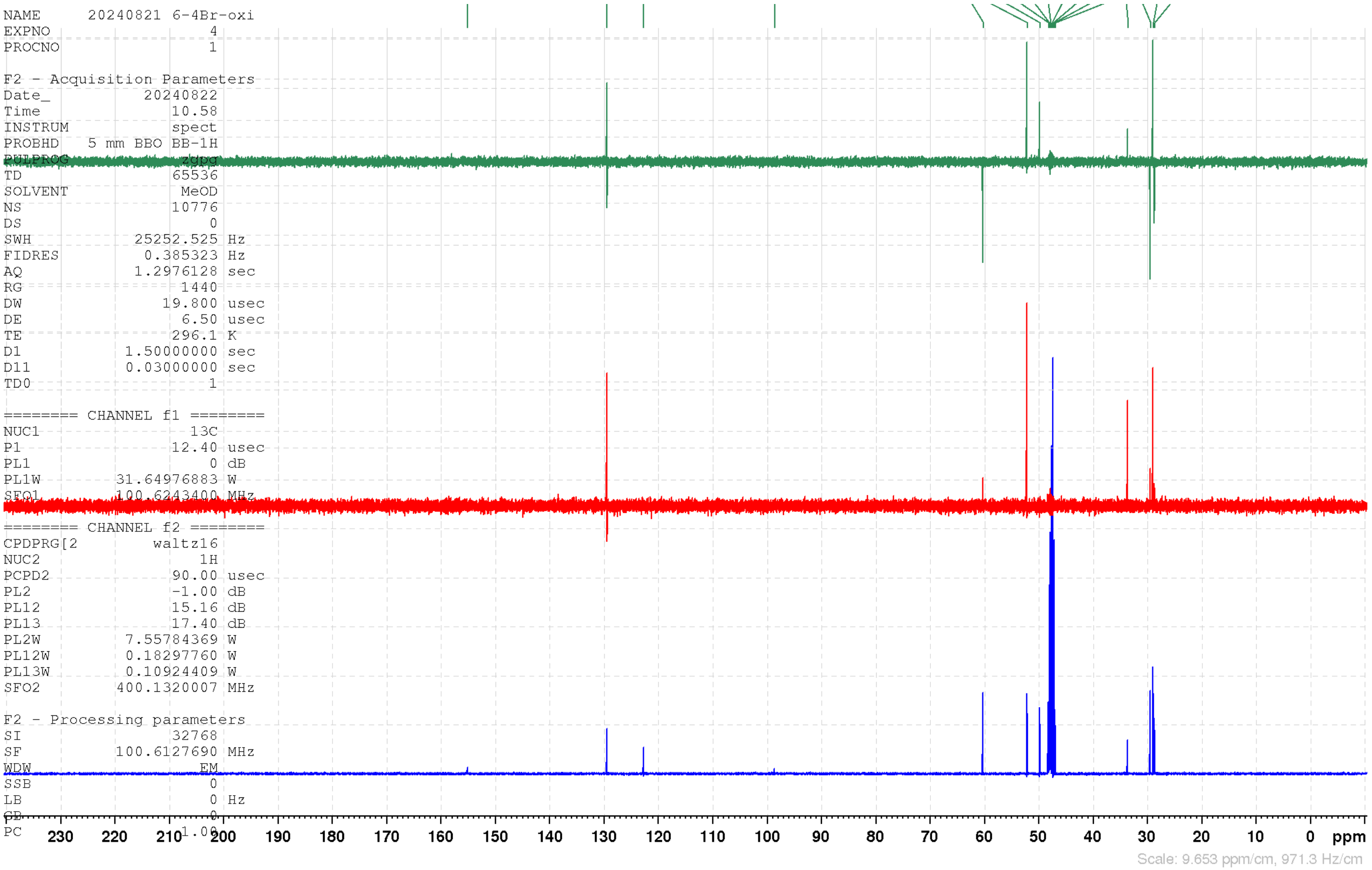

^{1}H NMR of 3k

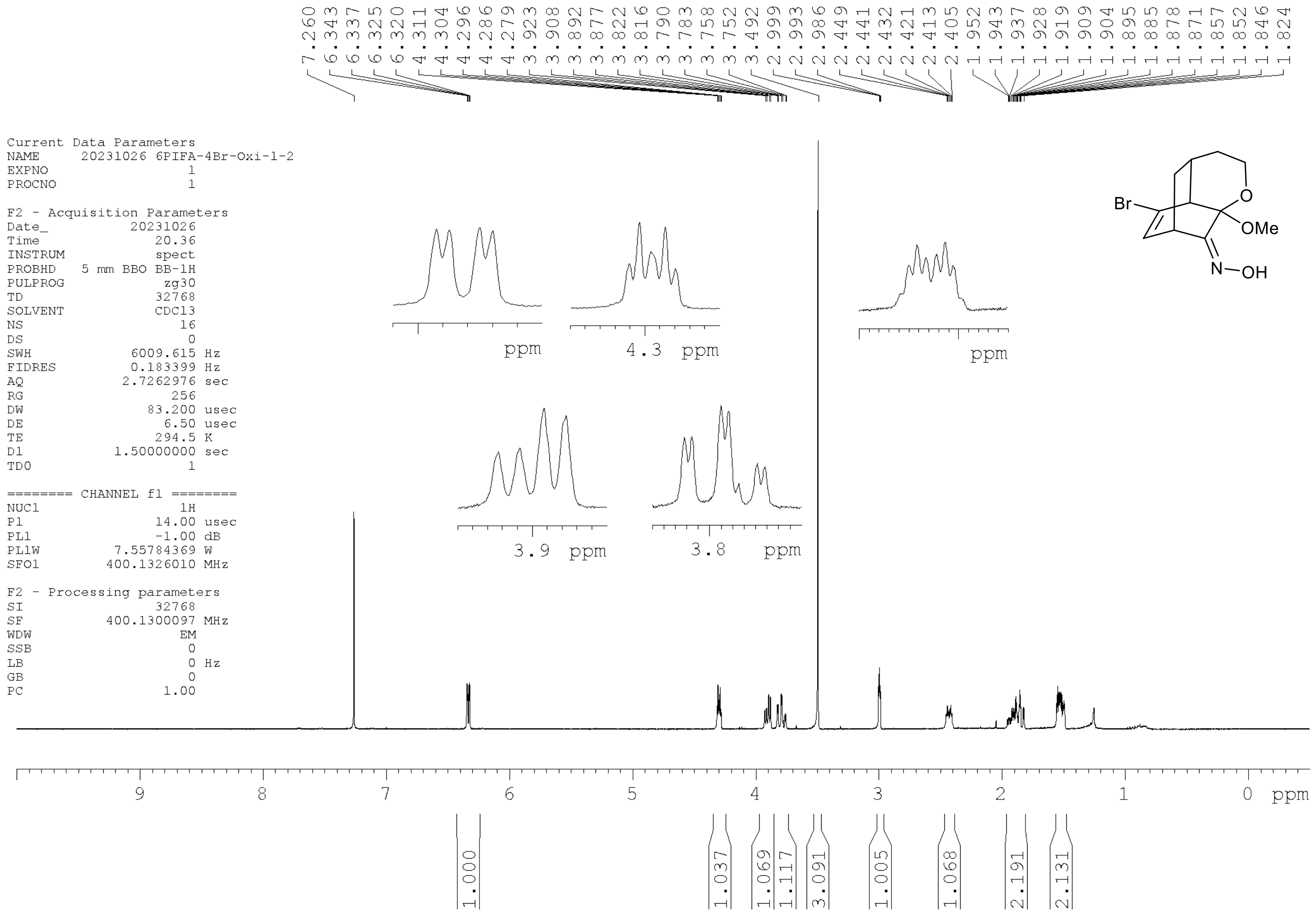

^{13}C{1H} and DEPT 90, 135 NMR of 3k

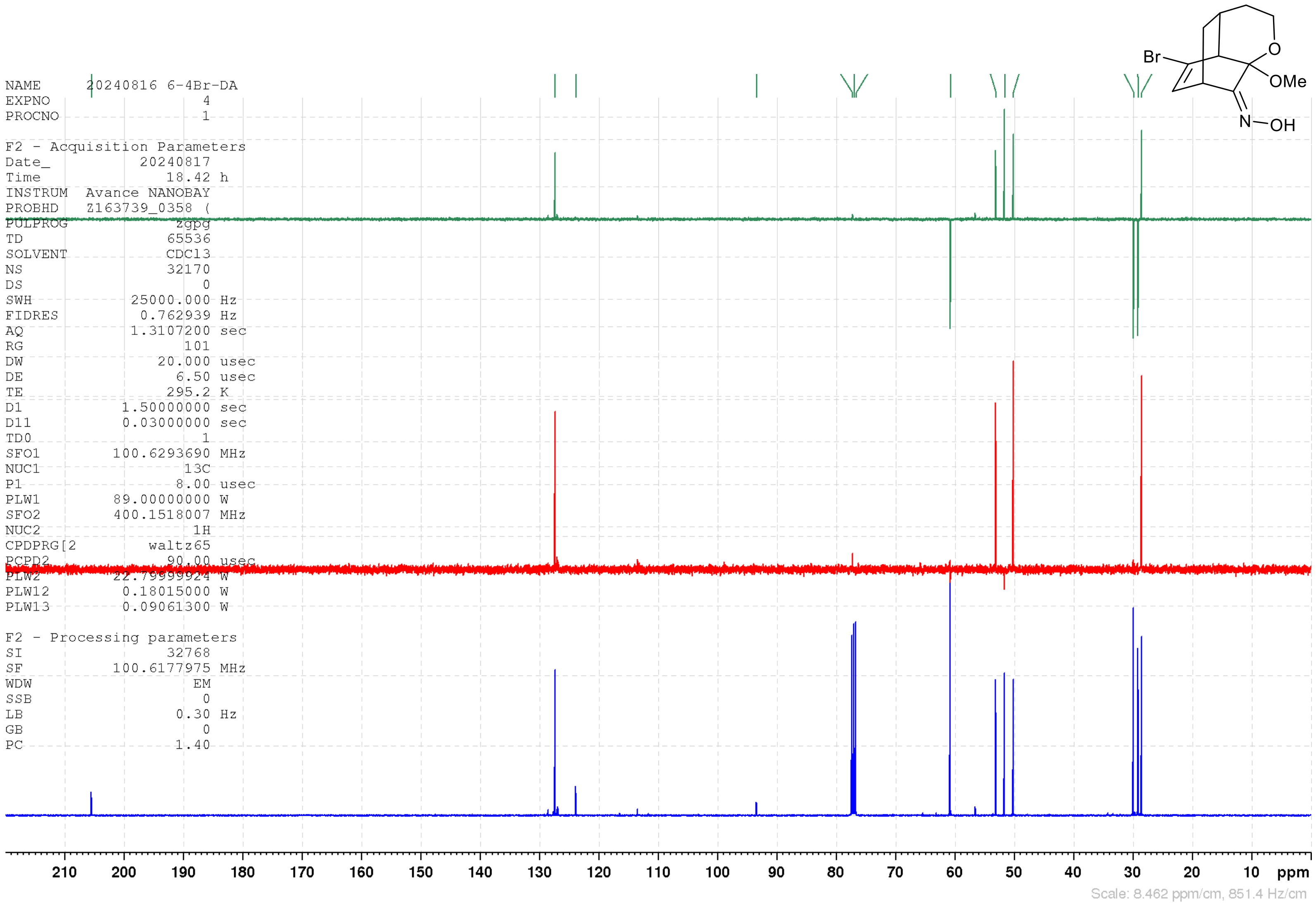

^{1}H NMR of 4a

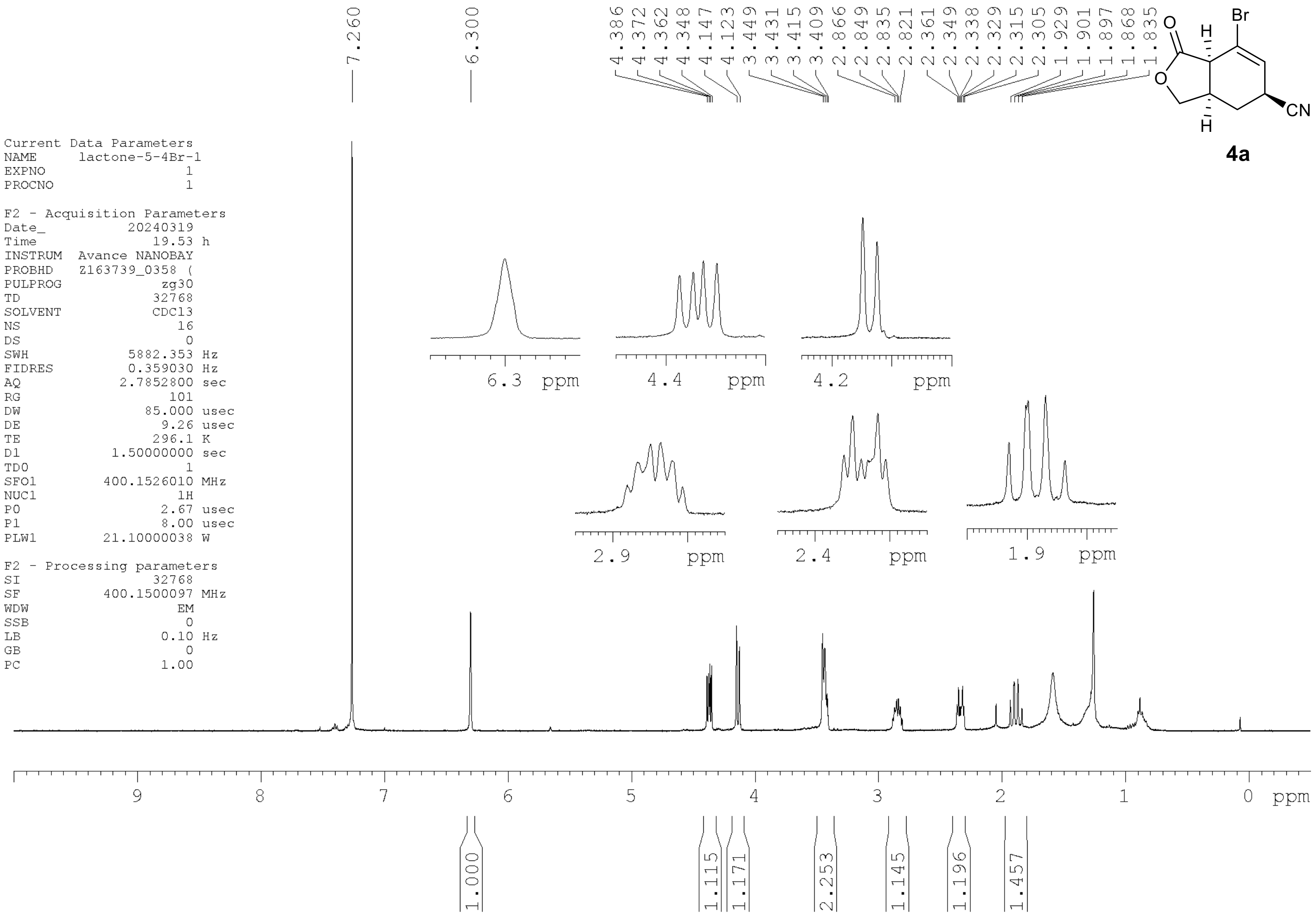

7.260
6.300
4.386 4.372 4.362 4.348 4.147 4.123 3.449 3.431 3.415 3.409 2.866 2.849 2.835 2.821 2.361 2.349 2.338 2.329 2.315 2.305 1.929 1.901 1.897 1.868 1.835
O
Br
H
O
CN
H
4a
Current Data Parameters
NAME lactone-5-4Br-1
EXPNO 1
PROCNO 1
F2 - Acquisition Parameters
Date_ 20240319
Time 19.53 h
INSTRUM Avance NANOBAY
PROBHD Z163739_0358 (
PULPROG zg30
TD 32768
SOLVENT CDCl3
NS 16
DS 0
SWH 5882.353 Hz
FIDRES 0.359030 Hz
AQ 2.7852800 sec
RG 101
DW 85.000 usec
DE 9.26 usec
TE 296.1 K
D1 1.50000000 sec
TD0 1
SFO1 400.1526010 MHz
NUC1 1H
P0 2.67 usec
P1 8.00 usec
PLW1 21.10000038 W
F2 - Processing parameters
SI 32768
SF 400.1500097 MHz
WDW EM
SSB 0
LB 0.10 Hz
GB 0
PC 1.00
6.3 ppm
4.4 ppm
4.2 ppm
2.9 ppm
2.4 ppm
1.9 ppm
9 8 7 6 5 4 3 2 1 0 ppm
1.000
1.115
1.171
2.253
1.145
1.196
1.457

^{13}C{1H} and DEPT 90, 135 NMR of 4a

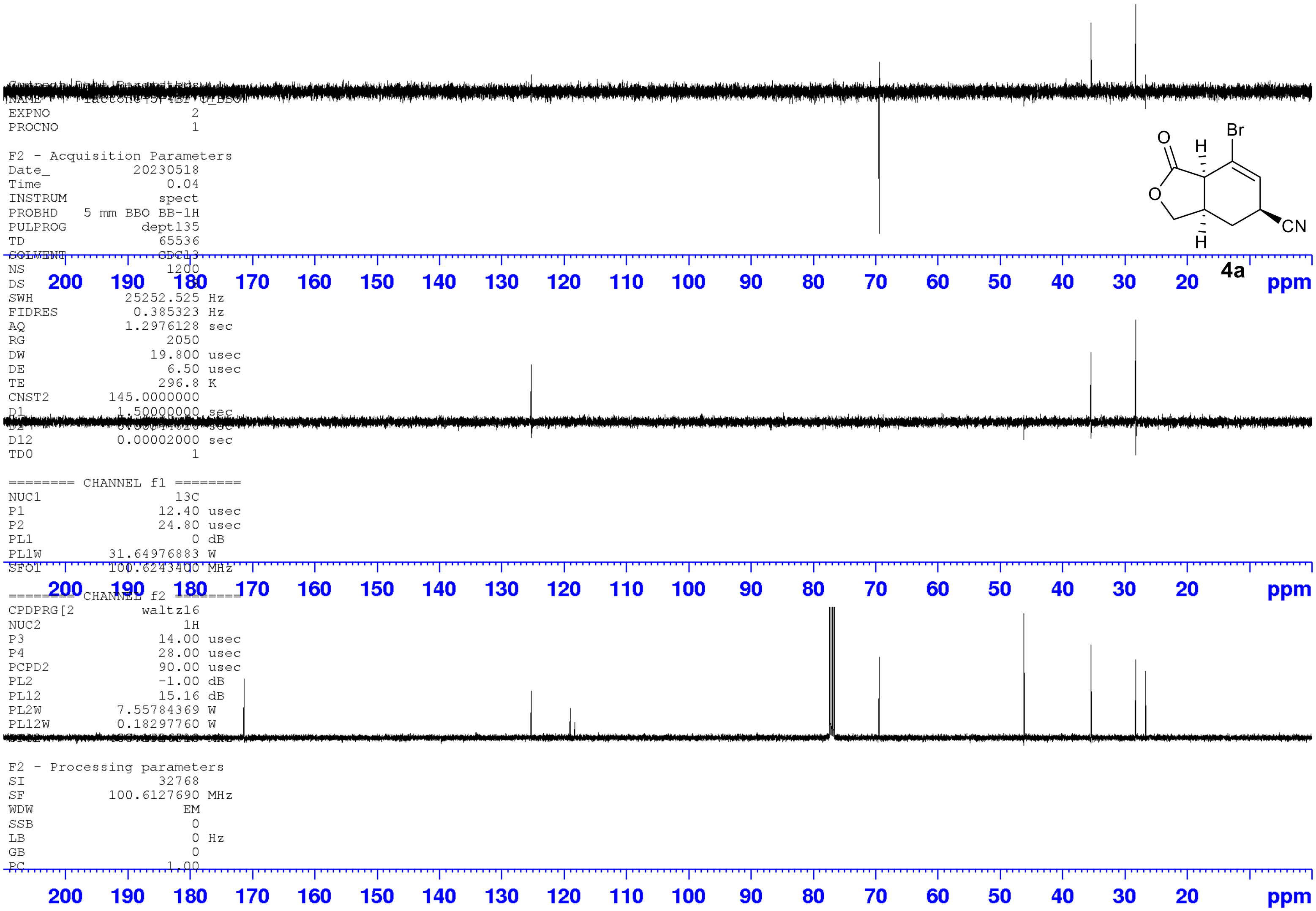

^{1}H NMR of 4b

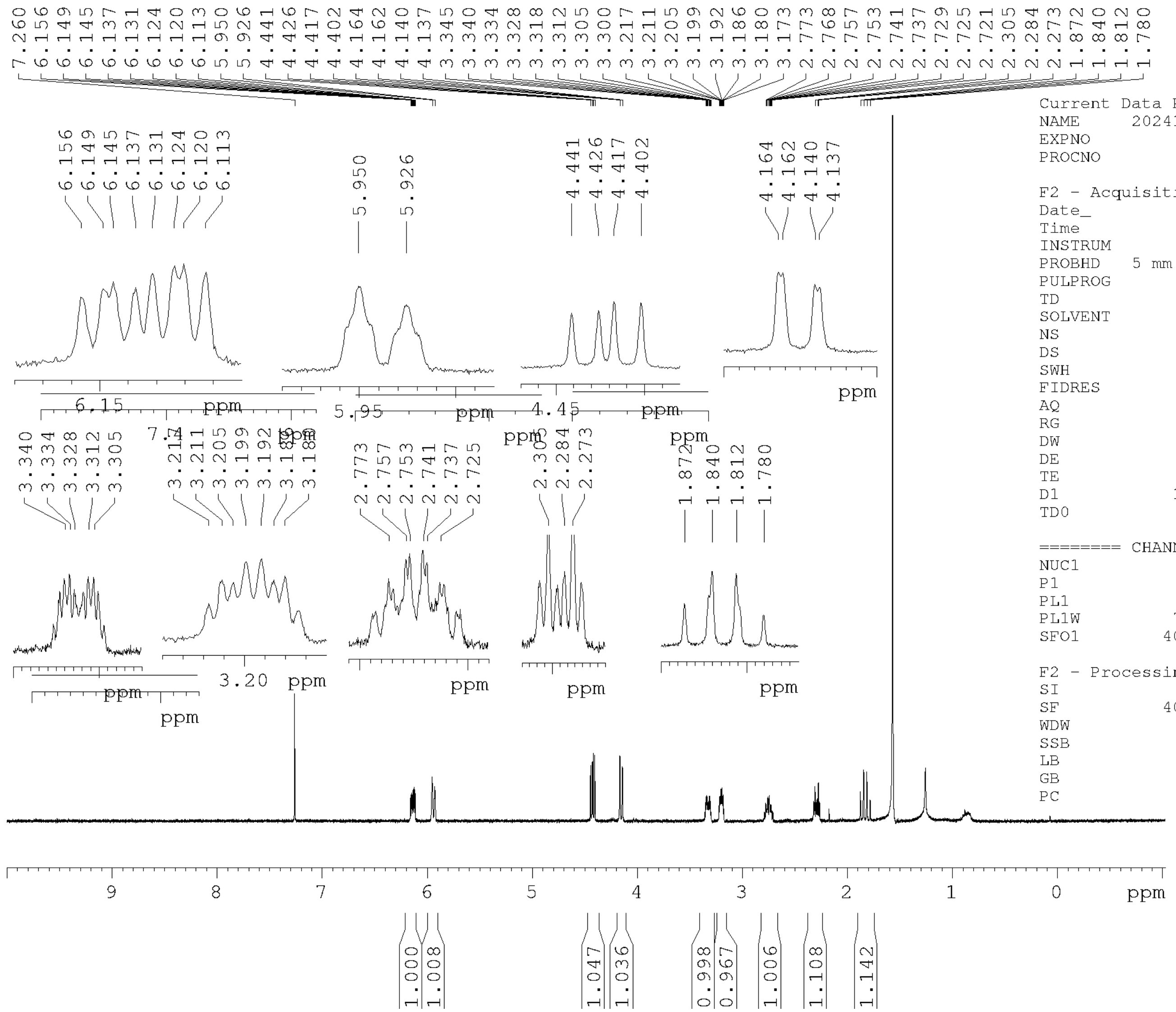

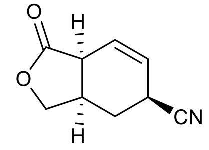

4b

^{13}C{1H} and DEPT 90, 135 NMR of 4b

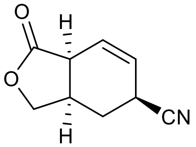

4b

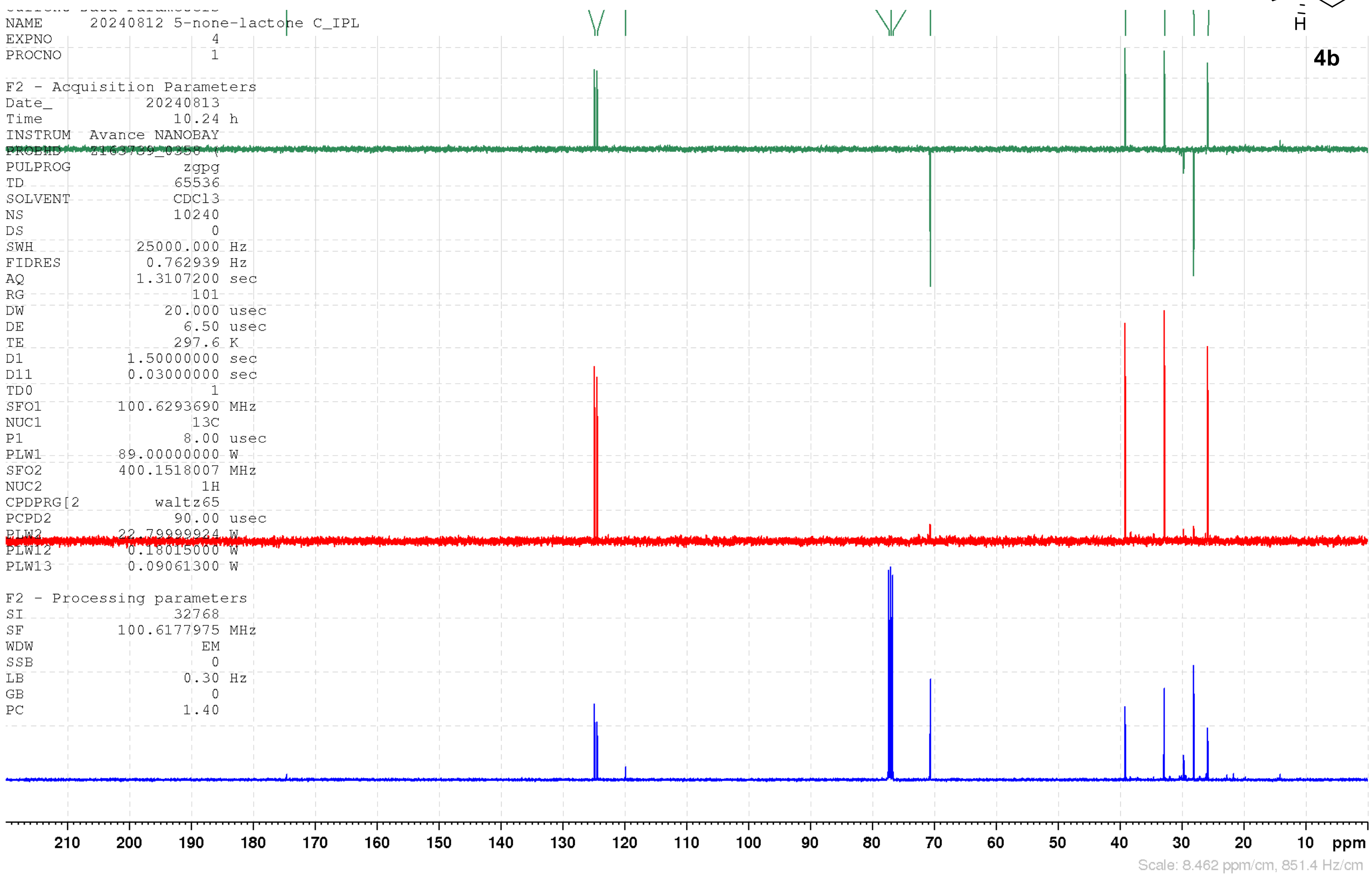

^{1}H NMR of 4c

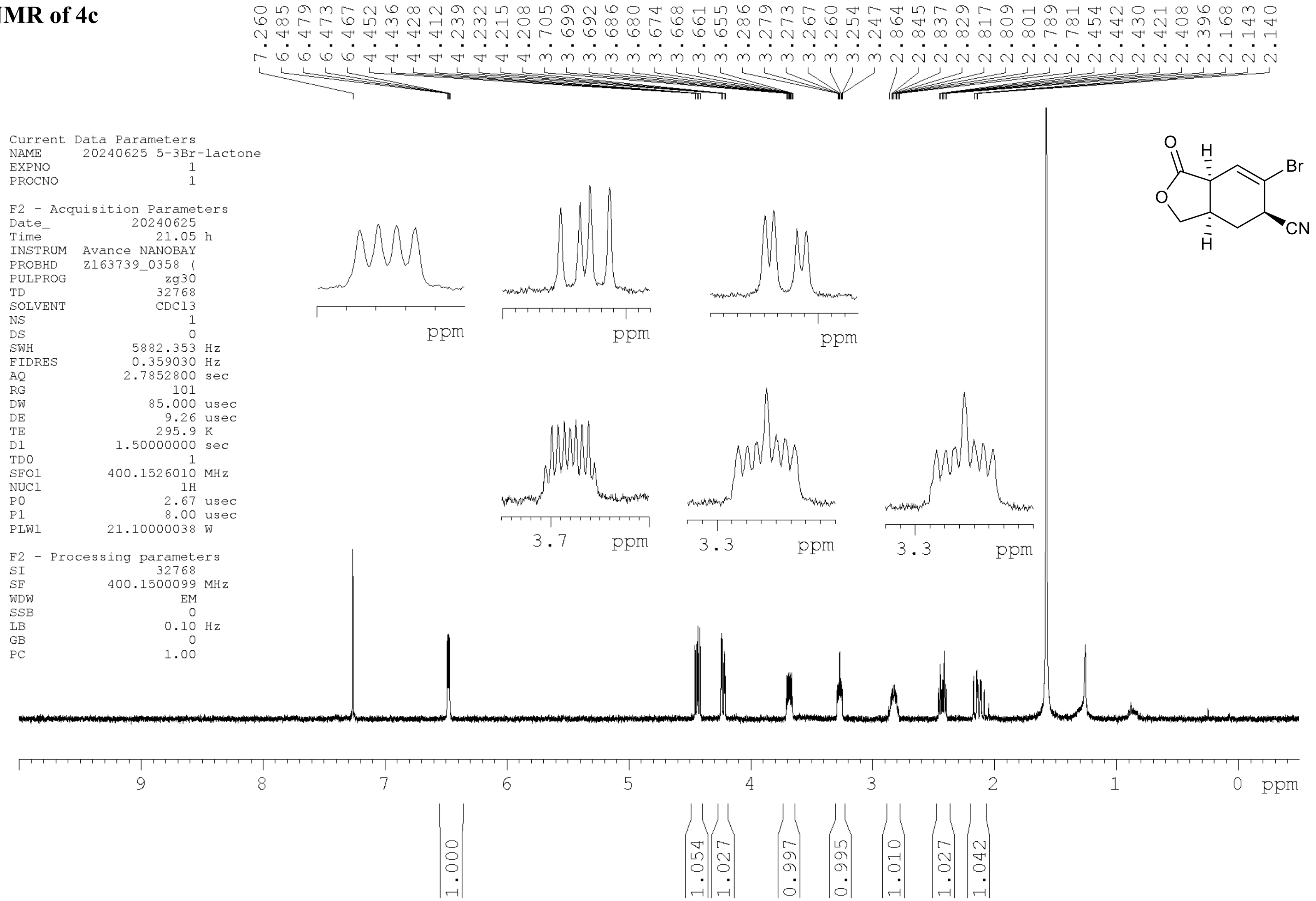
7.260
6.485
6.479
6.473
6.467
4.452
4.436
4.428
4.412
4.239
4.232
4.215
4.208
3.705
3.699
3.692
3.686
3.680
3.674
3.668
3.661
3.655
3.286
3.279
3.273
3.267
3.260
3.254
3.247
2.864
2.845
2.837
2.829
2.817
2.809
2.801
2.789
2.781
2.454
2.442
2.430
2.421
2.408
2.396
2.168
2.143
2.140
Current Data Parameters
NAME 20240625 5-3Br-lactone
EXPNO 1
PROCNO 1
F2 - Acquisition Parameters
Date_ 20240625
Time 21.05 h
INSTRUM Avance NANOBAY
PROBHD Z163739_0358 (
PULPROG zg30
TD 32768
SOLVENT CDCl3
NS 1
DS 0
SWH 5882.353 Hz
FIDRES 0.359030 Hz
AQ 2.7852800 sec
RG 101
DW 85.000 usec
DE 9.26 usec
TE 295.9 K
D1 1.50000000 sec
TD0 1
SFO1 400.1526010 MHz
NUC1 1H
P0 2.67 usec
P1 8.00 usec
PLW1 21.10000038 W
F2 - Processing parameters
SI 32768
SF 400.1500099 MHz
WDW EM
SSB 0
LB 0.10 Hz
GB 0
PC 1.00
O
H
Br
O
CN
H
ppm
ppm
ppm
3.7 ppm
3.3 ppm
3.3 ppm
9 8 7 6 5 4 3 2 1 0 ppm
1.000
1.054
1.027
0.997
0.995
1.010
1.027
1.042

^{13}C{1H} and DEPT 90, 135 NMR of 4c

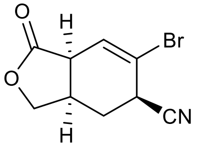

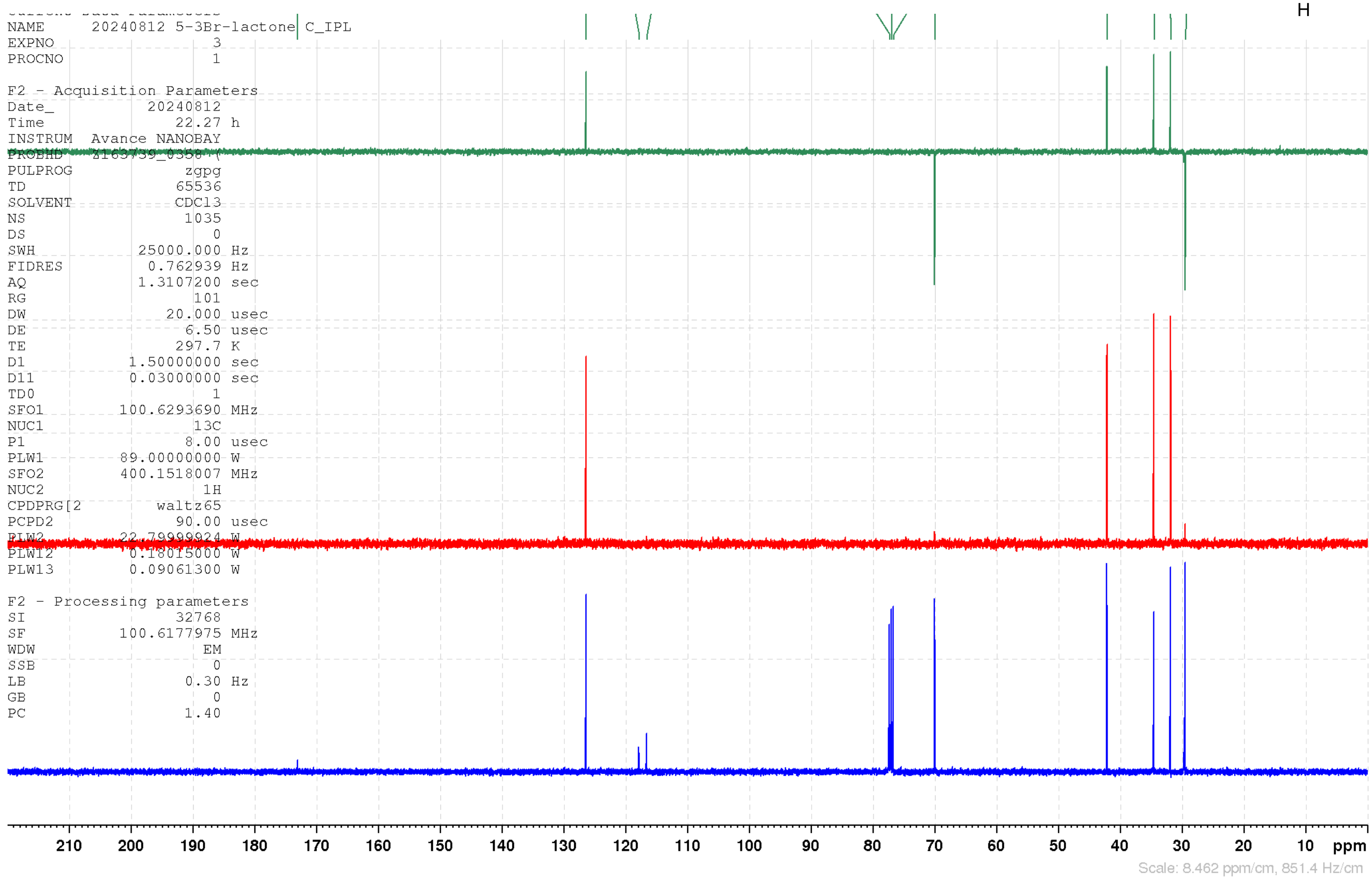

^{1}H NMR of 4d

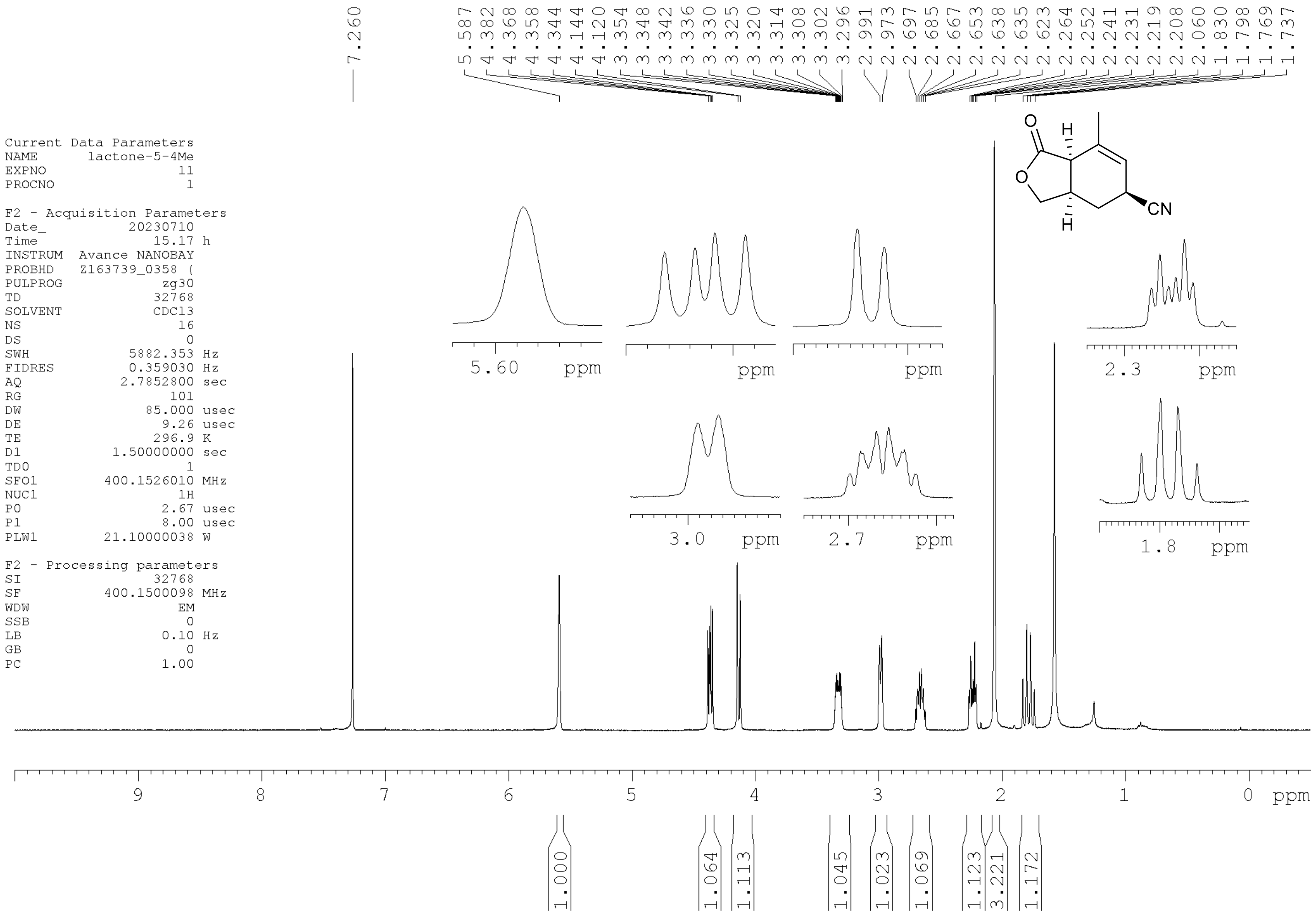

7.260
5.587 4.382 4.368 4.358 4.344 4.144 4.120 3.354 3.348 3.342 3.336 3.330 3.325 3.320 3.314 3.308 3.302 3.296 2.991 2.973 2.697 2.685 2.667 2.653 2.638 2.635 2.623 2.264 2.252 2.241 2.231 2.219 2.208 2.060 1.830 1.798 1.769 1.737
Current Data Parameters
NAME lactone-5-4Me
EXPNO 11
PROCNO 1
F2 - Acquisition Parameters
Date_ 20230710
Time 15.17 h
INSTRUM Avance NANOBAY
PROBHD Z163739_0358 (
PULPROG zg30
TD 32768
SOLVENT CDCl3
NS 16
DS 0
SWH 5882.353 Hz
FIDRES 0.359030 Hz
AQ 2.7852800 sec
RG 101
DW 85.000 usec
DE 9.26 usec
TE 296.9 K
D1 1.50000000 sec
TD0 1
SFO1 400.1526010 MHz
NUC1 1H
P0 2.67 usec
P1 8.00 usec
PLW1 21.10000038 W
F2 - Processing parameters
SI 32768
SF 400.1500098 MHz
WDW EM
SSB 0
LB 0.10 Hz
GB 0
PC 1.00
5.60 ppm
ppm
ppm
2.3 ppm
3.0 ppm
2.7 ppm
1.8 ppm
O
O
H
H
CN
9 8 7 6 5 4 3 2 1 0 ppm
1.000 1.064 1.113 1.045 1.023 1.069 1.123 3.221 1.172

^{13}C{1H} and DEPT 90, 135 NMR of 4d

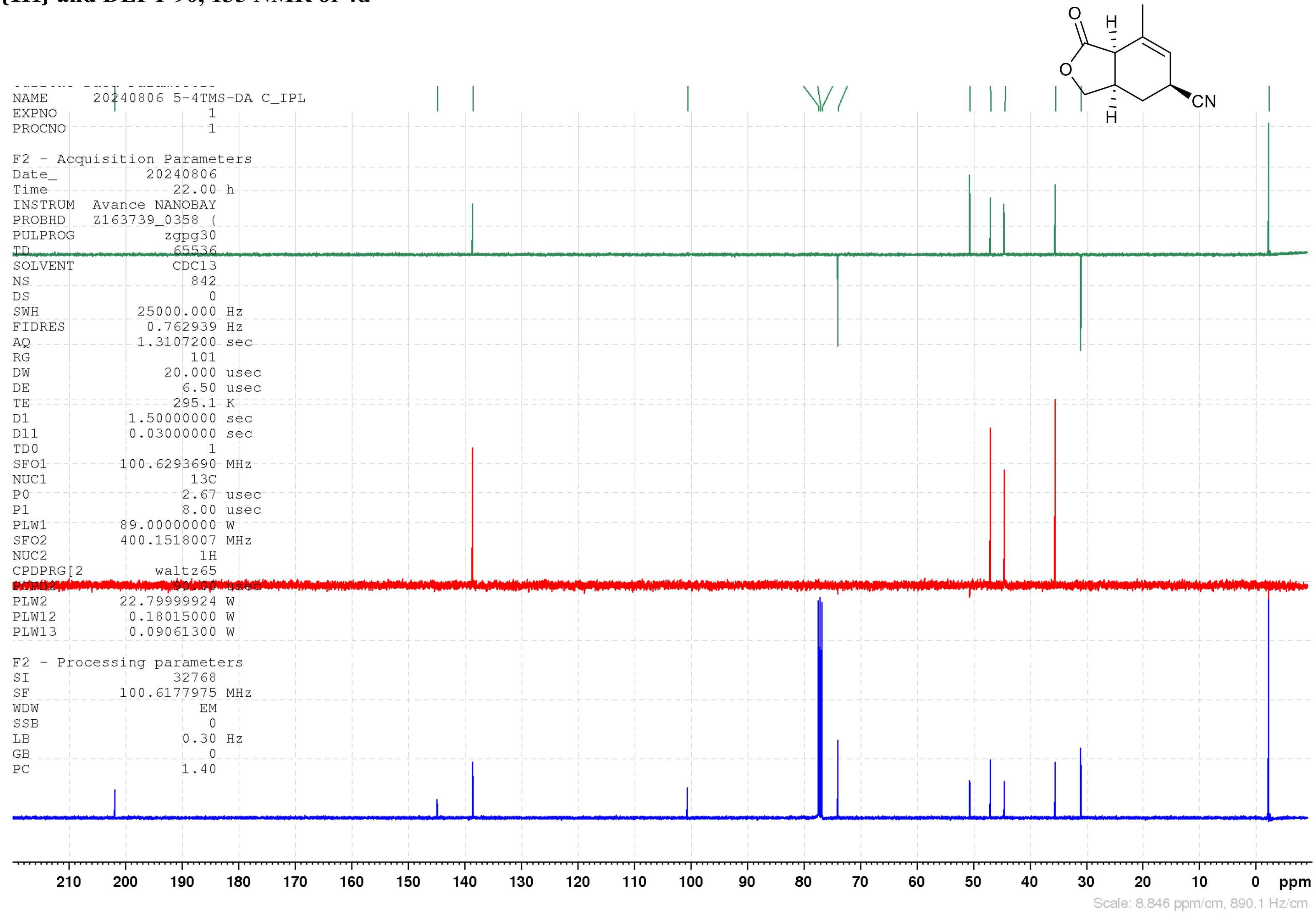

^{1}H NMR of 4e

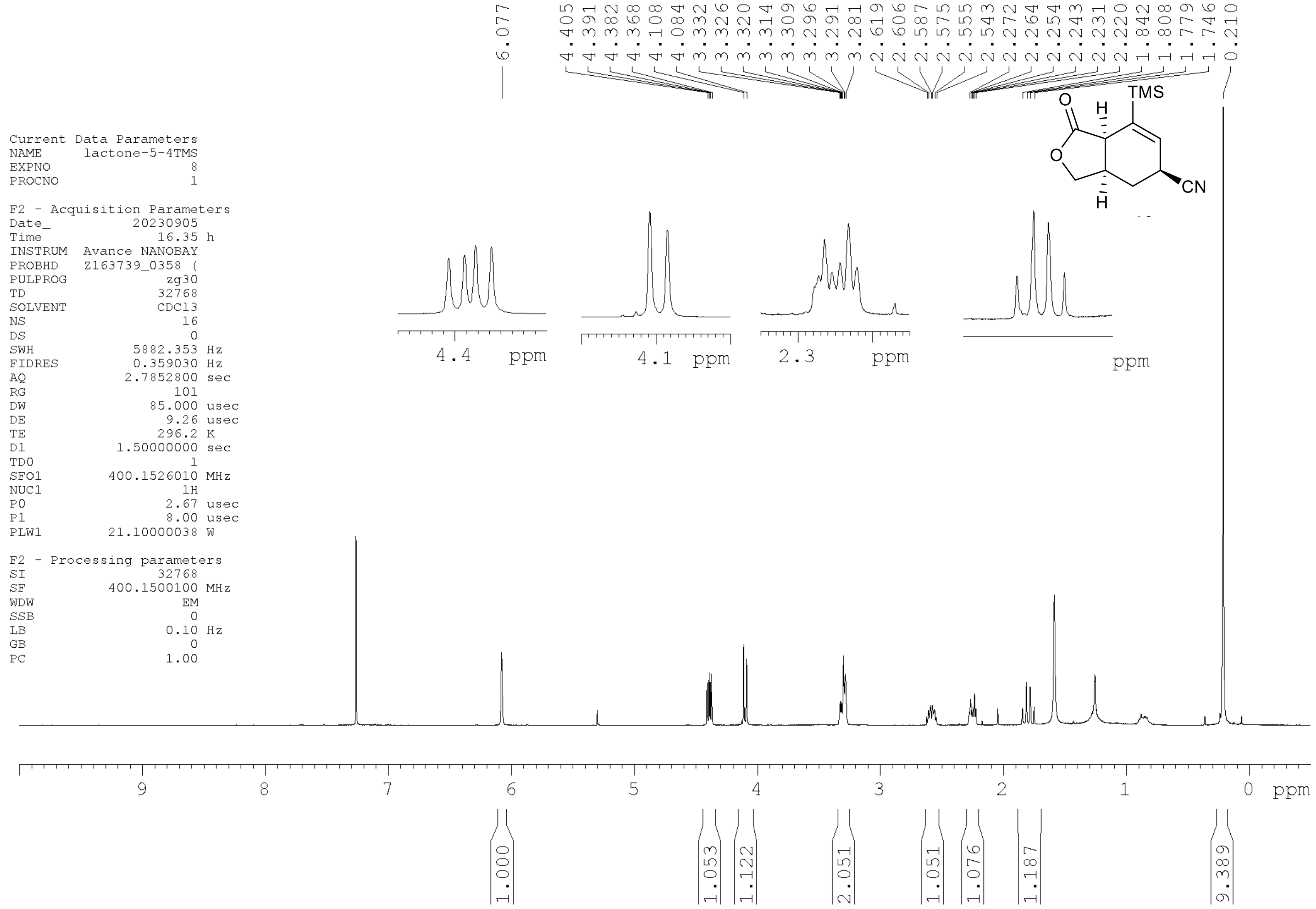
6.077
4.405
4.391
4.382
4.368
4.108
4.084
3.332
3.326
3.320
3.314
3.309
3.296
3.291
3.281
2.619
2.606
2.587
2.575
2.555
2.543
2.272
2.264
2.254
2.243
2.231
2.220
1.842
1.808
1.779
1.746
0.210
TMS
O
H
O
H
CN
Current Data Parameters
NAME lactone-5-4TMS
EXPNO 8
PROCNO 1
F2 - Acquisition Parameters
Date_ 20230905
Time 16.35 h
INSTRUM Avance NANOBAY
PROBHD Z163739_0358 (
PULPROG zg30
TD 32768
SOLVENT CDCl3
NS 16
DS 0
SWH 5882.353 Hz
FIDRES 0.359030 Hz
AQ 2.7852800 sec
RG 101
DW 85.000 usec
DE 9.26 usec
TE 296.2 K
D1 1.50000000 sec
TD0 1
SFO1 400.1526010 MHz
NUC1 1H
P0 2.67 usec
P1 8.00 usec
PLW1 21.10000038 W
F2 - Processing parameters
SI 32768
SF 400.1500100 MHz
WDW EM
SSB 0
LB 0.10 Hz
GB 0
PC 1.00
4.4 ppm
4.1 ppm
2.3 ppm
ppm
9
8
7
6
5
4
3
2
1
0 ppm
1.000
1.053
1.122
2.051
1.051
1.076
1.187
9.389

^{13}C{1H} and DEPT 90, 135 NMR of 4e

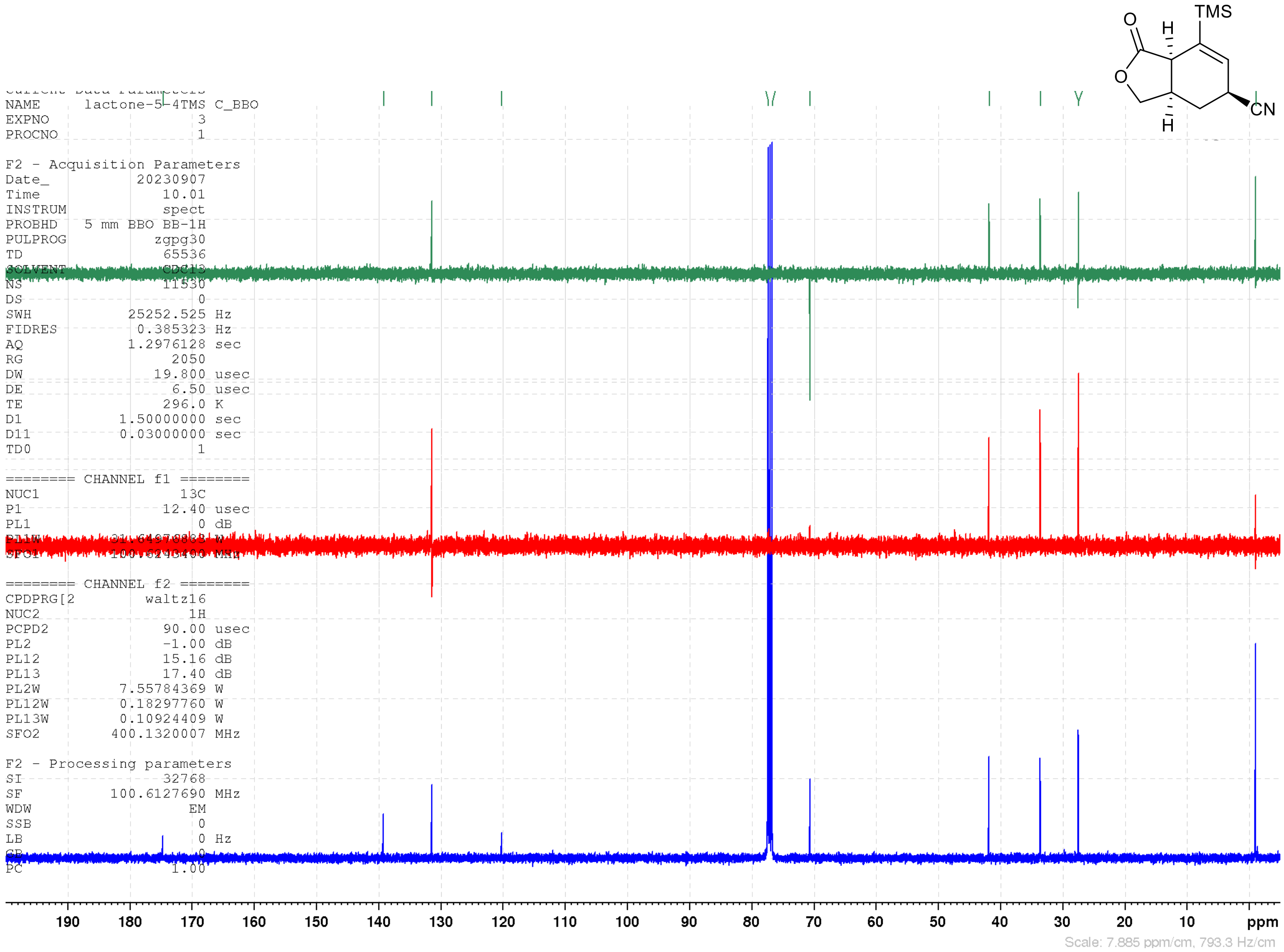

^{1}H NMR of 4f

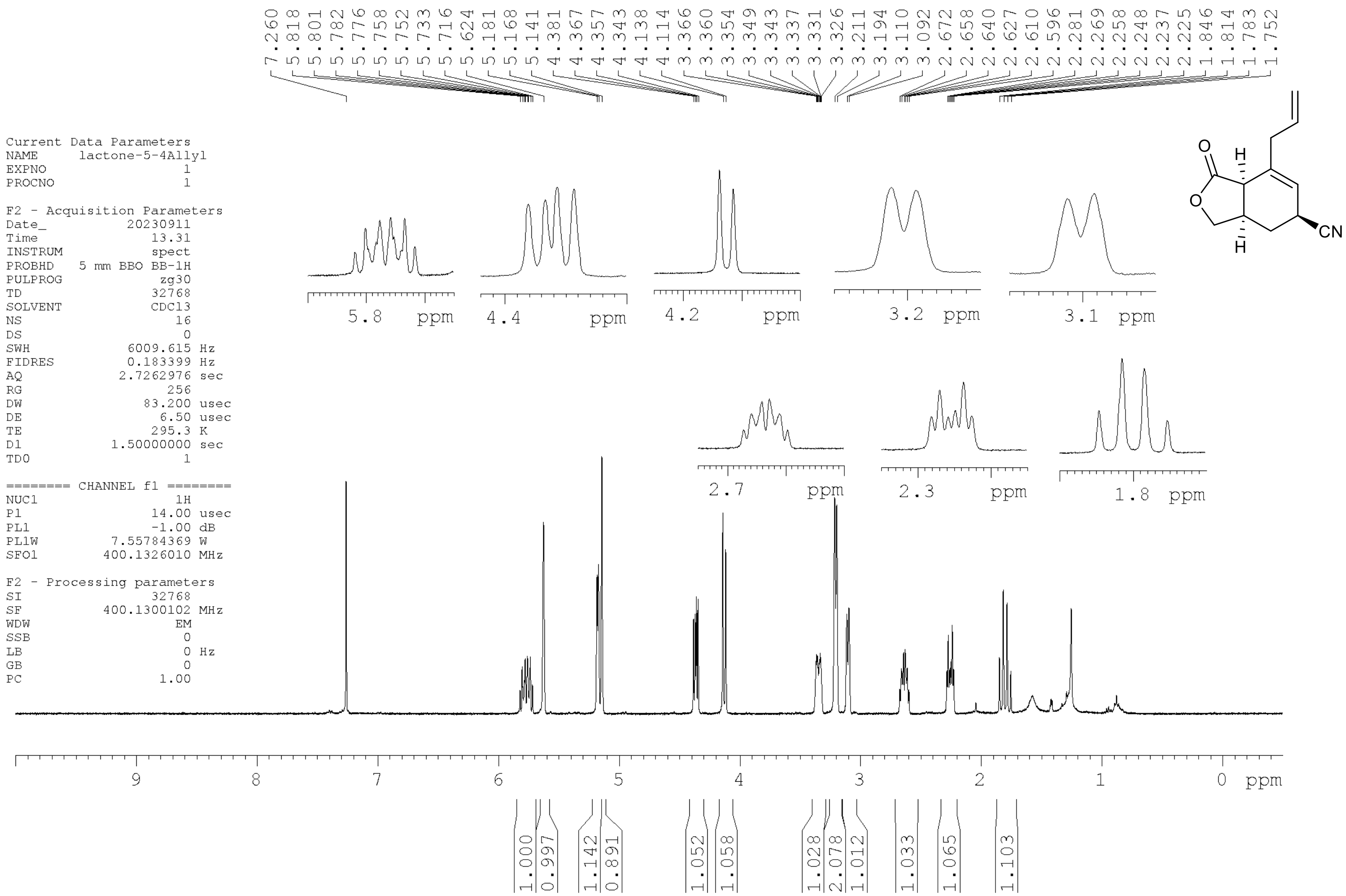

^{13}C{1H} and DEPT 90, 135 NMR of 4f

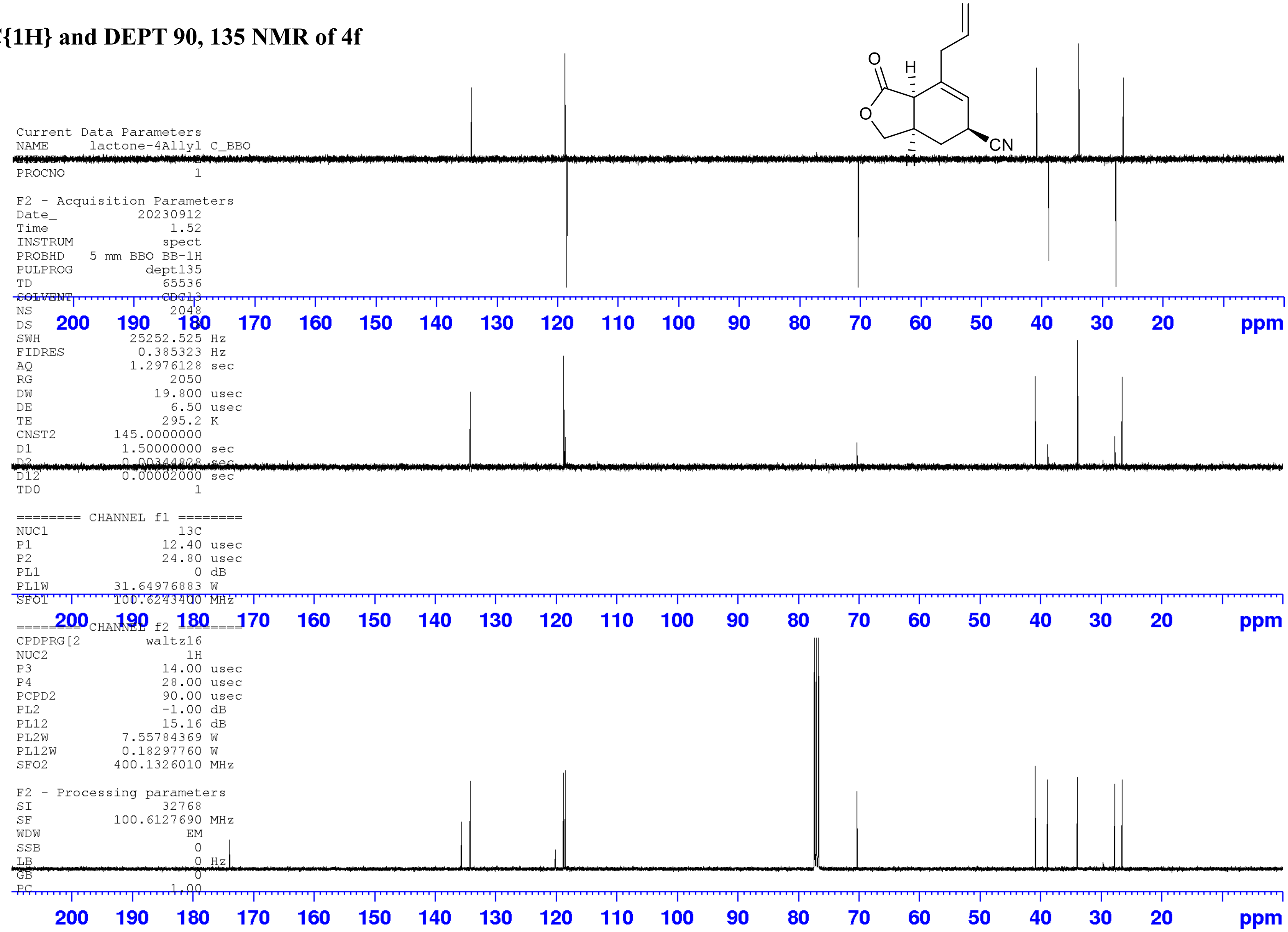

^{1}H NMR of 4g

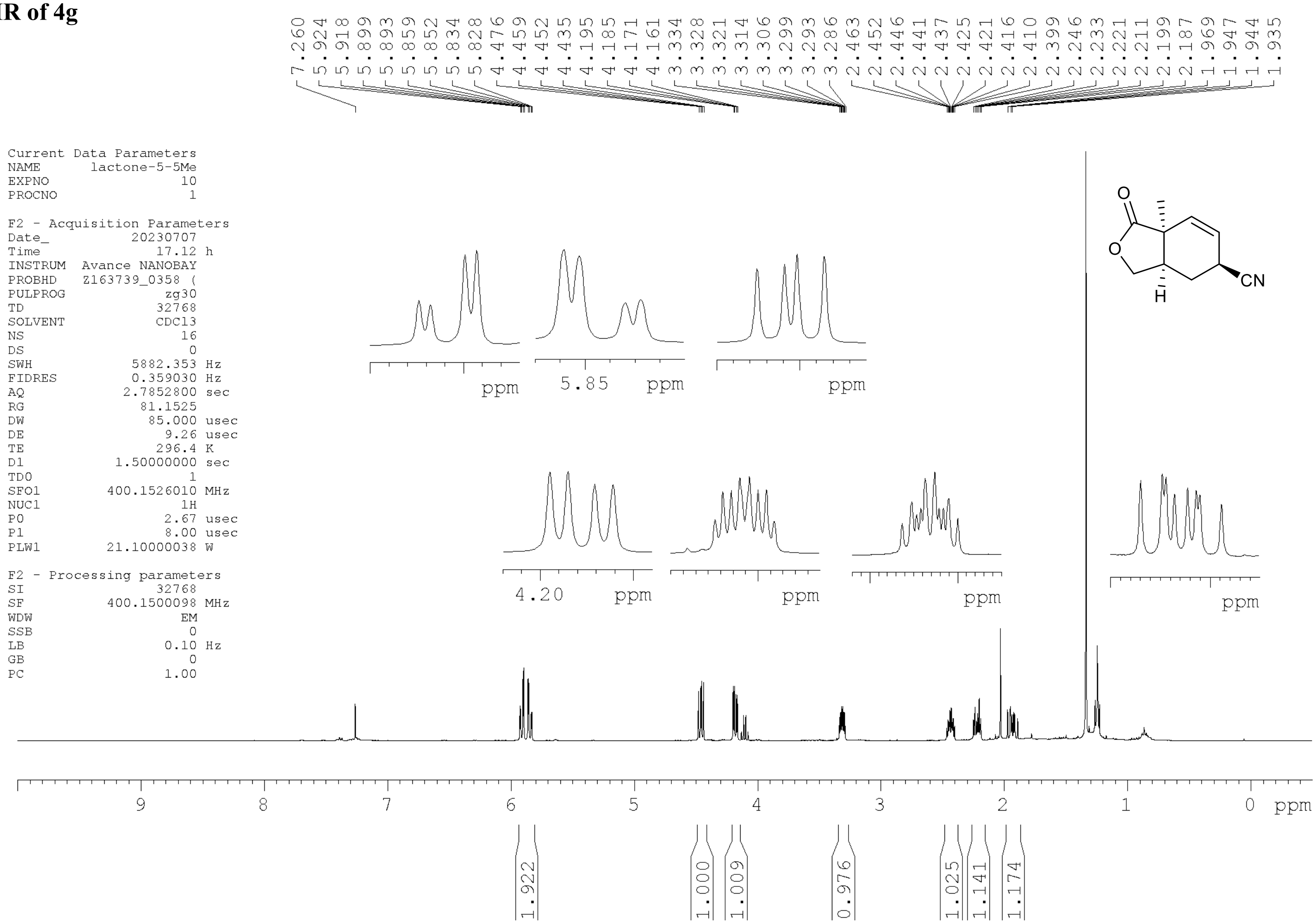
7.260
5.924
5.918
5.899
5.893
5.859
5.852
5.834
5.828
4.476
4.459
4.452
4.435
4.195
4.185
4.171
4.161
3.334
3.328
3.321
3.314
3.306
3.299
3.293
3.286
2.463
2.452
2.446
2.441
2.437
2.425
2.421
2.416
2.410
2.399
2.246
2.233
2.221
2.211
2.199
2.187
1.969
1.947
1.944
1.935
Current Data Parameters
NAME lactone-5-5Me
EXPNO 10
PROCNO 1
F2 - Acquisition Parameters
Date_ 20230707
Time 17.12 h
INSTRUM Avance NANOBAY
PROBHD Z163739_0358 (
PULPROG zg30
TD 32768
SOLVENT CDCl3
NS 16
DS 0
SWH 5882.353 Hz
FIDRES 0.359030 Hz
AQ 2.7852800 sec
RG 81.1525
DW 85.000 usec
DE 9.26 usec
TE 296.4 K
D1 1.50000000 sec
TD0 1
SFO1 400.1526010 MHz
NUC1 1H
P0 2.67 usec
P1 8.00 usec
PLW1 21.10000038 W
F2 - Processing parameters
SI 32768
SF 400.1500098 MHz
WDW EM
SSB 0
LB 0.10 Hz
GB 0
PC 1.00
O
O
H
CN
5.85 ppm
4.20 ppm
ppm
9
8
7
6
5
4
3
2
1
0 ppm
1.922
1.000
1.009
0.976
1.025
1.141
1.174

^{13}C{1H} and DEPT 90, 135 NMR of 4g

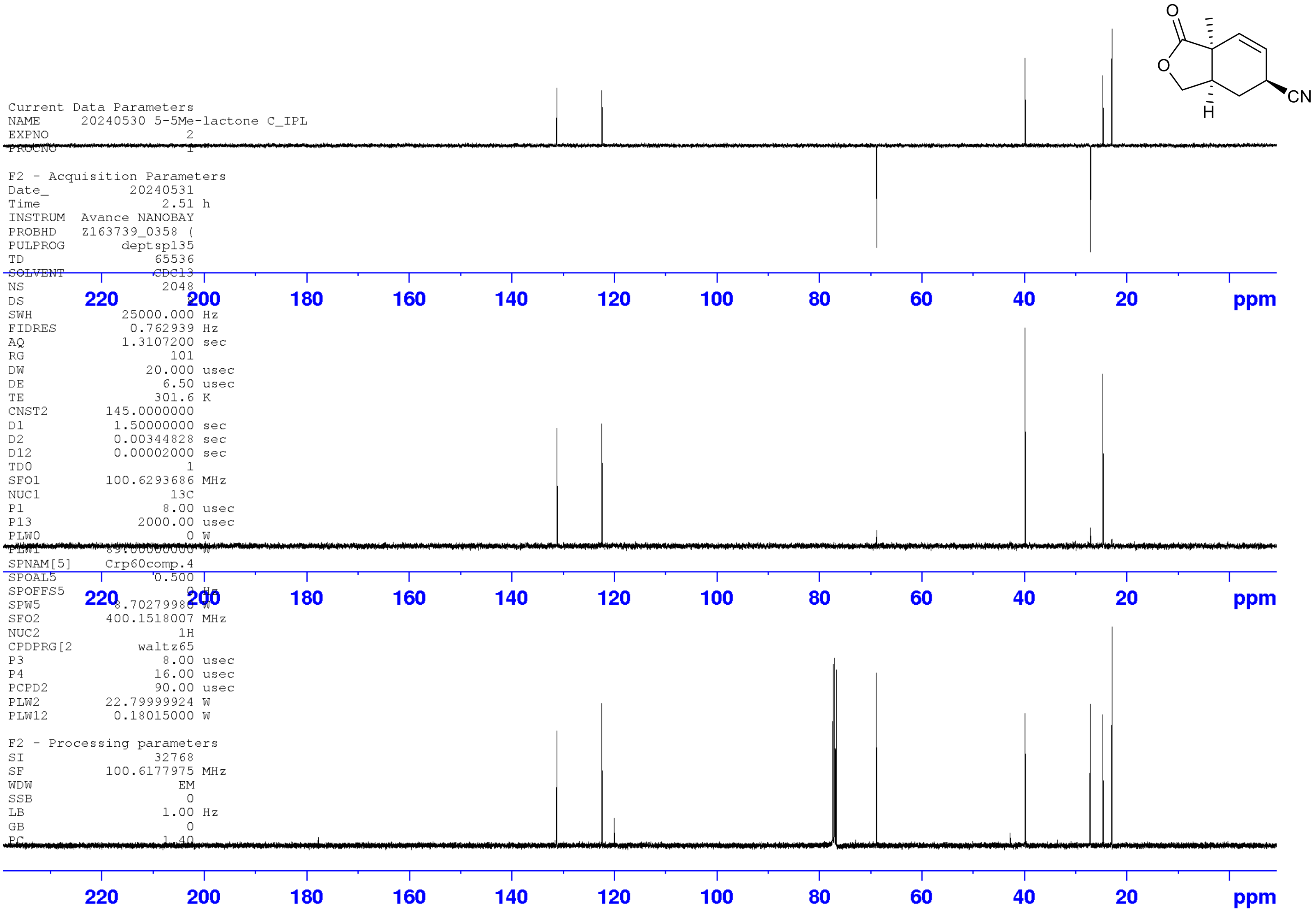

^{1}H NMR of 4h

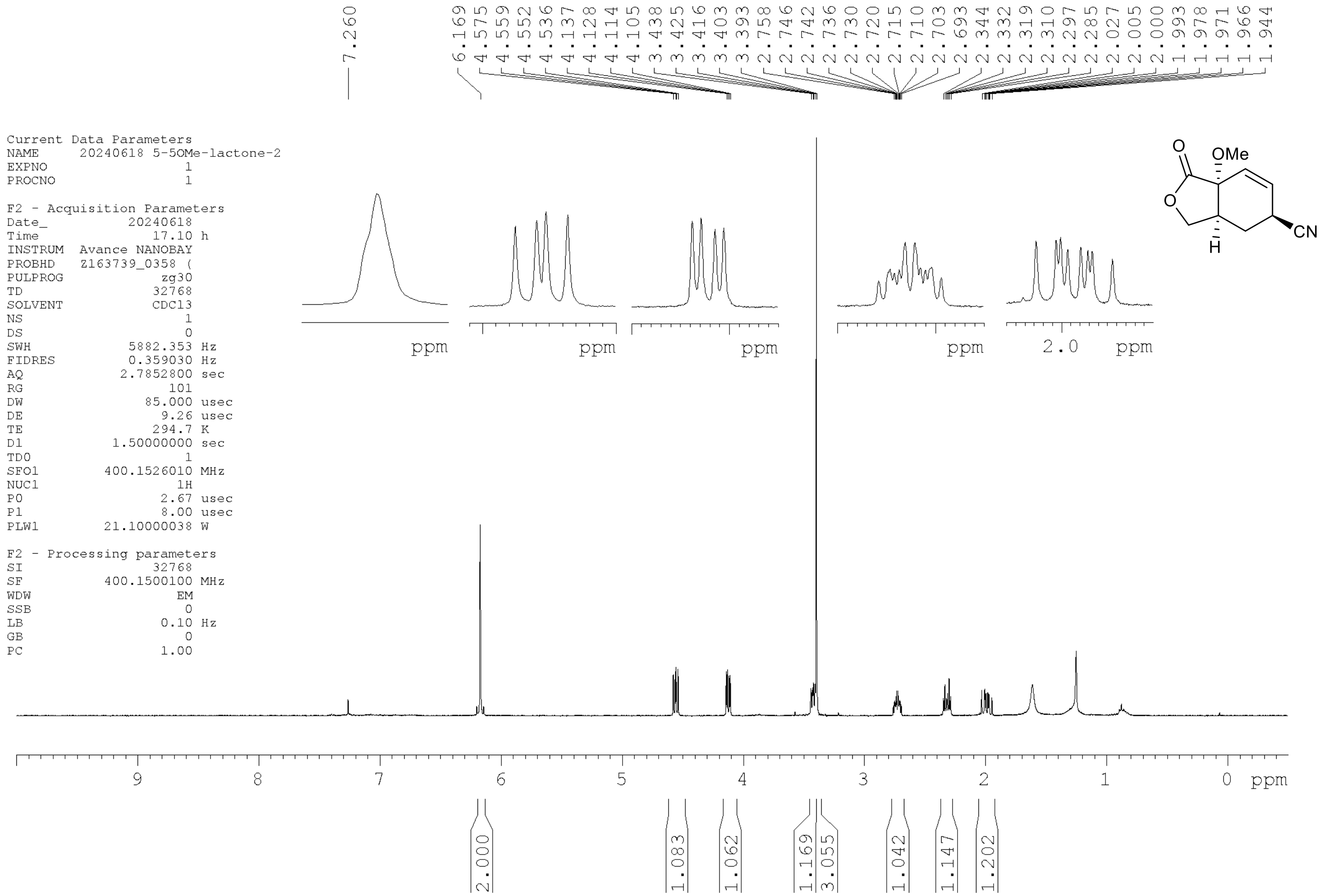

7.260
6.169
4.575
4.559
4.552
4.536
4.137
4.128
4.114
4.105
3.438
3.425
3.416
3.403
3.393
2.758
2.746
2.742
2.736
2.730
2.720
2.715
2.710
2.703
2.693
2.344
2.332
2.319
2.310
2.297
2.285
2.027
2.005
2.000
1.993
1.978
1.971
1.966
1.944
Current Data Parameters
NAME 20240618 5-5OMe-lactone-2
EXPNO 1
PROCNO 1
F2 - Acquisition Parameters
Date_ 20240618
Time 17.10 h
INSTRUM Avance NANOBAY
PROBHD Z163739_0358 (
PULPROG zg30
TD 32768
SOLVENT CDCl3
NS 1
DS 0
SWH 5882.353 Hz
FIDRES 0.359030 Hz
AQ 2.7852800 sec
RG 101
DW 85.000 usec
DE 9.26 usec
TE 294.7 K
D1 1.50000000 sec
TD0 1
SFO1 400.1526010 MHz
NUC1 1H
P0 2.67 usec
P1 8.00 usec
PLW1 21.10000038 W
F2 - Processing parameters
SI 32768
SF 400.1500100 MHz
WDW EM
SSB 0
LB 0.10 Hz
GB 0
PC 1.00
OMe
O
O
H
CN
ppm
2.0
9
8
7
6
5
4
3
2
1
0
2.000
1.083
1.062
1.169
3.055
1.042
1.147
1.202

^{13}C{1H} and DEPT 90, 135 NMR of 4h

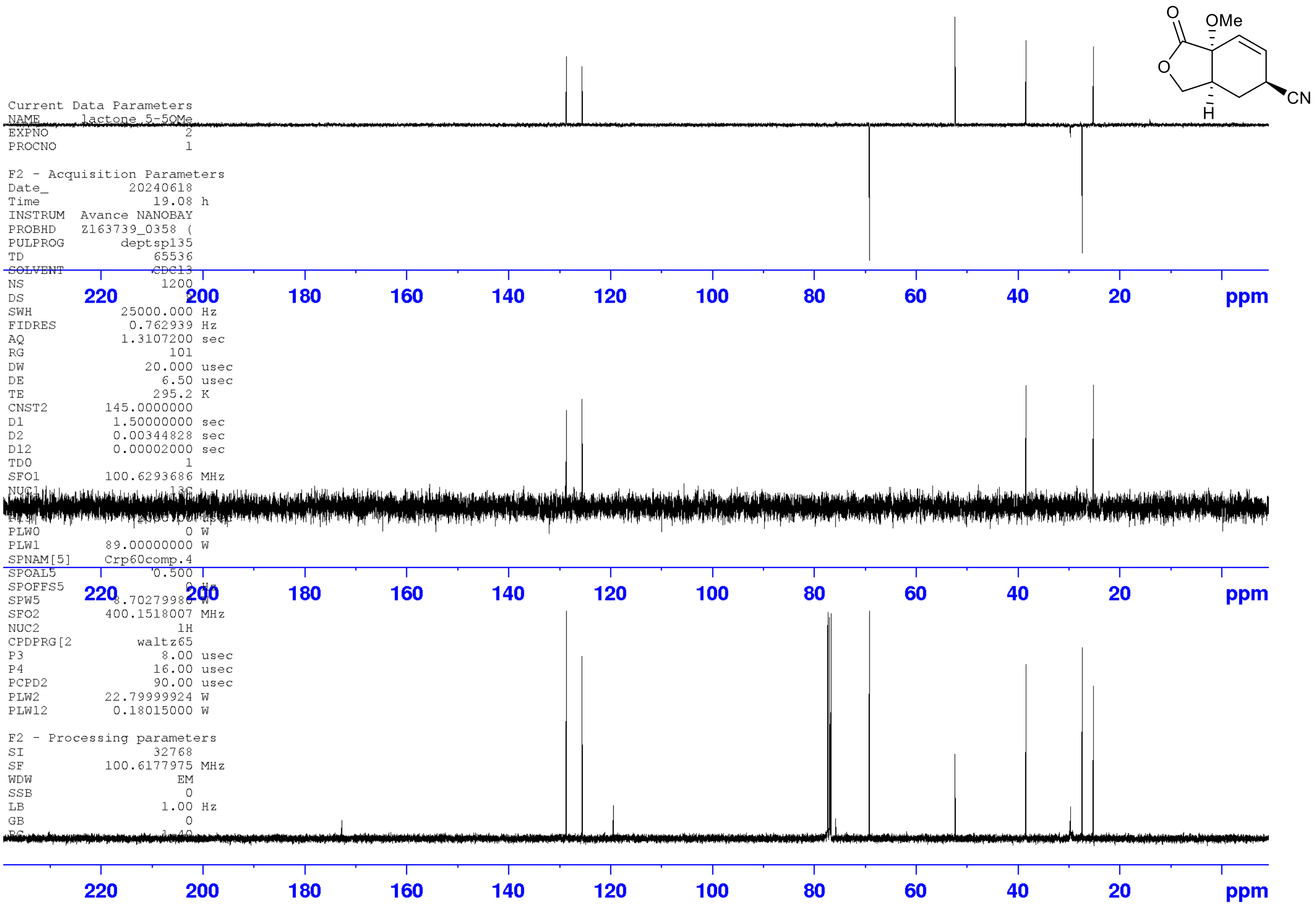

^{1}H NMR of 4i

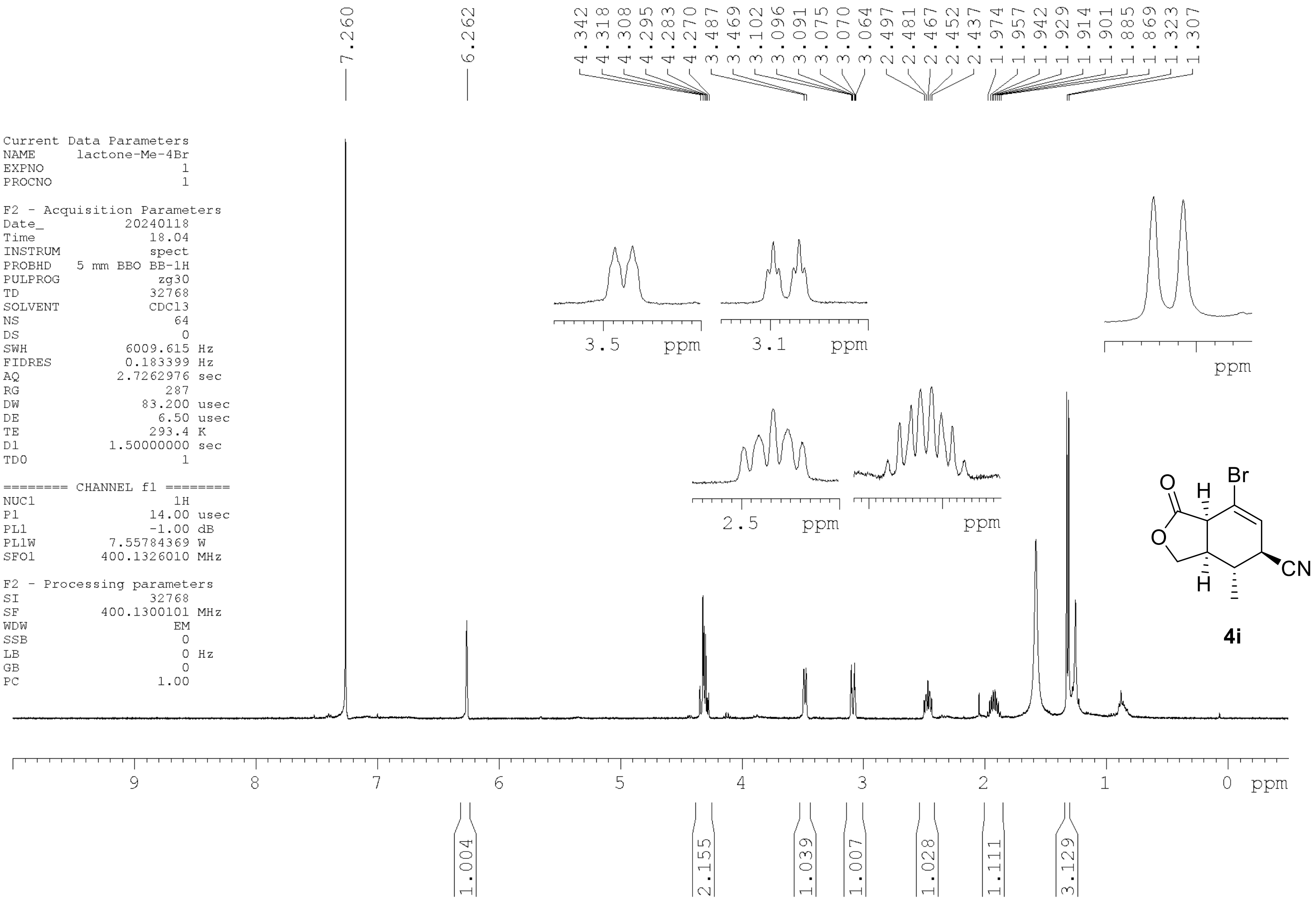

4i

^{13}C{1H} and DEPT 90, 135 NMR of 4i

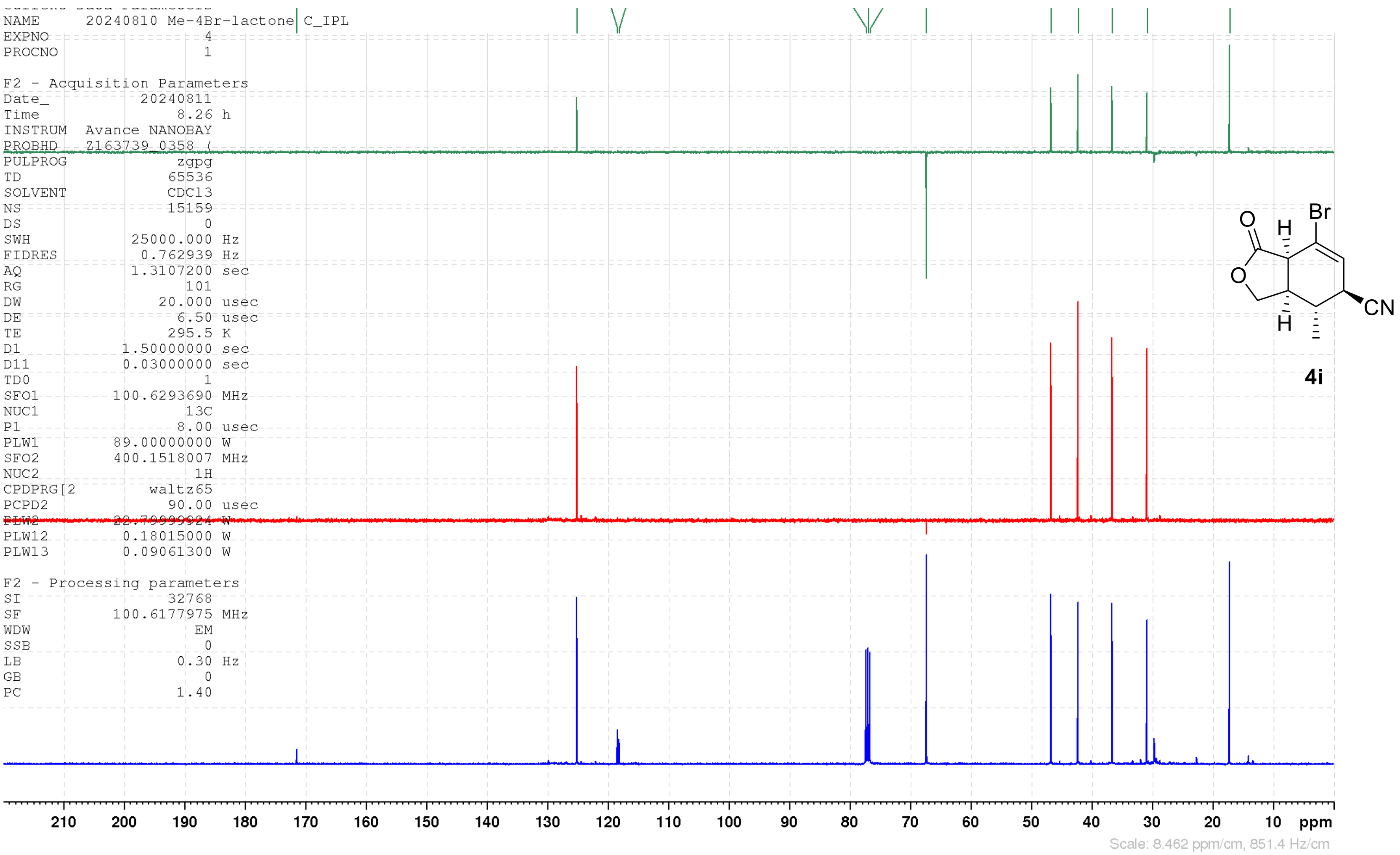

^{1}H NMR of 4j

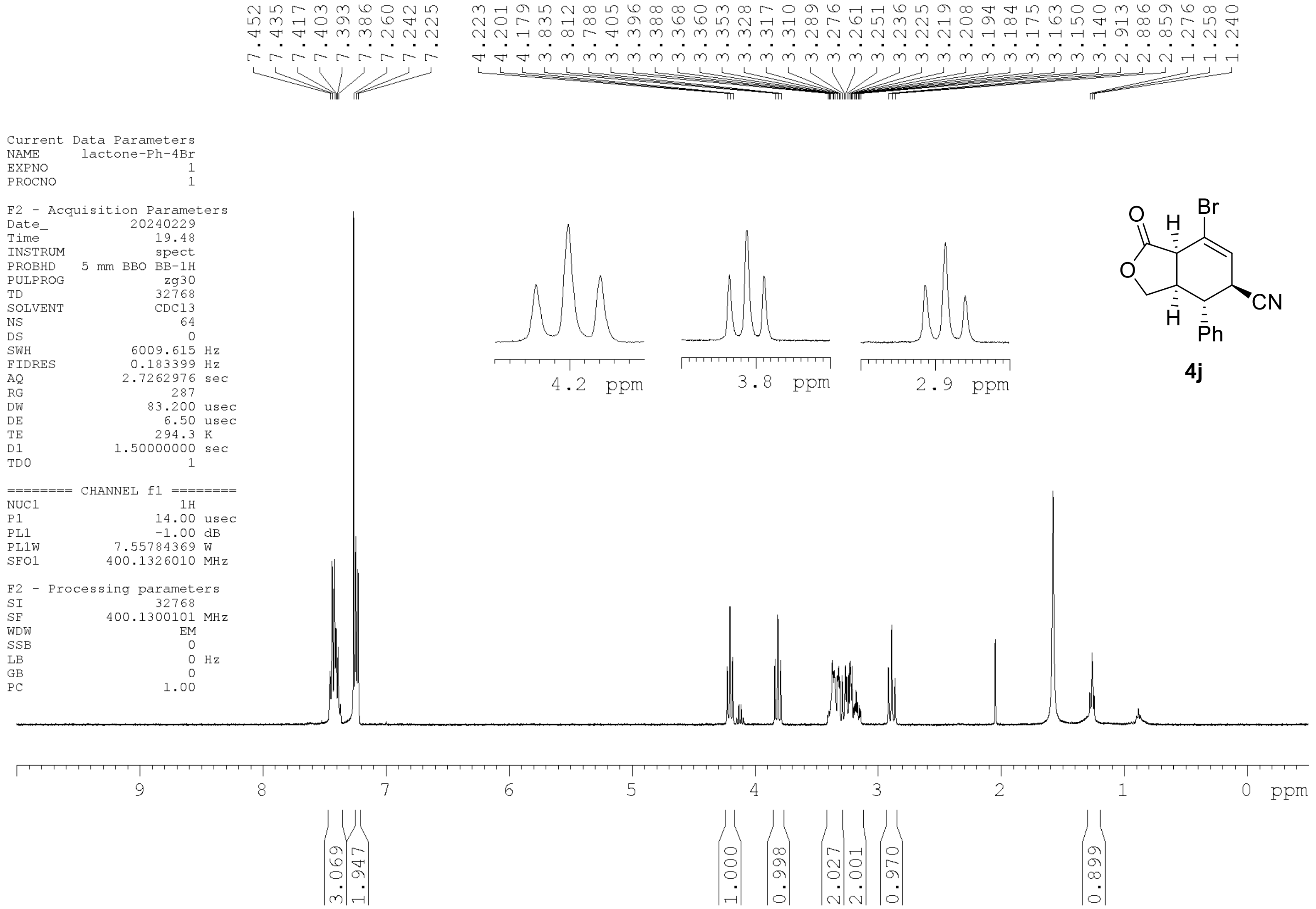

4j

^{13}C{1H} and DEPT 90, 135 NMR of 4j

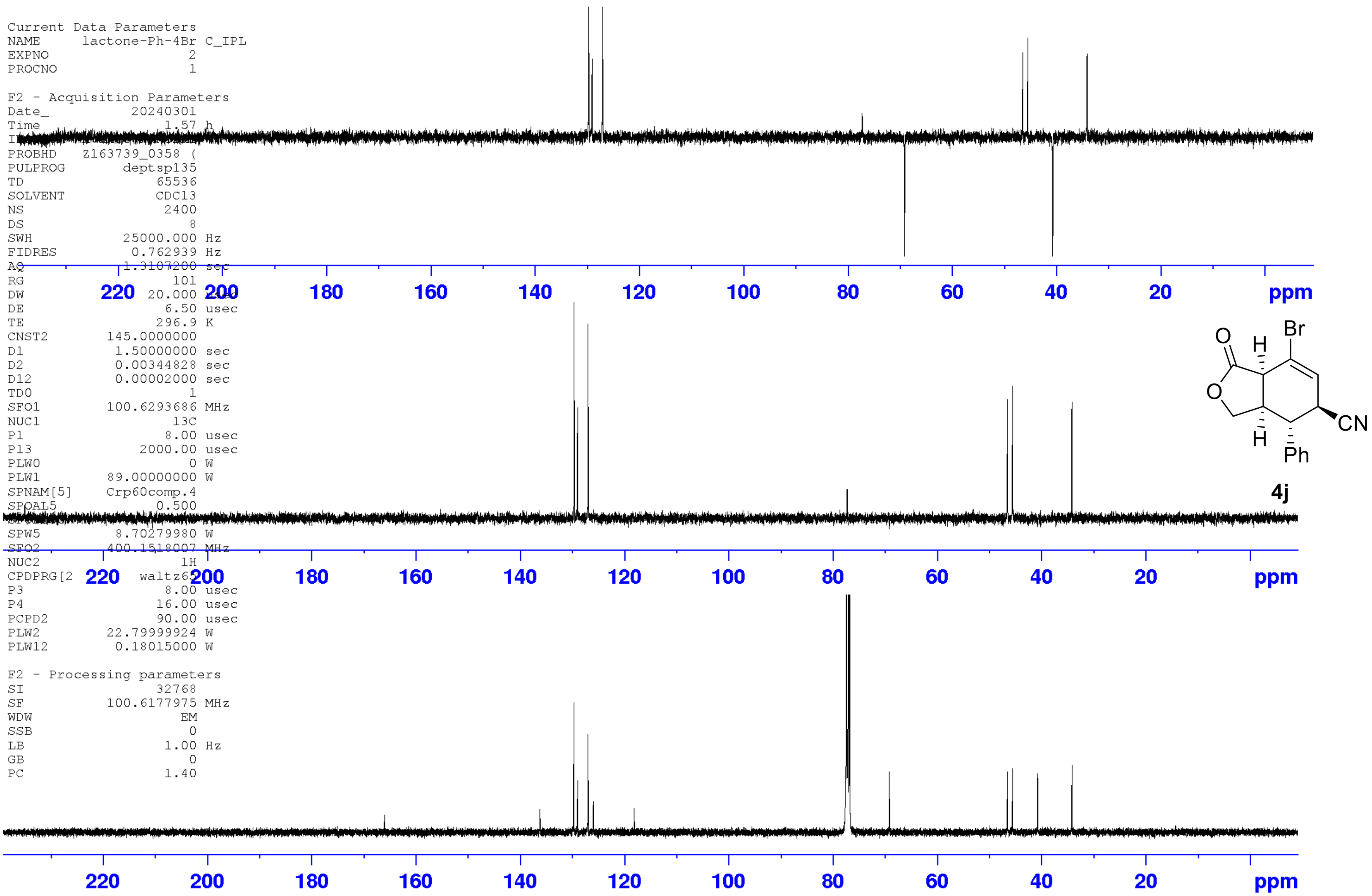

^{1}H NMR of 4k

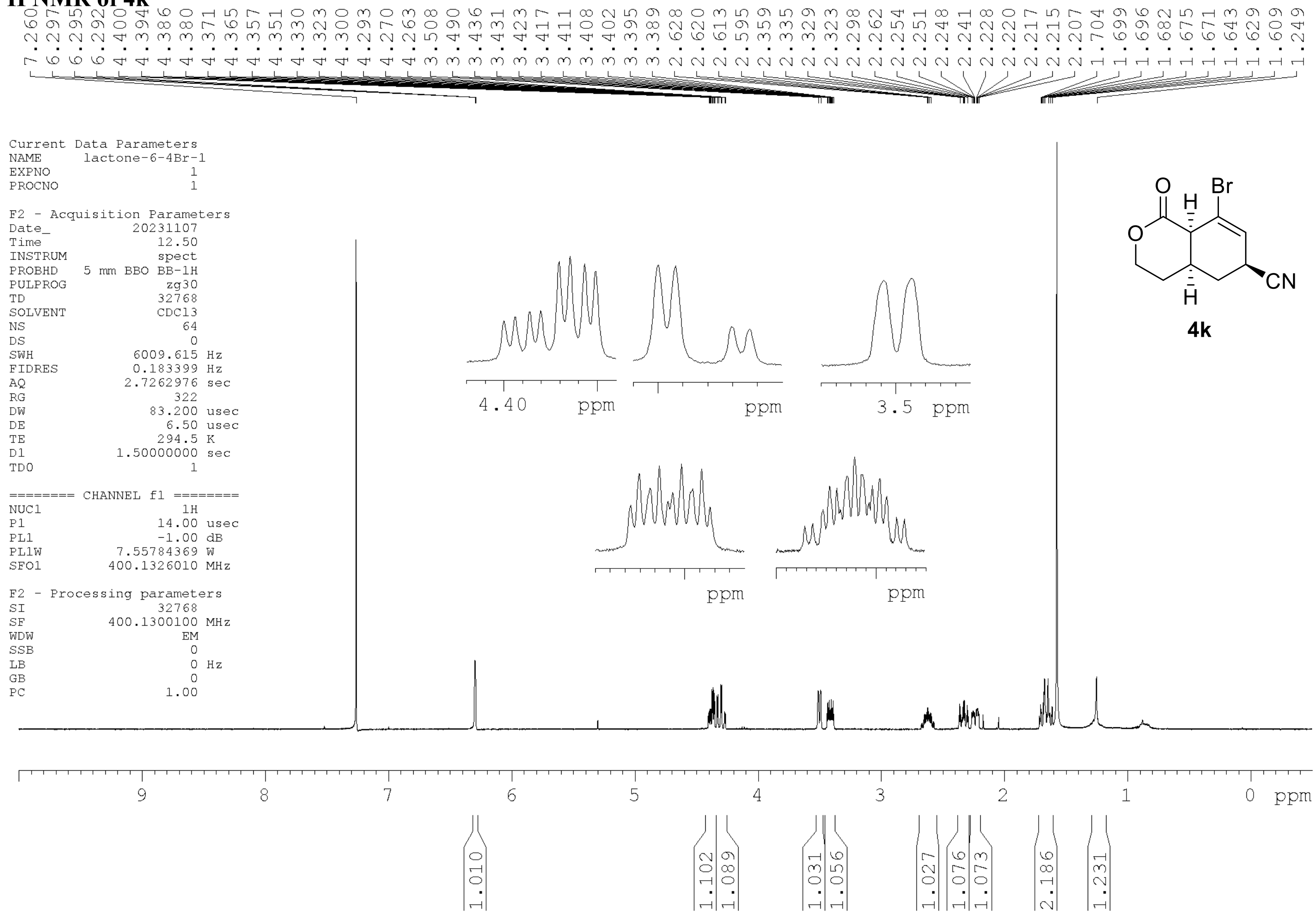

^{13}C{1H} and DEPT 90, 135 NMR of 4k

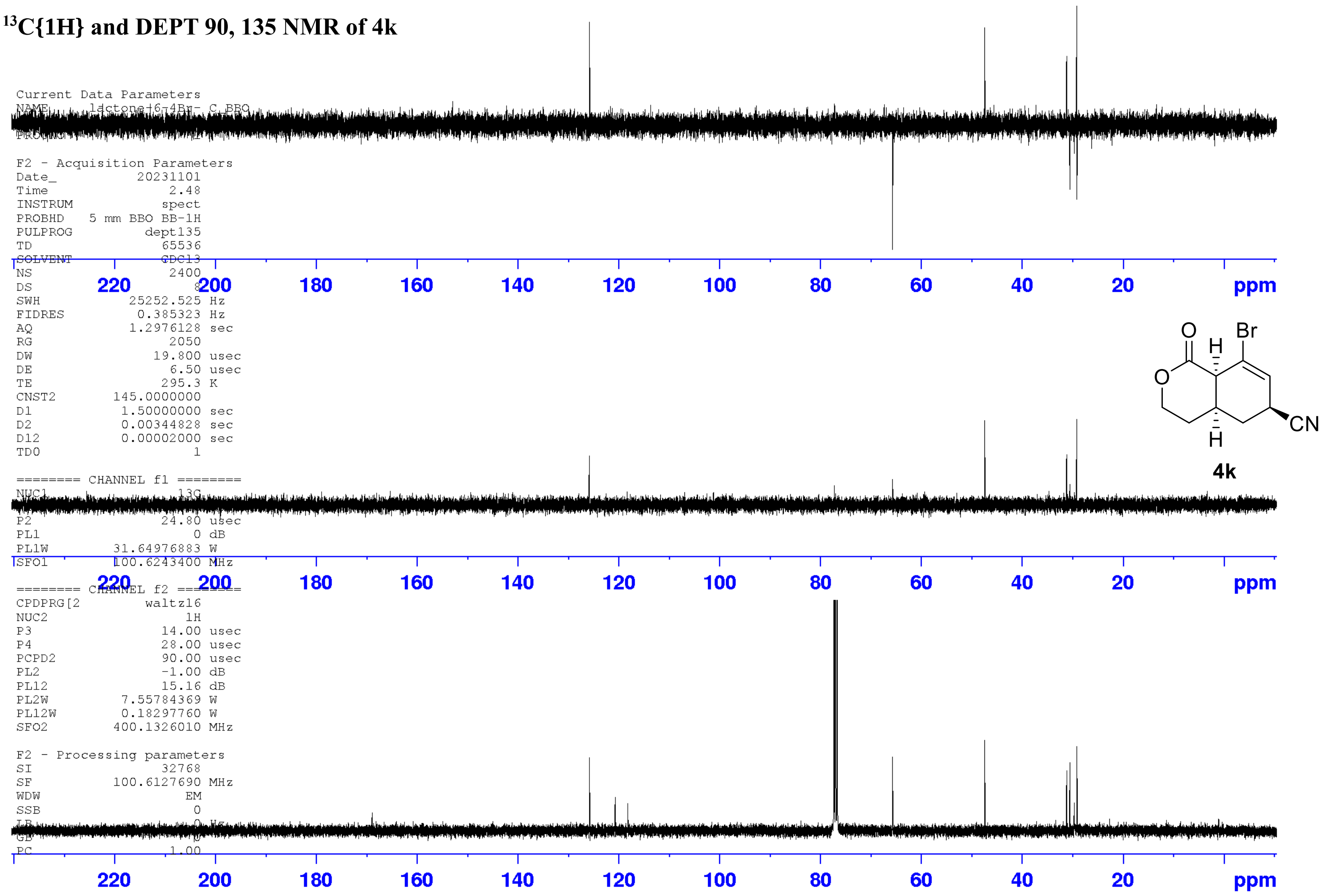

4k

^{1}H NMR of 5a

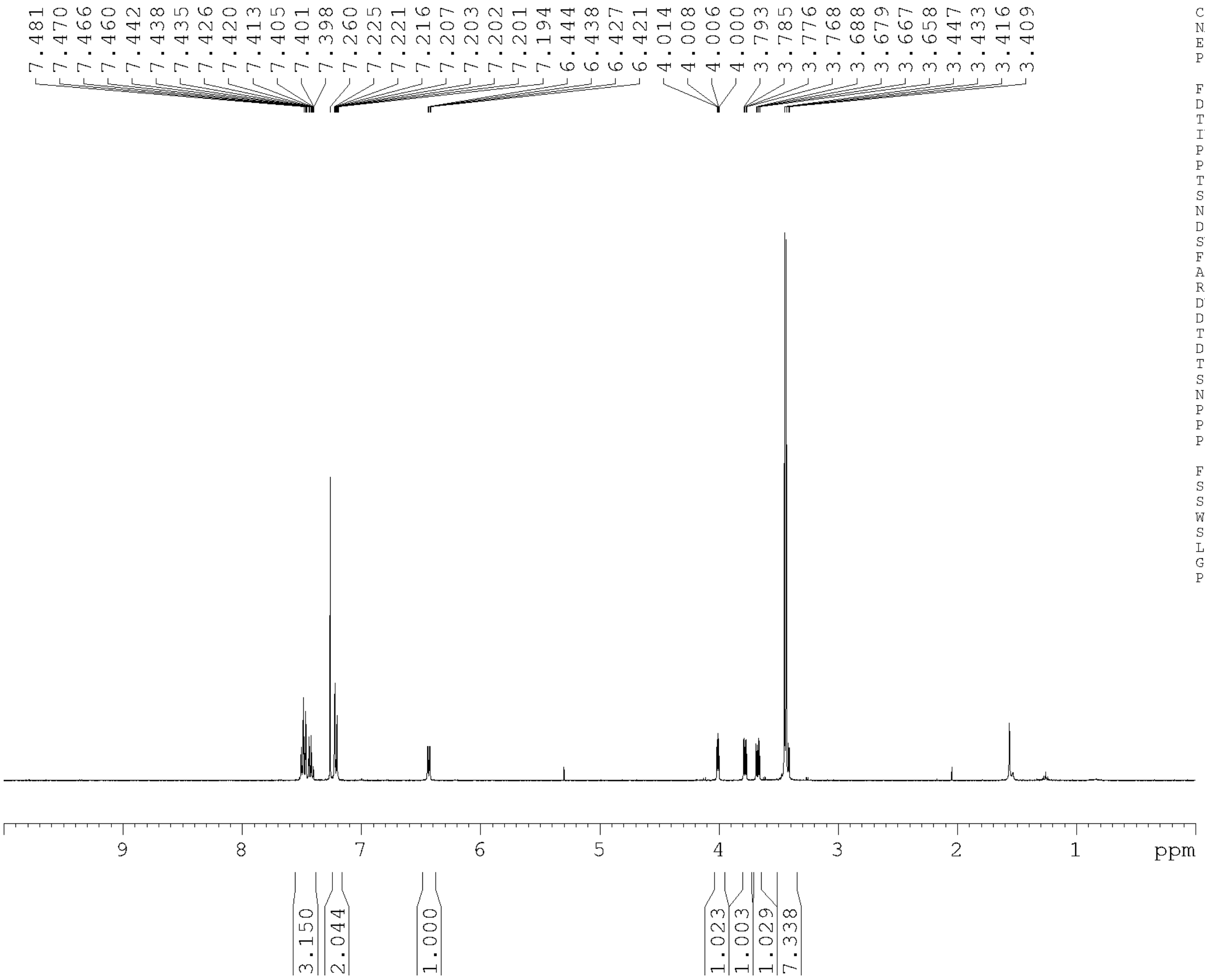

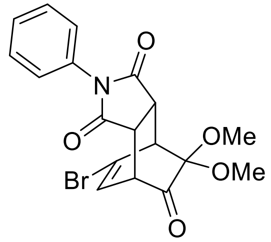

5a

^{1}H NMR of 5b

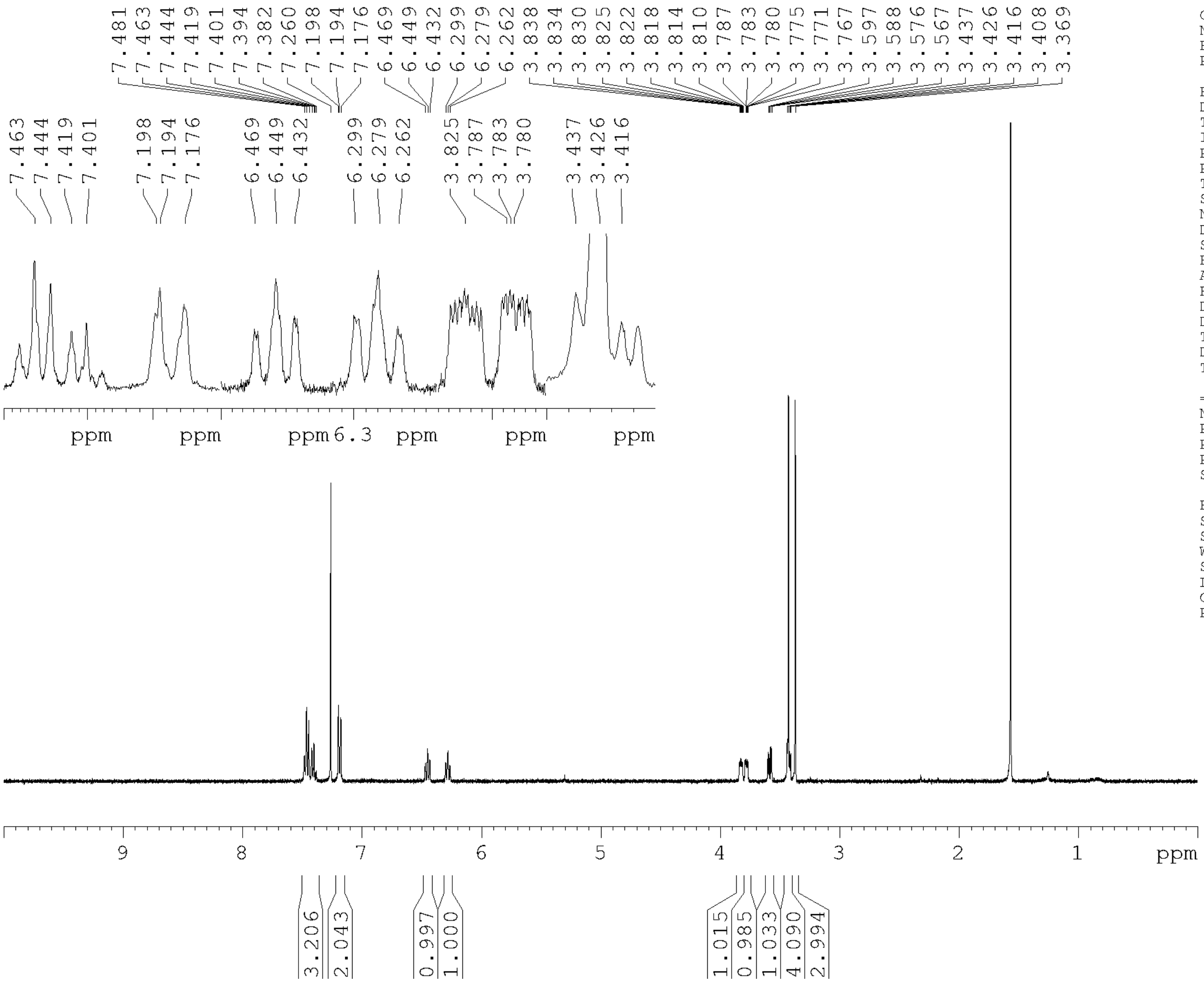

Current Data Parameters
NAME 20230428 SZ103-2
EXPNO 1
PROCNO 1

F2 - Acquisition Parameters
Date_ 20230428
Time 21.12
INSTRUM spect
PROBHD 5 mm BBO BB-1H
PULPROG zg30
TD 32768
SOLVENT CDCl3
NS 5
DS 0
SWH 6009.615 Hz
FIDRES 0.183399 Hz
AQ 2.7262976 sec
RG 287
DW 83.200 usec
DE 6.50 usec
TE 292.5 K
D1 1.50000000 sec
TD0 1

======== CHANNEL f1 ========
NUC1 1H
P1 14.00 usec
PL1 -1.00 dB
PL1W 7.55784369 W
SFO1 400.1326010 MHz

F2 - Processing parameters
SI 32768
SF 400.1300099 MHz
WDW EM
SSB 0
LB 0 Hz
GB 0
PC 1.00

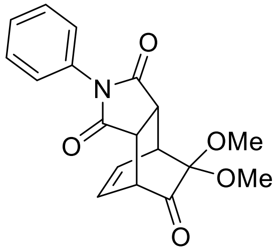

5b

^{1}H NMR of 5c

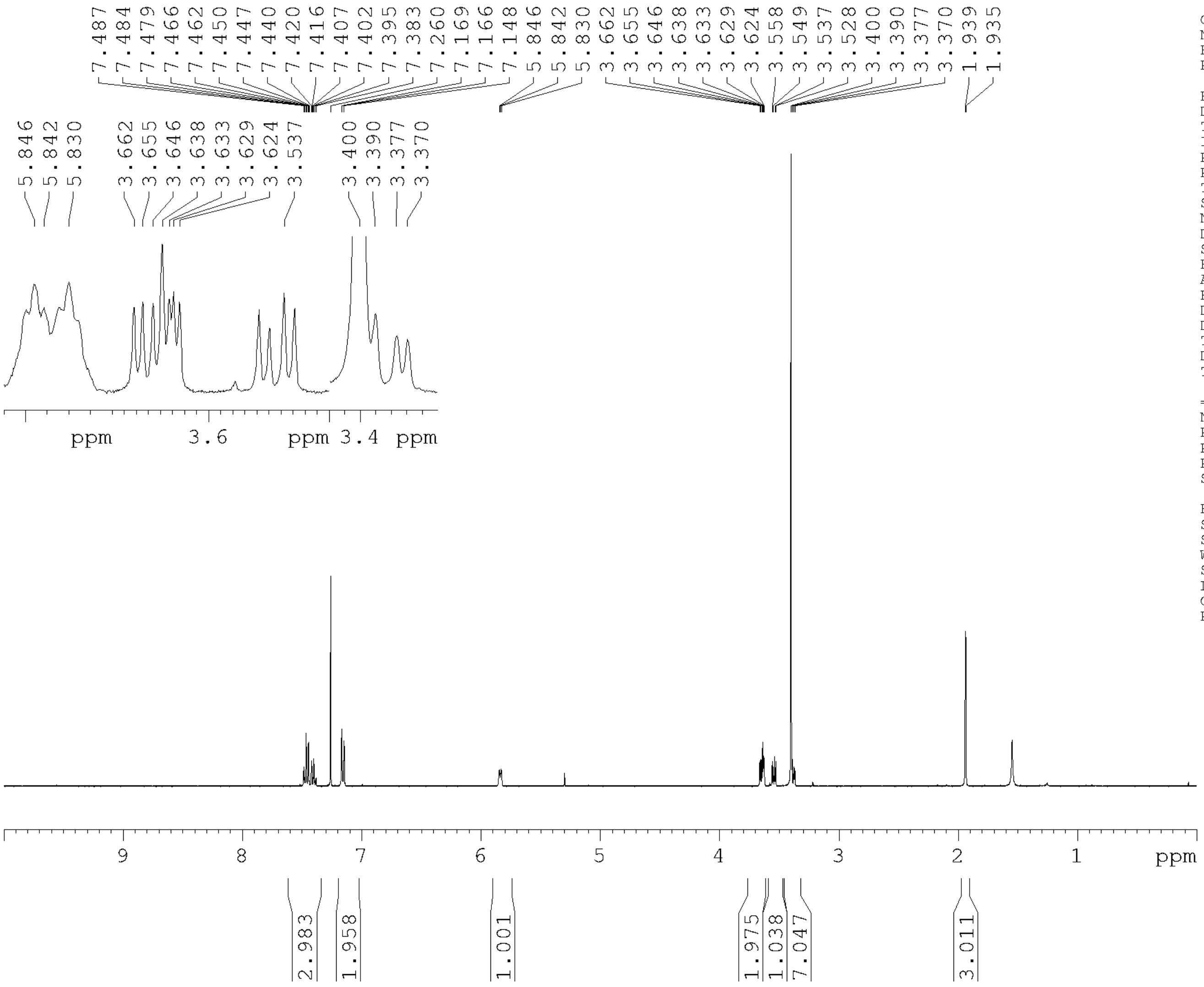


```
Current Data Parameters
NAME     20230918 N-4-MeDA
EXPNO                   1
PROCNO                  1

F2 - Acquisition Parameters
Date_            20230918
Time                13.56
INSTRUM             spect
PROBHD   5 mm BBO BB-1H
PULPROG              zg30
TD                  32768
SOLVENT             CDCl3
NS                     16
DS                      0
SWH              6009.615 Hz
FIDRES           0.183399 Hz
AQ              2.7262976 sec
RG                    256
DW                 83.200 usec
DE                   6.50 usec
TE                  295.1 K
D1             1.50000000 sec
TD0                     1

======== CHANNEL f1 ========
NUC1                   1H
P1                  14.00 usec
PL1                 -1.00 dB
PL1W           7.55784369 W
SFO1          400.1326010 MHz

F2 - Processing parameters
SI                  32768
SF            400.1300099 MHz
WDW                    EM
SSB                     0
LB                      0 Hz
GB                      0
PC                   1.00
```

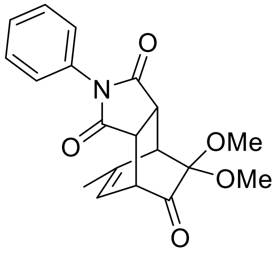

5c

^{1}H NMR of 5d

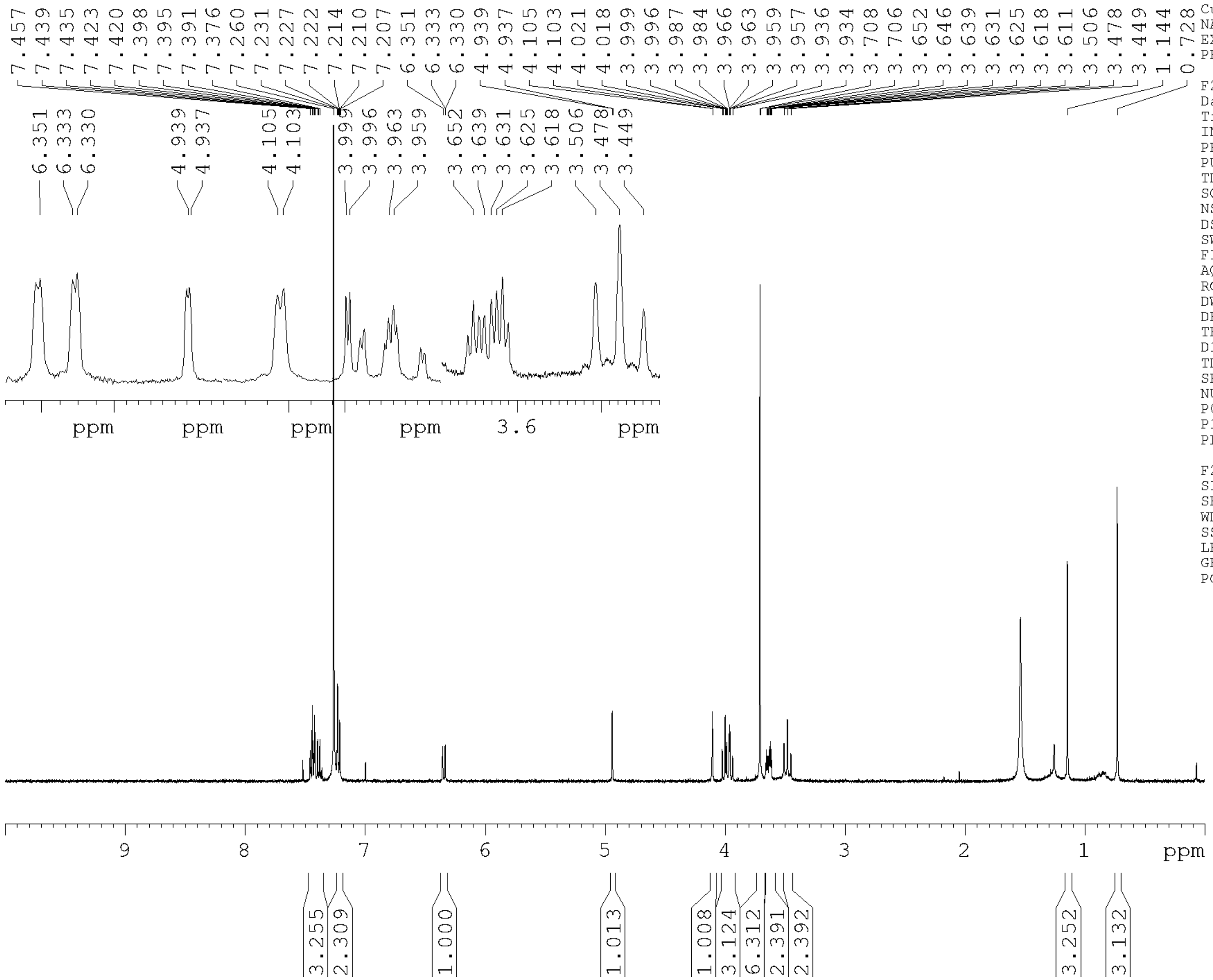

Current Data Parameters
NAME 20240122 SZ266
EXPNO 1
PROCNO 1

F2 - Acquisition Parameters
Date_ 20240122
Time 19.17 h
INSTRUM Avance NANOBAY
PROBHD Z163739_0358 (
PULPROG zg30
TD 32768
SOLVENT CDCl3
NS 16
DS 0
SWH 5882.353 Hz
FIDRES 0.359030 Hz
AQ 2.7852800 sec
RG 101
DW 85.000 usec
DE 9.26 usec
TE 296.1 K
D1 1.50000000 sec
TD0 1
SFO1 400.1526010 MHz
NUC1 1H
P0 2.67 usec
P1 8.00 usec
PLW1 21.10000038 W

F2 - Processing parameters
SI 32768
SF 400.1500098 MHz
WDW EM
SSB 0
LB 0.10 Hz
GB 0
PC 1.00

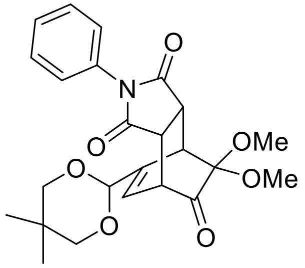

5d

^{1}H NMR of 5e

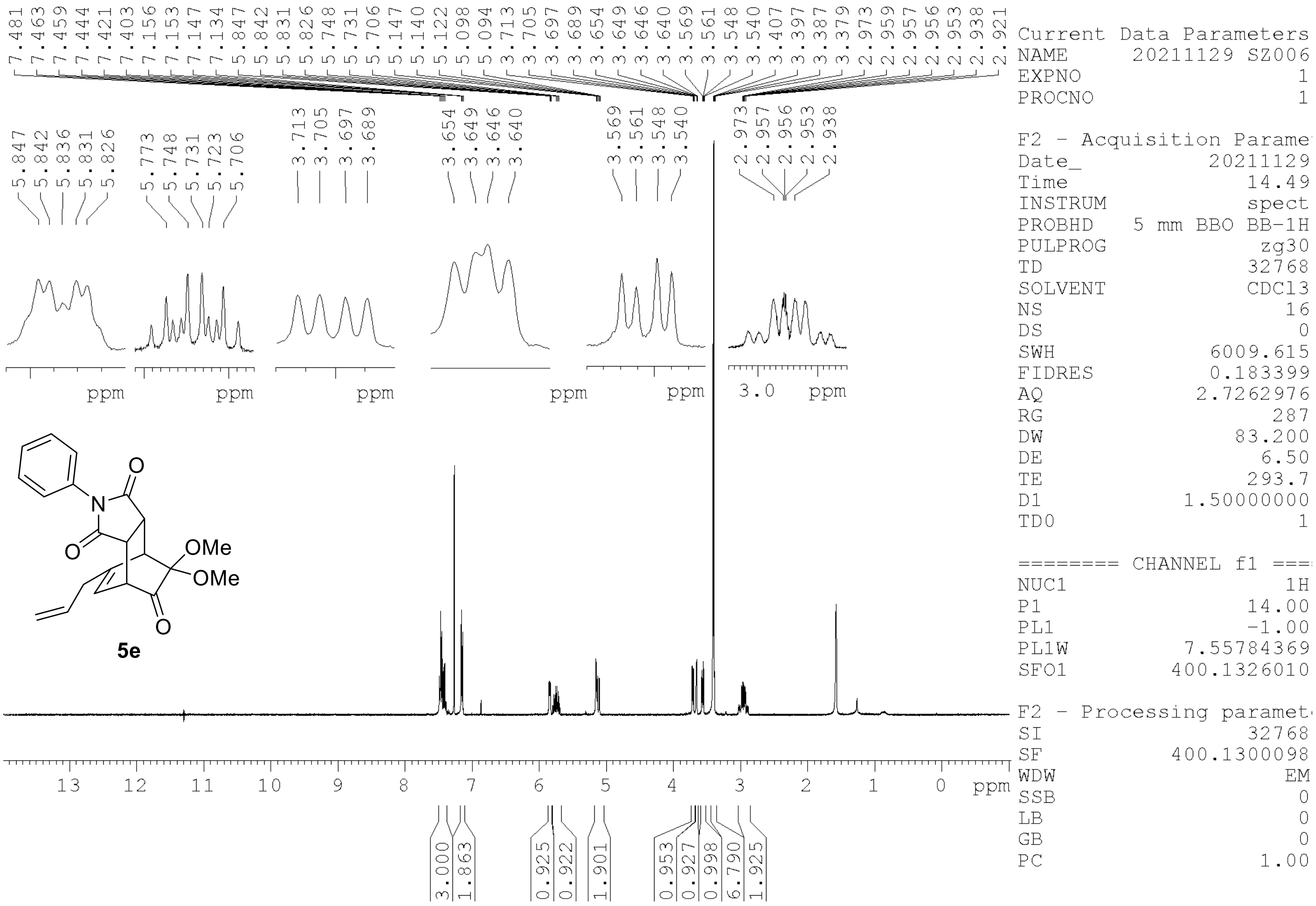
5e
Current Data Parameters
NAME 20211129 SZ006
EXPNO 1
PROCNO 1
F2 - Acquisition Parame
Date_ 20211129
Time 14.49
INSTRUM spect
PROBHD 5 mm BBO BB-1H
PULPROG zg30
TD 32768
SOLVENT CDCl3
NS 16
DS 0
SWH 6009.615
FIDRES 0.183399
AQ 2.7262976
RG 287
DW 83.200
DE 6.50
TE 293.7
D1 1.50000000
TD0 1
======== CHANNEL f1 ===
NUC1 1H
P1 14.00
PL1 -1.00
PL1W 7.55784369
SFO1 400.1326010
F2 - Processing paramet
SI 32768
SF 400.1300098
WDW EM
SSB 0
LB 0
GB 0
PC 1.00

^{1}H NMR of 5f

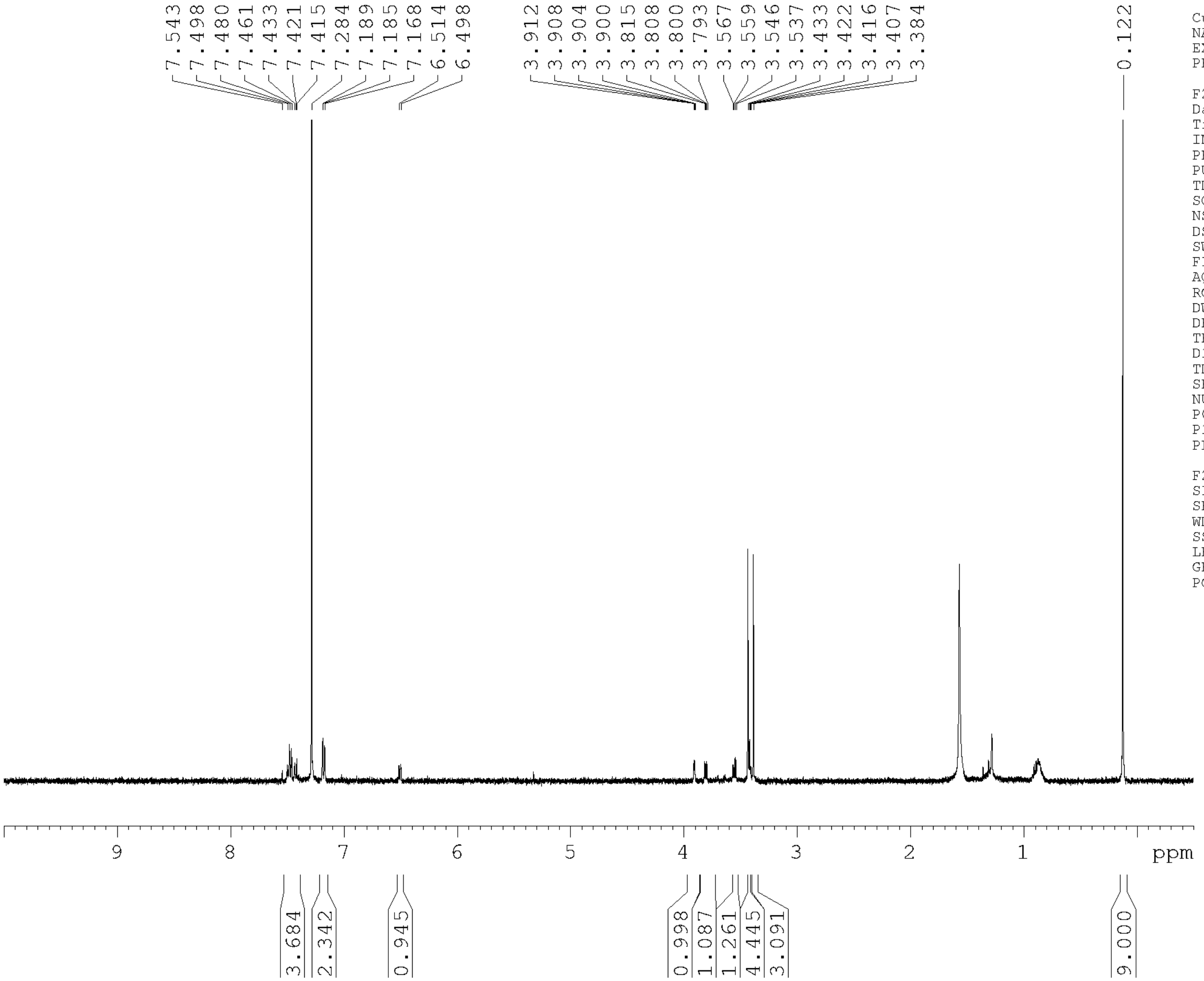

Current Data Parameters
NAME 20230703 4-TMS-D-1
EXPNO 1
PROCNO 1

F2 - Acquisition Parameters
Date_ 20230703
Time 22.16 h
INSTRUM Avance NANOBAY
PROBHD Z163739_0358 (
PULPROG zg30
TD 32768
SOLVENT CDCl3
NS 1
DS 0
SWH 5882.353 Hz
FIDRES 0.359030 Hz
AQ 2.7852800 sec
RG 101
DW 85.000 usec
DE 9.26 usec
TE 296.9 K
D1 1.50000000 sec
TD0 1
SFO1 400.1526010 MHz
NUC1 1H
P0 2.67 usec
P1 8.00 usec
PLW1 21.10000038 W

F2 - Processing parameters
SI 32768
SF 400.1500000 MHz
WDW EM
SSB 0
LB 0.10 Hz
GB 0
PC 1.00

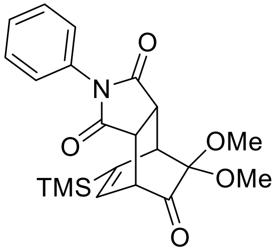

5f

^{1}H NMR of 5g

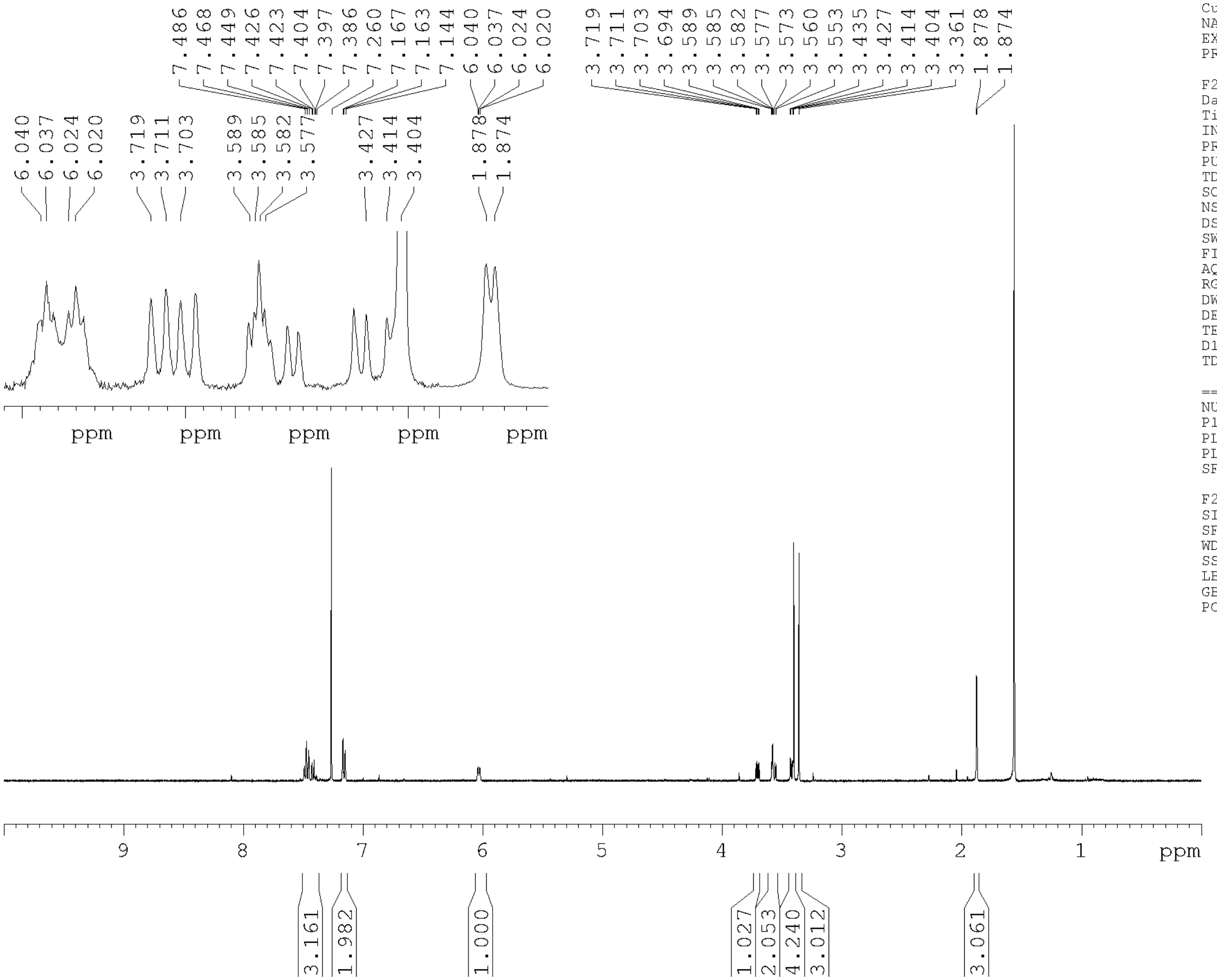

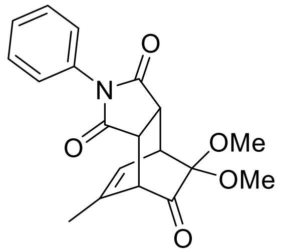

5g

^{1}H NMR of 5h

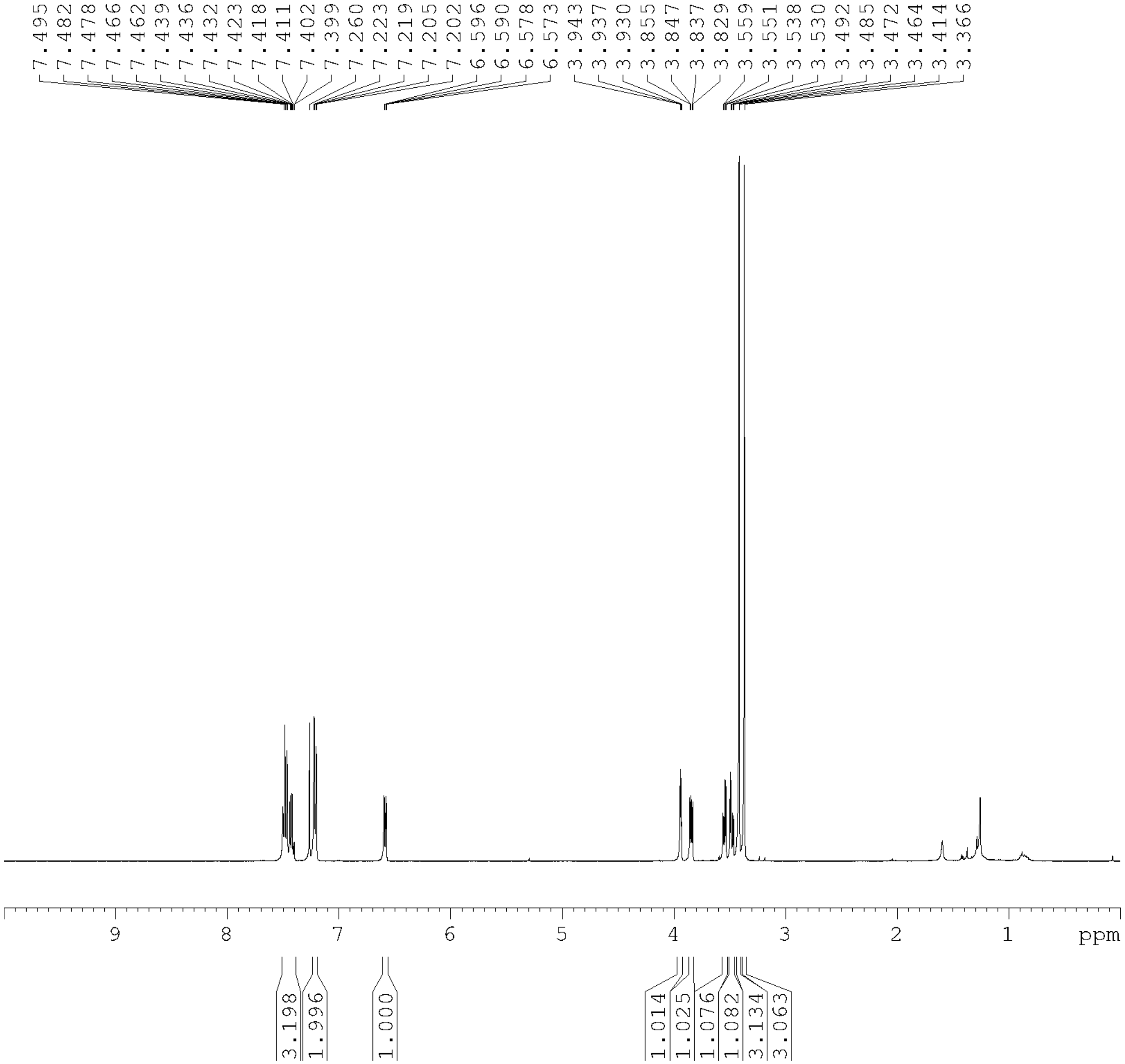


```
Current Data Parameters
NAME          20241219-5h
EXPNO                   1
PROCNO                  1

F2 - Acquisition Parameters
Date_            20241219
Time                20.08
INSTRUM             spect
PROBHD   5 mm BBO BB-1H
PULPROG              zg30
TD                  32768
SOLVENT             CDCl3
NS                     16
DS                      0
SWH             6009.615 Hz
FIDRES          0.183399 Hz
AQ             2.7262976 sec
RG                    114
DW                 83.200 usec
DE                   6.50 usec
TE                  296.0 K
D1             1.50000000 sec
TD0                     1

======== CHANNEL f1 ========
NUC1                   1H
P1                  14.00 usec
PL1                 -1.00 dB
PL1W           7.55784369 W
SFO1          400.1326010 MHz

F2 - Processing parameters
SI                  32768
SF            400.1300098 MHz
WDW                    EM
SSB                     0
LB                      0 Hz
GB                      0
PC                   1.00
```

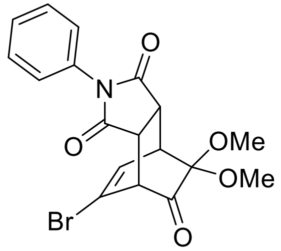

5h

^{1}H NMR of 5i

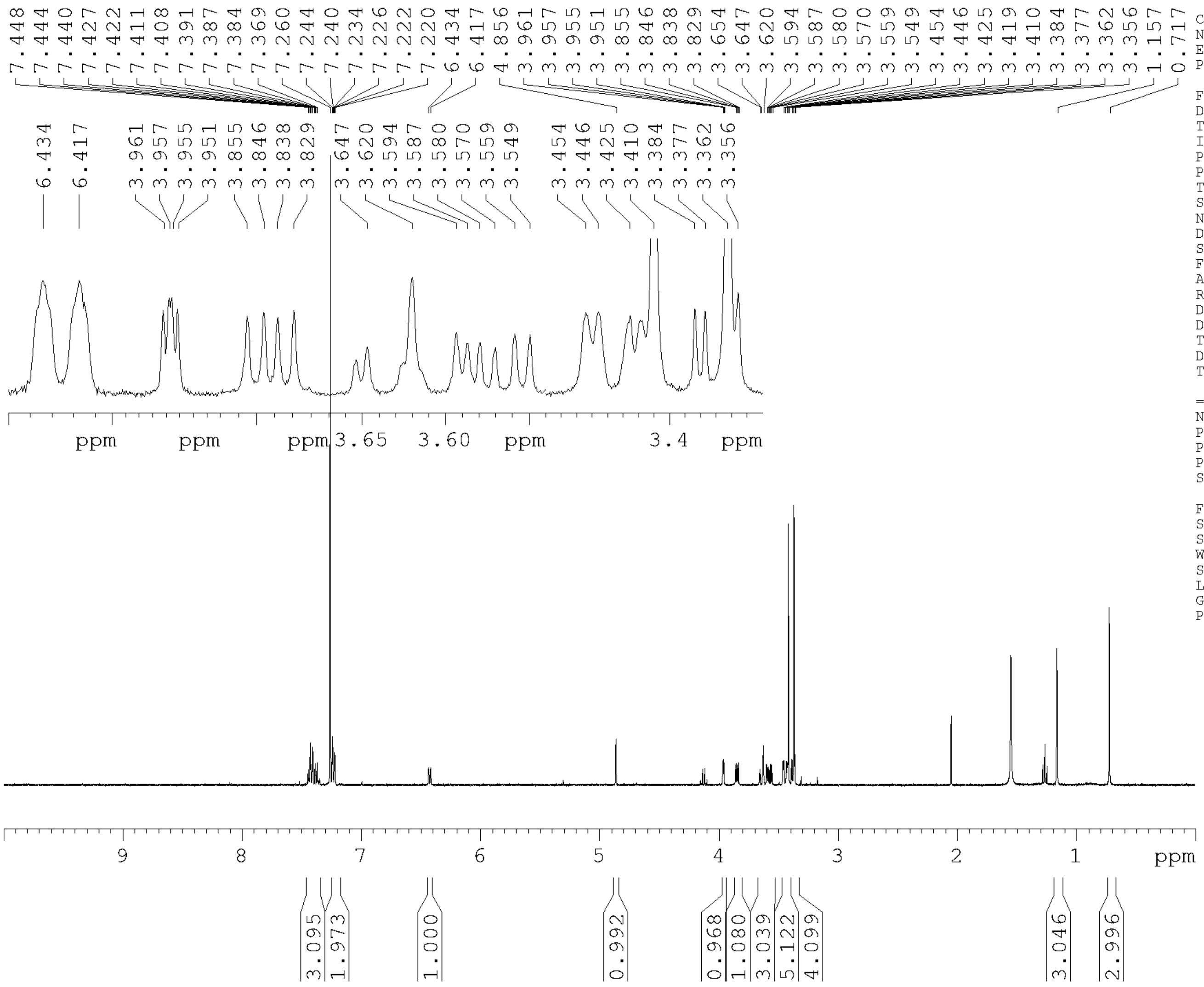

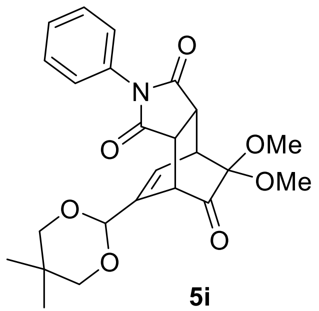

5i

^{1}H NMR of 5j

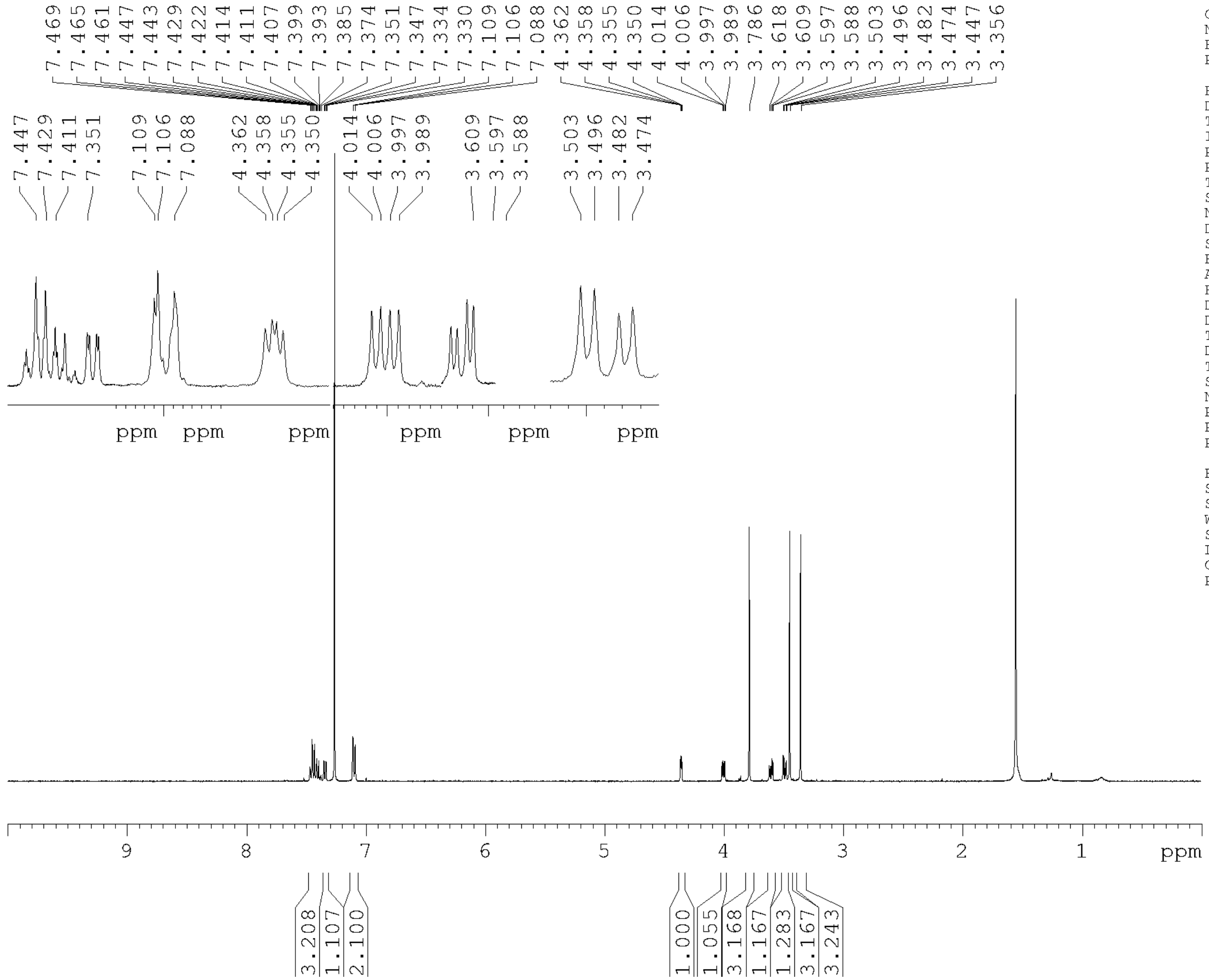


```
Current Data Parameters
NAME     20240306 4-CO2Me-D
EXPNO                     1
PROCNO                    1

F2 - Acquisition Parameters
Date_              20240306
Time                  14.12 h
INSTRUM   Avance NANOBAY
PROBHD    Z163739_0358 (
PULPROG                zg30
TD                    32768
SOLVENT               CDCl3
NS                       16
DS                        0
SWH                5882.353 Hz
FIDRES             0.359030 Hz
AQ                2.7852800 sec
RG                      101
DW                   85.000 usec
DE                     9.26 usec
TE                    296.2 K
D1               1.50000000 sec
TD0                       1
SFO1            400.1526010 MHz
NUC1                     1H
P0                     2.67 usec
P1                     8.00 usec
PLW1            21.10000038 W

F2 - Processing parameters
SI                    32768
SF              400.1500097 MHz
WDW                      EM
SSB                       0
LB                     0.10 Hz
GB                        0
PC                     1.00
```

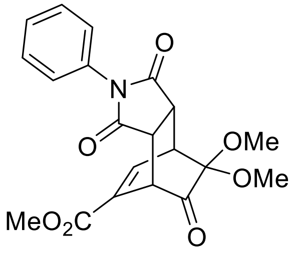

5j

^{1}H NMR of 5k

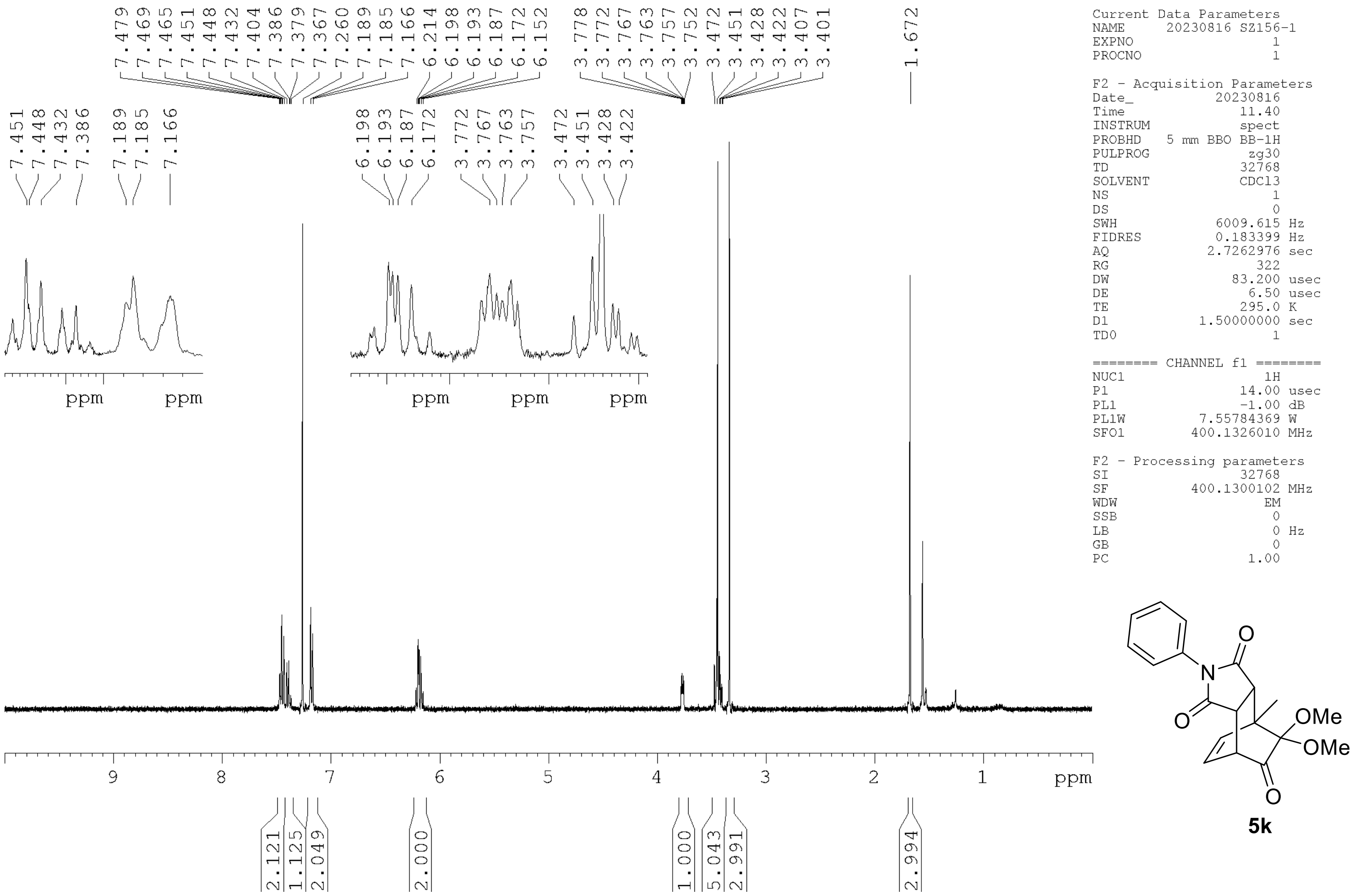

5k

^{1}H NMR of 5l

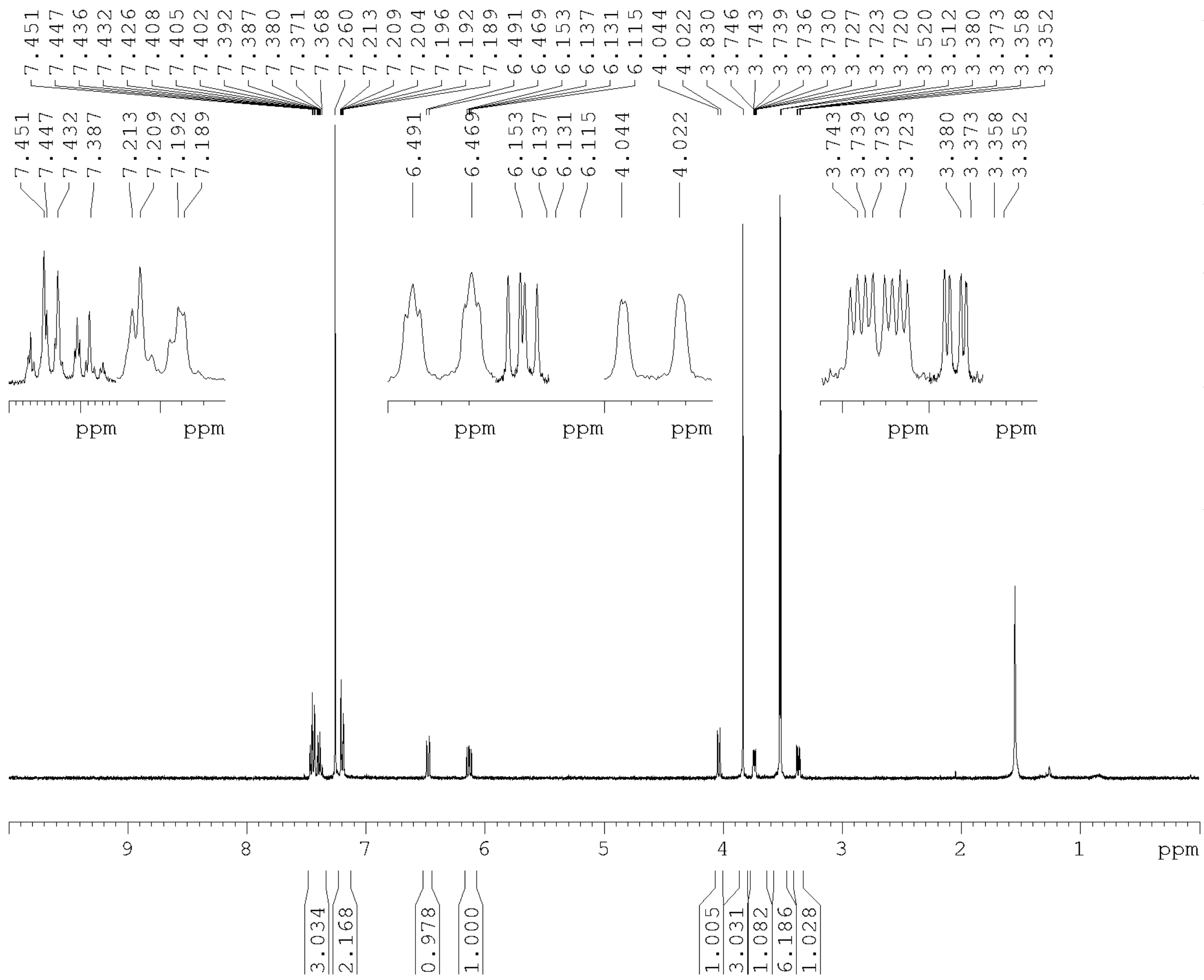

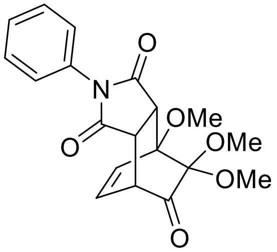

5l

^{1}H NMR of 5m

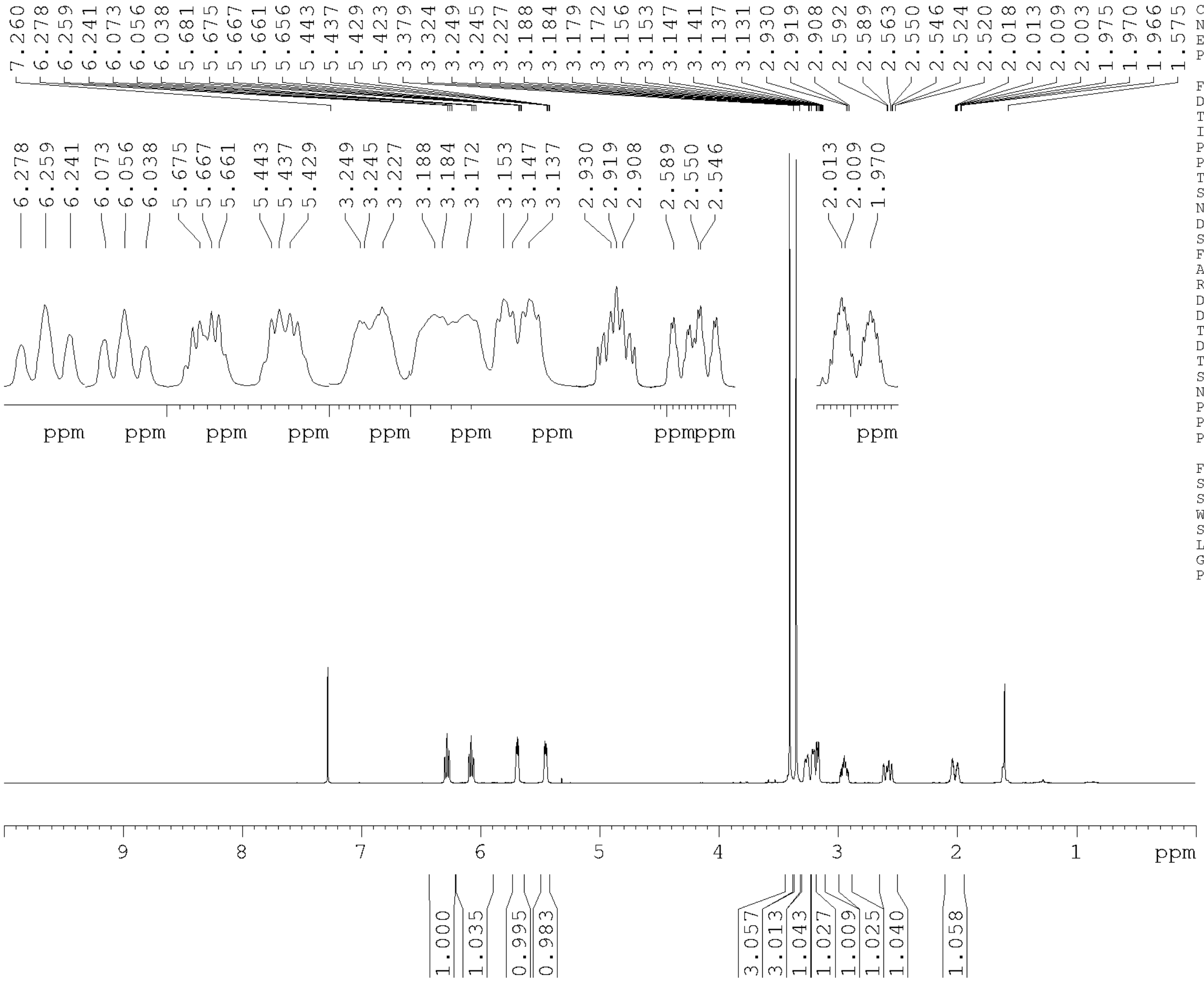

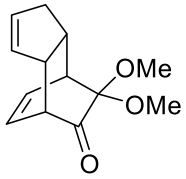

5m

^{1}H NMR of 5n

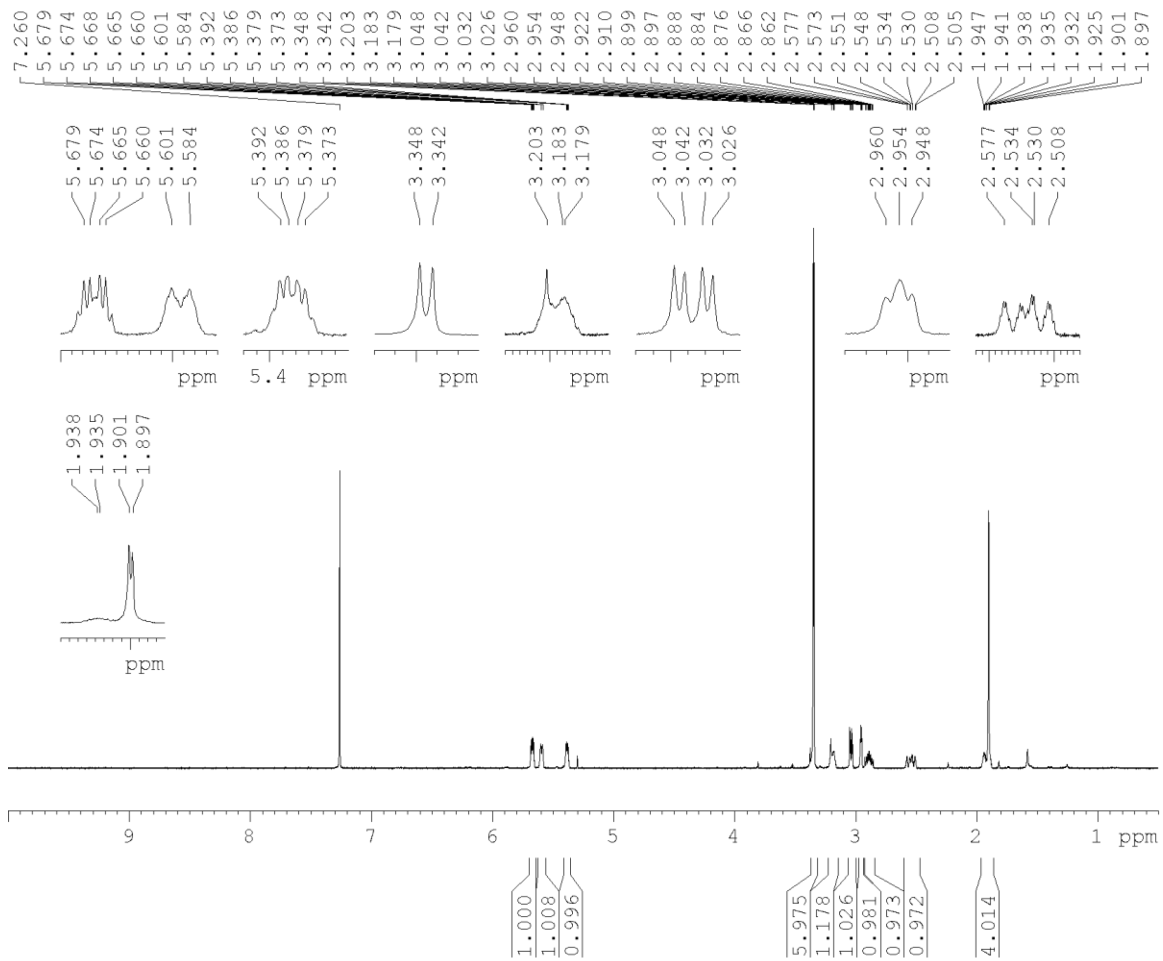

```
Current Data Parameters
NAME     20240729 DATA-4-Me-cp-DA
EXPNO              1
PROCNO             1

F2 - Acquisition Parameters
Date_          20240729
Time              22.28
INSTRUM           spect
PROBHD   5 mm BBO BB-1H
PULPROG            zg30
TD                32768
SOLVENT           CDCl3
NS                   33
DS                    0
SWH            6009.615 Hz
FIDRES         0.183399 Hz
AQ            2.7262976 sec
RG                  203
DW               83.200 usec
DE                 6.50 usec
TE                295.4 K
D1           1.50000000 sec
TD0                   1

======== CHANNEL f1 ========
NUC1                 1H
P1                14.00 usec
PL1               -1.00 dB
PL1W         7.55784369 W
SFO1        400.1326010 MHz

F2 - Processing parameters
SI                32768
SF          400.1300102 MHz
WDW                  EM
SSB                   0
LB                    0 Hz
GB                    0
PC                 1.00
```

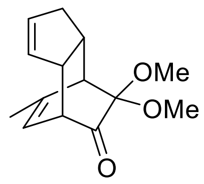

5n

^{1}H NMR of 5o

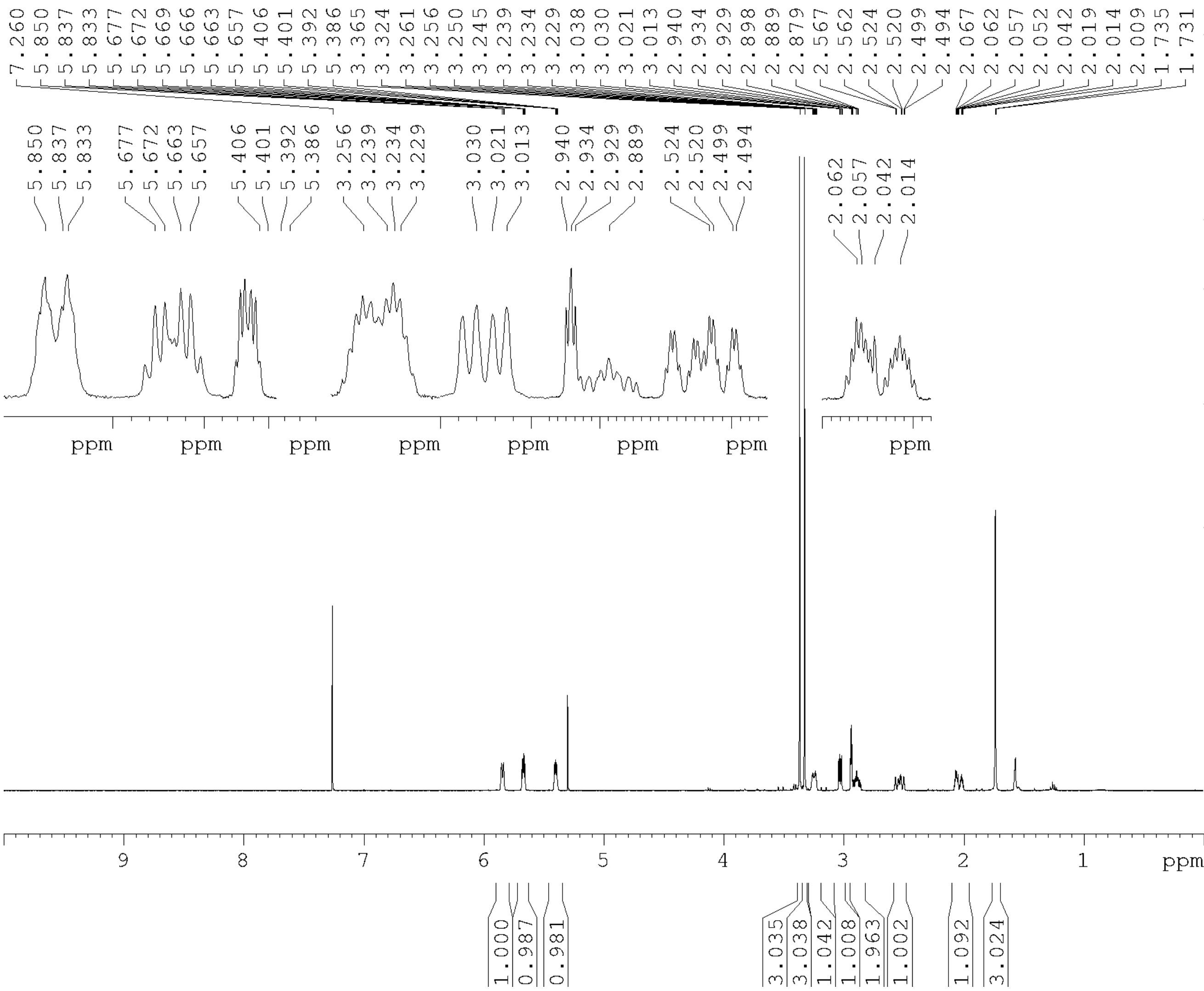

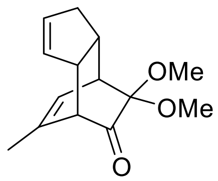

5o

^{1}H NMR of 5p

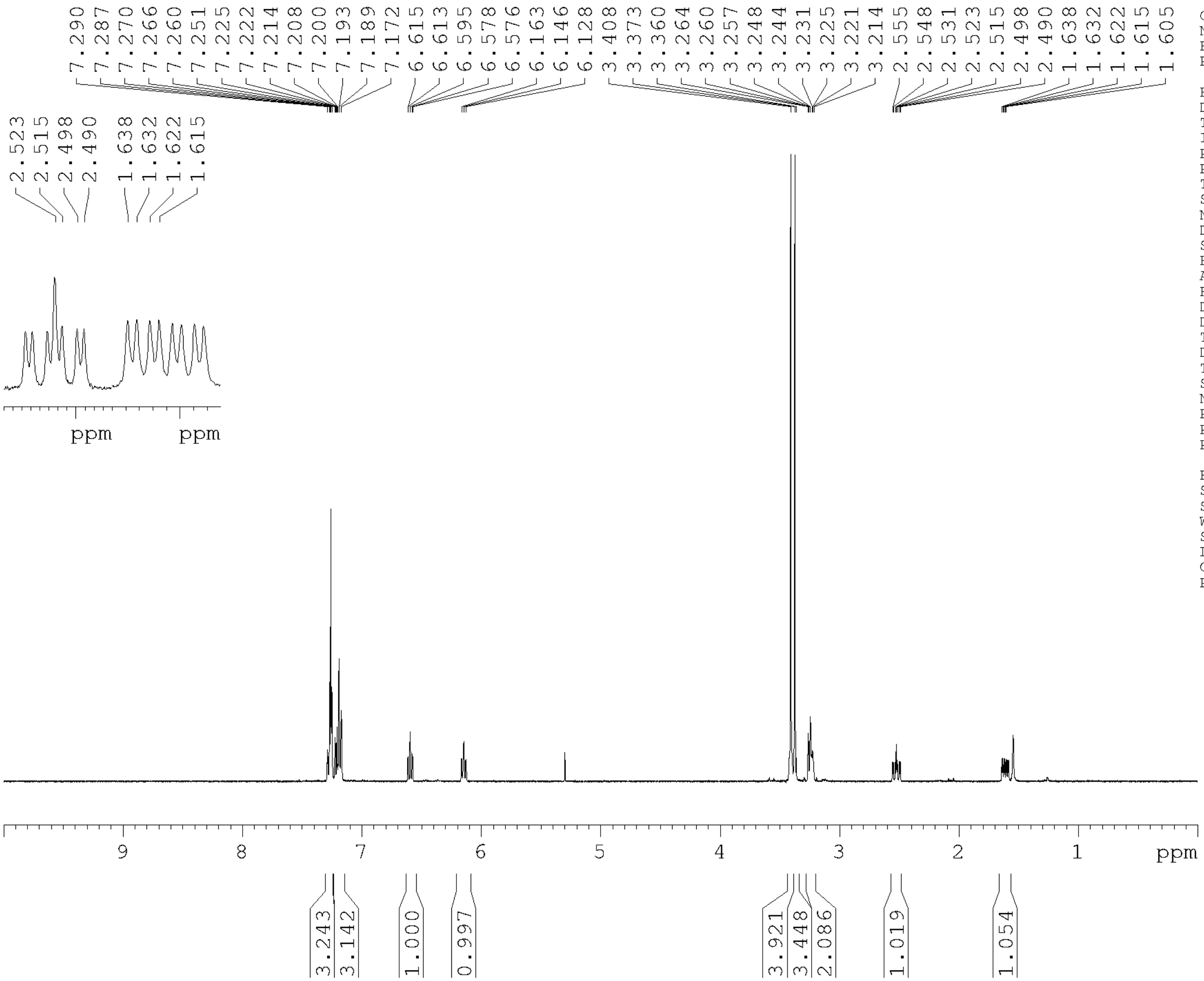


```
Current Data Parameters
NAME     20230927 NS-DS
EXPNO                 4
PROCNO                1

F2 - Acquisition Parameters
Date_          20230927
Time              13.25 h
INSTRUM  Avance NANOBAY
PROBHD   Z163739_0358 (
PULPROG            zg30
TD                32768
SOLVENT           CDCl3
NS                    1
DS                    0
SWH            5882.353 Hz
FIDRES         0.359030 Hz
AQ            2.7852800 sec
RG                  101
DW               85.000 usec
DE                 9.26 usec
TE                296.7 K
D1          1.50000000 sec
TD0                   1
SFO1       400.1526010 MHz
NUC1                 1H
P0                 2.67 usec
P1                 8.00 usec
PLW1        21.10000038 W

F2 - Processing parameters
SI                32768
SF          400.1500099 MHz
WDW                  EM
SSB                   0
LB                 0.10 Hz
GB                    0
PC                 1.00
```

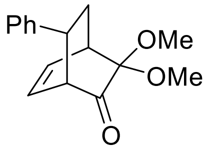

5p

^{1}H NMR of 5q

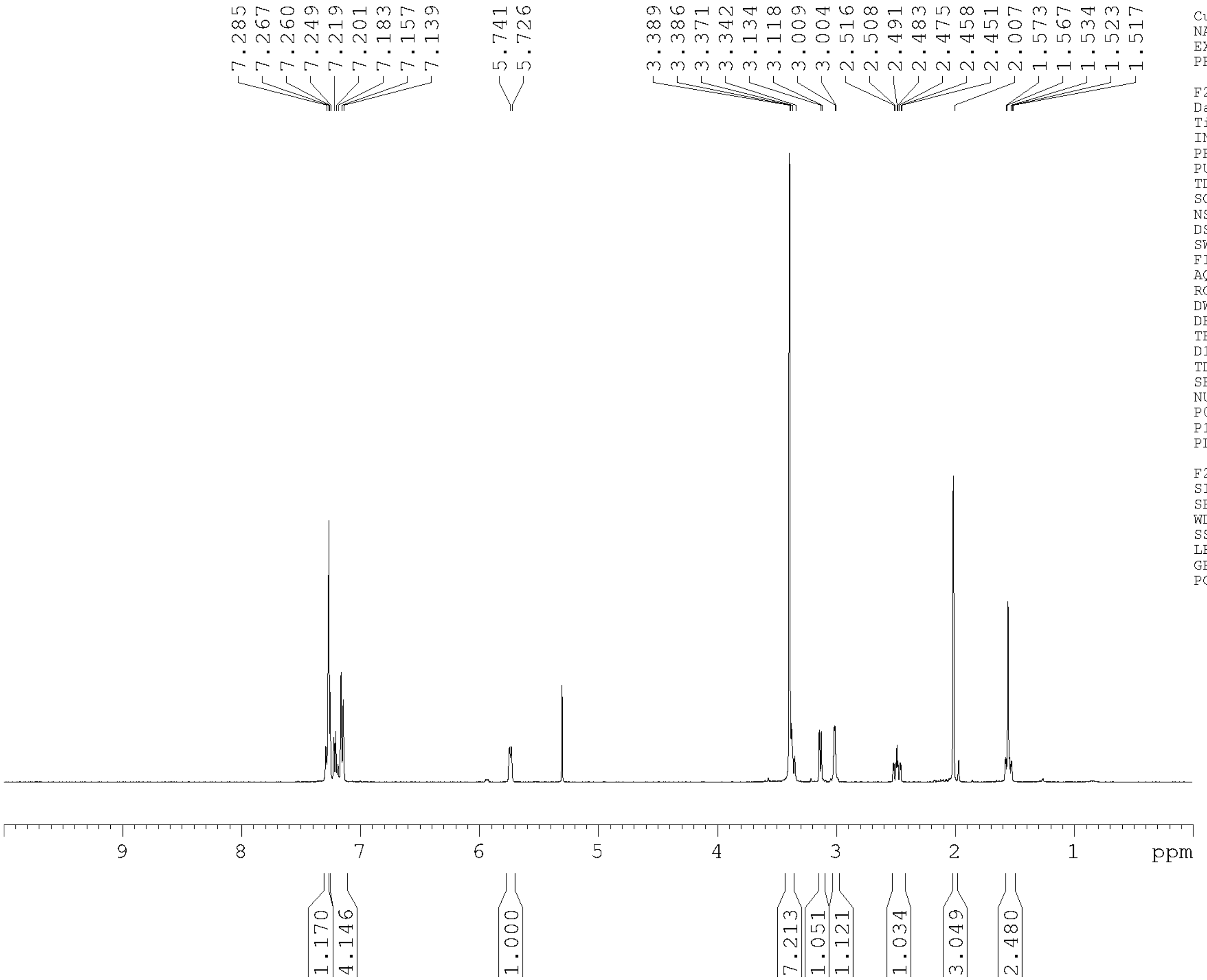


```
Current Data Parameters
NAME     20240202 SZ274-1
EXPNO                   1
PROCNO                  1

F2 - Acquisition Parameters
Date_            20240202
Time                18.15 h
INSTRUM  Avance NANOBAY
PROBHD   Z163739_0358 (
PULPROG              zg30
TD                  32768
SOLVENT             CDCl3
NS                     16
DS                      0
SWH              5882.353 Hz
FIDRES           0.359030 Hz
AQ              2.7852800 sec
RG                    101
DW                 85.000 usec
DE                   9.26 usec
TE                  296.8 K
D1             1.50000000 sec
TD0                     1
SFO1          400.1526010 MHz
NUC1                   1H
P0                   2.67 usec
P1                   8.00 usec
PLW1          21.10000038 W

F2 - Processing parameters
SI                  32768
SF            400.1500096 MHz
WDW                    EM
SSB                     0
LB                   0.10 Hz
GB                      0
PC                   1.00
```

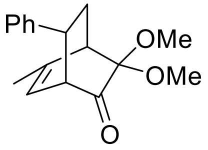

5q

^{1}H NMR of 5r

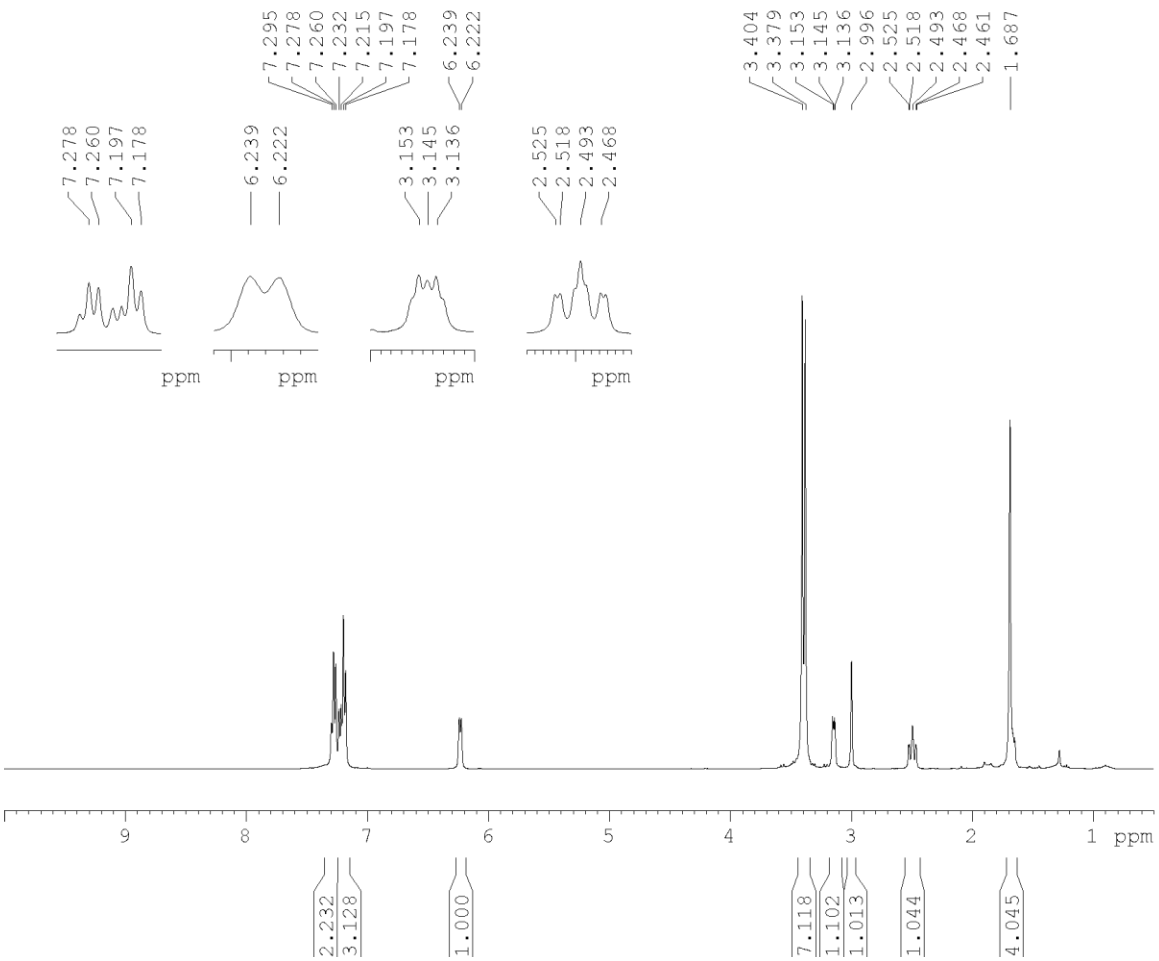


```
Current Data Parameters
NAME     20240611 DATA-3-Me-sty-DA
EXPNO                    1
PROCNO                   1

F2 - Acquisition Parameters
Date_            20240611
Time                23.32 h
INSTRUM  Avance NANOBAY
PROBHD   Z163739_0358 (
PULPROG              zg30
TD                  32768
SOLVENT             CDCl3
NS                     17
DS                      0
SWH              5882.353 Hz
FIDRES           0.359030 Hz
AQ              2.7852800 sec
RG                18.8616
DW                 85.000 usec
DE                   9.26 usec
TE                  294.5 K
D1            1.50000000 sec
TD0                     1
SFO1          400.1526010 MHz
NUC1                   1H
P0                   2.67 usec
P1                   8.00 usec
PLW1          21.10000038 W

F2 - Processing parameters
SI                  32768
SF            400.1500014 MHz
WDW                    EM
SSB                     0
LB                   0.10 Hz
GB                      0
PC                   1.00
```

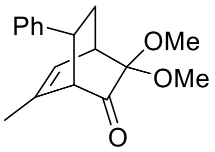

5r

^{1}H NMR of 5s

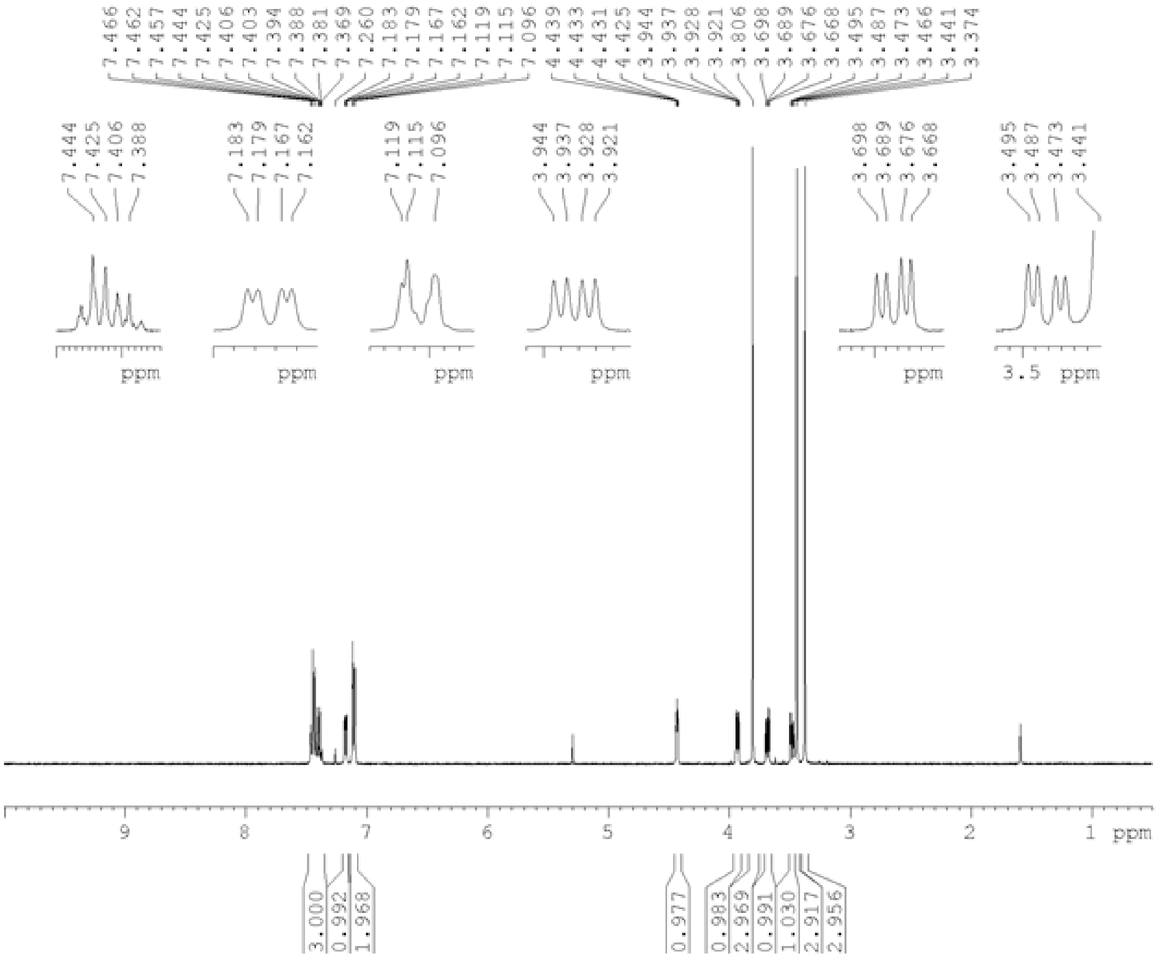


```
Current Data Parameters
NAME     20240520 Nana-rxn3-4-CO2Me DA col
EXPNO                 1
PROCNO                1

F2 - Acquisition Parameters
Date_          20240520
Time              21.15
INSTRUM           spect
PROBHD   5 mm BBO BB-1H
PULPROG            zg30
TD                32768
SOLVENT           CDCl3
NS                   17
DS                    0
SWH         6009.615 Hz
FIDRES      0.183399 Hz
AQ         2.7262976 sec
RG                  181
DW           83.200 usec
DE             6.50 usec
TE              295.4 K
D1       1.50000000 sec
TD0                   1

======== CHANNEL f1 ========
NUC1                 1H
P1            14.00 usec
PL1            -1.00 dB
PL1W      7.55784369 W
SFO1   400.1326010 MHz

F2 - Processing parameters
SI                32768
SF     400.1300100 MHz
WDW                  EM
SSB                   0
LB                 0 Hz
GB                    0
PC                 1.00
```

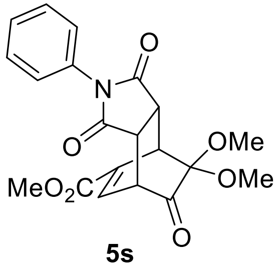

5s

^{1}H NMR of 5t

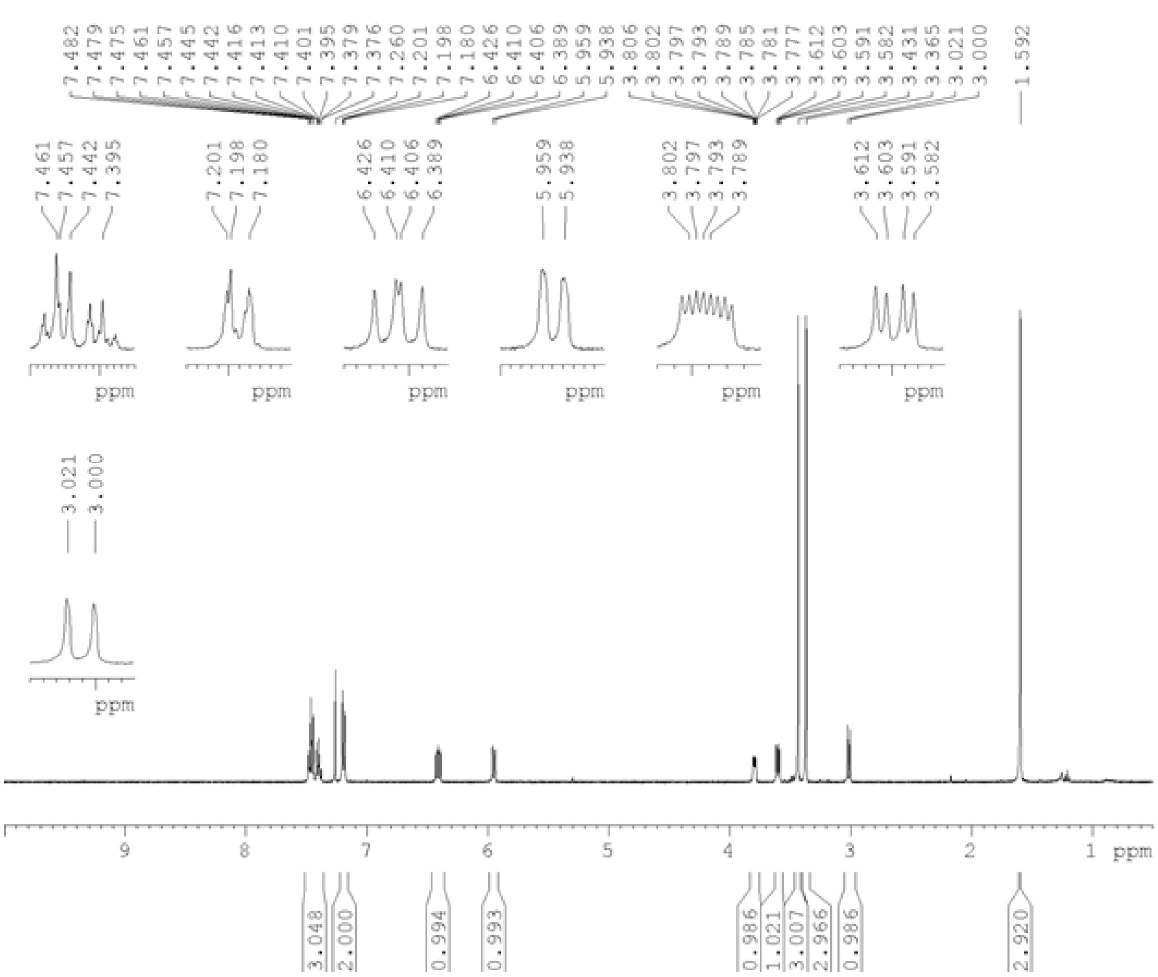

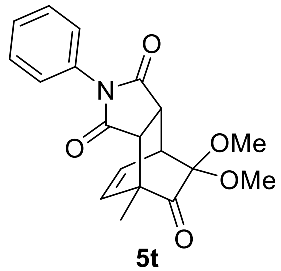

5t

^{1}H NMR of 5u

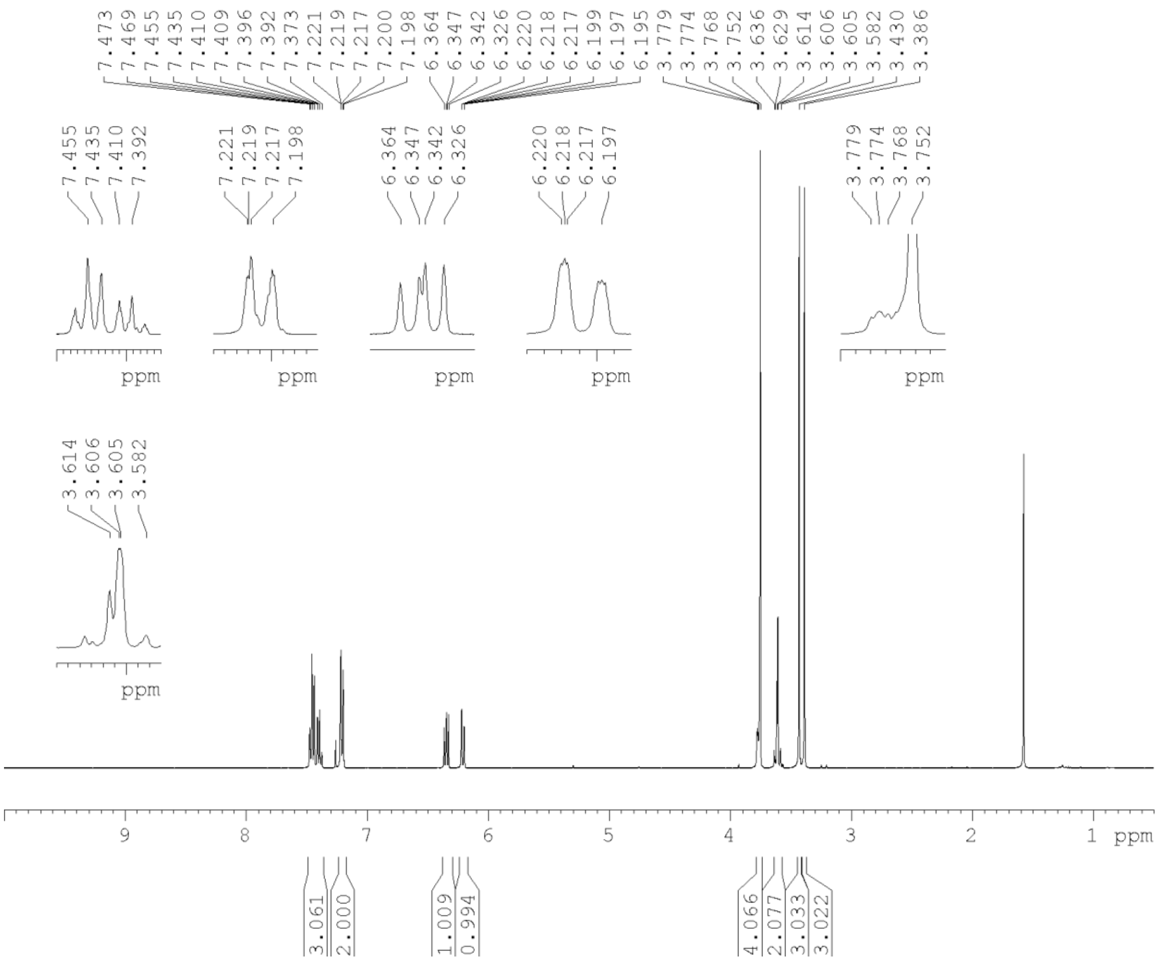


```
Current Data Parameters
NAME     20240606 DATA-2-OMe-DA
EXPNO                 1
PROCNO                1

F2 - Acquisition Parameters
Date_          20240606
Time              23.05
INSTRUM           spect
PROBHD   5 mm BBO BB-1H
PULPROG            zg30
TD                32768
SOLVENT           CDCl3
NS                   32
DS                    0
SWH            6009.615 Hz
FIDRES         0.183399 Hz
AQ            2.7262976 sec
RG                  256
DW               83.200 usec
DE                 6.50 usec
TE                295.4 K
D1           1.50000000 sec
TD0                   1

======== CHANNEL f1 ========
NUC1                 1H
P1                14.00 usec
PL1               -1.00 dB
PL1W         7.55784369 W
SFO1        400.1326010 MHz

F2 - Processing parameters
SI                32768
SF          400.1300102 MHz
WDW                  EM
SSB                   0
LB                    0 Hz
GB                    0
PC                 1.00
```

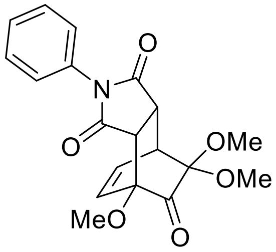

5u

^{1}H NMR of 5v

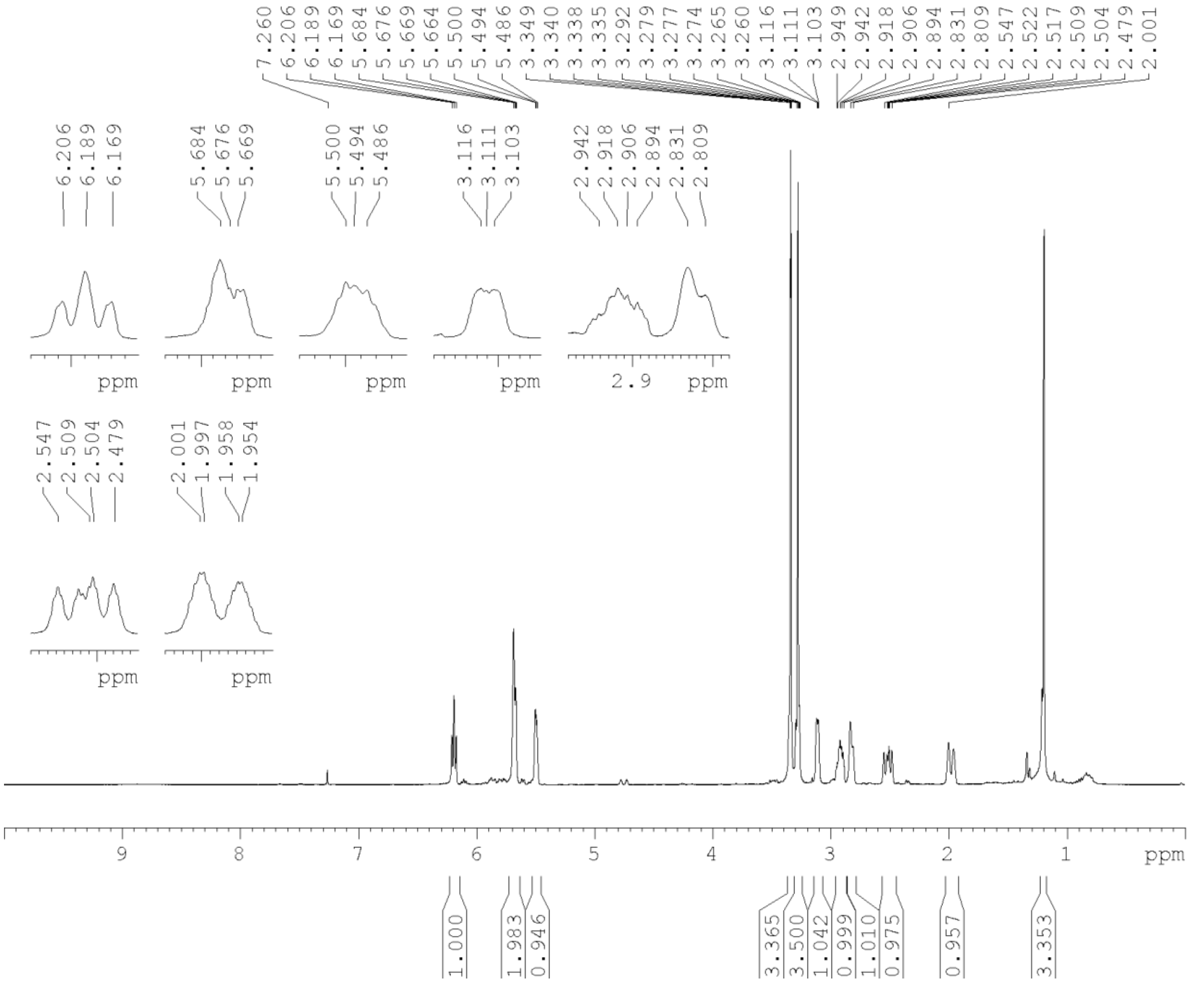

```
Current Data Parameters
NAME     20240829 DATA-2-Me-cp-DA
EXPNO                   1
PROCNO                  1

F2 - Acquisition Parameters
Date_          20240829
Time              15.54
INSTRUM           spect
PROBHD   5 mm BBO BB-1H
PULPROG            zg30
TD                32768
SOLVENT           CDCl3
NS                   33
DS                    0
SWH            6009.615 Hz
FIDRES         0.183399 Hz
AQ            2.7262976 sec
RG                 20.2
DW               83.200 usec
DE                 6.50 usec
TE                295.3 K
D1           1.50000000 sec
TD0                   1

======== CHANNEL f1 ========
NUC1                 1H
P1                14.00 usec
PL1               -1.00 dB
PL1W         7.55784369 W
SFO1        400.1326010 MHz

F2 - Processing parameters
SI                32768
SF          400.1300102 MHz
WDW                  EM
SSB                   0
LB                    0 Hz
GB                    0
PC                 1.00
```

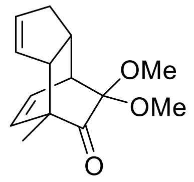

5v

^{1}H NMR of 5w

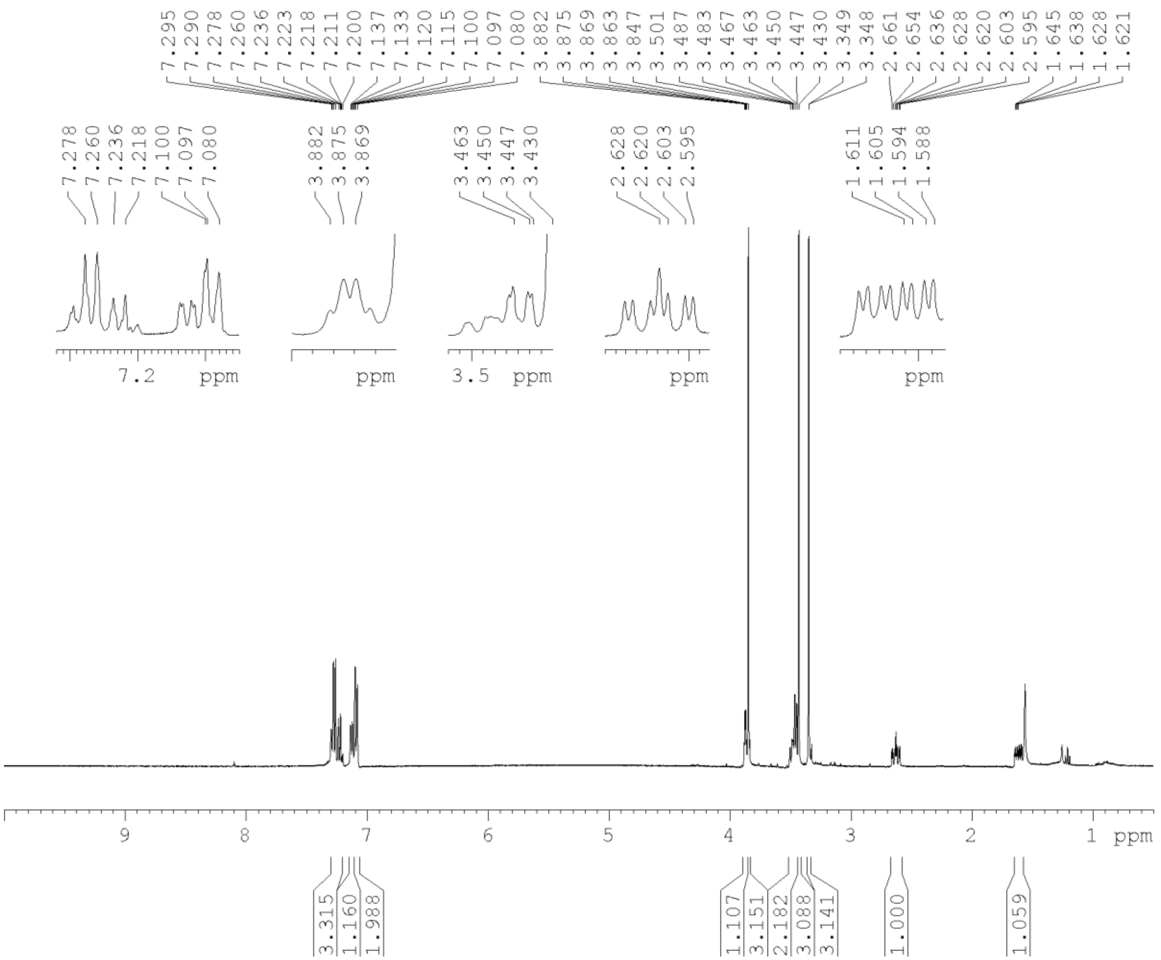

```
Current Data Parameters
NAME     20240627 DATA-4-CO2Me-sty-DA
EXPNO                   1
PROCNO                  1

F2 - Acquisition Parameters
Date_            20240627
Time                22.44
INSTRUM             spect
PROBHD   5 mm BBO BB-1H
PULPROG              zg30
TD                  32768
SOLVENT             CDCl3
NS                     31
DS                      0
SWH              6009.615 Hz
FIDRES           0.183399 Hz
AQ              2.7262976 sec
RG                    181
DW                 83.200 usec
DE                   6.50 usec
TE                  295.6 K
D1             1.50000000 sec
TD0                     1

======== CHANNEL f1 ========
NUC1                   1H
P1                  14.00 usec
PL1                 -1.00 dB
PL1W           7.55784369 W
SFO1          400.1326010 MHz

F2 - Processing parameters
SI                  32768
SF            400.1300099 MHz
WDW                    EM
SSB                     0
LB                      0 Hz
GB                      0
PC                   1.00
```

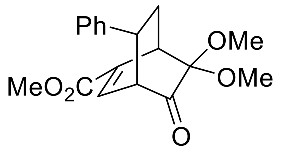

5w

^{1}H NMR of 5x

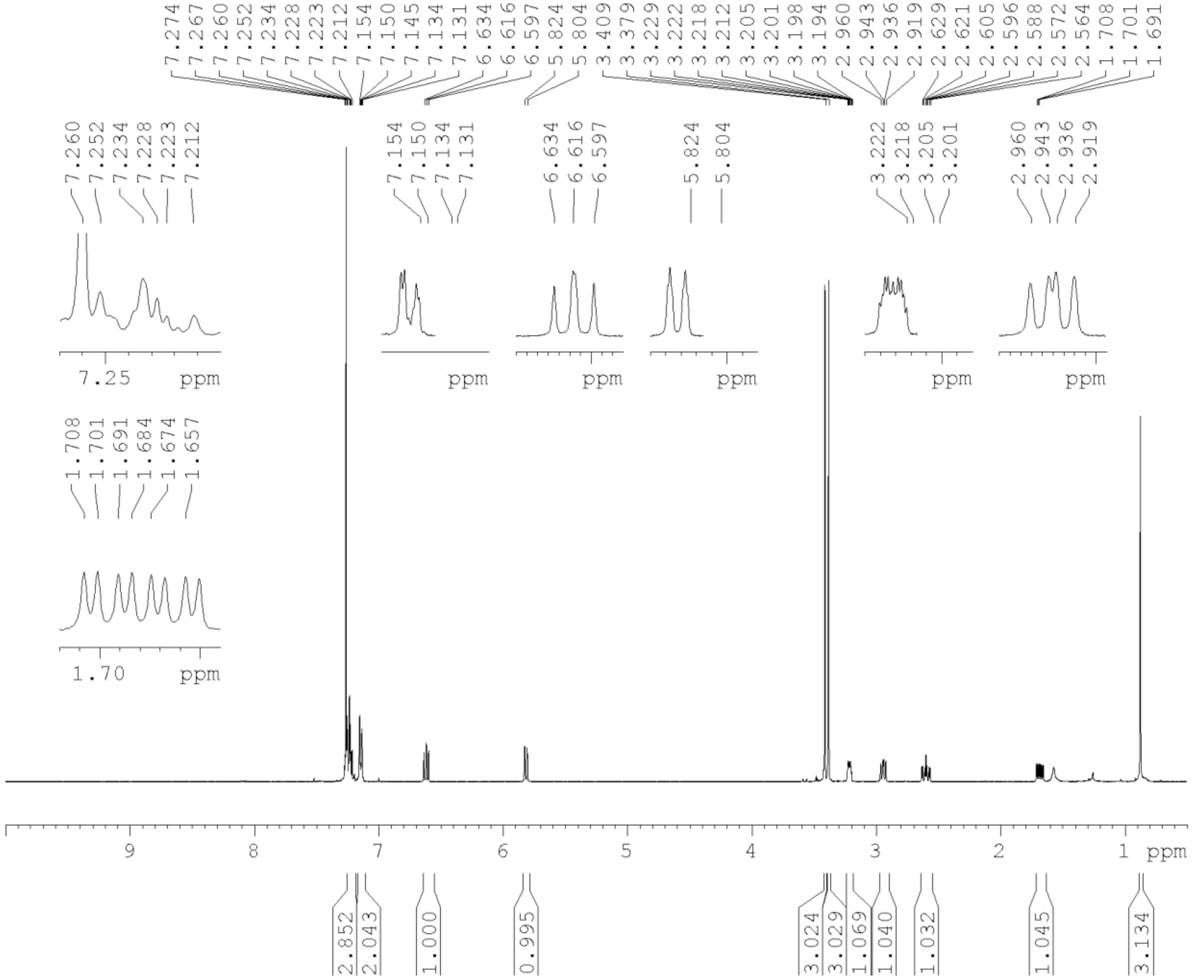

Current Data Parameters
NAME 20240806 DATA-2-Me-sty-DA
EXPNO 1
PROCNO 1

F2 - Acquisition Parameters
Date_ 20240806
Time 20.59 h
INSTRUM Avance NANOBAY
PROBHD Z163739_0358 (
PULPROG zg30
TD 32768
SOLVENT CDCl3
NS 24
DS 0
SWH 5882.353 Hz
FIDRES 0.359030 Hz
AQ 2.7852800 sec
RG 101
DW 85.000 usec
DE 9.26 usec
TE 294.1 K
D1 1.50000000 sec
TD0 1
SFO1 400.1526010 MHz
NUC1 1H
P0 2.67 usec
P1 8.00 usec
PLW1 21.10000038 W

F2 - Processing parameters
SI 32768
SF 400.1500097 MHz
WDW EM
SSB 0
LB 0.10 Hz
GB 0
PC 1.00

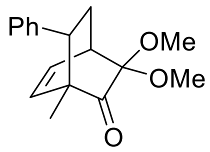

5x

^{1}H NMR of 6a

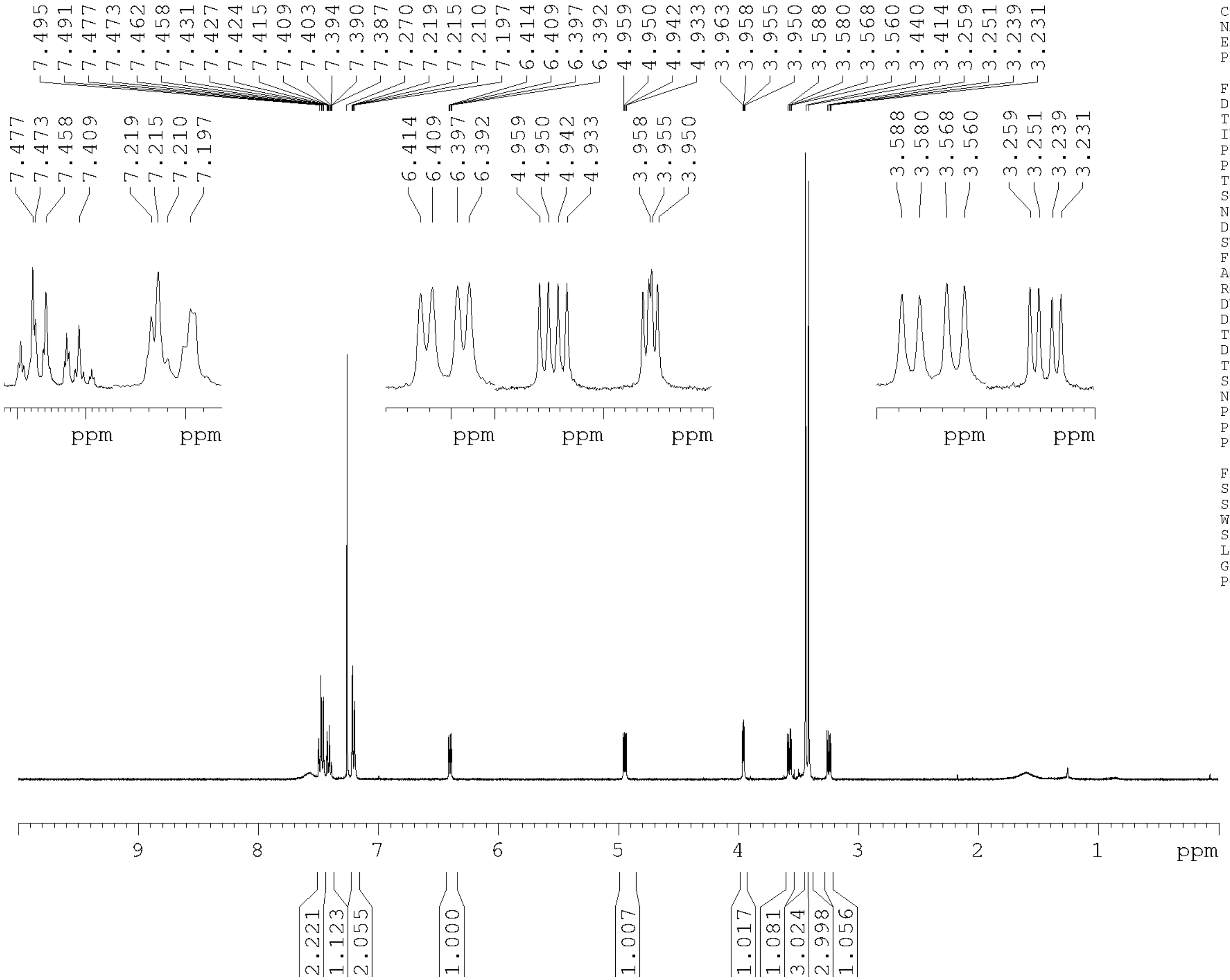

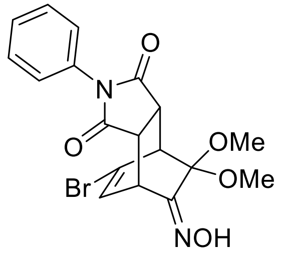

6a

^{1}H NMR of 6c

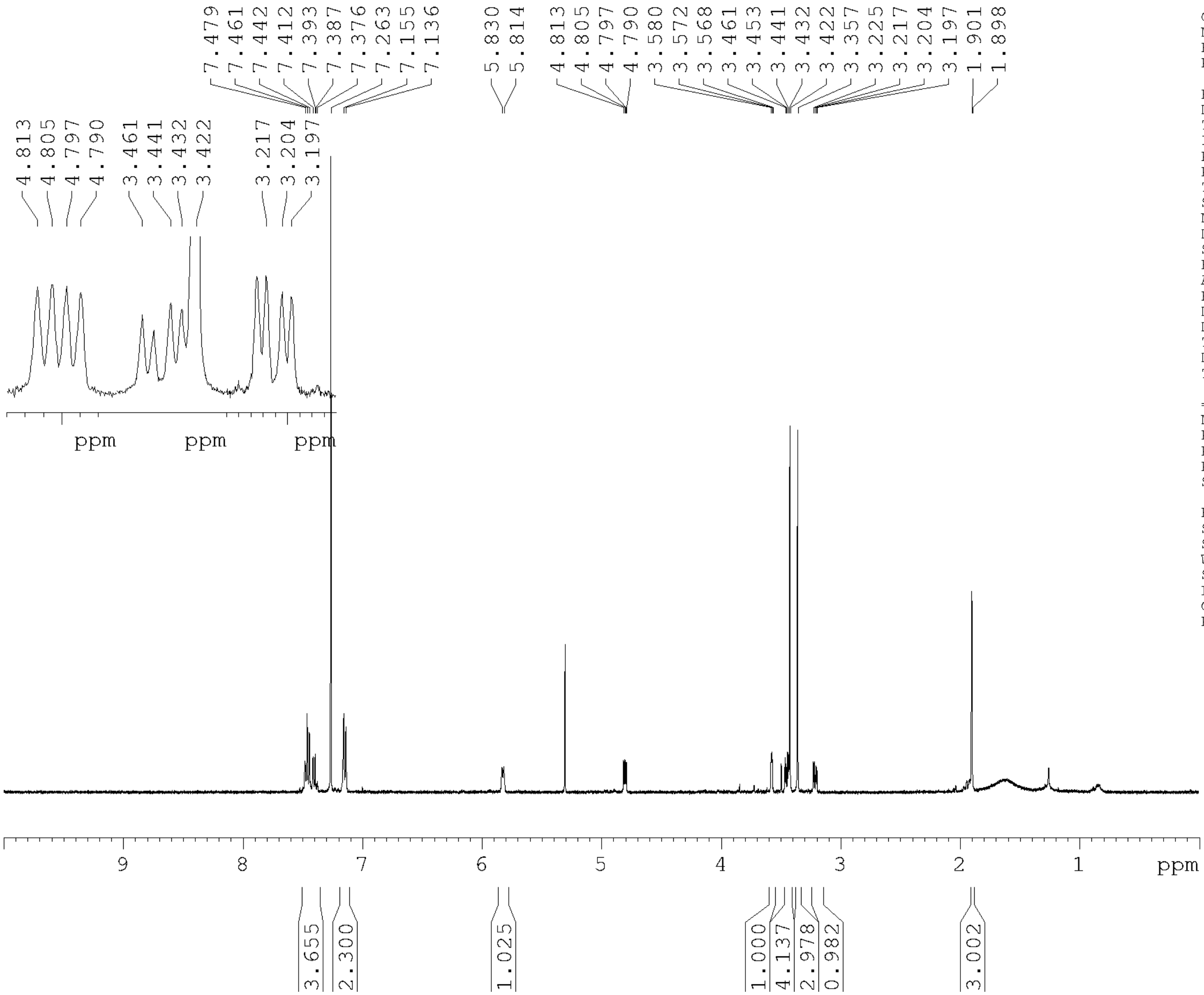

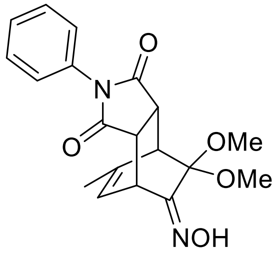

6c

^{1}H NMR of 6d

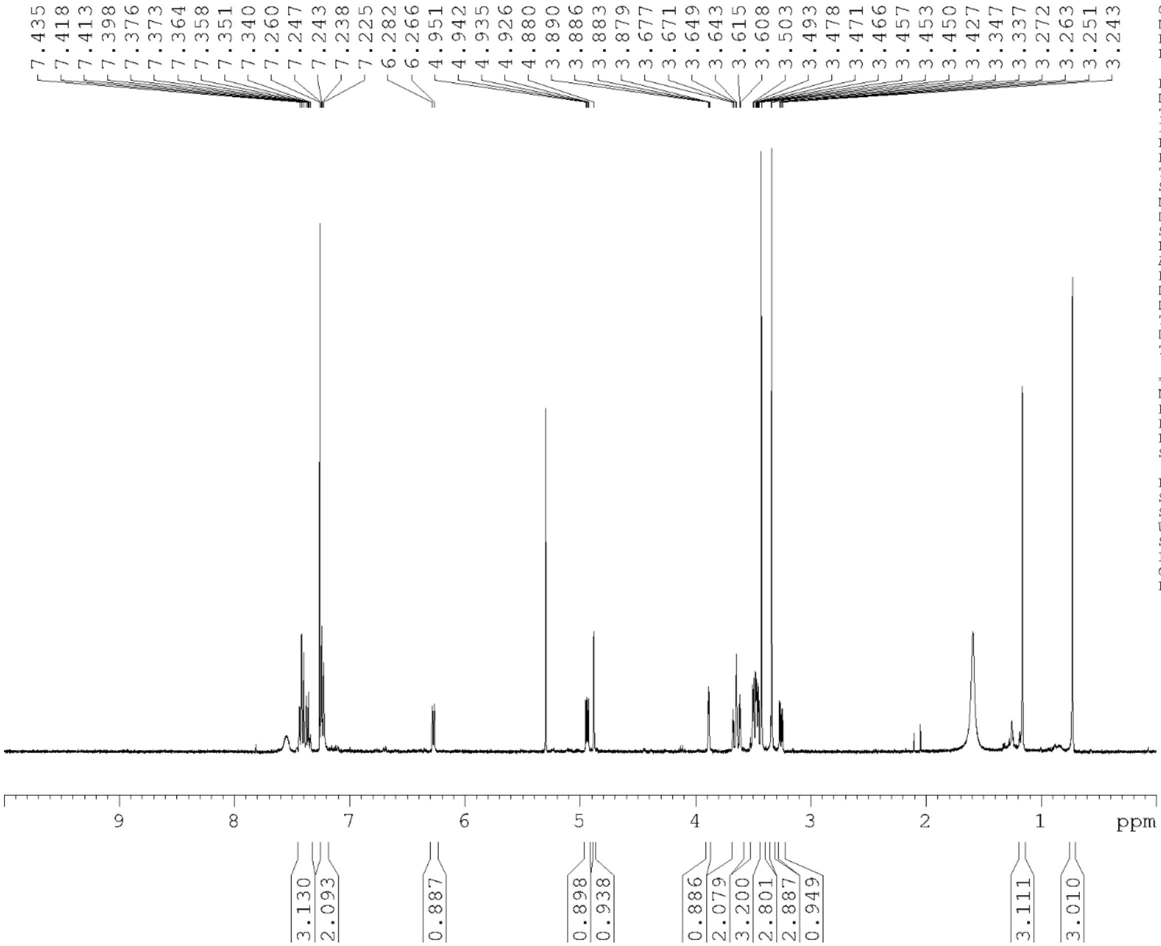


```
Current Data Parameters
NAME      20230509 SZ112-C
EXPNO                    1
PROCNO                   1

F2 - Acquisition Parameters
Date_             20230509
Time                 16.14
INSTRUM              spect
PROBHD   5 mm BBO BB-1H
PULPROG               zg30
TD                   32768
SOLVENT              CDCl3
NS                      16
DS                       0
SWH               6009.615 Hz
FIDRES            0.183399 Hz
AQ               2.7262976 sec
RG                     287
DW                  83.200 usec
DE                    6.50 usec
TE                   292.8 K
D1              1.50000000 sec
TD0                      1

======== CHANNEL f1 ========
NUC1                    1H
P1                   14.00 usec
PL1                  -1.00 dB
PL1W            7.55784369 W
SFO1           400.1326010 MHz

F2 - Processing parameters
SI                   32768
SF             400.1300100 MHz
WDW                     EM
SSB                      0
LB                       0 Hz
GB                       0
PC                    1.00
```

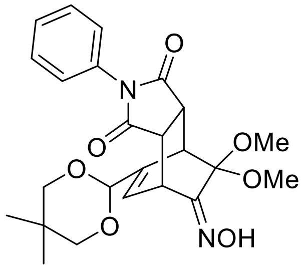

6d

^{1}H NMR of 6e

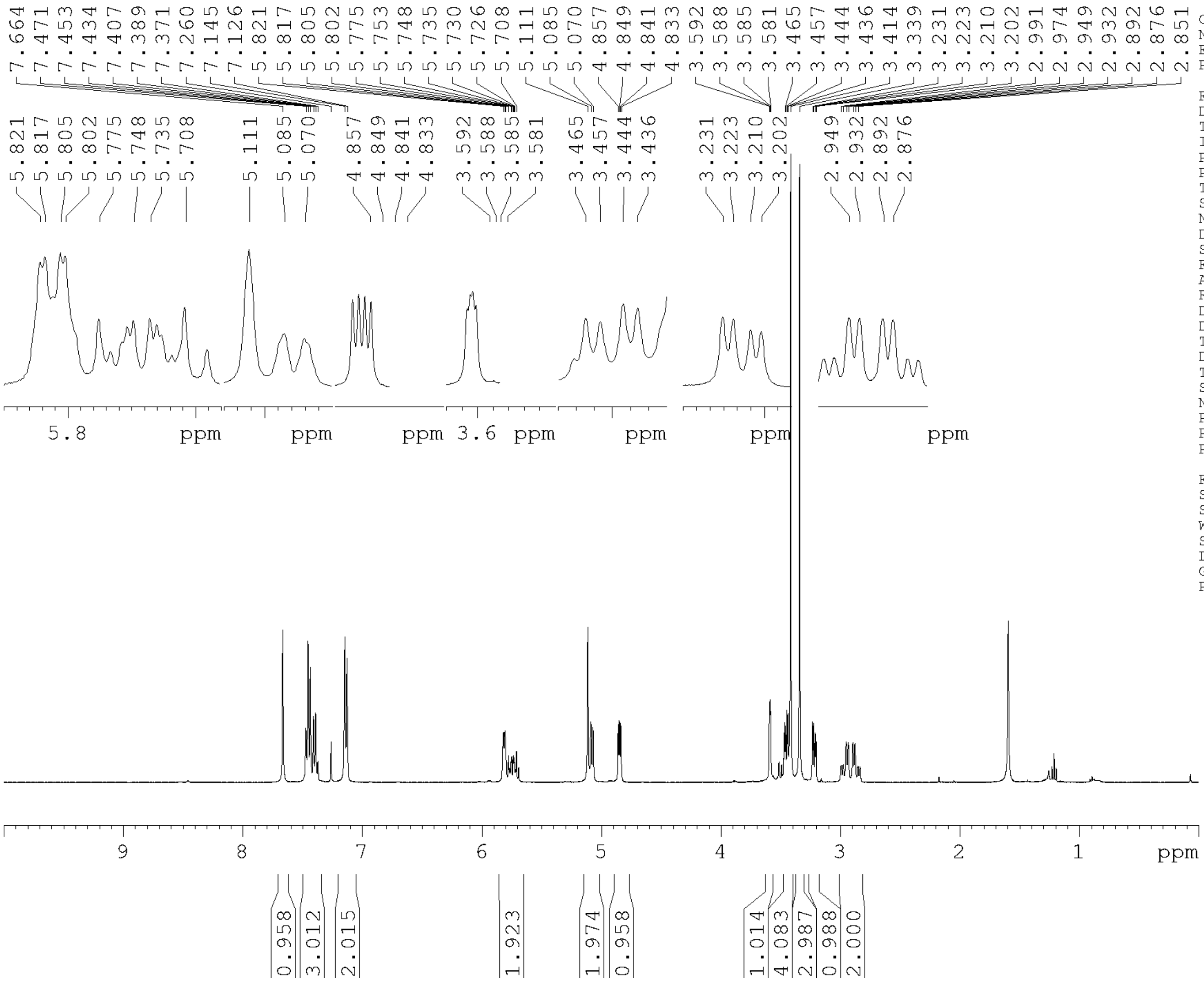

Current Data Parameters
NAME 20240521 4-allyl-O
EXPNO 1
PROCNO 1

F2 - Acquisition Parameters
Date_ 20240522
Time 7.40 h
INSTRUM Avance NANOBAY
PROBHD Z163739_0358 (
PULPROG zg30
TD 32768
SOLVENT CDCl3
NS 16
DS 0
SWH 5882.353 Hz
FIDRES 0.359030 Hz
AQ 2.7852800 sec
RG 101
DW 85.000 usec
DE 9.26 usec
TE 294.9 K
D1 1.50000000 sec
TD0 1
SFO1 400.1526010 MHz
NUC1 1H
P0 2.67 usec
P1 8.00 usec
PLW1 21.10000038 W

F2 - Processing parameters
SI 32768
SF 400.1500100 MHz
WDW EM
SSB 0
LB 0.10 Hz
GB 0
PC 1.00

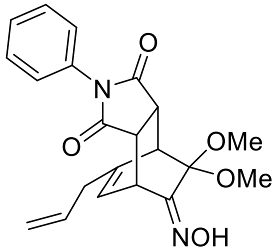

6e

^{1}H NMR of 6f

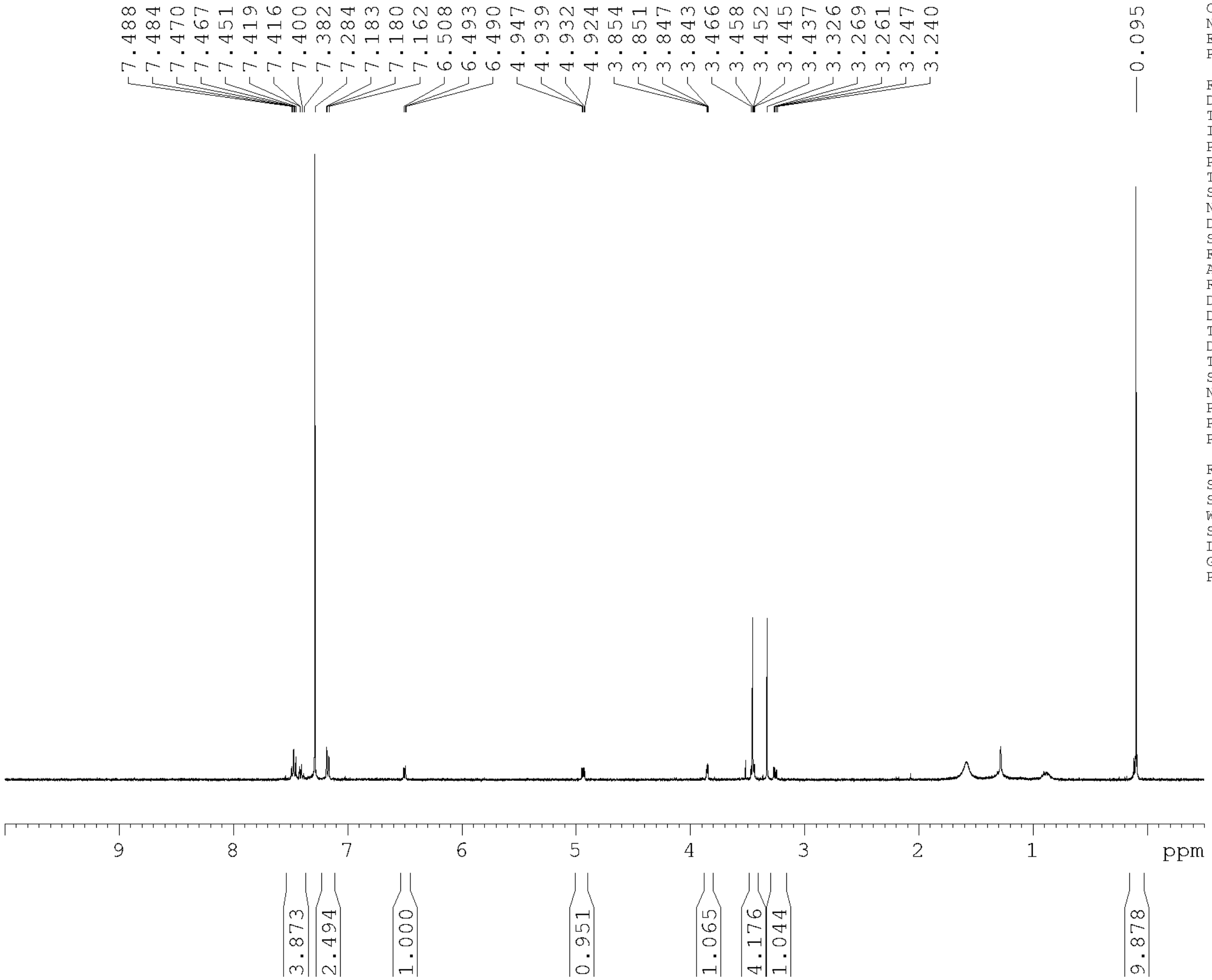

Current Data Parameters
NAME 20230712 4-TMS-D
EXPNO 2
PROCNO 1

F2 - Acquisition Parameters
Date_ 20230712
Time 14.17 h
INSTRUM Avance NANOBAY
PROBHD Z163739_0358 (
PULPROG zg30
TD 32768
SOLVENT CDCl3
NS 1
DS 0
SWH 5882.353 Hz
FIDRES 0.359030 Hz
AQ 2.7852800 sec
RG 101
DW 85.000 usec
DE 9.26 usec
TE 297.5 K
D1 1.50000000 sec
TD0 1
SFO1 400.1526010 MHz
NUC1 1H
P0 2.67 usec
P1 8.00 usec
PLW1 21.10000038 W

F2 - Processing parameters
SI 32768
SF 400.1500000 MHz
WDW EM
SSB 0
LB 0.10 Hz
GB 0
PC 1.00

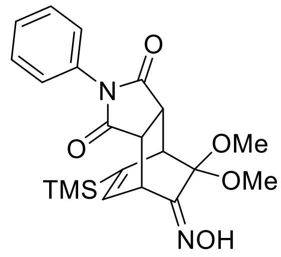

6f

^{1}H NMR of 6g

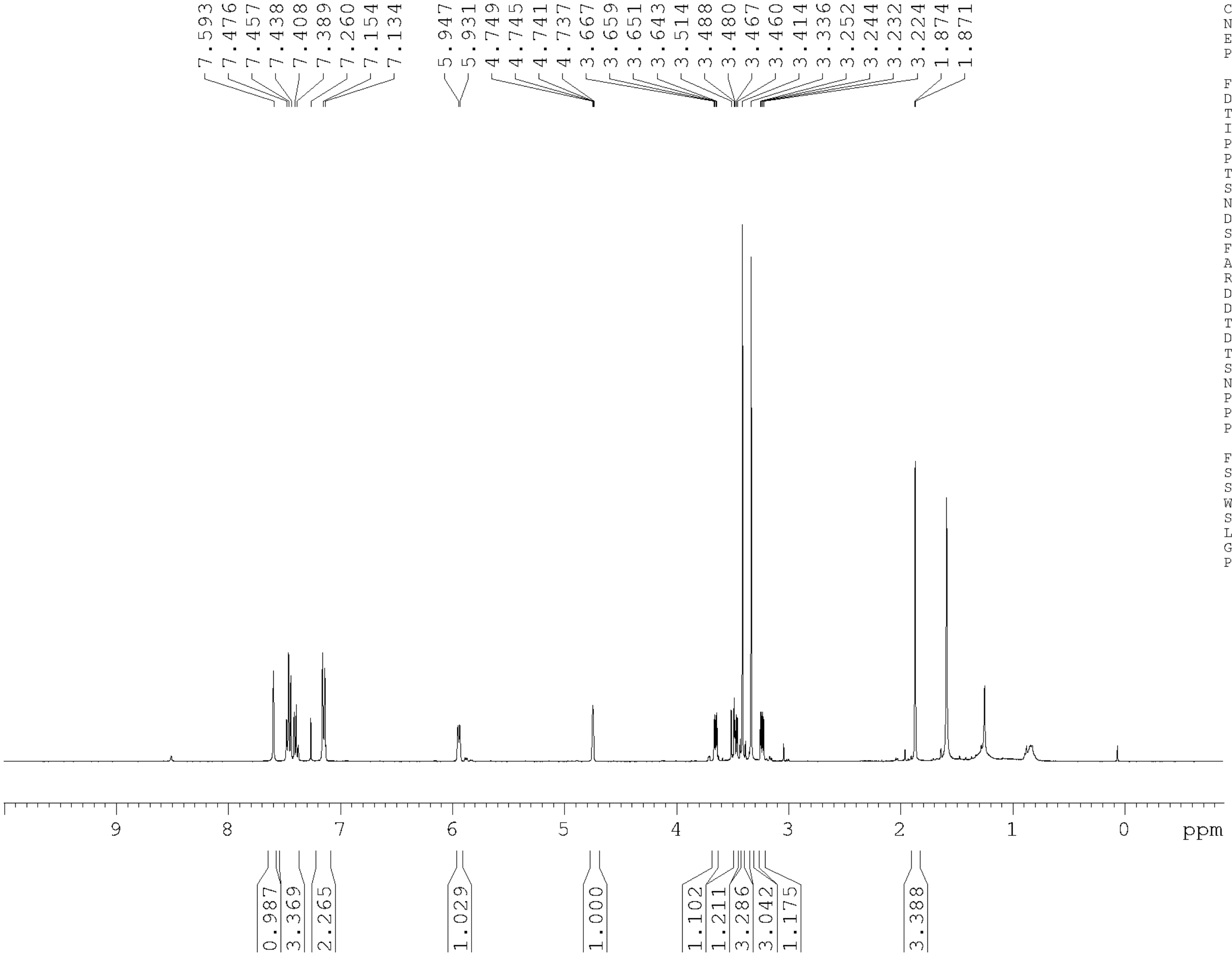


```
Current Data Parameters
NAME     20240801 3-Me-O
EXPNO                  1
PROCNO                 1

F2 - Acquisition Parameters
Date_           20240801
Time                9.02 h
INSTRUM  Avance NANOBAY
PROBHD   Z163739_0358 (
PULPROG             zg30
TD                 32768
SOLVENT            CDCl3
NS                    16
DS                     0
SWH             5882.353 Hz
FIDRES          0.359030 Hz
AQ             2.7852800 sec
RG                   101
DW                85.000 usec
DE                  9.26 usec
TE                 296.1 K
D1            1.50000000 sec
TD0                    1
SFO1         400.1526010 MHz
NUC1                  1H
P0                  2.67 usec
P1                  8.00 usec
PLW1         21.10000038 W

F2 - Processing parameters
SI                 32768
SF           400.1500100 MHz
WDW                   EM
SSB                    0
LB                  0.10 Hz
GB                     0
PC                  1.00
```

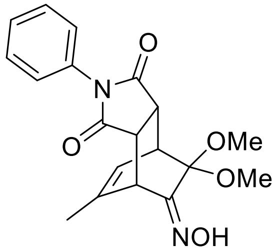

6g

^{1}H NMR of 6h

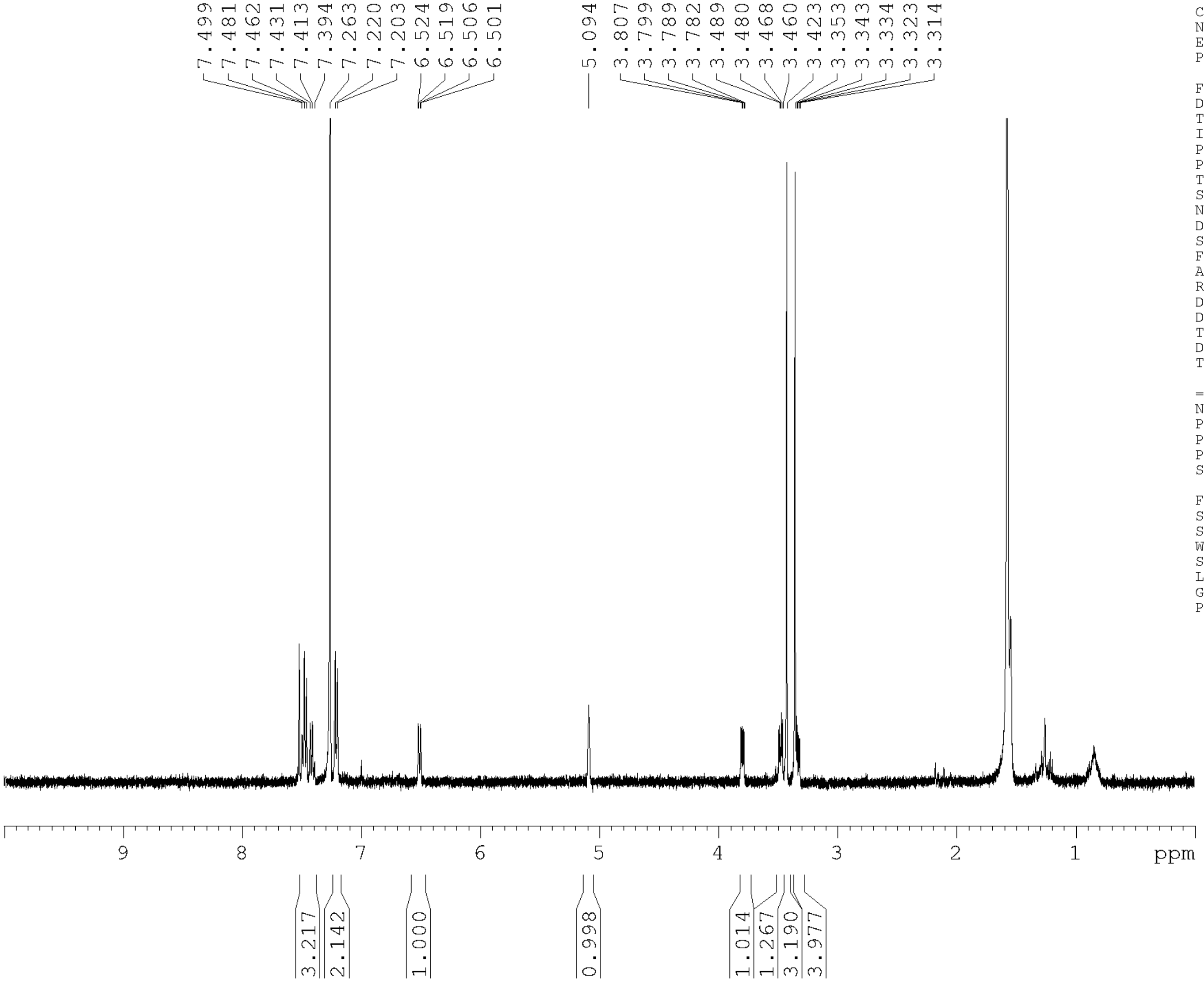


```
Current Data Parameters
NAME     20230901 3-Br-O
EXPNO                  1
PROCNO                 1

F2 - Acquisition Parameters
Date_           20230901
Time               18.44
INSTRUM            spect
PROBHD   5 mm BBO BB-1H
PULPROG             zg30
TD                 32768
SOLVENT            CDCl3
NS                    16
DS                     0
SWH             6009.615 Hz
FIDRES          0.183399 Hz
AQ             2.7262976 sec
RG                   322
DW                83.200 usec
DE                  6.50 usec
TE                 295.0 K
D1            1.50000000 sec
TD0                    1

======== CHANNEL f1 ========
NUC1                  1H
P1                 14.00 usec
PL1                -1.00 dB
PL1W          7.55784369 W
SFO1         400.1326010 MHz

F2 - Processing parameters
SI                 32768
SF           400.1300087 MHz
WDW                   EM
SSB                    0
LB                     0 Hz
GB                     0
PC                  1.00
```

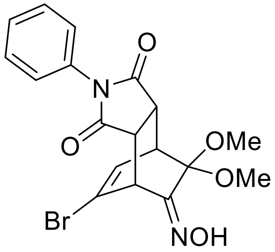

6h

^{1}H NMR of 6i

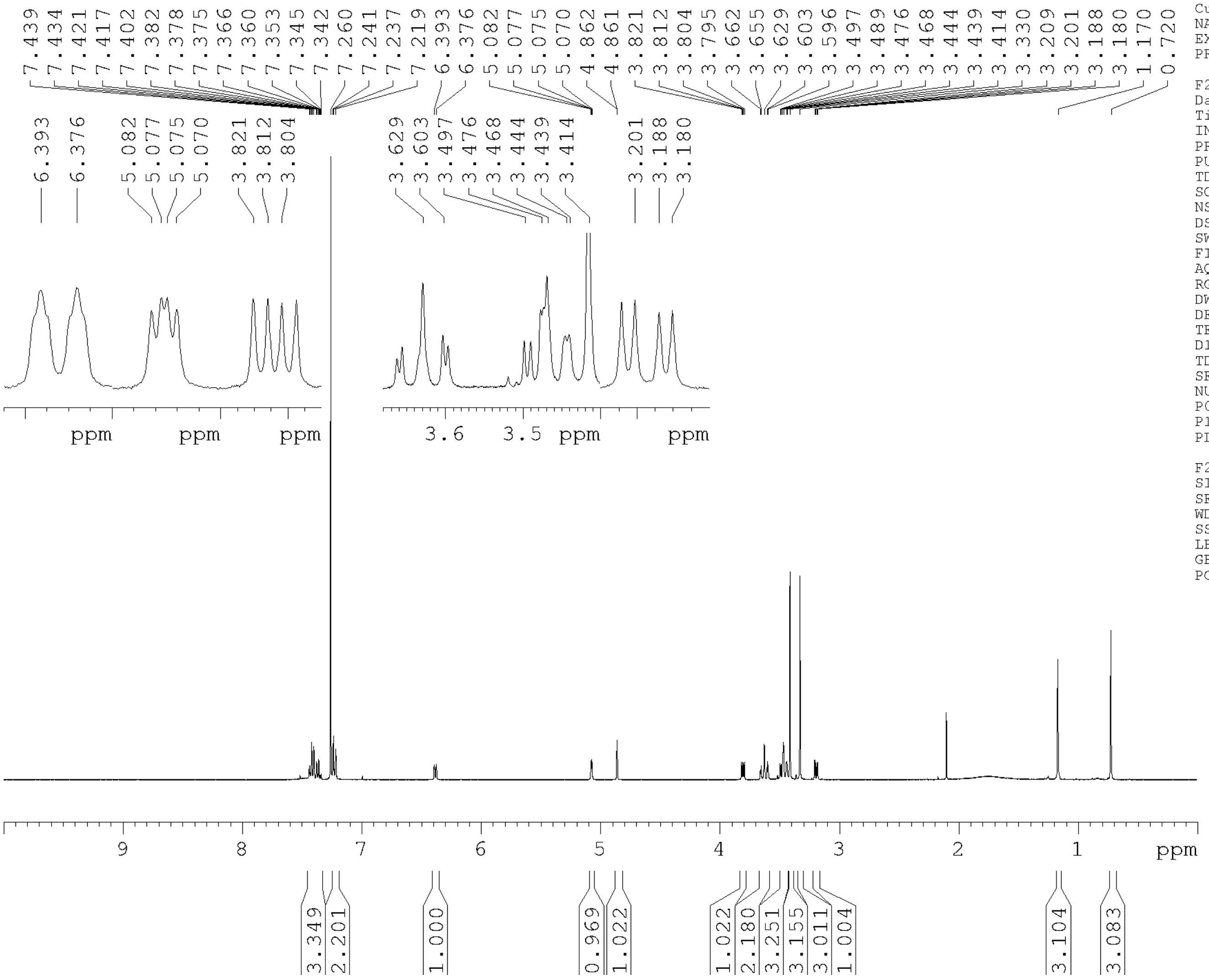

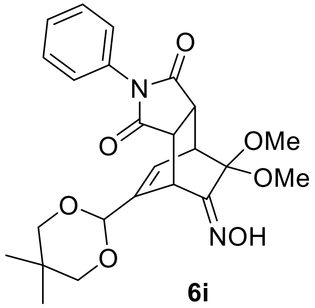

6i

^{1}H NMR of 6j

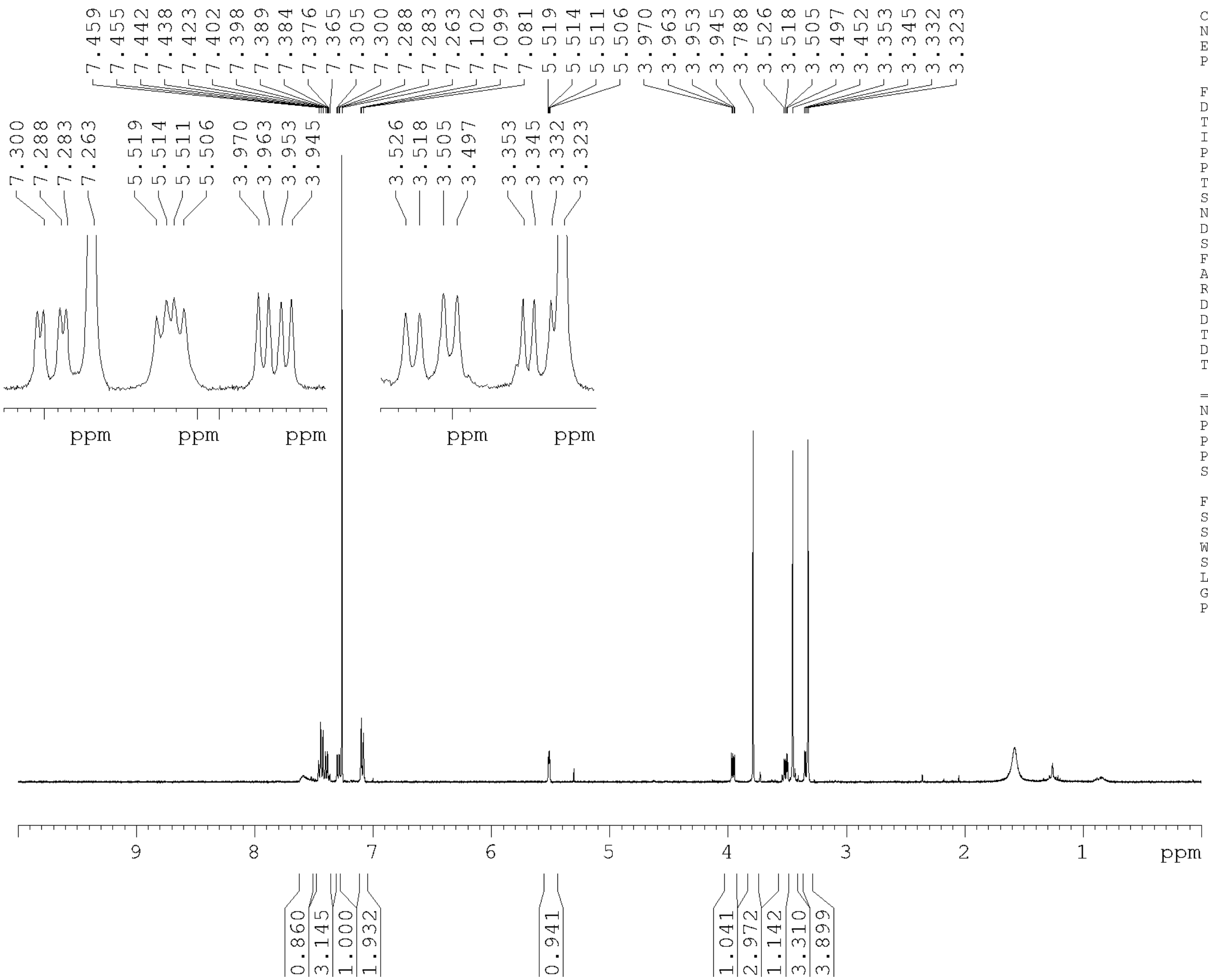


```
Current Data Parameters
NAME     20230818 3-CO2Me-O-3
EXPNO                   1
PROCNO                  1

F2 - Acquisition Parameters
Date_            20230818
Time                22.23
INSTRUM             spect
PROBHD   5 mm BBO BB-1H
PULPROG              zg30
TD                  32768
SOLVENT             CDCl3
NS                     16
DS                      0
SWH              6009.615 Hz
FIDRES           0.183399 Hz
AQ              2.7262976 sec
RG                    362
DW                 83.200 usec
DE                   6.50 usec
TE                  294.9 K
D1             1.50000000 sec
TD0                     1

======== CHANNEL f1 ========
NUC1                   1H
P1                  14.00 usec
PL1                 -1.00 dB
PL1W           7.55784369 W
SFO1          400.1326010 MHz

F2 - Processing parameters
SI                  32768
SF            400.1300087 MHz
WDW                    EM
SSB                     0
LB                      0 Hz
GB                      0
PC                   1.00
```

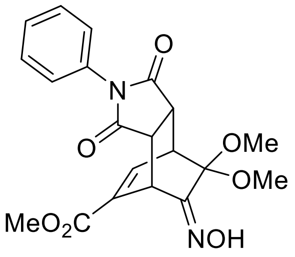

6j

^{1}H NMR of 6k

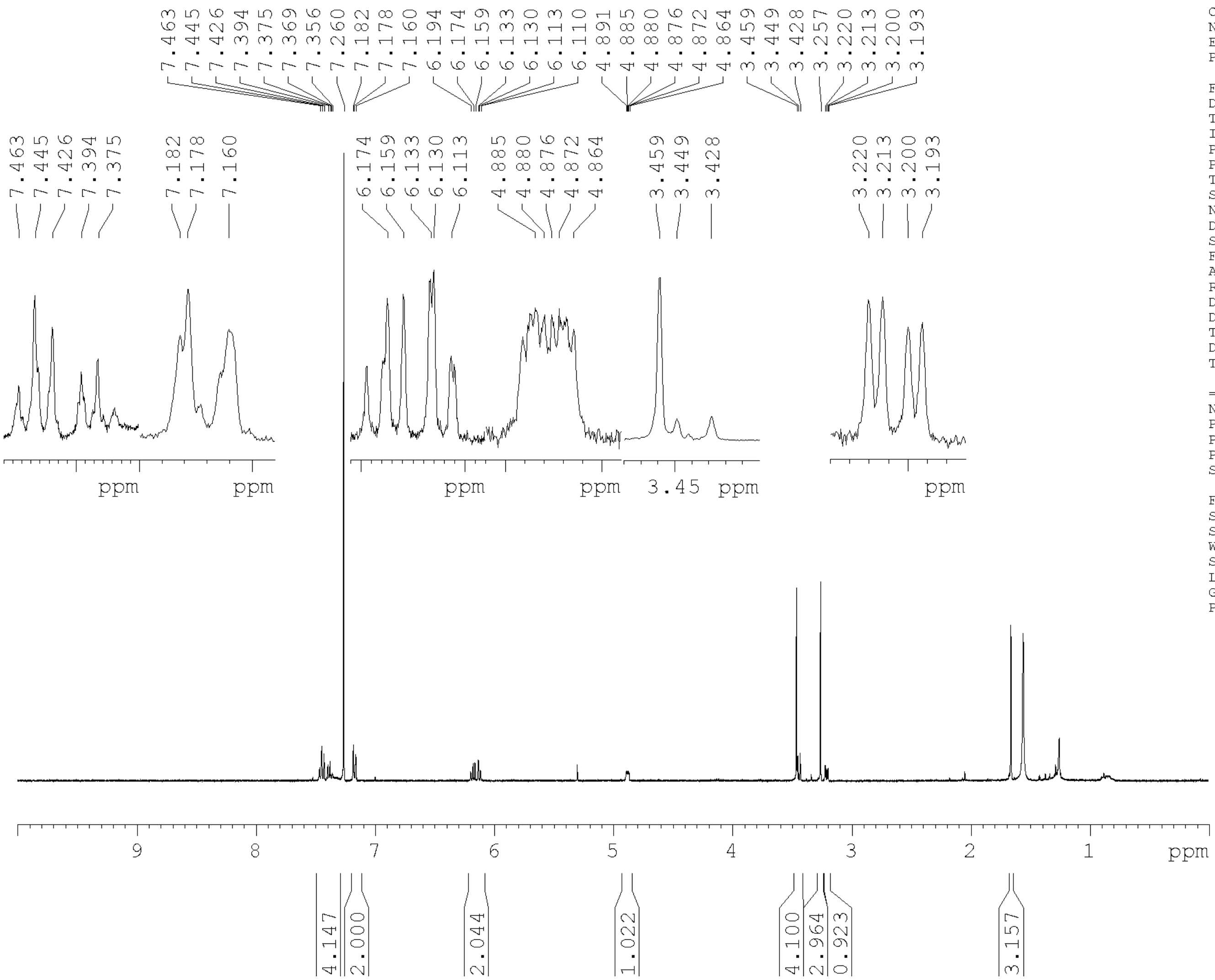

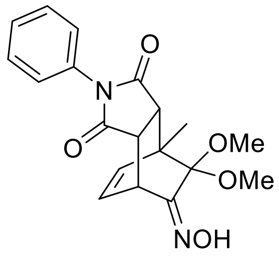

6k

^{1}H NMR of 6l

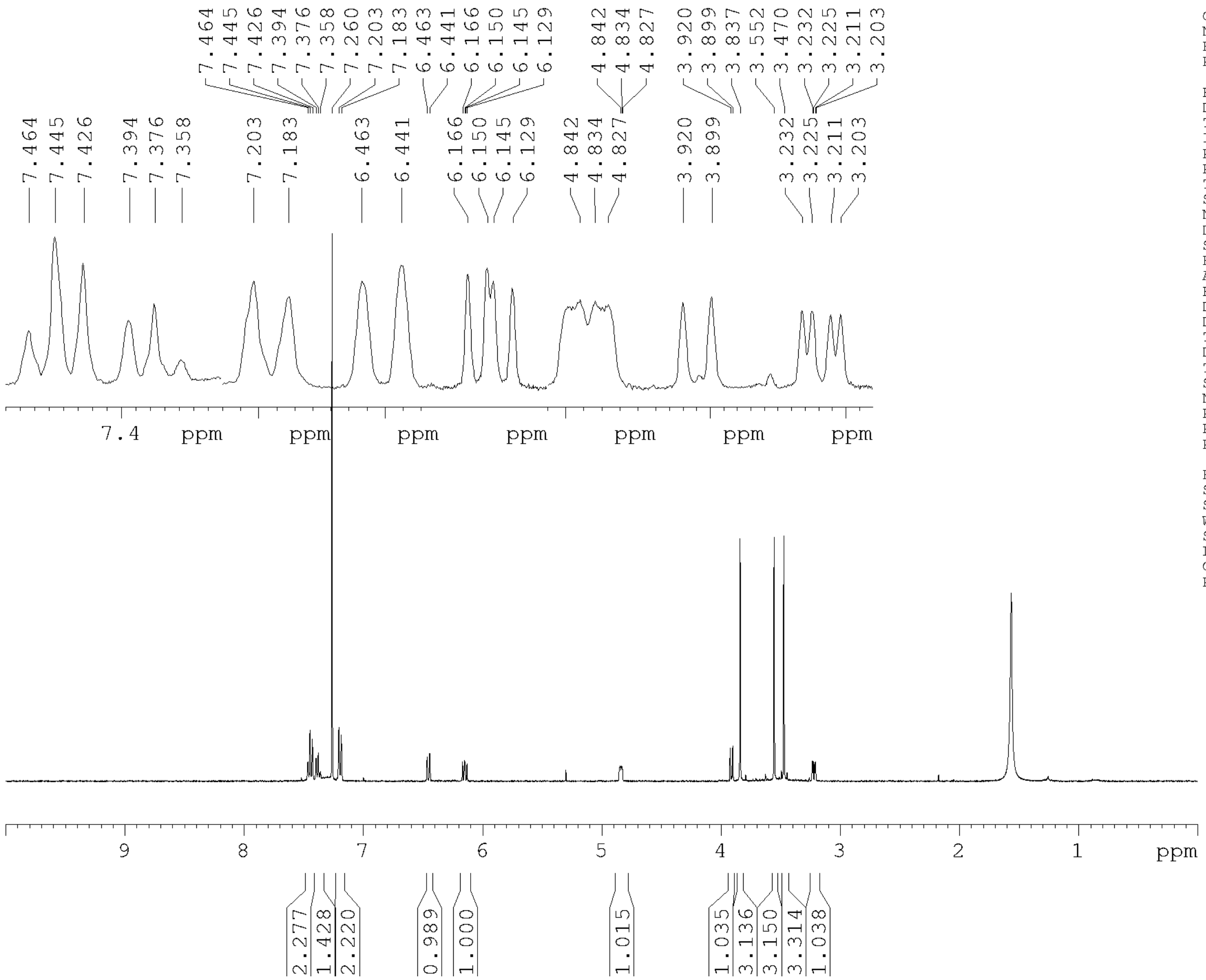


```
Current Data Parameters
NAME     20240521 5-OMe-O
EXPNO                   1
PROCNO                  1

F2 - Acquisition Parameters
Date_            20240521
Time                17.10 h
INSTRUM  Avance NANOBAY
PROBHD   Z163739_0358 (
PULPROG              zg30
TD                  32768
SOLVENT             CDCl3
NS                     16
DS                      0
SWH              5882.353 Hz
FIDRES           0.359030 Hz
AQ              2.7852800 sec
RG                    101
DW                 85.000 usec
DE                   9.26 usec
TE                  295.8 K
D1             1.50000000 sec
TD0                     1
SFO1          400.1526010 MHz
NUC1                   1H
P0                   2.67 usec
P1                   8.00 usec
PLW1          21.10000038 W

F2 - Processing parameters
SI                  32768
SF            400.1500097 MHz
WDW                    EM
SSB                     0
LB                   0.10 Hz
GB                      0
PC                   1.00
```

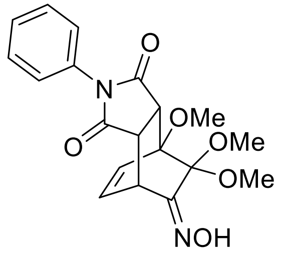

6l

^{1}H NMR of 6m

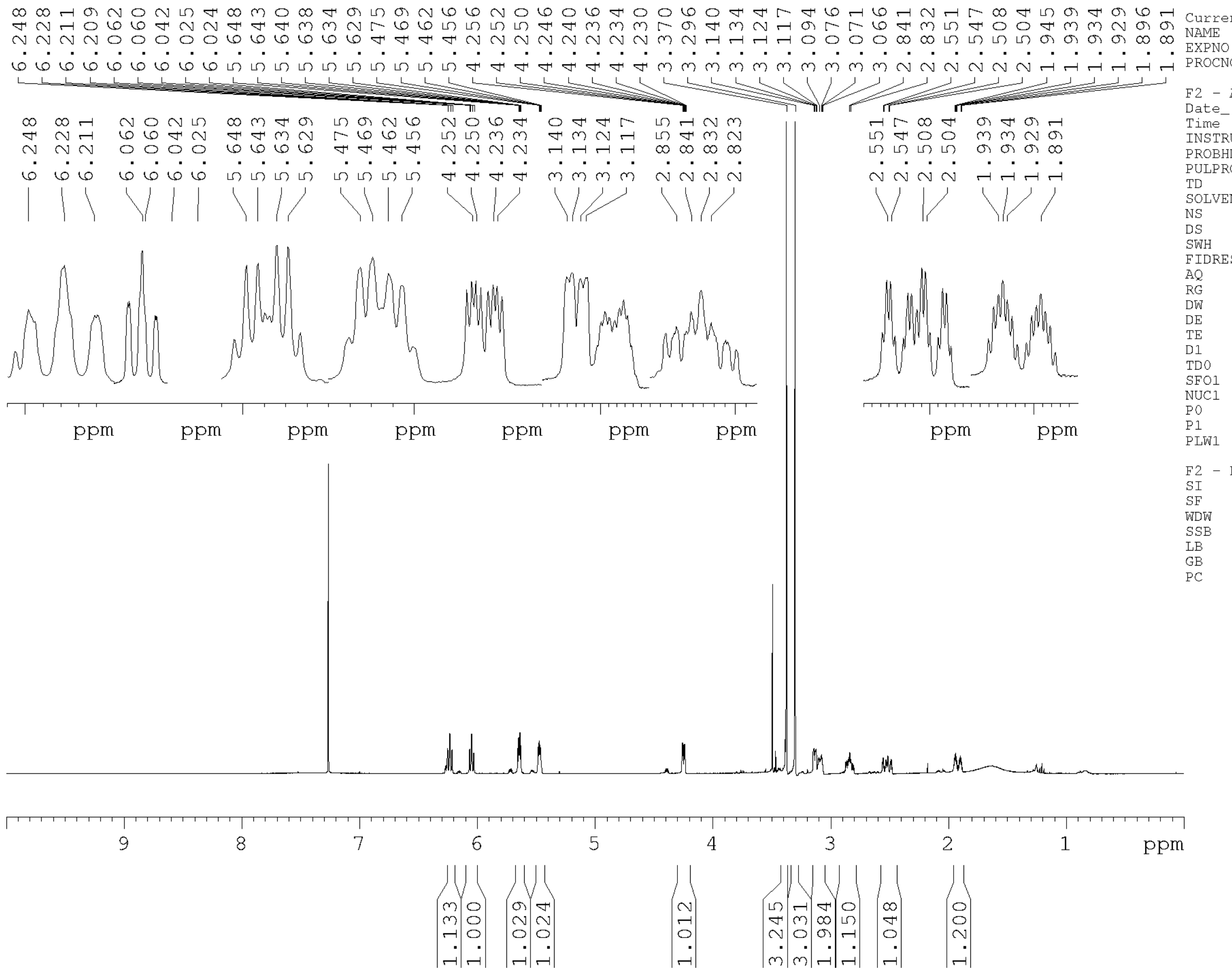

Current Data Parameters
NAME 20240508 NS-OC
EXPNO 1
PROCNO 1

F2 - Acquisition Parameters
Date_ 20240508
Time 20.14 h
INSTRUM Avance NANOBAY
PROBHD Z163739_0358 (
PULPROG zg30
TD 32768
SOLVENT CDCl3
NS 16
DS 0
SWH 5882.353 Hz
FIDRES 0.359030 Hz
AQ 2.7852800 sec
RG 101
DW 85.000 usec
DE 9.26 usec
TE 296.4 K
D1 1.50000000 sec
TD0 1
SFO1 400.1526010 MHz
NUC1 1H
P0 2.67 usec
P1 8.00 usec
PLW1 21.10000038 W

F2 - Processing parameters
SI 32768
SF 400.1500095 MHz
WDW EM
SSB 0
LB 0.10 Hz
GB 0
PC 1.00

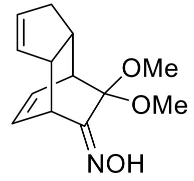

6m

^{1}H NMR of 6o

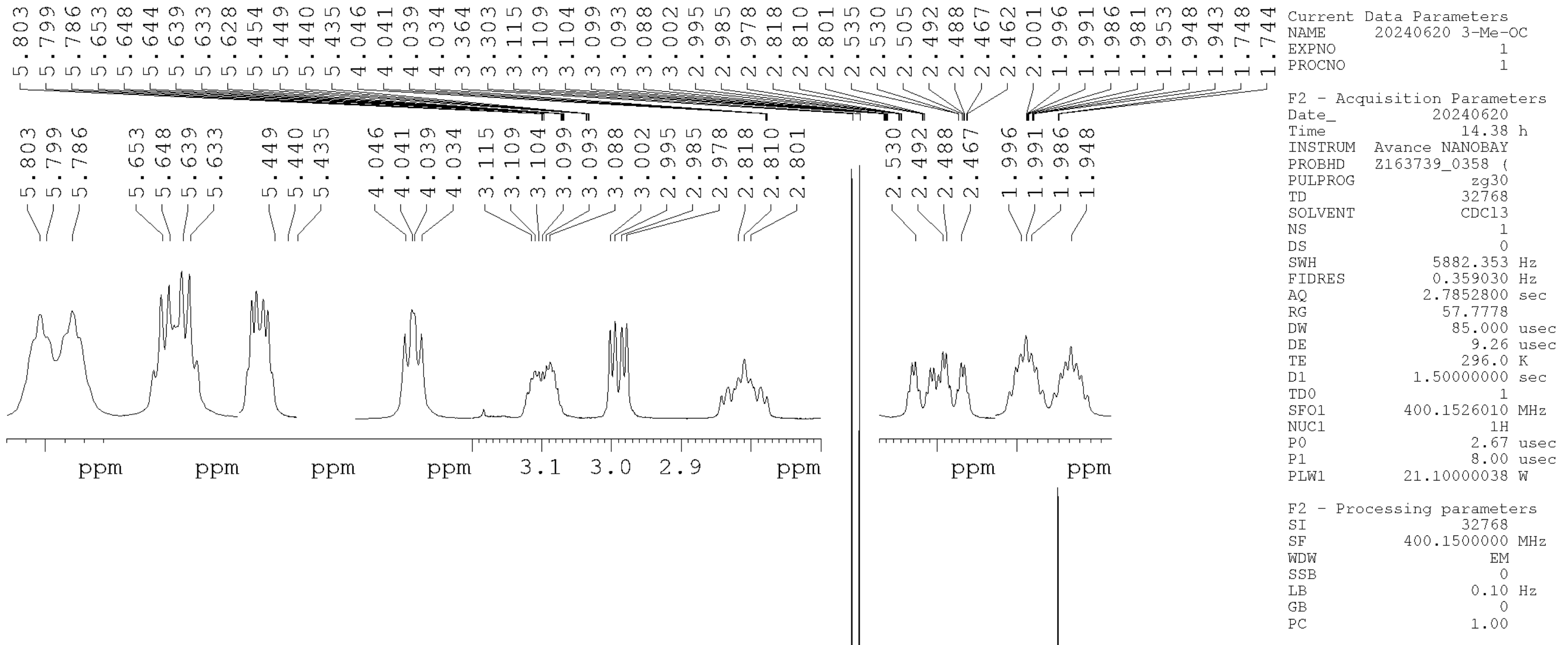

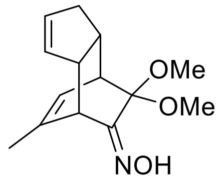

6o

^{1}H NMR of 6p

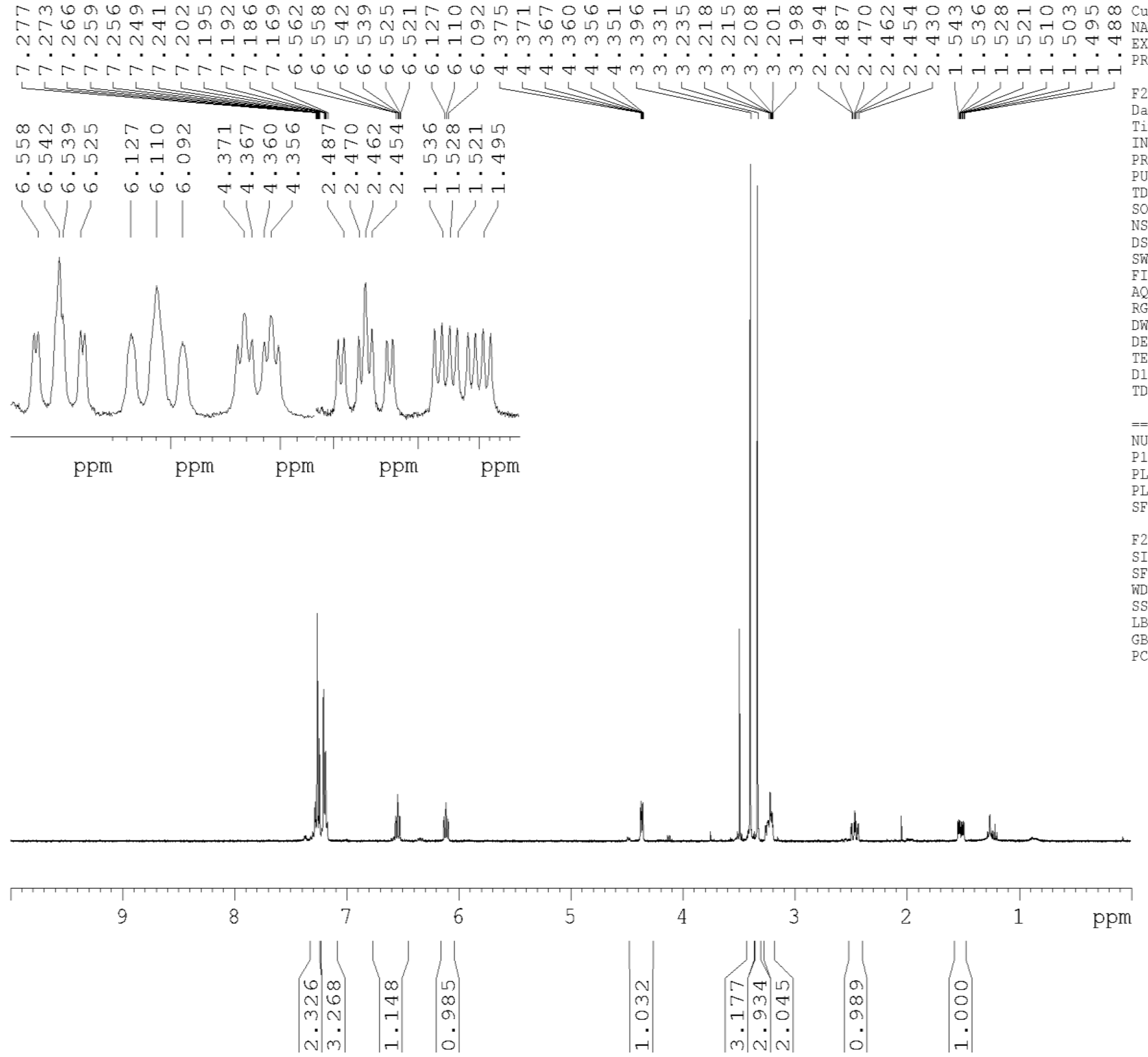

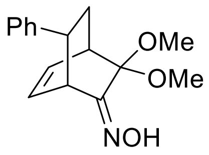

6p

^{1}H NMR of 6q

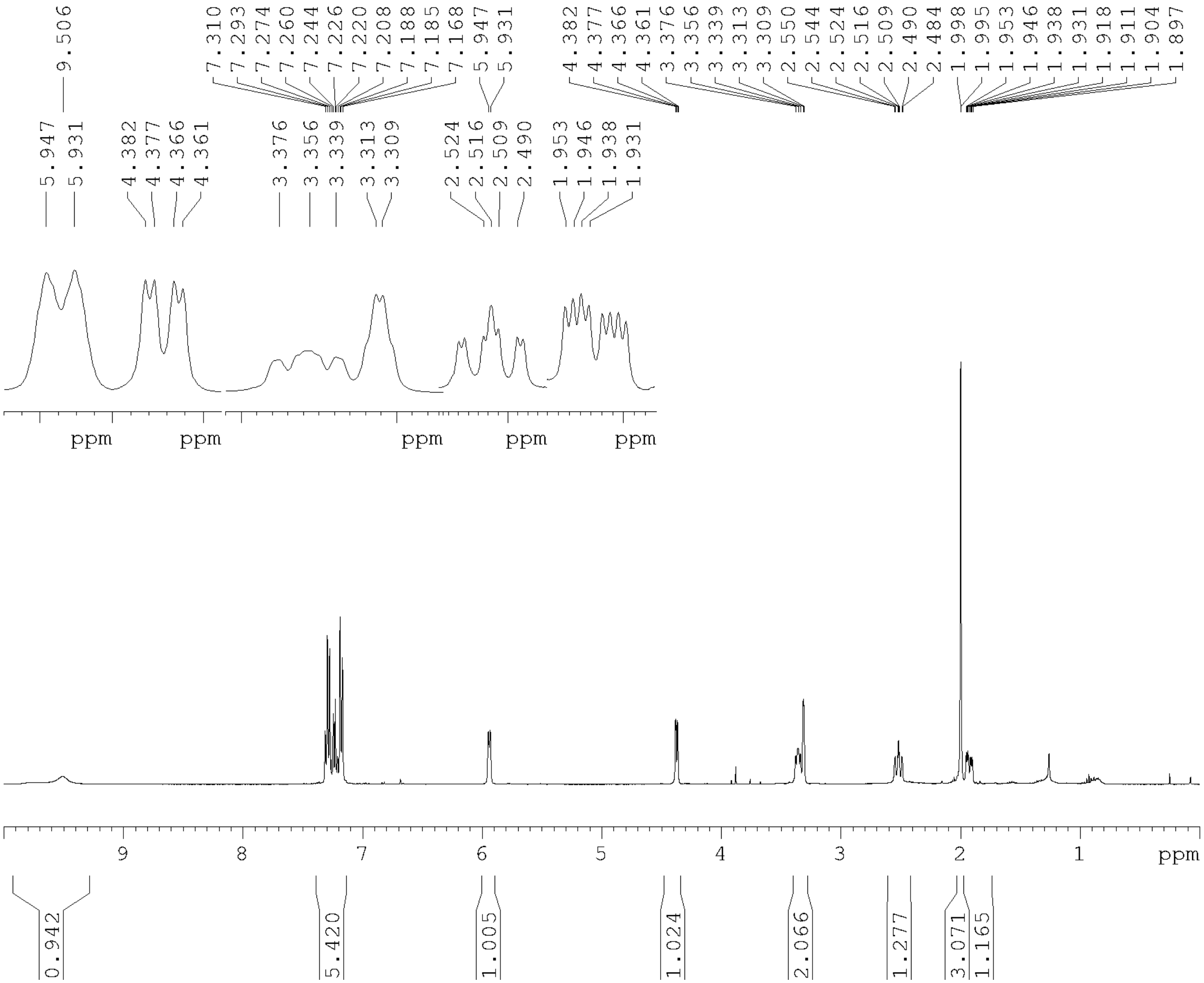

Current Data Parameters
NAME 20240714 4-Me-OS
EXPNO 1
PROCNO 1

F2 - Acquisition Parameters
Date_ 20240714
Time 19.13 h
INSTRUM Avance NANOBAY
PROBHD Z163739_0358 (
PULPROG zg30
TD 32768
SOLVENT CDCl3
NS 16
DS 0
SWH 5882.353 Hz
FIDRES 0.359030 Hz
AQ 2.7852800 sec
RG 101
DW 85.000 usec
DE 9.26 usec
TE 295.0 K
D1 1.50000000 sec
TD0 1
SFO1 400.1526010 MHz
NUC1 1H
P0 2.67 usec
P1 8.00 usec
PLW1 21.10000038 W

F2 - Processing parameters
SI 32768
SF 400.1500098 MHz
WDW EM
SSB 0
LB 0.10 Hz
GB 0
PC 1.00

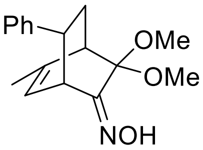

6q

^{1}H NMR of 6r

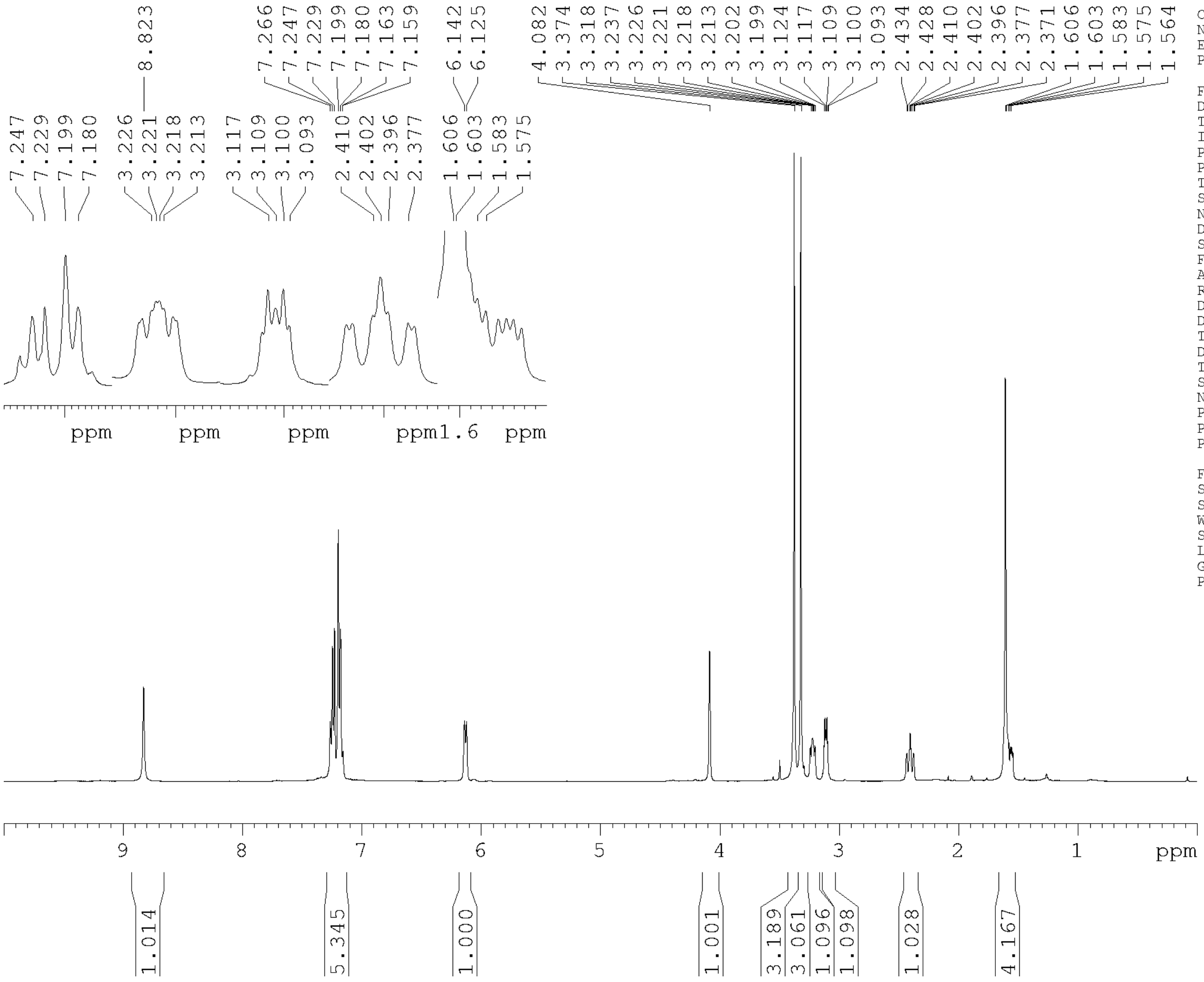

Current Data Parameters
NAME 20240620 3-Me-OS
EXPNO 1
PROCNO 1

F2 - Acquisition Parameters
Date_ 20240620
Time 15.02 h
INSTRUM Avance NANOBAY
PROBHD Z163739_0358 (
PULPROG zg30
TD 32768
SOLVENT CDCl3
NS 16
DS 0
SWH 5882.353 Hz
FIDRES 0.359030 Hz
AQ 2.7852800 sec
RG 32
DW 85.000 usec
DE 9.26 usec
TE 296.3 K
D1 1.50000000 sec
TD0 1
SFO1 400.1526010 MHz
NUC1 1H
P0 2.67 usec
P1 8.00 usec
PLW1 21.10000038 W

F2 - Processing parameters
SI 32768
SF 400.1500151 MHz
WDW EM
SSB 0
LB 0.10 Hz
GB 0
PC 1.00

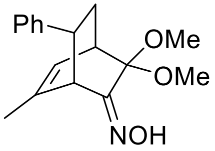

6r

^{1}H NMR of 7a

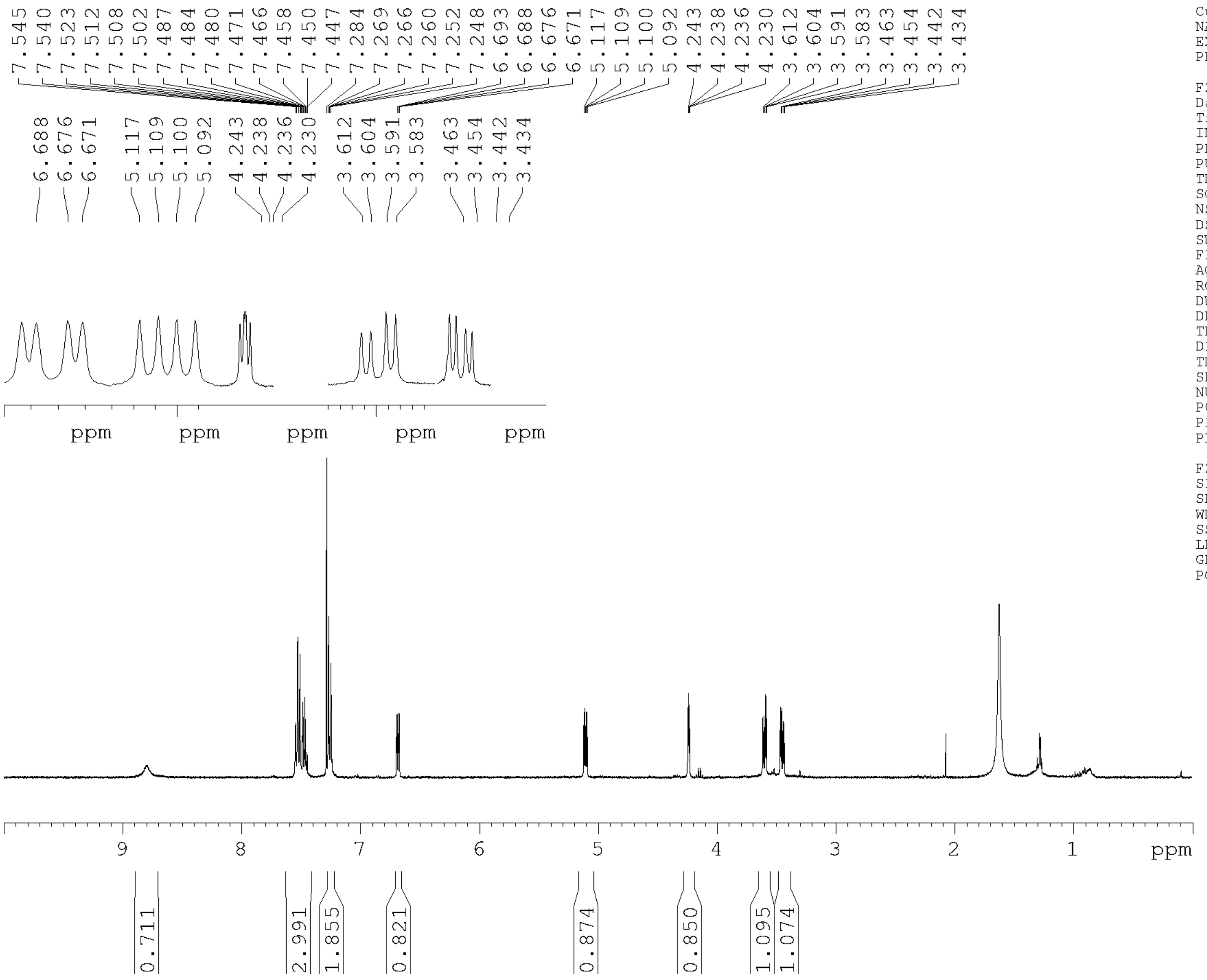

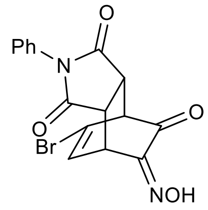

7a

^{13}C{1H} and DEPT 90, 135 NMR of 7a

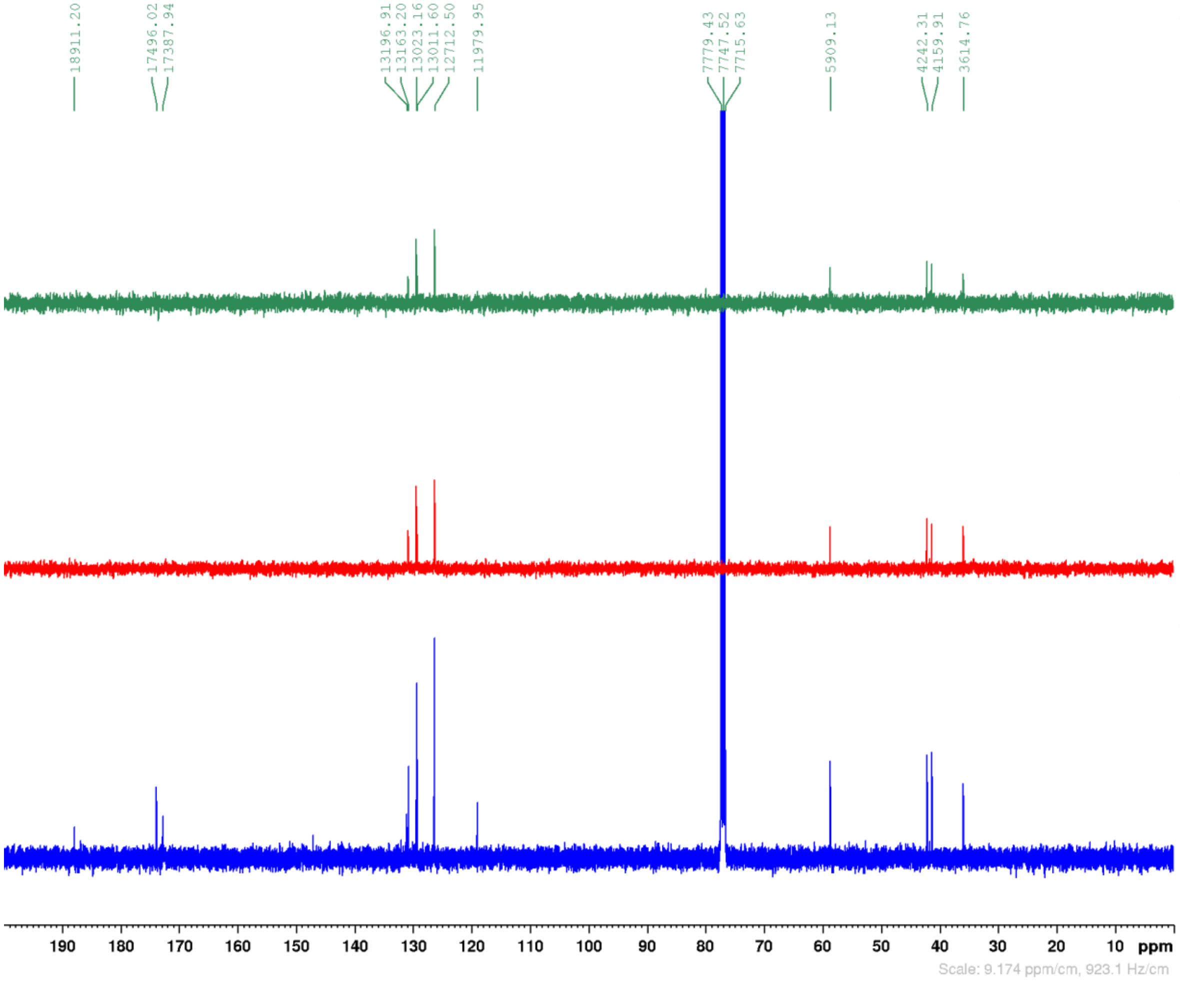

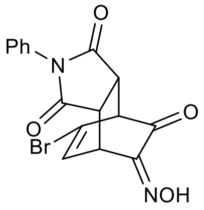

7a

^{1}H NMR of 7c

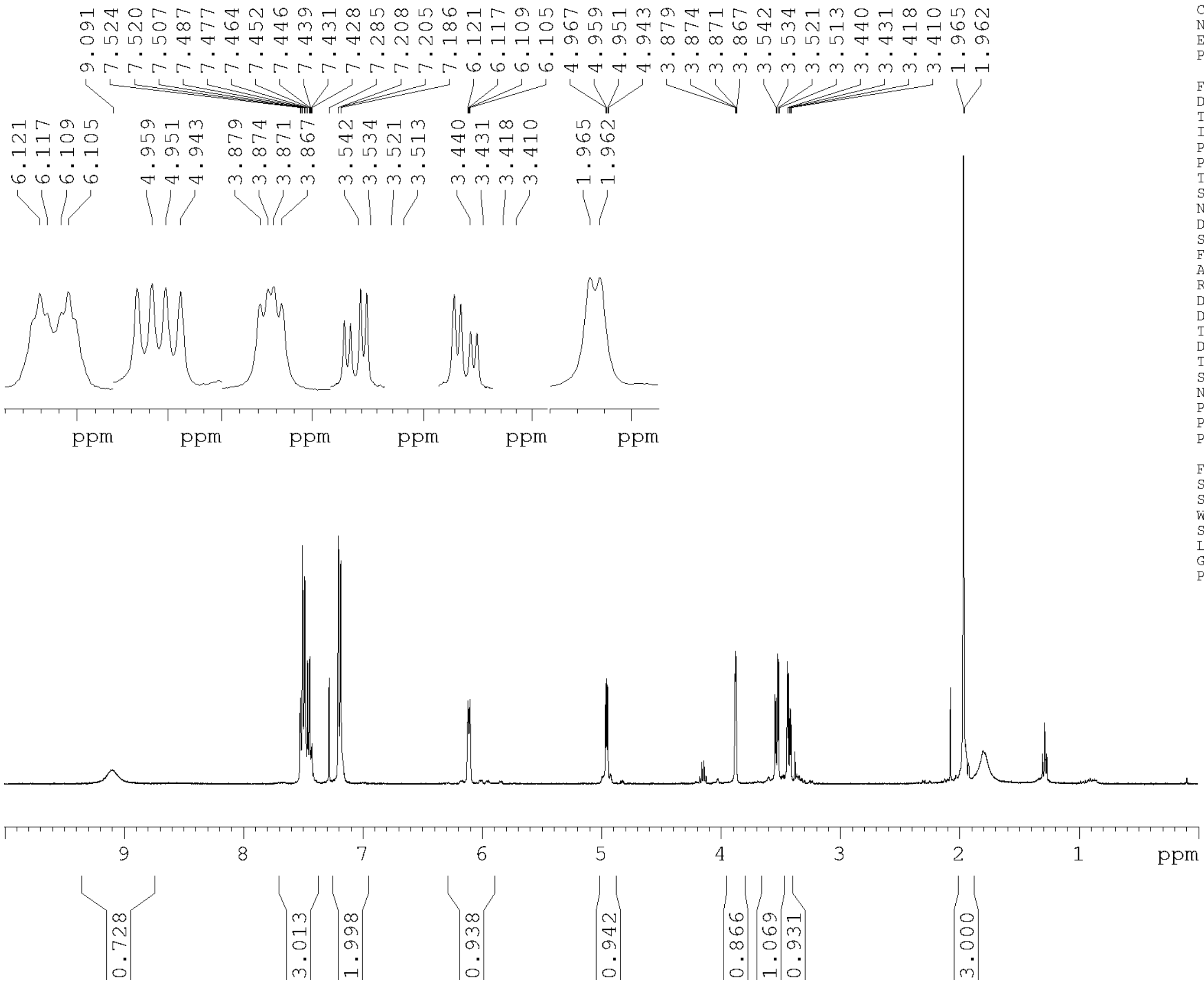

Current Data Parameters
NAME 20240710 4-Me-T
EXPNO 1
PROCNO 1

F2 - Acquisition Parameters
Date_ 20240711
Time 2.10 h
INSTRUM Avance NANOBAY
PROBHD Z163739_0358 (
PULPROG zg30
TD 32768
SOLVENT CDCl3
NS 16
DS 0
SWH 5882.353 Hz
FIDRES 0.359030 Hz
AQ 2.7852800 sec
RG 101
DW 85.000 usec
DE 9.26 usec
TE 294.2 K
D1 1.50000000 sec
TD0 1
SFO1 400.1526010 MHz
NUC1 1H
P0 2.67 usec
P1 8.00 usec
PLW1 21.10000038 W

F2 - Processing parameters
SI 32768
SF 400.1500000 MHz
WDW EM
SSB 0
LB 0.10 Hz
GB 0
PC 1.00

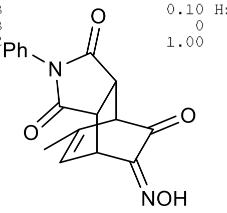

7c

^{13}C{1H} and DEPT 90, 135 NMR of 7c

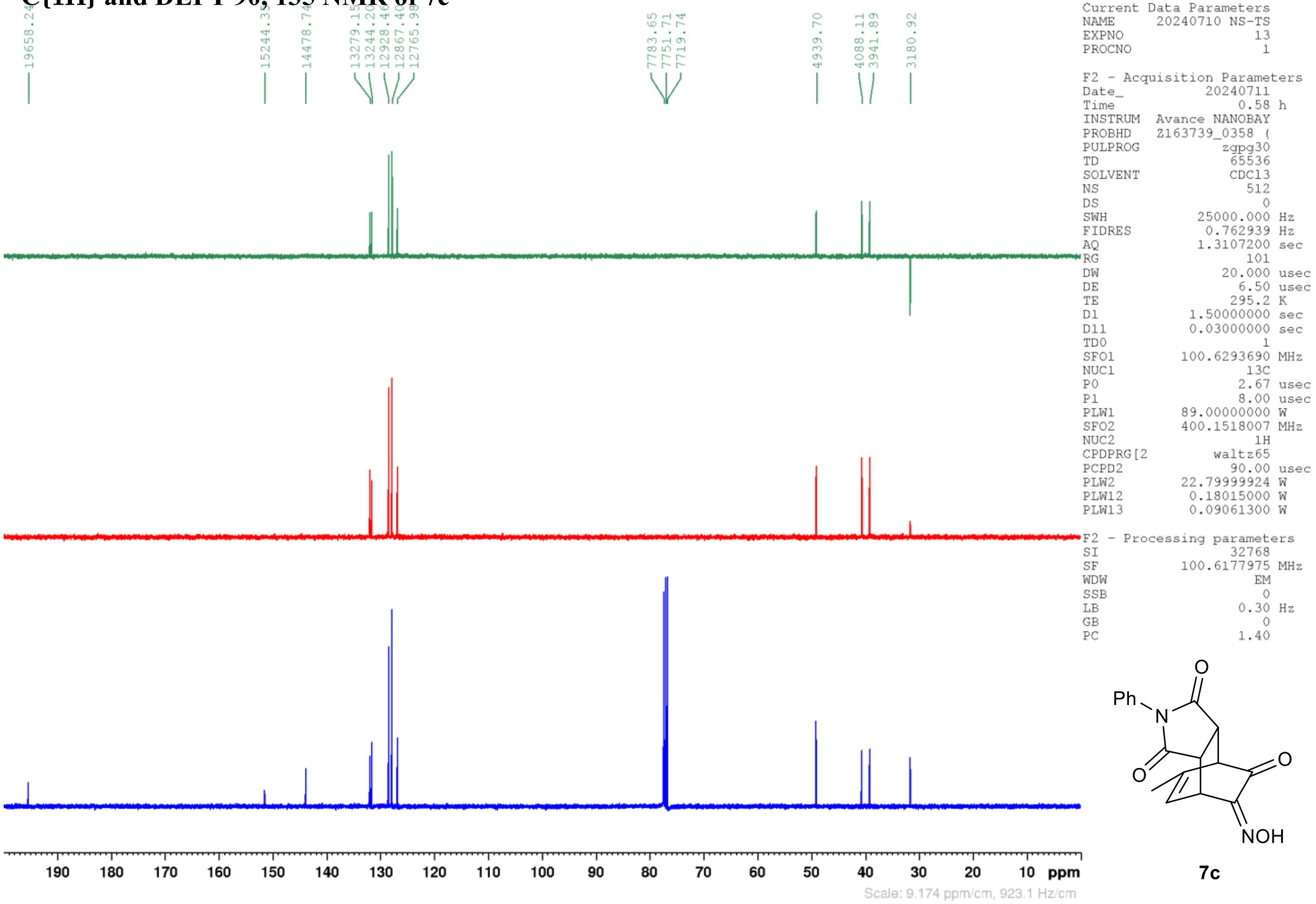

^{1}H NMR of 7d

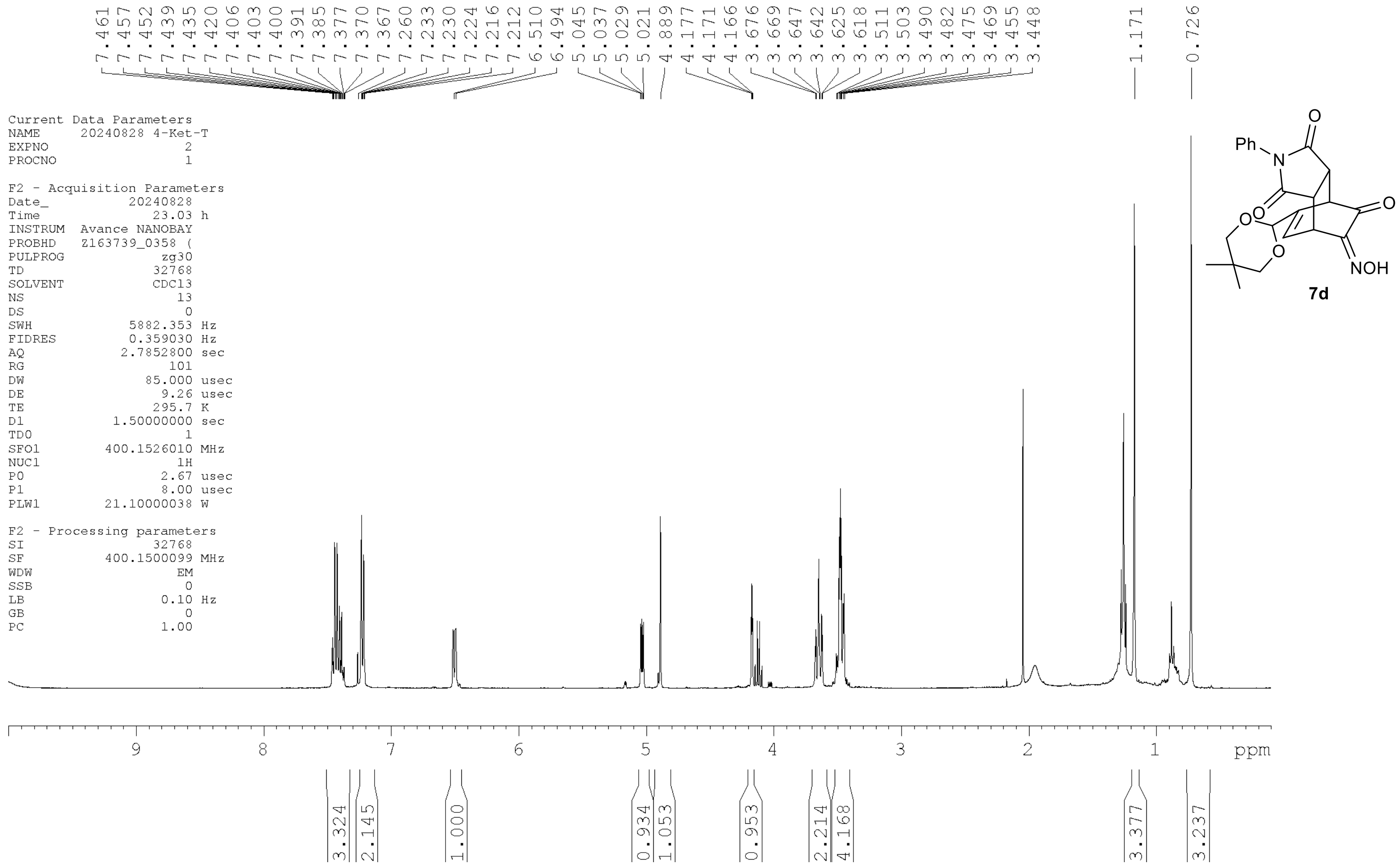

7.461 7.457 7.452 7.439 7.435 7.420 7.406 7.403 7.400 7.391 7.385 7.377 7.370 7.367 7.260 7.233 7.230 7.224 7.216 7.212 6.510 6.494 5.045 5.037 5.029 5.021 4.889 4.177 4.171 4.166 3.676 3.669 3.647 3.642 3.625 3.618 3.511 3.503 3.490 3.482 3.475 3.469 3.455 3.448
1.171
0.726
Current Data Parameters
NAME 20240828 4-Ket-T
EXPNO 2
PROCNO 1
F2 - Acquisition Parameters
Date_ 20240828
Time 23.03 h
INSTRUM Avance NANOBAY
PROBHD Z163739_0358 (
PULPROG zg30
TD 32768
SOLVENT CDCl3
NS 13
DS 0
SWH 5882.353 Hz
FIDRES 0.359030 Hz
AQ 2.7852800 sec
RG 101
DW 85.000 usec
DE 9.26 usec
TE 295.7 K
D1 1.50000000 sec
TD0 1
SFO1 400.1526010 MHz
NUC1 1H
P0 2.67 usec
P1 8.00 usec
PLW1 21.10000038 W
F2 - Processing parameters
SI 32768
SF 400.1500099 MHz
WDW EM
SSB 0
LB 0.10 Hz
GB 0
PC 1.00
Ph
N
O
O
O
O
O
NOH
7d
9
8
7
6
5
4
3
2
1
ppm
3.324
2.145
1.000
0.934
1.053
0.953
2.214
4.168
3.377
3.237

^{13}C{1H} and DEPT 90, 135 NMR of 7d

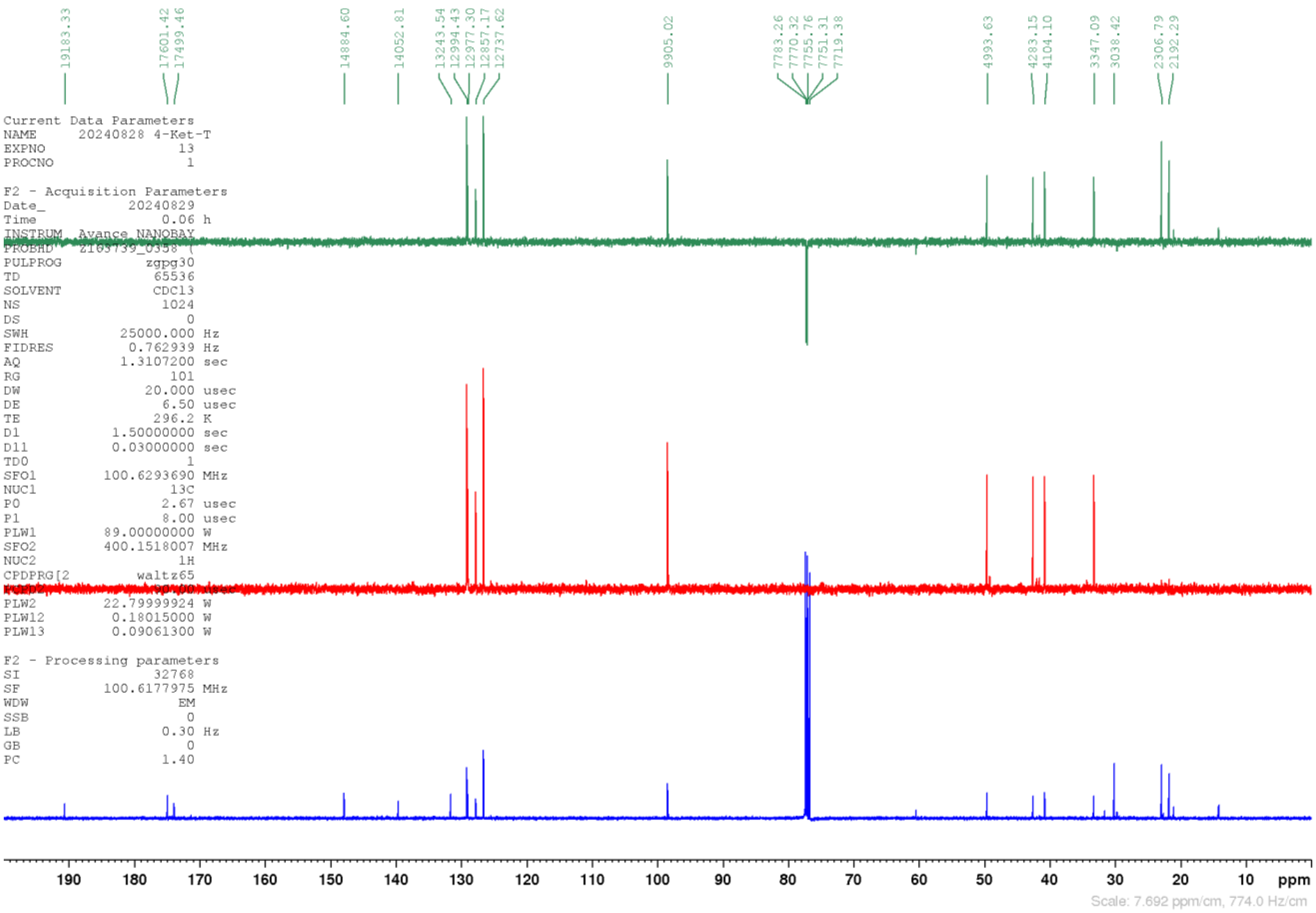

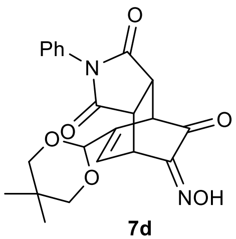

7d

^{1}H NMR of 7e

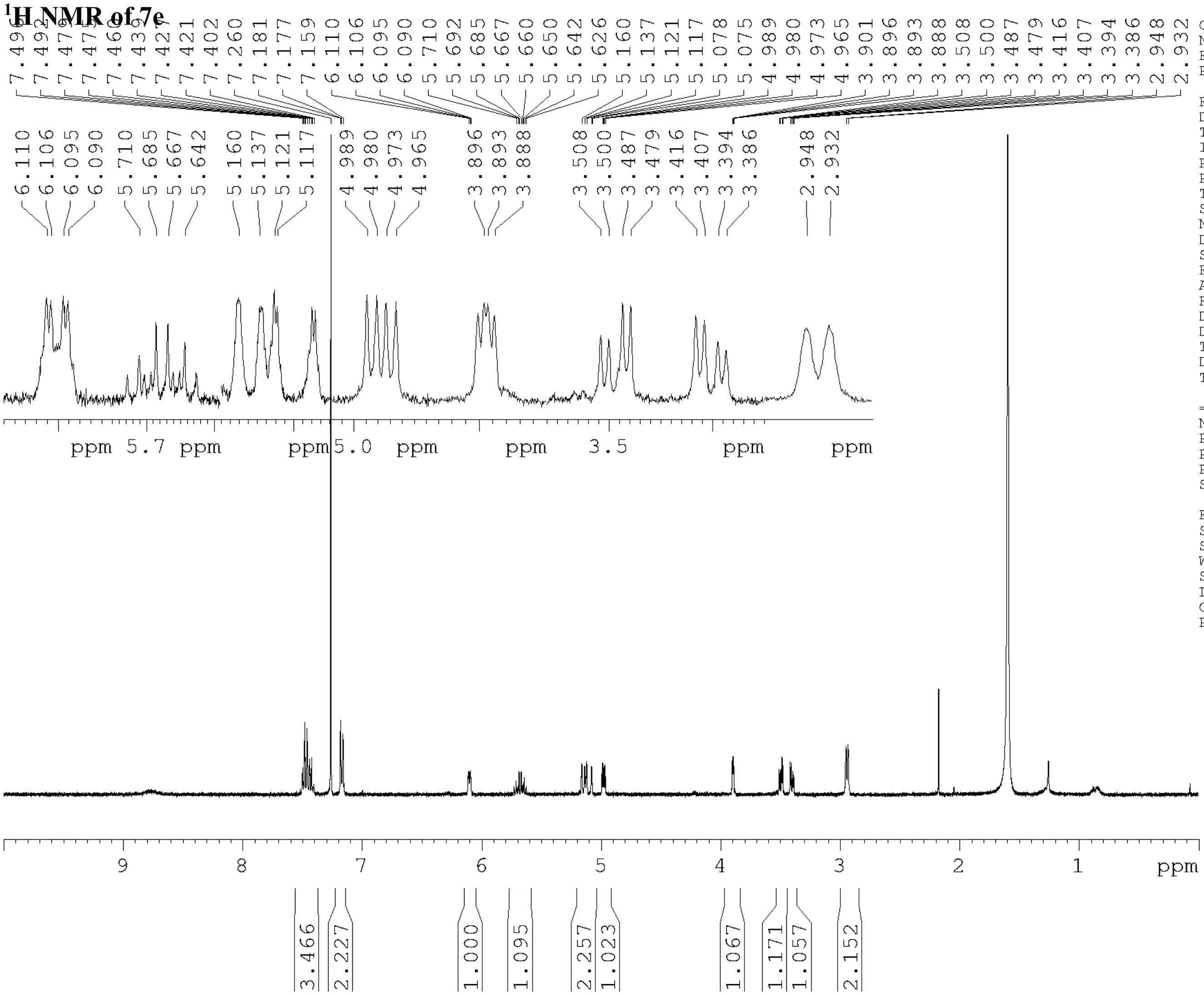

```
Current Data Parameters
NAME       20220802 SZ034 E-H
EXPNO                       1
PROCNO                      1

F2 - Acquisition Parameters
Date_              20220802
Time                  19.11
INSTRUM               spect
PROBHD    5 mm BBO BB-1H
PULPROG                zg30
TD                    32768
SOLVENT               CDCl3
NS                       16
DS                        0
SWH                6009.615 Hz
FIDRES             0.183399 Hz
AQ                2.7262976 sec
RG                      322
DW                   83.200 usec
DE                     6.50 usec
TE                    296.6 K
D1               1.50000000 sec
TD0                       1

======== CHANNEL f1 ========
NUC1                     1H
P1                    14.00 usec
PL1                   -1.00 dB
PL1W             7.55784369 W
SFO1            400.1326010 MHz

F2 - Processing parameters
SI                    32768
SF              400.1300087 MHz
WDW                      EM
SSB                       0
LB                        0 Hz
GB                        0
PC                     1.00
```

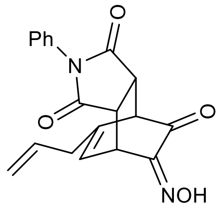

7e

$^{13}C\{^1H\}$ and DEPT 90, 135 NMR of 7e

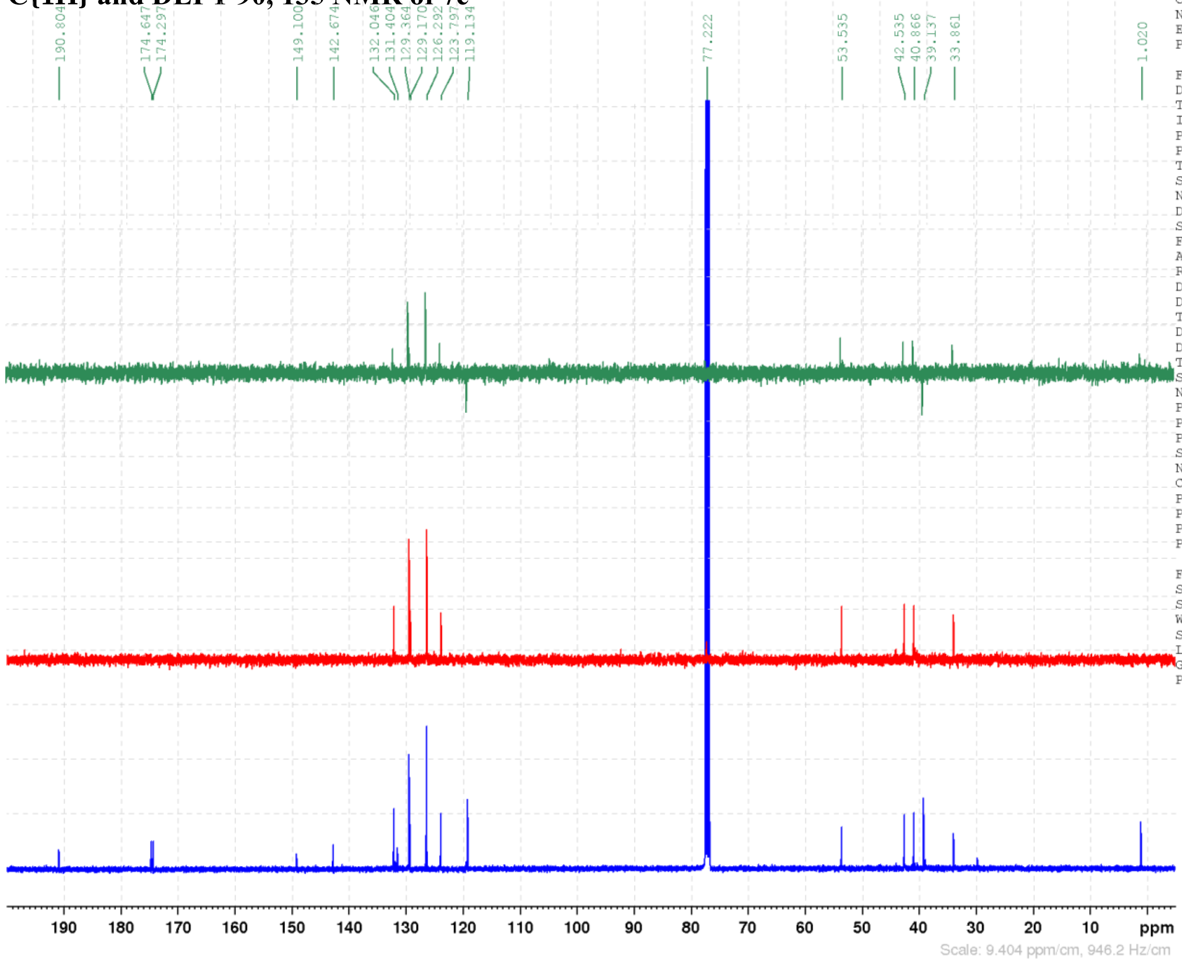

Current Data Parameters
NAME SZ034-E+H
EXPNO 15
PROCNO 1

F2 - Acquisition Parameters
Date_ 20220805
Time 8.17 h
INSTRUM Avance NANOBAY
PROBHD Z163739_0358 (
PULPROG zgpg30
TD 65536
SOLVENT CDCl3
NS 13914
DS 0
SWH 25000.000 Hz
FIDRES 0.762939 Hz
AQ 1.3107200 sec
RG 101
DW 20.000 usec
DE 6.50 usec
TE 299.5 K
D1 1.50000000 sec
D11 0.03000000 sec
TD0 1
SFO1 100.6293690 MHz
NUC1 13C
P0 2.67 usec
P1 8.00 usec
PLW1 89.00000000 W
SFO2 400.1518007 MHz
NUC2 1H
CPDPRG[2 waltz65
PCPD2 90.00 usec
PLW2 22.79999924 W
PLW12 0.18015000 W
PLW13 0.09061300 W

F2 - Processing parameters
SI 32768
SF 100.6177975 MHz
WDW EM
SSB 0
LB 0.30 Hz
GB 0
PC 1.40

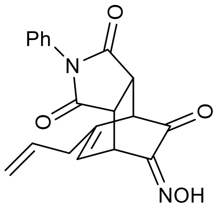

7e

^{1}H NMR of 7f

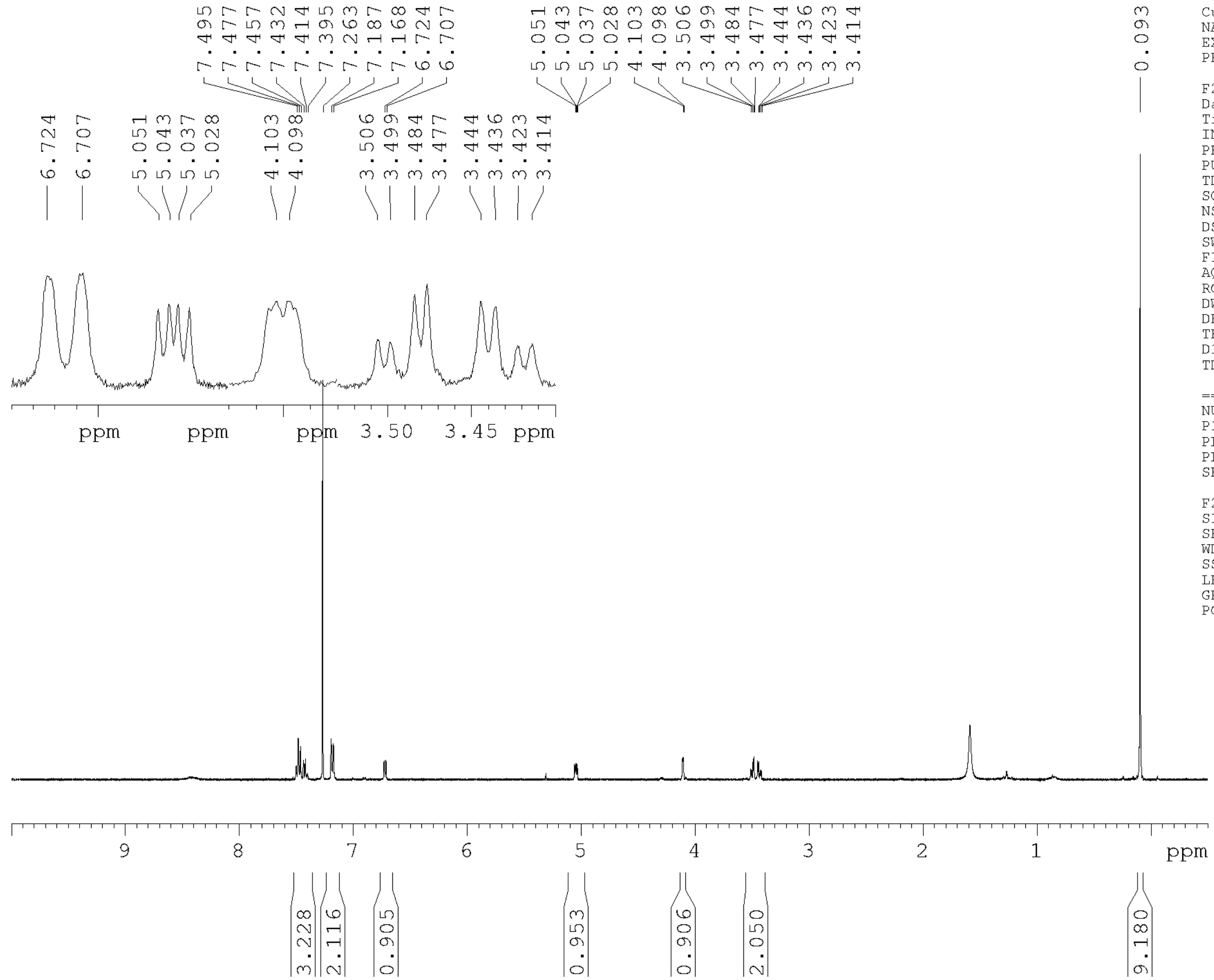

```
Current Data Parameters
NAME     20230714 SZ138-C
EXPNO                   1
PROCNO                  1

F2 - Acquisition Parameters
Date_          20230714
Time              18.04
INSTRUM           spect
PROBHD   5 mm BBO BB-1H
PULPROG            zg30
TD                32768
SOLVENT           CDCl3
NS                   16
DS                    0
SWH            6009.615 Hz
FIDRES         0.183399 Hz
AQ            2.7262976 sec
RG                  322
DW               83.200 usec
DE                 6.50 usec
TE                296.7 K
D1           1.50000000 sec
TD0                   1

======== CHANNEL f1 ========
NUC1                 1H
P1                14.00 usec
PL1               -1.00 dB
PL1W         7.55784369 W
SFO1        400.1326010 MHz

F2 - Processing parameters
SI                32768
SF          400.1300087 MHz
WDW                  EM
SSB                   0
LB                    0 Hz
GB                    0
PC                 1.00
```

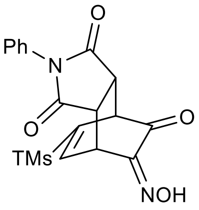

7f

^{13}C{1H} and DEPT 90, 135 NMR of 7f

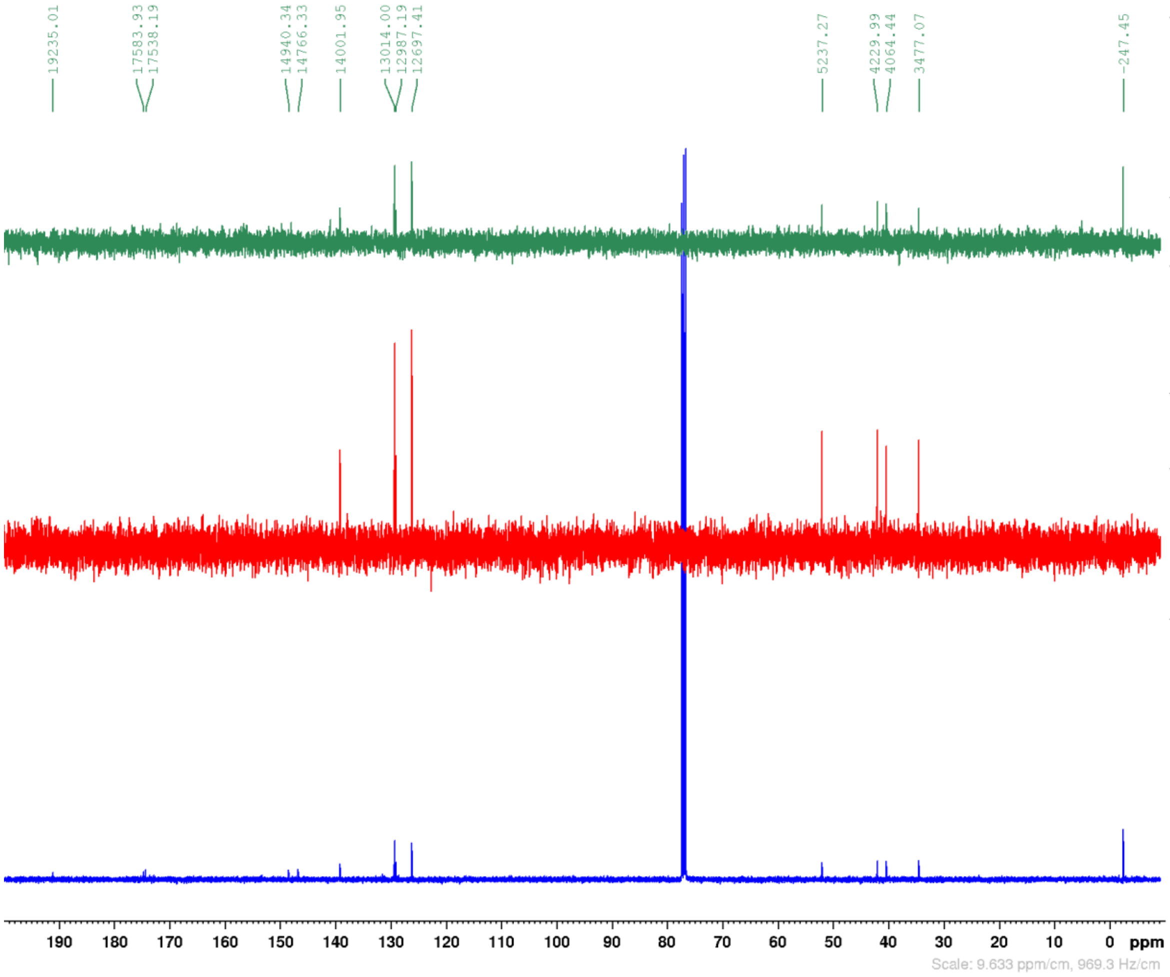

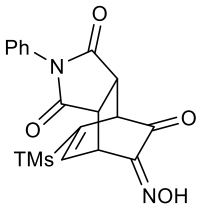

7f

^{1}H NMR of 7g

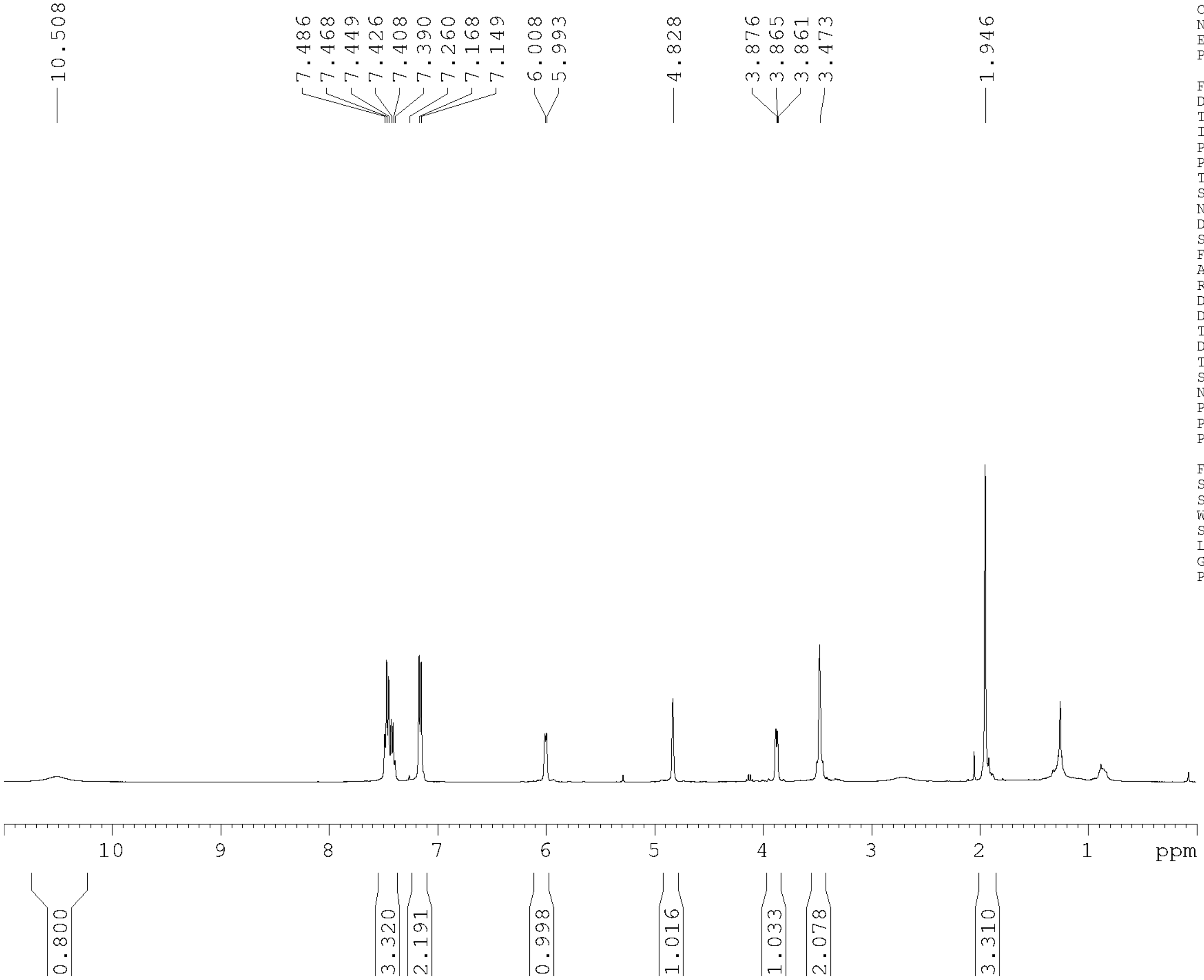


```
Current Data Parameters
NAME     20240719 3-Me-T
EXPNO                  1
PROCNO                 1

F2 - Acquisition Parameters
Date_           20240719
Time               23.24 h
INSTRUM  Avance NANOBAY
PROBHD   Z163739_0358 (
PULPROG             zg30
TD                 32768
SOLVENT            CDCl3
NS                    16
DS                     0
SWH             5882.353 Hz
FIDRES          0.359030 Hz
AQ             2.7852800 sec
RG               89.0059
DW                85.000 usec
DE                  9.26 usec
TE                 296.6 K
D1            1.50000000 sec
TD0                    1
SFO1         400.1526010 MHz
NUC1                  1H
P0                  2.67 usec
P1                  8.00 usec
PLW1         21.10000038 W

F2 - Processing parameters
SI                 32768
SF           400.1500101 MHz
WDW                   EM
SSB                    0
LB                  0.10 Hz
GB                     0
PC                  1.00
```

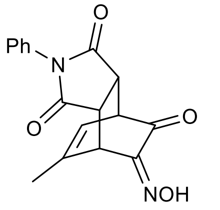

7g

^{13}C{1H} and DEPT 90, 135 NMR of 7g

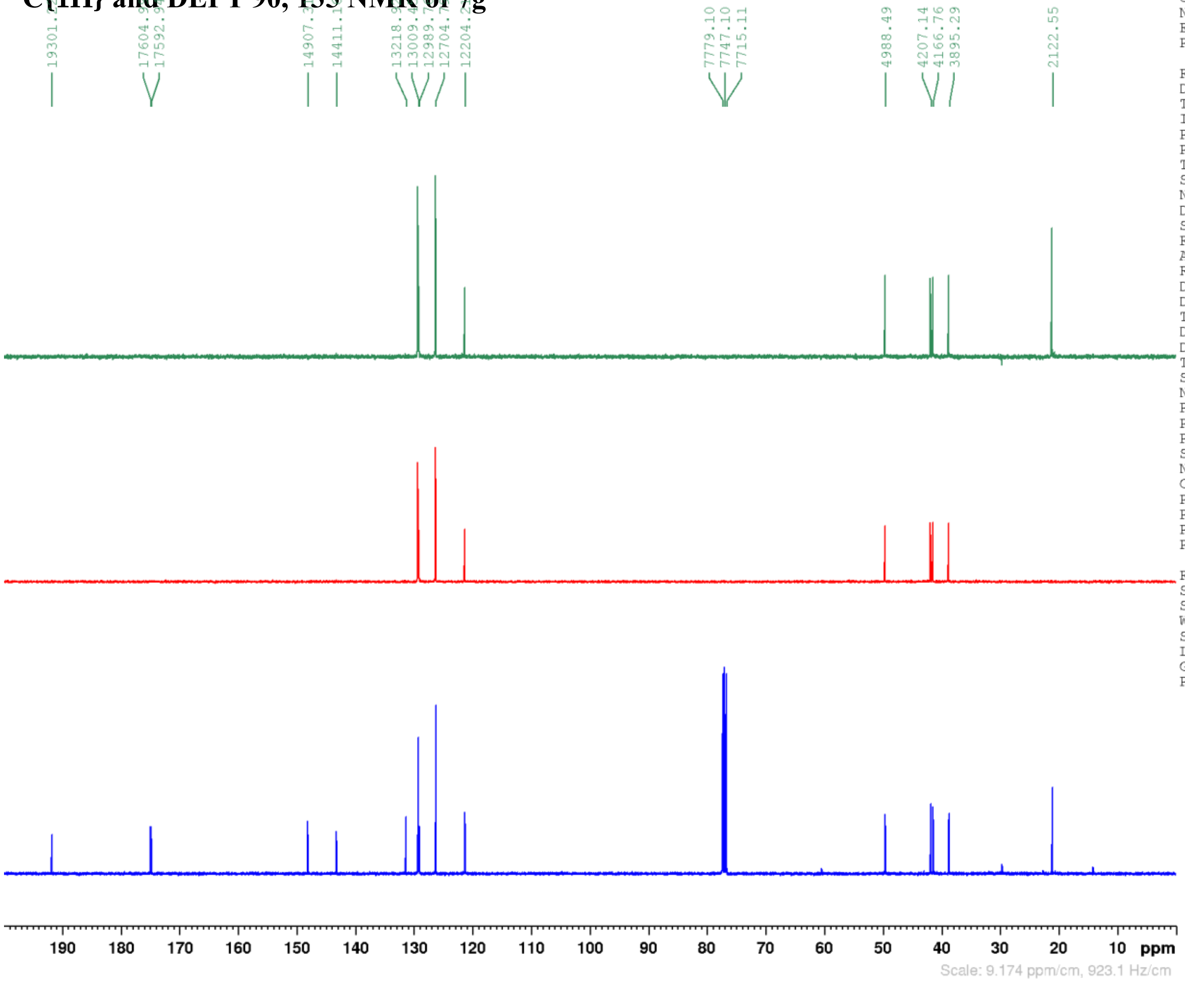

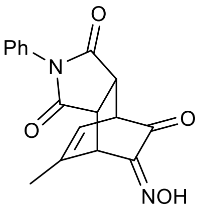

7g

^{1}H NMR of 7h

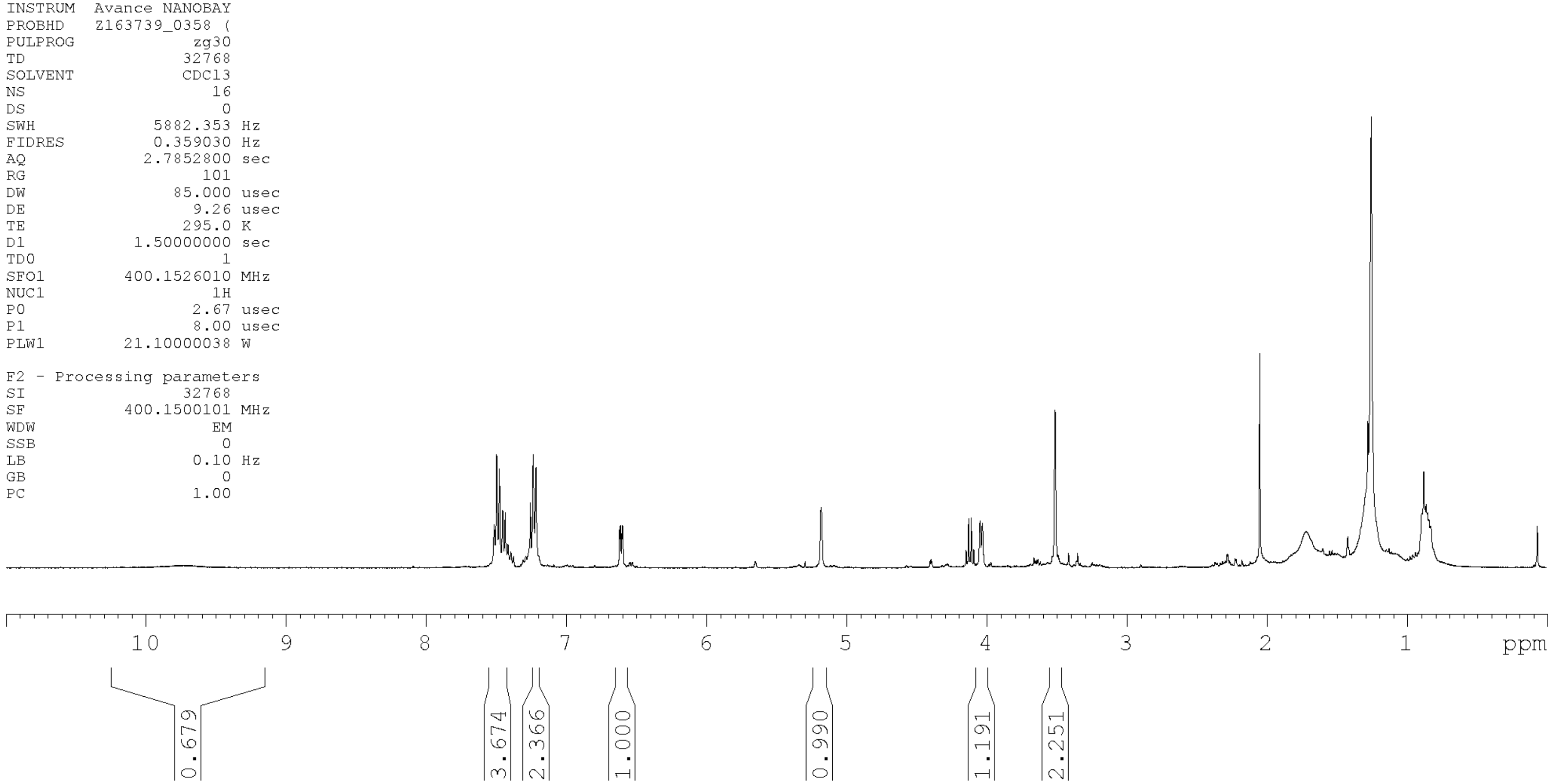

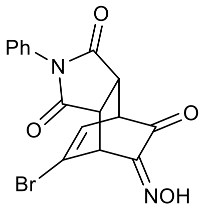

7h

^{13}C{1H} and DEPT 90, 135 NMR of 7h

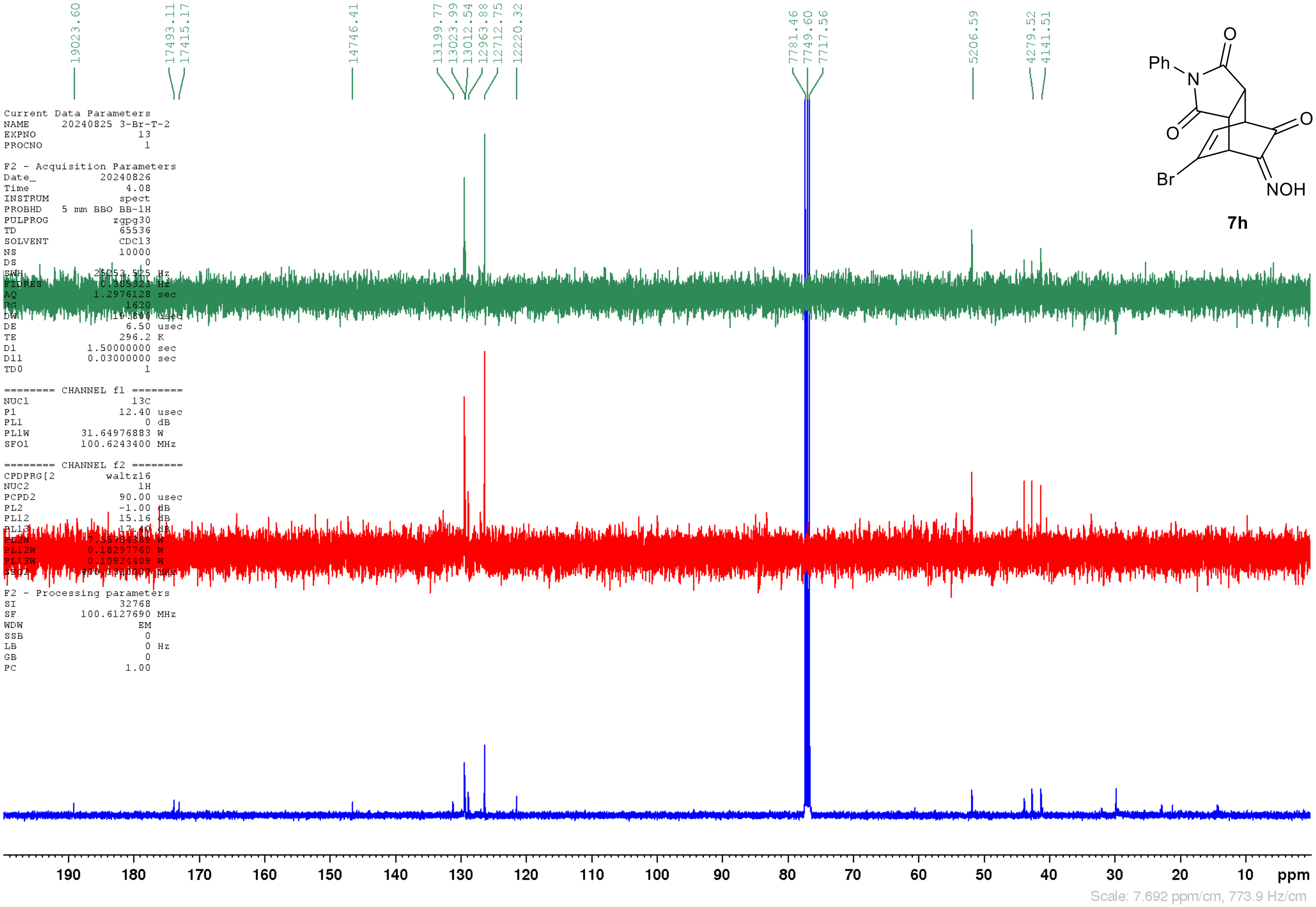

7h

^{1}H NMR of 7i

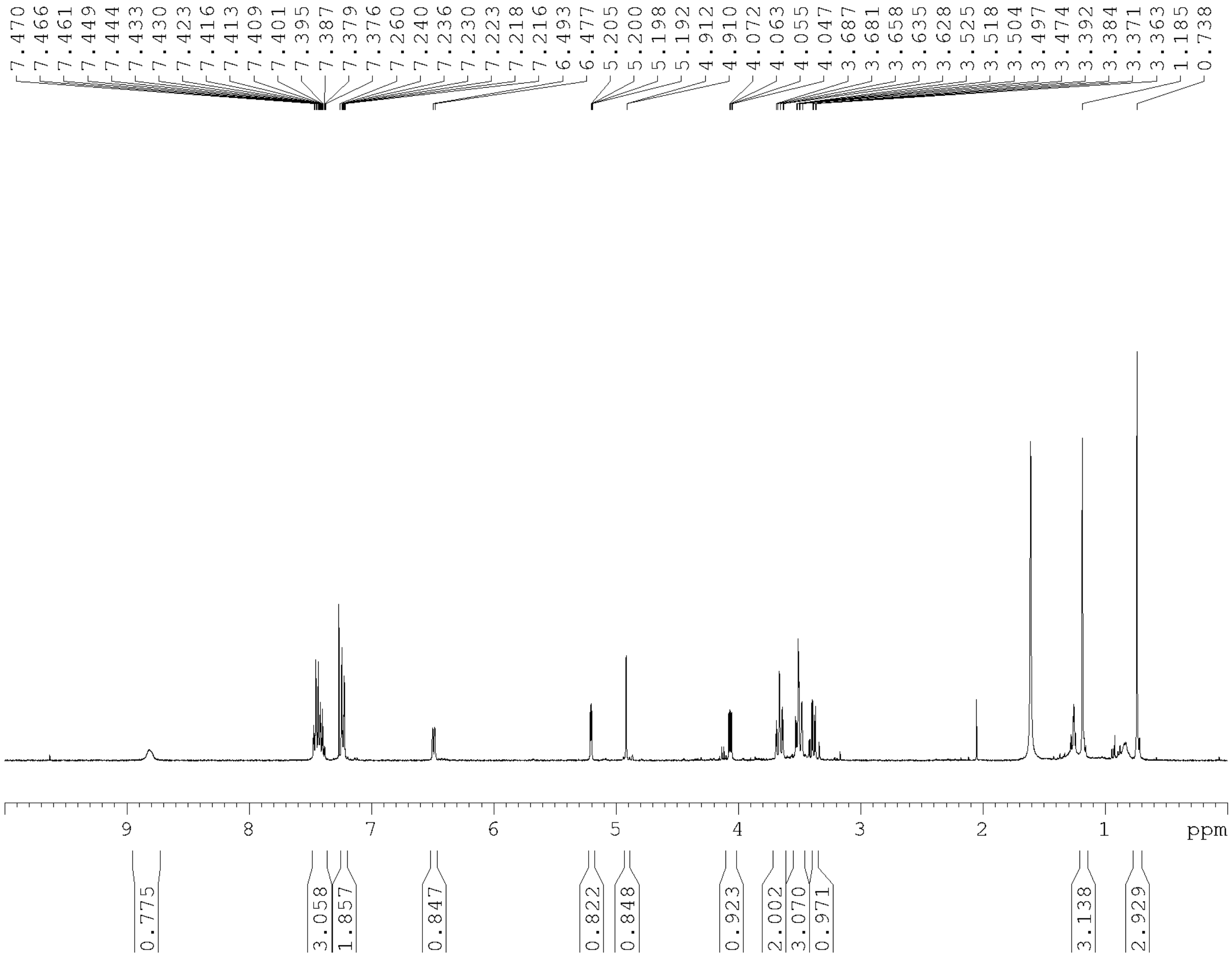


```
Current Data Parameters
NAME     20240806 3-ket-T
EXPNO                  1
PROCNO                 1

F2 - Acquisition Parameters
Date_         20240806
Time             16.24 h
INSTRUM  Avance NANOBAY
PROBHD   Z163739_0358 (
PULPROG           zg30
TD               32768
SOLVENT          CDCl3
NS                  16
DS                   0
SWH           5882.353 Hz
FIDRES        0.359030 Hz
AQ           2.7852800 sec
RG                 101
DW              85.000 usec
DE                9.26 usec
TE               294.0 K
D1          1.50000000 sec
TD0                  1
SFO1       400.1526010 MHz
NUC1                1H
P0                2.67 usec
P1                8.00 usec
PLW1       21.10000038 W

F2 - Processing parameters
SI               32768
SF         400.1500098 MHz
WDW                 EM
SSB                  0
LB                0.10 Hz
GB                   0
PC                1.00
```

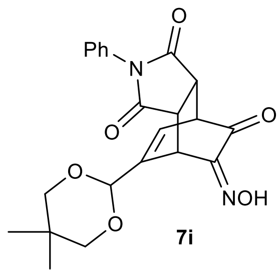

7i

^{13}C{1H} and DEPT 90, 135 NMR of 7i

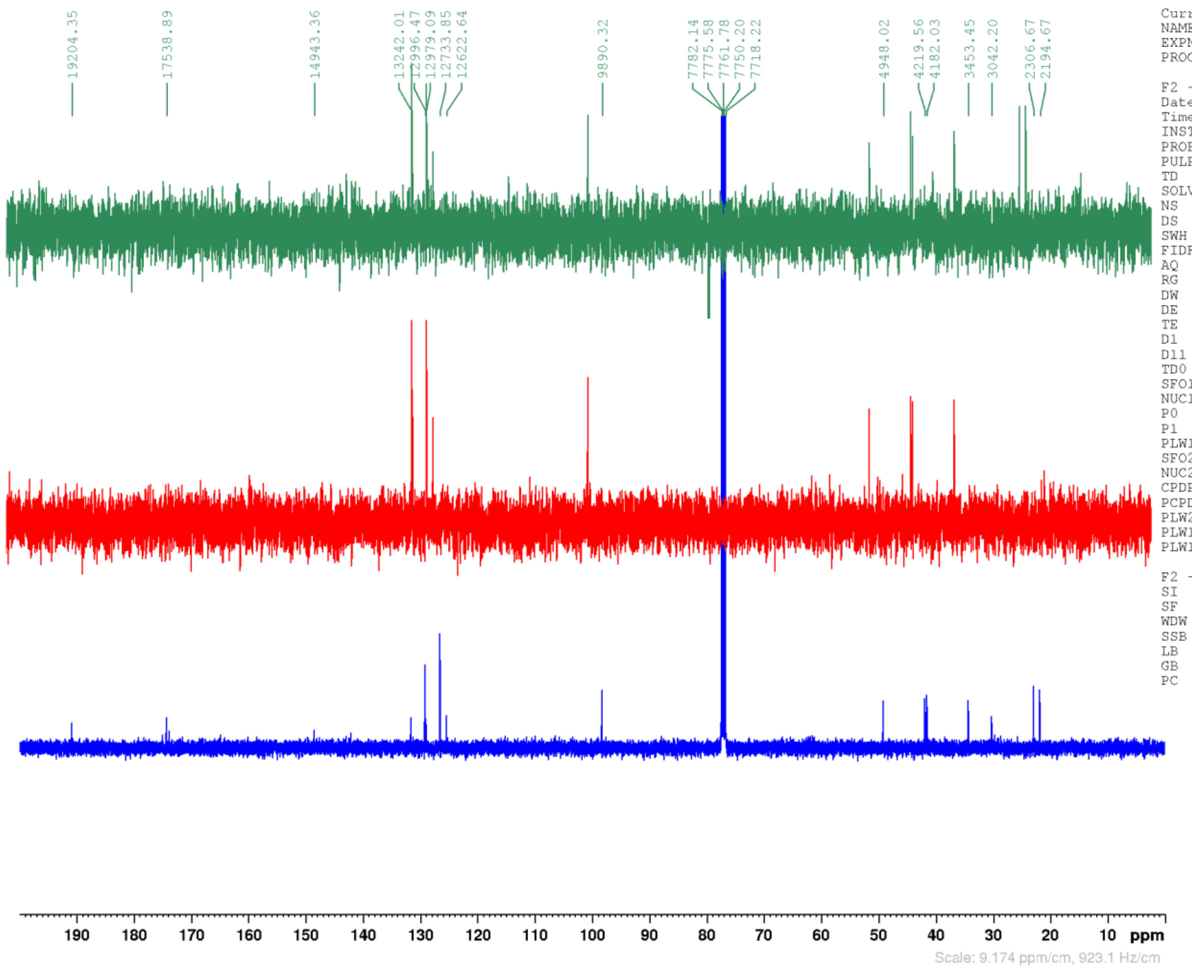

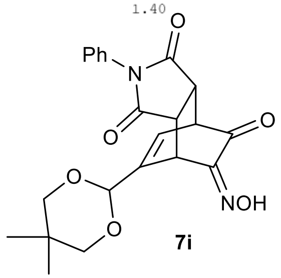

7i

^{1}H NMR of 7j

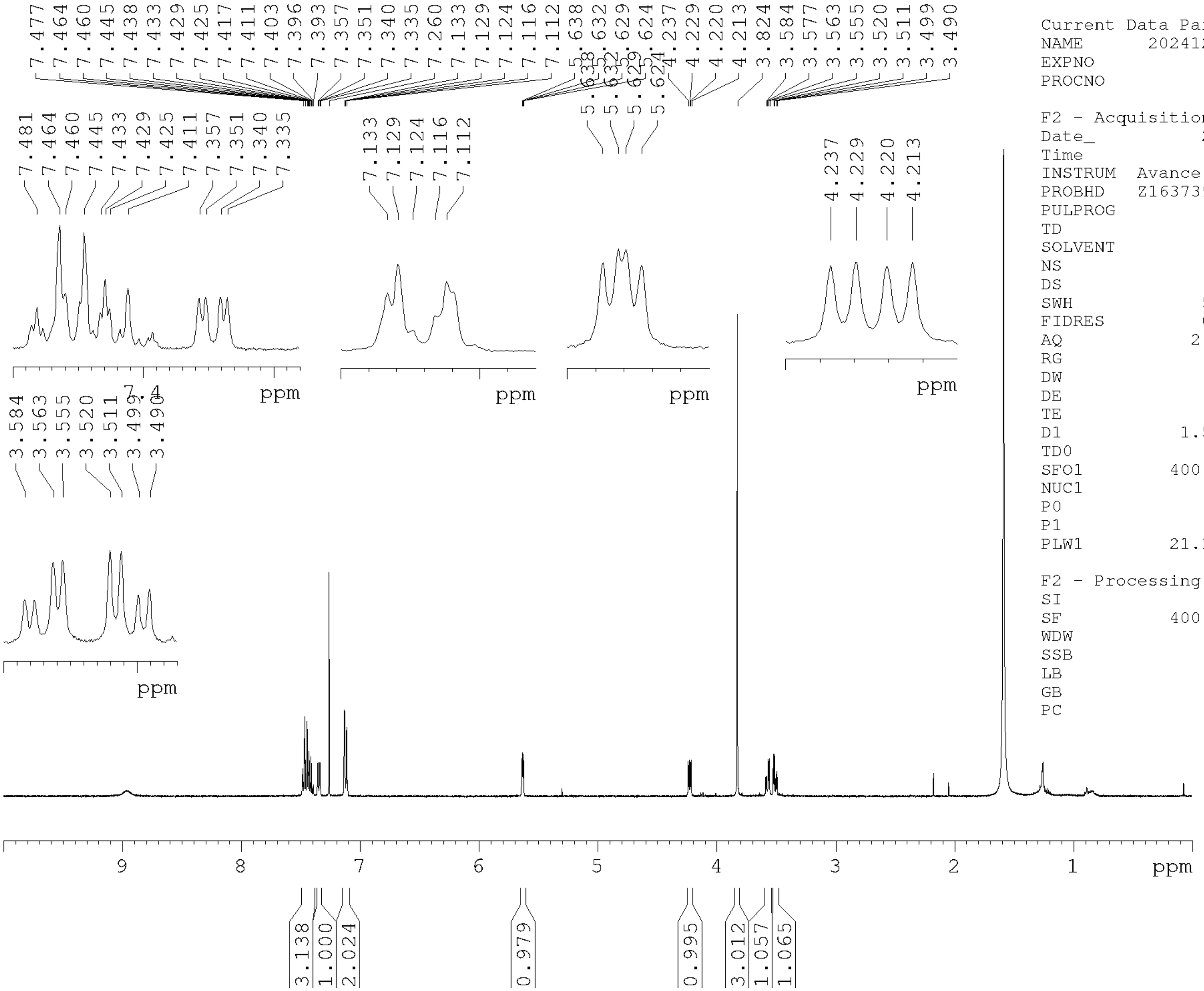

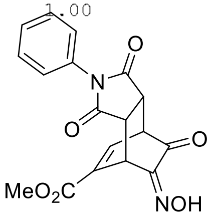

7j

1H NMR of 7k

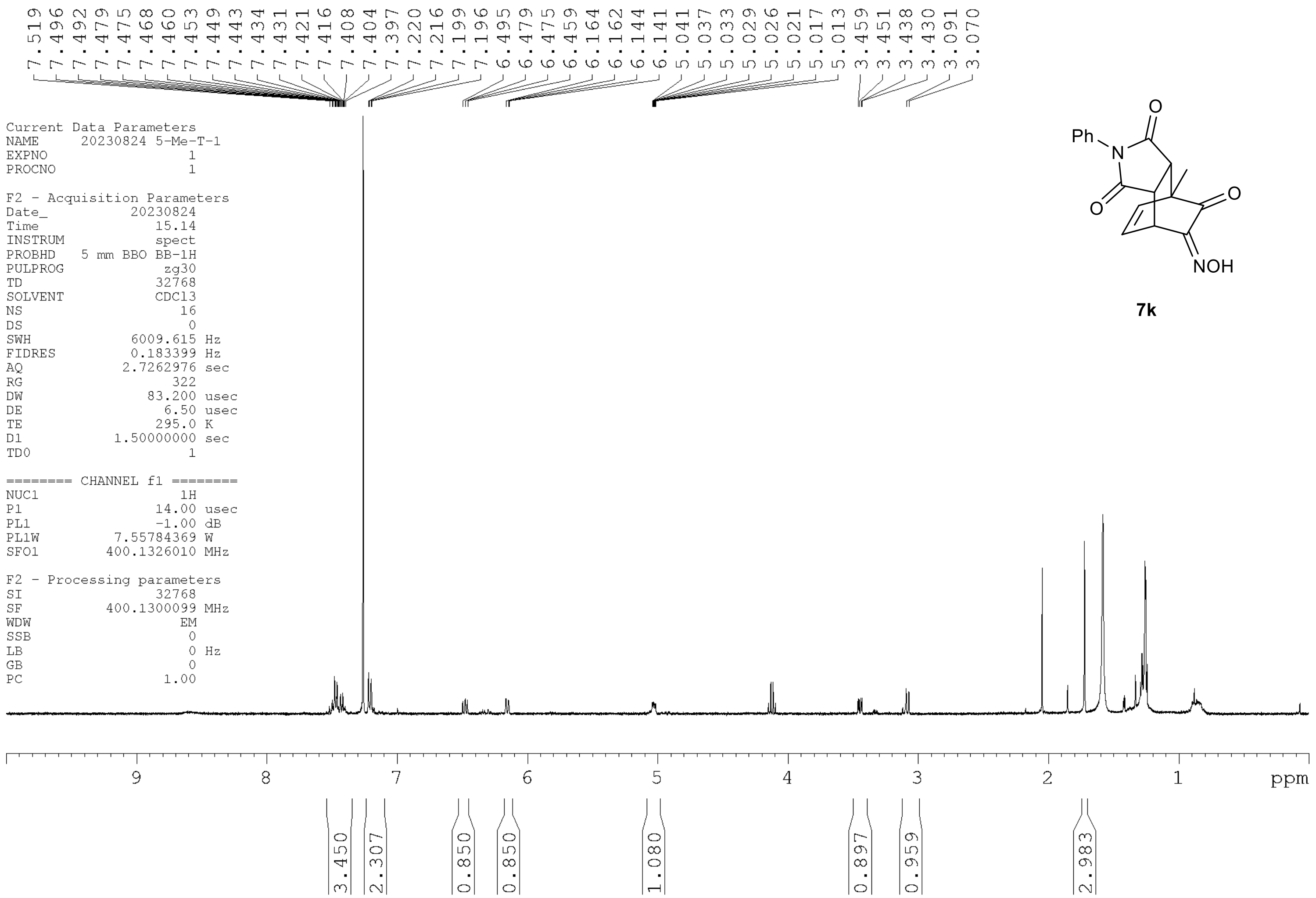

7k

^{13}C{1H} NMR of 7k

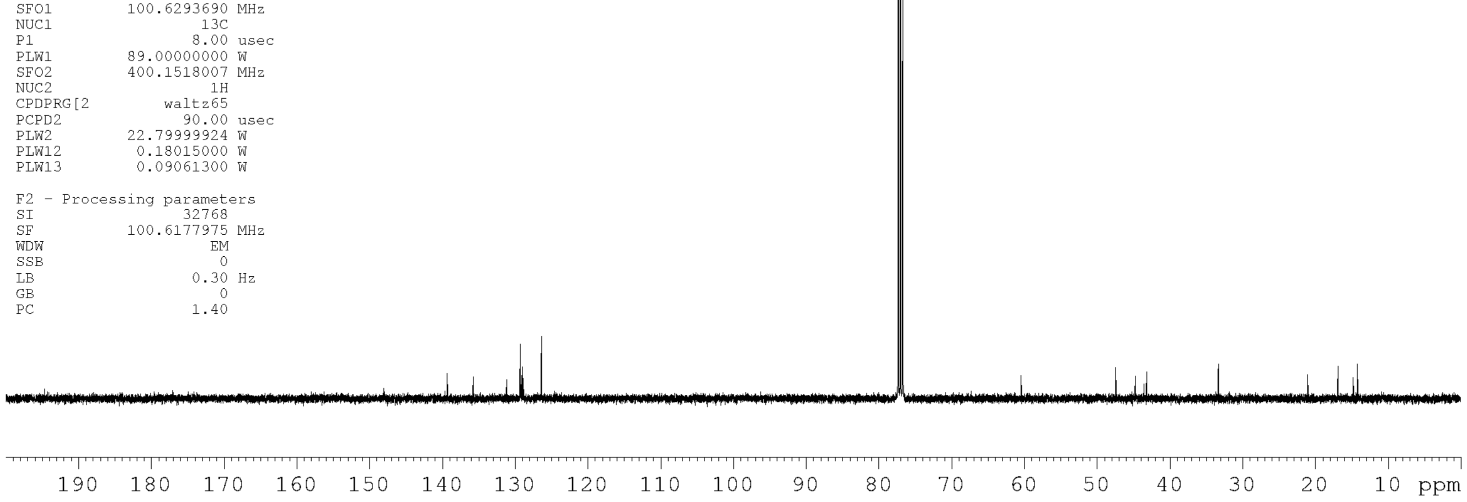

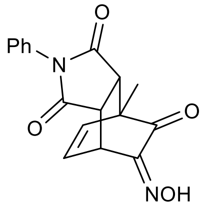

7k

^{1}H NMR of 7l

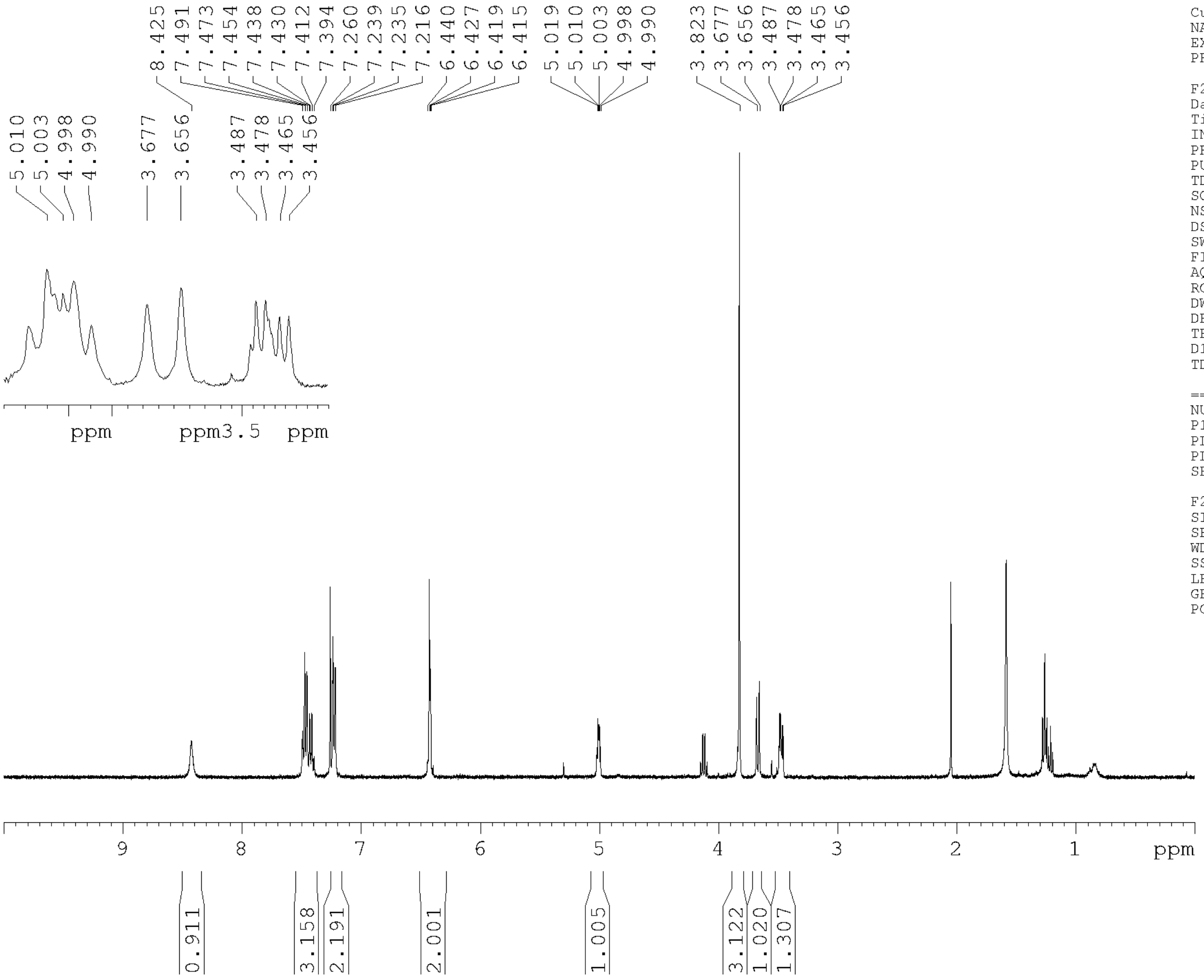


```
Current Data Parameters
NAME     20240523 5-OMe-T
EXPNO                   1
PROCNO                  1

F2 - Acquisition Parameters
Date_            20240523
Time                23.19
INSTRUM             spect
PROBHD   5 mm BBO BB-1H
PULPROG              zg30
TD                  32768
SOLVENT             CDCl3
NS                     64
DS                      0
SWH              6009.615 Hz
FIDRES           0.183399 Hz
AQ              2.7262976 sec
RG                    362
DW                 83.200 usec
DE                   6.50 usec
TE                  295.3 K
D1             1.50000000 sec
TD0                     1

======== CHANNEL f1 ========
NUC1                   1H
P1                  14.00 usec
PL1                 -1.00 dB
PL1W           7.55784369 W
SFO1          400.1326010 MHz

F2 - Processing parameters
SI                  32768
SF            400.1300104 MHz
WDW                    EM
SSB                     0
LB                      0 Hz
GB                      0
PC                   1.00
```

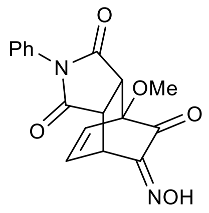

7l

$^{13}C\{1H\}$ and DEPT 90, 135 NMR of 7l

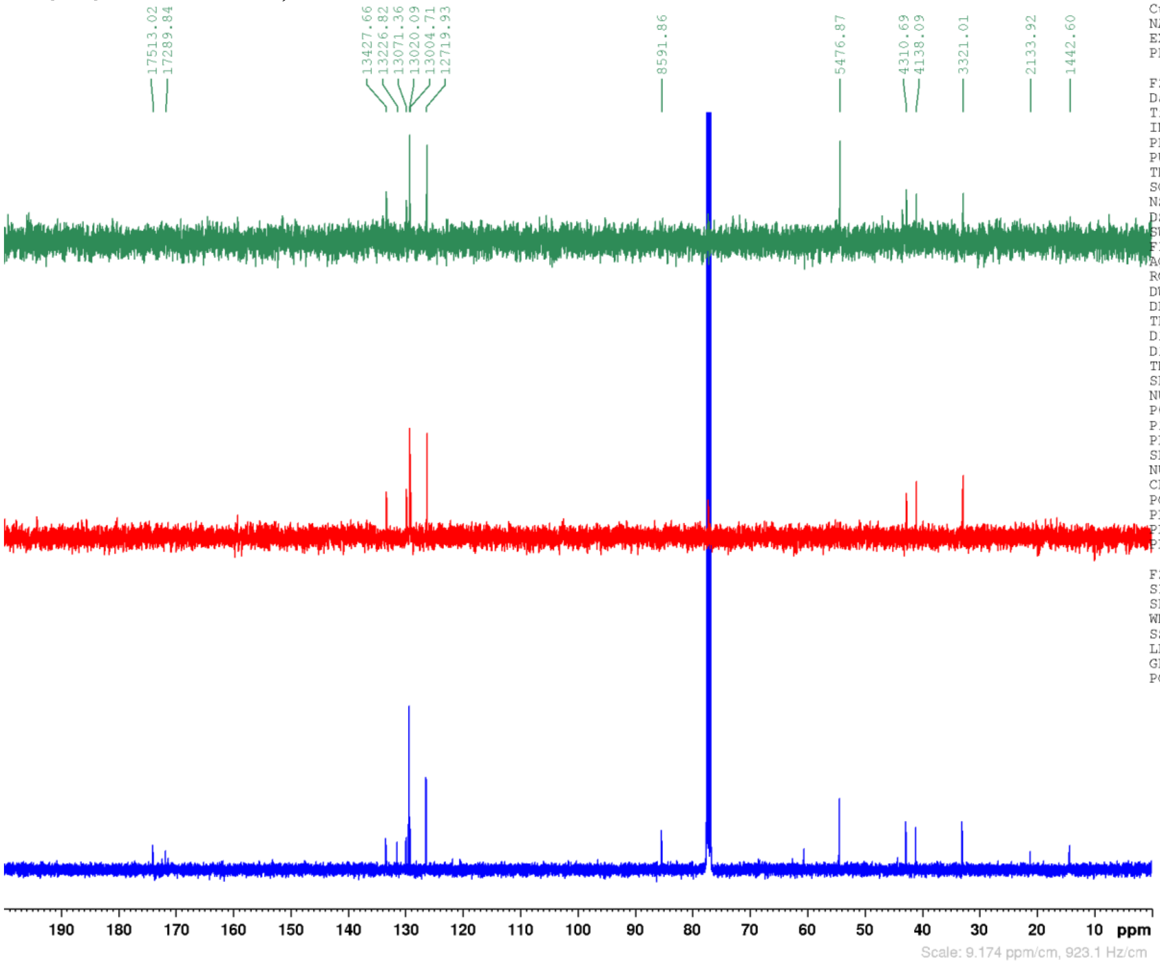


```
Current Data Parameters
NAME      20240521 5-OMe-T
EXPNO                   14
PROCNO                   1

F2 - Acquisition Parameters
Date_             20240522
Time                  7.28 h
INSTRUM   Avance NANOBAY
PROBHD    Z163739_0358 (
PULPROG            zgpg30
TD                  65536
SOLVENT             CDCl3
NS                   5928
DS                      0
SWH             25000.000 Hz
FIDRES           0.762939 Hz
AQ              1.3107200 sec
RG                    101
DW                 20.000 usec
DE                   6.50 usec
TE                  295.8 K
D1             1.50000000 sec
D11            0.03000000 sec
TD0                     1
SFO1          100.6293690 MHz
NUC1                  13C
P0                   2.67 usec
P1                   8.00 usec
PLW1          89.00000000 W
SFO2          400.1518007 MHz
NUC2                   1H
CPDPRG[2          waltz65
PCPD2               90.00 usec
PLW2          22.79999924 W
PLW12          0.18015000 W
PLW13          0.09061300 W

F2 - Processing parameters
SI                  32768
SF            100.6177845 MHz
WDW                    EM
SSB                     0
LB                   0.30 Hz
GB                      0
PC                   1.40
```

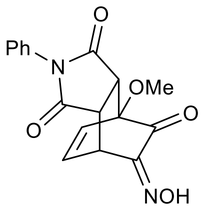

7l

^{1}H NMR of 7m

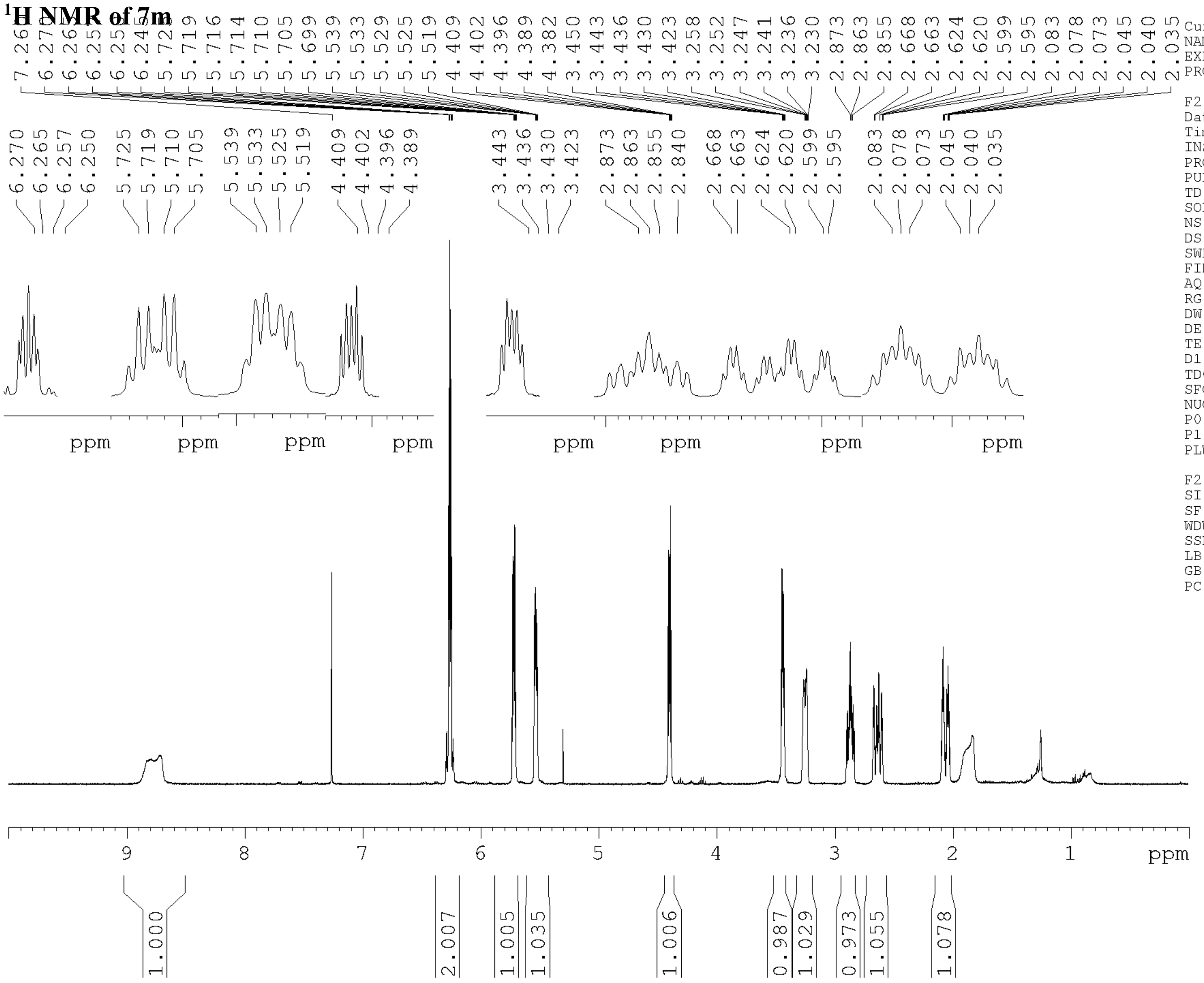

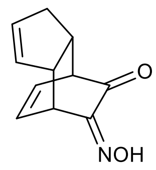

7m

^{13}C{1H} and DEPT 90, 135 NMR of 7m

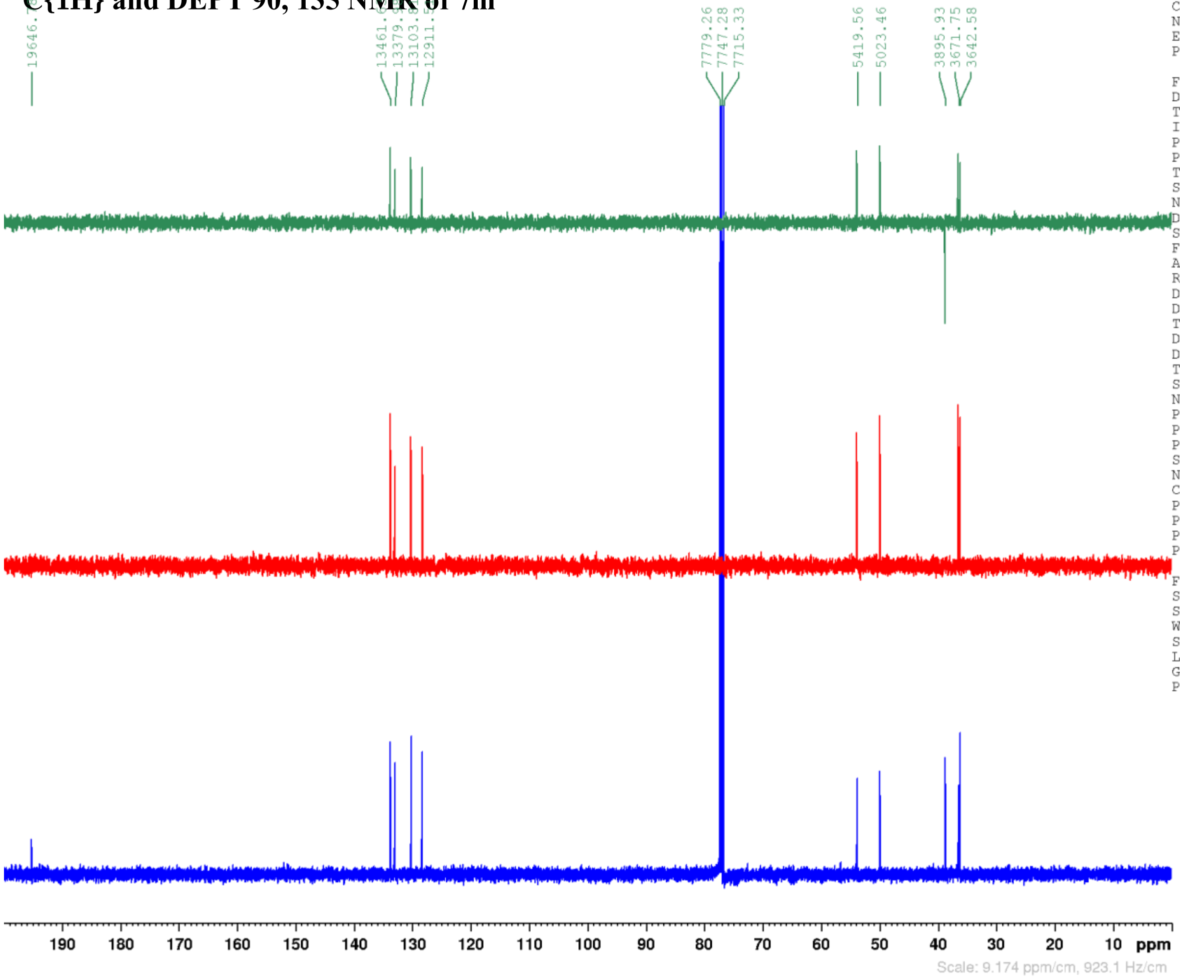

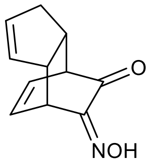

7m

^{1}H NMR of 7o

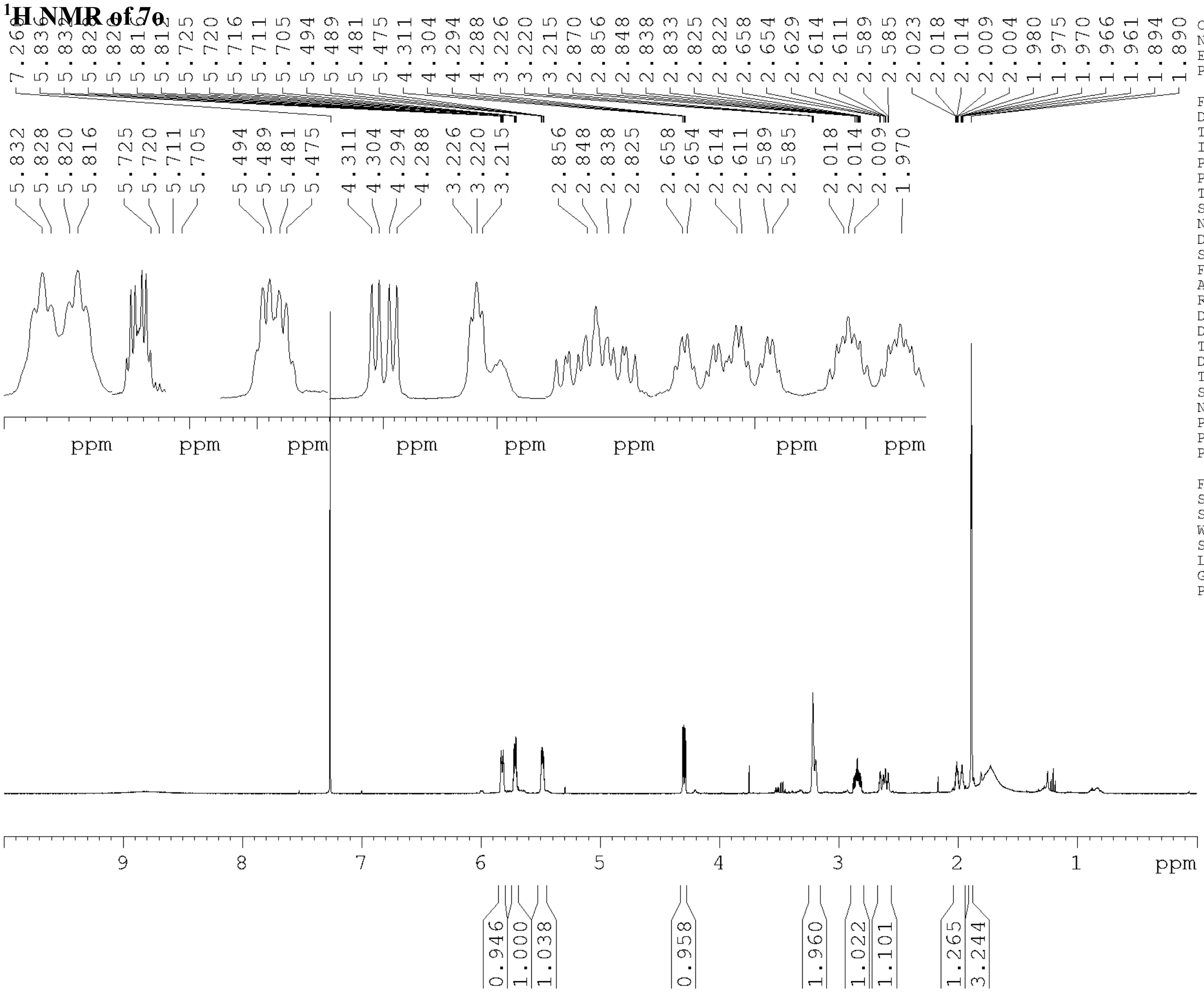


```
Current Data Parameters
NAME      20240508 4-Me-C
EXPNO                   2
PROCNO                  1

F2 - Acquisition Parameters
Date_            20240508
Time                17.20 h
INSTRUM   Avance NANOBAY
PROBHD    Z163739_0358 (
PULPROG              zg30
TD                  32768
SOLVENT             CDCl3
NS                     16
DS                      0
SWH              5882.353 Hz
FIDRES           0.359030 Hz
AQ              2.7852800 sec
RG                    101
DW                 85.000 usec
DE                   9.26 usec
TE                  297.3 K
D1             1.50000000 sec
TD0                     1
SFO1          400.1526010 MHz
NUC1                   1H
P0                   2.67 usec
P1                   8.00 usec
PLW1          21.10000038 W

F2 - Processing parameters
SI                  32768
SF            400.1500095 MHz
WDW                    EM
SSB                     0
LB                   0.10 Hz
GB                      0
PC                   1.00
```

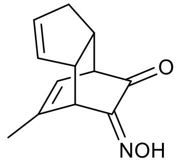

7o

$^{13}C\{1H\}$ and DEPT 90, 135 NMR of 7o

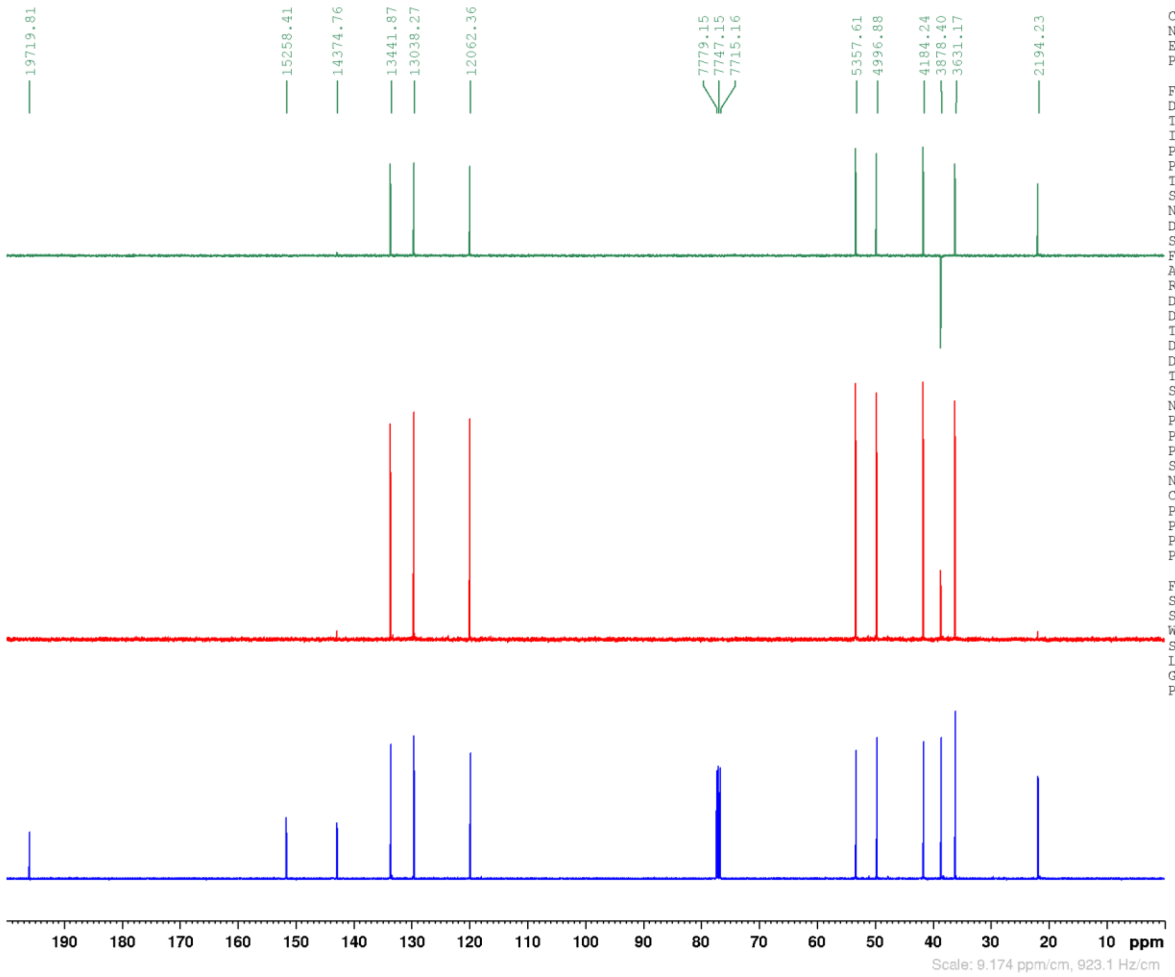

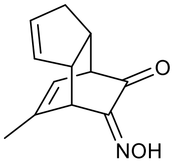

7o

^{1}H NMR of 7p

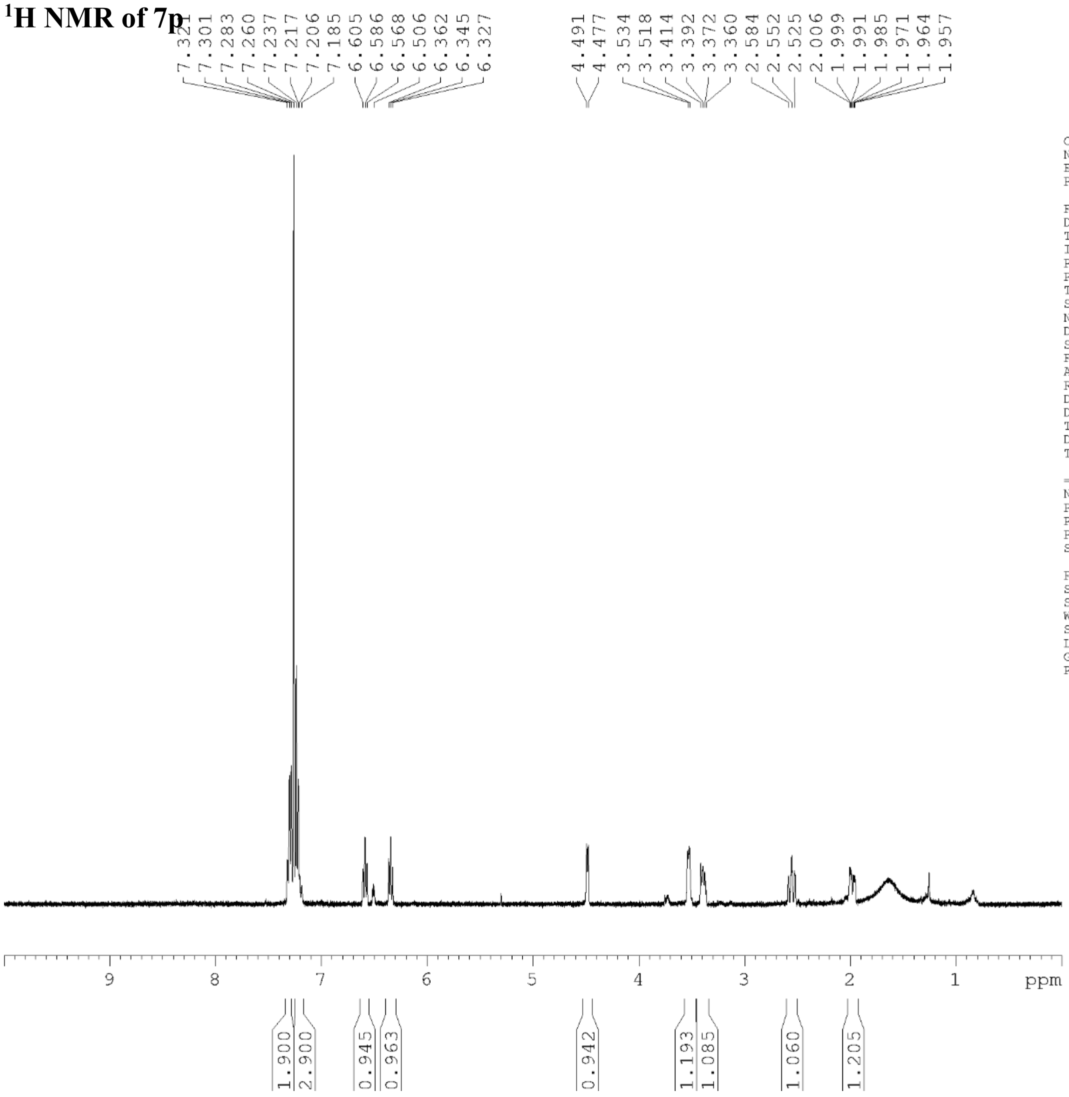

```
Current Data Parameters
NAME     20240308 SZ286
EXPNO                 1
PROCNO                1

F2 - Acquisition Parameters
Date_          20240308
Time              14.55
INSTRUM           spect
PROBHD   5 mm BBO BB-1H
PULPROG            zg30
TD                32768
SOLVENT           CDCl3
NS                   16
DS                    0
SWH            6009.615 Hz
FIDRES         0.183399 Hz
AQ            2.7262976 sec
RG                  287
DW               83.200 usec
DE                 6.50 usec
TE                294.7 K
D1           1.50000000 sec
TD0                   1

======== CHANNEL f1 ========
NUC1                 1H
P1                14.00 usec
PL1               -1.00 dB
PL1W         7.55784369 W
SFO1       400.1326010 MHz

F2 - Processing parameters
SI                32768
SF          400.1300099 MHz
WDW                  EM
SSB                   0
LB                    0 Hz
GB                    0
PC                 1.00
```

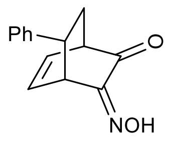

7p

^{13}C{1H} and DEPT 90, 135 NMR of 7p

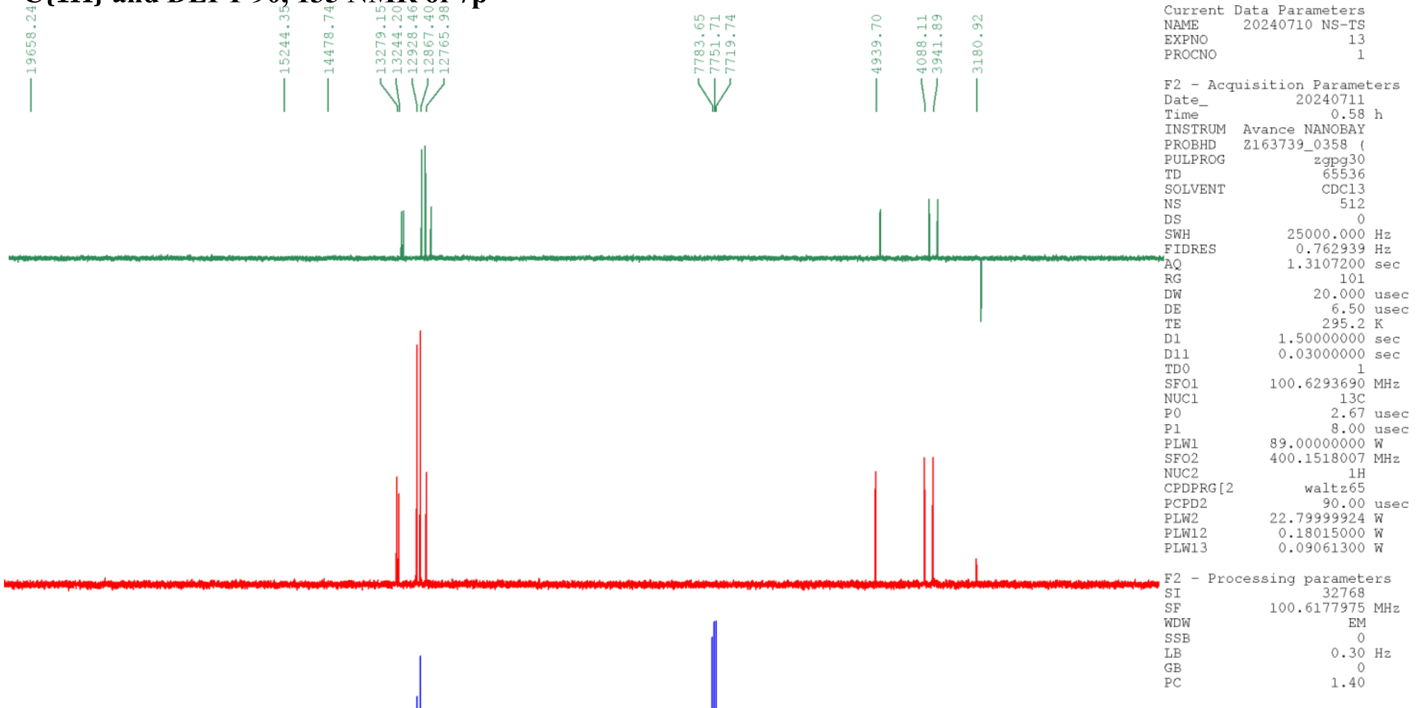

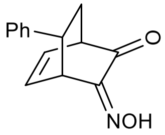

7p

^{1}H NMR of 7q

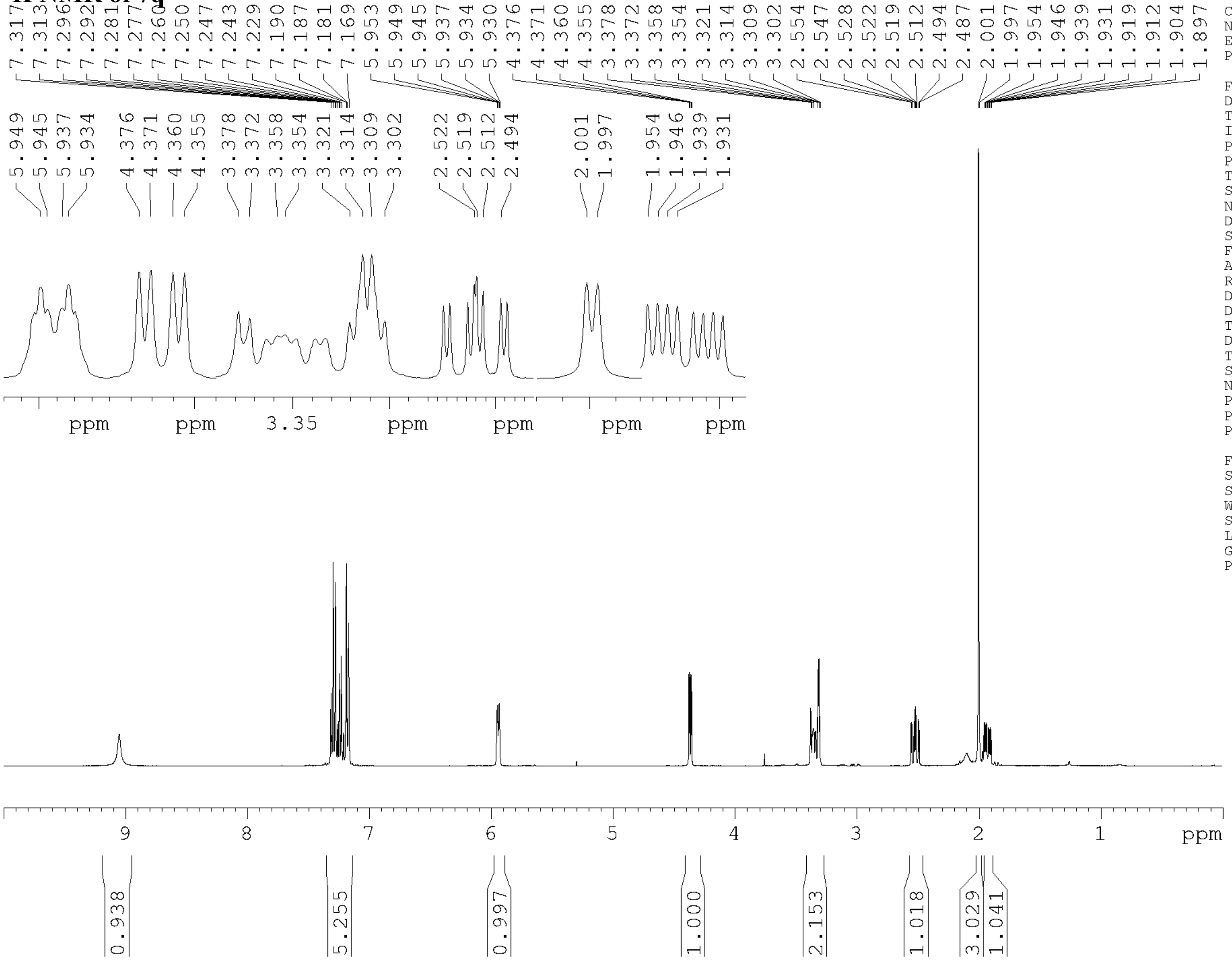

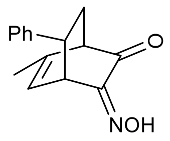

7q

^{13}C{1H} and DEPT 90, 135 NMR of 7q

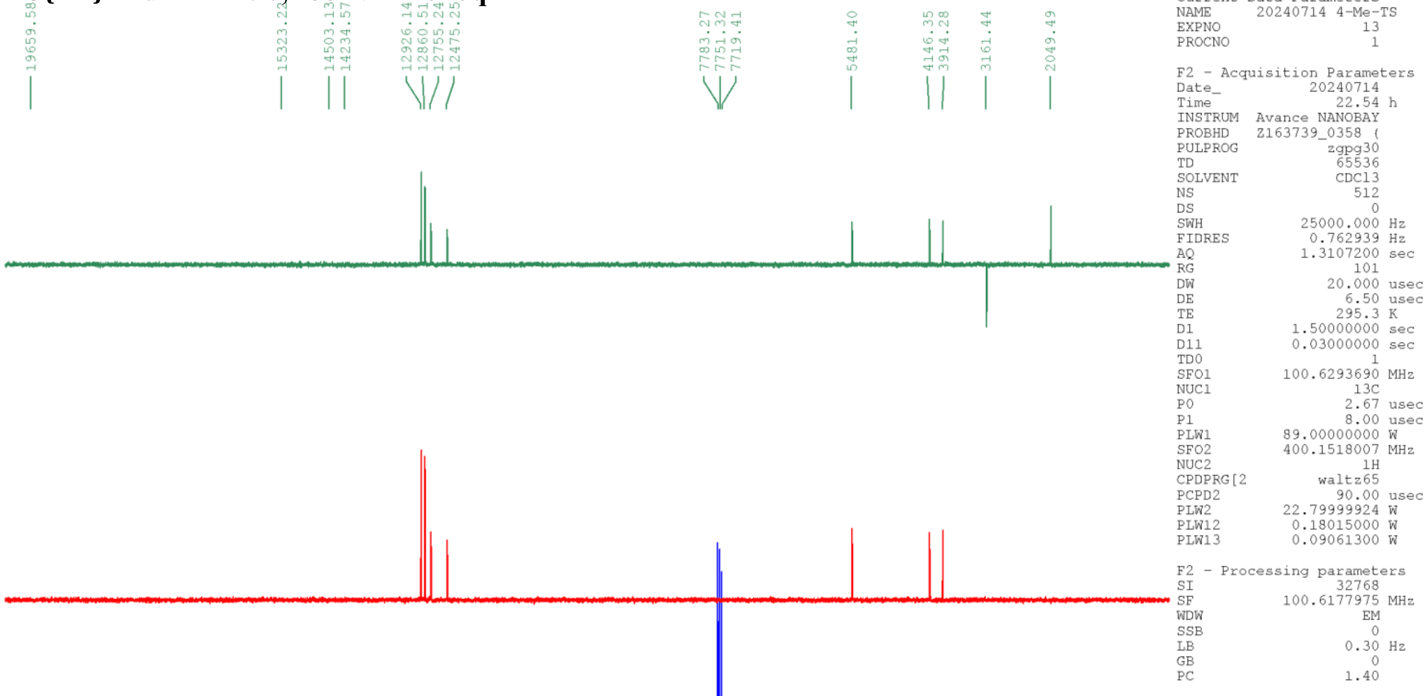

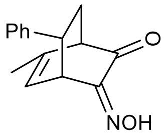

7q

^{1}H NMR of 7r

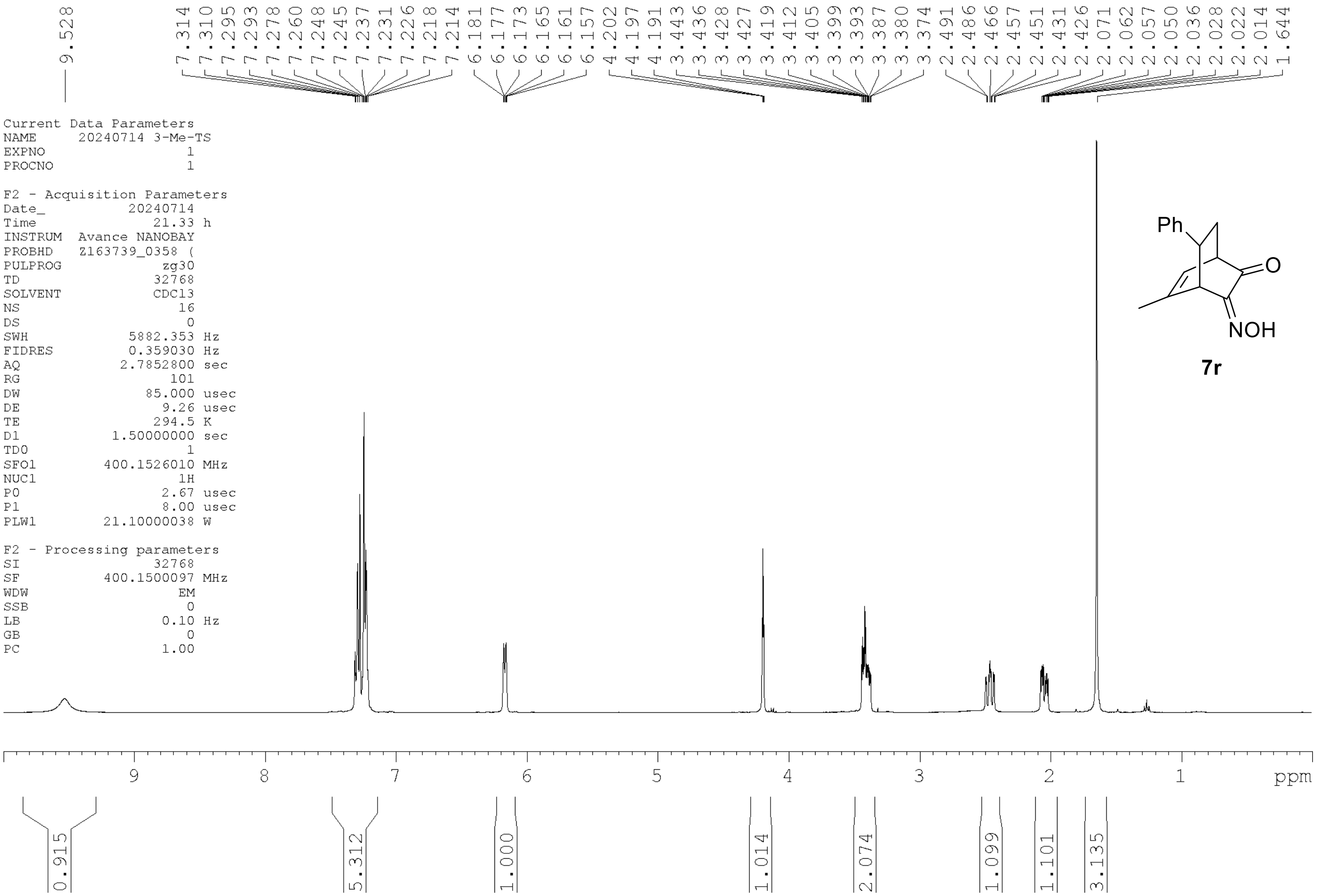
9.528
7.314 7.310 7.295 7.293 7.278 7.260 7.248 7.245 7.237 7.231 7.226 7.218 7.214
6.181 6.177 6.173 6.165 6.161 6.157
4.202 4.197 4.191
3.443 3.436 3.428 3.427 3.419 3.412 3.405 3.399 3.393 3.387 3.380 3.374
2.491 2.486 2.466 2.457 2.451 2.431 2.426
2.071 2.062 2.057 2.050 2.036 2.028 2.022 2.014
1.644
Current Data Parameters
NAME 20240714 3-Me-TS
EXPNO 1
PROCNO 1
F2 - Acquisition Parameters
Date_ 20240714
Time 21.33 h
INSTRUM Avance NANOBAY
PROBHD Z163739_0358 (
PULPROG zg30
TD 32768
SOLVENT CDCl3
NS 16
DS 0
SWH 5882.353 Hz
FIDRES 0.359030 Hz
AQ 2.7852800 sec
RG 101
DW 85.000 usec
DE 9.26 usec
TE 294.5 K
D1 1.50000000 sec
TD0 1
SFO1 400.1526010 MHz
NUC1 1H
P0 2.67 usec
P1 8.00 usec
PLW1 21.10000038 W
F2 - Processing parameters
SI 32768
SF 400.1500097 MHz
WDW EM
SSB 0
LB 0.10 Hz
GB 0
PC 1.00
Ph
O
NOH
7r
9
8
7
6
5
4
3
2
1
ppm
0.915
5.312
1.000
1.014
2.074
1.099
1.101
3.135

^{13}C{1H} and DEPT 90, 135 NMR of 7r

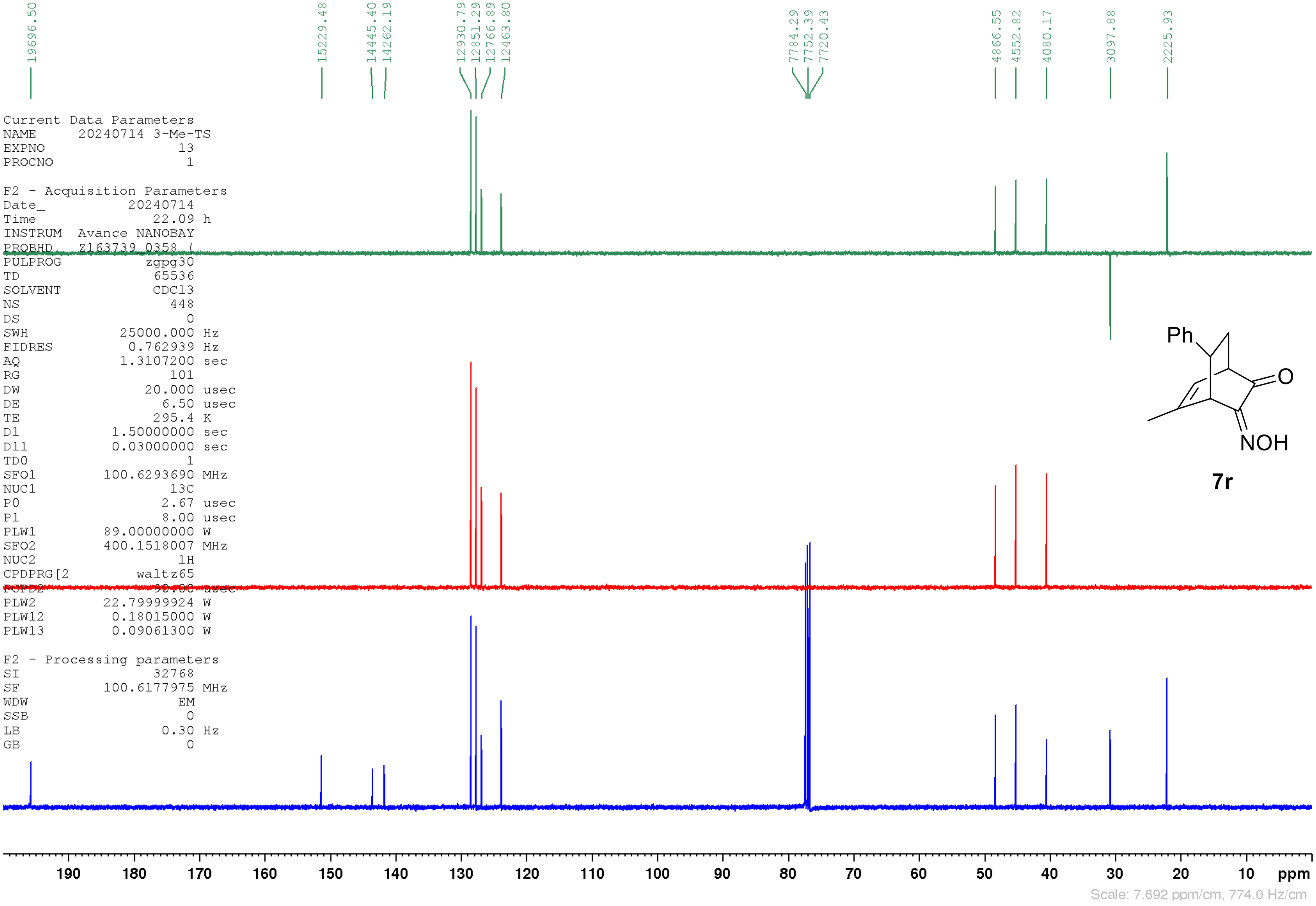

^{1}H NMR of 8a(8h)

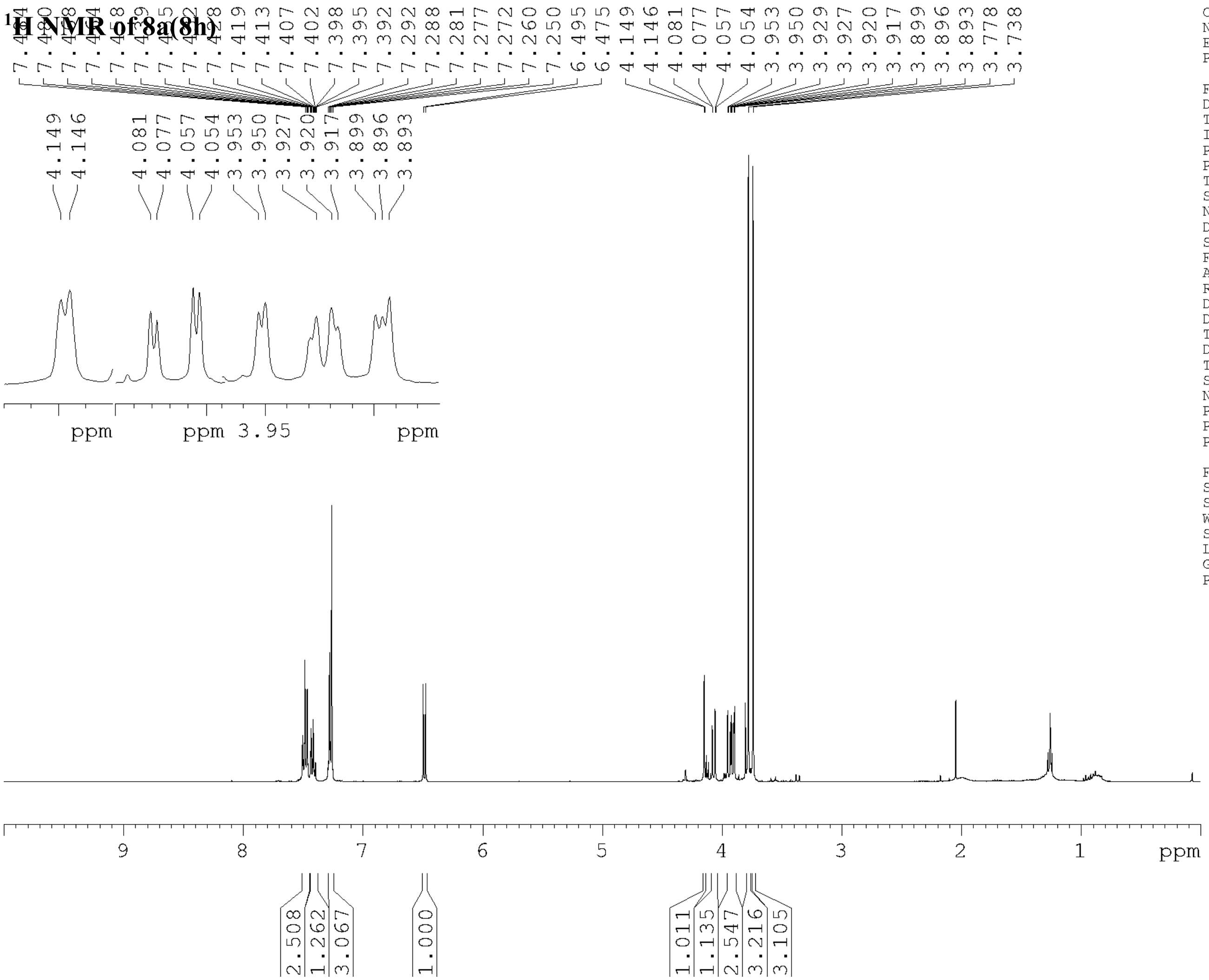

Current Data Parameters
NAME 20240116 SZ262
EXPNO 1
PROCNO 1

F2 - Acquisition Parameters
Date_ 20240116
Time 17.07 h
INSTRUM Avance NANOBAY
PROBHD Z163739_0358 (
PULPROG zg30
TD 32768
SOLVENT CDCl3
NS 16
DS 0
SWH 5882.353 Hz
FIDRES 0.359030 Hz
AQ 2.7852800 sec
RG 101
DW 85.000 usec
DE 9.26 usec
TE 295.3 K
D1 1.50000000 sec
TD0 1
SFO1 400.1526010 MHz
NUC1 1H
P0 2.67 usec
P1 8.00 usec
PLW1 21.10000038 W

F2 - Processing parameters
SI 32768
SF 400.1500099 MHz
WDW EM
SSB 0
LB 0.10 Hz
GB 0
PC 1.00

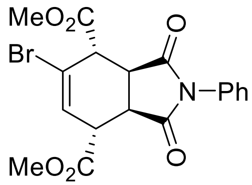

8a(8h)

^{13}C{1H} and DEPT 90, 135 NMR of 8a(8h)

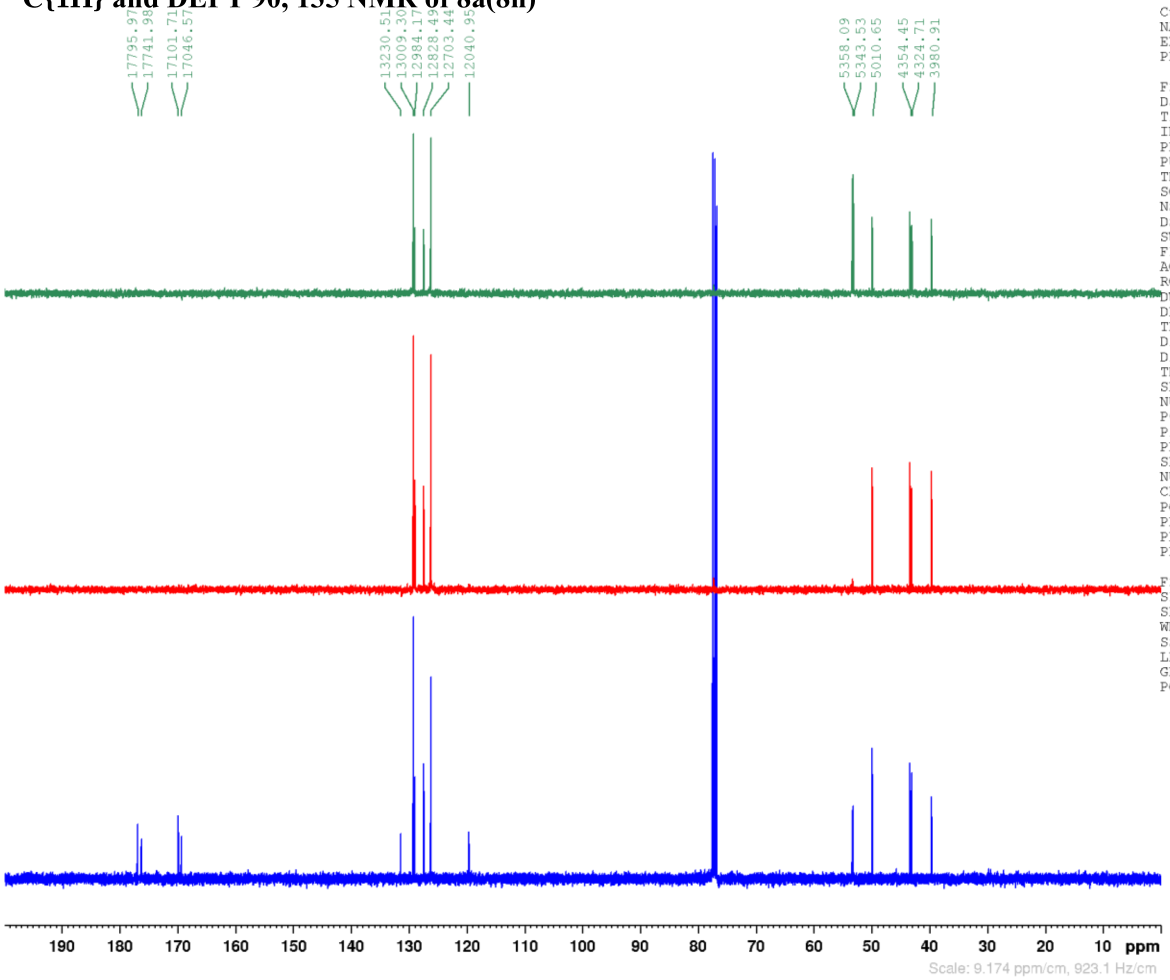

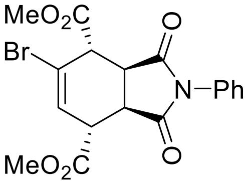

8a(8h)

^{1}H NMR of 8b

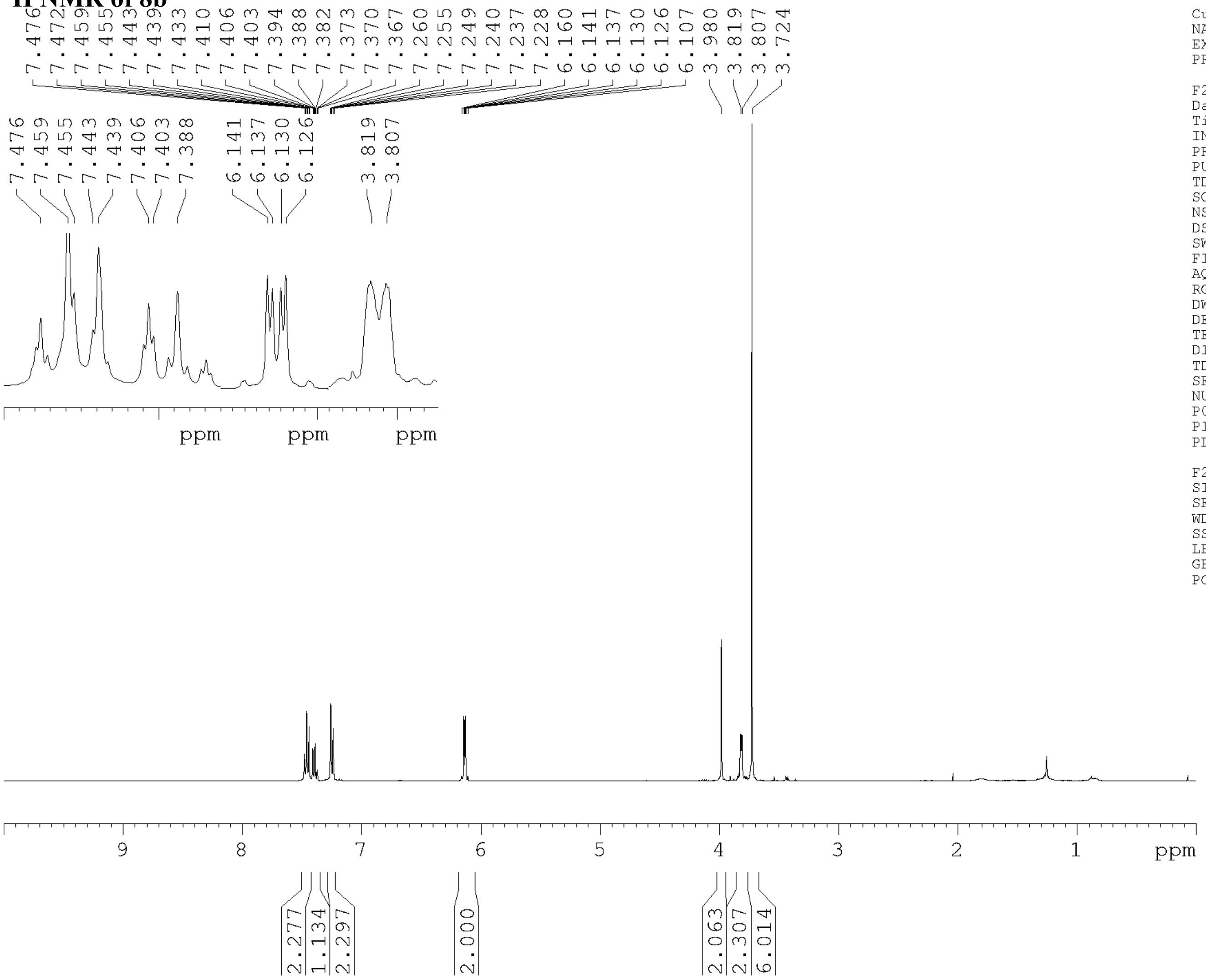


```
Current Data Parameters
NAME     20231109 SZ225-C
EXPNO                   2
PROCNO                  1

F2 - Acquisition Parameters
Date_            20231109
Time                23.28 h
INSTRUM   Avance NANOBAY
PROBHD   Z163739_0358 (
PULPROG              zg30
TD                  32768
SOLVENT             CDCl3
NS                      1
DS                      0
SWH              5882.353 Hz
FIDRES           0.359030 Hz
AQ              2.7852800 sec
RG                97.1264
DW                 85.000 usec
DE                   9.26 usec
TE                  296.0 K
D1             1.50000000 sec
TD0                     1
SFO1          400.1526010 MHz
NUC1                   1H
P0                   2.67 usec
P1                   8.00 usec
PLW1          21.10000038 W

F2 - Processing parameters
SI                  32768
SF            400.1500097 MHz
WDW                    EM
SSB                     0
LB                   0.10 Hz
GB                      0
PC                   1.00
```

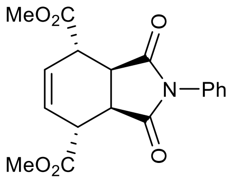

8b

^{13}C{1H} and DEPT 90, 135 NMR of 8b

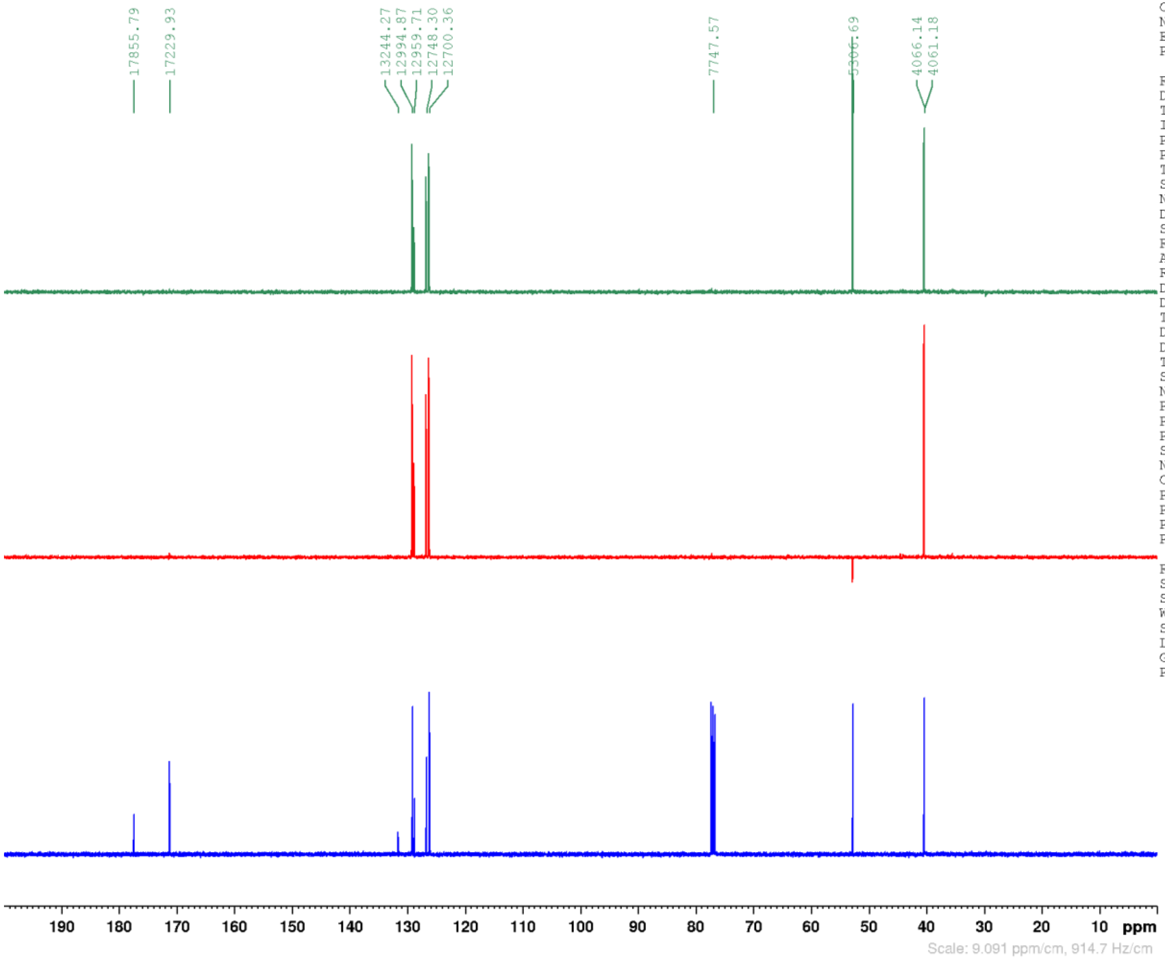


```
Current Data Parameters
NAME       20231109 SZ225-C
EXPNO                    13
PROCNO                    1

F2 - Acquisition Parameters
Date_              20231109
Time                  23.49 h
INSTRUM    Avance NANOBAY
PROBHD     Z163739_0358 (
PULPROG              zgpg30
TD                    65536
SOLVENT               CDCl3
NS                      128
DS                       16
SWH               25000.000 Hz
FIDRES             0.762939 Hz
AQ                1.3107200 sec
RG                      101
DW                   20.000 usec
DE                     6.50 usec
TE                    297.0 K
D1               1.50000000 sec
D11              0.03000000 sec
TD0                       1
SFO1            100.6293690 MHz
NUC1                    13C
P0                     2.67 usec
P1                     8.00 usec
PLW1            89.00000000 W
SFO2            400.1518007 MHz
NUC2                     1H
CPDPRG[2            waltz65
PCPD2                 90.00 usec
PLW2            22.79999924 W
PLW12            0.18015000 W
PLW13            0.09061300 W

F2 - Processing parameters
SI                    32768
SF              100.6178036 MHz
WDW                      EM
SSB                       0
LB                     0.30 Hz
GB                        0
PC                     1.40
```

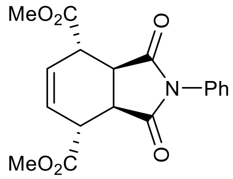

8b

^{1}H NMR of 8c(8g)

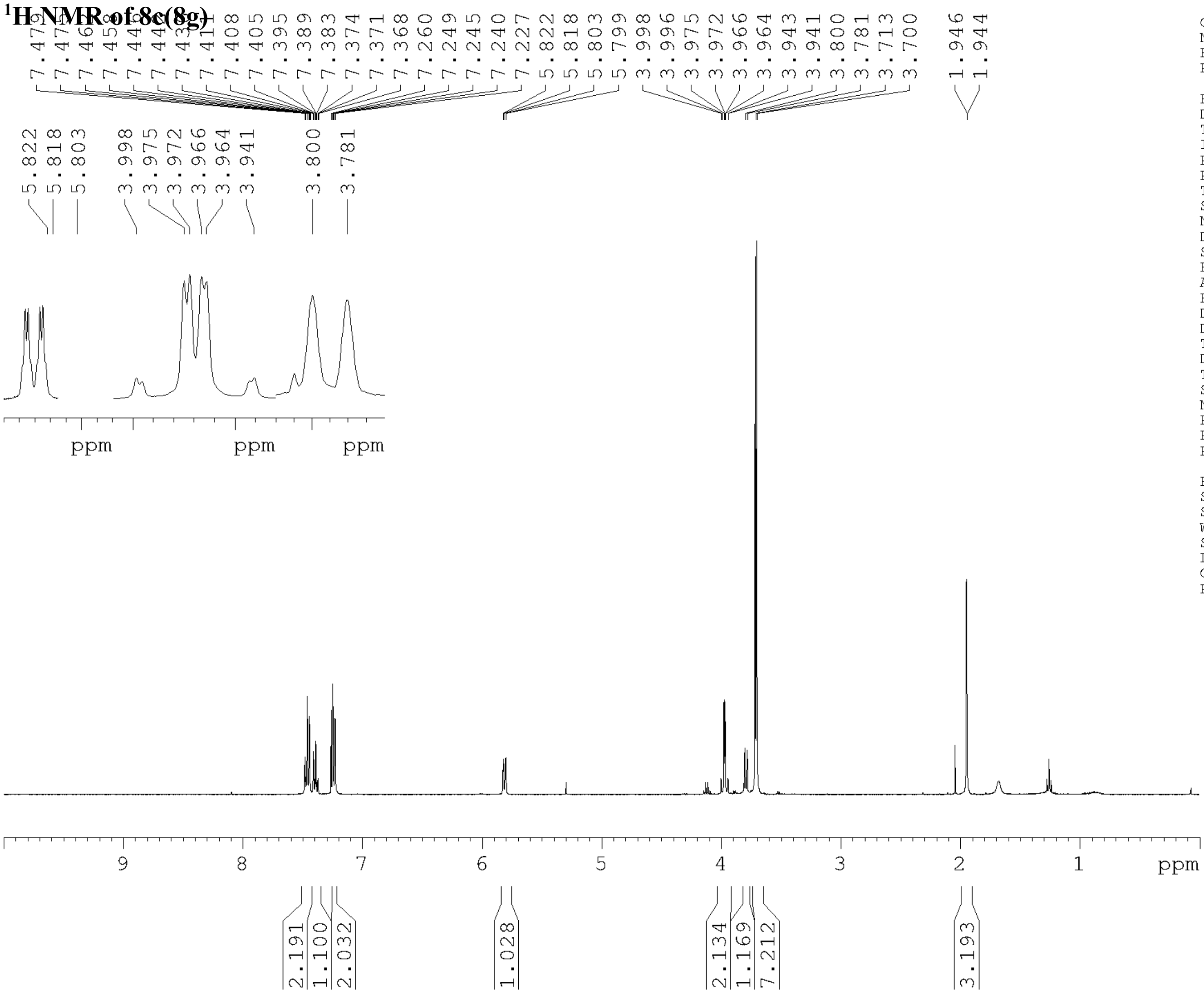


```
Current Data Parameters
NAME     20240109 3-Me-OR-DT
EXPNO                  1
PROCNO                 1

F2 - Acquisition Parameters
Date_           20240110
Time                0.03 h
INSTRUM  Avance NANOBAY
PROBHD   Z163739_0358 (
PULPROG             zg30
TD                 32768
SOLVENT            CDCl3
NS                     1
DS                     0
SWH             5882.353 Hz
FIDRES          0.359030 Hz
AQ             2.7852800 sec
RG                   101
DW                85.000 usec
DE                  9.26 usec
TE                 296.9 K
D1            1.50000000 sec
TD0                    1
SFO1        400.1526010 MHz
NUC1                  1H
P0                  2.67 usec
P1                  8.00 usec
PLW1         21.10000038 W

F2 - Processing parameters
SI                 32768
SF          400.1500097 MHz
WDW                   EM
SSB                    0
LB                  0.10 Hz
GB                     0
PC                  1.00
```

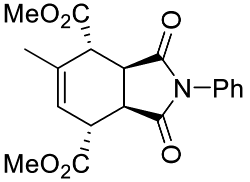

8c(8g)

^{13}C{1H} and DEPT 90, 135 NMR of 8c(8g)

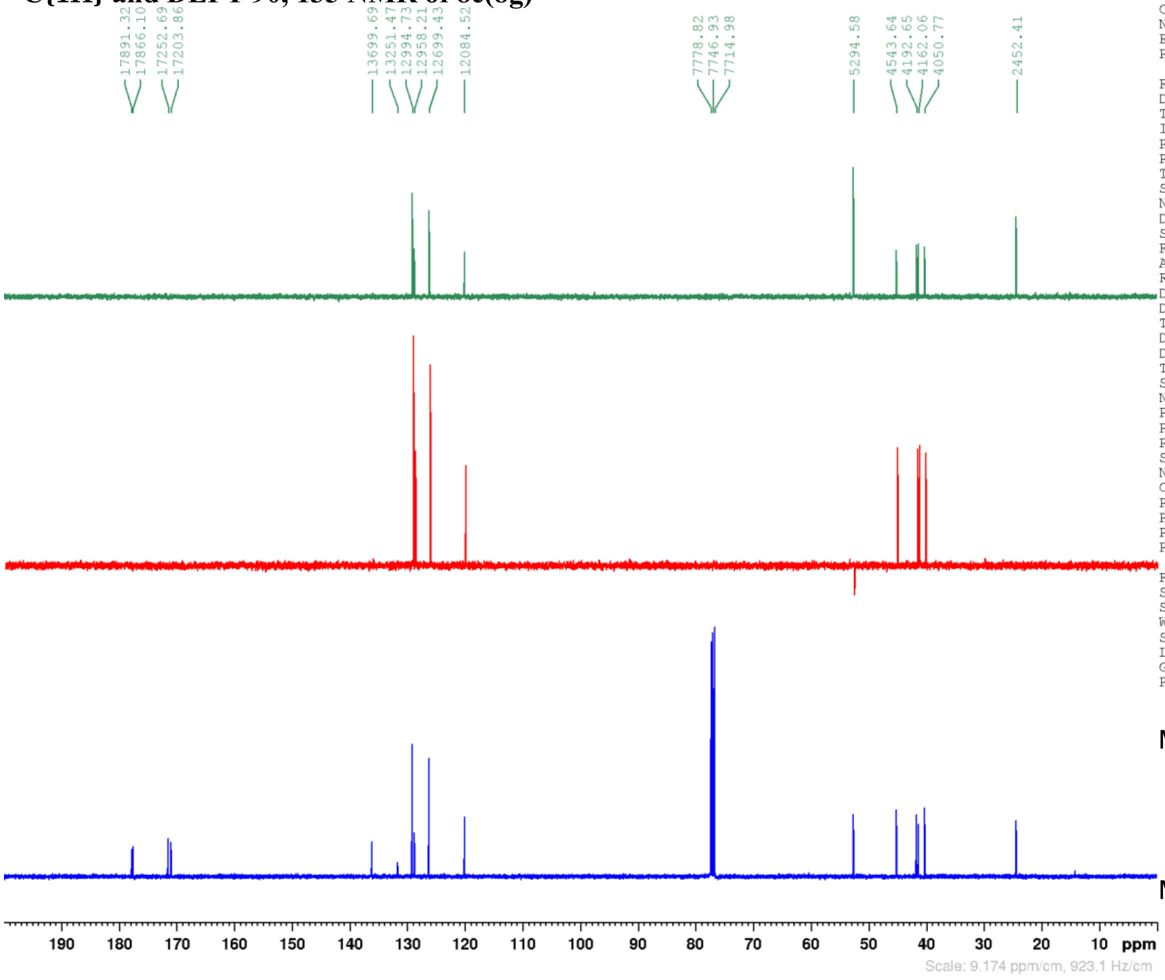


```
Current Data Parameters
NAME        20240109 3-Me-OR-DT
EXPNO                    13
PROCNO                    1

F2 - Acquisition Parameters
Date_              20240110
Time                   0.28 h
INSTRUM    Avance NANOBAY
PROBHD     Z163739_0358 (
PULPROG              zgpg30
TD                    65536
SOLVENT               CDCl3
NS                      512
DS                        0
SWH               25000.000 Hz
FIDRES             0.762939 Hz
AQ                1.3107200 sec
RG                      101
DW                   20.000 usec
DE                     6.50 usec
TE                    297.1 K
D1               1.50000000 sec
D11              0.03000000 sec
TD0                       1
SFO1            100.6293690 MHz
NUC1                    13C
P0                     2.67 usec
P1                     8.00 usec
PLW1            89.00000000 W
SFO2            400.1518007 MHz
NUC2                     1H
CPDPRG[2            waltz65
PCPD2                 90.00 usec
PLW2            22.79999924 W
PLW12            0.18015000 W
PLW13            0.09061300 W

F2 - Processing parameters
SI                    32768
SF              100.6178014 MHz
WDW                      EM
SSB                       0
LB                     0.30 Hz
GB                        0
PC                     1.40
```

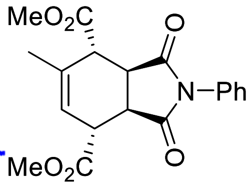

8c(8g)

^{1}H NMR of 8d(8i)

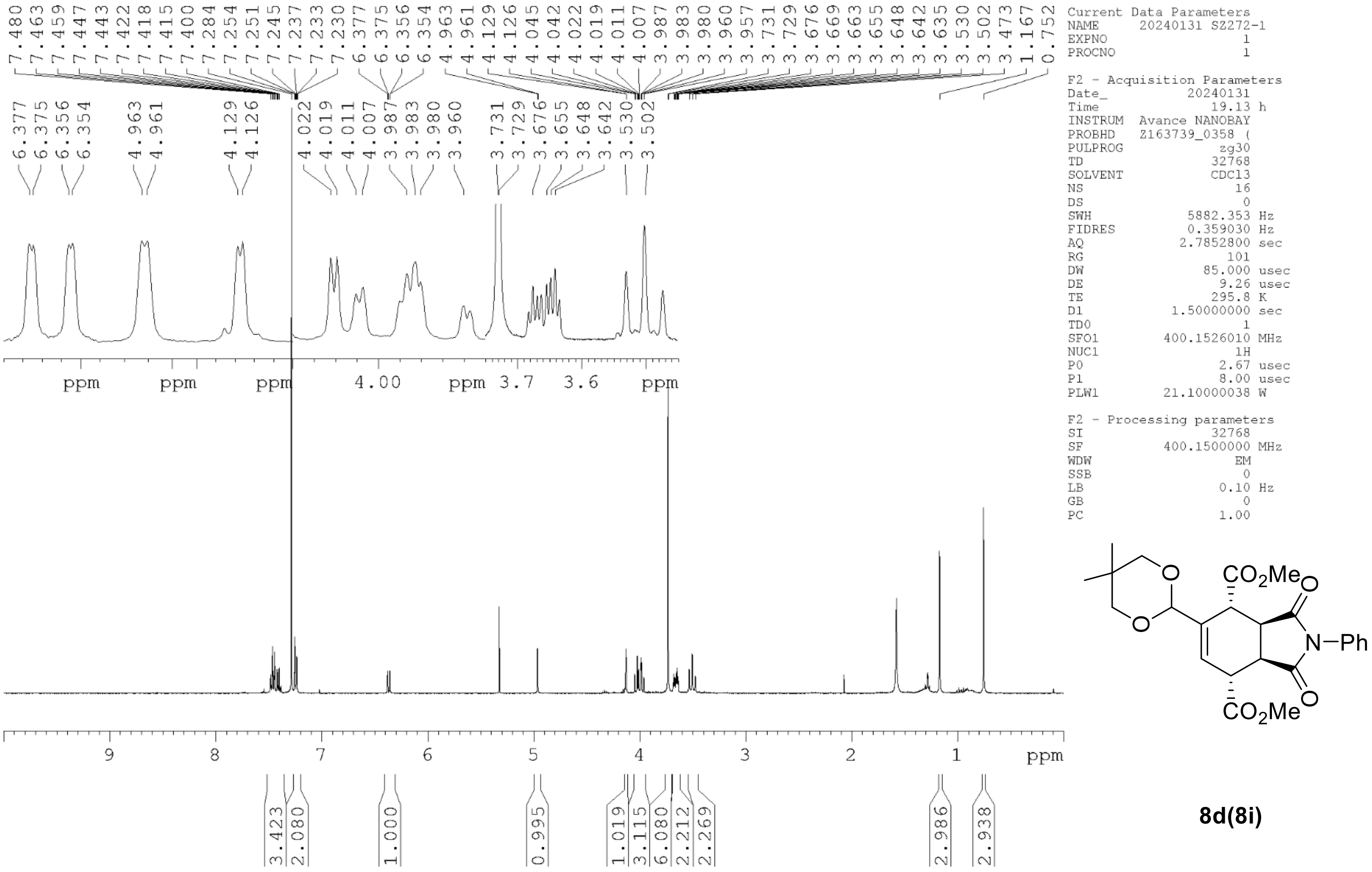

8d(8i)

$^{13}C\{1H\}$ and DEPT 90, 135 NMR of 8d(8i)

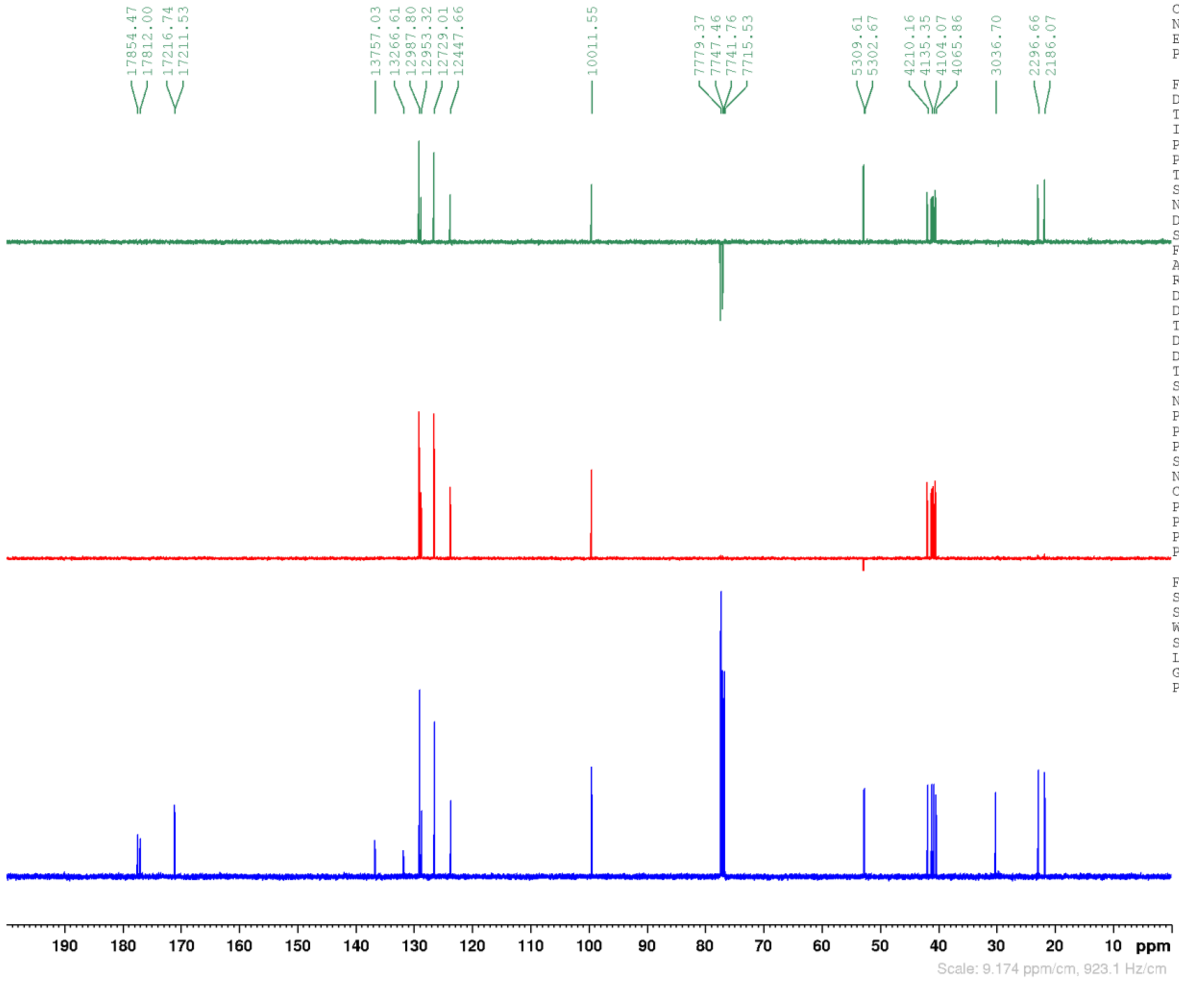

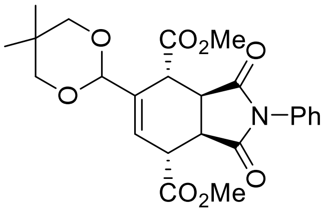

8d(8i)

^{1}H NMR of 8e

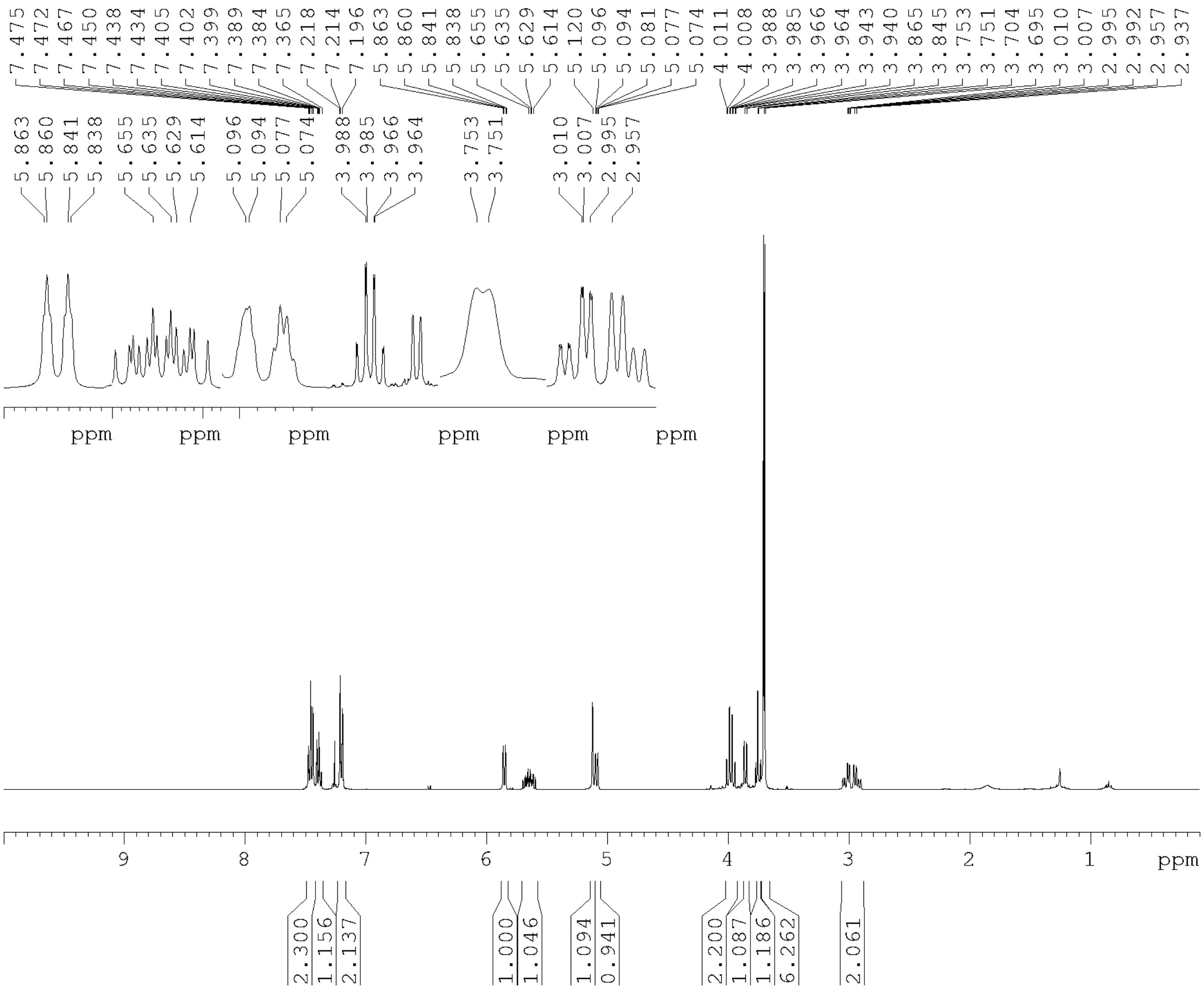


```
Current Data Parameters
NAME     20231212 4-Allyl-Openring
EXPNO                 1
PROCNO                1

F2 - Acquisition Parameters
Date_            20231128
Time                17.22 h
INSTRUM  Avance NANOBAY
PROBHD   Z163739_0358 (
PULPROG              zg30
TD                  32768
SOLVENT             CDCl3
NS                     24
DS                      0
SWH              5882.353 Hz
FIDRES           0.359030 Hz
AQ              2.7852800 sec
RG                     65
DW                 85.000 usec
DE                   9.26 usec
TE                  296.5 K
D1            10.00000000 sec
TD0                     1
SFO1          400.1526010 MHz
NUC1                   1H
P0                   2.67 usec
P1                   8.00 usec
PLW1          23.43799973 W

F2 - Processing parameters
SI                  32768
SF            400.1500097 MHz
WDW                    EM
SSB                     0
LB                   0.10 Hz
GB                      0
PC                   1.00
```

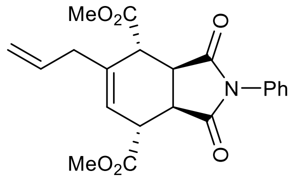

8e

$^{13}C\{1H\}$ and DEPT 90, 135 NMR of 8e

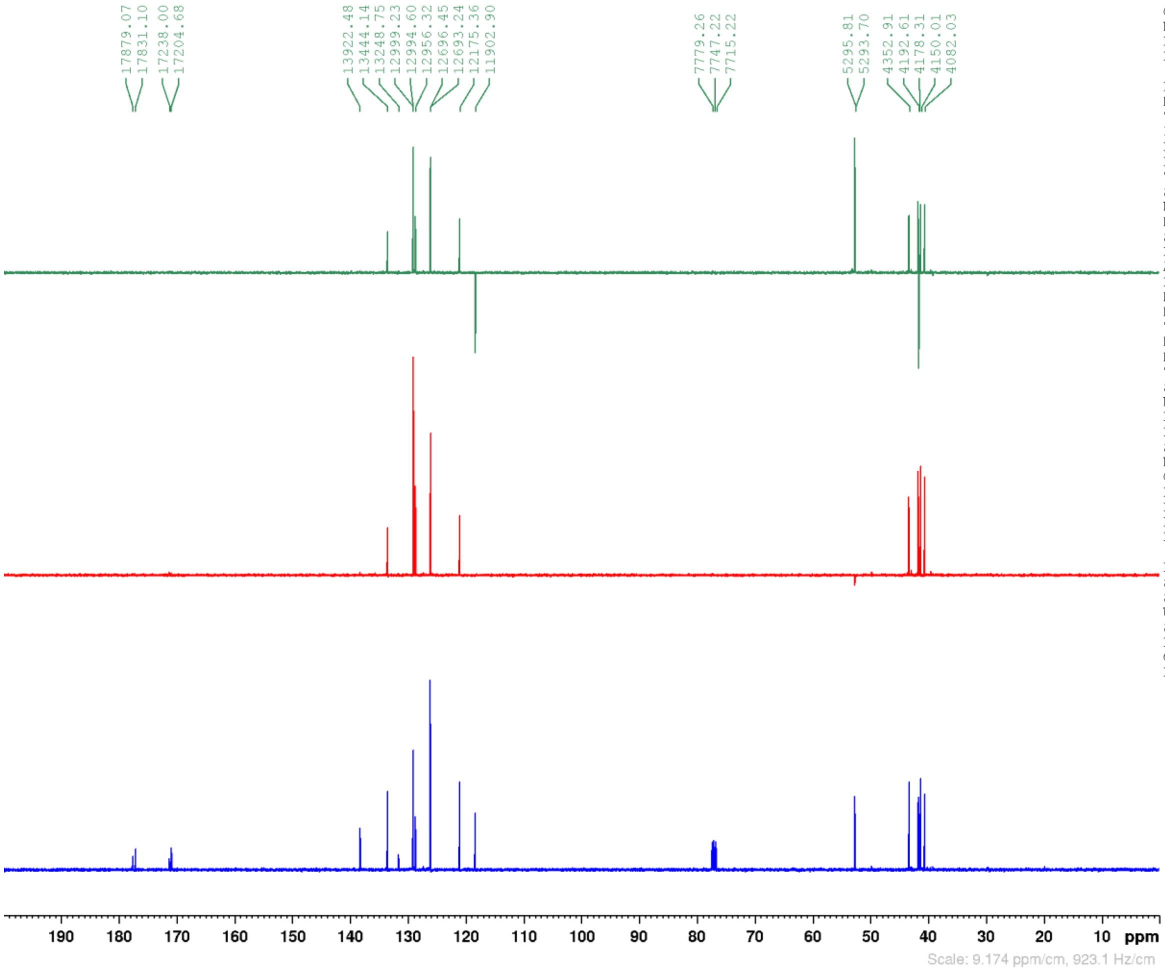

Current Data Parameters
NAME 20231212 4-Allyl-Openring
EXPNO 13
PROCNO 1

F2 - Acquisition Parameters
Date_ 20231128
Time 16.42 h
INSTRUM Avance NANOBAY
PROBHD Z163739_0358 (
PULPROG zgpg
TD 65536
SOLVENT CDCl3
NS 128
DS 0
SWH 25000.000 Hz
FIDRES 0.762939 Hz
AQ 1.3107200 sec
RG 101
DW 20.000 usec
DE 6.50 usec
TE 296.7 K
D1 1.50000000 sec
D11 0.03000000 sec
TD0 1
SFO1 100.6293690 MHz
NUC1 13C
P1 8.00 usec
PLW1 89.00000000 W
SFO2 400.1518007 MHz
NUC2 1H
CPDPRG[2 waltz65
PCPD2 90.00 usec
PLW2 22.79999924 W
PLW12 0.18015000 W
PLW13 0.09061300 W

F2 - Processing parameters
SI 32768
SF 100.6178052 MHz
WDW EM
SSB 0
LB 0.10 Hz
GB 0
PC 1.40

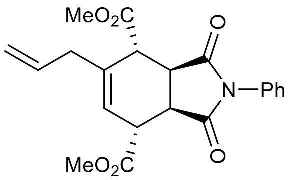

8e

^{1}H NMR of 8f

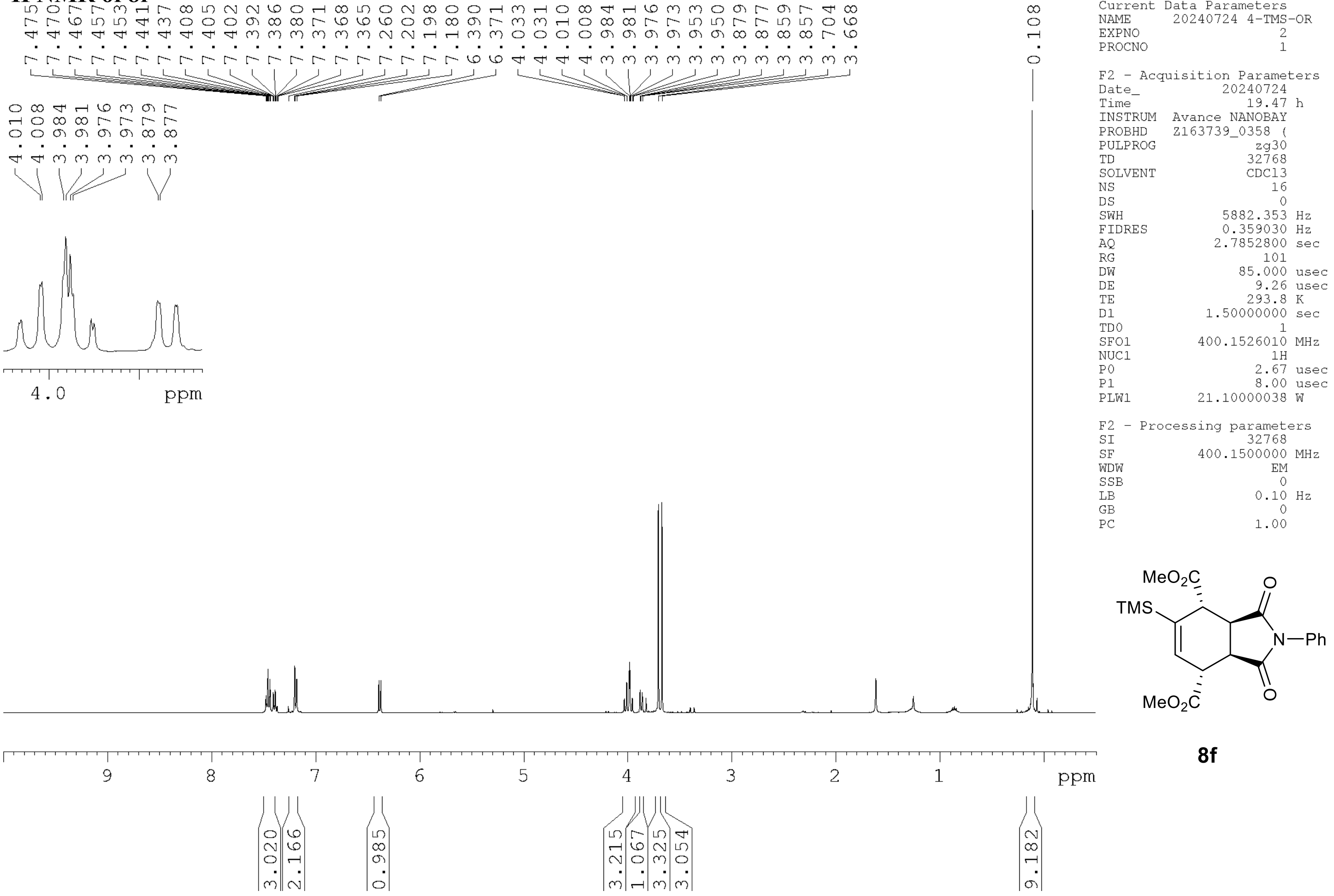

8f

^{13}C{1H} and DEPT 90, 135 NMR of 8f

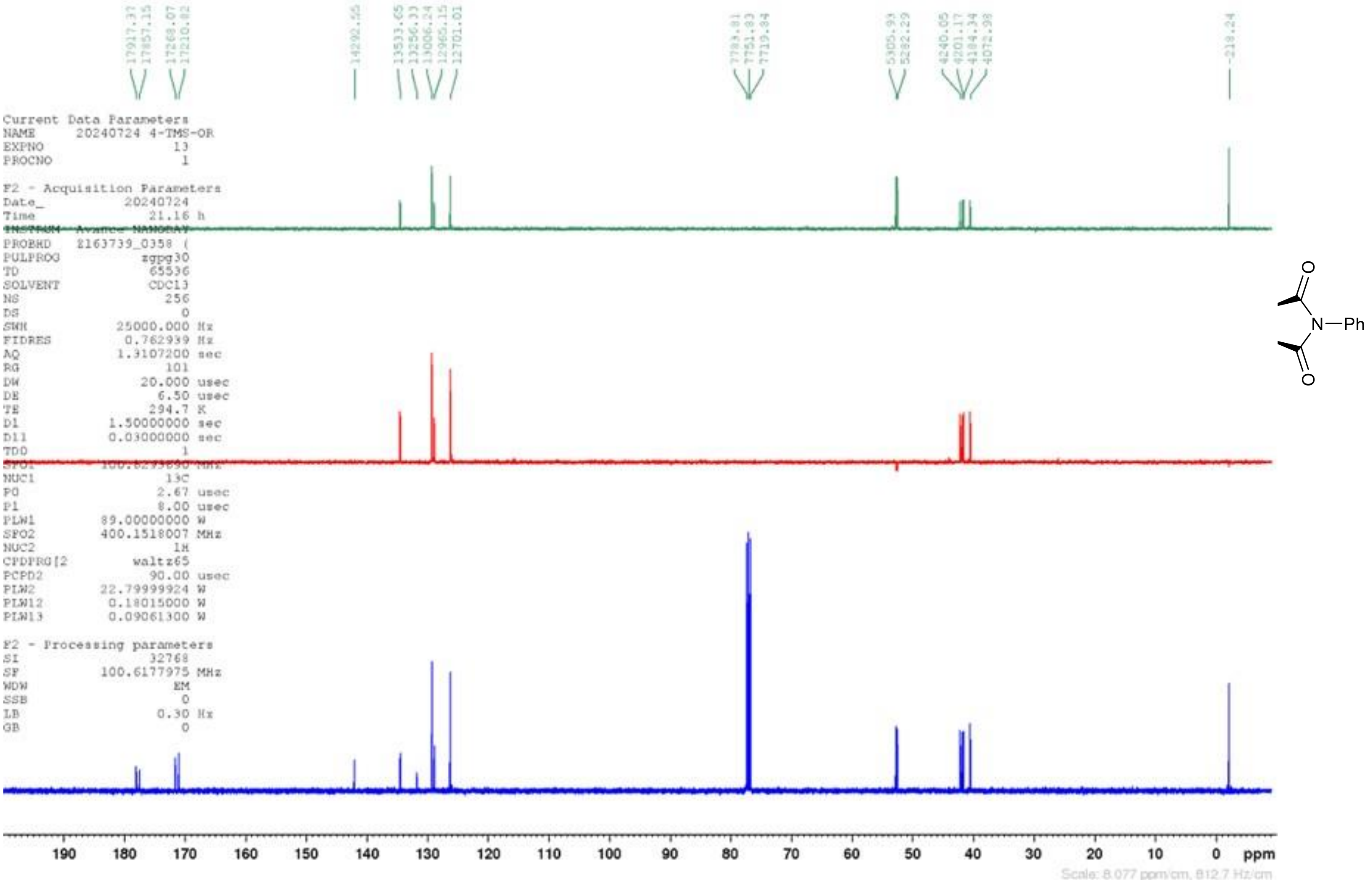

^{1}H NMR of 8l

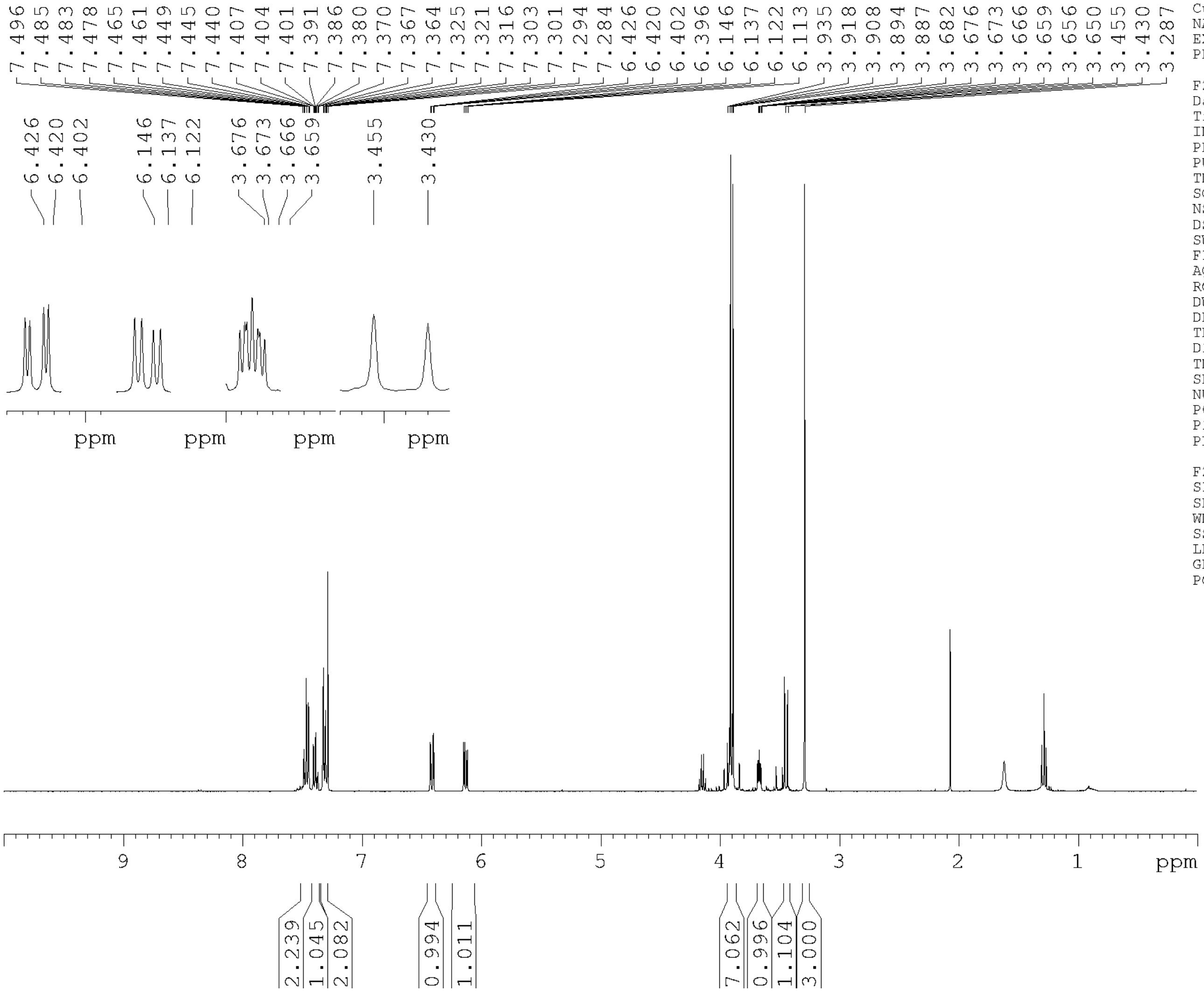

```
Current Data Parameters
NAME      20240410 lu058d
EXPNO                   1
PROCNO                  1

F2 - Acquisition Parameters
Date_            20240410
Time                21.53 h
INSTRUM   Avance NANOBAY
PROBHD    Z163739_0358 (
PULPROG              zg30
TD                  32768
SOLVENT             CDCl3
NS                     16
DS                      0
SWH              5882.353 Hz
FIDRES           0.359030 Hz
AQ              2.7852800 sec
RG                    101
DW                 85.000 usec
DE                   9.26 usec
TE                  297.1 K
D1             1.50000000 sec
TD0                     1
SFO1          400.1526010 MHz
NUC1                   1H
P0                   2.67 usec
P1                   8.00 usec
PLW1          21.10000038 W

F2 - Processing parameters
SI                  32768
SF            400.1500000 MHz
WDW                    EM
SSB                     0
LB                   0.10 Hz
GB                      0
PC                   1.00
```

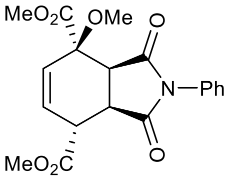

8l

$^{13}C\{1H\}$ and DEPT 90, 135 NMR of 8l

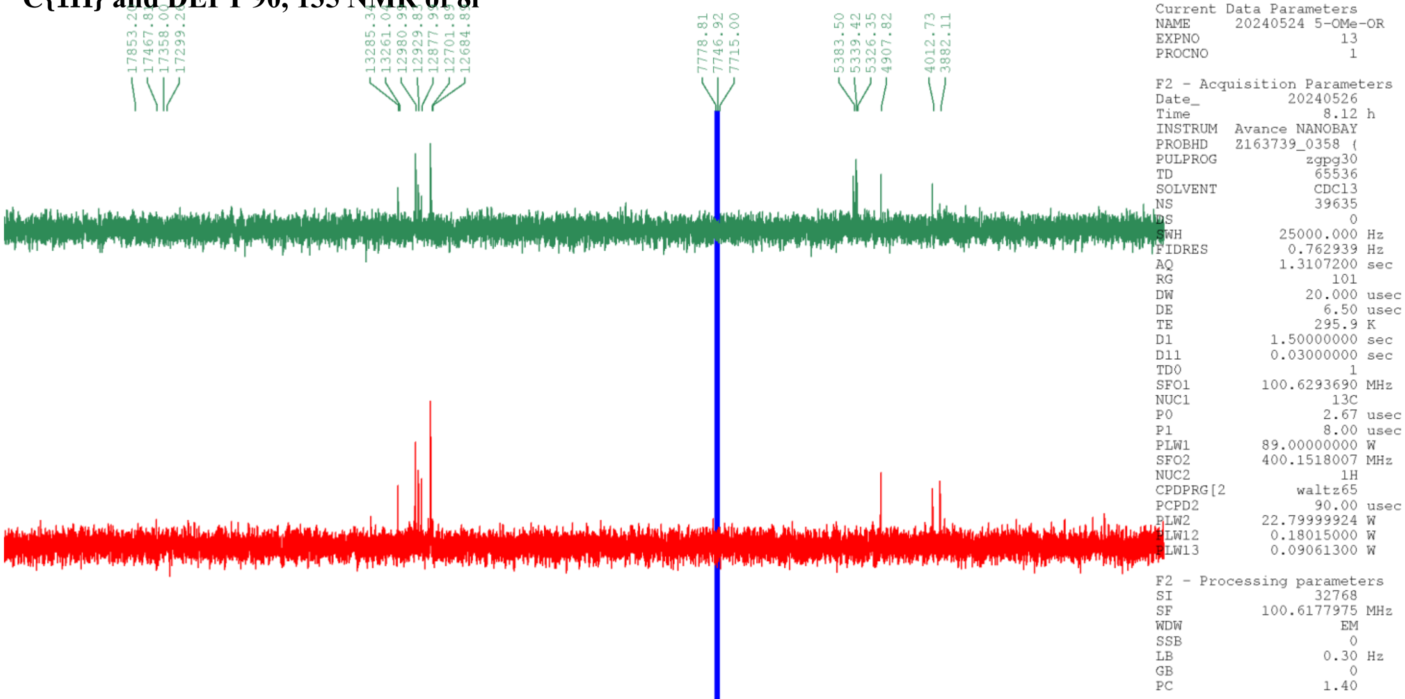

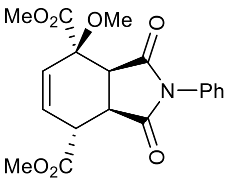

8l

^{1}H NMR of 8m

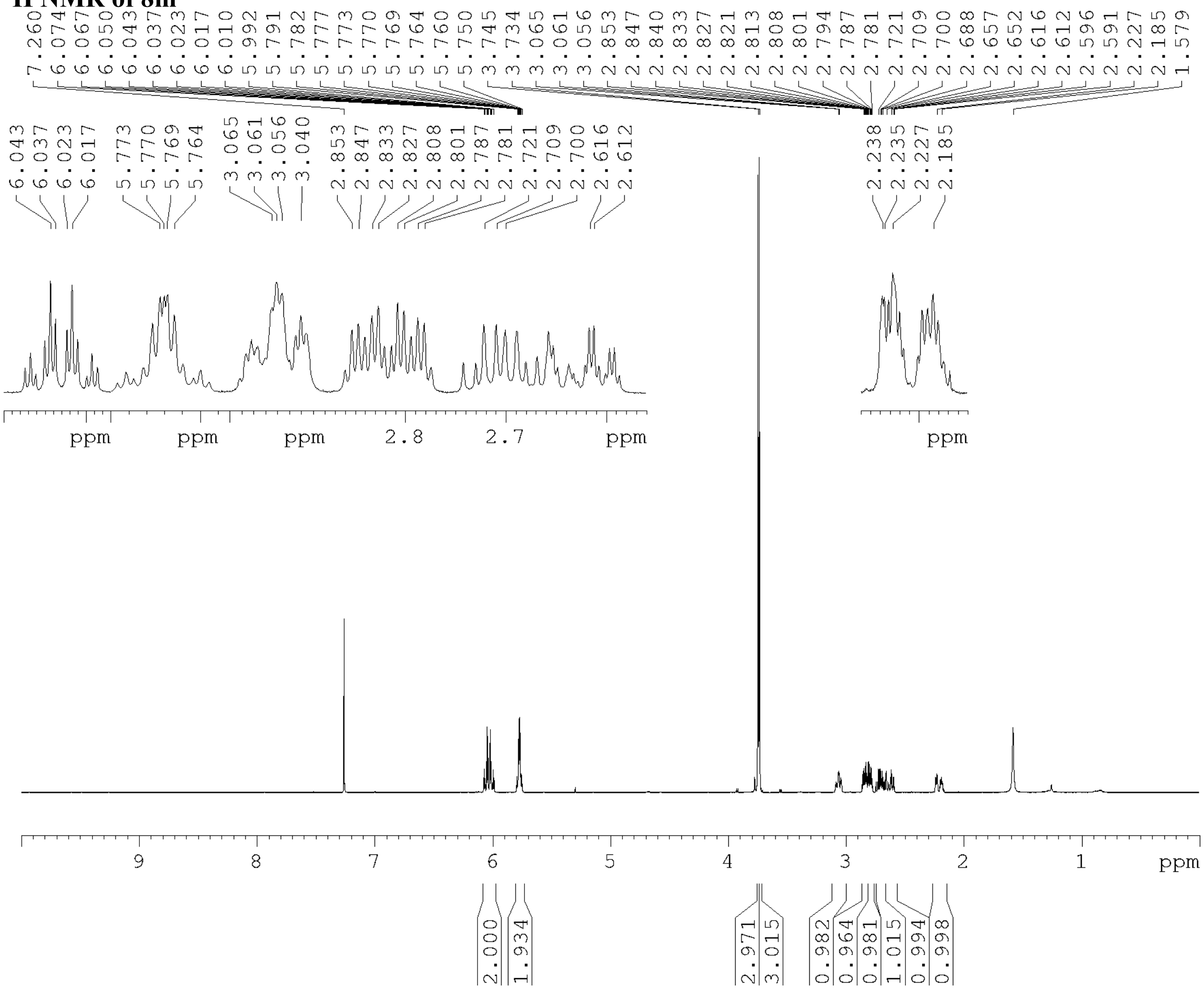


```
Current Data Parameters
NAME     20240503 lu068-1
EXPNO                  16
PROCNO                  1

F2 - Acquisition Parameters
Date_            20240503
Time                14.45
INSTRUM             spect
PROBHD   5 mm BBO BB-1H
PULPROG              zg30
TD                  32768
SOLVENT             CDCl3
NS                     16
DS                      0
SWH           6009.615 Hz
FIDRES        0.183399 Hz
AQ           2.7262976 sec
RG                    256
DW             83.200 usec
DE               6.50 usec
TE              298.6 K
D1         1.50000000 sec
TD0                     1

======== CHANNEL f1 ========
NUC1                   1H
P1             14.00 usec
PL1             -1.00 dB
PL1W       7.55784369 W
SFO1      400.1326010 MHz

F2 - Processing parameters
SI                  32768
SF        400.1300098 MHz
WDW                    EM
SSB                     0
LB                      0 Hz
GB                      0
PC                   1.00
```

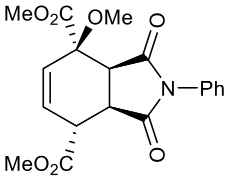

8l

$^{13}C\{1H\}$ and DEPT 90, 135 NMR of 8m

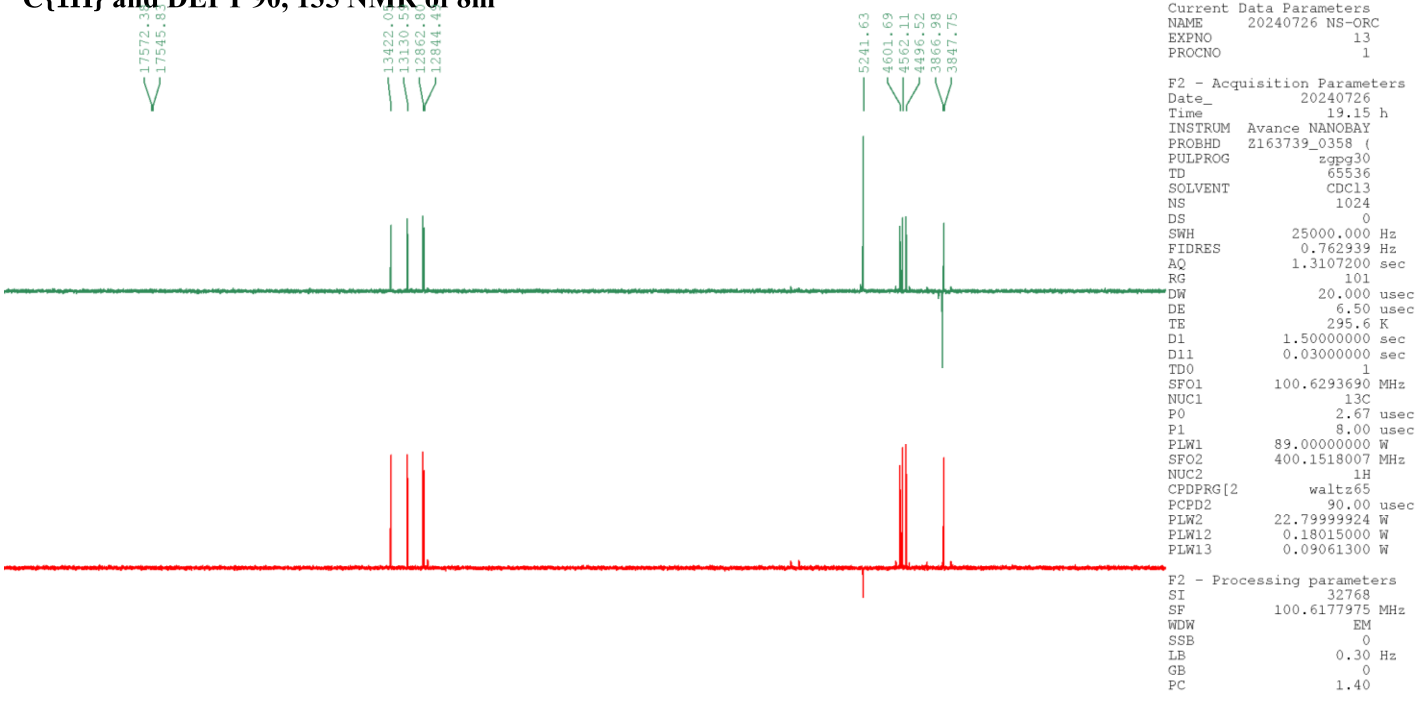

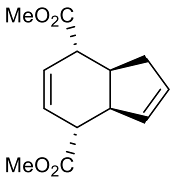

8m

^{1}H NMR of 8n

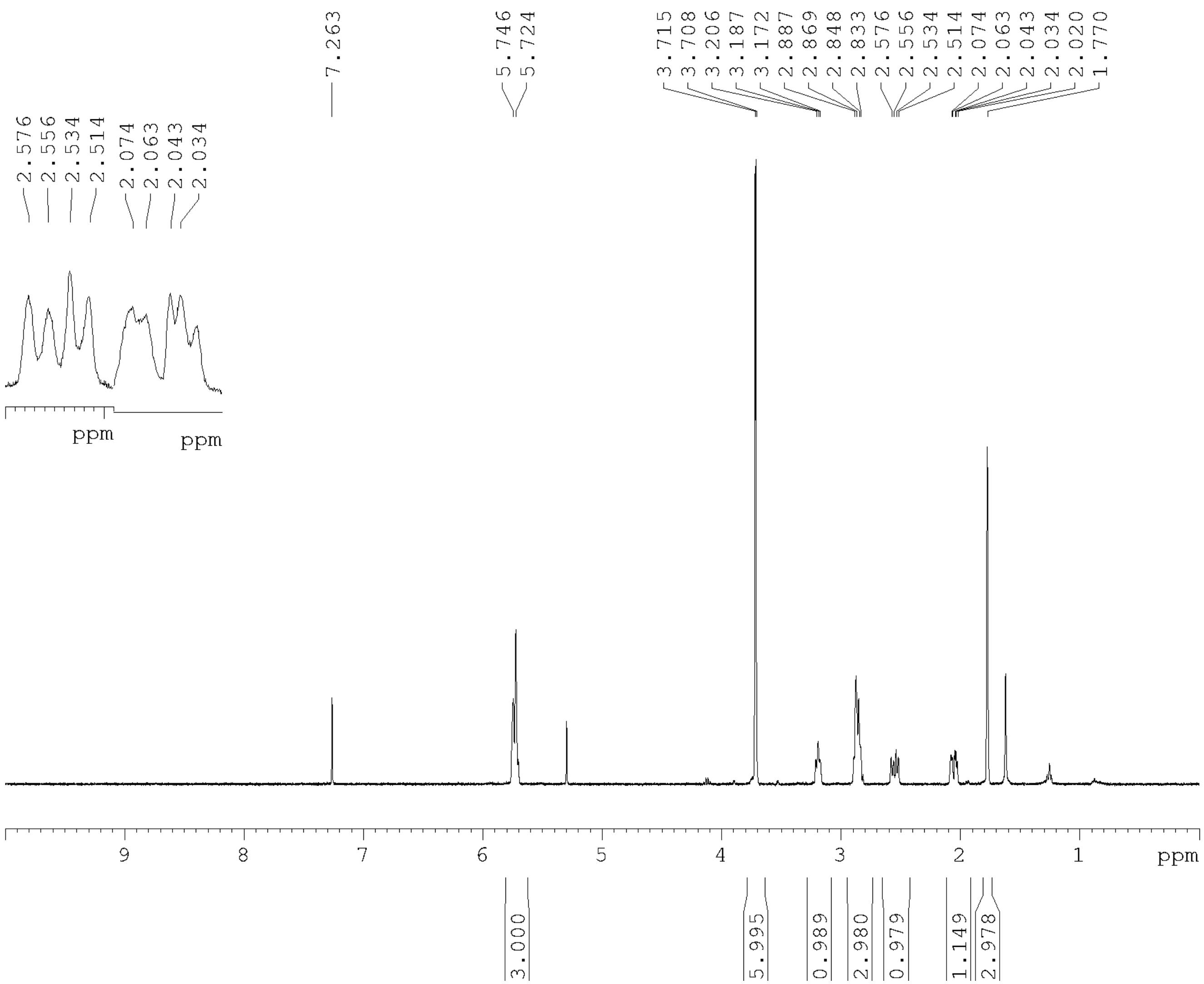


```
Current Data Parameters
NAME       20240401 SZ301-1
EXPNO                     1
PROCNO                    1

F2 - Acquisition Parameters
Date_            20240401
Time                12.22
INSTRUM             spect
PROBHD   5 mm BBO BB-1H
PULPROG              zg30
TD                  32768
SOLVENT             CDCl3
NS                      1
DS                      0
SWH              6009.615 Hz
FIDRES           0.183399 Hz
AQ              2.7262976 sec
RG                    203
DW                 83.200 usec
DE                   6.50 usec
TE                  294.8 K
D1             1.50000000 sec
TD0                     1

======== CHANNEL f1 ========
NUC1                   1H
P1                  14.00 usec
PL1                 -1.00 dB
PL1W           7.55784369 W
SFO1          400.1326010 MHz

F2 - Processing parameters
SI                  32768
SF            400.1300087 MHz
WDW                    EM
SSB                     0
LB                      0 Hz
GB                      0
PC                   1.00
```

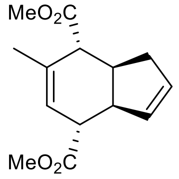

8n

^{13}C{1H} and DEPT 90, 135 NMR of 8n

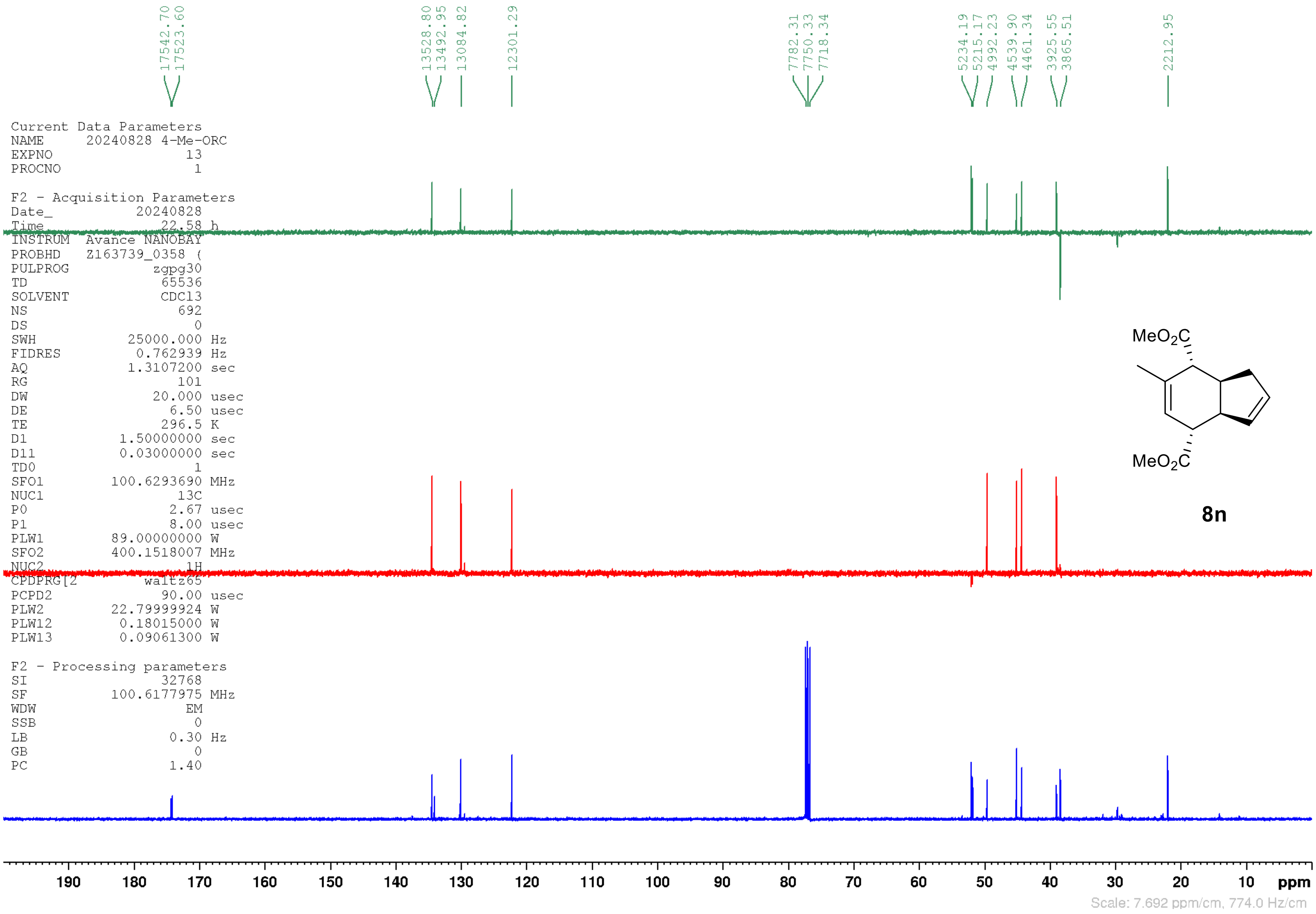

^{1}H NMR of 8o

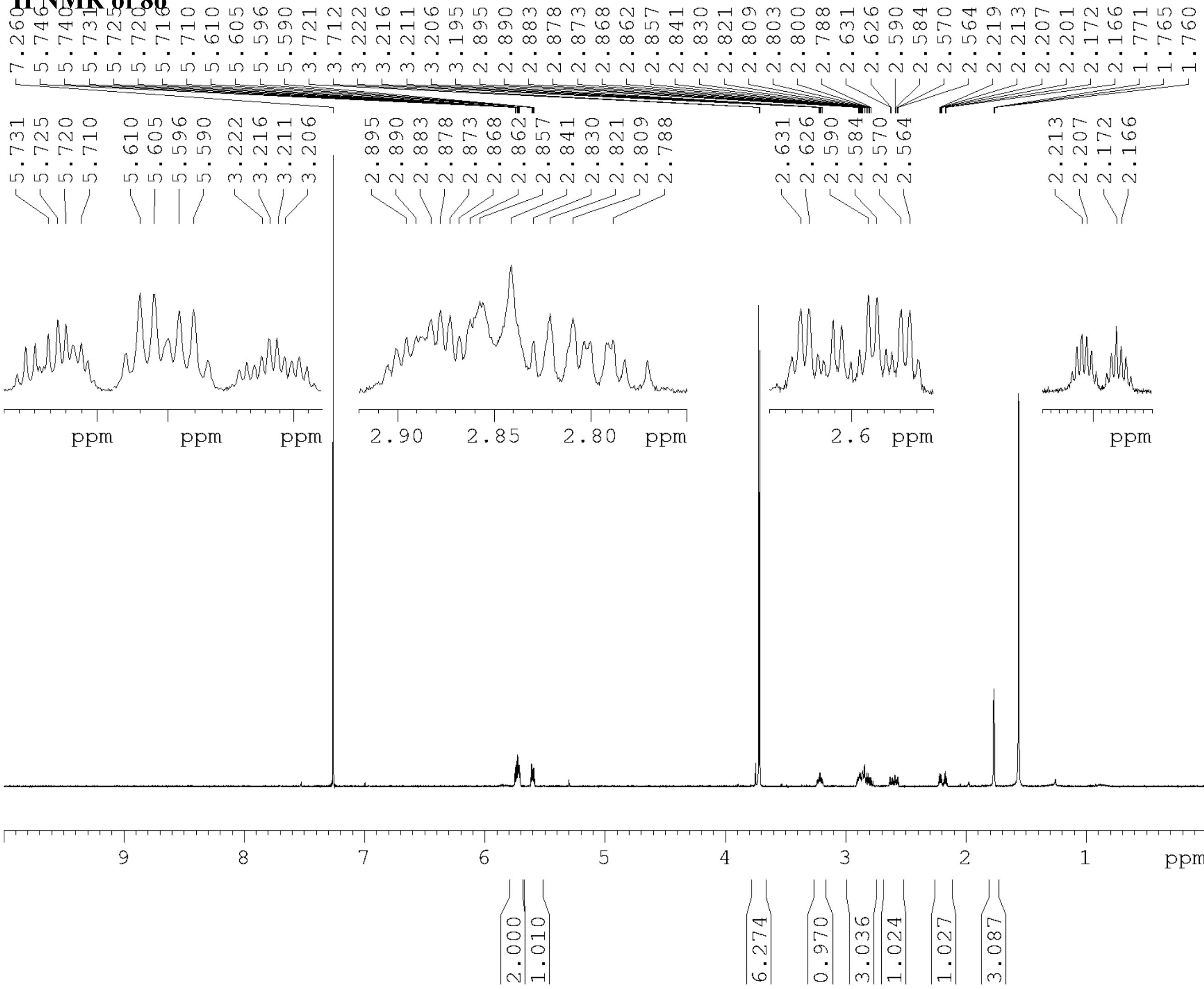

Current Data Parameters
NAME 20240430 lu067-1
EXPNO 1
PROCNO 1

F2 - Acquisition Parameters
Date_ 20240430
Time 18.59
INSTRUM spect
PROBHD 5 mm BBO BB-1H
PULPROG zg30
TD 32768
SOLVENT CDCl3
NS 16
DS 0
SWH 6009.615 Hz
FIDRES 0.183399 Hz
AQ 2.7262976 sec
RG 456
DW 83.200 usec
DE 6.50 usec
TE 295.6 K
D1 1.50000000 sec
TD0 1

======== CHANNEL f1 ========
NUC1 1H
P1 14.00 usec
PL1 -1.00 dB
PL1W 7.55784369 W
SFO1 400.1326010 MHz

F2 - Processing parameters
SI 32768
SF 400.1300099 MHz
WDW EM
SSB 0
LB 0 Hz
GB 0
PC 1.00

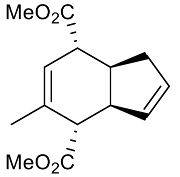

8o

^{13}C{1H} and DEPT 90, 135 NMR of 8o

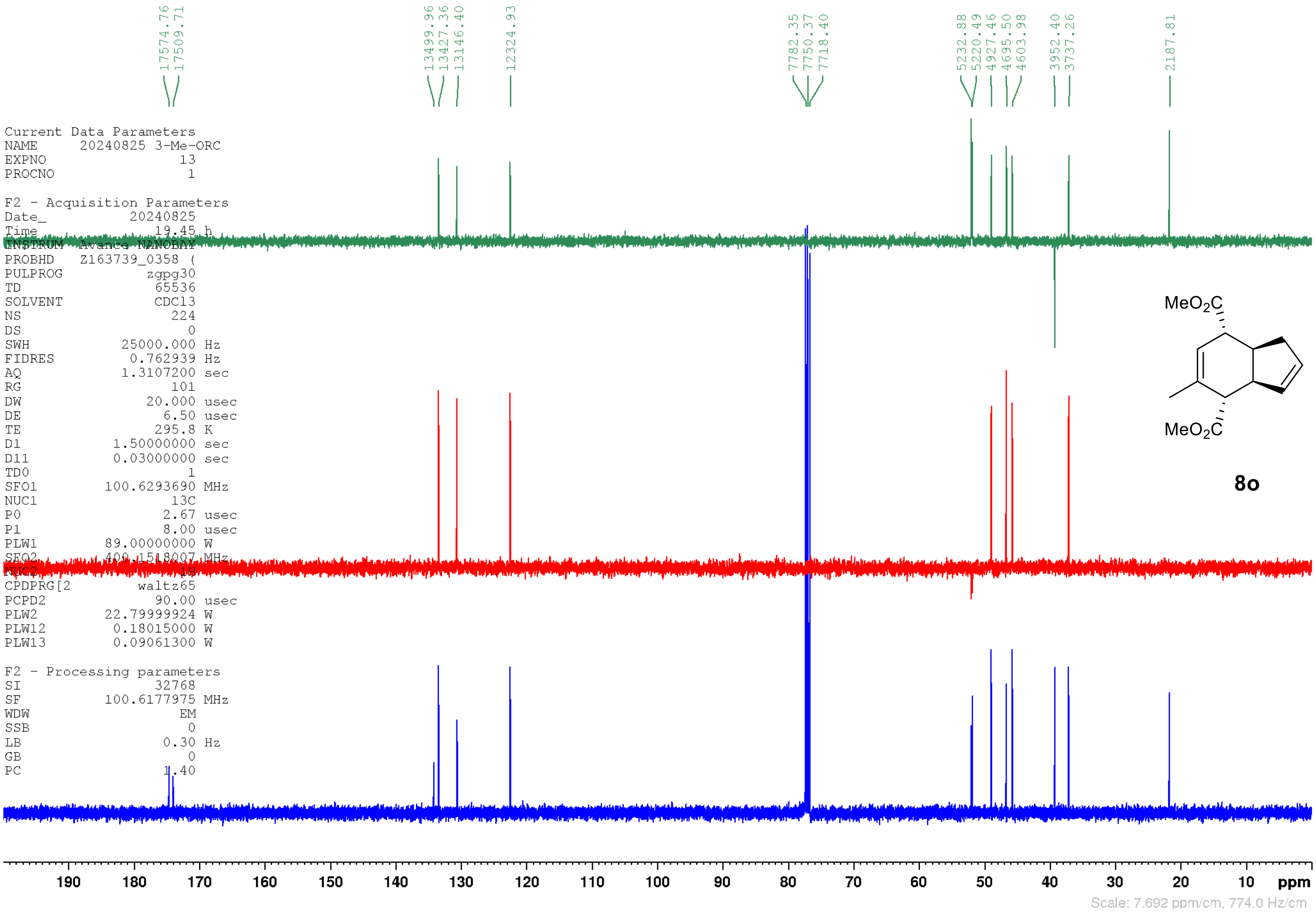

^{1}H NMR of 8p

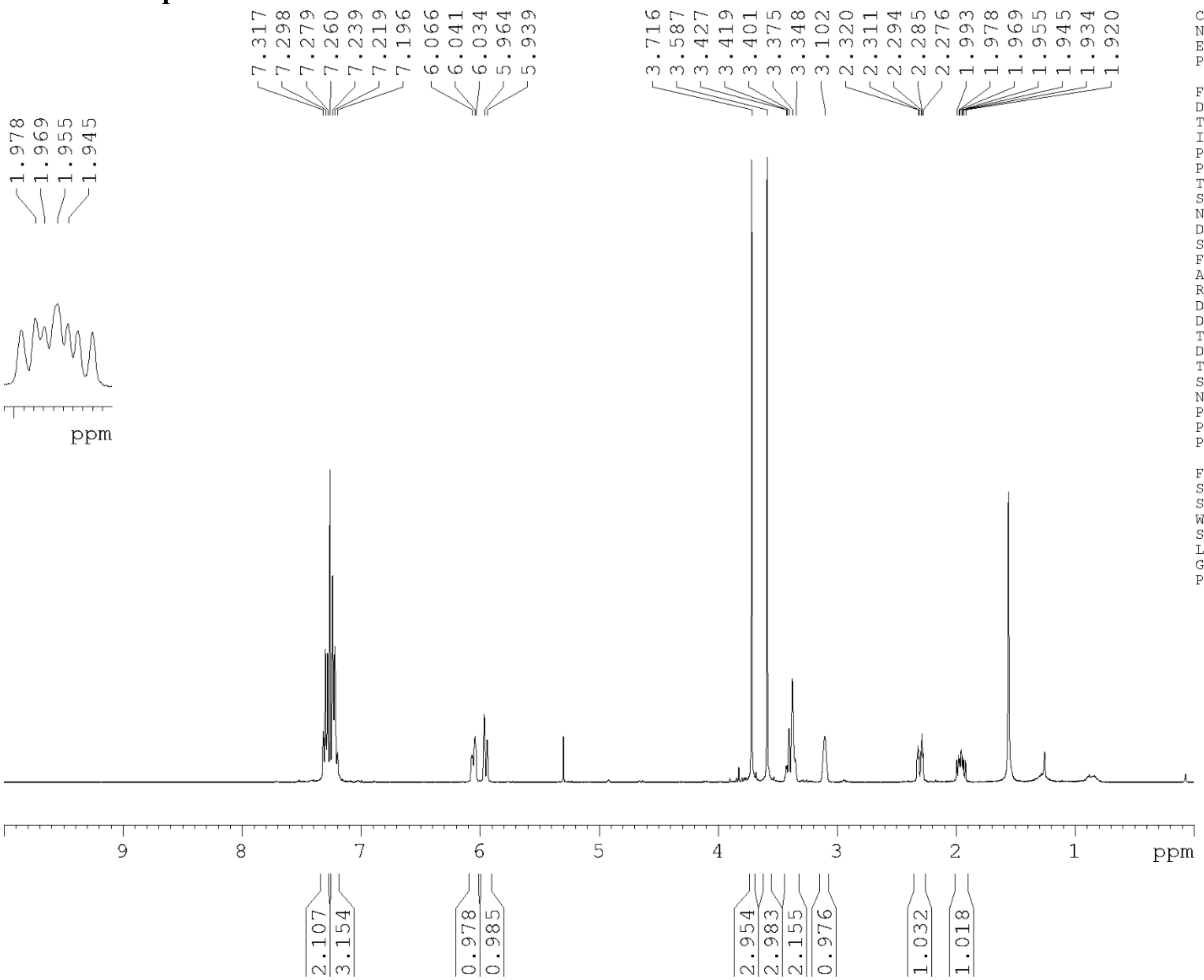

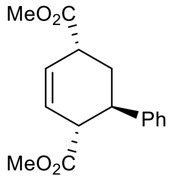

8p

$^{13}C\{1H\}$ and DEPT 90, 135 NMR of 8p

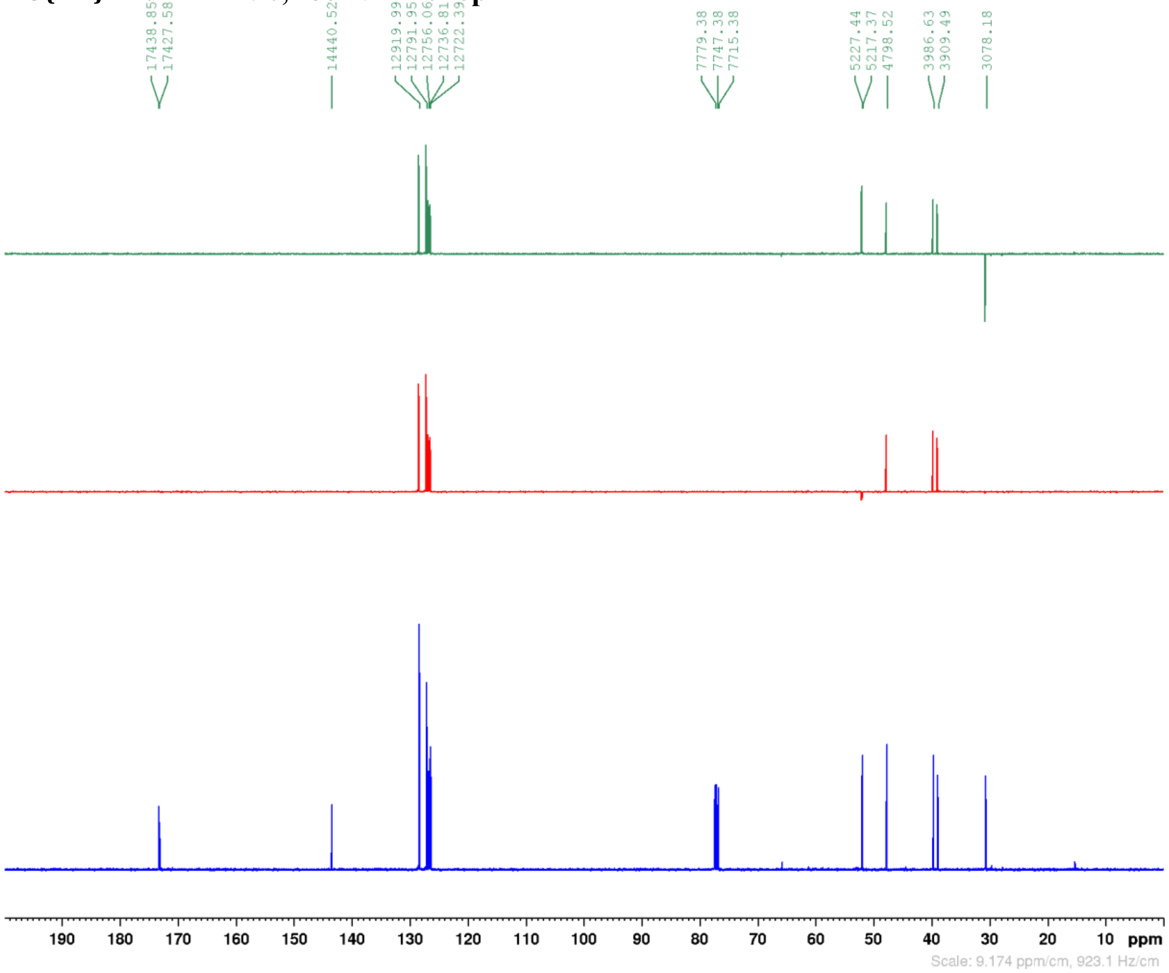

Current Data Parameters

NAME	20240714 NS-ORS
EXPNO	13
PROCNO	1

F2 - Acquisition Parameters

Date_	20240714
Time	21.05 h
INSTRUM	Avance NANOBAY
PROBHD	Z163739_0358 (
PULPROG	zgpg30
TD	65536
SOLVENT	CDCl3
NS	301
DS	0
SWH	25000.000 Hz
FIDRES	0.762939 Hz
AQ	1.3107200 sec
RG	101
DW	20.000 usec
DE	6.50 usec
TE	295.5 K
D1	1.50000000 sec
D11	0.03000000 sec
TD0	1
SFO1	100.6293690 MHz
NUC1	13C
P0	2.67 usec
P1	8.00 usec
PLW1	89.00000000 W
SFO2	400.1518007 MHz
NUC2	1H
CPDPRG[2	waltz65
PCPD2	90.00 usec
PLW2	22.79999924 W
PLW12	0.18015000 W
PLW13	0.09061300 W

F2 - Processing parameters

SI	32768
SF	100.6177975 MHz
WDW	EM
SSB	0
LB	0.30 Hz
GB	0
PC	1.40

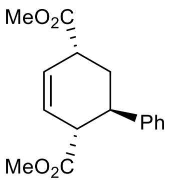

8p

^{1}H NMR of 8q

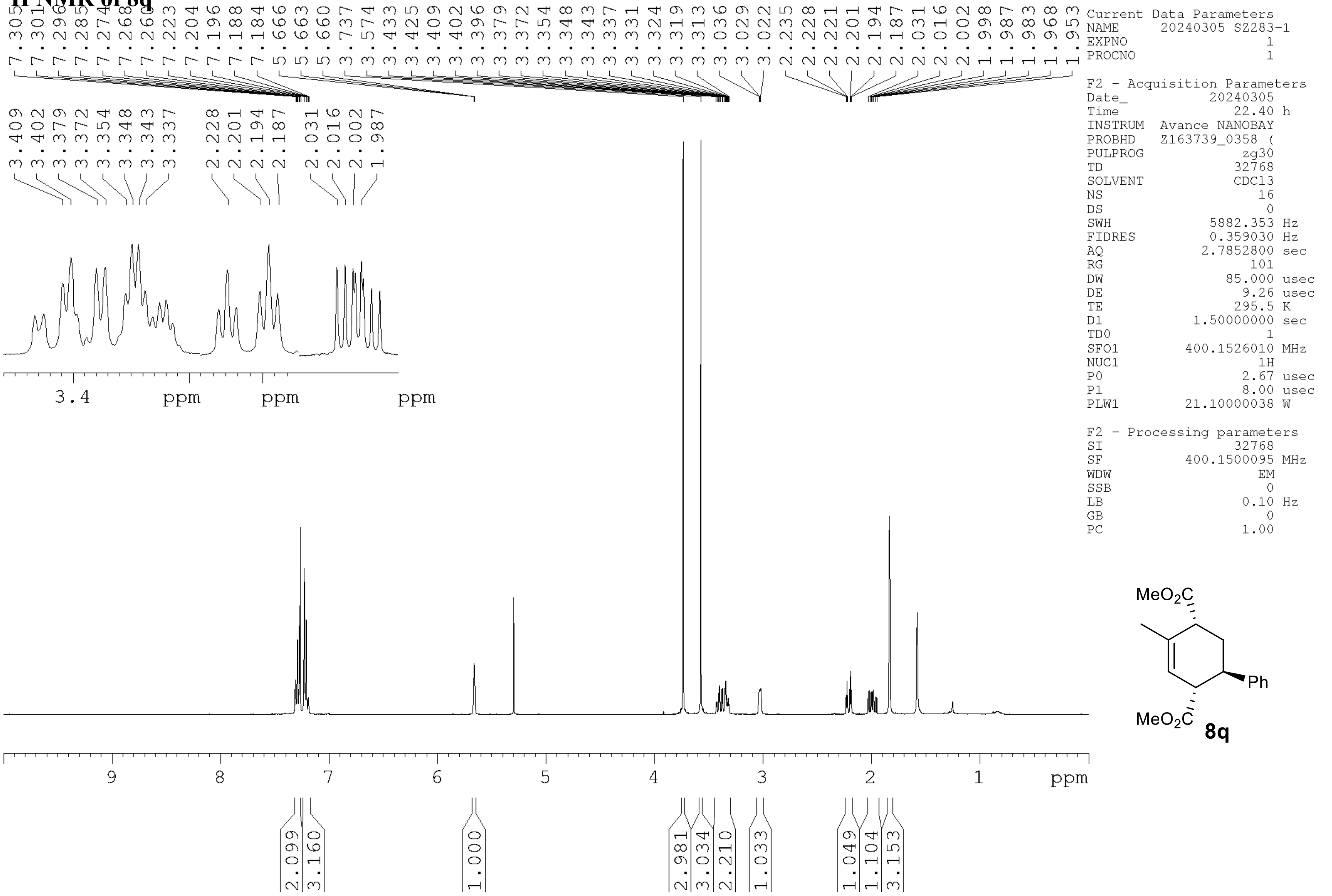
Current Data Parameters
NAME 20240305 SZ283-1
EXPNO 1
PROCNO 1
F2 - Acquisition Parameters
Date_ 20240305
Time 22.40 h
INSTRUM Avance NANOBAY
PROBHD Z163739_0358 (
PULPROG zg30
TD 32768
SOLVENT CDCl3
NS 16
DS 0
SWH 5882.353 Hz
FIDRES 0.359030 Hz
AQ 2.7852800 sec
RG 101
DW 85.000 usec
DE 9.26 usec
TE 295.5 K
D1 1.50000000 sec
TD0 1
SFO1 400.1526010 MHz
NUC1 1H
P0 2.67 usec
P1 8.00 usec
PLW1 21.10000038 W
F2 - Processing parameters
SI 32768
SF 400.1500095 MHz
WDW EM
SSB 0
LB 0.10 Hz
GB 0
PC 1.00
MeO2C
Ph
MeO2C
8q
ppm

$^{13}C\{^1H\}$ and DEPT 90, 135 NMR of 8q

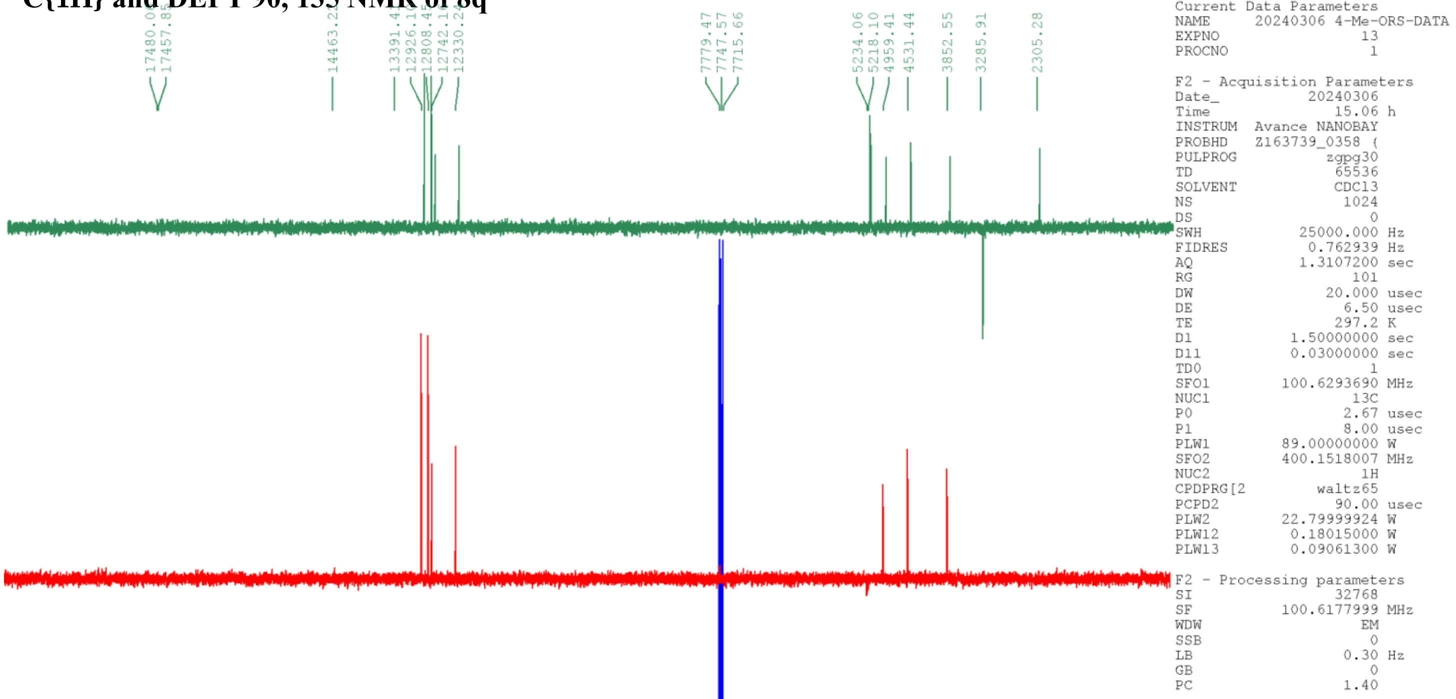

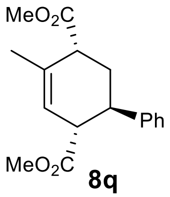

8q

^{1}H NMR of 8r

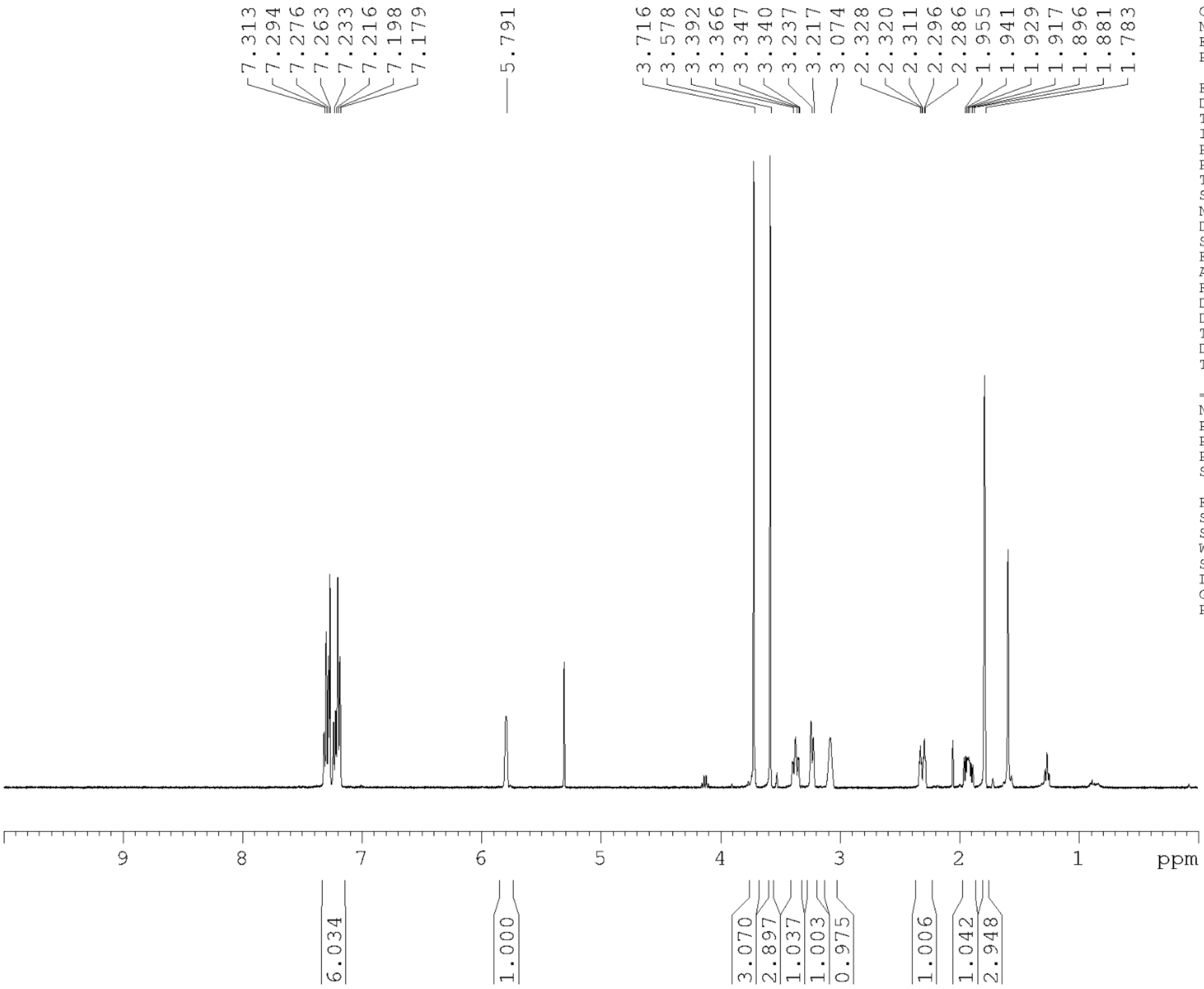


```
Current Data Parameters
NAME     20240320 SZ293-1
EXPNO                   1
PROCNO                  1

F2 - Acquisition Parameters
Date_            20240320
Time                21.37
INSTRUM             spect
PROBHD   5 mm BBO BB-1H
PULPROG              zg30
TD                  32768
SOLVENT             CDCl3
NS                     16
DS                      0
SWH              6009.615 Hz
FIDRES           0.183399 Hz
AQ              2.7262976 sec
RG                    287
DW                 83.200 usec
DE                   6.50 usec
TE                  293.6 K
D1             1.50000000 sec
TD0                     1

======== CHANNEL f1 ========
NUC1                   1H
P1                  14.00 usec
PL1                 -1.00 dB
PL1W           7.55784369 W
SFO1          400.1326010 MHz

F2 - Processing parameters
SI                  32768
SF            400.1300087 MHz
WDW                    EM
SSB                     0
LB                      0 Hz
GB                      0
PC                   1.00
```

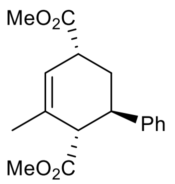

8r

^{13}C{1H} and DEPT 90, 135 NMR of 8r

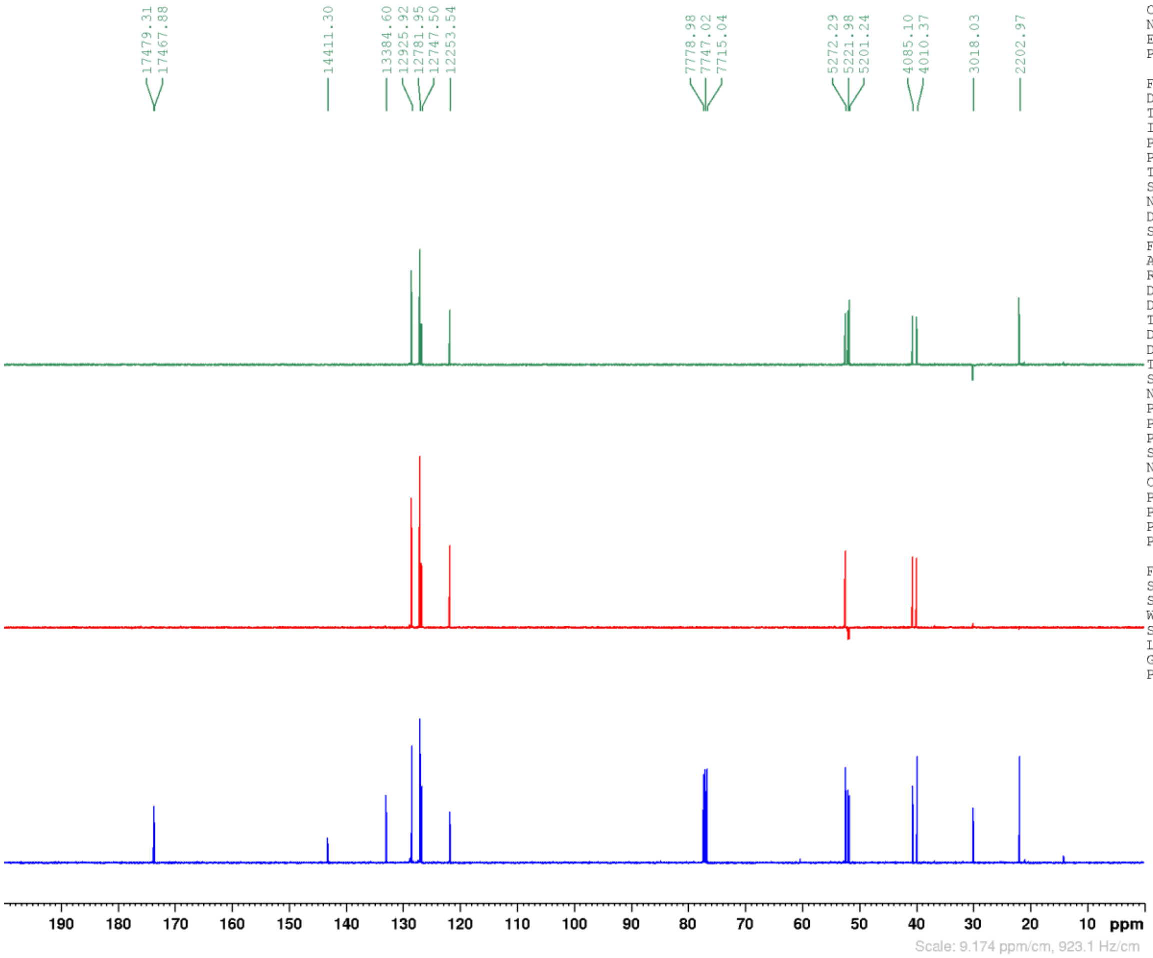


```
Current Data Parameters
NAME      20240815 3-Me-ORS
EXPNO                    13
PROCNO                    1

F2 - Acquisition Parameters
Date_              20240815
Time                  17.04 h
INSTRUM   Avance NANOBAY
PROBHD    Z163739_0358 (
PULPROG             zgpg30
TD                   65536
SOLVENT              CDCl3
NS                     699
DS                       0
SWH              25000.000 Hz
FIDRES            0.762939 Hz
AQ               1.3107200 sec
RG                     101
DW                  20.000 usec
DE                    6.50 usec
TE                   297.1 K
D1              1.50000000 sec
D11             0.03000000 sec
TD0                      1
SFO1           100.6293690 MHz
NUC1                   13C
P0                    2.67 usec
P1                    8.00 usec
PLW1           89.00000000 W
SFO2           400.1518007 MHz
NUC2                    1H
CPDPRG[2           waltz65
PCPD2                90.00 usec
PLW2           22.79999924 W
PLW12           0.18015000 W
PLW13           0.09061300 W

F2 - Processing parameters
SI                   32768
SF             100.6178044 MHz
WDW                     EM
SSB                      0
LB                    0.30 Hz
GB                       0
PC                    1.40
```

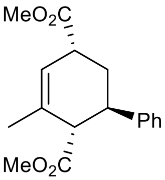

8r

^{1}H NMR of 9a

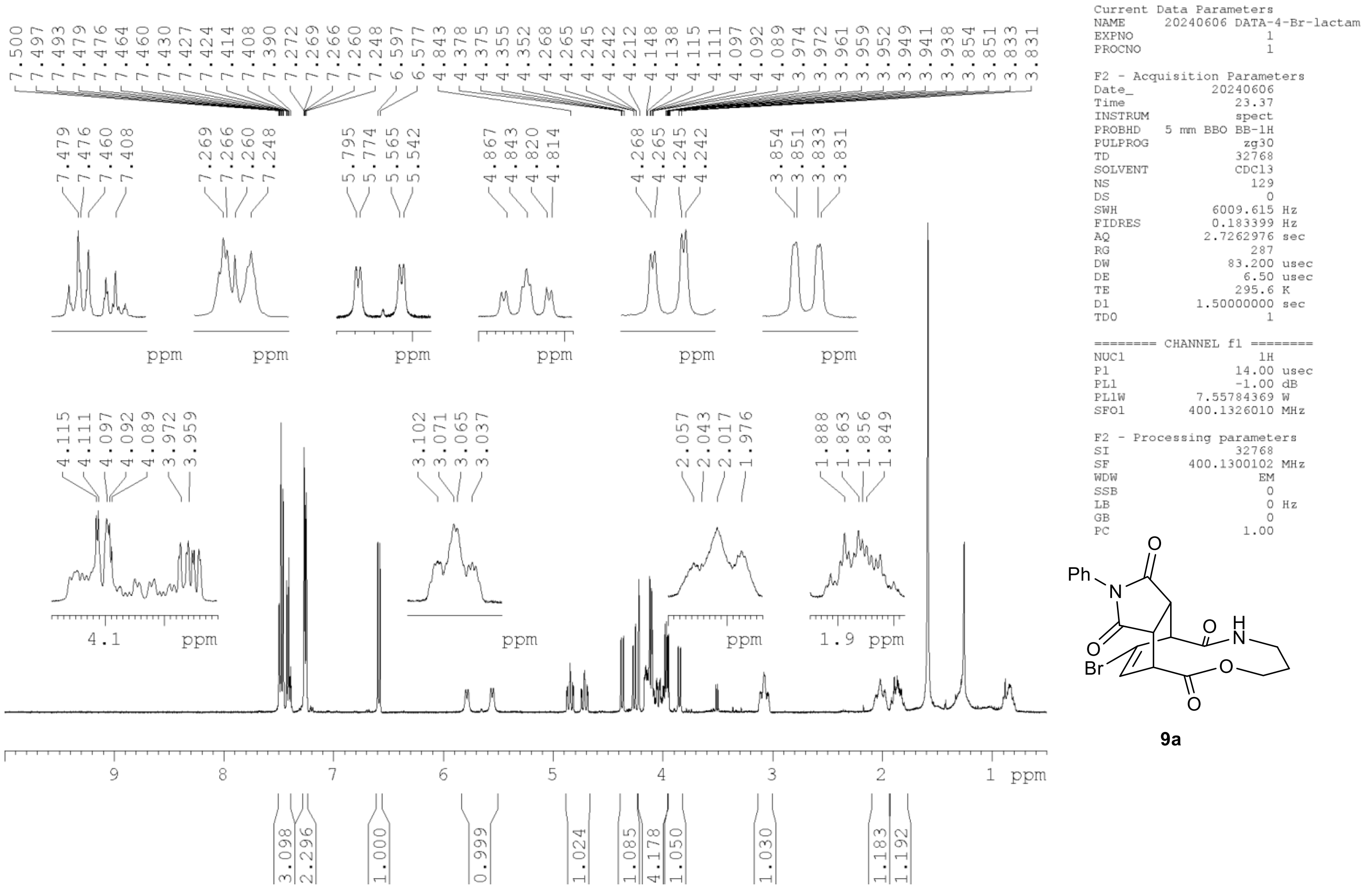

9a

^{13}C{1H} and DEPT 90, 135 NMR of 9a

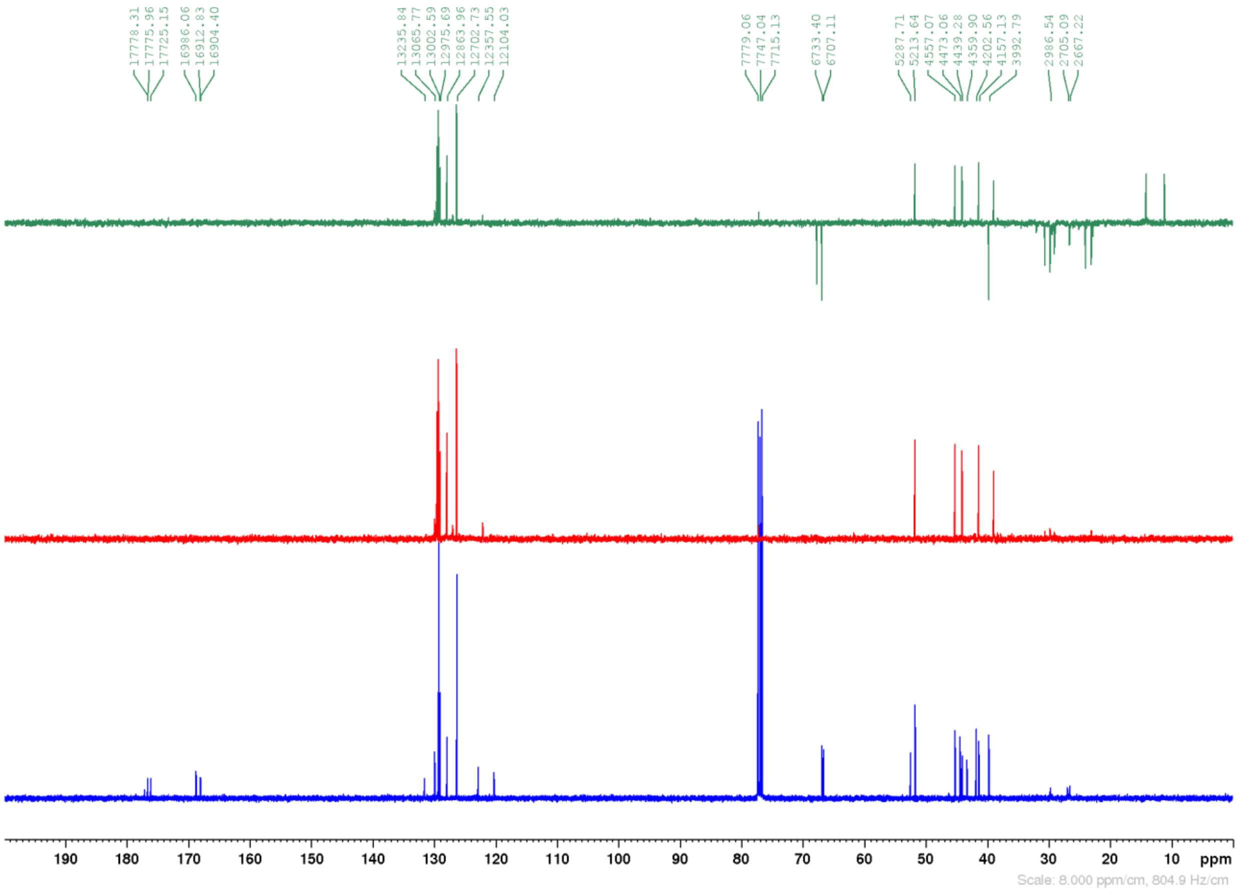


```
Current Data Parameters
NAME      20240603 DATA-4-Br-lactam
EXPNO                     13
PROCNO                     1

F2 - Acquisition Parameters
Date_               20240604
Time                    0.43
INSTRUM                spect
PROBHD    5 mm BBO BB-1H
PULPROG                 zgpg
TD                     65536
SOLVENT                CDCl3
NS                     12807
DS                         0
SWH                25252.525 Hz
FIDRES              0.385323 Hz
AQ                 1.2976128 sec
RG                       181
DW                    19.800 usec
DE                      6.50 usec
TE                     295.9 K
D1                1.50000000 sec
D11               0.03000000 sec
TD0                        1

======== CHANNEL f1 ========
NUC1                     13C
P1                     12.40 usec
PL1                        0 dB
PL1W             31.64976883 W
SFO1             100.6243400 MHz

======== CHANNEL f2 ========
CPDPRG[2             waltz16
NUC2                      1H
PCPD2                  90.00 usec
PL2                    -1.00 dB
PL12                   15.16 dB
PL13                   17.40 dB
PL2W              7.55784369 W
PL12W             0.18297760 W
PL13W             0.10924409 W
SFO2             400.1320007 MHz

F2 - Processing parameters
SI                     32768
SF               100.6127712 MHz
WDW                       EM
SSB                        0
LB                         0 Hz
GB                         0
PC                      1.00
```

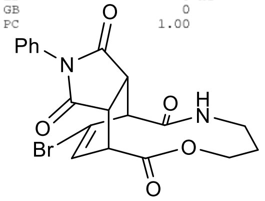

9a

^{1}H NMR of 9b + 9b'

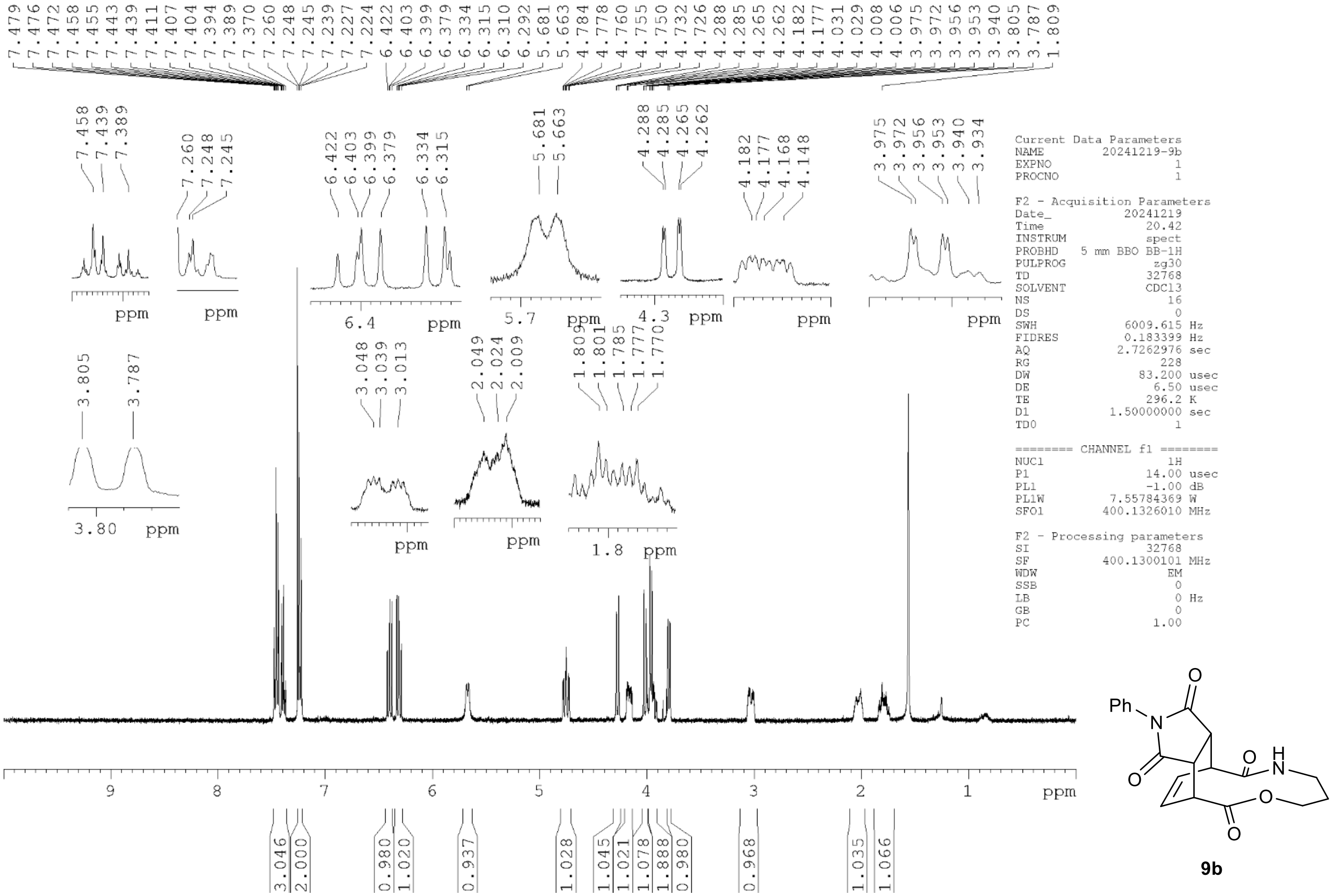

^{13}C{1H} and DEPT 90, 135 NMR of 9b + 9b'

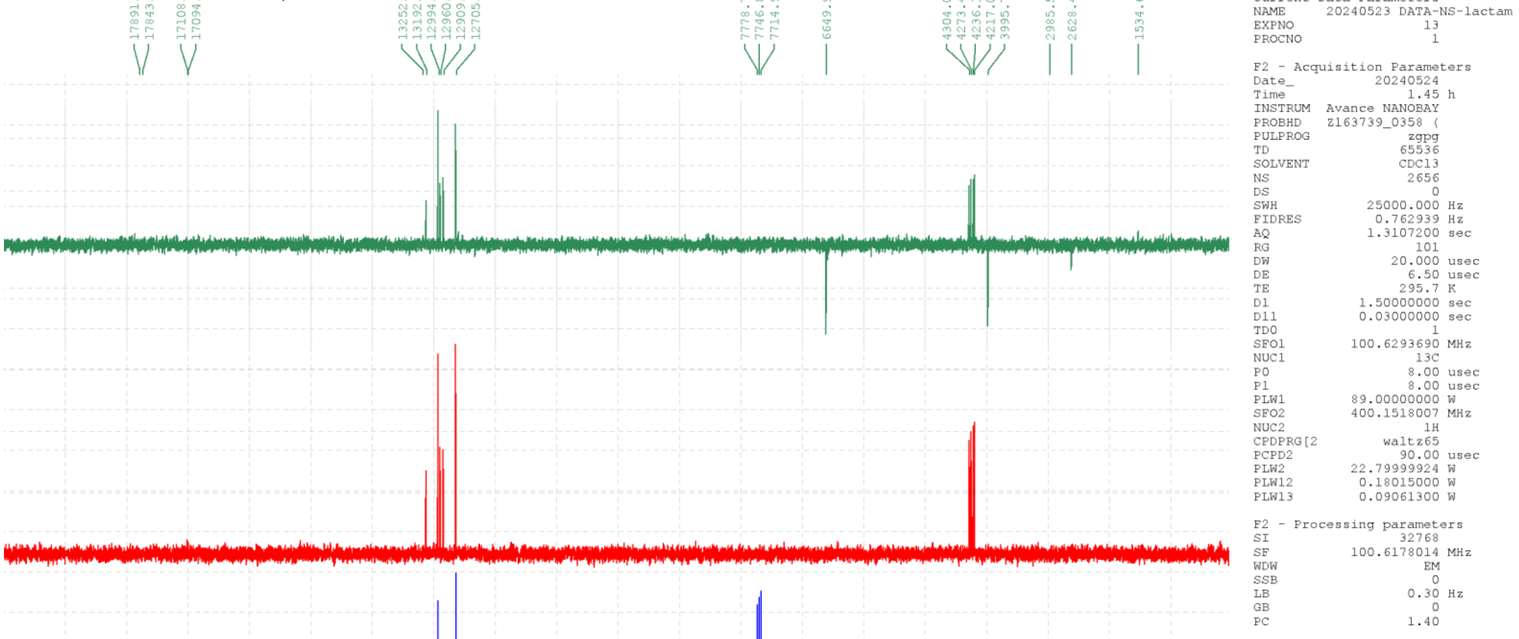
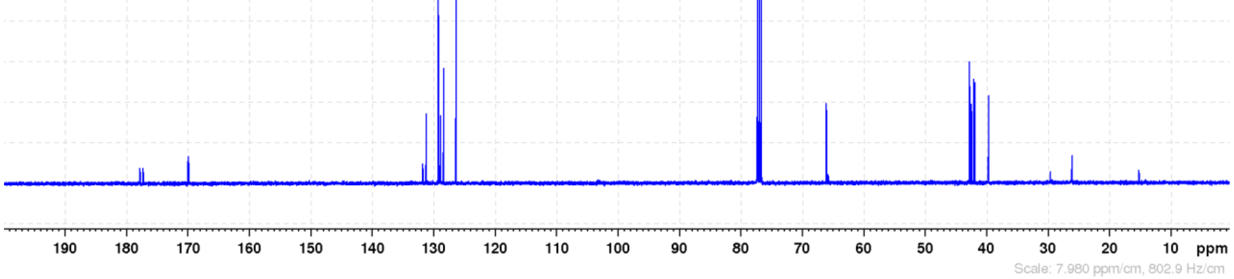

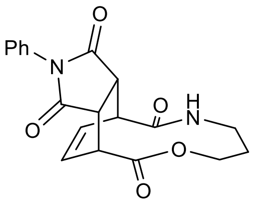

9b

1H NMR of 9c+9c’

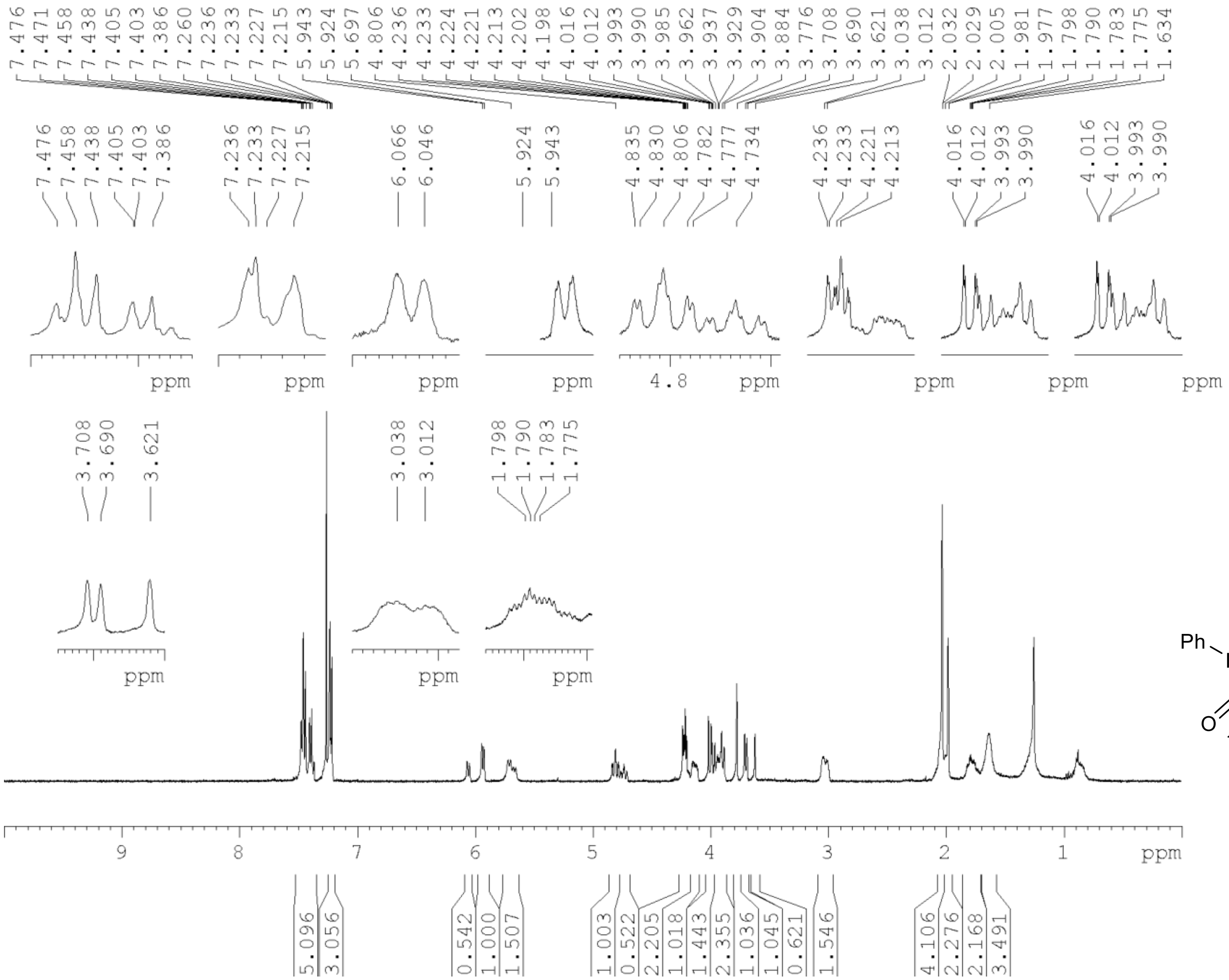

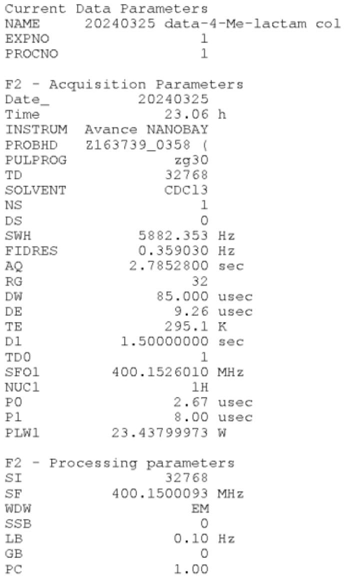

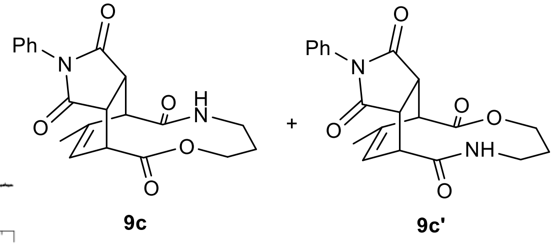

^{13}C{1H} and DEPT 90, 135 NMR of 9c+9c'

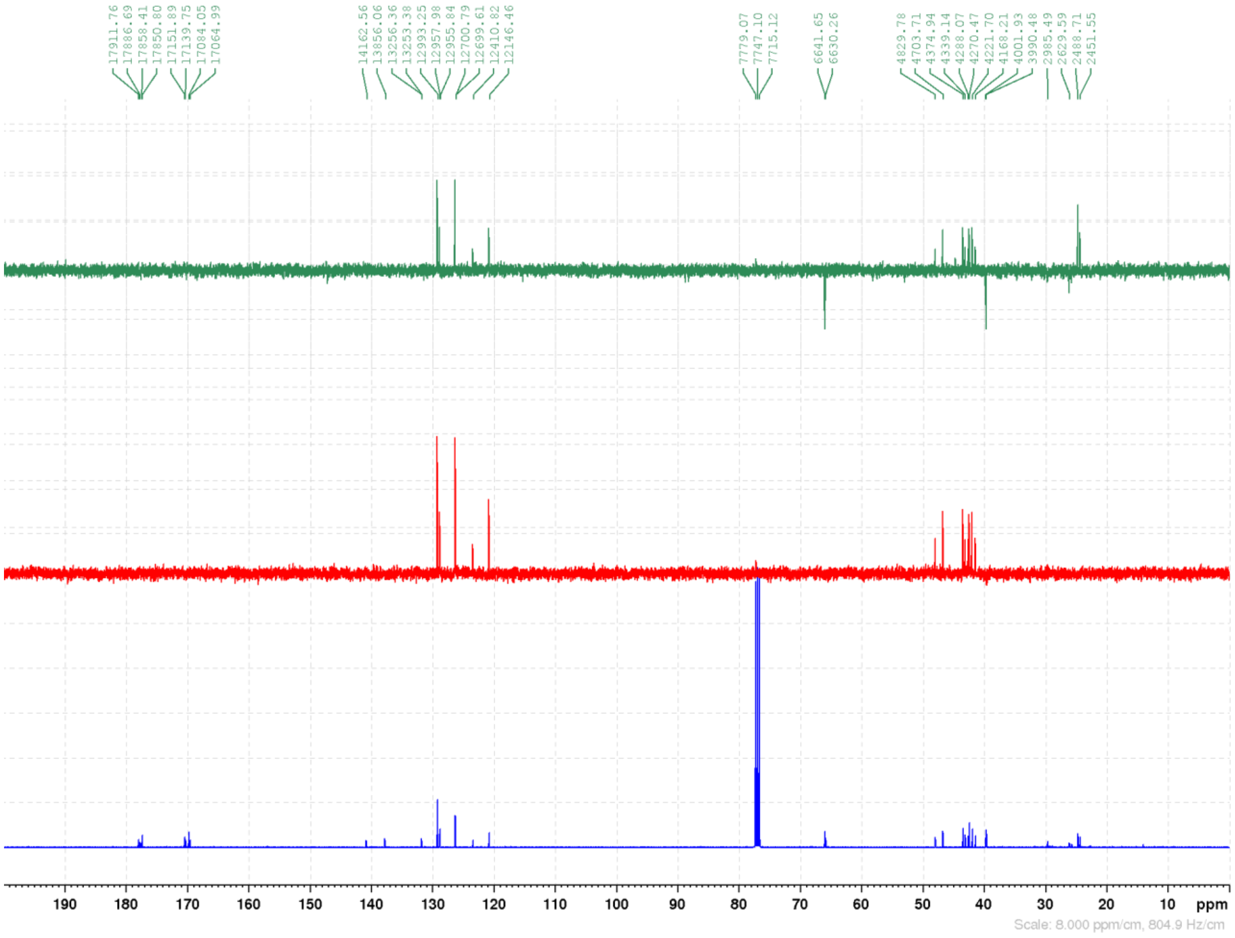

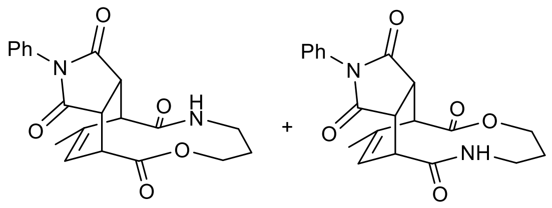

9c + **9c'**

^{1}H NMR of 9e + 9e'

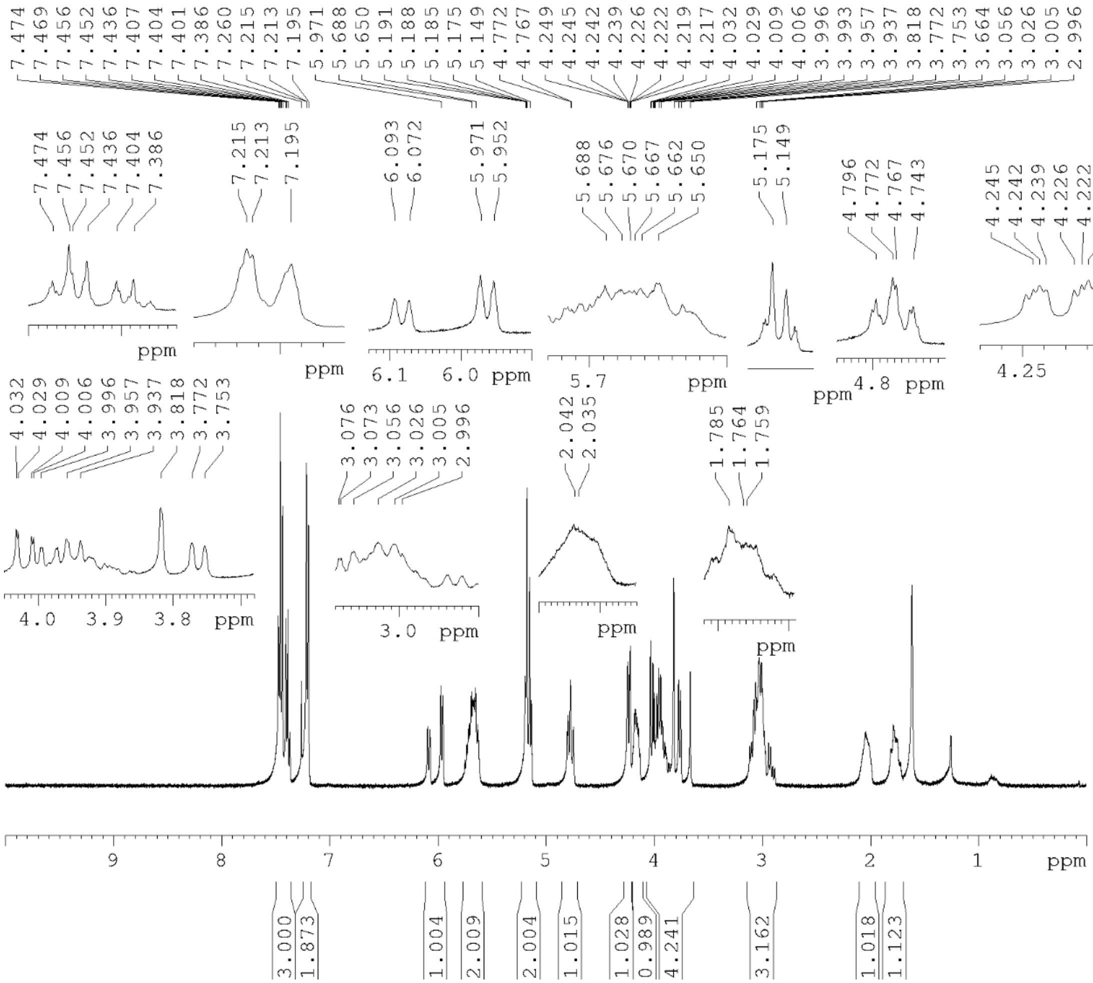

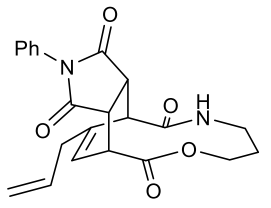

9e

^{13}C{1H} and DEPT 90, 135 NMR of 9e + 9e’

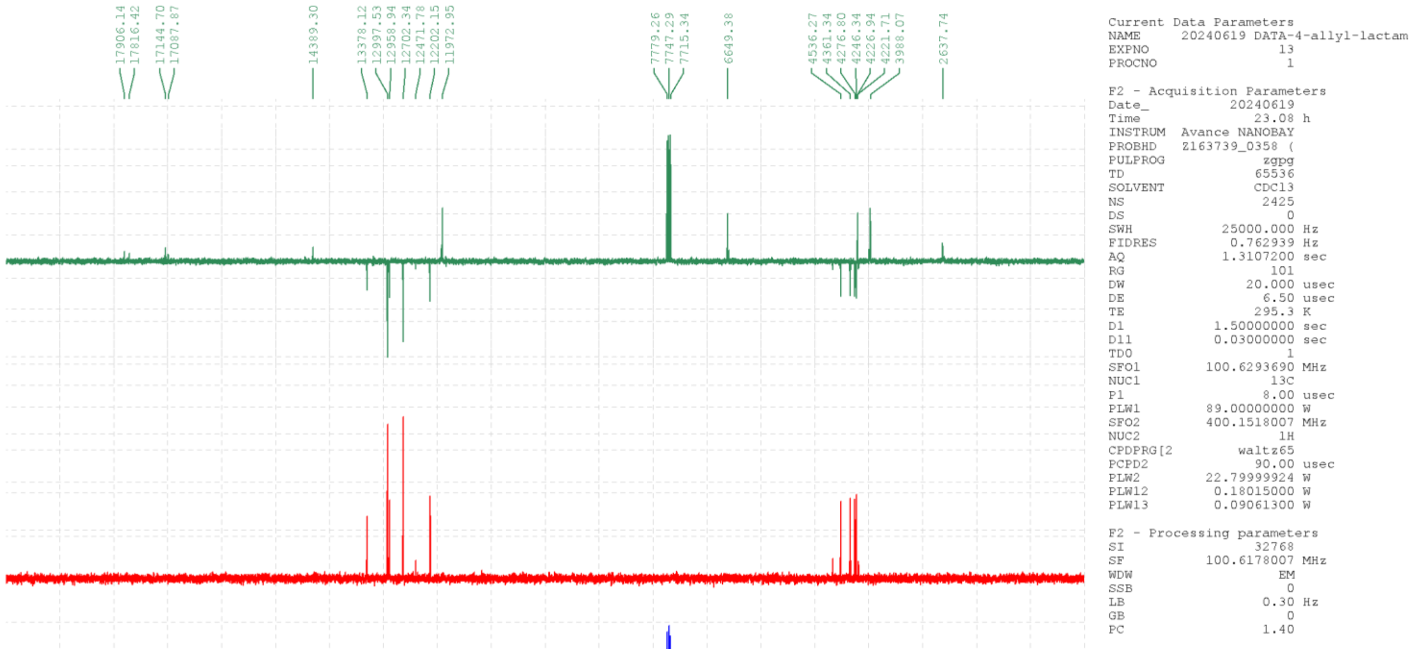

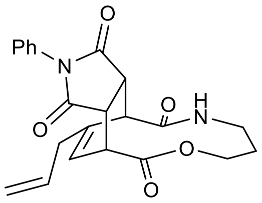

9e

^{1}H NMR of 9s + 9s'

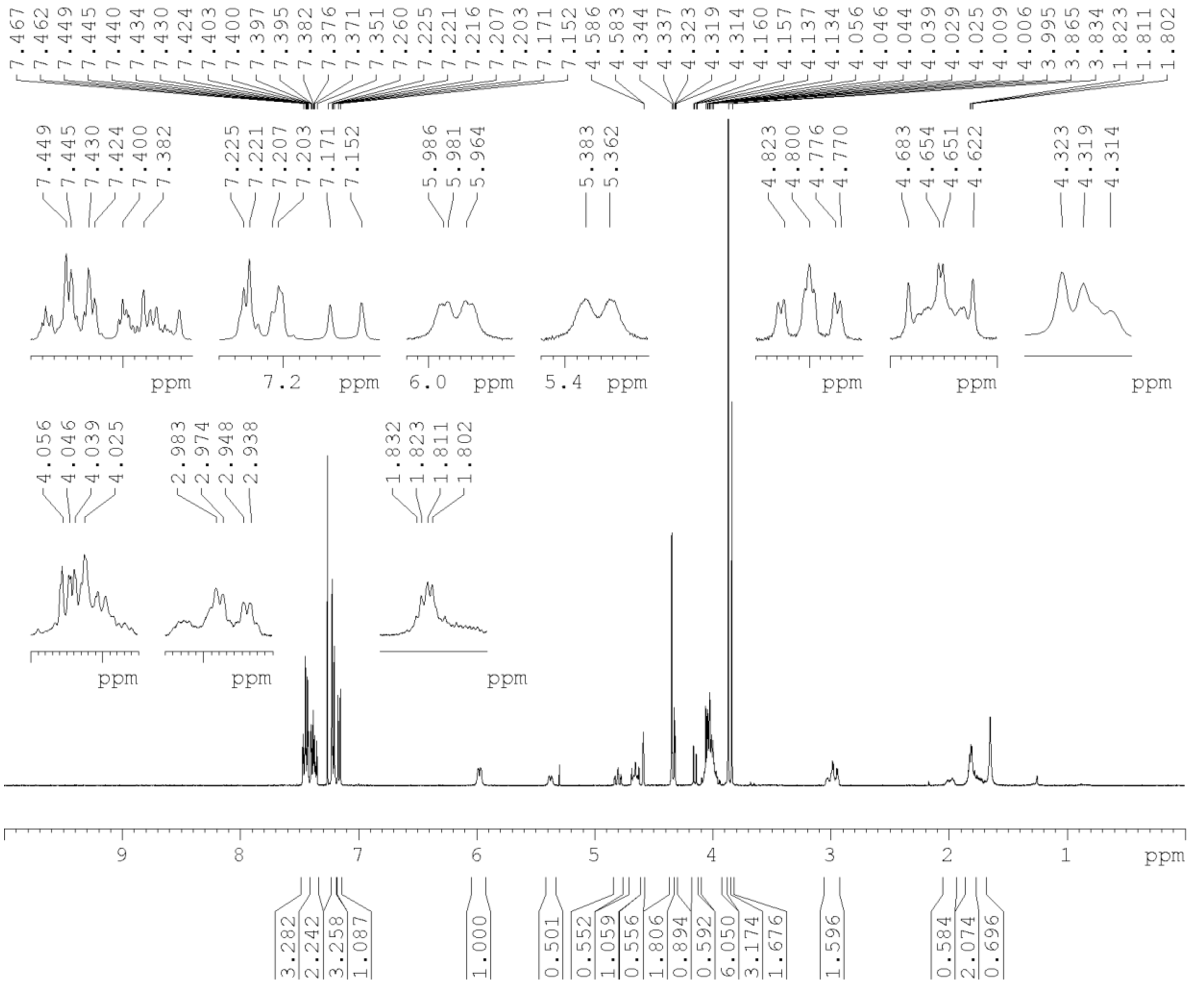

Current Data Parameters
NAME 20240429 data-4-CO2Me-lactam
EXPNO 1
PROCNO 1

F2 - Acquisition Parameters
Date_ 20240429
Time 23.11 h
INSTRUM Avance NANOBAY
PROBHD Z163739_0358 (
PULPROG zg30
TD 32768
SOLVENT CDCl3
NS 1
DS 0
SWH 5882.353 Hz
FIDRES 0.359030 Hz
AQ 2.7852800 sec
RG 101
DW 85.000 usec
DE 9.26 usec
TE 295.8 K
D1 1.50000000 sec
TD0 1
SFO1 400.1526010 MHz
NUC1 1H
P0 2.67 usec
P1 8.00 usec
PLW1 21.10000038 W

F2 - Processing parameters
SI 32768
SF 400.1500097 MHz
WDW EM
SSB 0
LB 0.10 Hz
GB 0
PC 1.00

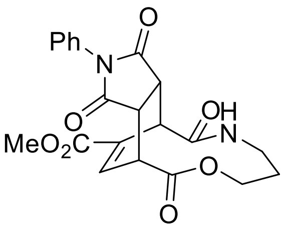

9s

^{13}C{1H} and DEPT 90, 135 NMR of 9s + 9s’

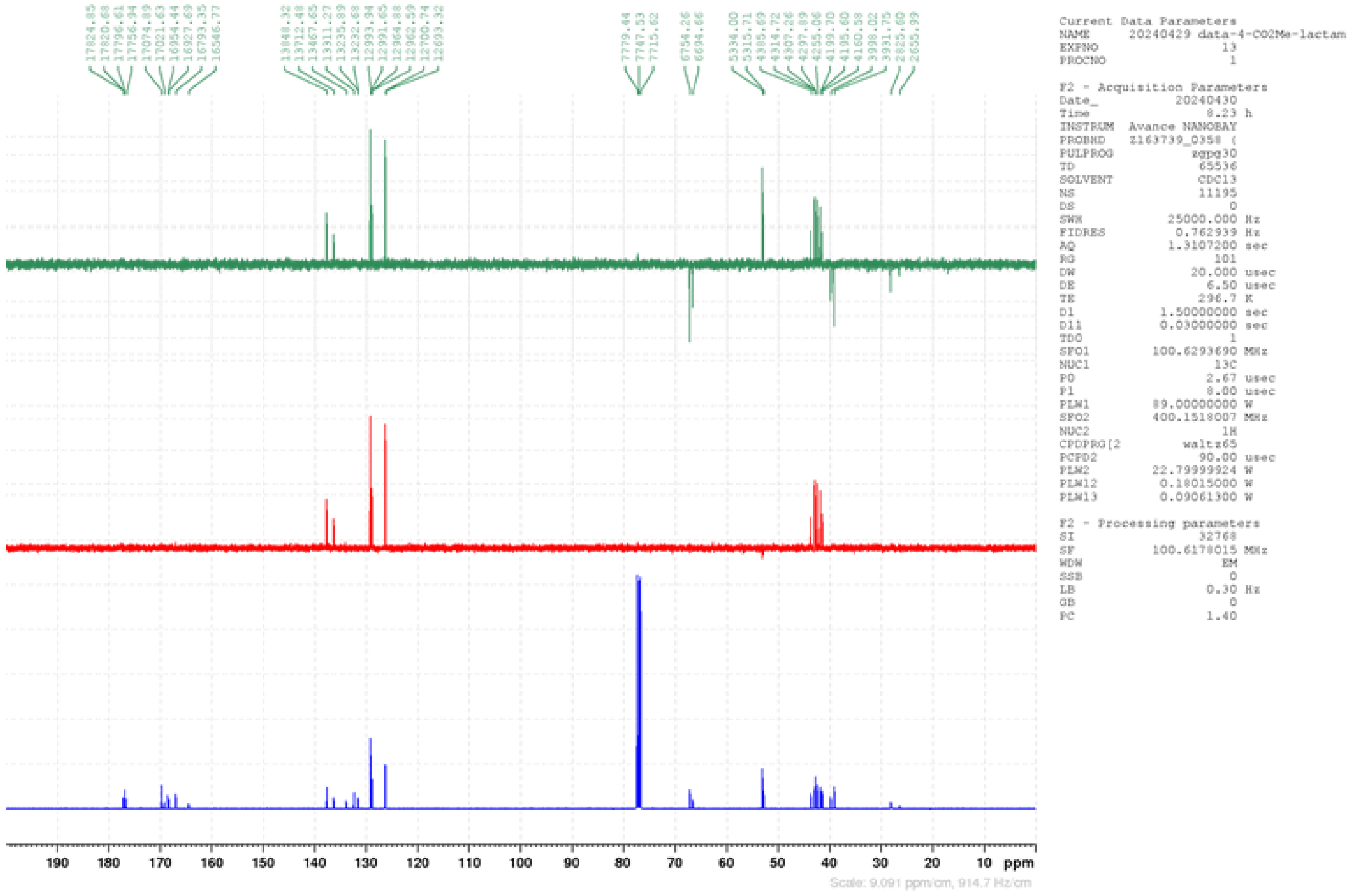

^{1}H NMR of 9g+9g’

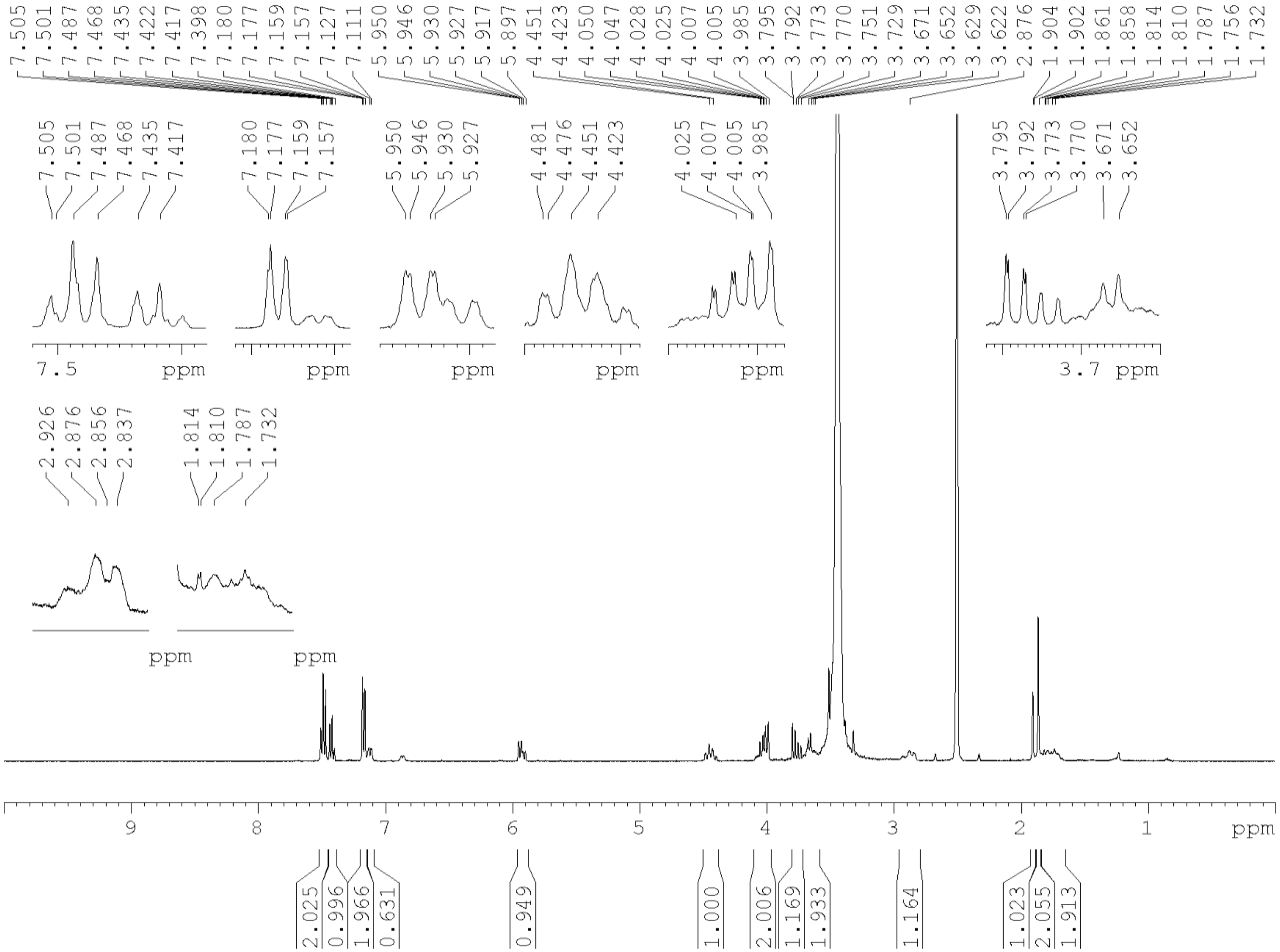

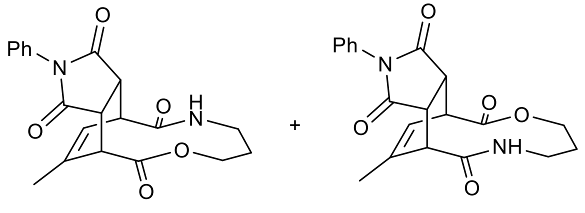

9g **9g'**

^{13}C{1H} and DEPT 90, 135 NMR of 9g+9g'

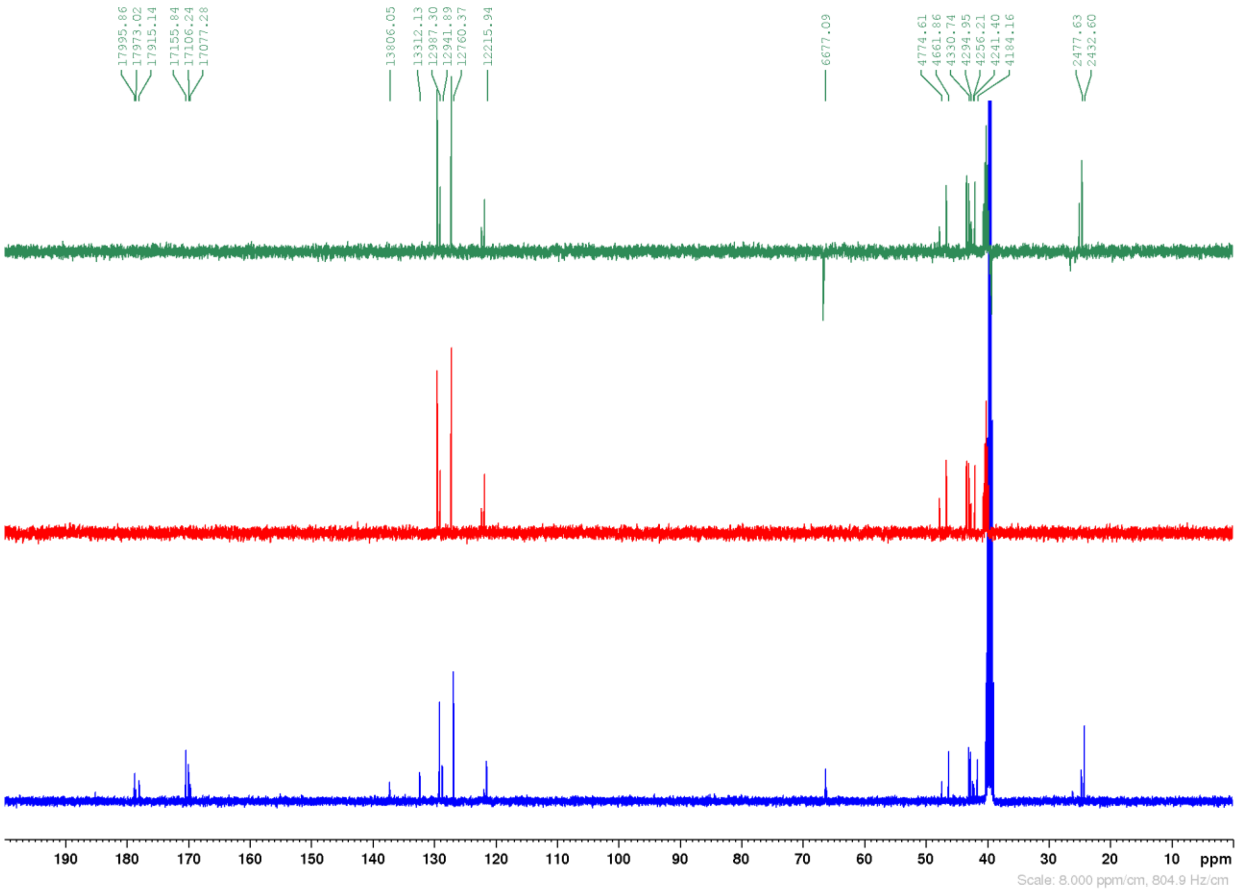


```
Current Data Parameters
NAME      3Me-LactaminDMSOd6-20240821_IPL
EXPNO                  13
PROCNO                  1

F2 - Acquisition Parameters
Date_            20240821
Time                17.46 h
INSTRUM   Avance NANOBAY
PROBHD    Z163739_0358 (
PULPROG              zgpg
TD                  65536
SOLVENT              DMSO
NS                   2048
DS                      8
SWH             25000.000 Hz
FIDRES           0.762939 Hz
AQ              1.3107200 sec
RG                    101
DW                 20.000 usec
DE                   6.50 usec
TE                  296.7 K
D1             1.50000000 sec
D11            0.03000000 sec
TD0                     1
SFO1          100.6293690 MHz
NUC1                  13C
P1                   8.00 usec
PLW1          89.00000000 W
SFO2          400.1518007 MHz
NUC2                   1H
CPDPRG[2          waltz65
PCPD2               90.00 usec
PLW2          22.79999924 W
PLW12          0.18015000 W
PLW13          0.09061300 W

F2 - Processing parameters
SI                  32768
SF            100.6178350 MHz
WDW                    EM
SSB                     0
LB                   0.30 Hz
GB                      0
PC                   1.40
```

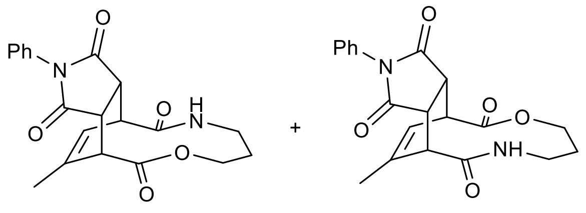

9g + **9g'**

^{1}H NMR of 9j+9j'

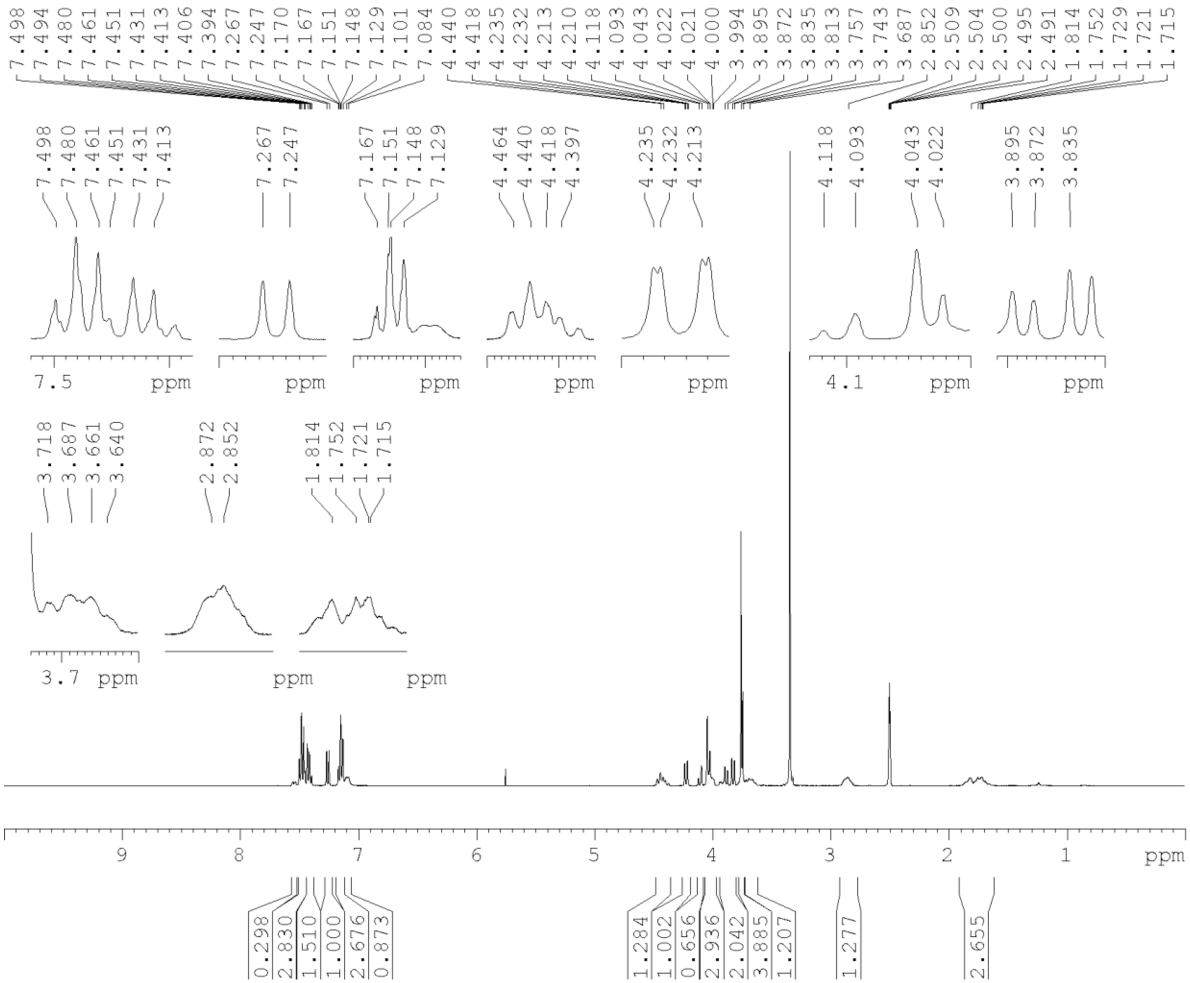

```
Current Data Parameters
NAME      3CO2Me-LactaminDMSOd6-20240820_IPL
EXPNO                    1
PROCNO                   1

F2 - Acquisition Parameters
Date_            20240820
Time                14.22 h
INSTRUM   Avance NANOBAY
PROBHD   Z163739_0358 (
PULPROG              zg30
TD                  32768
SOLVENT              DMSO
NS                      1
DS                      0
SWH              5882.353 Hz
FIDRES           0.359030 Hz
AQ              2.7852800 sec
RG                    101
DW                 85.000 usec
DE                   9.26 usec
TE                  296.6 K
D1             1.50000000 sec
TD0                     1
SFO1          400.1526010 MHz
NUC1                   1H
P0                   2.67 usec
P1                   8.00 usec
PLW1          21.10000038 W

F2 - Processing parameters
SI                  32768
SF            400.1500033 MHz
WDW                    EM
SSB                     0
LB                   0.10 Hz
GB                      0
PC                   1.00
```

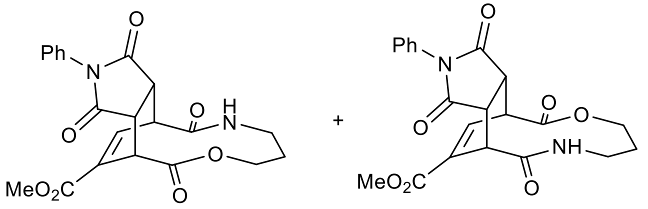

9j + **9j'**

^{13}C{1H} and DEPT 90, 135 NMR of 9j+9j’

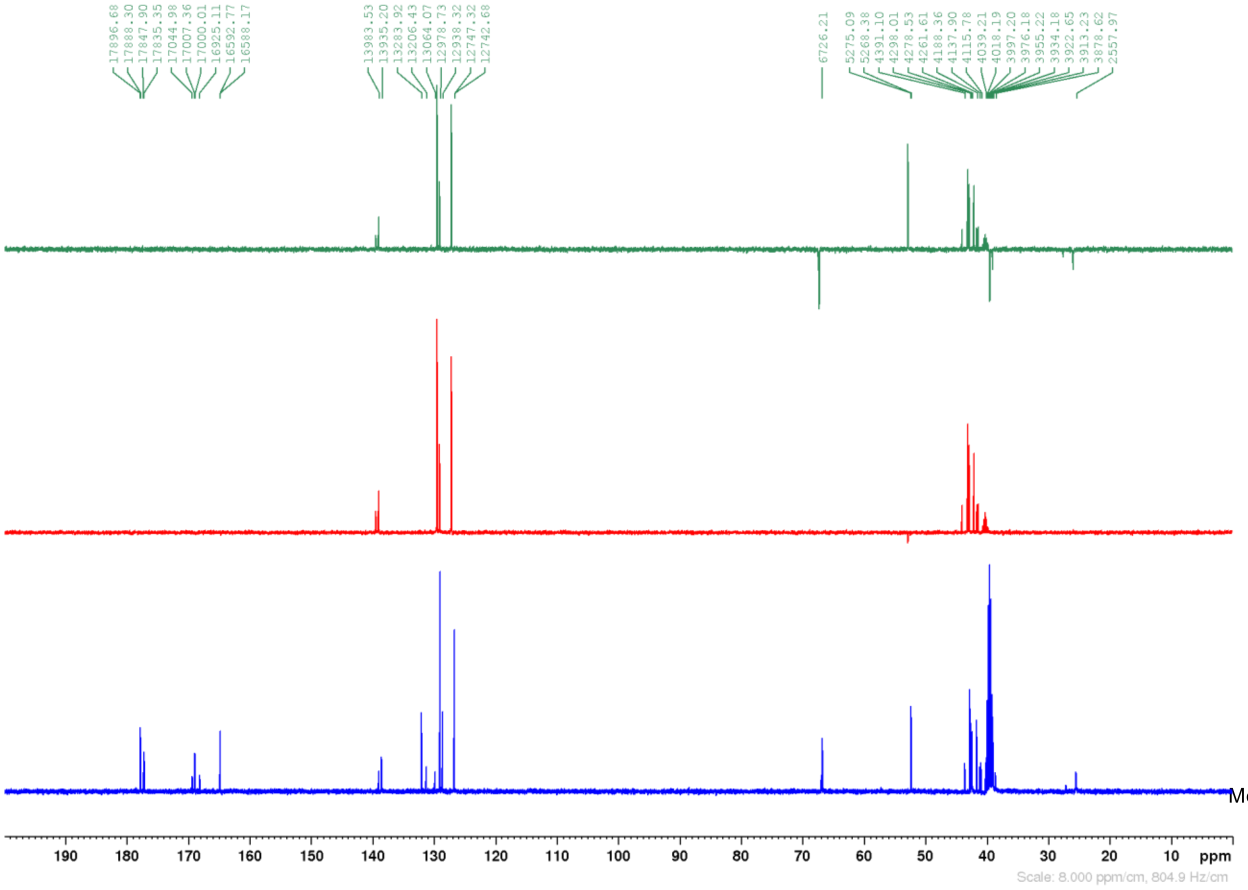


```
Current Data Parameters
NAME     3CO2Me-LactaminDMSOd6-20240820_IPL
EXPNO                 13
PROCNO                 1

F2 - Acquisition Parameters
Date_           20240820
Time               14.54 h
INSTRUM   Avance NANOBAY
PROBHD    Z163739_0358 (
PULPROG             zgpg
TD                 65536
SOLVENT             DMSO
NS                   512
DS                     0
SWH            25000.000 Hz
FIDRES          0.762939 Hz
AQ             1.3107200 sec
RG                   101
DW                20.000 usec
DE                  6.50 usec
TE                 297.6 K
D1            1.50000000 sec
D11           0.03000000 sec
TD0                    1
SFO1         100.6293690 MHz
NUC1                 13C
P1                  8.00 usec
PLW1         89.00000000 W
SFO2         400.1518007 MHz
NUC2                  1H
CPDPRG[2         waltz65
PCPD2              90.00 usec
PLW2         22.79999924 W
PLW12         0.18015000 W
PLW13         0.09061300 W

F2 - Processing parameters
SI                 32768
SF           100.6178441 MHz
WDW                   EM
SSB                    0
LB                  0.30 Hz
GB                     0
PC                  1.40
```

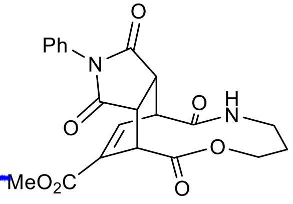

+

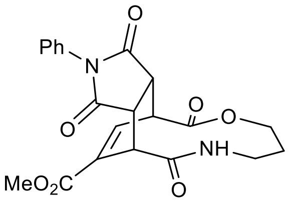

9j **9j'**

^{1}H NMR of 9k

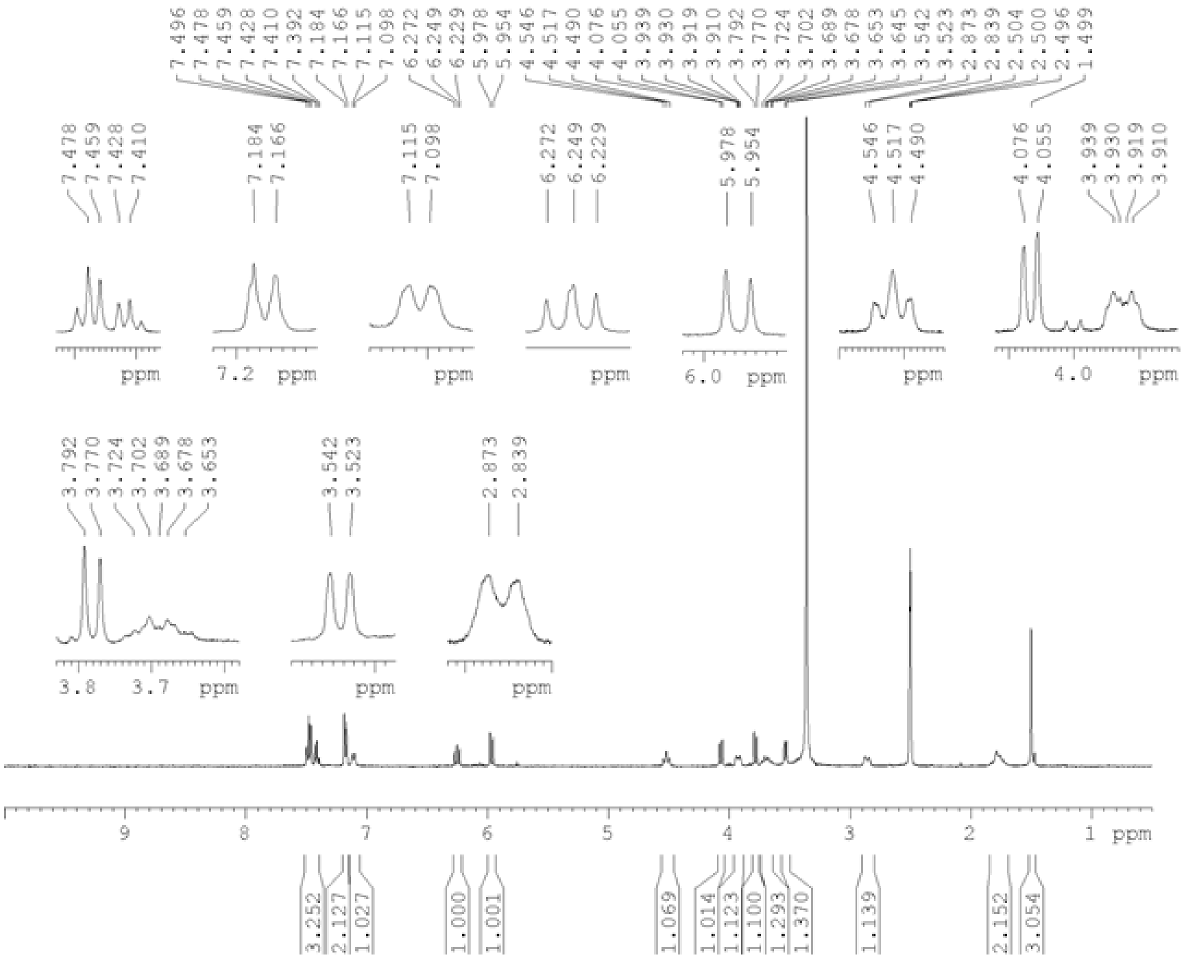

Current Data Parameters
NAME 20240618 DATA-5-Me-lactam
EXPNO 1
PROCNO 1

F2 - Acquisition Parameters
Date_ 20240618
Time 23.09 h
INSTRUM Avance NANOBAY
PROBHD Z163739_0358 (
PULPROG zg30
TD 32768
SOLVENT DMSO
NS 1
DS 0
SWH 5882.353 Hz
FIDRES 0.359030 Hz
AQ 2.7852800 sec
RG 101
DW 85.000 usec
DE 9.26 usec
TE 293.8 K
D1 1.50000000 sec
TD0 1
SFO1 400.1526010 MHz
NUC1 1H
P0 2.67 usec
P1 8.00 usec
PLW1 23.43799973 W

F2 - Processing parameters
SI 32768
SF 400.1500056 MHz
WDW EM
SSB 0
LB 0.10 Hz
GB 0
PC 1.00

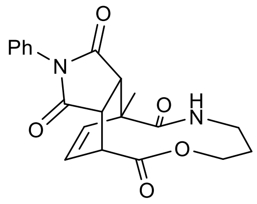

9k

^{13}C{1H} and DEPT 90, 135 NMR of 9k

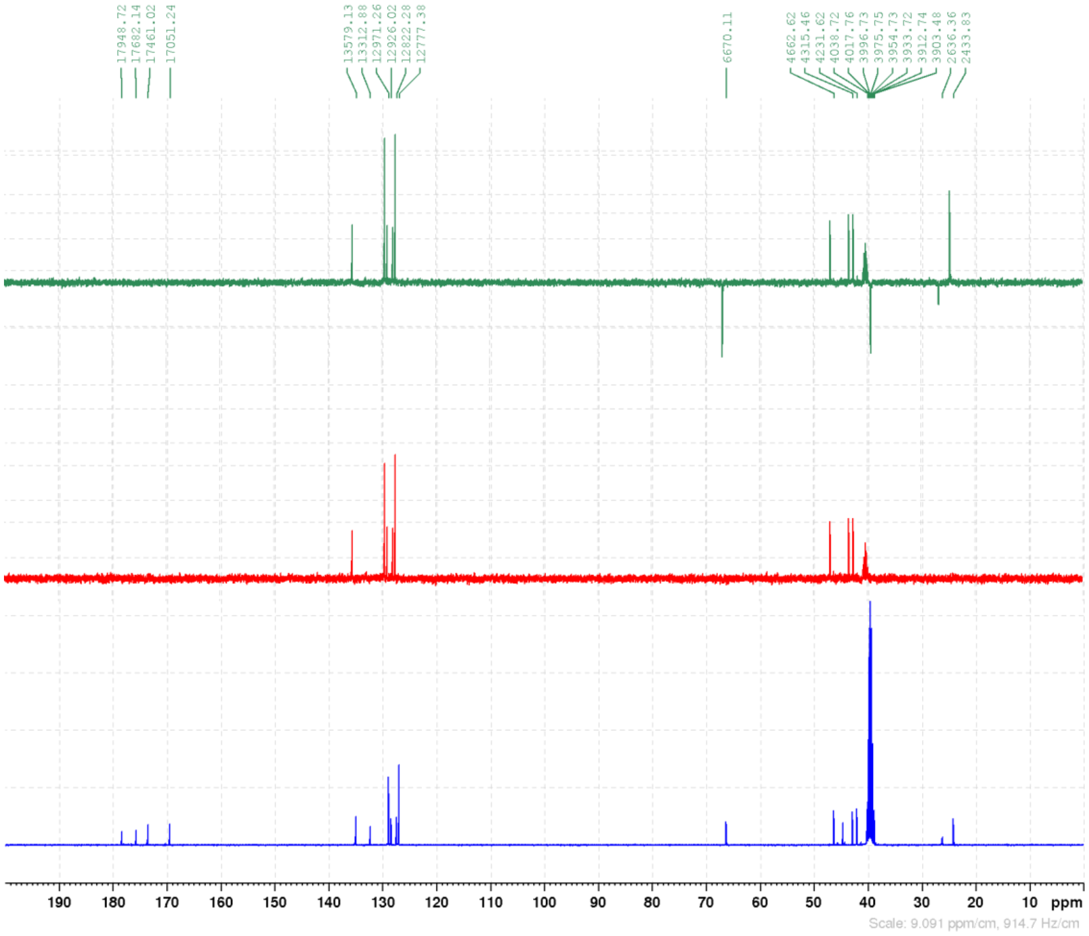


```
Current Data Parameters
NAME      20240618 DATA-5-Me-lactam
EXPNO                    13
PROCNO                    1

F2 - Acquisition Parameters
Date_              20240619
Time                   9.04 h
INSTRUM     Avance NANOBAY
PROBHD    Z163739_0358 (
PULPROG                zgpg
TD                    65536
SOLVENT                DMSO
NS                    10360
DS                        0
SWH               25000.000 Hz
FIDRES             0.762939 Hz
AQ                1.3107200 sec
RG                      101
DW                   20.000 usec
DE                     6.50 usec
TE                    295.8 K
D1               1.50000000 sec
D11              0.03000000 sec
TD0                       1
SFO1            100.6293690 MHz
NUC1                    13C
P1                     8.00 usec
PLW1            89.00000000 W
SFO2            400.1518007 MHz
NUC2                     1H
CPDPRG[2            waltz65
PCPD2                 90.00 usec
PLW2            22.79999924 W
PLW12            0.18015000 W
PLW13            0.09061300 W

F2 - Processing parameters
SI                    32768
SF              100.6178441 MHz
WDW                      EM
SSB                       0
LB                     0.30 Hz
GB                        0
PC                     1.40
```

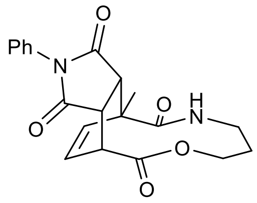

9k

^{1}H NMR of 9l

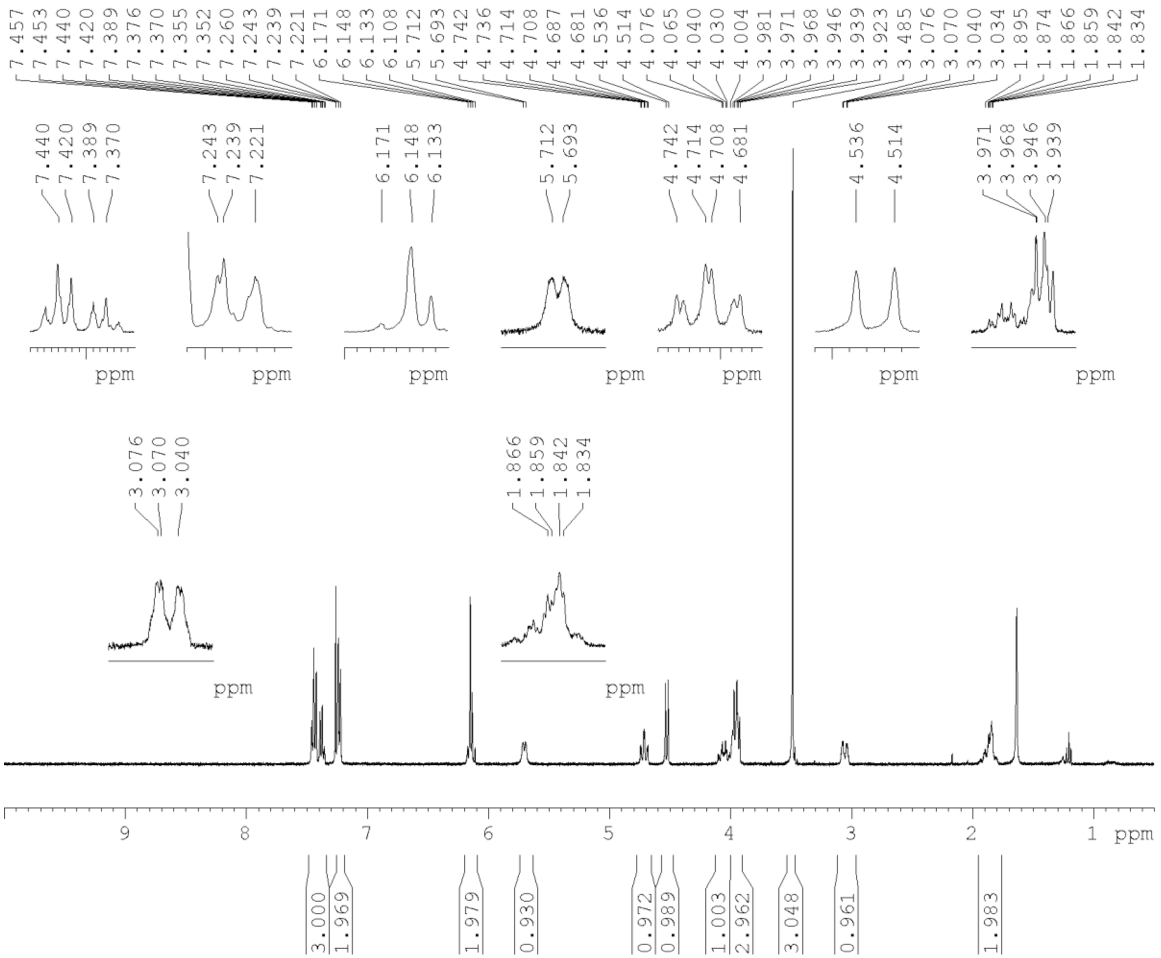

Current Data Parameters
NAME 20240528 DATA-5-OMe-lactam
EXPNO 1
PROCNO 1

F2 - Acquisition Parameters
Date_ 20240528
Time 23.37
INSTRUM spect
PROBHD 5 mm BBO BB-1H
PULPROG zg30
TD 32768
SOLVENT CDCl3
NS 33
DS 0
SWH 6009.615 Hz
FIDRES 0.183399 Hz
AQ 2.7262976 sec
RG 228
DW 83.200 usec
DE 6.50 usec
TE 295.3 K
D1 1.50000000 sec
TD0 1

======== CHANNEL f1 ========
NUC1 1H
P1 14.00 usec
PL1 -1.00 dB
PL1W 7.55784369 W
SFO1 400.1326010 MHz

F2 - Processing parameters
SI 32768
SF 400.1300098 MHz
WDW EM
SSB 0
LB 0 Hz
GB 0
PC 1.00

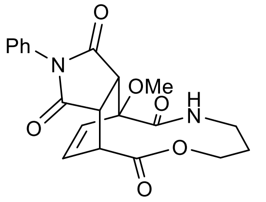

9l

^{13}C{1H} and DEPT 90, 135 NMR of 9l

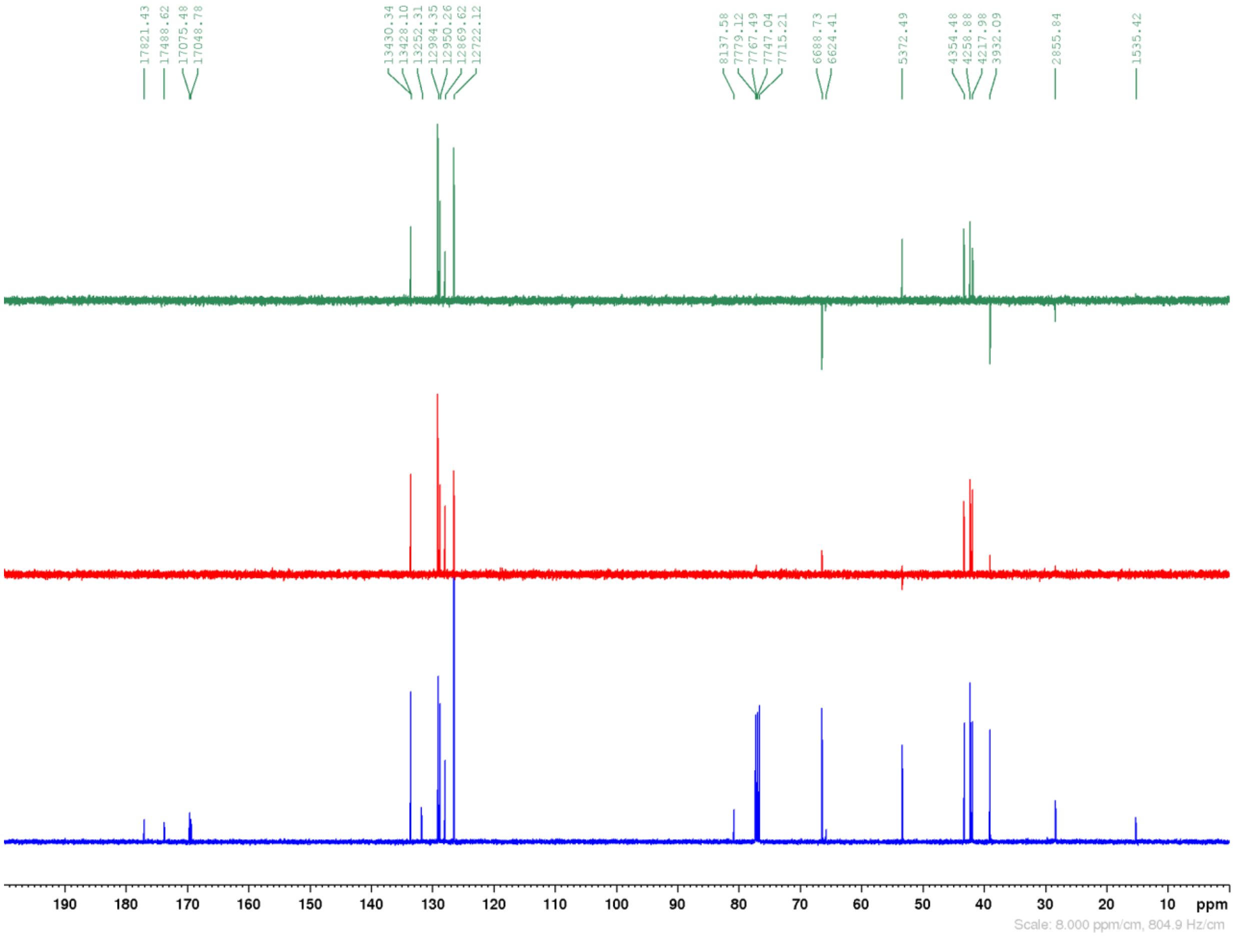

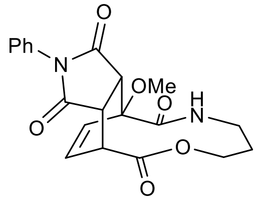

9l

^{1}H NMR of 9n

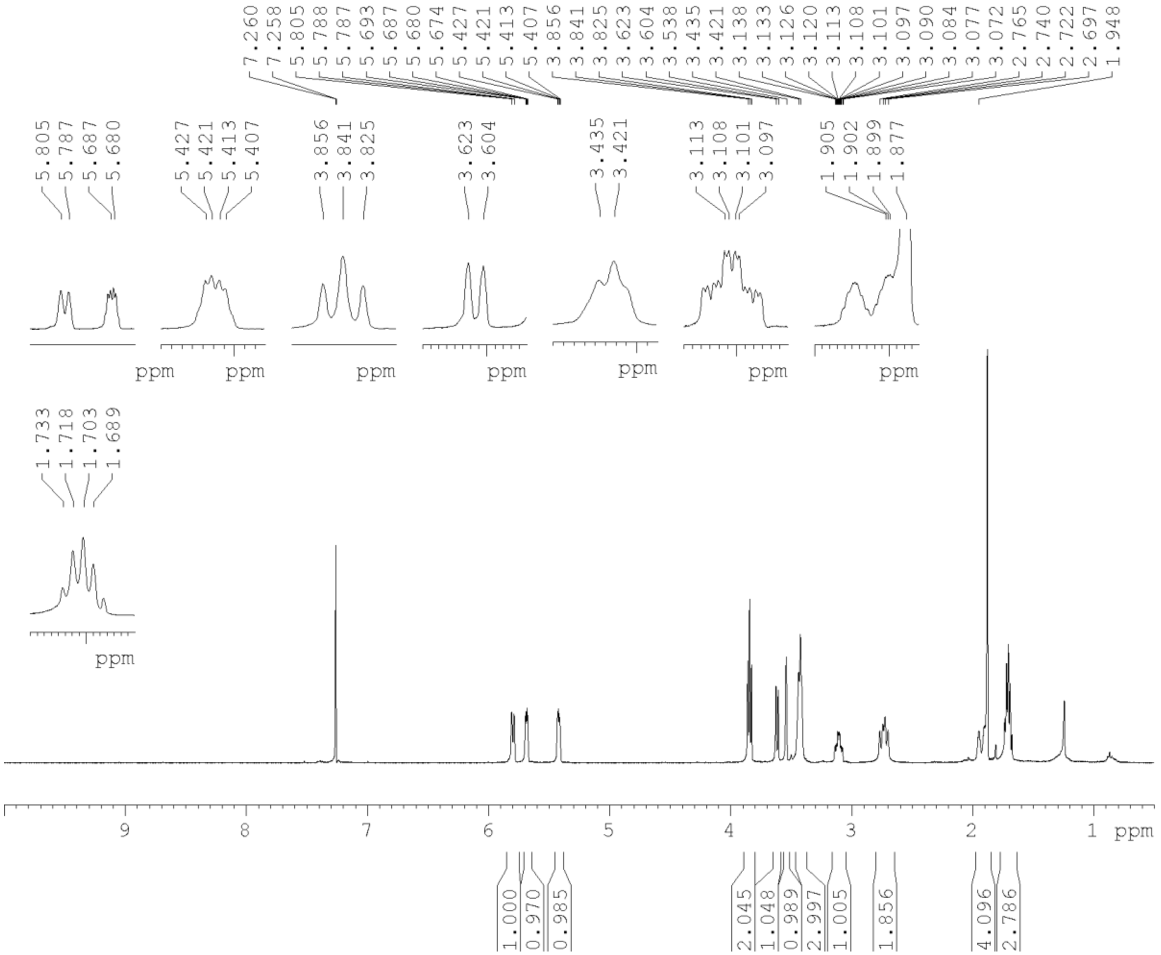


```
Current Data Parameters
NAME     20240731 DATA-4-Me-cp-lactam
EXPNO                  1
PROCNO                 1

F2 - Acquisition Parameters
Date_           20240731
Time               14.37 h
INSTRUM  Avance NANOBAY
PROBHD   Z163739_0358 (
PULPROG             zg30
TD                 32768
SOLVENT            CDCl3
NS                    17
DS                     0
SWH             5882.353 Hz
FIDRES          0.359030 Hz
AQ             2.7852800 sec
RG                   101
DW                85.000 usec
DE                  9.26 usec
TE                 296.1 K
D1            1.50000000 sec
TD0                    1
SFO1         400.1526010 MHz
NUC1                  1H
P0                  2.67 usec
P1                  8.00 usec
PLW1         23.43799973 W

F2 - Processing parameters
SI                 32768
SF           400.1500100 MHz
WDW                   EM
SSB                    0
LB                  0.10 Hz
GB                     0
PC                  1.00
```

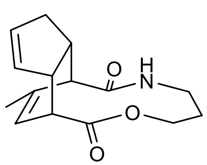

9n

^{13}C{1H} and DEPT 90, 135 NMR of 9n

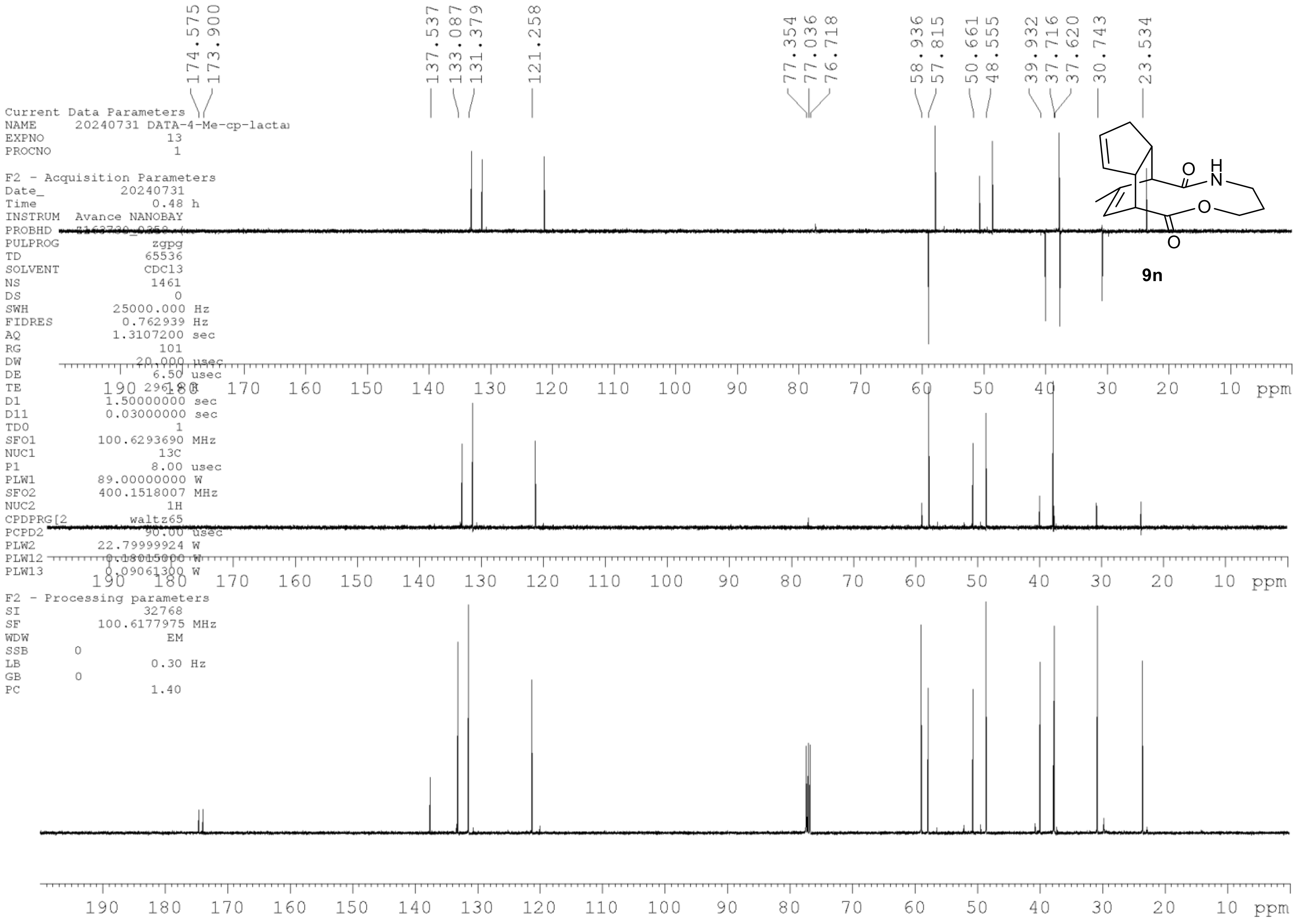

^{1}H NMR of 9v

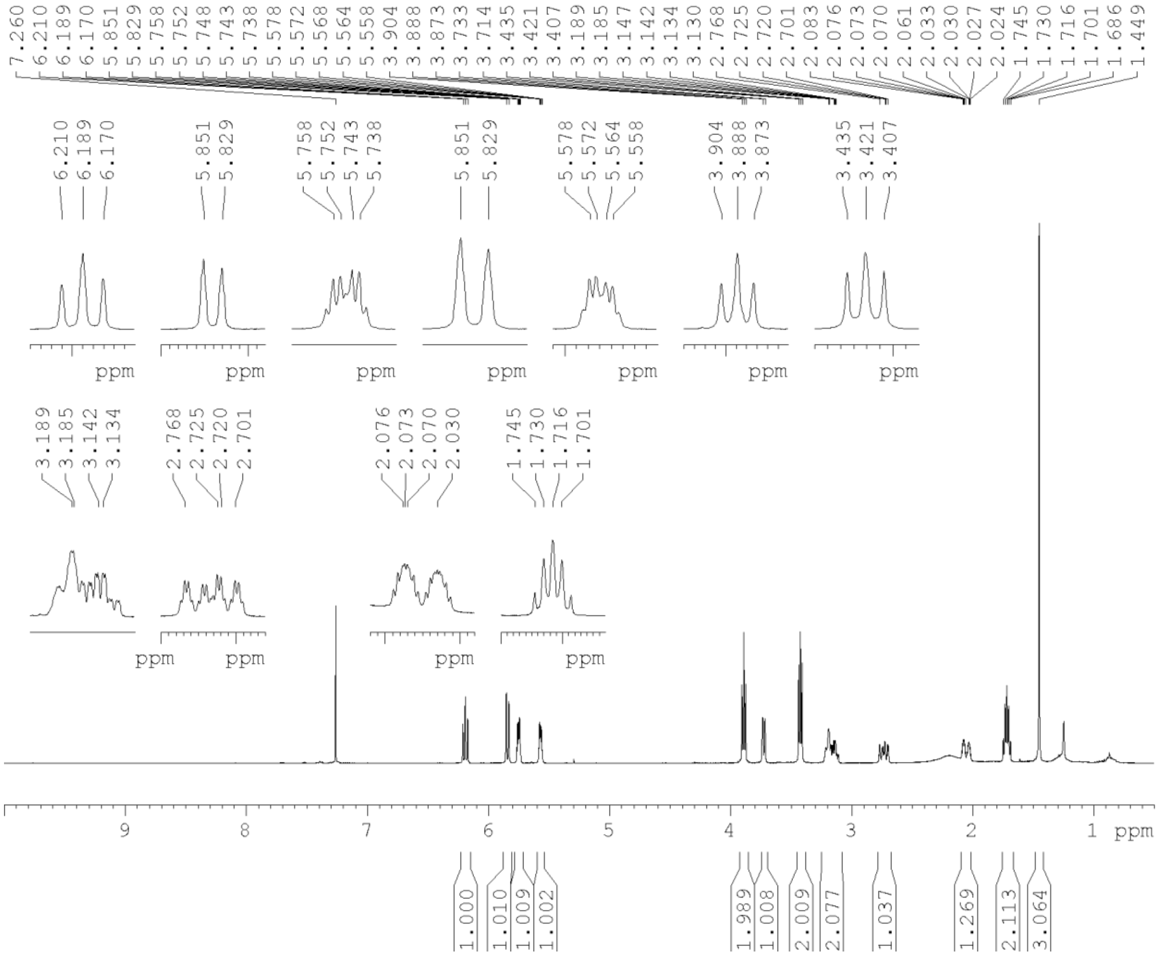

Current Data Parameters
NAME 20240306 2-me-cp-lac col-1
EXPNO 3
PROCNO 1

F2 - Acquisition Parameters
Date_ 20240306
Time 21.26 h
INSTRUM Avance NANOBAY
PROBHD Z163739_0358 (
PULPROG zg30
TD 32768
SOLVENT CDCl3
NS 2
DS 4
SWH 5882.353 Hz
FIDRES 0.359030 Hz
AQ 2.7852800 sec
RG 101
DW 85.000 usec
DE 9.26 usec
TE 296.2 K
D1 10.00000000 sec
TD0 1
SFO1 400.1526010 MHz
NUC1 1H
P0 2.67 usec
P1 8.00 usec
PLW1 21.10000038 W

F2 - Processing parameters
SI 32768
SF 400.1500097 MHz
WDW EM
SSB 0
LB 0.10 Hz
GB 0
PC 1.00

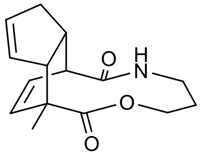

9v

^{13}C{1H} and DEPT 90, 135 NMR of 9v

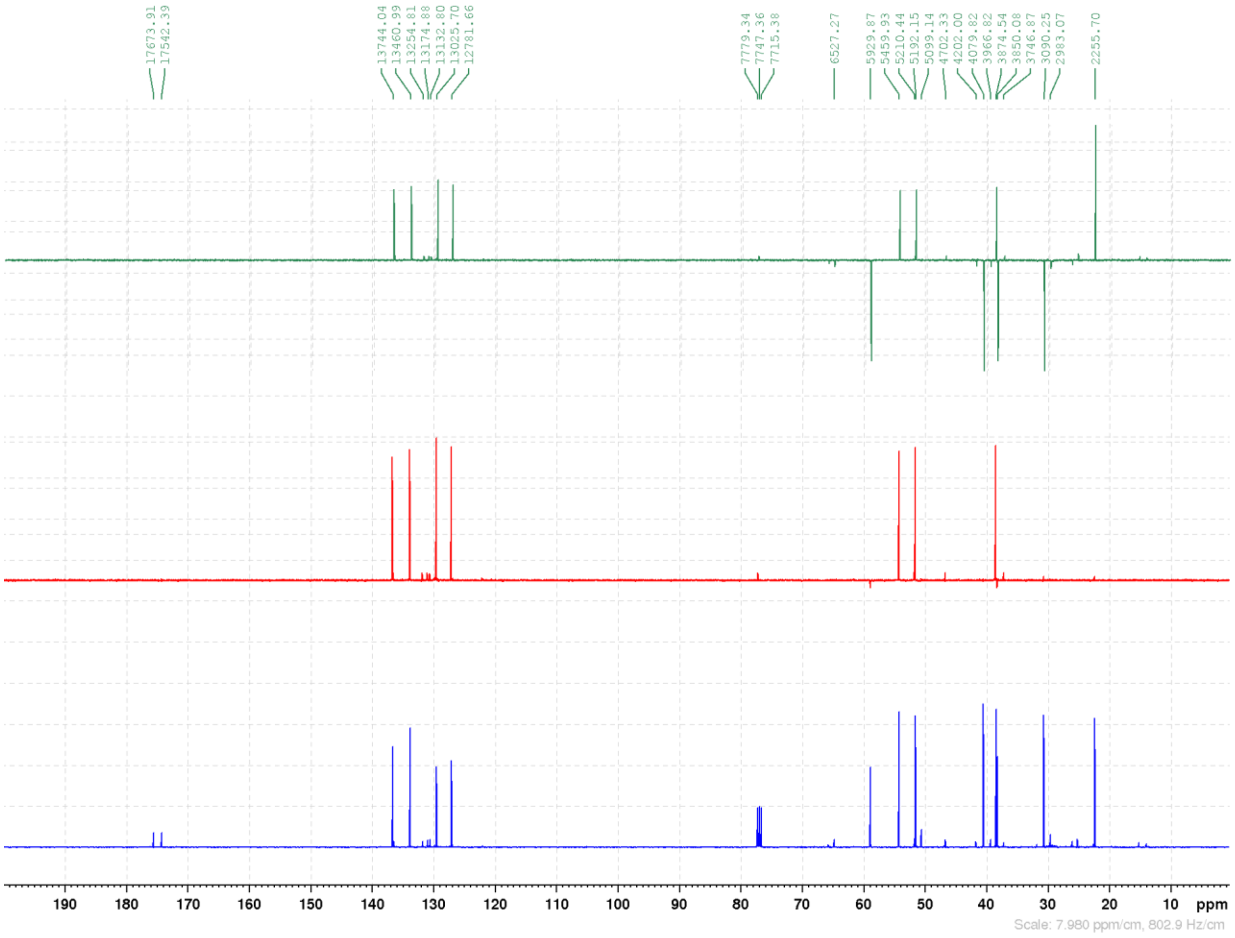

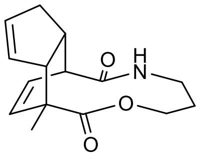

9v

^{1}H NMR of 9q

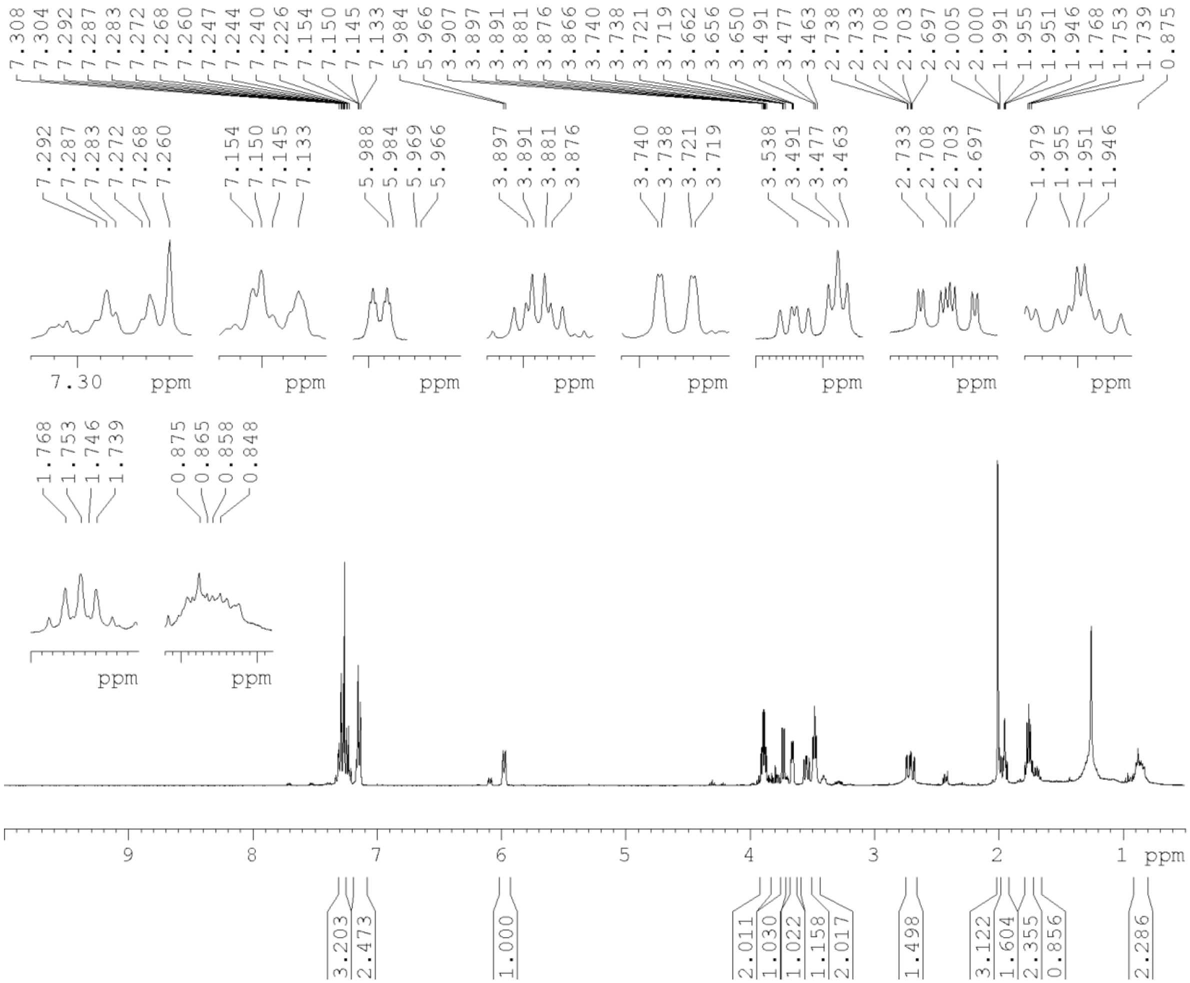

```
Current Data Parameters
NAME     20240722 DATA-4-Me-sty-lac-col
EXPNO                    1
PROCNO                   1

F2 - Acquisition Parameters
Date_          20240722
Time              23.14 h
INSTRUM  Avance NANOBAY
PROBHD   Z163739_0358 (
PULPROG            zg30
TD                32768
SOLVENT           CDCl3
NS                   17
DS                    0
SWH            5882.353 Hz
FIDRES         0.359030 Hz
AQ            2.7852800 sec
RG              63.0303
DW               85.000 usec
DE                 9.26 usec
TE                294.2 K
D1           1.50000000 sec
TD0                   1
SFO1        400.1526010 MHz
NUC1                 1H
P0                 2.67 usec
P1                 8.00 usec
PLW1        23.43799973 W

F2 - Processing parameters
SI                32768
SF          400.1500097 MHz
WDW                  EM
SSB                   0
LB                 0.10 Hz
GB                    0
PC                 1.00
```

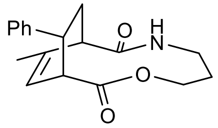

9q

^{13}C{1H} and DEPT 90, 135 NMR of 9q

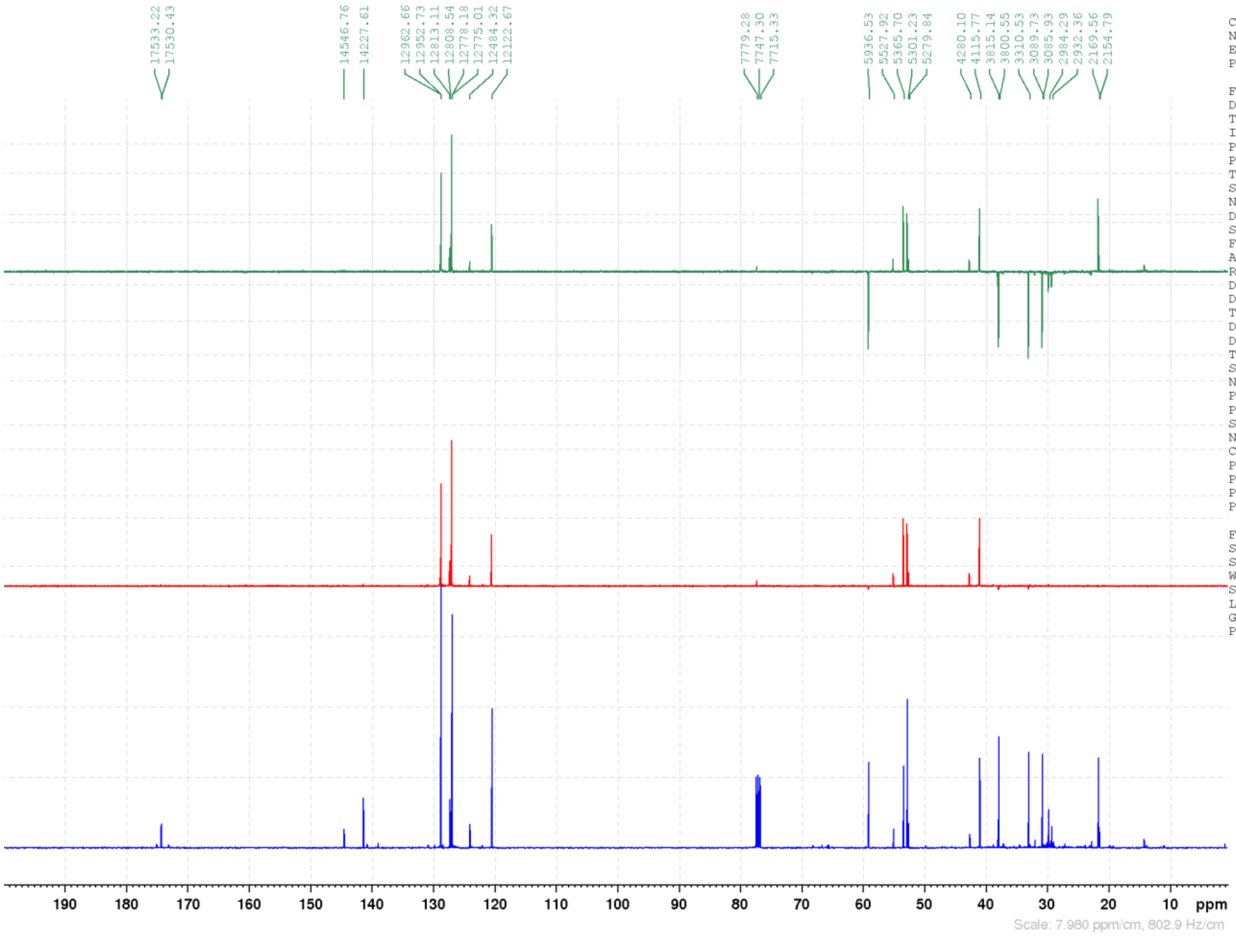

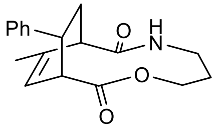

9q

^{1}H NMR of 9w

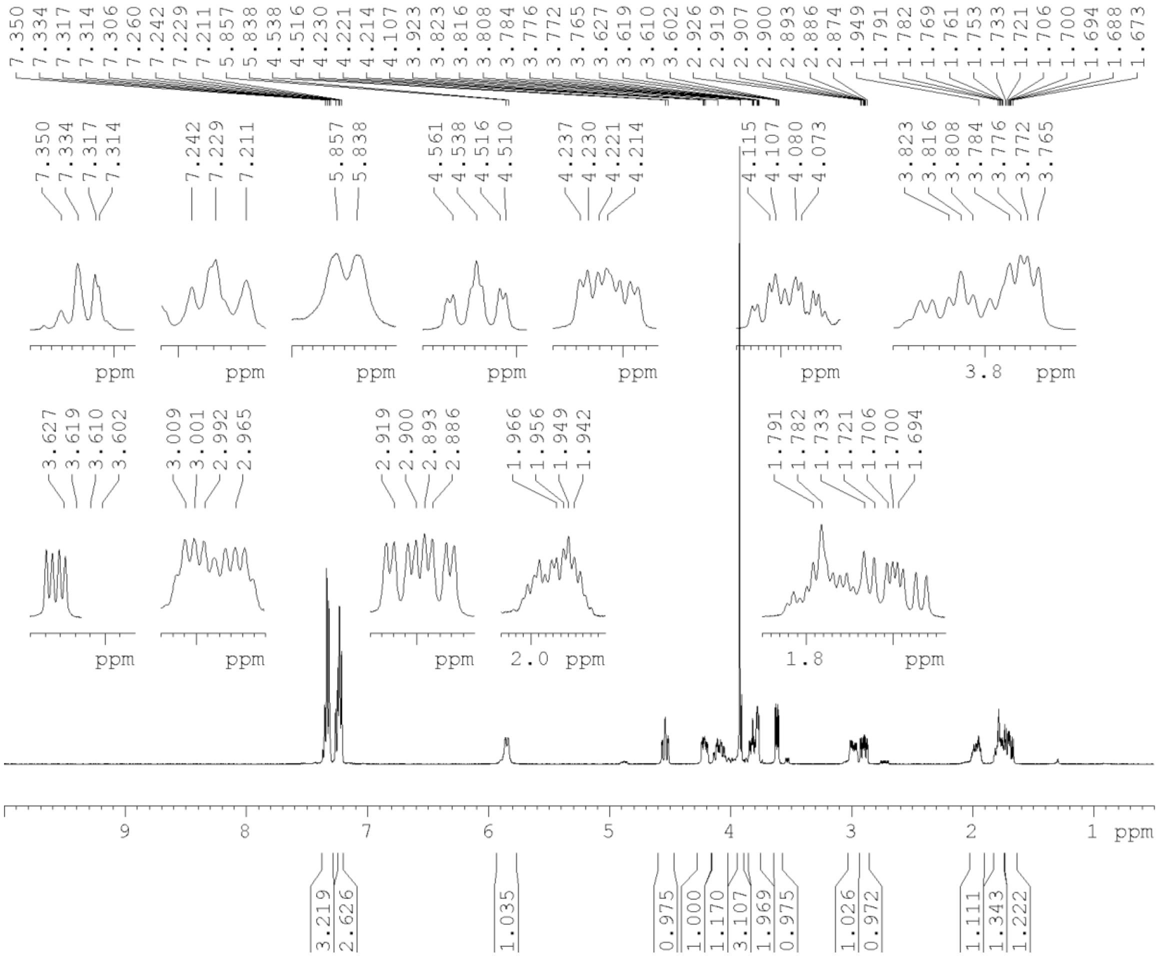

Current Data Parameters
NAME 20240627 DATA-4-CO2Me-sty-lac
EXPNO 1
PROCNO 1

F2 - Acquisition Parameters
Date_ 20240627
Time 23.17 h
INSTRUM Avance NANOBAY
PROBHD Z163739_0358 (
PULPROG zg30
TD 32768
SOLVENT CDCl3
NS 17
DS 0
SWH 5882.353 Hz
FIDRES 0.359030 Hz
AQ 2.7852800 sec
RG 101
DW 85.000 usec
DE 9.26 usec
TE 296.5 K
D1 1.50000000 sec
TD0 1
SFO1 400.1526010 MHz
NUC1 1H
P0 2.67 usec
P1 8.00 usec
PLW1 21.10000038 W

F2 - Processing parameters
SI 32768
SF 400.1499914 MHz
WDW EM
SSB 0
LB 0.10 Hz
GB 0
PC 1.00

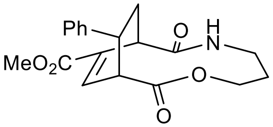

9w

^{13}C{1H} and DEPT 90, 135 NMR of 9w

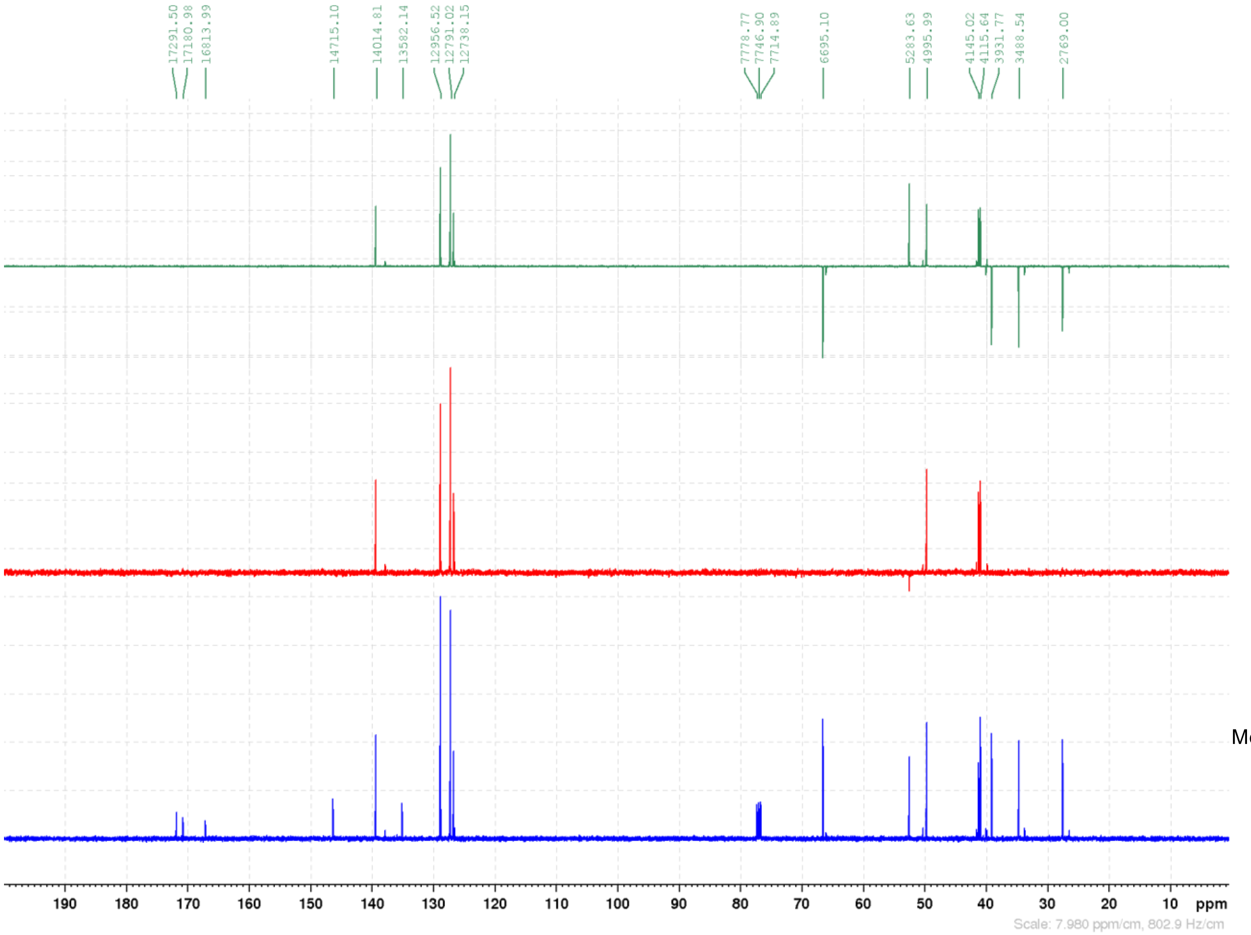

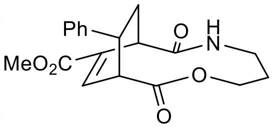

9w

^{1}H NMR of 9x

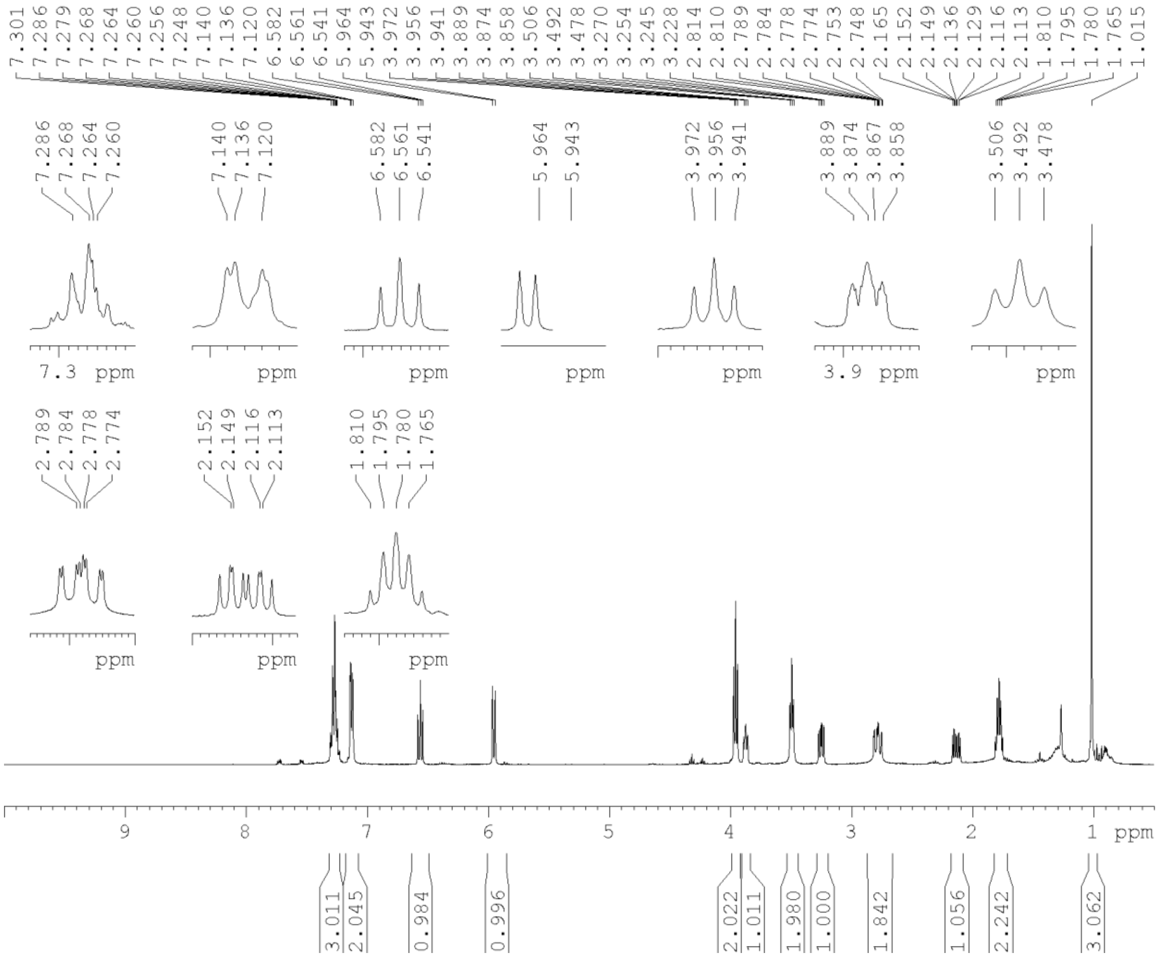

```
Current Data Parameters
NAME     20240624 DATA-2-Me-sty-lactam
EXPNO                  1
PROCNO                 1

F2 - Acquisition Parameters
Date_          20240624
Time              22.41 h
INSTRUM  Avance NANOBAY
PROBHD   Z163739_0358 (
PULPROG            zg30
TD                32768
SOLVENT           CDCl3
NS                    1
DS                    0
SWH            5882.353 Hz
FIDRES         0.359030 Hz
AQ            2.7852800 sec
RG              56.2162
DW               85.000 usec
DE                 9.26 usec
TE                294.0 K
D1           1.50000000 sec
TD0                   1
SFO1        400.1526010 MHz
NUC1                 1H
P0                 2.67 usec
P1                 8.00 usec
PLW1        23.43799973 W

F2 - Processing parameters
SI                32768
SF          400.1500012 MHz
WDW                  EM
SSB                   0
LB                 0.10 Hz
GB                    0
PC                 1.00
```

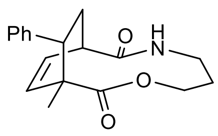

9x

$^{13}C\{1H\}$ and DEPT 90, 135 NMR of 9x

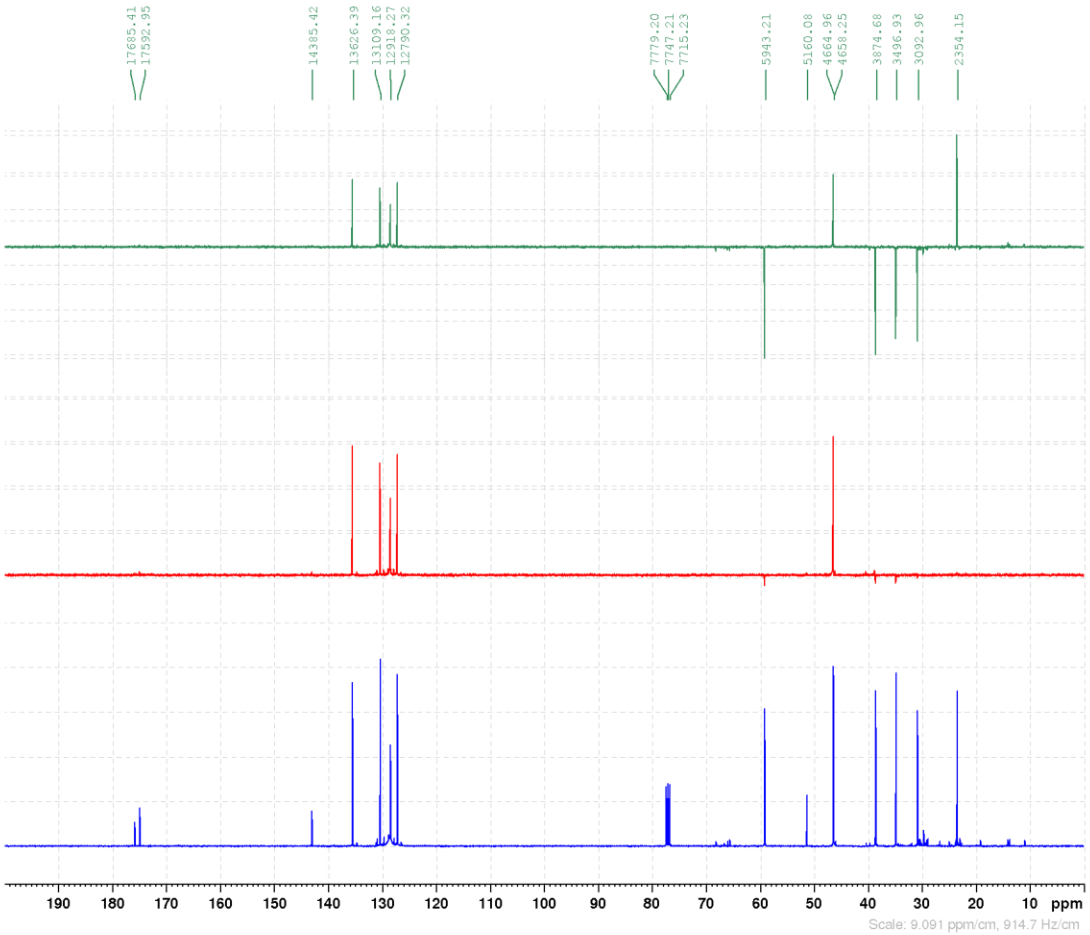


```
Current Data Parameters
NAME      20240624 DATA-2-Me-sty-lactam
EXPNO                    13
PROCNO                    1

F2 - Acquisition Parameters
Date_              20240625
Time                   8.30 h
INSTRUM   Avance NANOBAY
PROBHD    Z163739_0358 (
PULPROG              zgpg
TD                        0
SOLVENT               CDCl3
NS                    11459
DS                        0
SWH              25000.000 Hz
FIDRES                    0 Hz
AQ                        0 sec
RG                      101
DW                   20.000 usec
DE                     6.50 usec
TE                    295.4 K
D1               1.50000000 sec
D11              0.03000000 sec
TD0                       1
SFO1            100.6293690 MHz
NUC1                    13C
P1                     8.00 usec
PLW1            89.00000000 W
SFO2            400.1518007 MHz
NUC2                     1H
CPDPRG[2            waltz65
PCPD2                 90.00 usec
PLW2            22.79999924 W
PLW12            0.18015000 W
PLW13            0.09061300 W

F2 - Processing parameters
SI                    32768
SF              100.6178045 MHz
WDW                      EM
SSB                       0
LB                     0.30 Hz
GB                        0
PC                     1.40
```

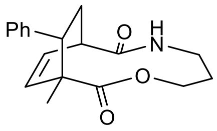

9x

^{1}H NMR of 9y

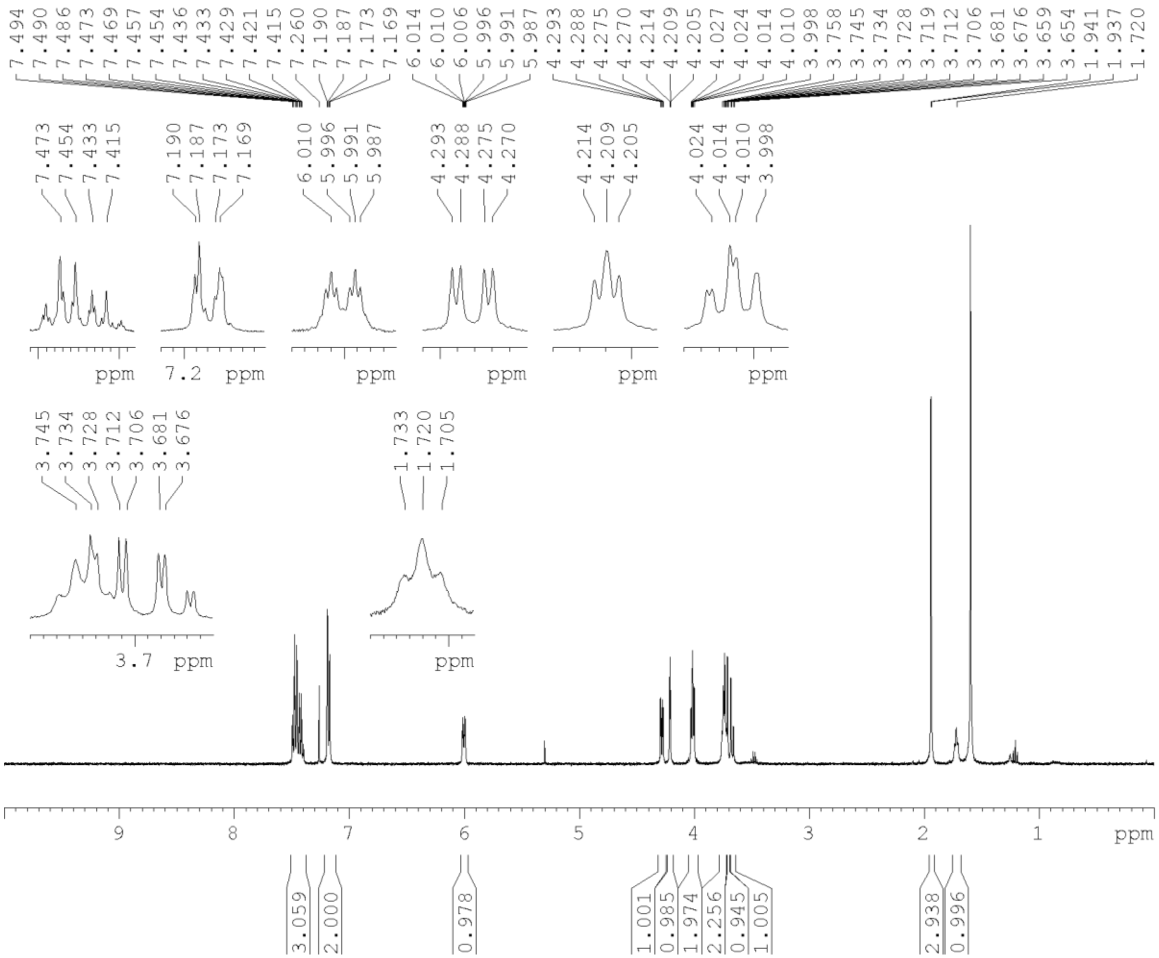

```
Current Data Parameters
NAME     20240604 DATA-4-Me-lactam-2C
EXPNO                   1
PROCNO                  1

F2 - Acquisition Parameters
Date_           20240604
Time               23.24 h
INSTRUM  Avance NANOBAY
PROBHD   Z163739_0358 (
PULPROG             zg30
TD                 32768
SOLVENT            CDCl3
NS                     1
DS                     0
SWH             5882.353 Hz
FIDRES          0.359030 Hz
AQ             2.7852800 sec
RG                   101
DW                85.000 usec
DE                  9.26 usec
TE                 294.1 K
D1            1.50000000 sec
TD0                    1
SFO1         400.1526010 MHz
NUC1                  1H
P0                  2.67 usec
P1                  8.00 usec
PLW1         21.10000038 W

F2 - Processing parameters
SI                 32768
SF           400.1500100 MHz
WDW                   EM
SSB                    0
LB                  0.10 Hz
GB                     0
PC                  1.00
```

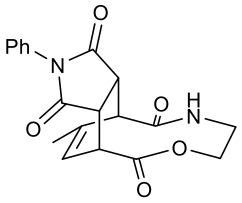

9y

^{13}C{1H} and DEPT 90, 135 NMR of 9y

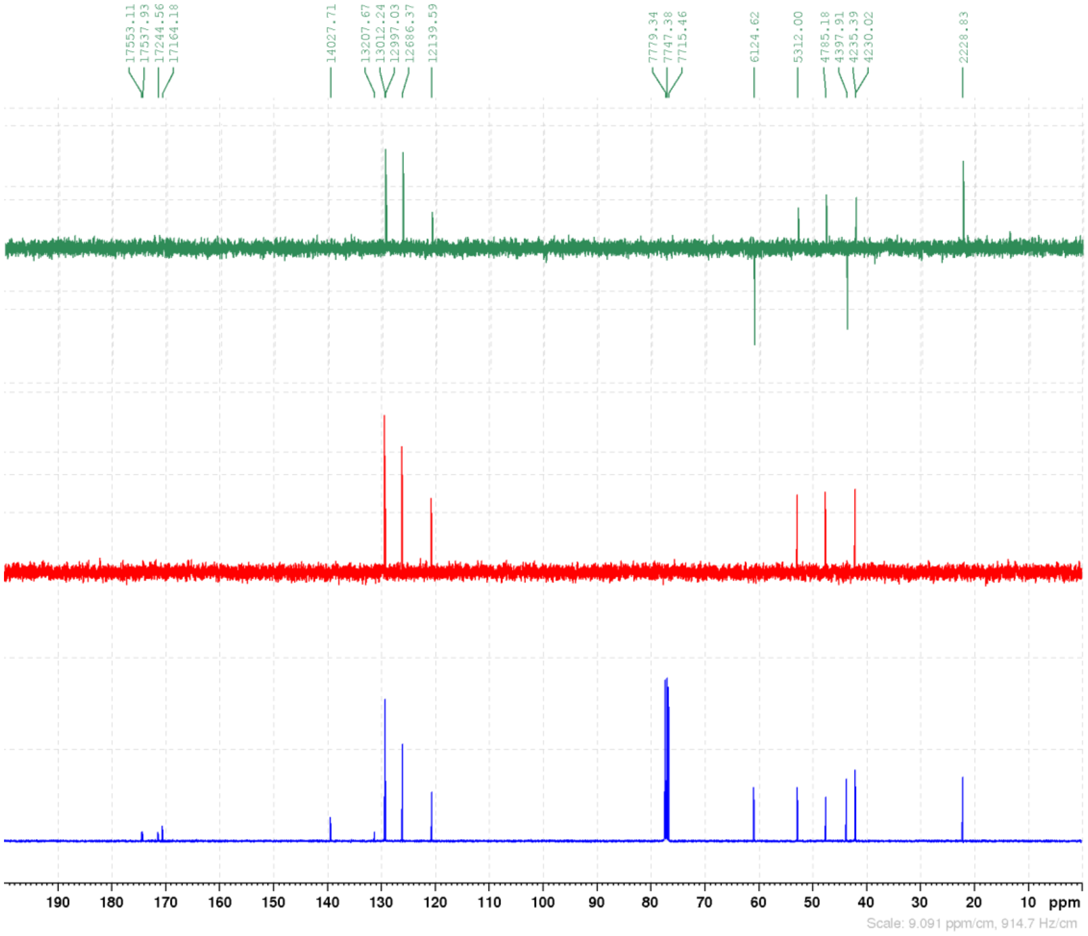


```
Current Data Parameters
NAME      20240604 DATA-4-Me-lactam-2C
EXPNO                   13
PROCNO                   1

F2 - Acquisition Parameters
Date_             20240605
Time                  8.30 h
INSTRUM   Avance NANOBAY
PROBHD    Z163739_0358 (
PULPROG               zgpg
TD                   65536
SOLVENT              CDCl3
NS                   11066
DS                       0
SWH              25000.000 Hz
FIDRES            0.762939 Hz
AQ               1.3107200 sec
RG                     101
DW                  20.000 usec
DE                    6.50 usec
TE                   295.3 K
D1              1.50000000 sec
D11             0.03000000 sec
TD0                      1
SFO1           100.6293690 MHz
NUC1                   13C
P0                    8.00 usec
P1                    8.00 usec
PLW1           89.00000000 W
SFO2           400.1518007 MHz
NUC2                    1H
CPDPRG[2           waltz65
PCPD2                90.00 usec
PLW2           22.79999924 W
PLW12           0.18015000 W
PLW13           0.09061300 W

F2 - Processing parameters
SI                   32768
SF             100.6178007 MHz
WDW                     EM
SSB                      0
LB                    0.30 Hz
GB                       0
PC                    1.40
```

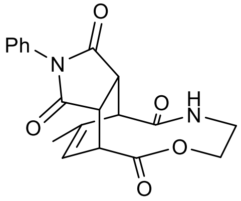

9y

ORTEP and x-ray data of 4a

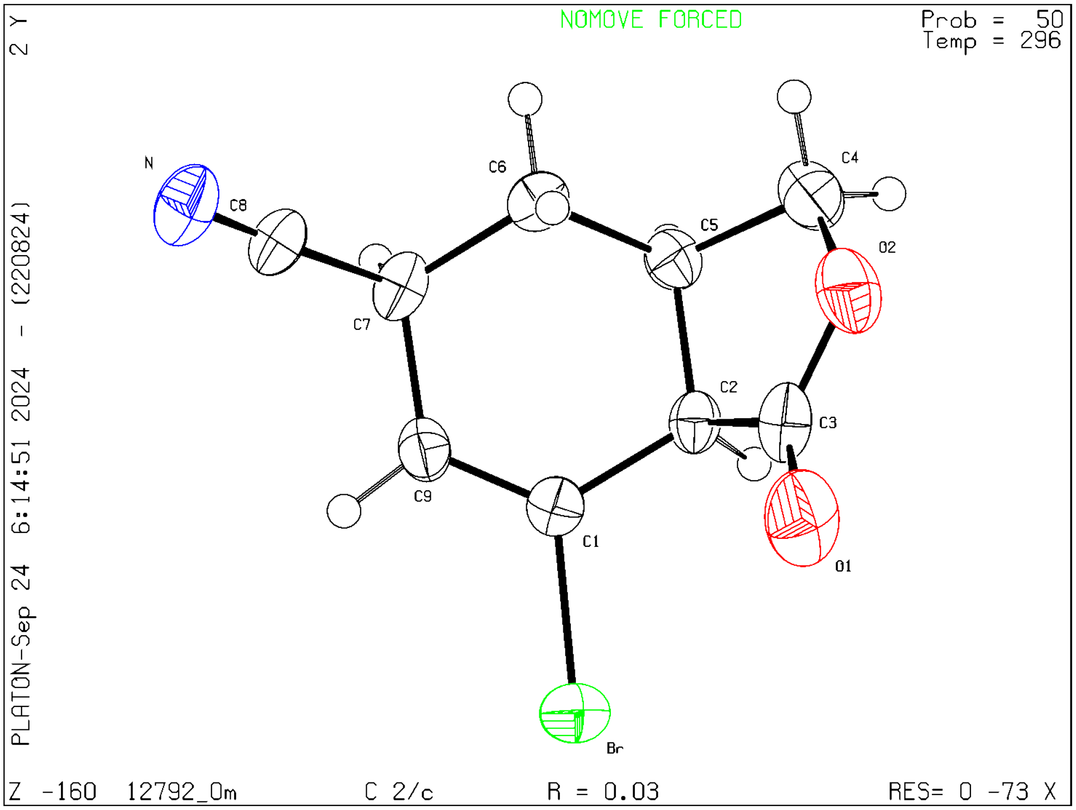

Table S1. Crystal data and structure refinement for **4a**.(CCDC 2386153)

Identification code	**4a**	
Empirical formula	C9 H8 Br N O2	
Formula weight	242.07	
Temperature	296(2) K	
Wavelength	0.71073 Å	
Crystal system	Monoclinic	
Space group	C2/c	
Unit cell dimensions	a = 13.9161(11) Å	a= 90°.
	b = 8.2751(7) Å	b= 109.809(2)°.
	c = 16.7594(13) Å	g = 90°.
Volume	1815.8(3) $Å^3$	
Z	8	
Density (calculated)	1.771 Mg/m^3	
Absorption coefficient	4.491 mm^{-1}	
F(000)	960	
Crystal size	0.400 x 0.300 x 0.200 mm^3	
Theta range for data collection	2.583 to 28.709°.	
Index ranges	-18<=h<=13, -10<=k<=11, -20<=l<=22	
Reflections collected	10856	
Independent reflections	2296 [R(int) = 0.0506]	
Completeness to theta = 25.242°	100.0 %	
Absorption correction	None	
Refinement method	Full-matrix least-squares on F^2	
Data / restraints / parameters	2296 / 0 / 119	
Goodness-of-fit on F^2	1.001	
Final R indices [I>2sigma(I)]	R1 = 0.0337, wR2 = 0.0577	
R indices (all data)	R1 = 0.0636, wR2 = 0.0654	
Extinction coefficient	0.0064(3)	
Largest diff. peak and hole	0.472 and -0.590 e.$Å^{-3}$	

Table S2. Atomic coordinates (x 10^4) and equivalent isotropic displacement parameters ($Å^2$ x 10^3) for **4a**. U(eq) is defined as one third of the trace of the orthogonalized U^{ij} tensor.

	x	y	z	U(eq)
Br	1117(1)	609(1)	4030(1)	49(1)
O(1)	946(2)	2109(2)	5852(2)	62(1)
O(2)	1625(2)	615(2)	7007(1)	49(1)
N	5581(2)	1012(3)	6184(2)	47(1)
C(1)	2125(2)	-38(3)	5067(2)	30(1)
C(2)	1733(2)	-504(3)	5762(2)	28(1)
C(3)	1375(2)	905(3)	6171(2)	38(1)
C(4)	2131(2)	-936(3)	7228(2)	42(1)
C(5)	2533(2)	-1322(3)	6515(2)	31(1)
C(6)	3586(2)	-587(3)	6683(2)	36(1)
C(7)	3917(2)	-669(3)	5900(2)	33(1)
C(8)	4857(2)	276(3)	6046(2)	35(1)
C(9)	3080(2)	-101(3)	5109(2)	31(1)

Table S3. Bond lengths [Å] and angles [°] for **4a**.

Br-C(1)	1.903(2)
O(1)-C(3)	1.191(3)
O(2)-C(3)	1.347(3)
O(2)-C(4)	1.451(3)
N-C(8)	1.132(3)
C(1)-C(9)	1.308(3)
C(1)-C(2)	1.495(3)
C(2)-C(3)	1.520(3)
C(2)-C(5)	1.529(3)
C(2)-H(2A)	0.9800
C(4)-C(5)	1.516(3)
C(4)-H(4A)	0.9700
C(4)-H(4B)	0.9700
C(5)-C(6)	1.522(3)
C(5)-H(5A)	0.9800
C(6)-C(7)	1.531(3)
C(6)-H(6A)	0.9700
C(6)-H(6B)	0.9700
C(7)-C(8)	1.472(3)
C(7)-C(9)	1.513(3)
C(7)-H(7A)	0.9800
C(9)-H(9A)	0.9300
C(3)-O(2)-C(4)	110.76(19)
C(9)-C(1)-C(2)	125.5(2)
C(9)-C(1)-Br	118.79(19)
C(2)-C(1)-Br	115.66(17)
C(1)-C(2)-C(3)	114.5(2)
C(1)-C(2)-C(5)	113.65(19)

C(3)-C(2)-C(5)	103.0(2)
C(1)-C(2)-H(2A)	108.4
C(3)-C(2)-H(2A)	108.4
C(5)-C(2)-H(2A)	108.4
O(1)-C(3)-O(2)	121.9(3)
O(1)-C(3)-C(2)	129.1(3)
O(2)-C(3)-C(2)	109.0(2)
O(2)-C(4)-C(5)	105.2(2)
O(2)-C(4)-H(4A)	110.7
C(5)-C(4)-H(4A)	110.7
O(2)-C(4)-H(4B)	110.7
C(5)-C(4)-H(4B)	110.7
H(4A)-C(4)-H(4B)	108.8
C(4)-C(5)-C(6)	111.1(2)
C(4)-C(5)-C(2)	101.4(2)
C(6)-C(5)-C(2)	111.22(19)
C(4)-C(5)-H(5A)	110.9
C(6)-C(5)-H(5A)	110.9
C(2)-C(5)-H(5A)	110.9
C(5)-C(6)-C(7)	112.0(2)
C(5)-C(6)-H(6A)	109.2
C(7)-C(6)-H(6A)	109.2
C(5)-C(6)-H(6B)	109.2
C(7)-C(6)-H(6B)	109.2
H(6A)-C(6)-H(6B)	107.9
C(8)-C(7)-C(9)	110.9(2)
C(8)-C(7)-C(6)	110.0(2)
C(9)-C(7)-C(6)	111.8(2)
C(8)-C(7)-H(7A)	108.0
C(9)-C(7)-H(7A)	108.0
C(6)-C(7)-H(7A)	108.0

N-C(8)-C(7)	177.8(3)
C(1)-C(9)-C(7)	121.7(2)
C(1)-C(9)-H(9A)	119.2
C(7)-C(9)-H(9A)	119.2

Symmetry transformations used to generate equivalent atoms:

Table S4. Anisotropic displacement parameters (Å^{2}x 10^3) for **5c**. The anisotropic displacement factor exponent takes the form: -2p^2[h^2 a*^{2}U^{11} + ... + 2 h k a* b* U^{12}]

	U^{11}	U^{22}	U^{33}	U^{23}	U^{13}	U^{12}
Br	38(1)	66(1)	39(1)	15(1)	8(1)	3(1)
O(1)	72(2)	42(1)	87(2)	10(1)	48(1)	22(1)
O(2)	58(1)	51(1)	47(1)	-13(1)	32(1)	-2(1)
N	33(1)	45(1)	66(2)	-1(1)	19(1)	-4(1)
C(1)	30(1)	28(1)	32(1)	0(1)	11(1)	0(1)
C(2)	26(1)	26(1)	33(1)	-3(1)	12(1)	-6(1)
C(3)	35(1)	35(2)	54(2)	-4(1)	26(1)	-2(1)
C(4)	45(2)	48(2)	35(2)	-2(1)	16(1)	-9(1)
C(5)	33(1)	30(1)	31(1)	1(1)	13(1)	-2(1)
C(6)	29(1)	44(2)	34(1)	1(1)	8(1)	-1(1)
C(7)	25(1)	32(1)	44(2)	-5(1)	13(1)	-2(1)
C(8)	26(1)	35(2)	44(2)	-2(1)	12(1)	4(1)
C(9)	31(1)	36(1)	31(1)	-4(1)	15(1)	-4(1)

Table S5. Hydrogen coordinates (x 10^4) and isotropic displacement parameters ($Å^2$ x 10^3)for **4a**.

	x	y	z	U(eq)
H(2A)	1159	-1251	5529	33
H(4A)	1653	-1759	7269	50
H(4B)	2687	-872	7766	50
H(5A)	2546	-2492	6426	37
H(6A)	4080	-1159	7148	44
H(6B)	3578	533	6852	44
H(7A)	4068	-1800	5815	40
H(9A)	3249	210	4640	38

ORTEP and x-ray data of 4k (CCDC 2386154)

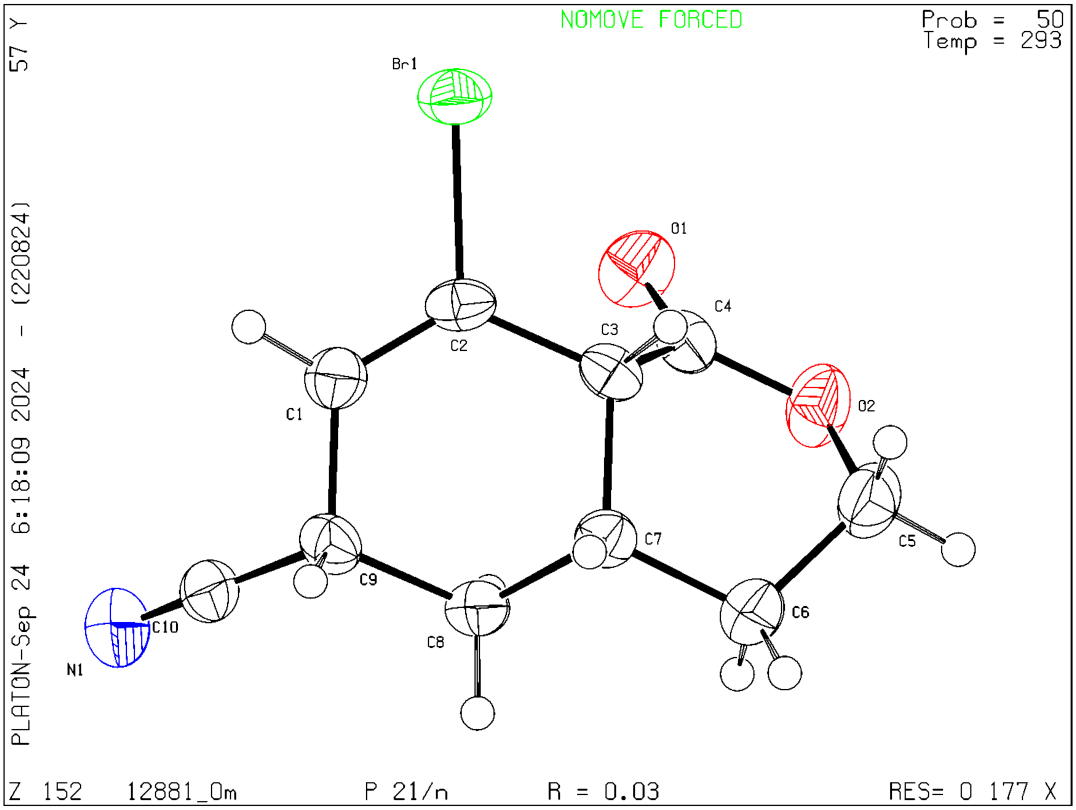

Table S6. Crystal data and structure refinement for **4k**

Identification code	**4k**	
Empirical formula	C10 H10 Br N O2	
Formula weight	256.10	
Temperature	293(2) K	
Wavelength	0.71073 Å	
Crystal system	Monoclinic	
Space group	$P2_1/n$	
Unit cell dimensions	a = 6.634(7) Å	a= 90°.
	b = 11.582(12) Å	b= 102.54(2)°.
	c = 13.199(14) Å	g = 90°.
Volume	990.0(18) Å^3	
Z	4	
Density (calculated)	1.718 Mg/m^3	
Absorption coefficient	4.124 mm^{-1}	
F(000)	512	
Crystal size	0.100 x 0.100 x 0.100 mm^3	
Theta range for data collection	2.364 to 25.995°.	
Index ranges	-8<=h<=8, -14<=k<=14, -16<=l<=16	
Reflections collected	16378	
Independent reflections	1945 [R(int) = 0.0624]	
Completeness to theta = 25.242°	100.0 %	
Absorption correction	None	
Refinement method	Full-matrix least-squares on F^2	
Data / restraints / parameters	1945 / 0 / 127	
Goodness-of-fit on F^2	1.038	
Final R indices [I>2sigma(I)]	R1 = 0.0284, wR2 = 0.0589	
R indices (all data)	R1 = 0.0440, wR2 = 0.0640	
Extinction coefficient	n/a	
Largest diff. peak and hole	0.249 and -0.520 e.Å^{-3}	

Table S7. Atomic coordinates (x 10^4) and equivalent isotropic displacement parameters ($Å^2$x 10^3) for **4k**. U(eq) is defined as one third of the trace of the orthogonalized U^{ij} tensor.

	x	y	z	U(eq)
Br(1)	3948(1)	7350(1)	1059(1)	41(1)
O(1)	496(3)	5515(2)	1750(2)	45(1)
O(2)	355(3)	3830(2)	948(2)	49(1)
N(1)	7571(4)	5160(2)	5225(2)	54(1)
C(1)	6198(4)	5922(2)	2622(2)	36(1)
C(2)	4716(4)	5917(2)	1766(2)	32(1)
C(3)	3626(4)	4852(2)	1254(2)	30(1)
C(4)	1379(4)	4794(2)	1349(2)	34(1)
C(5)	1530(4)	2948(2)	527(2)	46(1)
C(6)	3461(4)	2655(2)	1333(2)	42(1)
C(7)	4817(4)	3735(2)	1676(2)	31(1)
C(8)	5486(4)	3801(2)	2868(2)	33(1)
C(9)	6982(4)	4820(2)	3202(2)	33(1)
C(10)	7330(4)	5016(2)	4345(2)	38(1)

Table S8. Bond lengths [Å] and angles [°] for **4k**.

Br(1)-C(2)	1.919(3)
O(1)-C(4)	1.206(3)
O(2)-C(4)	1.354(3)
O(2)-C(5)	1.466(3)
N(1)-C(10)	1.151(4)
C(1)-C(2)	1.326(4)
C(1)-C(9)	1.520(4)
C(1)-H(1)	0.9300
C(2)-C(3)	1.513(4)
C(3)-C(4)	1.524(4)
C(3)-C(7)	1.555(4)
C(3)-H(9)	0.9800
C(5)-C(6)	1.516(4)
C(5)-H(3)	0.9700
C(5)-H(2)	0.9700
C(6)-C(7)	1.550(4)
C(6)-H(10)	0.9700
C(6)-H(4)	0.9700
C(7)-C(8)	1.541(4)
C(7)-H(5)	0.9800
C(8)-C(9)	1.544(4)
C(8)-H(7)	0.9700
C(8)-H(6)	0.9700
C(9)-C(10)	1.493(4)
C(9)-H(8)	0.9800
C(4)-O(2)-C(5)	117.4(2)
C(2)-C(1)-C(9)	122.3(2)
C(2)-C(1)-H(1)	118.8
C(9)-C(1)-H(1)	118.8

C(1)-C(2)-C(3)	125.3(2)
C(1)-C(2)-Br(1)	118.7(2)
C(3)-C(2)-Br(1)	115.93(19)
C(2)-C(3)-C(4)	112.3(2)
C(2)-C(3)-C(7)	111.2(2)
C(4)-C(3)-C(7)	111.3(2)
C(2)-C(3)-H(9)	107.2
C(4)-C(3)-H(9)	107.2
C(7)-C(3)-H(9)	107.2
O(1)-C(4)-O(2)	119.5(2)
O(1)-C(4)-C(3)	125.6(2)
O(2)-C(4)-C(3)	114.9(2)
O(2)-C(5)-C(6)	109.1(2)
O(2)-C(5)-H(3)	109.9
C(6)-C(5)-H(3)	109.9
O(2)-C(5)-H(2)	109.9
C(6)-C(5)-H(2)	109.9
H(3)-C(5)-H(2)	108.3
C(5)-C(6)-C(7)	111.9(2)
C(5)-C(6)-H(10)	109.2
C(7)-C(6)-H(10)	109.2
C(5)-C(6)-H(4)	109.2
C(7)-C(6)-H(4)	109.2
H(10)-C(6)-H(4)	107.9
C(8)-C(7)-C(6)	111.0(2)
C(8)-C(7)-C(3)	109.6(2)
C(6)-C(7)-C(3)	110.5(2)
C(8)-C(7)-H(5)	108.6
C(6)-C(7)-H(5)	108.6
C(3)-C(7)-H(5)	108.6
C(7)-C(8)-C(9)	110.7(2)

C(7)-C(8)-H(7)	109.5
C(9)-C(8)-H(7)	109.5
C(7)-C(8)-H(6)	109.5
C(9)-C(8)-H(6)	109.5
H(7)-C(8)-H(6)	108.1
C(10)-C(9)-C(1)	110.0(2)
C(10)-C(9)-C(8)	110.6(2)
C(1)-C(9)-C(8)	111.7(2)
C(10)-C(9)-H(8)	108.1
C(1)-C(9)-H(8)	108.1
C(8)-C(9)-H(8)	108.1
N(1)-C(10)-C(9)	179.0(3)

Symmetry transformations used to generate equivalent atoms:

Table S9. Anisotropic displacement parameters ($Å^2$x 10^3) for **5q**.

The anisotropic displacement factor exponent takes the form: $-2p^2[h^2 a^{*2}U^{11} + ... + 2 h k a^* b^* U^{12}]$

	U^{11}	U^{22}	U^{33}	U^{23}	U^{13}	U^{12}
Br(1)	41(1)	33(1)	49(1)	12(1)	7(1)	1(1)
O(1)	38(1)	44(1)	59(1)	-8(1)	20(1)	4(1)
O(2)	34(1)	48(1)	64(1)	-14(1)	8(1)	-7(1)
N(1)	58(2)	51(2)	46(2)	-8(1)	-2(1)	-3(1)
C(1)	28(2)	33(2)	45(2)	4(1)	4(1)	-5(1)
C(2)	27(2)	28(1)	42(2)	8(1)	14(1)	4(1)
C(3)	28(2)	36(1)	26(2)	4(1)	8(1)	5(1)
C(4)	30(2)	40(2)	31(2)	3(1)	4(1)	-1(1)
C(5)	46(2)	39(2)	52(2)	-14(1)	6(2)	-1(1)
C(6)	49(2)	34(2)	41(2)	-5(1)	6(1)	0(1)
C(7)	30(2)	31(1)	32(2)	-1(1)	9(1)	4(1)
C(8)	34(2)	29(1)	36(2)	3(1)	7(1)	3(1)
C(9)	24(1)	38(1)	36(2)	3(1)	4(1)	2(1)
C(10)	28(2)	33(2)	49(2)	-1(1)	-1(1)	-2(1)

Table S10. Hydrogen coordinates (x 10^4) and isotropic displacement parameters (Å^{2}x 10^3) for **4k**.

	x	y	z	U(eq)
H(1)	6778	6624	2876	43
H(9)	3624	4901	513	36
H(3)	1905	3235	-98	56
H(2)	691	2261	348	56
H(10)	4260	2087	1049	50
H(4)	3069	2316	1934	50
H(5)	6057	3682	1389	37
H(7)	6159	3085	3133	40
H(6)	4278	3898	3162	40
H(8)	8311	4622	3037	40

ORTEP and x-ray data of 7a (CCDC 2386155)

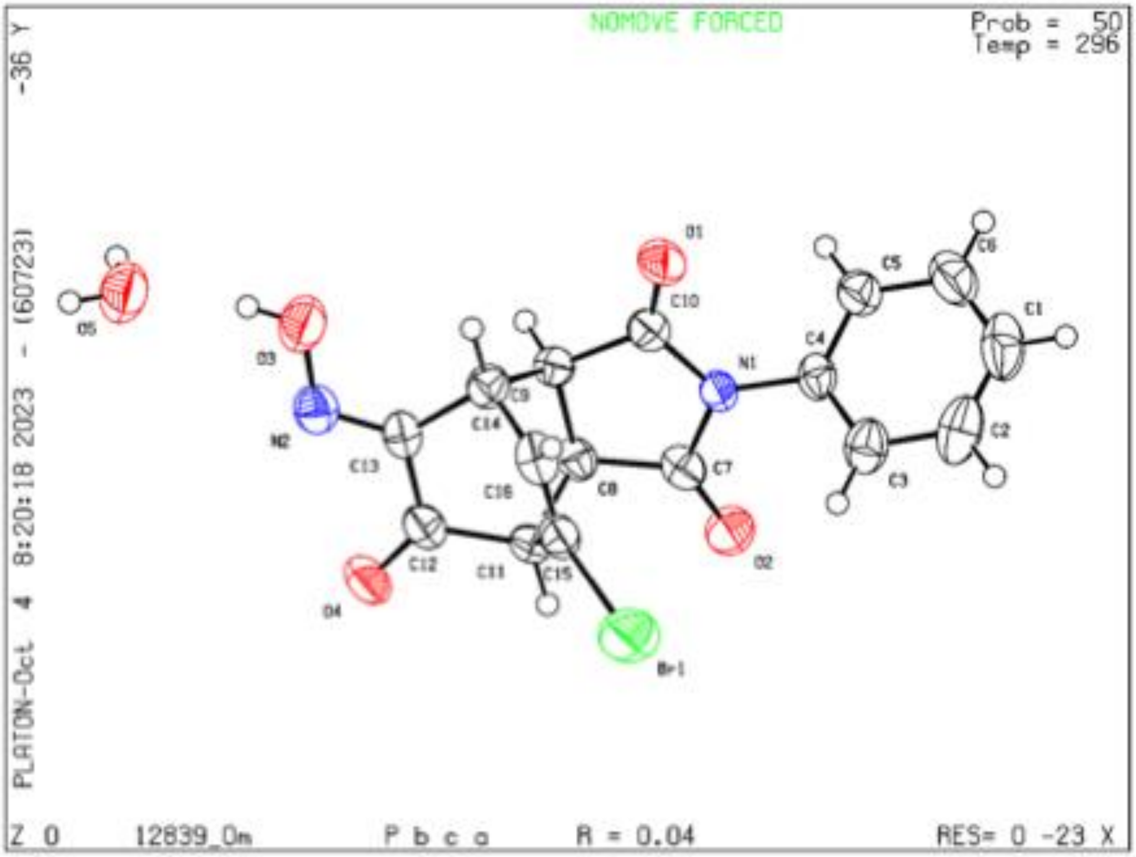

Table S11. Crystal data and structure refinement for 7a

Identification code	12839_0m	
Empirical formula	C16 H13 Br N2 O5	
Formula weight	393.19	
Temperature	296(2) K	
Wavelength	0.71073 Å	
Crystal system	Orthorhombic	
Space group	Pbca	
Unit cell dimensions	a = 7.545(2) Å	a= 90°.
	b = 15.291(4) Å	b= 90°.
	c = 27.516(8) Å	g = 90°.
Volume	3174.5(16) $Å^3$	
Z	8	
Density (calculated)	1.645 Mg/m^3	
Absorption coefficient	2.619 mm^{-1}	
F(000)	1584	
Crystal size	0.300 x 0.100 x 0.100 mm^3	
Theta range for data collection	1.480 to 28.367°.	
Index ranges	-10<=h<=10, -20<=k<=20, -34<=l<=36	
Reflections collected	69204	
Independent reflections	3953 [R(int) = 0.1350]	
Completeness to theta = 25.242°	100.0 %	
Absorption correction	None	
Refinement method	Full-matrix least-squares on F^2	
Data / restraints / parameters	3953 / 0 / 217	
Goodness-of-fit on F^2	1.003	
Final R indices [I>2sigma(I)]	R1 = 0.0449, wR2 = 0.0728	
R indices (all data)	R1 = 0.1355, wR2 = 0.0938	
Extinction coefficient	n/a	
Largest diff. peak and hole	0.303 and -0.511 e.$Å^{-3}$	

Table S12. Atomic coordinates (x 10^4) and equivalent isotropic displacement parameters ($Å^2$x 10^3) for 7a. U(eq) is defined as one third of the trace of the orthogonalized U^{ij} tensor.

	x	y	z	U(eq)
Br(1)	1034(1)	-163(1)	2041(1)	78(1)
O(1)	2310(3)	2874(1)	1099(1)	58(1)
O(2)	5211(3)	265(2)	1202(1)	54(1)
O(3)	-3196(3)	1577(2)	239(1)	62(1)
O(4)	-778(3)	-820(1)	463(1)	54(1)
O(5)	-5478(3)	1339(2)	-470(1)	67(1)
N(1)	4094(3)	1668(2)	1207(1)	35(1)
N(2)	-2485(4)	751(2)	280(1)	45(1)
C(1)	7646(5)	2858(3)	2163(2)	66(1)
C(2)	6737(5)	2124(3)	2304(1)	64(1)
C(3)	5576(4)	1717(2)	1990(1)	49(1)
C(4)	5344(4)	2066(2)	1533(1)	36(1)
C(5)	6261(4)	2796(2)	1385(1)	46(1)
C(6)	7424(5)	3193(2)	1706(2)	61(1)
C(7)	4124(4)	792(2)	1072(1)	38(1)
C(8)	2560(4)	621(2)	742(1)	36(1)
C(9)	1629(4)	1507(2)	689(1)	35(1)
C(10)	2668(4)	2126(2)	1012(1)	40(1)
C(11)	1291(4)	-52(2)	980(1)	38(1)
C(12)	-303(4)	-139(2)	645(1)	39(1)
C(13)	-1162(4)	723(2)	573(1)	39(1)
C(14)	-323(4)	1440(2)	867(1)	41(1)
C(15)	587(4)	371(2)	1437(1)	41(1)
C(16)	-246(4)	1115(2)	1385(1)	43(1)

Table S13. Bond lengths [Å] and angles [°] for 7a.

Br(1)-C(15)	1.884(3)
O(1)-C(10)	1.200(3)
O(2)-C(7)	1.205(3)
O(3)-N(2)	1.377(3)
O(3)-H(2A)	0.8200
O(4)-C(12)	1.210(3)
O(5)-H(5A)	0.8500
O(5)-H(5B)	0.8499
N(1)-C(7)	1.390(4)
N(1)-C(10)	1.392(4)
N(1)-C(4)	1.437(4)
N(2)-C(13)	1.283(4)
C(1)-C(6)	1.367(5)
C(1)-C(2)	1.371(5)
C(1)-H(1B)	0.9300
C(2)-C(3)	1.379(5)
C(2)-H(2B)	0.9300
C(3)-C(4)	1.377(4)
C(3)-H(3A)	0.9300
C(4)-C(5)	1.374(4)
C(5)-C(6)	1.386(4)
C(5)-H(8A)	0.9300
C(6)-H(4A)	0.9300
C(7)-C(8)	1.512(4)
C(8)-C(9)	1.534(4)
C(8)-C(11)	1.551(4)
C(8)-H(9A)	0.9800
C(9)-C(10)	1.515(4)
C(9)-C(14)	1.556(4)
C(9)-H(11A)	0.9800
C(11)-C(15)	1.509(4)
C(11)-C(12)	1.523(4)
C(11)-H(13A)	0.9800
C(12)-C(13)	1.481(4)

C(13)-C(14)	1.503(4)
C(14)-C(16)	1.512(4)
C(14)-H(6A)	0.9800
C(15)-C(16)	1.307(4)
C(16)-H(12A)	0.9300
N(2)-O(3)-H(2A)	109.5
H(5A)-O(5)-H(5B)	108.6
C(7)-N(1)-C(10)	113.2(3)
C(7)-N(1)-C(4)	124.4(2)
C(10)-N(1)-C(4)	122.3(2)
C(13)-N(2)-O(3)	112.7(3)
C(6)-C(1)-C(2)	120.4(4)
C(6)-C(1)-H(1B)	119.8
C(2)-C(1)-H(1B)	119.8
C(1)-C(2)-C(3)	120.6(4)
C(1)-C(2)-H(2B)	119.7
C(3)-C(2)-H(2B)	119.7
C(4)-C(3)-C(2)	118.5(3)
C(4)-C(3)-H(3A)	120.7
C(2)-C(3)-H(3A)	120.7
C(5)-C(4)-C(3)	121.4(3)
C(5)-C(4)-N(1)	119.3(3)
C(3)-C(4)-N(1)	119.3(3)
C(4)-C(5)-C(6)	119.1(3)
C(4)-C(5)-H(8A)	120.5
C(6)-C(5)-H(8A)	120.5
C(1)-C(6)-C(5)	119.9(4)
C(1)-C(6)-H(4A)	120.0
C(5)-C(6)-H(4A)	120.0
O(2)-C(7)-N(1)	125.2(3)
O(2)-C(7)-C(8)	126.4(3)
N(1)-C(7)-C(8)	108.3(3)
C(7)-C(8)-C(9)	105.1(2)
C(7)-C(8)-C(11)	110.0(2)
C(9)-C(8)-C(11)	110.1(2)
C(7)-C(8)-H(9A)	110.5

C(9)-C(8)-H(9A)	110.5
C(11)-C(8)-H(9A)	110.5
C(10)-C(9)-C(8)	105.1(2)
C(10)-C(9)-C(14)	110.3(3)
C(8)-C(9)-C(14)	110.2(2)
C(10)-C(9)-H(11A)	110.4
C(8)-C(9)-H(11A)	110.4
C(14)-C(9)-H(11A)	110.4
O(1)-C(10)-N(1)	125.2(3)
O(1)-C(10)-C(9)	126.6(3)
N(1)-C(10)-C(9)	108.2(3)
C(15)-C(11)-C(12)	105.3(2)
C(15)-C(11)-C(8)	106.6(2)
C(12)-C(11)-C(8)	106.8(2)
C(15)-C(11)-H(13A)	112.6
C(12)-C(11)-H(13A)	112.6
C(8)-C(11)-H(13A)	112.6
O(4)-C(12)-C(13)	125.5(3)
O(4)-C(12)-C(11)	124.0(3)
C(13)-C(12)-C(11)	110.4(3)
N(2)-C(13)-C(12)	117.0(3)
N(2)-C(13)-C(14)	129.9(3)
C(12)-C(13)-C(14)	113.1(3)
C(13)-C(14)-C(16)	106.5(3)
C(13)-C(14)-C(9)	106.2(2)
C(16)-C(14)-C(9)	106.4(2)
C(13)-C(14)-H(6A)	112.4
C(16)-C(14)-H(6A)	112.4
C(9)-C(14)-H(6A)	112.4
C(16)-C(15)-C(11)	116.9(3)
C(16)-C(15)-Br(1)	124.0(3)
C(11)-C(15)-Br(1)	119.1(2)
C(15)-C(16)-C(14)	114.0(3)
C(15)-C(16)-H(12A)	123.0
C(14)-C(16)-H(12A)	123.0

Symmetry transformations used to generate equivalent atoms:

Table S14. Anisotropic displacement parameters ($Å^2$x 10^3) for 7a. The anisotropic displacement factor exponent takes the form: $-2p^2[h^2 a^{*2}U^{11} + ... + 2 h k a^* b^* U^{12}]$

	U^{11}	U^{22}	U^{33}	U^{23}	U^{13}	U^{12}
Br(1)	115(1)	73(1)	45(1)	16(1)	-11(1)	-22(1)
O(1)	58(2)	29(1)	86(2)	-9(1)	-20(1)	4(1)
O(2)	42(1)	42(1)	78(2)	-2(1)	-8(1)	11(1)
O(3)	58(2)	61(2)	69(2)	-5(1)	-21(1)	8(1)
O(4)	62(2)	42(1)	56(2)	-7(1)	-8(1)	-16(1)
O(5)	49(2)	86(2)	64(2)	10(1)	-10(1)	-3(1)
N(1)	31(1)	31(1)	43(2)	-2(1)	-5(1)	-2(1)
N(2)	40(2)	49(2)	47(2)	-1(1)	1(2)	-3(2)
C(1)	54(3)	72(3)	73(3)	-33(2)	-20(2)	8(2)
C(2)	51(2)	99(3)	42(2)	-11(2)	-8(2)	9(2)
C(3)	37(2)	68(2)	42(2)	0(2)	1(2)	-2(2)
C(4)	26(2)	40(2)	43(2)	-6(2)	-1(2)	2(2)
C(5)	42(2)	38(2)	58(2)	-2(2)	-6(2)	0(2)
C(6)	49(2)	43(2)	91(3)	-14(2)	-9(2)	-3(2)
C(7)	32(2)	35(2)	48(2)	-5(2)	8(2)	-1(2)
C(8)	37(2)	33(2)	37(2)	-6(1)	0(2)	-2(2)
C(9)	38(2)	34(2)	34(2)	1(1)	-1(2)	-2(2)
C(10)	38(2)	36(2)	47(2)	2(2)	-2(2)	-3(2)
C(11)	42(2)	26(2)	45(2)	-2(1)	-4(2)	-1(2)
C(12)	43(2)	40(2)	34(2)	-3(2)	2(2)	-9(2)
C(13)	34(2)	44(2)	38(2)	-4(2)	-2(2)	-6(2)
C(14)	35(2)	39(2)	48(2)	-2(2)	-4(2)	4(2)
C(15)	43(2)	44(2)	37(2)	2(2)	-3(2)	-12(2)
C(16)	36(2)	53(2)	41(2)	-11(2)	5(2)	-4(2)

Table S15. Hydrogen coordinates (x 10^4) and isotropic displacement parameters ($Å^2$x 10^3) for 7a.

	x	y	z	U(eq)
H(2A)	-3942	1581	22	94
H(5A)	-6566	1214	-507	80
H(5B)	-5050	1486	-743	80
H(1B)	8418	3129	2379	79
H(2B)	6906	1899	2615	77
H(3A)	4963	1217	2085	59
H(8A)	6103	3019	1074	55
H(4A)	8054	3688	1611	73
H(9A)	2964	409	424	43
H(11A)	1669	1705	351	42
H(13A)	1869	-614	1044	45
H(6A)	-961	1996	837	49
H(12A)	-746	1418	1645	52

ORTEP and x-ray data of 9c (CCDC 2393498)

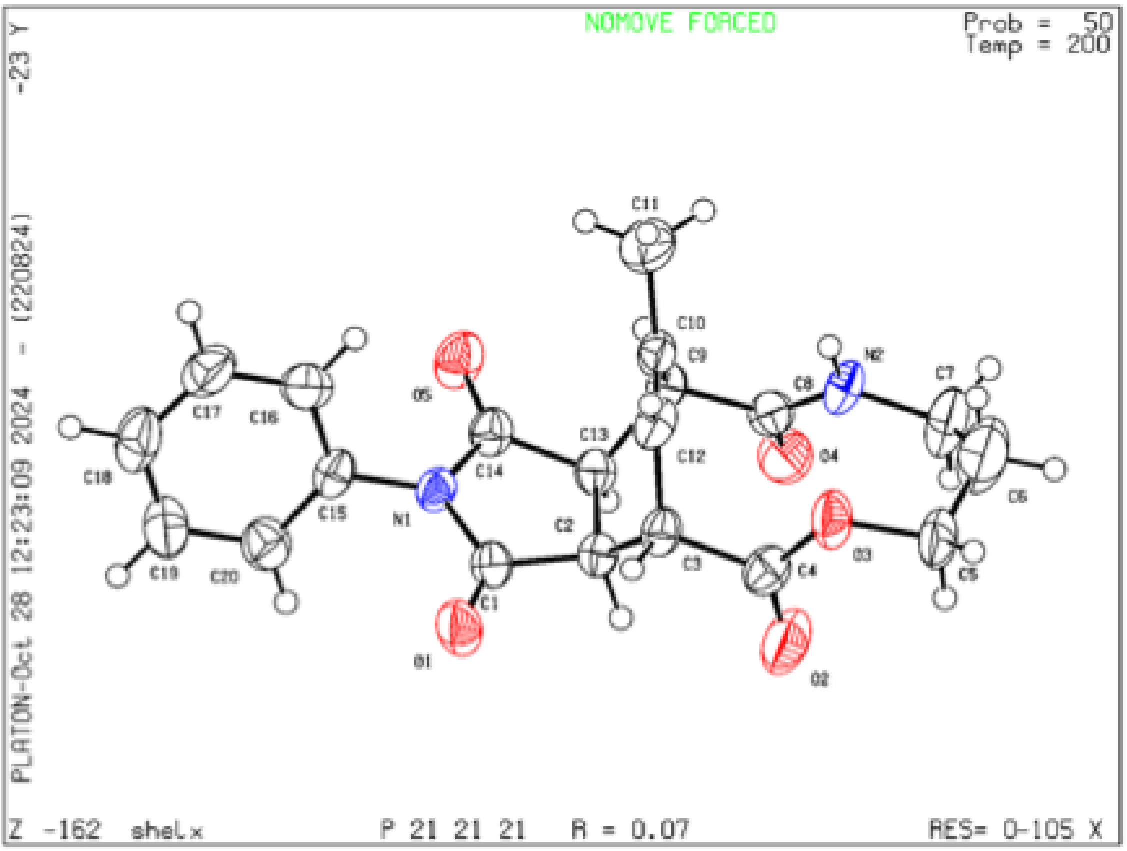

Table S16. Crystal data and structure refinement for 9c.

Identification code	d24994	
Empirical formula	C20 H20 N2 O5	
Formula weight	368.38	
Temperature	200(2) K	
Wavelength	0.71073 Å	
Crystal system	Orthorhombic	
Space group	P 21 21 21	
Unit cell dimensions	a = 10.6991(18) Å	a= 90°.
	b = 10.7558(18) Å	b= 90°.
	c = 15.421(3) Å	g = 90°.
Volume	1774.6(5) $Å^3$	
Z	4	
Density (calculated)	1.379 Mg/m^3	
Absorption coefficient	0.100 mm^{-1}	
F(000)	776	
Crystal size	0.27 x 0.08 x 0.04 mm^3	
Theta range for data collection	2.31 to 25.47°.	
Index ranges	-12<=h<=11, -12<=k<=12, -18<=l<=18	
Reflections collected	12473	
Independent reflections	3266 [R(int) = 0.0940]	
Completeness to theta = 25.47°	99.5 %	
Absorption correction	None	
Max. and min. transmission	0.9960 and 0.9735	
Refinement method	Full-matrix least-squares on F^2	
Data / restraints / parameters	3266 / 0 / 246	
Goodness-of-fit on F^2	1.033	
Final R indices [I>2sigma(I)]	R1 = 0.0695, wR2 = 0.1657	
R indices (all data)	R1 = 0.1093, wR2 = 0.1947	
Absolute structure parameter	-3(3)	
Extinction coefficient	0.064(7)	
Largest diff. peak and hole	0.690 and -0.323 e.$Å^{-3}$	

Table S17. Atomic coordinates (x 10^4) and equivalent isotropic displacement parameters ($Å^2$x 10^3) for 9c. U(eq) is defined as one third of the trace of the orthogonalized U^{ij} tensor.

	x	y	z	U(eq)
C(1)	4299(4)	4528(4)	4926(3)	41(1)
C(2)	3316(4)	3837(4)	5441(3)	39(1)
C(3)	2251(4)	4747(4)	5650(3)	40(1)
C(4)	1013(4)	4046(5)	5741(3)	46(1)
C(5)	-932(5)	3966(6)	6513(3)	61(2)
C(6)	-882(6)	3634(8)	7421(5)	107(3)
C(7)	117(5)	2799(5)	7754(4)	66(2)
C(8)	2358(4)	2839(4)	7328(3)	42(1)
C(9)	3461(4)	3691(4)	7122(3)	37(1)
C(10)	3162(4)	5067(4)	7116(3)	41(1)
C(11)	3560(5)	5801(5)	7882(4)	68(2)
C(12)	2582(4)	5544(4)	6423(3)	41(1)
C(13)	4022(4)	3346(4)	6237(3)	37(1)
C(14)	5301(4)	3944(4)	6179(3)	40(1)
C(15)	6465(4)	5283(4)	5135(3)	39(1)
C(16)	6857(5)	6298(5)	5615(3)	51(1)
C(17)	7894(5)	6945(5)	5357(4)	59(2)
C(18)	8552(5)	6595(5)	4634(4)	63(2)
C(19)	8146(5)	5597(5)	4138(4)	62(2)
C(20)	7095(4)	4909(5)	4402(3)	52(1)
N(1)	5382(3)	4603(3)	5405(2)	35(1)
N(2)	1343(3)	3395(3)	7654(3)	44(1)
O(1)	4156(3)	4986(3)	4208(2)	60(1)
O(2)	784(3)	3097(4)	5358(3)	73(1)
O(3)	216(3)	4642(3)	6256(2)	54(1)
O(4)	2434(3)	1716(3)	7212(2)	60(1)
O(5)	6126(3)	3897(3)	6716(2)	57(1)

Table S18. Bond lengths [Å] and angles [°] for 9c.

C(1)-O(1)	1.221(6)
C(1)-N(1)	1.376(6)
C(1)-C(2)	1.513(6)
C(2)-C(3)	1.536(6)
C(2)-C(13)	1.536(6)
C(2)-H(2)	1.0000
C(3)-C(12)	1.511(6)
C(3)-C(4)	1.530(6)
C(3)-H(3)	1.0000
C(4)-O(2)	1.205(6)
C(4)-O(3)	1.330(6)
C(5)-C(6)	1.446(8)
C(5)-O(3)	1.481(6)
C(5)-H(5A)	0.9900
C(5)-H(5B)	0.9900
C(6)-C(7)	1.487(9)
C(6)-H(6A)	0.9900
C(6)-H(6B)	0.9900
C(7)-N(2)	1.468(6)
C(7)-H(7A)	0.9900
C(7)-H(7B)	0.9900
C(8)-O(4)	1.224(5)
C(8)-N(2)	1.337(6)
C(8)-C(9)	1.528(6)
C(9)-C(10)	1.515(6)
C(9)-C(13)	1.535(6)
C(9)-H(9)	1.0000
C(10)-C(12)	1.338(6)
C(10)-C(11)	1.483(6)
C(11)-H(11A)	0.9800
C(11)-H(11B)	0.9800
C(11)-H(11C)	0.9800
C(12)-H(12)	0.9500
C(13)-C(14)	1.515(6)

C(13)-H(13)	1.0000
C(14)-O(5)	1.211(5)
C(14)-N(1)	1.390(6)
C(15)-C(20)	1.377(6)
C(15)-C(16)	1.384(6)
C(15)-N(1)	1.432(5)
C(16)-C(17)	1.370(7)
C(16)-H(16)	0.9500
C(17)-C(18)	1.371(8)
C(17)-H(17)	0.9500
C(18)-C(19)	1.387(8)
C(18)-H(18)	0.9500
C(19)-C(20)	1.407(7)
C(19)-H(19)	0.9500
C(20)-H(20)	0.9500
N(2)-H(2A)	0.8800

O(1)-C(1)-N(1)	124.7(4)
O(1)-C(1)-C(2)	125.9(4)
N(1)-C(1)-C(2)	109.4(4)
C(1)-C(2)-C(3)	108.3(4)
C(1)-C(2)-C(13)	104.3(3)
C(3)-C(2)-C(13)	114.6(4)
C(1)-C(2)-H(2)	109.8
C(3)-C(2)-H(2)	109.8
C(13)-C(2)-H(2)	109.8
C(12)-C(3)-C(4)	114.2(4)
C(12)-C(3)-C(2)	110.7(4)
C(4)-C(3)-C(2)	110.4(4)
C(12)-C(3)-H(3)	107.1
C(4)-C(3)-H(3)	107.1
C(2)-C(3)-H(3)	107.1
O(2)-C(4)-O(3)	124.8(5)
O(2)-C(4)-C(3)	123.3(5)
O(3)-C(4)-C(3)	111.8(4)
C(6)-C(5)-O(3)	110.5(5)

C(6)-C(5)-H(5A)	109.6
O(3)-C(5)-H(5A)	109.6
C(6)-C(5)-H(5B)	109.6
O(3)-C(5)-H(5B)	109.6
H(5A)-C(5)-H(5B)	108.1
C(5)-C(6)-C(7)	120.7(7)
C(5)-C(6)-H(6A)	107.2
C(7)-C(6)-H(6A)	107.2
C(5)-C(6)-H(6B)	107.2
C(7)-C(6)-H(6B)	107.2
H(6A)-C(6)-H(6B)	106.8
N(2)-C(7)-C(6)	110.0(4)
N(2)-C(7)-H(7A)	109.7
C(6)-C(7)-H(7A)	109.7
N(2)-C(7)-H(7B)	109.7
C(6)-C(7)-H(7B)	109.7
H(7A)-C(7)-H(7B)	108.2
O(4)-C(8)-N(2)	123.4(4)
O(4)-C(8)-C(9)	120.7(4)
N(2)-C(8)-C(9)	115.9(4)
C(10)-C(9)-C(8)	115.1(3)
C(10)-C(9)-C(13)	108.3(4)
C(8)-C(9)-C(13)	110.0(3)
C(10)-C(9)-H(9)	107.7
C(8)-C(9)-H(9)	107.7
C(13)-C(9)-H(9)	107.7
C(12)-C(10)-C(11)	124.4(4)
C(12)-C(10)-C(9)	118.5(4)
C(11)-C(10)-C(9)	117.1(4)
C(10)-C(11)-H(11A)	109.5
C(10)-C(11)-H(11B)	109.5
H(11A)-C(11)-H(11B)	109.5
C(10)-C(11)-H(11C)	109.5
H(11A)-C(11)-H(11C)	109.5
H(11B)-C(11)-H(11C)	109.5
C(10)-C(12)-C(3)	121.4(4)

C(10)-C(12)-H(12)	119.3
C(3)-C(12)-H(12)	119.3
C(14)-C(13)-C(9)	107.7(4)
C(14)-C(13)-C(2)	104.5(3)
C(9)-C(13)-C(2)	115.8(3)
C(14)-C(13)-H(13)	109.5
C(9)-C(13)-H(13)	109.5
C(2)-C(13)-H(13)	109.5
O(5)-C(14)-N(1)	124.2(4)
O(5)-C(14)-C(13)	126.8(4)
N(1)-C(14)-C(13)	108.9(4)
C(20)-C(15)-C(16)	121.4(4)
C(20)-C(15)-N(1)	119.1(4)
C(16)-C(15)-N(1)	119.5(4)
C(17)-C(16)-C(15)	119.4(5)
C(17)-C(16)-H(16)	120.3
C(15)-C(16)-H(16)	120.3
C(16)-C(17)-C(18)	120.9(5)
C(16)-C(17)-H(17)	119.6
C(18)-C(17)-H(17)	119.6
C(17)-C(18)-C(19)	120.0(5)
C(17)-C(18)-H(18)	120.0
C(19)-C(18)-H(18)	120.0
C(18)-C(19)-C(20)	119.9(5)
C(18)-C(19)-H(19)	120.1
C(20)-C(19)-H(19)	120.1
C(15)-C(20)-C(19)	118.4(5)
C(15)-C(20)-H(20)	120.8
C(19)-C(20)-H(20)	120.8
C(1)-N(1)-C(14)	112.3(4)
C(1)-N(1)-C(15)	123.7(4)
C(14)-N(1)-C(15)	124.1(4)
C(8)-N(2)-C(7)	124.7(4)
C(8)-N(2)-H(2A)	117.7
C(7)-N(2)-H(2A)	117.7
C(4)-O(3)-C(5)	117.0(4)

Symmetry transformations used to generate equivalent atoms:

Table S19. Anisotropic displacement parameters (Å^{2}x 10^3)for d24994. The anisotropic displacement factor exponent takes the form: -2p^2[h^{2}a*^{2}U^{11} + ... + 2 h k a* b* U^{12}]

	U^{11}	U^{22}	U^{33}	U^{23}	U^{13}	U^{12}
C(1)	32(2)	48(3)	43(3)	-3(2)	4(2)	3(2)
C(2)	31(2)	46(2)	40(2)	-1(2)	3(2)	-2(2)
C(3)	28(2)	43(2)	48(3)	4(2)	-1(2)	2(2)
C(4)	34(3)	50(3)	53(3)	0(3)	-6(2)	5(2)
C(5)	30(3)	87(4)	67(4)	5(3)	7(3)	-8(3)
C(6)	60(4)	156(7)	103(6)	38(5)	-9(4)	-43(5)
C(7)	37(3)	72(4)	90(4)	25(4)	-2(3)	-13(3)
C(8)	47(3)	40(2)	38(3)	6(2)	-2(2)	-8(2)
C(9)	31(2)	38(2)	42(2)	2(2)	0(2)	0(2)
C(10)	34(2)	39(2)	50(3)	-10(2)	10(2)	-8(2)
C(11)	65(4)	65(4)	74(4)	-24(3)	6(3)	-14(3)
C(12)	30(2)	29(2)	63(3)	-5(2)	0(2)	2(2)
C(13)	32(2)	34(2)	43(3)	1(2)	3(2)	4(2)
C(14)	34(2)	41(2)	45(3)	5(2)	-1(2)	3(2)
C(15)	30(2)	39(2)	49(3)	7(2)	-4(2)	-1(2)
C(16)	56(3)	53(3)	43(3)	-3(2)	-2(3)	-5(3)
C(17)	60(3)	58(3)	59(3)	7(3)	-12(3)	-20(3)
C(18)	47(3)	67(3)	75(4)	26(3)	-1(3)	-14(3)
C(19)	48(3)	70(4)	67(4)	3(3)	15(3)	-6(3)
C(20)	44(3)	53(3)	58(3)	-7(2)	5(2)	5(2)
N(1)	27(2)	39(2)	40(2)	-1(2)	-2(2)	1(2)
N(2)	32(2)	39(2)	61(3)	6(2)	-2(2)	-11(2)
O(1)	42(2)	96(3)	42(2)	19(2)	-1(2)	-1(2)
O(2)	40(2)	75(3)	104(3)	-23(2)	7(2)	-10(2)
O(3)	30(2)	68(2)	64(2)	2(2)	8(2)	1(2)
O(4)	69(2)	48(2)	62(2)	-4(2)	6(2)	-11(2)
O(5)	38(2)	75(2)	58(2)	21(2)	-14(2)	-3(2)

Table S20. Hydrogen coordinates (x 10^4) and isotropic displacement parameters ($Å^2$x 10^3) for 9c.

	x	y	z	U(eq)
H(2)	2987	3125	5092	47
H(3)	2164	5316	5141	47
H(5A)	-1671	4497	6405	73
H(5B)	-1017	3204	6158	73
H(6A)	-1693	3245	7569	128
H(6B)	-830	4418	7755	128
H(7A)	-35	2614	8374	80
H(7B)	104	2005	7429	80
H(9)	4118	3546	7572	44
H(11A)	3251	6656	7826	101
H(11B)	4475	5809	7915	101
H(11C)	3217	5425	8409	101
H(12)	2374	6403	6417	49
H(13)	4108	2422	6195	44
H(16)	6410	6543	6119	61
H(17)	8162	7645	5683	71
H(18)	9285	7035	4472	76
H(19)	8577	5380	3621	74
H(20)	6826	4204	4082	62
H(2A)	1416	4174	7820	53
